# Controlled Self-Immolative Release of β‑Lapachone via an Optimized *para*-Hydroxybenzyl Linker for Targeted Pancreatic Cancer Therapy

**DOI:** 10.1021/jacs.5c17598

**Published:** 2025-12-15

**Authors:** Julie B. Becher, Nisita Dutta, Claudio D. Navo, Lavinia Dunsmore, Roman Misteli, Enrique Gil de Montes, Grant G. Simpson, Christine C. Alewine, Gonzalo Jiménez-Osés, Gonçalo J. L. Bernardes

**Affiliations:** † Yusuf Hamied Department of Chemistry, 2152 University of Cambridge, Cambridge CB2 1EW, U.K.; ‡ Laboratory of Molecular Biology, National Cancer Institute, National Institutes of Health, Bethesda, Maryland 20892, United States; § Medical Scientist Training Program, University of Maryland School of Medicine, Baltimore, Maryland 21201, United States; ∥ Center for Cooperative Research in Biosciences (CIC bioGUNE), 73038 Basque Research and Technology Alliance (BRTA), Derio 48160, Spain; ⊥ Ikerbasque, Basque Foundation for Science, Bilbao 48013, Spain; # Translational Chemical Biology Group, Spanish National Cancer Research Centre 26 (CNIO), Madrid 28029, Spain

## Abstract

Methodologies to target cancer cells without affecting healthy surrounding cells have the potential to vastly improve both patient outcomes and quality of life. *ortho*-Quinone natural products such as β-lapachone have exhibited great therapeutic potential, but their clinical use has been limited thus far due to systemic toxicity, making them ideal candidates for targeted prodrug development. Previously developed β-lapachone prodrugs suffered from suboptimal release rates, low solubility, and poor tumor specificity, which have hindered their advancement into the clinic. This work explores the development of an optimized β-lapachone small molecule prodrug platform that utilizes a β-glucuronide-protected *para*-hydroxybenzyl (PHB) moiety alkylated to one of β-lapachone’s carbonyls via an indium-mediated Barbier-type reaction. The β-glucuronide moiety detoxifies and solubilizes the *ortho*-quinone systemically and triggers specific release in the tumor microenvironment (TME). Additionally, the drug release rate is largely controlled by pH and can be fine-tuned via the addition of electron-donating/withdrawing groups to the PHB self-immolative linker. The prodrug is rationally designed to have numerous layers of pancreatic cancer targeting via the tumor-selective overexpression of the key β-lapachone activating enzymes NQO1 and 5-LO, the TME expression pattern of the release-triggering enzyme β-glucuronidase, and the opportunity for modular attachment to carrier systems targeting pancreatic cancer membrane proteins. Various derivatives of this prodrug have shown promising stability, release, and efficacy against pancreatic cancer cell lines. This unique prodrug platform allows us to mask the toxicity of an *ortho*-quinone payload and utilize several layers of tumor specificity in the hope of effectively treating pancreatic cancer patients while minimizing toxic side effects.

## Graphical abstract

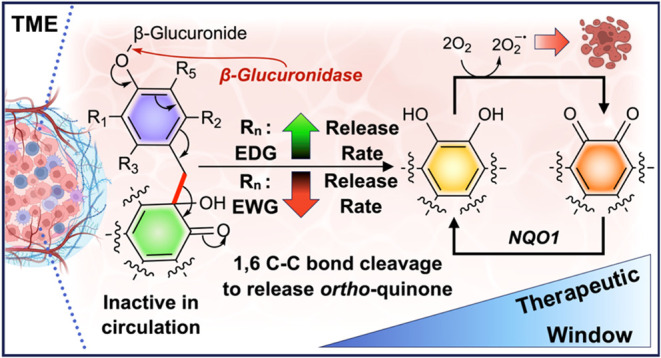

## Introduction

The *ortho*-quinone functional group is a natural product scaffold that has underutilized cancer treatment potential. *ortho*-Quinones have repeatedly exhibited cytotoxicity against numerous cancer cell lines,^1–6^ but have been excluded from clinical development due to both pan-assay interference (PAINS) character and dose-limiting systemic toxicities caused by indiscriminate mechanisms of action. Many metabolic processes are known to reduce quinones to either their semiquinone or hydroquinone forms.^7,8^ In contrast to clinically relevant *para*-quinones, for many *ortho*-quinones, these reduced forms are highly unstable and spontaneously undergo reoxidation. This futile redox cycling generates reactive oxygen species (ROS) that damage DNA, lipids, and proteins, consume reducing substrates (glutathione, NAD­(P)­H), and induce cellular oxidative stress.^8,9^ In order to combat the toxic systemic effects of *ortho*-quinones, specialized prodrug formats can be synthesized that facilitate the highly specific release of these payloads in the tumor microenvironment (TME), allowing for effective dosing with limited clinical side effects for patients suffering from various cancers.

While *ortho*-quinones show promising anticancer activity against multiple diseases, one with a large unmet therapeutic need is pancreatic ductal adenocarcinoma (PDAC). PDAC is a major oncologic disease that is currently the third-leading cause of cancer-related deaths in the U.S., with a five-year survival rate of just 8% in 2025.^10^ The lack of improvement in PDAC patient survival rates over the decades is, in part, due to the limited effective therapies for this fatal disease. Current first-line chemotherapy regimens are substantially toxic, with neutropenia occurring in about 40–50% of patients.^11–15^

Additionally, due to the dense stroma of the PDAC TME, large molecule therapeutics have had little efficacy in penetrating these tumors and eliminating cancer cells.^16^ Thus, new, effective small-molecule therapeutics are desperately needed to improve survival rates and preserve the quality of life for PDAC patients.

The *ortho*-quinone natural product β-lapachone (**1**) originates from the bark of the Central and South American Lapacho tree.^17^ This small molecule (MW = 242) has been shown to have micro- to submicromolar cytotoxicity against a range of cancer cell lines including colon,^18^ breast,^18–22^ NSCLC,^17,23^ prostate,^4,20,24^ and of particular interest, PDAC.^19,25–29^ β-Lapachone’s mechanism of action involves hijacking NAD­(P)­H:quinone oxidoreductase 1’s (NQO1) natural cytoprotective function to kill the malignant cells that overexpress this enzyme (Figure S1).^21^ When β-lapachone is reduced by NQO1 into its hydroquinone form (**2**), its instability causes it to swiftly and spontaneously reoxidize back to the *ortho*-quinone.^17,21^ Each step of the reoxidation process generates ROS, and further interaction with NQO1 perpetuates a futile redox cycle. This allows for a small amount of the *ortho*-quinone molecule to cause drastic oxidative stress to cells.^23^ Eventually, this process leads to a unique pattern of cell death termed “programmed necrosis” or “necroptosis” (Figure S1).^17^ Importantly, this method of cell death does not rely on caspase or oncogenic driver mutation (e.g., KRAS, p53) pathways that are often defunct in cancer cells.^17,27^ In addition, β-lapachone kills cells regardless of their cell cycle status, making it effective against both actively dividing and growth-arrested stem-like malignant cells.^27,30^ This manner of cytotoxicity could be beneficial to overcoming cancer drug resistance by breaking the 1:1 drug to target ratio.^27,31^

For PDAC, >90% of cases exhibit NQO1 overexpression due to mutant KRAS-driven transcription.^26,27,32,33^ Importantly, NQO1 expression is correlated to both disease progression and poor patient prognosis in pancreatic cancer as well as various other cancers.^27,34–38^ Beyond causing oxidative stress, β-lapachone has also been identified as an inhibitor for the 5-lipoxygenase (5-LO) enzyme,^39^ which is overexpressed in >90% of PDAC cases.^40–46^ 5-LO is part of the arachidonic acid pathway that is implicated in inflammation-associated carcinogenesis for numerous cancers including PDAC.^43,45,47–49^ Additionally, there is a correlation between increased 5-LO expression and PDAC disease progression.^42,46^

β-Lapachone has two main shortcomings—insolubility in water^50^ and systemic dose-limiting toxicity.^51^ It advanced to clinical trials for PDAC in two solubilizing formulations, ARQ 501 (phase II)^52^ and ARQ 761 (phase I/Ib),^51,53^ which both showed moderate efficacy by stabilizing disease in patients. However, major dose-limiting side effects of anemia and methemoglobinemia were observed due to off-target redox cycling of the *ortho-*quinone pharmacophore by interaction with the b5 reductase 1 (CYB5R1) enzyme expressed ubiquitously in mammalian erythrocyte cells.^51^ Thus, additional layers of target specificity must be added to β-lapachone to clinically utilize the *ortho*-quinone scaffold. Previous β-lapachone prodrugs developed by Boothman, Gao, and co-workers involved esterase-cleavable hydroquinone alkyl esters,^54,55^ and pH-sensitive aryl imine,^56^ acyl hydrazone, ketal,^57^ aminoalkyl alcohol and amino aromatic phenol derivatives.^58^ However, these prodrug strategies failed to advance to clinic due to their drawbacks—esters and hydrazones are too labile under physiological conditions in circulation, while ketals are not sufficiently labile in tumors.^57^

A more sophisticated *ortho*-quinone prodrug strategy was developed in our group using a targeted carrier-linked prodrug scaffold with a site-specific trigger–spacer–drug design.^59^ The trigger and spacer can then be designed to improve the low solubility of the drug through the addition of polar or ionizable groups. Dunsmore’s first-generation prodrug attached a *para*-aminobenzyl (PAB) self-immolative linker (SIL) via C-alkylation to one of the *ortho*-quinone carbonyls to form a *para*-aminobenzyl ketol species (Figure 1a). While the PAB linker was attached, the prodrug exhibited no redox activity, decreased hemolysis, and decreased methemoglobinemia formation in *in vitro* assays, indicating that this C-alkylation strategy is successful at stopping the off-target toxicity of the pharmacophore in circulation. Upon enzymatic or chemical cleavage of a protecting group from the PAB linker, the *ortho*-quinone was released via a C–C bond cleaving 1,6-elimination mechanism.^59^ While this first-generation prodrug represented a breakthrough in *ortho*-quinone prodrug technology, it exhibited suboptimal solubility and slow release rates at physiologically relevant pHs. A similar boronate ester β-lapachone prodrug (Figure 1b) was developed by the Zhang group, but relied on ROS as the trigger for prodrug activation rather than a tumor-specific enzyme.^60^ We hypothesized that switching the peptide-protected PAB linker to a β-glucuronide-protected *para*-hydroxybenzyl (PHB) self-immolative linker would facilitate both faster and more controlled drug release at physiologically relevant pHs via the much lower p*K*_a_ of the phenol in comparison to that of the PAB aniline and increased solubility via the charged, hydrophilic β-glucuronide trigger moiety (Figure 1c). Our novel prodrug approach addresses several limitations of its predecessors, improving solubility, minimizing off-target toxicity, and increasing the specificity of prodrug activation to target tissues.

**Figure 1:**
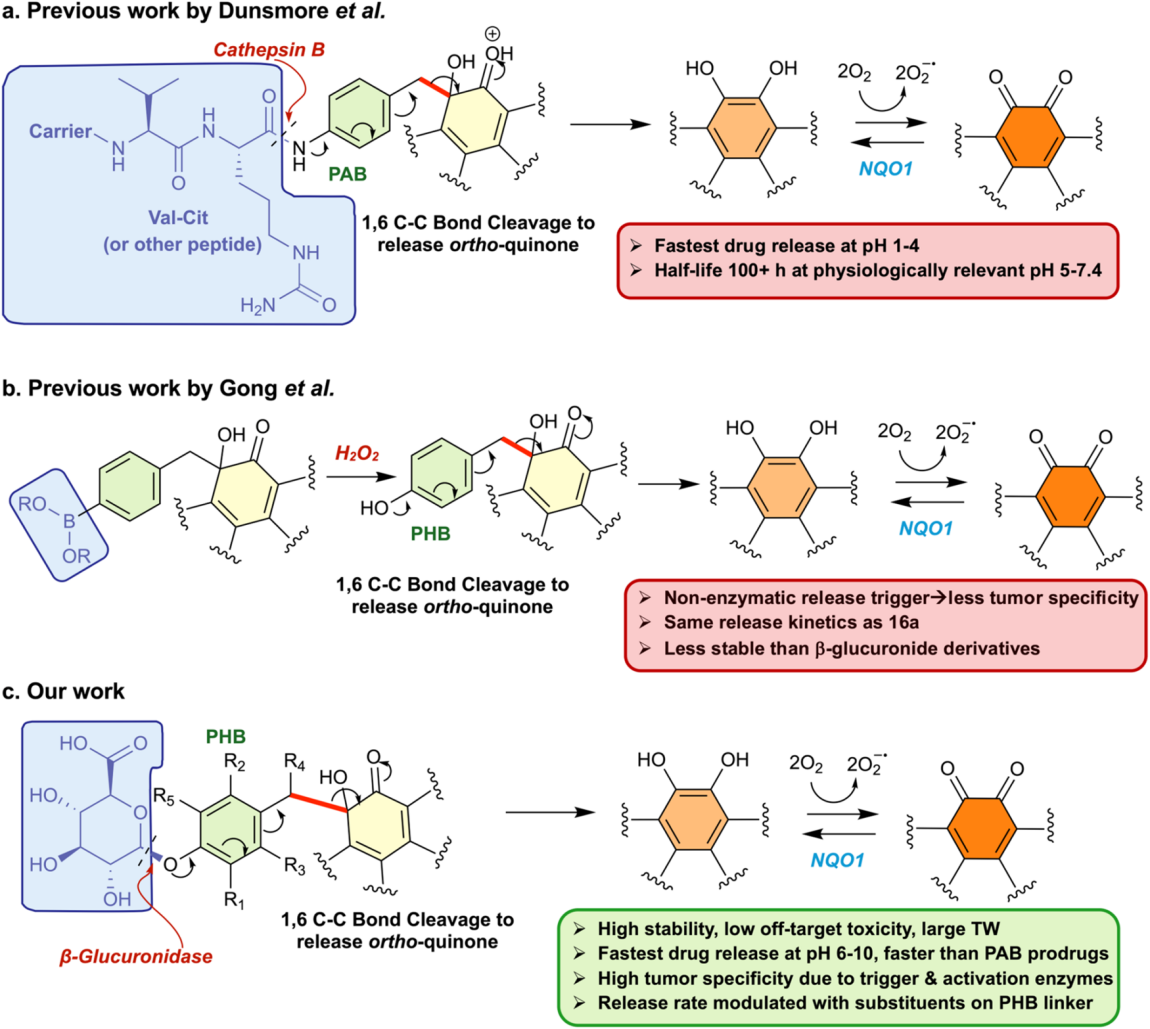
Previous β-lapachone prodrugs. (a) The first-generation PAB β-lapachone prodrug developed by Dunsmore et al.^59^ and (b) the ROS-triggered prodrug developed by Gong et al.^60^ are shown. (c) The β-lapachone prodrug platform developed in this work involves a β-glucuronidase-triggered release that is kinetically modulated by the PHB linker substituents. Pro-moiety (blue), linker (green), benzyl ketol core (yellow), and drug release-triggering enzyme/conditions (red) for each prodrug platform are indicated. The key C–C bond (red) connecting the linker to the ketol stops the redox cycling of the quinone and is cleaved during a 1,6-elimination mechanism (see arrows) to release the active drug.

The β-glucuronidase/β-glucuronide enzyme/prodrug pair is thought to be optimal for tumor-specific enzyme prodrug therapy (EPT) due to the highly advantageous features imparted by both the glucuronide pro-moiety and the triggering enzyme.^61,62^ The glucuronide moiety is both strongly solubilizing^63–67^ and detoxifying^66,67^ by stopping passive cellular uptake. The β-glucuronidase enzyme is widely expressed in endosomes and lysosomes in nearly all tissues, but is specifically found extracellularly in the necrotic areas of the TME where it is excreted by monocytes, granulocytes, and necrotic/apoptotic cells.^68,69^ Pancreatic cancer exhibits significant β-glucuronidase overexpression in tumor and stromal tumor bed cells, and in the necrotic tumor extracellular space.^70^

A β-lapachone β-glucuronide prodrug would take advantage of not only the target specificity imparted by NQO1 and 5-LO overexpression in PDAC, but also the tumor-specific activation given by the expression of β-glucuronidase. In addition, the β-glucuronide moiety used in conjunction with a redox-masking C-alkylated PHB SIL should drastically improve the off-target toxicity and solubility of the β-lapachone prodrugs, widening their therapeutic window (TW). By changing the substituents attached to the PHB linker, the release rate of the *ortho*-quinone can be optimized for fast release in the TME while maintaining stability in circulation. When compared to both the first-generation C-alkylated prodrug developed by Dunsmore et al.^59^ and the Zhang group’s boronate ester prodrug,^60^ the prodrug derivatives in this study demonstrated vastly improved pharmacological properties, advancing *ortho*-quinone drugs closer to clinical relevance.

## Results and Discussion

### Preliminary Modeling of pH-Dependent Cleavage of PHB vs PAB Linkers

To explore the hypothesis that a PHB β-lapachone prodrug could be faster releasing than a PAB β-lapachone prodrug, quantum mechanical calculations were performed for PHB-β-lapachone (**3a**) in comparison to PAB-β-lapachone (**4**) (Figure 2). Similar to what was found for the PAB linker,^59^ the active releasing species for the PHB linker were the deprotonated PHB phenol (**3**_**PhO‑**_, Δ*G* ^‡^ = 19.1 kcal mol^–1^) and the zwitterion species (**3**_**Zw1**_, Δ*G* ^‡^ = 5.6 kcal mol^–1^) (Figure 2a,b and Supporting Information). In this case, no C–C breaking transition state (TS) was found for the protonated ketol carbonyl species (**3**_**OH+**_) due to the lower nucleophilicity of the neutral phenol group in comparison to the aniline group (Figure 2b). The pH-dependent release rate profile could be calculated (Figure 2c) by using the equations shown in Figure 2d. Due to the fact that the p*K*_a_ of the PHB phenol (∼10) is much lower than that of the PAB aniline (∼25), the release rate of β-lapachone from **3a** is predicted to be ∼50 times faster than the release rate of β-lapachone from **4** at pH 6. Most importantly, the release rate for**3a** shows the opposite pH dependence than the release rate for **4** (Figure 2c). In the case of **4**, release is slowest at pH 7, increasing slightly as the pH decreases due to the larger concentration of the protonated ketol carbonyl species (**4**_**OH+**_) in solution. In the case of **3a**, in contrast, the release rate is predicted to increase rapidly as the pH (and therefore the concentration of the deprotonated phenol species **3**_**PhO‑**_) increases, exceeding that of **4** at pH 5.3 and above (Figure 2c).

**Figure 2:**
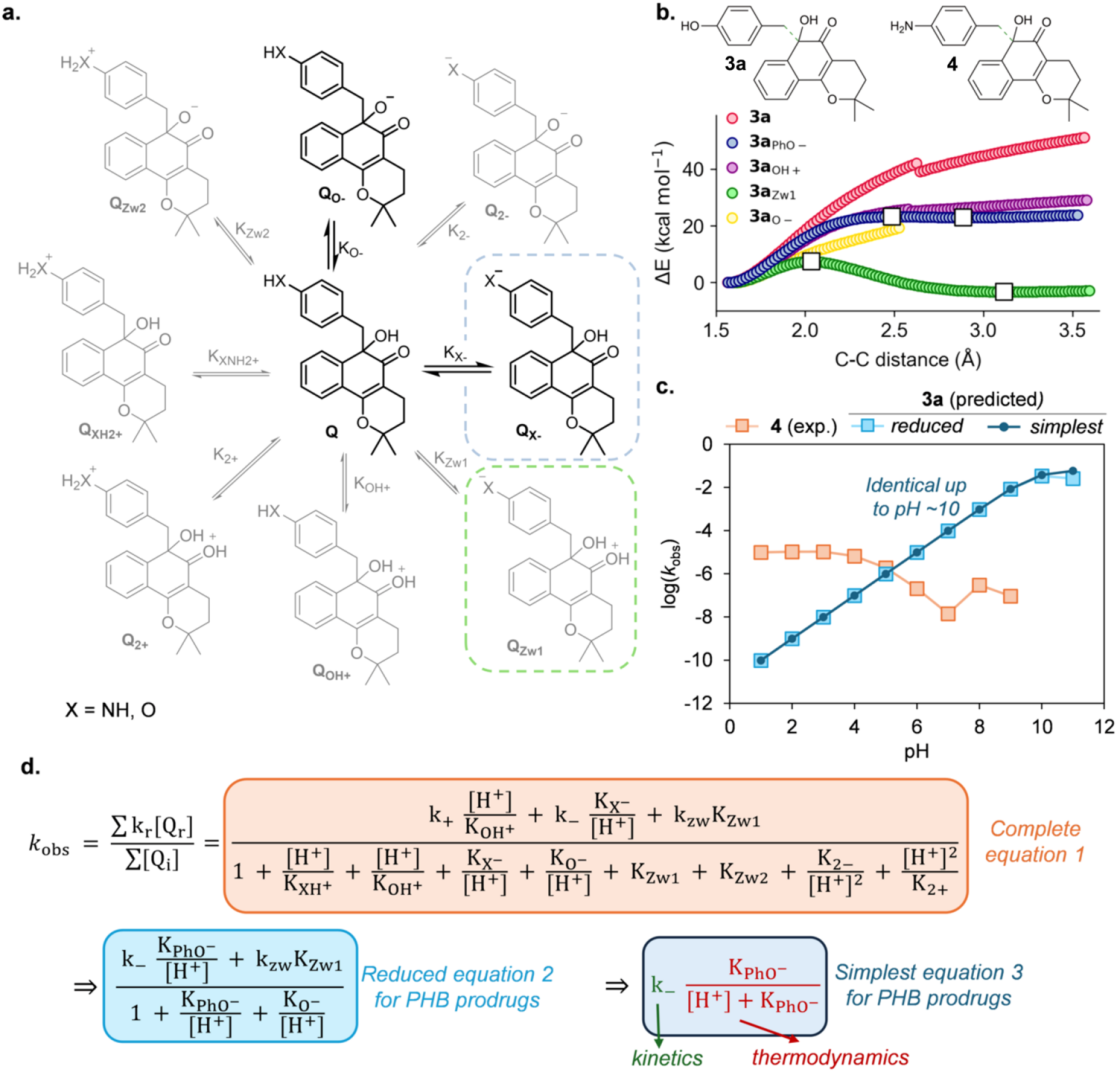
Prediction of the reactivity of PHB vs PAB β-lapachone prodrugs. (a) General chemical scheme showing nine different species denoted as Q (Q = **3a** or **4**, where subscript indicates neutral, cationic, anionic, and zwitterionic) that are in equilibrium for the PHB (X = O) and PAB (X = NH) ketol derivatives of β-lapachone in an aqueous solution. Structures in black denote the major species in solution at pH 1–14 (i.e., they have a p*K*_a_ in that range). Structures in gray denote the minor (negligible) species in solution at pH 1–14 (i.e., they have a p*K*_a_ outside that range). Species inside a dashed box (Q_X–_ and Q_Zw1_) are those for which a 1,6-elimination (i.e., C–C breaking) TS structure was found. (b) Theoretical potential energy surface (PES) calculated with PCM­(H_2_O)/M06-2X/6-31+G­(d,p) for the elimination of species **3**, **3**_**PhO‑**_, **3**_**OH+**_, **3**_**Zw1**_, and **3**_**O‑**_ along the breaking C–C bond (marked in dotted green in the chemical structure). White squares denote stationary points (i.e., TS or local minima). (c) Comparison of the experimental elimination rate constants (*k*_obs_) in logarithmic form previously reported for compound **4** (orange) vs those theoretically predicted for compound **3a** at different pHs using the reduced (light blue) or simplest equation (dark blue) in panel d. Theoretical intrinsic reaction rate constants for the phenolate (*k*_–_ = 6.2·10^–2^ s^–1^) and zwitterionic (*k*_zw_ = 4.9·10^8^ s^–1^) species were calculated quantum mechanically. Theoretical p*K*_a_ values for the phenol → phenolate (**3**_**PhO‑**_, 9.6) and ketol → ketolate (**3**_**O‑**_, 10.9) species were predicted using MolGpKa.^71^ (d) The complete eq 1 (orange) describes the effect of pH on the observed kinetic rate (*k*_obs_) considering all species represented in panel a. This equation can be approximated (eq 2, light blue) by considering only those species that are predominant at pH 1–14 and/or enable elimination (i.e., the zwitterion **3**_**Zw1**_), and even further simplified (eq 3, dark blue) by considering only the dominant phenol/phenolate equilibrium, as the contribution of zwitterion **3**_**Zw1**_ to *k*_obs_ proved to be negligible due to its very low abundance despite being highly reactive (light vs dark blue plots in panel c).

### Design and Synthesis of Prodrug Derivatives

Based on these predictions, a library of PHB prodrug derivatives was designed. Two opposing strategies were explored to modulate the release rate of β-lapachone from the PHB SIL. The first approach aimed at lowering the p*K*_a_ of the PHB phenol by attaching electron-withdrawing groups (EWGs). This would increase the concentration of the actively releasing negatively charged phenolate species in solution, at the expense, however, of deactivating the aromatic ring toward 1,6-elimination. According to the simplified equation shown in Figure 2d, lowering the p*K*_a_ of the phenol (predicted using MolGpKa^71^) from 9.5 to 6.5 while maintaining the intrinsic activation barrier should accelerate the reaction ∼1000 times at pH 5, but only 32-fold at pH 8. Of note, this effect would quickly vanish at sufficiently basic pH values around 10 (Figure S2). In principle, such pH-controlled acceleration would be ideal to quickly release the payload only when reaching the acidic TME, potentially allowing for tumor-selective chemotherapy delivery depending on the circulating dose of the prodrug. Four derivatives were designed toward this goal and compared to the unsubstituted PHB linker derivative (**16a**; predicted p*K*_a_ 9.8): one with two electron-withdrawing fluorine atoms attached *ortho* to the phenol (**16b**; predicted p*K*_a_ 7.8), one with four fluorine atoms at the *ortho* and *meta* positions (**16c**; predicted p*K*_a_ 6.3), one with two fluorine atoms *meta* to the phenol (**16d**; predicted p*K*_a_ 8.3), and one with a sulfonamide *ortho* to the phenol to act as a potential carrier attachment point (**16e**; predicted p*K*_a_ 7.6).

The second strategy explored reducing the intrinsic activation barrier by stabilizing the 1,6-elimination TS. A transient positive charge is hypothesized to form mainly at the benzylic position and *meta* to the phenol in the PHB linker during the elimination reaction. Adding electron-donating groups (EDGs) at these positions would stabilize the transient positive charge, thereby reducing the intrinsic activation barrier of the rate-limiting step, while only slightly altering the phenol’s p*K*_a_.^72,73^ As a drawback, no pH-controlled acceleration can be achieved through this approach (Figure S2). Five additional derivatives were designed to test this hypothesis: one with a methoxy group *meta* to the phenol (**16f**; predicted p*K*_a_ 9.5), one with two methoxy groups *meta* to the phenol (**16g**; predicted p*K*_a_ 9.3), one with two methoxy groups *ortho* to the phenol (**16h**; predicted p*K*_a_ 9.8), one with a methyl group at the benzylic position (**16i**; predicted p*K*_a_ 9.8), and one with a 2-methoxyethoxy group attached *meta* to the phenol (**16j**; predicted p*K*_a_ 9.5). Derivative **16j** also enables the evaluation of a PEG spacer attached to the *meta* oxygen atom. Based on the findings by Rose et al. with self-immolative hydroxybenzyl linkers releasing amine payloads via fast C–N bond breaking,^74^ the distal alkoxy group oxygen could promote drug release by forming a transient seven-membered ring with the benzylic position of the quinone methide formed after 1,6-elimination, in the unlikely case that the C–C bond breaking step is not rate-limiting (Figure S3).

The synthesis of these prodrug derivatives started with a commercially available *para*-hydroxybenzaldehyde or *para*-hydroxybenzyl ketone (**11**). For derivatives **c** and **e**, the corresponding PHB alcohols (**6** and **10**, respectively) had to be synthesized first, as they were not commercially available. **6** was synthesized by reducing **5** with borane (Scheme 1a). **10** was synthesized in 27% yield over 5 steps. First, **7** was chlorosulfonated and coupled with *N*-methylpropylamine to give **8**, which was then deprotected (BBr_3_) with a basic workup giving **9** and reduced (borane) to give **10** (Scheme 1b). Prodrug synthesis then followed six general steps (Scheme 1c). First **11**, **6**, or **10** were glycosylated with the acetyl-protected glucuronide moiety using modified Koenigs–Knorr conditions to give **12** for derivatives **a**, **b**, **d**, **f**, **g**, **h**, and **i**, or **13** for derivatives **c** and **e**. Glycosylation of **6** and **10** exhibited complete regioselectivity at the phenol oxygen rather than the benzylic alcohol (confirmed by cross peaks in the HMBC spectra between the anomeric proton (H1′) and the phenol carbon (C1) (Figures S4, S5)). For derivative **j**, **11j** was first glycosylated to give **12j** with good regioselectivity. The one-unit PEG tether was then attached via a strictly anhydrous Ag_2_O-activated S_N_2 reaction to give compound **17**. For derivatives **a**, **b**, **d**, **f**, **g**, **h**, **i**, and **j**, the benzyl aldehyde or ketone **12** was then reduced into **13** using sodium borohydride over silica gel at 0 °C to preserve the glucuronide acetyl protecting groups. Next, the benzyl alcohols (**13**) were activated via chlorination (SOCl_2_) or bromination (PBr_3_) to form the benzyl halide species (**14**).

**Scheme 1:**
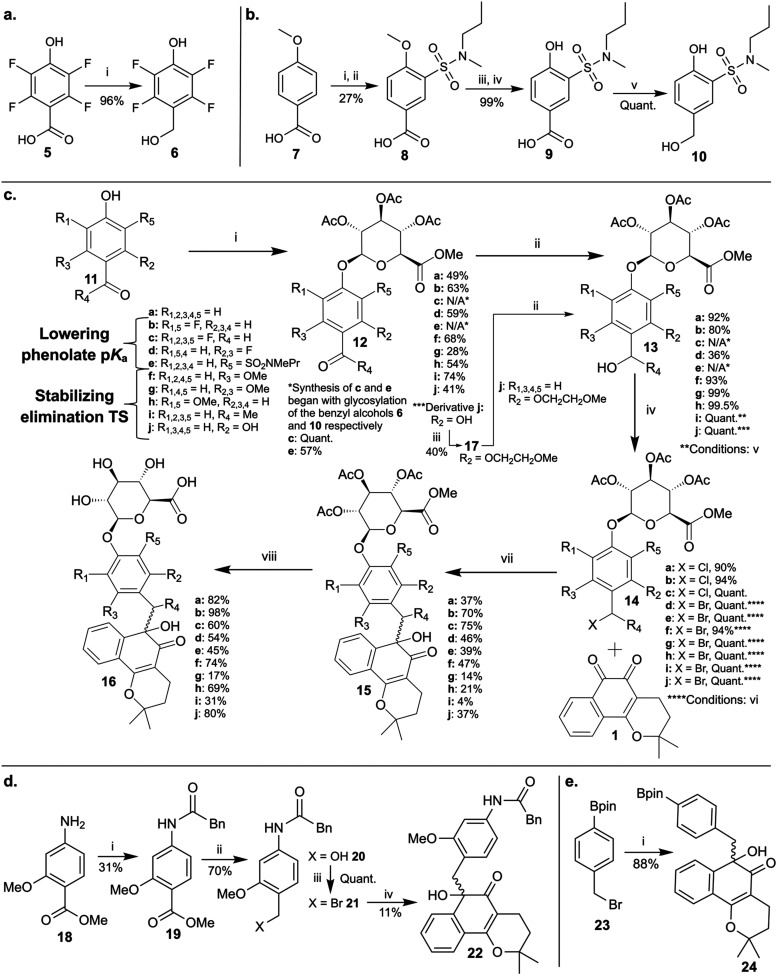
Synthesis of Prodrug Derivatives^a^

a (a) First **6** was synthesized by reducing **5**. i: BH_3_·THF, anhyd. THF. (b) Then **10** was synthesized from **7** over five steps. i: chlorosulfonic acid; ii: *N*-methylpropylamine, TEA, anhyd. DCM; iii: BBr_3_, anhyd. DCM, −78 °C; iv: 10% aq. NaOH; v: BH_3_·THF, anhyd. THF; (c) Finally, **11**, **6**, or **10** were glycosylated, reduced, chlorinated or brominated, and reacted with β-lapachone in an indium-mediated Barbier reaction. Removal of the sugar protecting groups furnished the prodrugs **16** after HPLC purification. i: Acetobromo-α-d-glucuronic acid methyl ester, **11**, Ag_2_O, anhyd. ACN; ii: NaBH_4_, silica, CHCl_3_:IPA 5:1; iii: BrCH_2_CH_2_OMe, Ag_2_O, K_2_CO_3_, DCM/DMF 1:1; iv: SOCl_2_, anhyd. DCM; v: NaBH_4_, MeOH/THF 1:1; vi: PBr_3_, anhyd. DCM or ether; vii: **14**, indium (0) powder, NaI, **1**, anhyd. DMF, sonicated at 40 °C for 12–24 h; viii: LiOH monohydrate, MeOH/THF 1:1, purified by HPLC. (d) Penicillin G amidase-triggered model compound **22** was synthesized from **18** in four steps. i: Phenylacetic acid, EEDQ, anhyd. DCM; ii: LiBH_4_, anhyd. 1:9 MeOH/THF; iii: PBr_3_, anhyd. DCM; iv: **21**, indium (0) powder, NaI, **1**, anhyd. DMF, sonicated at 40 °C for 24 h, purified by HPLC; (e) The prodrug developed by Gong et al., **24**, was synthesized from **23** and **1** using an indium-mediated Barbier reaction. i: **23**, indium (0) powder, NaI, **1**, anhyd. DMF, sonicated for 1 h.

The development of a new *ortho*-quinone alkylation procedure was necessary because the strongly basic aqueous conditions used by Dunsmore et al. (20 equiv KOH),^59^ Gong et al. (8 equiv K_2_CO_3_),^60^ and Kaye et al. (3 equiv NaOH)^75^ to C-alkylate *ortho*-quinones would remove the glucuronide protecting groups. A neutral anhydrous reaction was necessary. Nair et al. reported a facile indium-mediated Barbier alkylation that enabled C-benzylation of 9,10-phenanthrenequinone using benzyl bromide, sodium iodide, and powdered indium (0) metal in anhydrous DMF with ultrasonication.^76^ A modified version of this Barbier reaction using the benzyl halide linkers (**14**) and β-lapachone enabled the synthesis of the C-alkylated β-lapachone prodrugs (**15**) in serviceable yields. The yield was lowest for secondary benzyl bromide linker **14i** (4%), likely due to steric hindrance from the benzylic methyl group.

The C-alkylated β-lapachone prodrugs (**15**) were isolated as a mixture of two diastereomers, with only the two major product isomers isolated for **15i**. Alkylation occurred exclusively at the carbonyl adjacent to the benzene ring in β-lapachone (Figure S6), which is rationalized to be the more electrophilic carbonyl (Figure S7), leading to the calculated thermodynamically more stable regioisomer (Figure S8). Interestingly, this Barbier procedure reverses the polarity of the reaction components in comparison to the previously reported base-activated reactions, making the benzyl halide indium reagent the nucleophile and the *ortho*-quinone the electrophile (Figure S9).

Finally, the glucuronide acetyl protecting groups were removed using LiOH, and the final prodrugs (**16**) were purified using semipreparative HPLC. All prodrugs except **16e** were isolated as a mixture of two diastereomers. The two diastereomers of **16e** had adequate retention time resolution under the semipreparative HPLC conditions to be isolated separately, with the major product isomer being the one that was mainly studied. Derivative **16g** proved to be unstable during purification and isolation, with trace lapachone present after lyophilization. For all prodrug diastereomers, the β-anomer was preserved throughout the entire synthesis based on the *J*_H1″‑H2″_ coupling constant for the anomeric proton being >6 Hz (α: 2–5 Hz, β: 6–10 Hz^77^) (Figure S6, Table S3.1).

An optimized version of Dunsmore’s PAB prodrug was also synthesized for comparison (Scheme 1d). As lowering the p*K*_a_ of the PAB aniline to physiologically relevant values is impractical, only a TS stabilization derivative was explored. The PAB linker was designed with a methoxy group *meta* to the aniline. The aniline in **18** was first protected with a penicillin G amidase (PenG)-labile phenyl acetyl moiety to give **19**, which was reduced (**20**), brominated (**21**), and attached to β-lapachone using the indium-mediated Barbier reaction to furnish **22**. Derivative **22** serves as an enzyme-triggered model compound to measure the release rate of **1** from the modified PAB linker.

For comparison purposes, the pinacol boronate ester prodrug (**24**) developed by Gong et al.^60^ was also synthesized (Scheme 1e). Using the indium Barbier reaction procedure, **24** was synthesized in high yield (88%) from **23** and **1**. These reaction conditions have several advantages over those used by Gong et al. First, no base or water is necessary. Both of those conditions together accelerate the hydrolysis of the boronate ester and benzyl bromide. Second, only a short 1 h sonication was necessary rather than a 6 h heating step. Third, the β-lapachone was completely consumed during the indium-promoted reaction, making purification of **24** by silica gel flash column chromatography facile since **1** and **24** tend to coelute. Finally, the isolated yield of **24** obtained using the indium Barbier reaction (88%) was higher than that reported by Gong et al. (71%).^60^ The indium Barbier reaction is therefore superior to the dithionite/base reaction conditions used by Gong et al. on a practical level to furnish **24**.

### Release Rate Comparison of Self-Immolative Linkers

The mechanism of release of lapachone from the PHB or PAB prodrugs is shown in Figure 3a,b. First, the purified mixture of two prodrug diastereomers (**16a**–**j**) for each PHB derivative was incubated with β-glucuronidase. This enabled full removal of the glucuronide moiety from both diastereomers of the prodrug within 2–15 min, leaving a mixture of two PHB-β-lapachone enantiomers (**3a**–**j**, Figure 3c). For PAB compound **22**, penicillin G amidase was used in place of β-glucuronidase to give a mixture of two PAB-β-lapachone enantiomers (**25**). The **3** (or **25**) solution was then spiked into citrate-phosphate buffers from pH 3–10, and release of **1** from **3** (or **25**) was measured at various time points by analytical HPLC. A representative example of the HPLC traces from the release experiment of **3g** is shown in Figure 3d. A single peak for the two enantiomers of **3** was observed after glucuronide removal, and thus the measured *k*_obs_ was for both enantiomers in the mixture.

**Figure 3:**
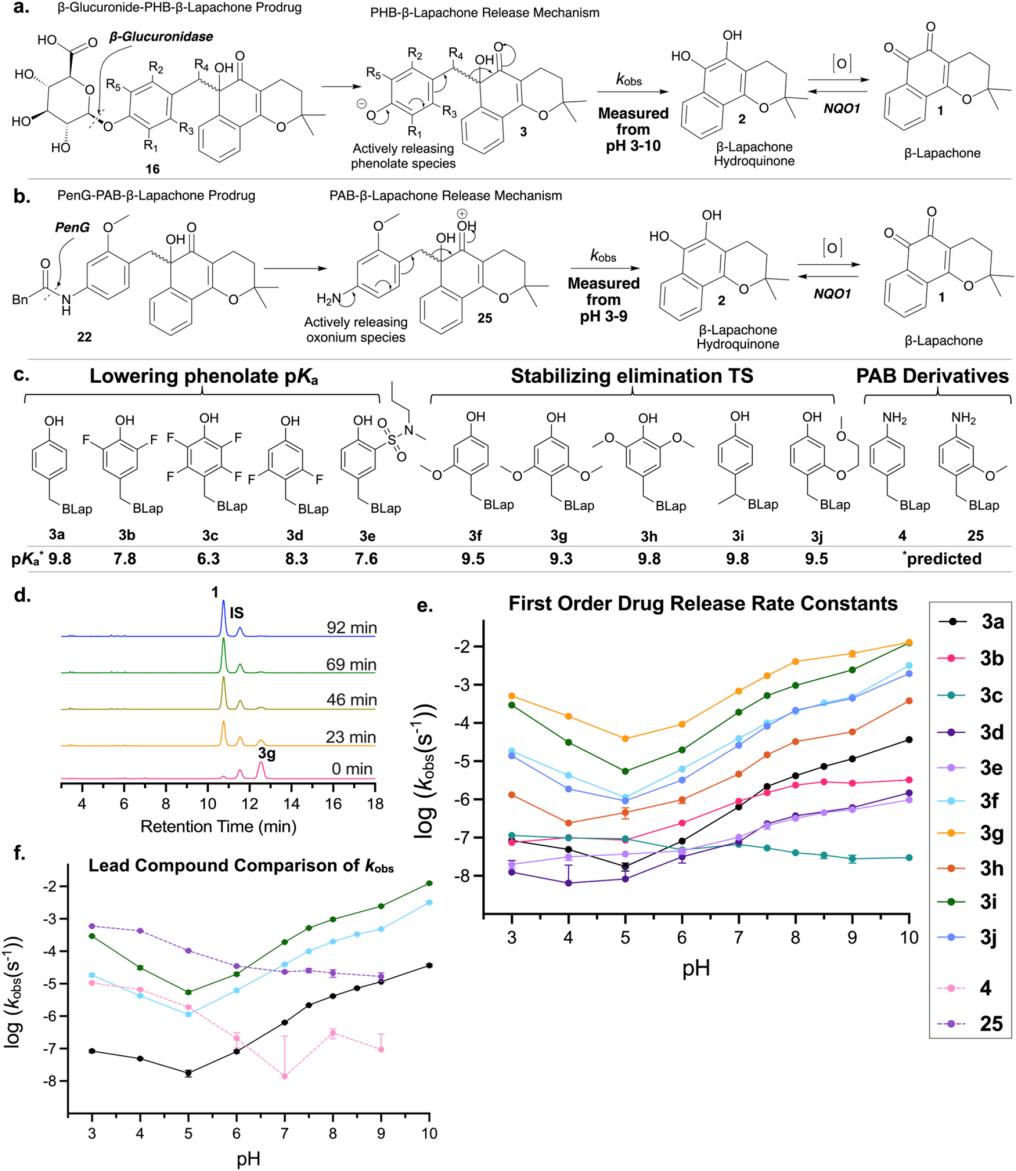
Release rate comparison for PHB and PAB prodrugs. The mechanisms of release for the β-glucuronide–PHB prodrug derivatives (a) and the PAB derivative (b) are shown. β-glucuronidase or penicillin G amidase deprotects the PHB or PAB linkers, yielding **3** or **25**, respectively. A 1,6-elimination then occurs, releasing the hydroquinone form of β-lapachone, which then oxidizes into the *ortho*-quinone. (c) The substituents on the PHB/PAB linker for each derivative are shown and classified into which strategy they employed, lowering phenolate p*K*_a_ or stabilizing the elimination TS. BLap = β-lapachone in its α-hydroxy ketol form (refer to panel a). The predicted p*K*_a_ values are also shown, calculated using MolGpKa.^71^ (d) The representative HPLC traces of a release experiment for 3**g** at pH 7 are shown (RT: retention time) normalized to the warfarin (WF) internal standard (IS, RT = 11.6 min). The peak for 3**g** (RT = 12.5 min) decreases while the peak for **1** (RT = 10.8 min) increases over time. The traces for all other derivatives at each pH are included in Figures S10–S20, S34–S45. (e) The calculated *k*_obs_ values from these release kinetic experiments for each PHB derivative are plotted versus pH on a logarithmic scale. Derivatives **3a**–**e** were designed to lower the PHB phenol p*K*_a_, while derivatives **3f**–**j** were designed to stabilize the 1,6-elimination TS. (f) The first-order reaction rate constants of the PAB derivatives **4** (pink) and **25** (purple) are plotted together with **3a** (black) and the best-performing PHB derivatives **3f** (cyan) and **3i** (dark green) for direct comparison. The derivations of the plotted first-order *k*_obs_ values from linearized initial velocities measured in triplicate are shown in Figures S23–S33. Error bars represent ±95% profile likelihood CI in panels d and e (asymmetric appearance around the mean is increased by the logarithmic *y*-axis, and some downward error bars are missing from extremely slow releasing reactions due to the lower CI boundary being clipped to 0 during fitting, i.e. *k*_obs_ is not tightly constrained by the data due to extremely slow release; such zero lower bounds cannot be presented on a logarithmic axis). See Tables S4.1–S4.14.

Over time, the intensity of the **3/25** peak decreased, while the intensity of the **1** peak increased (Figure 3d). A standard curve for β-lapachone (Figure S22) was prepared, enabling quantification of the released β-lapachone. Release of **1** from the PHB linker was significantly slower than the initial removal of the glucuronide moiety by the β-glucuronidase enzyme and oxidation of the released hydroquinone, so the rate-limiting step of drug release for the prodrugs is the 1,6-elimination of PHB/PAB from **3/25** via the mechanisms shown in Figure 3a,b.

As expected, a clear pH dependence for the release rate was observed (Figures 3e,f, S46), and thus, the computationally predicted opposite pH dependence behavior of PHB linkers in comparison to PAB linkers proved to be experimentally supported (Figure 3f). PHB linkers generally release the slowest at pH 4–5, with a marginal increase in release rate as pH is decreased further. As pH increases above 5, release rate drastically speeds up (Figure 3e). These trends confirm the prediction that the main species contributing to the observed release rate for PHB linkers is the deprotonated phenolate (Figure 2c). This trend held true for all PHB derivatives except tetrafluorinated **3c**, whose acidic phenol group is largely deprotonated near neutral pH, rapidly reaching its upper rate limit set by the intrinsic activation barrier. In contrast, both PAB linkers examined (**4** and **25**) showed the opposite pH dependence trends. Release was slowest at pH 7 and above, increasing rapidly as the pH decreased (Figure 3f).

Despite the observed general beneficial effect of running each individual reaction at higher pH and thus increasing the concentration of the active phenolate species, lowering the p*K*_a_ of the PHB phenol with EWGs was not successful at increasing the observed lapachone release rate from the PHB linker. While **3b** and **3c** showed a moderate release rate increase over **3a** at pH < 6–7, all other derivatives with phenol p*K*_a_ *’*s lower than that of **3a** released β-lapachone significantly slower than **3a**. This effect was especially pronounced for **3c** and **3d**, which had EWGs *meta* to the phenol. Unfortunately, attaching EWGs to the phenol ring proved to be detrimental to the release reaction, with ring deactivation outweighing the effect of increasing phenolate concentration and slowing drug release drastically.

These results highlight the importance of accounting for multiple potentially counteracting parameters when optimizing a chemical reaction. Conversely, as anticipated, the incorporation of EDG groups into the phenol ring significantly promoted lapachone release; unlike with EWG substituents, this effect was uniform across the full pH range, reflected by parallel upward shifts of the *k*_obs_ vs pH curves (Figure 3e). Hence, the release rate from **3f** was around 2 orders of magnitude faster than that from **3a**. The half-life of drug release was decreased from 87.8 ± 3.8 h for **3a** to 1.9 ± 0.1 h for **3f** at pH 7.5. Di-*meta*-substituted **3g** was even faster releasing than **3f**, achieving ∼2200 and ∼800-fold acceleration at pH 5 and 7.5 respectively, while di-*ortho*-substituted **3h** showed the weakest acceleration of the TS stabilizing derivatives.

The addition of an intramolecular “quenching tether” (**3j**, Figure S3) demonstrated none of the release rate increase in comparison to its nontethered analogue **3f** that was observed by Rose et al.^74^ for an analogous but mechanistically distinct reaction. The kinetics of **3j** instead demonstrated that the attachment of a PEG spacer to the *meta* oxygen atom has very little effect on quinone release rate, further proving that in our case, the reaction is kinetically controlled and that 1,6-elimination is the rate-limiting step rather than quinone methide quenching.

To examine whether the structure–activity relationship (SAR) findings made with the PHB linker could translate to PAB-β-lapachone prodrugs as well, the PAB *meta* methoxy derivative **25** was compared to the first-generation plain PAB prodrug (**4**) developed by Dunsmore.^59^

Following the same trends observed with the PHB linker, **25** released β-lapachone around 2 orders of magnitude faster than **4** (Figure 3f). It also maintained the same pH dependence as **4**, with the release rate increasing as pH decreased. The half-life of quinone release was decreased from 101 ± 7 h for **4** to 1.86 ± 0.06 h for **25** at pH 5. As the TME is known to be acidic,^78–80^ **25** possesses an optimal release profile, with faster release at acidic tumor pH 5.5–6.5 and slower release at circulation pH 7.4. However, it lacks the solubility and therapeutic window widening benefits of β-glucuronide masking.

Besides the phenol ring, the other position that proved to have a largely beneficial effect on the observed release rate was the benzylic position. Similarly to what Hay et al. found with PAB linkers,^73^ the attachment of a methyl group to the benzylic position of the PHB linker dramatically increased the release rate of **3i** in comparison to **3a**. A benzylic methyl group had a larger accelerating effect on the release rate than a single *meta* methoxy substituent (**3f**), but a smaller effect than two *meta* methoxy groups (**3g**). The half-life of drug release at tumor relevant pH 6 was decreased from 2371 ± 502 h for **3a** to 30.7 ± 8.4 and 9.8 ± 1.5 h for **3f** and **3i**, respectively (Table 1).

**Table 1:** Drug Release Half-Life from Masked β-Lapachone Prodrugs

derivative	substituents	*t*_1/2_ at pH 6 (h)	95% CI^a^	*t*_1/2_ at pH 7.5 (h)	95% CI^a^
**3a**	R_1,2,3,4,5_ = H	2371	2173–2608	87.9	86.2–89.6
**3b**	R_1,5_ = F, R_2,3,4_ = H	801	764–841	129	126–132
**3c**	R_1,2,3,5_ = F, R_4_ = H	3999	3640–4436	3606	3295–3981
**3d**	R_1,5,4_ = H, R_2,3_ = F	6138	4682–8910	835	756–932
**3e major product**	R_1,2,3,4_ = H, R_5_ = SO_2_NMePr	4284	3627–5231	930	774–1166
**3f**	R_1,2,4,5_ = H, R_3_ = OMe	30.7	27.5–34.7	1.93	1.85–2.02
**3g**	R_1,4,5_ = H, R_2,3_ = OMe	2.08	2.16–2.00	0.11	0.12–0.10
**3h**	R_2,3,4_ = H, R_1,5_ = OMe	196	163–246	13.1	12.7–13.6
**3i**	R_1,2,3,5_ = H, R_4_ = Me	9.8	9.0–10.8	0.37	0.36–0.37
**3j**	R_1,3,4,5_ = H, R_2_ = OCH_2_CH_2_OMe	59.8	52.7–69.2	2.34	2.27–2.42
**4**	None	931	624–1830	–	–
**25**	OMe	5.51	5.30–5.74	7.52	6.54–8.83

a 95% profile likelihood confidence interval (CI) for each half-life value. See Tables S4.1–S4.14 for all kinetic values for the studied derivatives.

### Mechanistic Discussion and Quantum Mechanical Calculations

The rate of chemical reactions triggered by (de)­protonation can be precisely fine-tuned by rationally designing the electronics of the substrate and/or modulating environmental effects (i.e., temperature, pH). The simplified eq 3 shown in Figure 2d quantifies the multiplicative effect on the observed reaction rate constant (*k*_obs_) of the substrate’s intrinsic reactivity in the rate-limiting chemical step (*k*_–_), its acidity (*K*_a_, or *K*_PhO–_ in our case) and the solution pH ([H^+^]), when only one deprotonated species (i.e., a phenolate) dominates conversion from reactant to product.

Of course, this is a simplified scenario; very often, multiple species contribute to the global reaction rate to different extents, depending on their relative abundance in the reaction medium and their intrinsic reactivities, as described by eq 1 in Figure 2d.^59^ Ideally, the 1,6-elimination reaction rates of PHB self-immolative linkers at specific pH values could be exquisitely controlled by rational substrate engineering *if* their intrinsic reactivity and acidity were completely decoupled. As shown in Figure S2, changes in activation energies (Δ*G* ^‡^, therefore in *k*_–_ at a given temperature) would simply shift upward or downward the pH-dependent *k*_obs_ values; intrinsically more reactive substrates would *always* react faster, at any pH. On the other hand, lowering the p*K*_a_ of the substrate would allow faster reactions (i.e., closer to the upper limit determined by *k*_–_) *selectively* at more acidic pH values.

In practice, however, both contributing factors to the reactivity are tightly coupled and influence each other in opposing ways. This is particularly true when engineering the properties of reactive aromatic compounds; it is nearly impossible to significantly increase the acidity of a phenol by adding EWG to the aromatic ring without deactivating it by inductive or resonance effects. According to the equation Δ*G* = 2.303·*RT*·Δp*K*_a_, lowering the p*K*_a_ of a phenol by 3 units (e.g., from 9.5 to 6.5) stabilizes the corresponding phenolate by approximately 4 kcal mol^–1^, corresponding to a ∼1000-fold increase in the concentration of the phenolate at pH 5. However, this stabilization and potential advantage is completely canceled if the activation energy of the reaction (Δ*G* ^‡^) increases by an equivalent of 4 kcal mol^–1^ (Figure S47). This interplay between deprotonation thermodynamics and elimination kinetics in PHB derivatives is unsurprising; the more thermodynamically stable the phenolate becomes, the less kinetically reactive it is.

Quantum mechanical calculations performed on phenolates **3a**, **3b**, **3c**, **3d**, **3f**, **3g**, **3h**, and **3i** (Figure 4 and Supporting Information) further supported this notion. In good agreement with the experimental trend, the free energy barriers calculated for 1,6-elimination leading to β-lapachone release increased from the most activated di-*meta*-OMe-substituted derivative **3g** (Δ*G* ^‡^ = 15.5 kcal mol^–1^; *k*_–_ = 25.37 s^–1^ at 25 °C) to *meta*-OMe-substituted **3f** (Δ*G* ^‡^ = 16.9 kcal mol^–1^; *k*_–_ = 2.54 s^–1^ at 25 °C) to di-*ortho*-OMe-substituted **3h** (Δ*G* ^‡^ = 17.4 kcal mol^–1^; *k*_–_ = 1.02 s^–1^ at 25 °C) and the unsubstituted parent compound **3a** (Δ*G* ^‡^ = 19.1 kcal mol^–1^; *k*_–_ = 0.07 s^–1^ at 25 °C) (Figure 4a).

**Figure 4:**
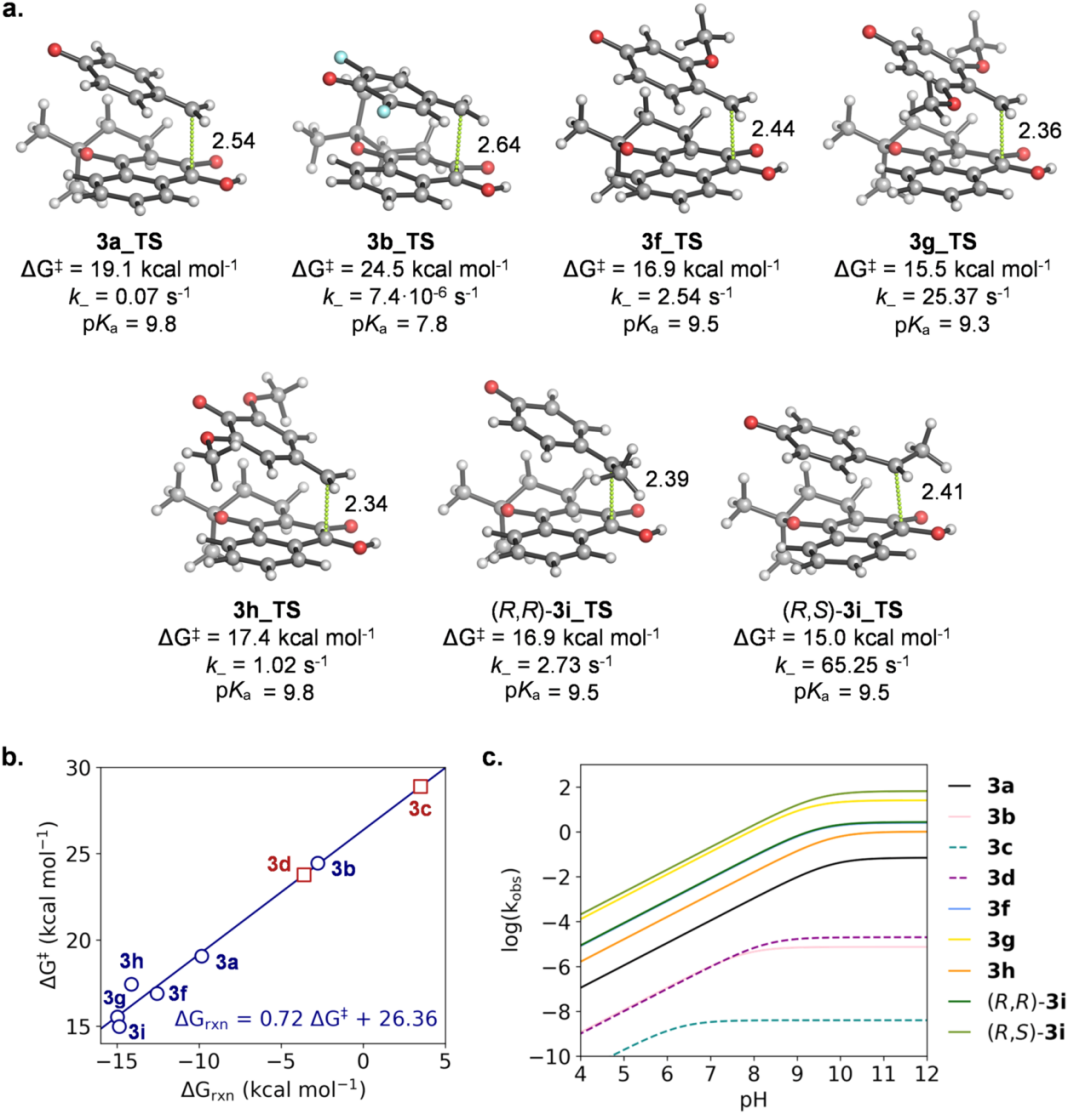
(a) TS structures for the 1,6-elimination reactions of phenolates **3a**, **3b**, **3f**, **3g**, and **3h** (arbitrarily drawn in (*R*) configurations) and diastereomers (*R,R*)- and (*R,S*)-**3i** calculated at the PCM­(H_2_O)/M06-2X/6-31+G­(d,p) level. Also see Figure S48 and Table S5.1. Green dashed lines show interatomic distances (in angstroms) for the breaking C–C bonds. Intrinsic first-order kinetic constants (*k*_–_) were calculated from the theoretical activation energies (Δ*G* ^‡^ in kcal mol^–1^) at 25 °C by using the Eyring equation. p*K*_a_ values were predicted using MolGpKa.^71^ (b) Bell–Evans–Polanyi (BEP) plot of the 1,6-elimination reactions calculated at the PCM­(H_2_O)/M06-2X/6-31+G­(d,p) level. Blue circles denote data points used for the linear fitting (i.e., where TS could be calculated), while red squares represent predicted activation energy estimates from the interpolation of the linear model for **3c** and **3d**, for which exact TS structures and energies could not be calculated. *R* ^2^ = 0.96. (c) Predicted values for *k*_obs_ in logarithmic form as a function of pH using the simplest eq 3 shown in Figure 2d for the elimination of phenolates **3a**, **3b**, **3c**, **3d**, **3f**, **3g**, and **3h** and diastereomers (*R,R*)- and (*R,S*)-**3i**. Curves calculated using Δ*G* ^‡^ from the optimized TS are shown as solid lines, while dashed lines represent curves calculated using the interpolated ΔG^‡^ from the BEP plot. The curves for **3f** and (*R*,*R*)-**3i** overlap as both compounds feature very similar *k*_–_ and p*K*_a_ calculated values.

Of note, the high reactivity exhibited by derivative **3i**, substituted with a methyl group at the benzylic position, was also reproduced: Δ*G* ^‡^ = 16.9 kcal mol^–1^; *k*_–_ = 2.73 s^–1^ at 25 °C for (*R*,*R*)- diastereomer and Δ*G* ^‡^ = 15.0 kcal mol^–1^; *k*_–_ = 65.25 s^–1^ at 25 °C for (*R*,*S*)-diastereomer. On the contrary, the poor reactivity of deactivated fluorine-substituted compounds, linked to the very low stability of the forming *para*-quinone methide intermediate (*vide infra*), precluded locating a stationary point (i.e., a TS) in the PES of derivatives **3c** and **3d**, and a high-energy TS could be optimized only for di-*ortho*-F-substituted **3b** (Δ*G* ^‡^ = 24.5 kcal mol^–1^; *k*_–_ = 7.4·10^–6^ s^–1^ at 25 °C). Notably, all of the calculated structures showed a conserved folded geometry, in which the phenolate group is placed above the lapachone moiety. Particularly in the reagents, the phenolate group is positioned toward the axial methyl groups of the lapachone due to a CH-π interaction (Figure S48). On the other hand, in the TS this interaction is gradually lost as the phenolate leaves, so that the methyl groups tend to reverse their arrangement (i.e., axial-to-equatorial and *vice versa*) through a conformational change in the β-lapachone ring (Figure 4a).

Conceptually related to the entangled phenol deprotonation thermodynamics and 1,6-elimination kinetics described above, the thermodynamic stability of the *para*-quinone methide byproducts was found to be an excellent predictor of β-lapachone release rate, unlike HOMO–LUMO energy gaps^72^ in the reacting phenolates (Figure S49). Therefore, a reasonably good linear correlation between activation (Δ*G* ^‡^) and reaction (Δ*G*_rxn_) energies with a slope of ∼0.7 was found for the calculated 1,6-elimination reactions (Bell–Evans–Polanyi plot, Figure 4b); this is in agreement with the Hammond–Leffler postulate^81^ and Marcus theory,^82^ which state that for similar reactions ΔΔ*G* ^‡^ ≈ 1/2ΔΔ*G*_rxn_.^83^ In line with the long distances calculated for the breaking C–C bonds (2.3–2.6 Å, Figure 4), slopes >0.5 indicate late, product-like transition states.^84^ In fact, the *more exergonic* reactions (after β-lapachone protonation, which is a common subsequent step involving an equal change in energy for all derivatives; 1,6-elimination is intrinsically endergonic despite being an entropically favored fragmentation reaction) corresponded to the *lower energy TS’s*. In turn, these exhibited an earlier character described by smaller elongations of the breaking C–C bonds with respect to the substrate (from 0.77 Å in the most reactive **3g_TS** to 1.07 Å in the least reactive calculable **3b_TS**). Interpolation on the Δ*G* ^‡^ vs Δ*G*_rxn_ plot (Figure 4b) provided estimated activation barriers for the very slow β-lapachone releasing derivatives lacking an identifiable elimination TS (Δ*G* ^‡^ ≈ 29 kcal mol^–1^; *k*_–_ ≈ 4·10^–9^ s^–1^ at 25 °C for **3c** and Δ*G* ^‡^ ≈ 24 kcal mol^–1^; *k*_–_ ≈ 2·10^–5^ s^–1^ at 25 °C for **3d**).

With the calculated activation barriers and p*K*_a_ values for each compound, and by using the simplest eq 3 shown in Figure 2d, the *k*_obs_ values can be predicted as a function of pH (Figure 4c). As in the experimental profiles, compounds bearing EWGs (**3b**, **3c**, and **3d**) reach their maximum *k*_obs_ at lower pH values; however, these values remain low due to their higher intrinsic activation barriers. In contrast, reagents with EDGs at either the aromatic ring (**3f**, **3g**, and **3h**) or the benzylic position (**3i**) reach their maximum *k*_obs_ at similar, higher pH values but with significantly higher rates due to their lower intrinsic activation barriers. This results in an upward vertical shift of the curves, consistent with the experimental trends. The comparison of release rate profiles between **3g** and **3i** suggests that the experimentally characterized form of **3i** consisted of the (*R*,*R*) and (*S*,*S*) enantiomers, which together derive from the isolated major product diastereomers of precursor **16i**.

All these results corroborate the idea that adding EDG substituents at either the aromatic ring (particularly *meta* to the phenol) or the benzylic position (ultimately yielding a more stable trisubstituted alkene in the *para*-quinone methide intermediate) benefits both the interlinked kinetics and thermodynamics of the self-immolative 1,6-elimination reaction. This strategy is much more beneficial than attempting to increase the acidity of the releasing phenol. Moreover, the remarkable agreement between the calculated and experimental reactivity trends highlights the importance of incorporating environmental effects (i.e., pH vs p*K*_a_) to properly model the behavior of ionizable reagents in aqueous media.

### Stability of β-Lapachone Prodrugs

The diastereomeric mixture of each β-glucuronide–PHB-β-lapachone prodrug (**16a**–**j**) and PAB derivative **22** were then tested for stability in citrate-phosphate buffers across a range of pHs (3, 5, 7, 7.4 (PBS), 9) via an HPLC stability assay. For derivatives **16a**, **16b**, **16d**, **16e**, **16h**, and **16j**, when no β-glucuronidase enzyme was added, both diastereomers of **16** were fully stable at 37 °C for 72 h (Figures 5a,d, S63, S64, S66–68, S71, and S73). For derivatives **16c**, **16f**, **16g**, **16i**, and **22**, trace β-lapachone release was detected (Figures 5b,e, S65, S69–70, S72, and S74). This premature release was greatest at acidic pH 3 for **16g**, with very minimal lapachone release detected at pH 5–9 (Figure 5c). The released lapachone was quantified and compared to the starting concentration of each prodrug to calculate the percent of the prodrug that had been prematurely released (Figures 5f,g, S76). The percentages of premature lapachone release across the pH range for each derivative are shown in Tables S6.1–S6.2. Generally, the prodrug derivatives became less stable as the release rate from **3** after β-glucuronide deprotection increased. The fastest-releasing precursor **16g** also proved to be the least stable prodrug and was impossible to purify cleanly without degradation. Therefore, a trade-off exists between ring activation, improving the release rate from **3** while also decreasing prodrug stability, and it rendered **16g** clinically disadvantageous.

**Figure 5:**
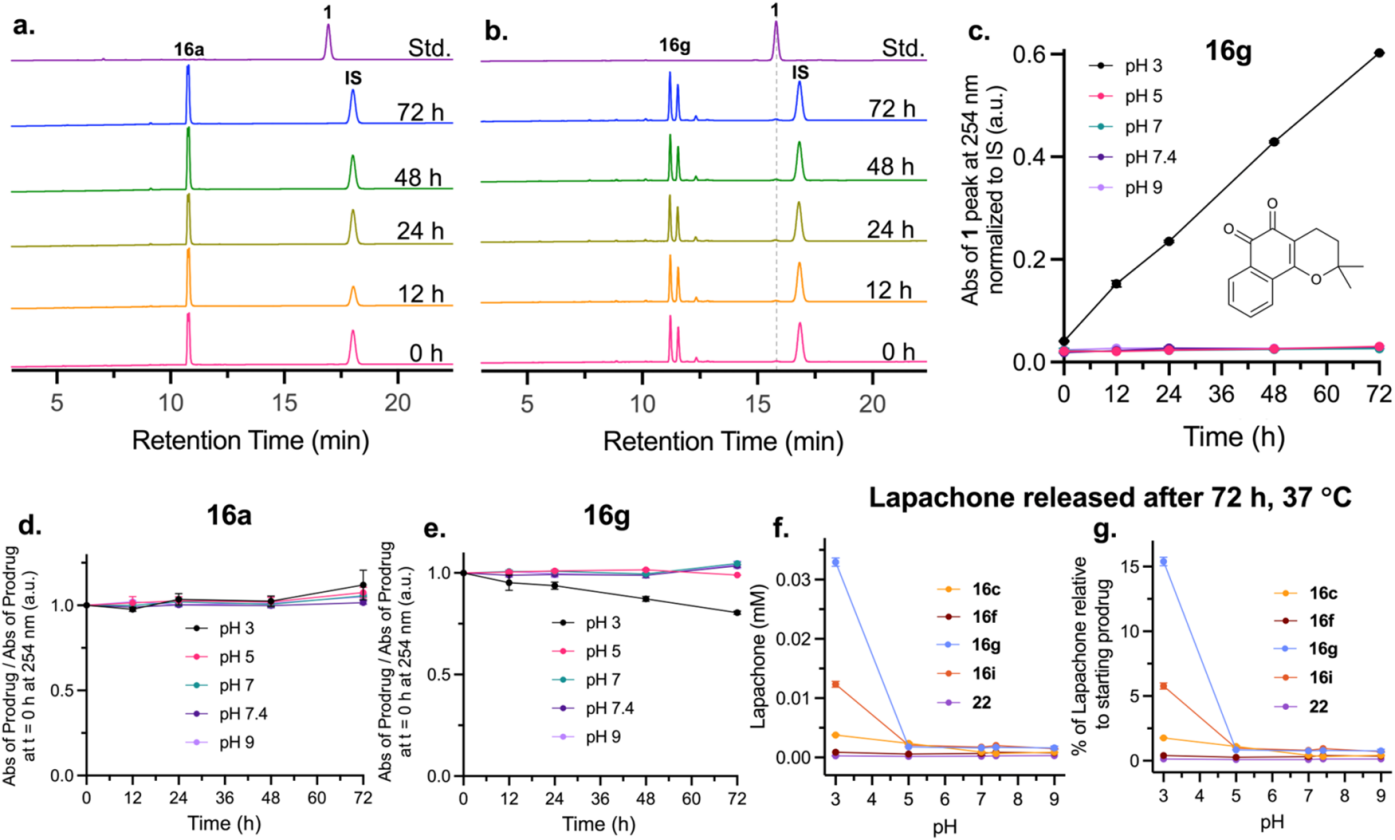
Stability of prodrugs is pH dependent. The representative HPLC traces for (a) a stable prodrug **16a** and (b) an unstable prodrug **16g** are shown after incubation at 37 °C in pH 7.4 PBS buffer. The peak for **16a** (RT = 10.8 min) remained unchanged (d), and no peak for **1** (RT = 16.9 min) was detected. For **16g**, a substantial change in the **16g** peak area (RT = 11.2, 11.5 min) was observed at pH 3 (e), and a growing **1** peak (RT = 15.8 min, trace “Std.”) was detected at all pHs tested. (c) The area of the lapachone peak for **16g** normalized to the IS was plotted, displaying substantial growth over time at pH 3. For those derivatives that exhibited instability, the premature lapachone release was quantified (f) and calculated as a percentage relative to the starting concentration of the prodrug (g). For panels c–g, each value is plotted as the mean ± SD of three replicates. The HPLC traces (Figures S50–S62) and peak area graphs (Figures S63–S75) for all other derivatives can be found in the Supporting Information.

### Comparison to the Boronate Ester Prodrug

To benchmark the β-glucuronide prodrug derivatives, they were directly compared with the stability and release behavior of boron-substituted **24**. Quinone release for **24** via the mechanism shown in Figure 6a was evaluated at pH 5, 7.4, and 9 after 5 equiv of hydrogen peroxide were added at 37 °C (Figures 6b,e, S78), the same ratio of prodrug to H_2_O_2_ used by Gong et al. They reported complete release of **1** from **24** within 2 h at pH 7.4,^60^ which is surprising considering that **16a** and **24** both react through the same phenolate **3a** intermediate during release (Figure 6a), and that we found **3a** (derived from **16a**) to have a half-life of 221.8 ± 23.1 h at pH 7.4.

**Figure 6:**
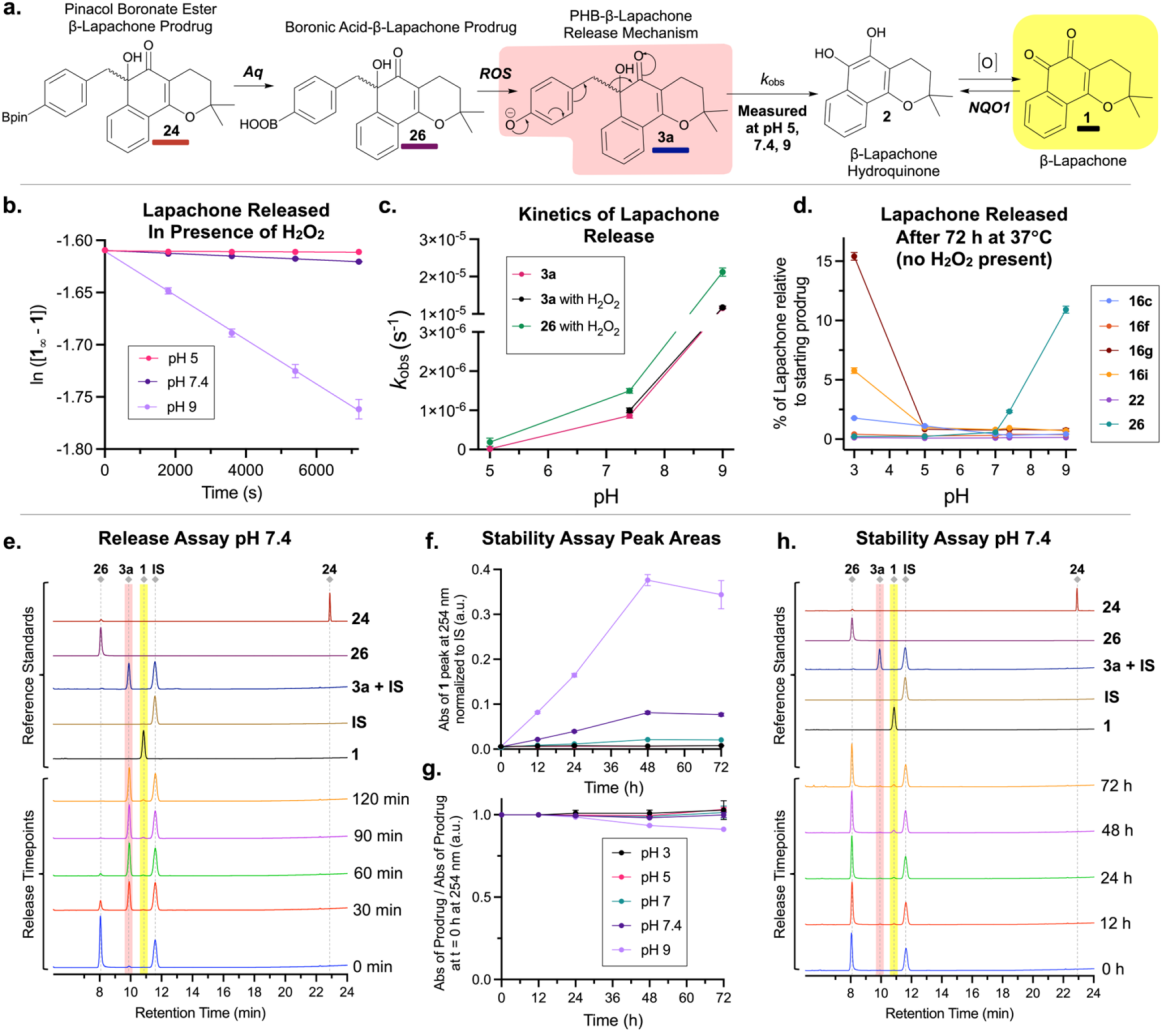
Release rate and stability comparison for glucuronide and boronate prodrugs. (a) The mechanism of release for pinacol boronate ester prodrug **24** is shown. **24** is hydrolyzed rapidly into **26**, which is then oxidized by ROS (H_2_O_2_ 5 equiv) to form **3a** during incubation in buffer at 37 °C. **3a** is the same intermediate that forms when β-glucuronidase deprotects **16a**. A 1,6-elimination then occurs, releasing the hydroquinone form of β-lapachone, which then oxidizes into the *ortho*-quinone. (b) The released lapachone was quantified and fitted linearly to the first-order kinetic equation for the release of **1** from **3a**, which was proved to be the rate-limiting step. The slopes of this graph were used to calculate the *k*_obs_. (Also see Figures S79 and S80) (c) The calculated *k*_obs_ values for the release of **1** from **3a** (derived via enzymatic deprotection of **16a** (maroon or black) or oxidation of **26** (teal)) are plotted as a function of pH. The *y*-axis is shown cut into two parts to allow better data visualization. (d) The premature lapachone release for **24** (**26** after hydrolysis) in the absence of exogenous H_2_O_2_ was quantified and calculated as a percentage relative to the starting concentration of the prodrug. This percentage was plotted as a function of pH in comparison to those PHB and PAB linker derivatives that exhibited instability. (e) Representative HPLC traces of a release experiment for **24** at pH 7.4 are shown, normalized to the IS (RT = 11.7 min), with reference standards shown for peak identification via retention time comparison. Peak identification by MS was also performed (Figure S85). (f, g) The peak areas of β-lapachone (f) and **26** (g) are plotted over the course of a 72 h stability assay performed in citrate-phosphate buffer at 37 °C. (h) The representative HPLC traces of a stability assay for **24** at pH 7.4 (PBS buffer) are shown, normalized to the IS (RT = 11.7 min), with reference standards shown for peak identification via retention time comparison. For panels b, d, f, and g, each value is plotted as the mean ± SD of three replicates. For panel c, each value is plotted as the mean ± 95% CI.

Boron-substituted **24** was found to be quite hydrophobic based on the high retention time (22.9 min) of a standard dissolved in DMSO on the analytical C18 HPLC column.

Within 5 min after dilution into aqueous buffer, **24** was completely hydrolyzed into the boronic acid β-lapachone prodrug (**26**) (RT = 7.9 min, Figures 6e,h, S78), as confirmed by LC-MS (Figure S85) and NMR (SI) analysis. This rapid hydrolysis explains the high solubility and matching retention times observed by Gong et al. for what they reported as the pinacol boronate ester, the boronic acid, and the propane boronate ester.^60^

Compound **26** quickly oxidized into the **3a** intermediate at pH 7.4 (Figure 6e) and pH 9 (Figures S78, S80a, and S81). At pH 5, oxidation was much slower, with the majority of **26** persisting in solution after 2 h (Figures S78, S80a, and S81). A sample of **16a** that was deprotected by β-glucuronidase to form **3a** was compared to a H_2_O_2_-treated **26** sample, confirming that the identity of the peak at 9.9 min that formed from the disappearance of **26** was **3a**. This was also confirmed by LC-MS (Figure S85) and NMR (SI). **3a** then persisted in solution over the 2 h incubation period, with a tiny β-lapachone peak emerging slowly (Figures 6e, S78). The rate of β-lapachone release from **3a** (derived from **24/26)** was then quantified via linear fitting of first-order kinetic graphs with 1,6-elimination from **3a** as the rate-limiting step (Figures 6b,c, S79, S80b, and S81). The presence or absence of peroxide did not affect the release rate of **3a** (derived from **16a**) (Figures 6c, S77, and S82).

Similarly to what was observed for **16a**, for the boronic acid prodrug **26**, β-lapachone release from the PHB linker was pH dependent. Slowest release occurred at pH 5, with the release rate increasing as pH increased. The half-life of β-lapachone release from **26** at 37 °C was found to be 128.6 ± 9.4 h at pH 7.4, contradicting what was reported by Gong et al. When the β-lapachone release rates from **3a** derived from **26** or from **16a** were compared, lapachone was released at approximately the same rate at pH 5, 7.4, and 9 (Figures 6c, S82). The rate-determining step in both cases is the 1,6-elimination of the PHB linker from the common **3a** intermediate (Figure S81). Derivatives **16f**–**j** and **22** had significantly faster lapachone release rates than **16a** and **26** (Figures S83–S84), confirming that the new lead compounds are an improvement over previous prodrug designs.

The stability of **26** was also evaluated from pH 3–9 (Figures 6d,f–h S86–S87). **26** was most stable at pH 3 and 5, with only trace lapachone release observed. At pH 7, 7.4, and 9, however, substantial release of lapachone was observed in the absence of hydrogen peroxide, with instability increasing with an increased pH (Figure 6d,f). This degradation was the largest at pH 9, with a measurable decrease in the prodrug peak area after 72 h (Figure 6g). The lapachone released after 72 h was quantified using a standard curve (Tables S6.1–S6.2). At pH 3 and 5, approximately 0.2% of the total prodrug lapachone had been released. This increased to ∼0.6% at pH 7, ∼2.3% at physiological pH 7.4, and ∼10.9% at pH 9 after 72 h at 37 °C (Figure 6d, Tables S6.1–S6.2).

In comparison, **16a**, containing a structurally analogous linker to **26**, was fully stable from pH 3–9 (Figure 5a,d) with no lapachone release detected in any sample during the 72 h incubation at 37 °C. When **26** was compared to derivatives **16c**, **16f**, **16g**, **16i**, and **22** that demonstrated varying levels of instability, **26** was more unstable at physiological pH than even the least stable glucuronide derivatives **16g** and **16i** (Figure 6d), indicating that a stability benefit is gained from using a glucuronide moiety rather than a boronate ester moiety as the prodrug masking group.

While premature release from **26** is more likely to occur in circulation rather than in the tumor, the glucuronide prodrugs prematurely release lapachone at acidic pHs, so premature release is likely to occur only in the acidic TME, which could pose an additional benefit. Therefore, the β-glucuronide prodrugs demonstrated superior *in vitro* release and stability profiles in comparison to the boronate ester prodrugs developed by Gong et al.

### *In Vitro* PDAC Cellular Efficacy

All *ortho*-quinone prodrug derivatives were tested on two pancreatic cancer cell lines, PANC-1 and AsPC-1, to assess efficacy in cell killing (Figures 7, S88–89, S91, Tables 2, and S8.1). Exogenous β-glucuronidase was added to the cell media to mimic the extracellular enzyme present in the TME. Optimal enzyme concentrations were screened, and it was shown that β-glucuronidase by itself had no effect on cell viability nor did it influence β-lapachone’s ability to kill cells (Figure S90). β-Lapachone (**1**) alone had an IC_50_ of 1.8 μM (Figure 7a) and 4.7 μM (Figure 7b) in PANC-1 and AsPC-1 cells, respectively. Therefore, we expected the derivatives that showed fast release of β-lapachone to have IC_50_s similar to these values. The original PHB derivative, **16a**, had IC_50_ values of 11 μM (PANC-1, Figure 7a) and 16 μM (AsPC-1, Figure 7b) upon treatment with β-glucuronidase, demonstrating poor payload release throughout the long incubation time (72 h). Those derivatives with release rates slower than that of **16a**, like **16e**, had less efficacy in cells comparatively (Table 2).

**Figure 7:**
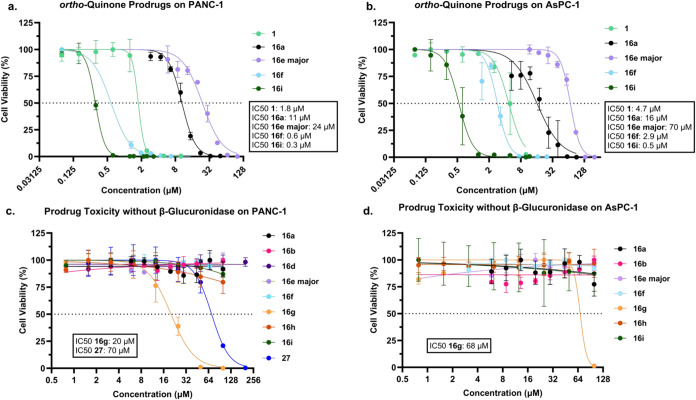
PDAC cell viability with prodrug treatment. Representative PANC-1 (a) and AsPC-1 (b) cell viability data of four β-lapachone prodrug derivatives after 72 h of treatment with exogenous β-glucuronidase added. The IC_50_ values (mean ± SD) of all replicates of each prodrug on the PANC-1 and AsPC-1 cell lines are listed in Table 2. Panels (c, d) show the toxicity of functional prodrugs up to 100 μM in PANC-1 (c) and AsPC-1 (d) cells without exogenous β-glucuronidase enzyme present after 72 h incubation. The unstable **16g** derivative has an IC_50_ of 26 ± 6 and 75 ± 7 μM without the exogenous enzyme trigger present in PANC-1 and AsPC-1, respectively. **27** without a glucuronide moiety had an IC_50_ of 70 μM in PANC-1 (not tested in AsPC-1), whereas the β-glucuronide prodrugs were nontoxic up to 100 μM without exogenous enzyme for both cell lines.

**Table 2:** PDAC Cell Viability with Prodrug Treatment for All Derivatives

Prodrug Derivative	PANC-1 IC_50_ (μM)[Table-fn t2fn1]	AsPC-1 IC_50_ (μM)[Table-fn t2fn1]
**1**	1.8 ± 0.2	5.4 ± 0.7
**27**	70	-
**16c**	Inactive	Inactive
**16e major product**	25 ± 3	59 ± 12
**16d**	21 ± 3	-
**16b**	11 ± 2	24
**16a**	9.6 ± 0.9	19 ± 3
**16h**	1.2 ± 0.4	3.2 ± 1.7
**16j**	1.1 ± 0.4	2.8 ± 1
**16g**	1.2 ± 0.3	1.6 ± 0.6
**16f**	0.7 ± 0.2	2.9 ± 0.06
**16i**	0.8 ± 0.5	0.9 ± 0.5

1 Average IC_50_ values ± standard deviation of at least two biological replicates for each prodrug on the PANC-1 and AsPC-1 cell lines are shown. All derivatives were tested on the PANC-1 cell line, and only those that performed better than **16a** were tested in replicates on both the PANC-1 and AsPC-1 cell lines. Values with no error represent only one biological replicate, and empty values indicate no viability assays were run for that condition.

However, for the best-performing derivatives with the fastest release rates, we observed IC_50_ values equal to or even lower than those of pure β-lapachone, demonstrating complete drug release during the incubation period. Prodrugs **16f** and **16i** showed IC_50_ values of 700 and 800 nM in PANC-1, respectively (Figure 7a), which is two to 3-fold more toxic than β-lapachone in this cell line. We also see that this increased toxicity is more pronounced in the AsPC-1 cell line, reducing the IC_50_ from 5.4 μM of **1** to 900 nM of **16i**. When comparing the measured half-lives of the derivatives to their IC_50_ values in cell studies, we see a strong positive correlation between the two variables, signaling that the β-lapachone release rate is the main predictor in cell killing efficacy (Figure S92).

To assess the TW of the prodrug derivatives, we tested each of them in both the PANC-1 and AsPC-1 cell lines at up to 100 μM without the β-glucuronidase enzyme present. In agreement with the HPLC stability assay data, we saw no toxicity in all stable prodrug derivatives up to this concentration (Figure 7c,d). Compound **16g**, which showed instability in previous assays (Figure 5b,c,e–g), also emulated the same behavior in cell culture, with an IC_50_ of 26 ± 6 μM in PANC-1 and 75 ± 7 μM AsPC-1 when no release-triggering enzyme was present. We also saw in PANC-1 that a nonreleasing benzyl lapachone control compound (**27**), which does not contain a glucuronide moiety, had an IC_50_ of 70 μM (Figure 7c). In comparison, all the stable glucuronide prodrug derivatives retained 100% viability at 100 μM. This confirms that the β-glucuronide sugar employed in our prodrug format increases the TW of the prodrug by preventing passive cellular uptake.^67,85–87^ This data indicates that the best-performing, stable derivatives have a therapeutic index (TI) greater than 110, since we were unable to reach any killing effects up to 100 μM. It is generally considered that a drug has a good therapeutic safety profile if the TI is greater than 10, which our best derivatives far exceed, signaling that our prodrug is safe to deliver systemically in future *in vivo* experiments and should not cause any adverse effects in areas where β-glucuronidase is not overexpressed.^88,89^

### Cellular Mechanism of the Prodrug

β-Lapachone’s established mechanism of action by generating ROS species through the NQO1 enzyme, as well as the additional inhibition of 5-LO shown through our group’s work, drove our interest in determining if the NQO1 and 5-LO pathways are responsible for our prodrugs’ killing effect in PDAC cells. We measured NQO1 and 5-LO protein expression levels via Western blot analysis in both the PANC-1 and AsPC-1 cell lines (Figures 8a, S94–S96). The analysis shows that both AsPC-1 and PANC-1 express the NQO1 enzyme, while only AsPC-1 expresses the 5-LO enzyme (Figure 8a). We also see that PANC-1 seems to exhibit lower levels of NQO1 expression compared to those of AsPC-1. This is an interesting observation since the cell viability assays in Figure 7 show that the prodrugs and β-lapachone itself are consistently more toxic in the PANC-1 cell line compared to the AsPC-1 cell line. This indicates a potential alternative mechanism of action occurring in the PANC-1 cell line that is responsible for β-lapachone’s toxic effects. Literature shows that there are other enzymes that also reduce β-lapachone in a similar manner to NQO1, including ferroptosis suppressor protein 1 (FSP1)^90^ and cytochrome P450 reductases.^91^ Additionally, new research has demonstrated that β-lapachone inhibits the activity of thioredoxin reductase 1 (TrxR1),^92^ which allows cells to maintain redox balance and recover from oxidative stress.^93^ Both FSP1 and TrxR1 have been found to be expressed in the PANC-1 and AsPC-1 cell lines and could contribute to the difference in potency of β-lapachone seen between the cell lines.^94,95^

**Figure 8:**
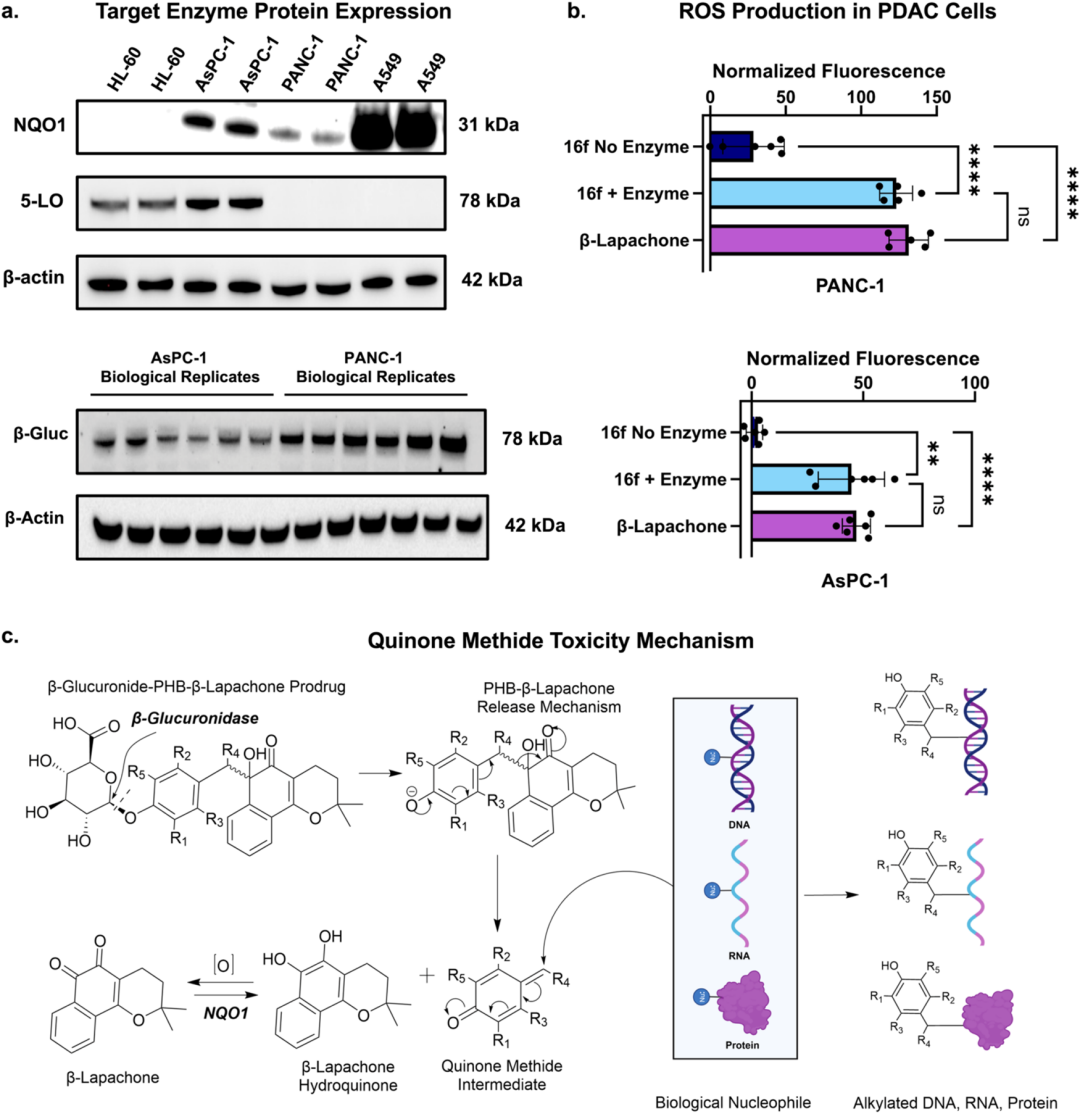
Target expression, ROS production, and quinone methide toxicity in PDAC Cell Lines. Panel (a) shows representative Western blot bands of varying expression levels of the prodrug target enzymes NQO1, 5-LO, and β-glucuronidase from whole cell lysates of PANC-1 and AsPC-1 with β-actin as a loading control. The A549 cell line was used as a positive control for NQO1 enzyme expression, and the HL-60 cell line was used as a positive control for 5-LO enzyme expression. For β-glucuronidase expression, two consecutive technical replicates are shown for each biological replicate, with all three biological replicates represented for both cell lines. Panel (b) displays the reactive oxygen species generation after treatment with **16f** with and without β-glucuronidase enzyme, compared to β-lapachone for 24 h. Results were normalized to baseline cellular ROS activity as 0 and positive control *tert*-butyl hydrogen peroxide (TBHP) as 100. Adjusted *p*-values of 0.0012 (**) and <0.0001 (****) are shown for statistically significant comparisons. Panel (c) shows the hypothesized mechanism of increased cellular toxicity from the quinone methide intermediate.

β-glucuronidase is found within the lysosomes of most cells,^69^ so we expected to also find this enzyme expressed within the two PDAC cell lines tested. Intracellular β-glucuronidase expression can prove useful in determining the potential β-glucuronidase levels released by necrotic cells *in vivo* when they excrete their cellular contents into the TME. Additionally, if an internalizing carrier is attached to our prodrugs via bioconjugation handles incorporated into the design, then they would make use of this intracellular lysosomal β-glucuronidase expression to trigger drug release. Both PDAC cell lines tested are shown to have β-glucuronidase expressed intracellularly (Figure 8a), with PANC-1 having slightly higher levels. Thus, the prodrugs can take advantage of a self-amplifying release mechanism. Once β-lapachone is released from the prodrugs by extracellular β-glucuronidase, it will exert a killing effect on a portion of the tumor. The necrotic cells then release their intracellular β-glucuronidase into the TME, which can act as the enzymatic trigger to release more β-lapachone from the prodrug. The confirmation of β-glucuronidase levels within these cell lines helps inform us of ideal targeting carriers and *in vivo* PDAC models to test the prodrugs in future experiments. Since increased β-glucuronidase levels have already been established in PDAC tumors, this finding indicates that we expect additional β-glucuronidase release from initial cell killing, allowing for selective prodrug activation and a self-amplified cell killing effect at the site of the tumor.

Additionally, we were able to show that ROS species are generated in both the PANC-1 and AsPC-1 cell lines after treatment with one of the lead compounds **16f** for 24 h. Without extracellular β-glucuronidase enzyme added, **16f** generates significantly less ROS than β-lapachone alone, supporting the conclusion that C-alkylation of the *ortho*-quinone carbonyl blocks the redox cycling of the pharmacophore^59^ (Figures 8b, S93). The restored generation of ROS after β-glucuronidase addition corroborates that the prodrug mechanism of cell killing is through ROS generation by the released, active, β-lapachone drug, most likely via interaction of a combination of the various reductive enzymes mentioned, including NQO1, FSP1, and cytochrome P450 reductases.

Lastly, as seen in Figure 7, we hypothesize that the increased cytotoxic effects of the prodrugs in cell lines compared to β-lapachone alone result from an additional toxic mechanism of the linker itself. The highly electrophilic quinone methide intermediate produced from the PHB linker upon 1,6-elimination during β-lapachone release is quenched by a nucleophile and aromatized into an alkylated phenol/phenolate product (Figure 8c).^72,87,96^ In a cellular environment, these nucleophiles likely include essential DNA, RNA, proteins, and lipids, which form alkylated products that are toxic to cells.^87^ Through this mechanism, the PHB SIL can act as an additional toxic agent in our prodrug format, allowing for even greater potency than β-lapachone alone (Figure 8c).

This mechanism must be investigated further to better understand its cytotoxic role and how specific cell lines may be more susceptible toward this mechanism of action.

## Conclusion

In summary, we designed an improved β-lapachone prodrug platform using β-glucuronide-triggered self-immolative *para*-hydroxybenzyl linkers attached via the C-alkylation of the *ortho*-quinone carbonyl. Development and application of an indium-mediated Barbier reaction to attach the glucuronide linker to the *ortho*-quinone carbonyl proved essential to facilitate the synthesis of these prodrug derivatives. The β-glucuronide moiety not only helped to solubilize the prodrugs but also widened their TW by minimizing off-target cellular uptake.

Upon glucuronide removal by extracellular β-glucuronidase, which is found exclusively in the TME,^66–69,77,97,98^ a pH-dependent 1,6-elimination occurs, breaking the C–C bond between the quinone and the linker and liberating the active β-lapachone drug. By attaching EDGs to the SIL, the release rate of β-lapachone was increased by several orders of magnitude, reaching clinically relevant rates and fully restoring cellular efficacy for lead compounds **16f** and **16i**. This computer-aided design also proved successful at accelerating the release rate of the PAB linker in model compound **22** without altering its pH dependence profile, suggesting a prodrug using this optimized linker design would be superior relative to the first-generation prodrugs developed by Dunsmore et al.^59^ The glucuronide prodrugs were then compared to the previously reported boronate prodrug **24**.^60^ **16a**, analogous in intermediate structure to **24**, released at approximately the same rate as **24**, contradicting the release rate findings reported by Gong et al.^60^ **24** also rapidly hydrolyzed into **26**, which was less stable at physiological pH relative to all the glucuronide derivatives studied. All prodrug derivatives performed as expected based on kinetics and stability data in two highly characterized pancreatic cancer cell line models. Additionally, we were able to further investigate potential mechanisms of action of the prodrug in these cell lines through β-lapachone target enzyme expression and ROS analysis. These findings highlight the potential of this prodrug-linker platform for translation into targeted therapies for pancreatic cancer. The optimized β-lapachone prodrugs developed in this study represent the best-performing *ortho*-quinone prodrugs developed to date, with bioconjugation to PDAC-targeted carriers and *in vivo* testing of the identified lead compounds underway.

## Supplementary Material



## Notes

Additional experimental details, compound synthesis and characterization (HPLC, mass spectrometry, and NMR), and supplemental figures (PDF)

## ∇

Dartmouth Health/Dartmouth Cancer Center/Geisel School of Medicine, One Medical Center Drive, Lebanon, New Hampshire 03753, United States

## References

[1] Dias R. B., de Araújo T. B. S., de Freitas R. D., Rodrigues A. C. B. d. C., Sousa L. P., Sales C. B. S., Valverde L. de F., Soares M. B. P., dos
Reis M. G., Coletta R. D., Ramos E. A. G., Camara C. A., Silva T. M. S., Filho J. M. B., Bezerra D. P., Rocha C. A. G. (2018). β-Lapachone
and Its Iodine Derivatives Cause Cell Cycle Arrest at G2/M Phase and
Reactive Oxygen Species-Mediated Apoptosis in Human Oral Squamous
Cell Carcinoma Cells. Free Radical Biol. Med..

[2] da
Silva E. N., Cavalcanti B. C., Guimarães T. T., Pinto M. d. C. F. R., Cabral I. O., Pessoa C., Costa-Lotufo L. V., de Moraes M. O., de Andrade C. K. Z., dos Santos M. R., de Simone C. A., Goulart M. O. F., Pinto A. V. (2011). Synthesis and Evaluation
of Quinonoid Compounds against Tumor Cell Lines. Eur. J. Med. Chem..

[3] Vieira A. A., Brandão I. R., Valença W. O., de Simone C. A., Cavalcanti B. C., Pessoa C., Carneiro T. R., Braga A. L., da Silva E. N. (2015). Hybrid Compounds with Two Redox Centres:
Modular Synthesis
of Chalcogen-Containing Lapachones and Studies on Their Antitumor
Activity. Eur. J. Med. Chem..

[4] Planchon S. M., Wuerzberger S., Frydman B., Witiak D. T., Hutson P., Church D. R., Wilding G., Boothman D. A. (1995). β-Lapachone-Mediated
Apoptosis in Human Promyelocytic Leukemia (HL-60) and Human Prostate
Cancer Cells: A P53-Independent Response. Cancer
Res..

[5] Bian J., Xu L., Deng B., Qian X., Fan J., Yang X., Liu F., Xu X., Guo X., Li X., Sun H., You Q., Zhang X. (2015). Synthesis and Evaluation of (±)-Dunnione and Its
Ortho-Quinone Analogues as Substrates for NAD­(P)­H:Quinone Oxidoreductase
1 (NQO1). Bioorg. Med. Chem. Lett..

[6] Li L. S., Bey E. A., Dong Y., Meng J., Patra B., Yan J., Xie X.-J., Brekken R. A., Barnett C. C., Bornmann W. G., Gao J., Boothman D. A. (2011). Modulating Endogenous NQO1 Levels Identifies Key Regulatory
Mechanisms of Action of β-Lapachone for Pancreatic Cancer Therapy. Clin. Cancer Res..

[7] Ross D., Siegel D. (2017). Functions of NQO1 in
Cellular Protection and CoQ10
Metabolism and Its Potential Role as a Redox Sensitive Molecular Switch. Front. Physiol..

[8] Bolton J. L., Dunlap T. (2017). Formation and Biological
Targets of Quinones: Cytotoxic
versus Cytoprotective Effects. Chem. Res. Toxicol..

[9] Mancini I., Vigna J., Sighel D., Defant A. (2022). Hybrid Molecules Containing
Naphthoquinone and Quinolinedione Scaffolds as Antineoplastic Agents. Molecules.

[10] Siegel R. L., Giaquinto A. N., Jemal A. (2024). Cancer Statistics,
2024. Ca-Cancer J. Clin..

[11] Kleeff J., Korc M., Apte M., La Vecchia C., Johnson C. D., Biankin A. V., Neale R. E., Tempero M., Tuveson D. A., Hruban R. H., Neoptolemos J. P. (2016). Pancreatic
Cancer. Nat. Rev. Dis. Primers.

[12] Hartwig W., Werner J., Jäger D., Debus J., Büchler M. W. (2013). Improvement
of Surgical Results for Pancreatic Cancer. Lancet
Oncol..

[13] Khorana A. A., Mangu P. B., Berlin J., Engebretson A., Hong T. S., Maitra A., Mohile S. G., Mumber M., Schulick R., Shapiro M., Urba S., Zeh H. J., Katz M. H. G. (2017). Potentially Curable Pancreatic Cancer: American Society
of Clinical Oncology Clinical Practice Guideline Update. J. Clin. Oncol..

[14] Von
Hoff D. D., Ramanathan R. K., Borad M. J., Laheru D. A., Smith L. S., Wood T. E., Korn R. L., Desai N., Trieu V., Iglesias J. L., Zhang H., Soon-Shiong P., Shi T., Rajeshkumar N. V., Maitra A., Hidalgo M. (2011). Gemcitabine
Plus Nab-Paclitaxel Is an Active Regimen in Patients With Advanced
Pancreatic Cancer: A Phase I/II Trial. J. Clin.
Oncol..

[15] Conroy T., Desseigne F., Ychou M., Bouché O., Guimbaud R., Bécouarn Y. (2011). FOLFIRINOX versus Gemcitabine
for Metastatic Pancreatic Cancer. N. Engl. J.
Med..

[16] Ho W. J., Jaffee E. M., Zheng L. (2020). The Tumour Microenvironment in Pancreatic
Cancer — Clinical Challenges and Opportunities. Nat. Rev. Clin. Oncol..

[17] Bentle M. S., Bey E. A., Dong Y., Reinicke K. E., Boothman D. A. (2006). New Tricks
for Old Drugs: The Anticarcinogenic Potential of DNA Repair Inhibitors. J. Mol. Histol..

[18] Li Y., Sun X., LaMont J. T., Pardee A. B., Li C. J. (2003). Selective Killing
of Cancer Cells by β-Lapachone: Direct Checkpoint Activation
as a Strategy against Cancer. Proc. Natl. Acad.
Sci. U.S.A..

[19] Ferraz
da Costa D. C., Pereira Rangel L., Martins-Dinis M. M. D. d. C., Ferretti G. D. d. S., Ferreira V. F., Silva J. L. (2020). Anticancer
Potential of Resveratrol, β-Lapachone and Their Analogues. Molecules.

[20] Tagliarino C., Pink J. J., Dubyak G. R., Nieminen A.-L., Boothman D. A. (2001). Calcium
Is a Key Signaling Molecule in β-Lapachone-Mediated Cell Death. J. Biol. Chem..

[21] Pink J. J., Planchon S. M., Tagliarino C., Varnes M. E., Siegel D., Boothman D. A. (2000). NAD­(P)­H:Quinone
Oxidoreductase Activity Is the Principal
Determinant of β-Lapachone Cytotoxicity. J. Biol. Chem..

[22] Wuerzberger S. M., Pink J. J., Planchon S. M., Byers K. L., Bornmann W. G., Boothman D. A. (1998). Induction of Apoptosis
in MCF-7: WS8 Breast Cancer
Cells by β-Lapachone. Cancer Res..

[23] Bey E. A., Bentle M. S., Reinicke K. E., Dong Y., Yang C.-R., Girard L., Minna J. D., Bornmann W. G., Gao J., Boothman D. A. (2007). An NQO1- and PARP-1-Mediated
Cell Death Pathway Induced
in Non-Small-Cell Lung Cancer Cells by β-Lapachone. Proc. Natl. Acad. Sci. U.S.A..

[24] Boothman, D. A. ; Gao, J. ; Bey, E. A. ; Dong, Y. Methods of Treating Cancer Comprising Targeting NQOl. WO2012/040492AL, 2012.

[25] Ough M., Lewis A., Bey E. A., Gao J., Ritchie J. M., Bornmann W., Boothman D. A., Oberley L. W., Cullen J. J. (2005). Efficacy
of β-Lapachone in Pancreatic Cancer Treatment: Exploiting the
Novel, Therapeutic Target NQO1. Cancer Biol.
Ther..

[26] Silvers M. A., Deja S., Singh N., Egnatchik R. A., Sudderth J., Luo X., Beg M. S., Burgess S. C., DeBerardinis R. J., Boothman D. A., Merritt M. E. (2017). The NQO1
Bioactivatable
Drug, β-Lapachone, Alters the Redox State of NQO1+ Pancreatic
Cancer Cells, Causing Perturbation in Central Carbon Metabolism. J. Biol. Chem..

[27] Beg M. S., Huang X., Silvers M. A., Gerber D. E., Bolluyt J., Sarode V., Fattah F., Deberardinis R. J., Merritt M. E., Xie X.-J., Leff R., Laheru D., Boothman D. A. (2017). Using a Novel NQO1 Bioactivatable
Drug, β-Lapachone
(ARQ761), to Enhance Chemotherapeutic Effects by Metabolic Modulation
in Pancreatic Cancer. J. Surg. Oncol..

[28] Chakrabarti G., Moore Z. R., Luo X., Ilcheva M., Ali A., Padanad M., Zhou Y., Xie Y., Burma S., Scaglioni P. P., Cantley L. C., DeBerardinis R. J., Kimmelman A. C., Lyssiotis C. A., Boothman D. A. (2015). Targeting Glutamine
Metabolism Sensitizes Pancreatic Cancer to PARP-Driven Metabolic Catastrophe
Induced by β-Lapachone. Cancer Metab..

[29] Breton C. S., Aubry D., Ginet V., Puyal J., Heulot M., Widmann C., Duchosal M. A., Nahimana A. (2015). Combinative Effects
of β-Lapachone and APO866 on Pancreatic Cancer Cell Death through
Reactive Oxygen Species Production and PARP-1 Activation. Biochimie.

[30] Dong Y., Chin S.-F., Blanco E., Bey E. A., Kabbani W., Xie X.-J., Bornmann W. G., Boothman D. A., Gao J. (2009). Intratumoral
Delivery of β-Lapachone via Polymer Implants for Prostate Cancer
Therapy. Clin. Cancer Res..

[31] Jung E. J., Kim H. J., Shin S. C., Kim G. S., Jung J.-M., Hong S. C., Kim C. W., Lee W. S. (2023). β-Lapachone
Exerts Anticancer Effects by Downregulating P53, Lys-Acetylated Proteins,
TrkA, P38 MAPK, SOD1, Caspase-2, CD44 and NPM in Oxaliplatin-Resistant
HCT116 Colorectal Cancer Cells. Int. J. Mol.
Sci..

[32] Hosein A. N., Beg M. S. (2018). Pancreatic Cancer Metabolism: Molecular Mechanisms
and Clinical Applications. Curr. Oncol. Rep..

[33] Awadallah N. S., Dehn D., Shah R. J., Russell Nash S., Chen Y. K., Ross D., Bentz J. S., Shroyer K. R. (2008). NQO1 Expression
in Pancreatic Cancer and Its Potential Use as a Biomarker. Appl. Immunohistochem. Mol. Morphol..

[34] Yang Y., Zhang Y., Wu Q., Cui X., Lin Z., Liu S., Chen L. (2014). Clinical Implications
of High NQO1 Expression in Breast
Cancers. J. Exp. Clin. Cancer Res..

[35] Li Z., Zhang Y., Jin T., Men J., Lin Z., Qi P., Piao Y., Yan G. (2015). NQO1 Protein
Expression Predicts
Poor Prognosis of Non-Small Cell Lung Cancers. BMC Cancer.

[36] Lin L., Sun J., Tan Y., Li Z., Kong F., Shen Y., Liu C., Chen L. (2017). Prognostic Implication
of NQO1 Overexpression in Hepatocellular
Carcinoma. Hum. Pathol..

[37] Cui X., Li L., Yan G., Meng K., Lin Z., Nan Y., Jin G., Li C. (2015). High Expression of NQO1 Is Associated with Poor Prognosis
in Serous Ovarian Carcinoma. BMC Cancer.

[38] Ji M., Jin A., Sun J., Cui X., Yang Y., Chen L., Lin Z. (2017). Clinicopathological
Implications of NQO1 Overexpression in the Prognosis
of Pancreatic Adenocarcinoma. Oncol. Lett..

[39] Rodrigues T., Werner M., Roth J., da Cruz E. H. G., Marques M. C., Akkapeddi P., Lobo S. A., Koeberle A., Corzana F., Júnior E. N. d. S., Werz O., Bernardes G. J. L. (2018). Machine
Intelligence Decrypts β-Lapachone as an Allosteric 5-Lipoxygenase
Inhibitor. Chem. Sci..

[40] Ding X. Z., Iversen P., Cluck M. W., Knezetic J. A., Adrian T. E. (1999). Lipoxygenase
Inhibitors Abolish Proliferation of Human Pancreatic Cancer Cells. Biochem. Biophys. Res. Commun..

[41] Ding X. Z., Kuszynski C. A., El-Metwally T. H., Adrian T. E. (1999). Lipoxygenase Inhibition
Induced Apoptosis, Morphological Changes, and Carbonic Anhydrase Expression
in Human Pancreatic Cancer Cells. Biochem. Biophys.
Res. Commun..

[42] Ding X.-Z., Adrian T. E. (2001). Role of Lipoxygenase Pathways in the Regulation of
Pancreatic Cancer Cell Proliferation and Survival. InflammoPharmacology.

[43] Zhou G. X., Ding X. L., Huang J. F., Zhang H., Wu S. B. (2007). Suppression
of 5-Lipoxygenase Gene Is Involved in Triptolide-Induced Apoptosis
in Pancreatic Tumor Cell Lines. Biochim. Biophys.
Acta, Gen. Subj..

[44] Knab L. M., Schultz M., Principe D. R., Mascarinas W. E., Gounaris E., Munshi H. G., Grippo P. J., Bentrem D. J. (2015). Ablation
of 5-Lipoxygenase Mitigates Pancreatic Lesion Development. J. Surg. Res..

[45] Hennig R., Ding X.-Z., Tong W.-G., Schneider M. B., Standop J., Friess H., Büchler M. W., Pour P. M., Adrian T. E. (2002). 5-Lipoxygenase and Leukotriene B(4)
Receptor Are Expressed in Human Pancreatic Cancers but Not in Pancreatic
Ducts in Normal Tissue. Am. J. Pathol..

[46] Ghosh J. (2008). Targeting
5-Lipoxygenase for Prevention and Treatment of Cancer. Curr. Enzyme Inhib..

[47] Avis I. M., Jett M., Boyle T., Vos M. D., Moody T., Treston A. M., Martínez A., Mulshine J. L. (1996). Growth Control of
Lung Cancer by Interruption of 5-Lipoxygenase-Mediated Growth Factor
Signaling. J. Clin. Invest..

[48] Shureiqi I., Lippman S. M. (2001). Lipoxygenase Modulation
to Reverse Carcinogenesis. Cancer Res..

[49] Ghosh J., Myers C. E. (1998). Inhibition of Arachidonate
5-Lipoxygenase Triggers
Massive Apoptosis in Human Prostate Cancer Cells. Proc. Natl. Acad. Sci. U.S.A..

[50] Nasongkla N., Wiedmann A. F., Bruening A., Beman M., Ray D., Bornmann W. G., Boothman D. A., Gao J. (2003). Enhancement of Solubility
and Bioavailability of β-Lapachone Using Cyclodextrin Inclusion
Complexes. Pharm. Res..

[51] Gerber D. E., Beg M. S., Fattah F., Frankel A. E., Fatunde O., Arriaga Y., Dowell J. E., Bisen A., Leff R. D., Meek C. C., Putnam W. C., Kallem R. R., Subramaniyan I., Dong Y., Bolluyt J., Sarode V., Luo X., Xie Y., Schwartz B., Boothman D. A. (2018). Phase 1 Study of ARQ 761, a β-Lapachone
Analogue That Promotes NQO1-Mediated Programmed Cancer Cell Necrosis. Br. J. Cancer.

[52] Khong H. T., Dreisbach L., Kindler H. L., Trent D. F., Jeziorski K. G., Bonderenko I., Popiela T., Yagovane D. M., Dombal G. (2007). A Phase 2
Study of ARQ 501 in Combination with Gemcitabine in Adult Patients
with Treatment Naïve, Unresectable Pancreatic Adenocarcinoma. J. Clin. Oncol..

[53] Beg M. S., Boothman D., Khosama L., Arriaga Y., Verma U., Sanjeeviaiah A., Kazmi S., Fattah F., Pilarski S., Rodriguez M., Lindsey D., Linden S., Schwartz B., Laheru D. (2019). A Phase I/Ib, Multi-Center Trial of ARQ-761 (β-Lapachone)
with Gemcitabine/Nab-Paclitaxel in Patients with Advanced Pancreatic
Cancer. Ann. Oncol..

[54] Ma X., Huang X., Moore Z., Huang G., Kilgore J. A., Wang Y., Hammer S., Williams N. S., Boothman D. A., Gao J. (2015). Esterase-Activatable β-Lapachone Prodrug Micelles for NQO1-Targeted
Lung Cancer Therapy. J. Controlled Release.

[55] Ma X., Huang X., Huang G., Li L., Wang Y., Luo X., Boothman D. A., Gao J. (2014). Prodrug Strategy
to Achieve Lyophilizable,
High Drug Loading Micelle Formulations Through Diester Derivatives
of β-Lapachone. Adv. Healthcare Mater..

[56] Reinicke K. E., Bey E. A., Bentle M. S., Pink J. J., Ingalls S. T., Hoppel C. L., Misico R. I., Arzac G. M., Burton G., Bornmann W. G., Sutton D., Gao J., Boothman D. A. (2005). Development
of β-Lapachone Prodrugs for Therapy Against Human Cancer Cells
with Elevated NAD­(P)­H:Quinone Oxidoreductase 1 Levels. Clin. Cancer Res..

[57] Zhou Y., Dong Y., Huang G., Wang Y., Huang X., Zhang F., Boothman D. A., Gao J., Liang W. (2016). Lysosome-Oriented,
Dual-Stage pH-Responsive Polymeric Micelles for β-Lapachone
Delivery. J. Mater. Chem. B.

[58] Gao, J. ; Boothman, D. ; Zhou, Y. ; Bey, E. Ph-Sensitive Compositions for Delivery of Beta Lapachone and Methods of Use, March 29, 2012. https://patentscope.wipo.int/search/en/detail.jsf?docId=WO2012039855&tab=PCTBIBLIO&_cid=P10-KCLOIO-25729-1 (accessed 2020–07–14).

[59] Dunsmore L., Navo C. D., Becher J., de Montes E. G., Guerreiro A., Hoyt E., Brown L., Zelenay V., Mikutis S., Cooper J., Barbieri I., Lawrinowitz S., Siouve E., Martin E., Ruivo P. R., Rodrigues T., da Cruz F. P., Werz O., Vassiliou G., Ravn P., Jiménez-Osés G., Bernardes G. J. L. (2022). Controlled
Masking and Targeted Release of Redox-Cycling Ortho-Quinones via a
C–C Bond-Cleaving 1,6-Elimination. Nat.
Chem..

[60] Gong Q., Li X., Li T., Wu X., Hu J., Yang F., Zhang X. (2022). A Carbon-Carbon Bond Cleavage-Based
Prodrug Activation Strategy Applied
to β-Lapachone for Cancer-Specific Targeting. Angew. Chem., Int. Ed..

[61] Walther R., Rautio J., Zelikin A. N. (2017). Prodrugs
in Medicinal Chemistry and
Enzyme Prodrug Therapies. Adv. Drug Delivery
Rev..

[62] Kratz F., Müller I. A., Ryppa C., Warnecke A. (2008). Prodrug Strategies
in Anticancer Chemotherapy. ChemMedChem.

[63] Leu Y.-L., Roffler S. R., Chern J.-W. (1999). Design and Synthesis of Water-Soluble
Glucuronide Derivatives of Camptothecin for Cancer Prodrug Monotherapy
and Antibody-Directed Enzyme Prodrug Therapy (ADEPT). J. Med. Chem..

[64] Jeffrey S. C., De Brabander J., Miyamoto J., Senter P. D. (2010). Expanded Utility
of the β-Glucuronide Linker: ADCs That Deliver Phenolic Cytotoxic
Agents. ACS Med. Chem. Lett..

[65] Wang Y., Xu K., Liu H., Zhang W., Hu C., Li Y. (2023). Design, Synthesis
of Auristatins-Glucuronide Conjugates Targeting the β-Glucuronidase
in Tumor Microenvironment. Bioorg. Med. Chem.
Lett..

[66] Prijovich Z. M., Burnouf P.-A., Chou H.-C., Huang P.-T., Chen K.-C., Cheng T.-L., Leu Y.-L., Roffler S. R. (2016). Synthesis and Antitumor
Properties of BQC-Glucuronide, a Camptothecin Prodrug for Selective
Tumor Activation. Mol. Pharmaceutics.

[67] Chen K.-C., Schmuck K., Tietze L. F., Roffler S. R. (2013). Selective Cancer
Therapy by Extracellular Activation of a Highly Potent Glycosidic
Duocarmycin Analogue. Mol. Pharmaceutics.

[68] Rooseboom M., Commandeur J. N. M., Vermeulen N. P. E. (2004). Enzyme-Catalyzed Activation of Anticancer
Prodrugs. Pharmacol. Rev..

[69] Tranoy-Opalinski I., Legigan T., Barat R., Clarhaut J., Thomas M., Renoux B., Papot S. (2014). β-Glucuronidase-Responsive
Prodrugs for Selective Cancer Chemotherapy: An Update. Eur. J. Med. Chem..

[70] Sperker B., Werner U., Mürdter T. E., Tekkaya C., Fritz P., Wacke R., Adam U., Gerken M., Drewelow B., Kroemer H. K. (2000). Expression and Function
of β-Glucuronidase in
Pancreatic Cancer: Potential Role in Drug Targeting. Naunyn. Schmiedebergs Arch. Pharmacol..

[71] Pan X., Wang H., Li C., Zhang J. Z. H., Ji C. (2021). MolGpka: A
Web Server for Small Molecule pKa Prediction Using a Graph-Convolutional
Neural Network. J. Chem. Inf. Model..

[72] Alouane A., Labruère R., Le Saux T., Schmidt F., Jullien L. (2015). Self-Immolative
Spacers: Kinetic Aspects, Structure–Property Relationships,
and Applications. Angew. Chem., Int. Ed..

[73] Hay M. P., Sykes B. M., Denny W. A., O’Connor C. J. (1999). Substituent
Effects on the Kinetics of Reductively-Initiated Fragmentation of
Nitrobenzyl Carbamates Designed as Triggers for Bioreductive Prodrugs. J. Chem. Soc., Perkin Trans. 1.

[74] Rose D. A., Treacy J. W., Yang Z. J., Ko J. H., Houk K. N., Maynard H. D. (2022). Self-Immolative
Hydroxybenzylamine Linkers for Traceless
Protein Modification. J. Am. Chem. Soc..

[75] Kaye E. G., Mirabi B., Lopez-Miranda I. R., Dissanayake K. C., Banerjee U., Austin M., Lautens M., Winter A. H., Beharry A. A. (2023). Photo-Uncaging by C­(Sp3)–C­(Sp3) Bond Cleavage
Restores β-Lapachone Activity. J. Am.
Chem. Soc..

[76] Nair V., Jayan C. N., Ros S. (2001). Novel Reactions of Indium Reagents
with 1,2-Diones: A Facile Synthesis of α-Hydroxy Ketones. Tetrahedron.

[77] Antunes I. F., Haisma H. J., Elsinga P. H., Dierckx R. A., de Vries E. F. J. (2010). Synthesis
and Evaluation of [18F]-FEAnGA as a PET Tracer for β-Glucuronidase
Activity. Bioconjugate Chem..

[78] Rofstad E. K., Mathiesen B., Kindem K., Galappathi K. (2006). Acidic Extracellular
pH Promotes Experimental Metastasis of Human Melanoma Cells in Athymic
Nude Mice. Cancer Res..

[79] Sung S.-Y., Hsieh C.-L., Wu D., Chung L. W. K., Johnstone P. A. S. (2007). Tumor
Microenvironment Promotes Cancer Progression, Metastasis, and Therapeutic
Resistance. Curr. Probl. Cancer.

[80] Tannock I. F., Rotin D. (1989). Acid pH in Tumors and
Its Potential for Therapeutic Exploitation. Cancer Res..

[81] Hammond G. S. (1955). A Correlation
of Reaction Rates. J. Am. Chem. Soc..

[82] Marcus R. A. (1964). Chemical
and Electrochemical Electron-Transfer Theory. Annu. Rev. Phys. Chem..

[83] Hayden A. E., Houk K. N. (2009). Transition State
Distortion Energies Correlate with
Activation Energies of 1,4-Dihydrogenations and Diels–Alder
Cycloadditions of Aromatic Molecules. J. Am.
Chem. Soc..

[84] Pross, A. Theoretical and Physical Principles of Organic Reactivity; Wiley: New York, 1995.

[85] Houba P. H. J., Leenders R. G. G., Boven E., Scheeren J. W., Pinedo H. M., Haisma H. J. (1996). Characterization of Novel Anthracycline
Prodrugs Activated by Human β-Glucuronidase for Use in Antibody-Directed
Enzyme Prodrug Therapy. Biochem. Pharmacol..

[86] Cheng T.-L., Chou W.-C., Chen B. M., Chern J.-W., Roffler S. R. (1999). Characterization
of an Antineoplastic Glucuronide Prodrug. Biochem.
Pharmacol..

[87] Grinda M., Clarhaut J., Tranoy-Opalinski I., Renoux B., Monvoisin A., Cronier L., Papot S. (2011). A Heterodimeric
Glucuronide Prodrug
for Cancer Tritherapy: The Double Role of the Chemical Amplifier. ChemMedChem.

[88] Tamargo J., Le Heuzey J.-Y., Mabo P. (2015). Narrow Therapeutic Index Drugs: A
Clinical Pharmacological Consideration to Flecainide. Eur. J. Clin. Pharmacol..

[89] Hansen, B. How Safe is this Drug? - Toxicology Education Foundation. https://toxedfoundation.org/how-safe-is-this-drug/ (accessed 2025–06–10).

[90] Liu F., Li Y., Li Y., Wang Z., Li X., Liu Y., Zhao Y. (2025). Triggering
Multiple Modalities of Cell Death *via* Dual-Responsive
Nanomedicines to Address the Narrow Therapeutic
Window of *β*-Lapachone. J. Colloid Interface Sci..

[91] Cassagnes L.-E., Perio P., Ferry G., Moulharat N., Antoine M., Gayon R., Boutin J. A., Nepveu F., Reybier K. (2015). In Cellulo Monitoring of Quinone Reductase Activity
and Reactive Oxygen Species Production during the Redox Cycling of
1,2 and 1,4 Quinones. Free Radical Biol. Med..

[92] Zhang J., Xu Q., Ma D. (2022). Inhibition
of Thioredoxin Reductase by Natural Anticancer
Candidate β-Lapachone Accounts for Triggering Redox Activation-Mediated
HL-60 Cell Apoptosis. Free Radical Biol. Med..

[93] Torrente L., Prieto-Farigua N., Falzone A., Elkins C. M., Boothman D. A., Haura E. B., DeNicola G. M. (2020). Inhibition of TXNRD or SOD1 Overcomes
NRF2-Mediated Resistance to β-Lapachone. Redox Biol..

[94] Cell line - AIFM2 - The Human Protein Atlas. https://www.proteinatlas.org/ENSG00000042286-AIFM2/cell+line (accessed 2025–02–21).

[95] Rios
Perez M. V., Roife D., Dai B., Pratt M., Dobrowolski R., Kang Y., Li X., Augustine J. J., Zielinski R., Priebe W., Fleming J. B. (2019). Antineoplastic Effects
of Auranofin in Human Pancreatic Adenocarcinoma Preclinical Models. Surg. Open Sci..

[96] Blencowe C. A., Russell A. T., Greco F., Hayes W., Thornthwaite D. W. (2011). Self-Immolative
Linkers in Polymeric Delivery Systems. Polym.
Chem..

[97] F
Antunes I., Haisma H. J., Elsinga P. H., Di Gialleonardo V., Waarde A. van., Willemsen A. T. M., Dierckx R. A., de Vries E. F. J. (2012). Induction
of β-Glucuronidase Release by Cytostatic Agents in Small Tumors. Mol. Pharmaceutics.

[98] Stachulski A. V., Meng X. (2013). Glucuronides from Metabolites to Medicines: A Survey of the in Vivo
Generation, Chemical Synthesis and Properties of Glucuronides. Nat. Prod. Rep..

