# Supplementary material for: Controlled Self-Immolative Release of β‑Lapachone via an Optimized para-Hydroxybenzyl Linker for Targeted Pancreatic Cancer Therapy
Source: J Am Chem Soc. 2025 Dec 15;147(52):48271–89. doi: 10.1021/jacs.5c17598 (PMC12766730; doi:10.1021/jacs.5c17598)
Supplement: Supplementary file 1 [file ja5c17598_si_001.pdf]

Controlled Self-Immolative Release of β-Lapachone via an Optimized *para*-Hydroxybenzyl Linker for Targeted Pancreatic Cancer Therapy

Supporting Information

Julie B. Becher[1‡], Nisita Dutta[1,2,3‡], Claudio D. Navo[4,5], Lavinia Dunsmore[1], Roman Misteli[1],

Enrique Gil de Montes[1], Grant G. Simpson[1], Christine C. Alewine[2†], Gonzalo Jiménez-Osés[4,5],*

Gonçalo J. L. Bernardes[1,6]*

[1]Yusuf Hamied Department of Chemistry, University of Cambridge, Cambridge, CB2 1EW, UK
[2]Laboratory of Molecular Biology, National Cancer Institute, National Institutes of Health, Bethesda, Maryland, 20892, USA
[3]Medical Scientist Training Program, University of Maryland School of Medicine, Baltimore, Maryland, 21201, USA
[4]Center for Cooperative Research in Biosciences (CIC bioGUNE), Basque Research and Technology Alliance (BRTA), Derio, 48160, Spain
[5]Ikerbasque, Basque Foundation for Science, Bilbao, 48013, Spain
[6]Translational Chemical Biology Group, Spanish National Cancer Research Centre 26 (CNIO), Madrid, 28029, Spain

Table of Contents

Section 1. Supplemental figures for introduction 4

Section 2. Supplemental figures for preliminary modeling of pH-dependent cleavage of PHB vs PAB linkers 4

2.1. Derivation of equations for kinetic models 4
2.2. Quantum Mechanics calculations 7

Section 3. Supplemental figures for design and synthesis of prodrug derivatives 8

Section 4. Supplemental figures for release rate comparison of self-immolative linkers 15

4.1. Representative HPLC traces for each derivative at each pH studied. 15
4.2. Linearized equations and fitting of kinetic data for each derivative. 37
4.3. Retention time comparison to known standards for peak identification. 44
4.4. Graphs of calculated k_{obs} on a non-logarithmic scale 52
4.5. Tables of kinetics data 53

Section 5. Supplemental figures for mechanistic discussion and quantum mechanical calculations 60

5.1. Cartesian coordinates of the lowest energy structures calculated at the PCM(H2O)/M06-2X/6-31+G(d,p) level 66

Section 6. Supplemental figures for stability of lapachone prodrugs 79

6.1. Representative HPLC traces for each derivative at each pH studied. 79
6.2. Prodrug and lapachone peak area graphs for each derivative 92
6.3. Quantified β-lapachone released in prodrug stability assays 99
6.4. Tables of stability data 99

Section 7. Supplemental figures for comparison to boronate ester prodrug 101

7.1. Kinetics figures for boronate ester 101
7.2. LC-MS peak identification for boronate ester experiments. 108
7.3. Stability of Boronate Prodrug 109

Section 8. Supplemental figures for *in vitro* PDAC cellular efficacy 111

Section 9. Supplemental figures for cellular mechanism of prodrug 117

Section 10. Materials and Methods 120

10.1. HPLC Prodrug Stability Assays 120
10.2. HPLC Drug Release Kinetics Assays 120
10.3. Cell Viability Assays 122
10.4. Reactive Oxygen Species Assays 123
10.5. Western Blot for Target Expression 123
10.6. LC-MS Analysis Methodology 124

Section 11. Synthetic Procedures 125

11.1. General Synthetic Information 125
11.2. General Synthetic Methods 125
Glycosylation *125*
Reduction *125*
Chlorination *126*
Bromination *126*
Indium Barbier Reaction *126*
Sugar Deprotection *126*
11.3. Synthetic Procedures for 1 and 27 126
11.4. Synthetic Procedures for 16a 129
11.5. Synthetic Procedures for 16b 135

11.6. Synthetic Procedures for 16c ... 141
11.7. Synthetic Procedures for 16d ... 146
11.8. Synthetic Procedures for 16e ... 152
11.9. Synthetic Procedures for 16f ... 160
11.10. Synthetic Procedures for 16g ... 166
11.11. Synthetic Procedures for 16h ... 175
11.12. Synthetic Procedures for 16i ... 181
11.13. Synthetic Procedures for 16j ... 187
11.14. Synthetic Procedures for 22 ... 194
11.15. Synthetic Procedures for 24 and 26 ... 198

Section 12. NMR Spectra of Synthesized Compounds ... 202

Section 13. References ... 263

Section 1. Supplemental figures for introduction

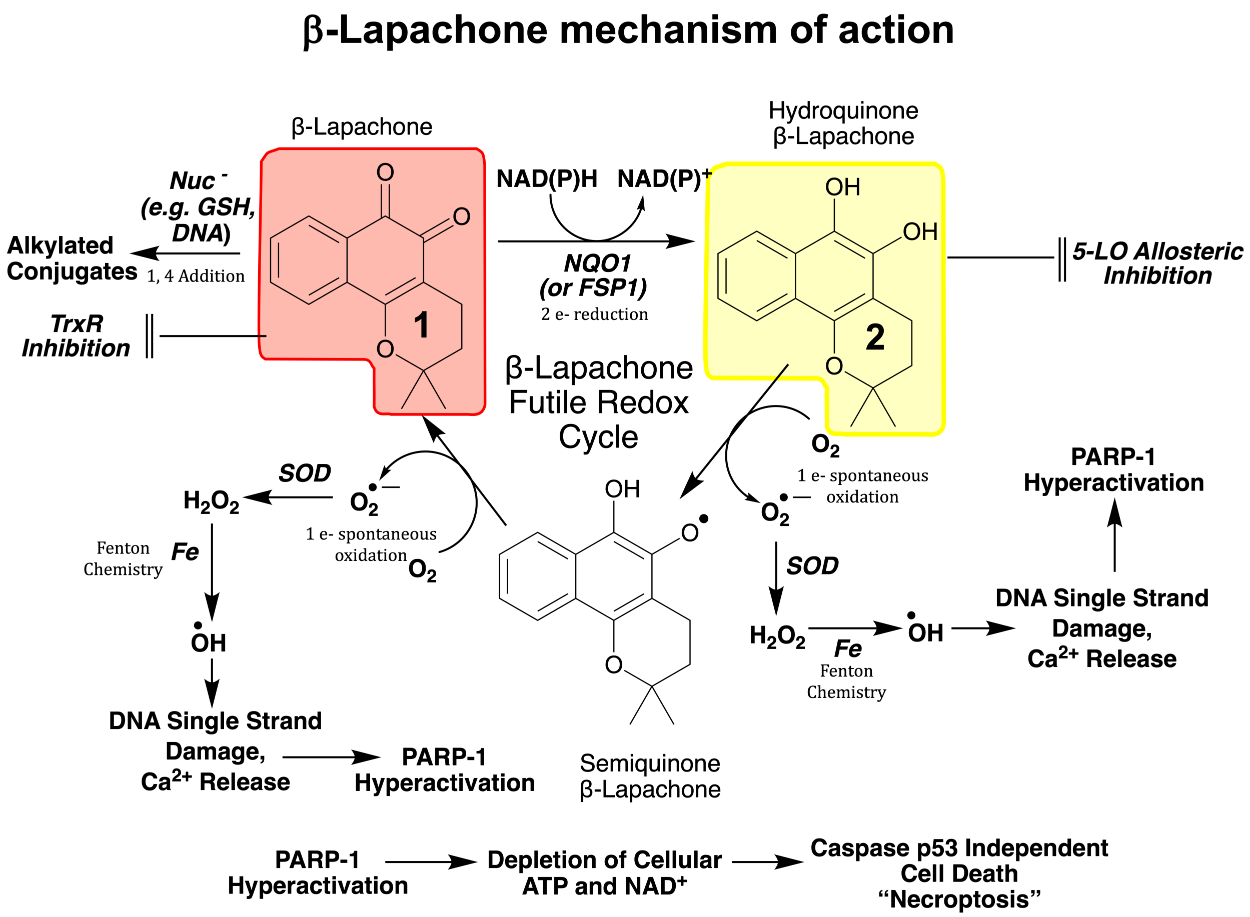

Figure S1. β-Lapachone Mechanism of Action. β-Lapachone (**1**) undergoes a futile redox cycle upon reduction by NQO1 to **2**. Recently, FSP1 has also been shown to perform this reduction.[1] This cycle creates ROS which damages DNA, leading to PARP-1 hyperactivation, depleting cellular ATP and NAD$^+$, and ultimately leading to necroptosis.[2–5] The hydroquinone form (**2**) can also inhibit 5-LO.[6] **1** has also recently been shown to inhibit TrxR.[7]

Section 2. Supplemental figures for preliminary modeling of pH-dependent cleavage of PHB vs PAB linkers

2.1. Derivation of equations for kinetic models

An expression for the theoretical reaction rate constant (k_{theo}) for the PHB prodrug **3a** was derived by applying the previously reported general equation[8], which takes into account several protonated and deprotonated species in equilibrium and the intrinsic rate constants of the reactive species. This expression, in turn, can be related to the experimentally observed rate constant (k_{obs}):

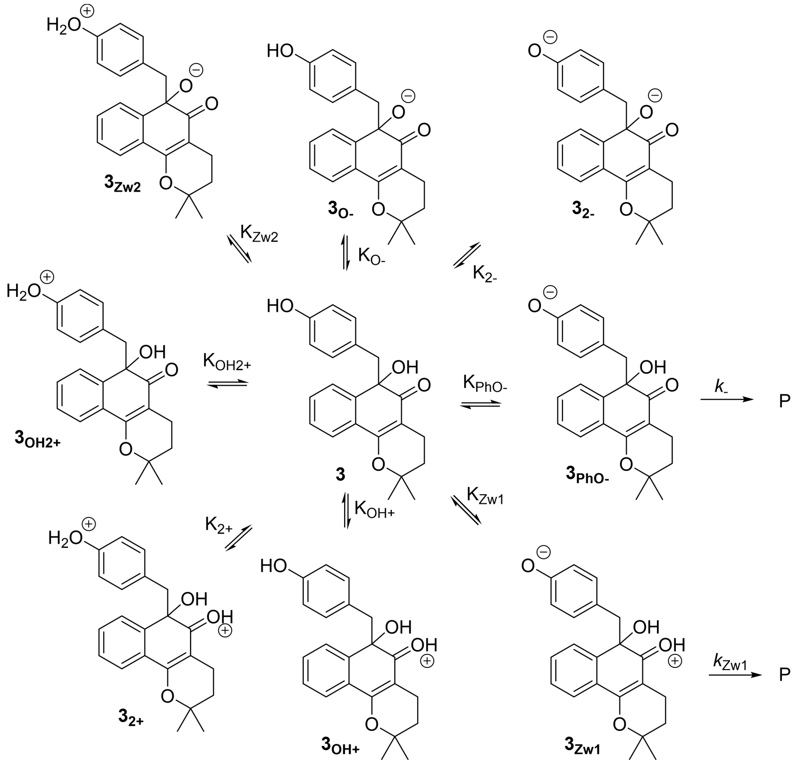

$$K_{\mathrm{OH2+}} = \frac{[3][H^+]}{[3_{\mathrm{OH2+}}]}$$

$$K_{OH+} = \frac{[3][H^+]}{[3_{OH+}]}$$

$$K_{\mathrm{PhO-}} = \frac{[3_{\mathrm{PhO-}}][H^+]}{[3]}$$

$$K_{O-} = \frac{[3_{O-}][H^+]}{[3]}$$

$$K_{Zw1} = \frac{[3_{Zw1}]}{[3]}$$

$$K_{Zw2} = \frac{[3_{Zw2}]}{[3]}$$

$$K_{2-} = \frac{[3_{2-}][H^+]^2}{[3]}$$

$$K_{2+} = \frac{[3][H^+]^2}{[3_{2+}]}$$

$$k_{obs} = \frac{\sum k_r[\mathbf{3}_r]}{[\mathbf{3}_T]} \approx k_{theo} =$$

$$= \frac{k_-[\mathbf{3}_{PhO-}] + k_{zw}[\mathbf{3}_{Zw1}]}{[\mathbf{3}] + [\mathbf{3}_{OH2+}] + [\mathbf{3}_{OH+}] + [\mathbf{3}_{PhO-}] + [\mathbf{3}_{O-}] + [\mathbf{3}_{Zw1}] + [\mathbf{3}_{Zw2}] + [\mathbf{3}_{2-}] + [\mathbf{3}_{2+}]}$$

$$k_{obs} \approx k_{theo}$$

$$= \frac{k_-\frac{K_{PhO^-}}{[H^+]}[\mathbf{3}] + k_{zw}K_{Zw1}[\mathbf{3}]}{[\mathbf{3}] + \frac{[H^+]}{K_{OH_2^+}}[\mathbf{3}] + \frac{[H^+]}{K_{OH^+}}[\mathbf{3}] + \frac{K_{PhO^-}}{[H^+]}[\mathbf{3}] + \frac{K_{O^-}}{[H^+]}[\mathbf{3}] + K_{Zw1}[\mathbf{3}] + K_{Zw2}[\mathbf{3}] + \frac{K_{2-}}{[H^+]^2}[\mathbf{3}] + \frac{[H^+]^2}{K_{2+}}[\mathbf{3}]}$$

$$k_{obs} \approx k_{theo} = \frac{k_-\frac{K_{PhO^-}}{[H^+]} + k_{zw}K_{Zw1}}{1 + \frac{[H^+]}{K_{OH_2^+}} + \frac{[H^+]}{K_{OH^+}} + \frac{K_{PhO^-}}{[H^+]} + \frac{K_{O^-}}{[H^+]} + K_{Zw1} + K_{Zw2} + \frac{K_{2-}}{[H^+]^2} + \frac{[H^+]^2}{K_{2+}}}$$

Equation 1

At typical pH values (1-14), the most predominant species are those whose equilibrium constants (pK_a, in logarithmic form) fall within that range. In this case, the predominant species will be the phenol (**3**), the phenolate (**3**$_{\mathrm{PhO-}}$), and ketolate (**3**$_{\mathrm{O-}}$). Therefore, the contribution of the other species in equation 1can be neglected, leading to reduced equation 2:

$$k_{obs} \approx k_{theo} = \frac{k_-\frac{K_{PhO^-}}{[H^+]} + k_{zw}K_{Zw1}}{1 + \frac{K_{PhO^-}}{[H^+]} + \frac{K_{O^-}}{[H^+]}}$$

Reduced equation 2

Similarly, given the extremely low pK_a of the carbonyl group of β-lapachone, the reactive zwitterion species **3**$_{Zw1}$ is presumed to be negligible, and therefore its contribution to k_{obs} is also negligible. On the other hand, the equilibrium between **3** and ketolate **3**$_{O-}$ groups is predicted to have a pK_a of ~11. Therefore, for pH values below 9, the concentration of this species is negligible and, therefore, simplest equation 3 can be derived:

$$k_{obs} \approx k_{theo} = \frac{k_{-}\frac{K_{PhO^{-}}}{[H^{+}]}}{1+\frac{K_{PhO^{-}}}{[H^{+}]}} = k_{-}\frac{K_{PhO^{-}}}{K_{PhO^{-}}+[H^{+}]}$$

Simplest equation 3

2.2. Quantum Mechanics calculations

Full geometry optimizations and transition structure (TS) searches were carried out with Gaussian 16[9] using the M06-2X hybrid functional[10] and 6-31+G(d,p) basis set in combination with ultrafine integration grids. Bulk solvent effects in water were considered implicitly through the IEF-PCM polarizable continuum model.[11] The possibility of different conformations was taken into account for all structures. Frequency analyses were carried out at the same level used in the geometry optimizations, and the nature of the stationary points was determined in each case according to the appropriate number of negative eigenvalues of the Hessian matrix. Thermal corrections were obtained at 298.15 K. The quasiharmonic approximation reported by Truhlar *et al.* was used to replace the harmonic oscillator approximation for the calculation of the vibrational contribution to enthalpy and entropy.[12] Scaled frequencies were not considered. Mass-weighted intrinsic reaction coordinate (IRC) calculations were carried out using the Hessian-based predictor-corrector integrator scheme by Hratchian and Schlegel[13,14] in order to ensure that the TSs indeed connected the appropriate reactants and products. Gibbs free energies (ΔG) were used for the discussion on the relative stabilities of the considered structures. The lowest energy conformer for each calculated stationary point (Figure S48) was considered in the discussion; all the computed structures can be obtained from authors upon request. Electronic energies, entropies, enthalpies, Gibbs free energies, lowest frequencies and Cartesian coordinates of the calculated structures are summarized in Supplementary Table S5.1.

Section 3. Supplemental figures for design and synthesis of prodrug derivatives

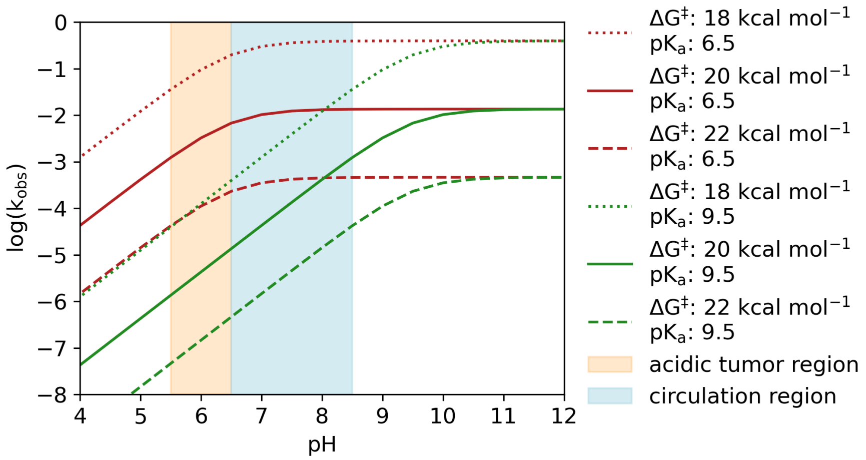

Figure S2. pH-dependence of the observed rate constant (k_{obs}, logarithmic scale; calculated with equation 3) for six hypothetical reactions characterized by different activation free energies ($\Delta G^{\ddagger}$ = 18, 20, and 22 kcal mol^{-1}; dotted, solid and dashed lines, respectively) and pK_a values (6.5 or 9.5; red and green lines, respectively). At acidic pH values corresponding to the tumor microenvironment (pH 5.5–6.5, orange region), a decrease of 3 pK_a units at fixed $\Delta G^{\ddagger}$ shifts the system closer to its maximum k_{obs}, resulting in faster kinetics. In contrast, under near-neutral conditions representative of physiological circulation (pH 6.5–8.5, blue region), the pH dependence levels off and both systems converge to similar k_{obs} values, thus eliminating the kinetic advantage.

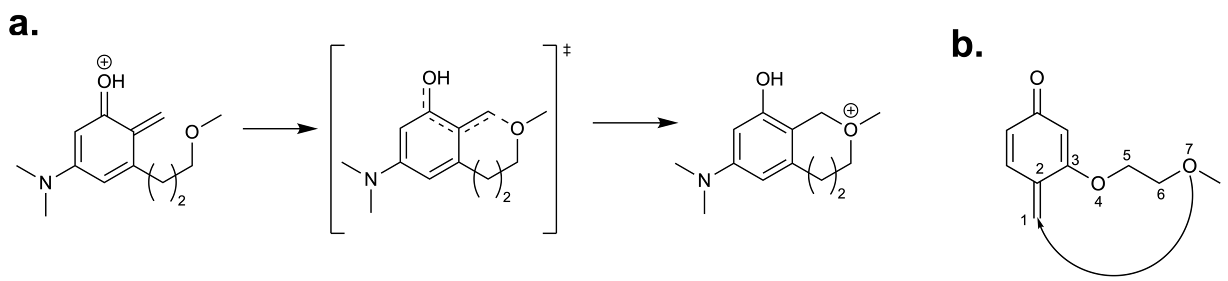

Figure S3. Findings from Rose *et al.* for a Methoxy Tether to Increase Release Rate. (a) This structure, adapted from Rose *et al.*,[15] shows the seven-membered ring between the tether oxygen and the benzylic position of the quinone methide that achieved the greatest increase in the observed release rate of their ammonium leaving group (first step corresponding to C–N bond cleavage not shown). (b) The structure of the PHB linker in **16j** was designed such that the quinone methide could form the same seven-membered ring upon β-lapachone drug release.

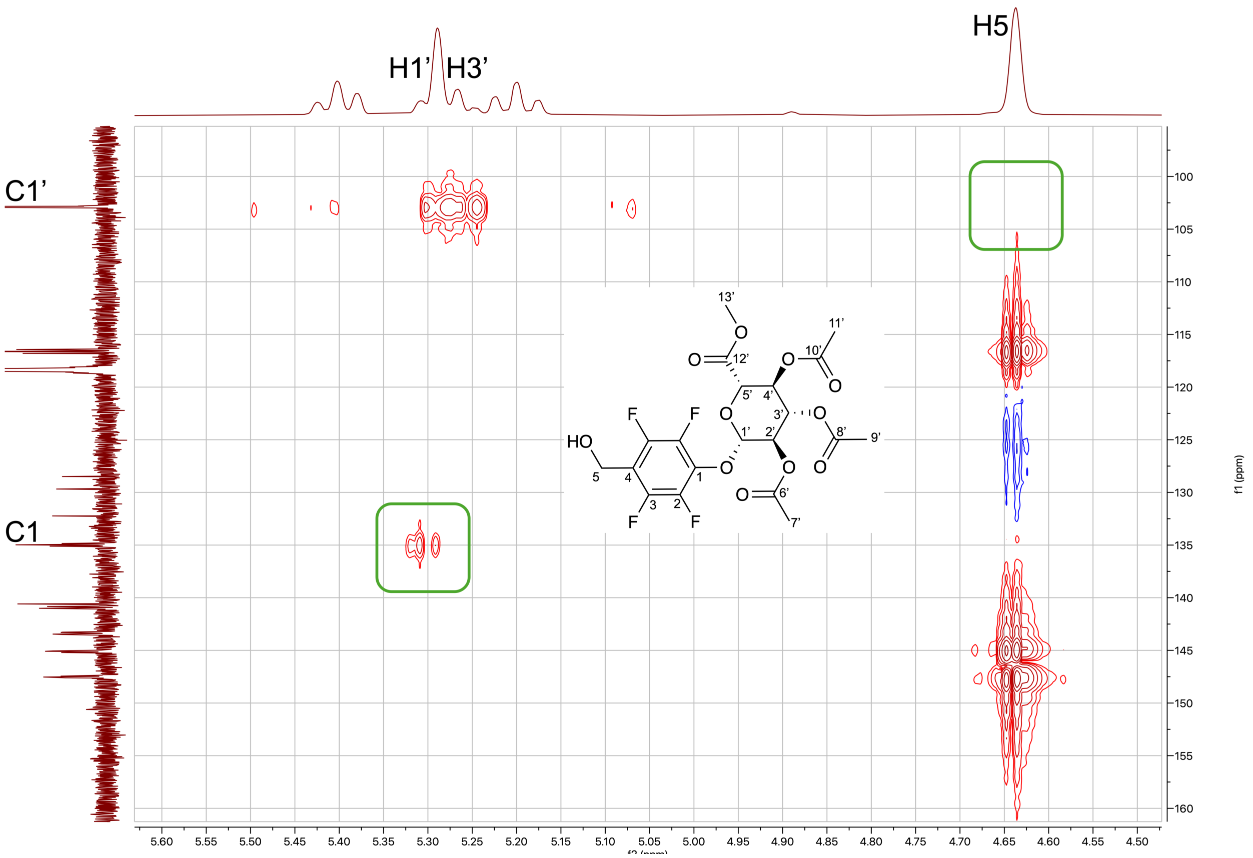

Figure S4. HMBC spectrum of 13c. Cross peaks (green box) between H1' and C1 are visible, while no cross peaks are present between H5 and C1'.

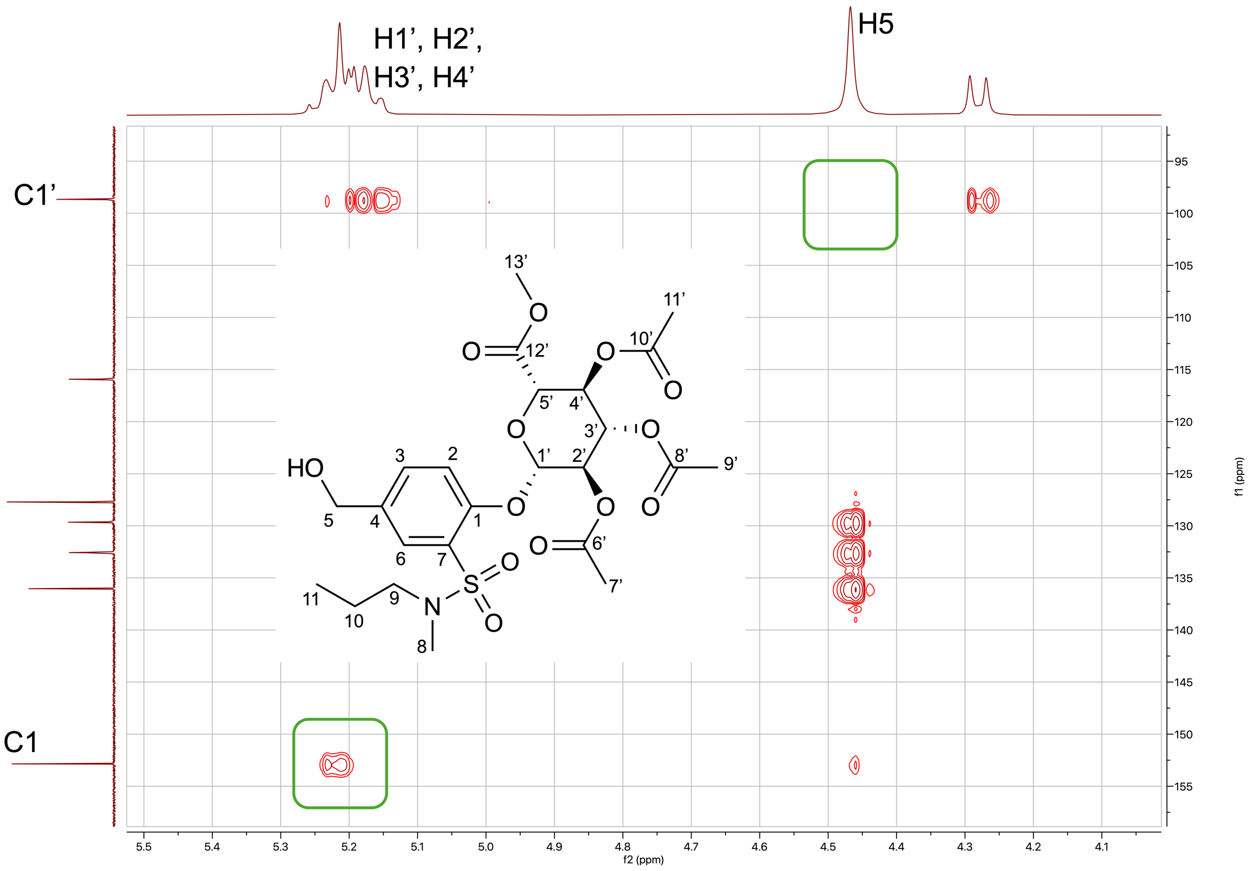

Figure S5. HMBC spectrum of 13e. Cross peaks (green box) between H1' and C1 are visible, while no cross peaks are present between H5 and C1'.

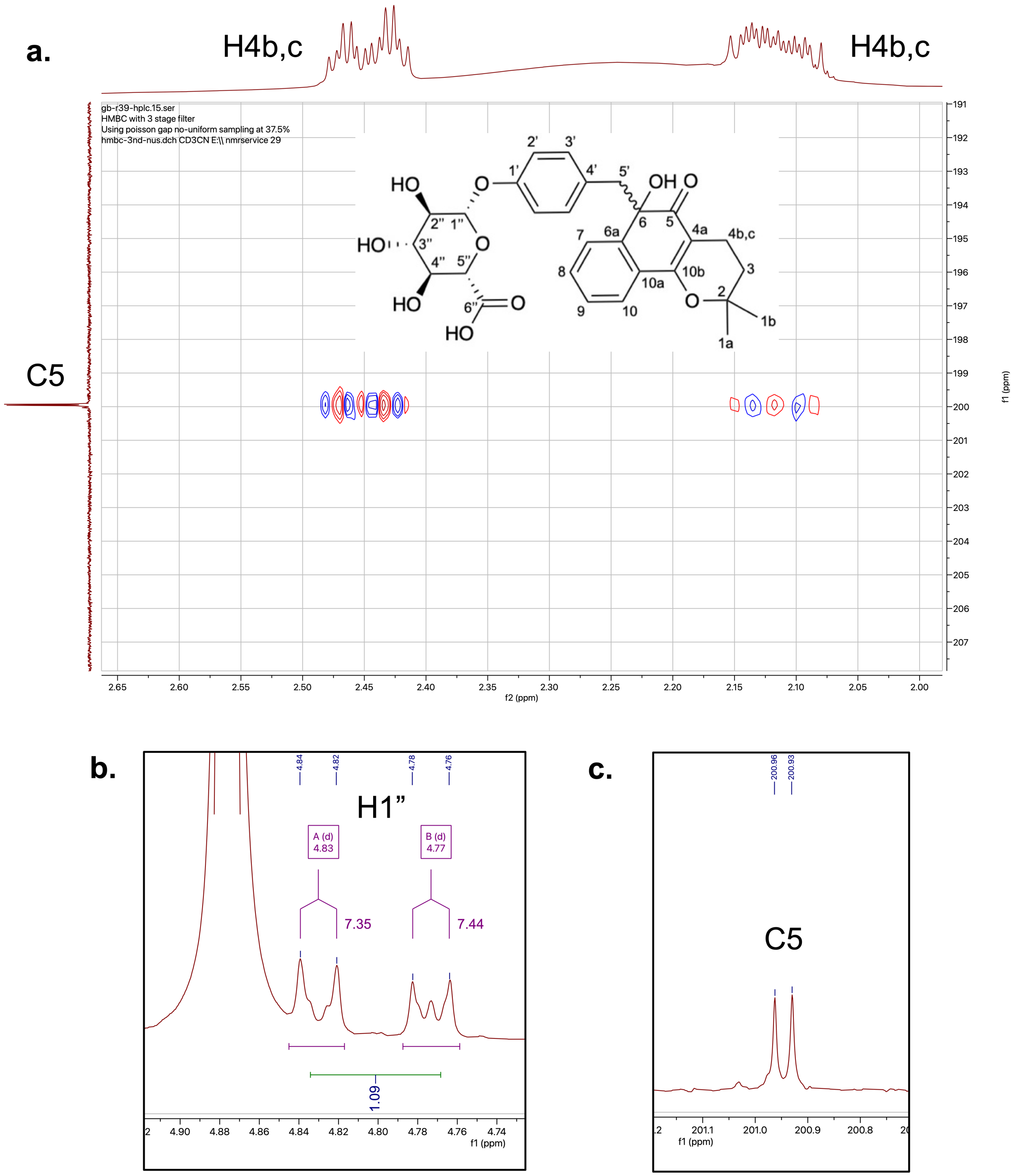

Figure S6. NMRs Proving Regio and Stereochemistry of 16a. (a) Cross peaks between H4b,c and C5 in the HMBC spectrum of **16a** confirms alkylation occurred at the carbonyl adjacent to the benzene ring. (b) In the ^{1}H NMR, the $J_{H1''\text{-}H2''}$ coupling constants of the anomeric proton in each diastereomer of **16a** are shown to be in the 6-10 Hz range,[16] confirming both diastereomers of **16a** have the desired β stereochemistry. (c) Two ^{13}C NMR peaks for C5 are displayed, confirming two diastereomers of **16a** are present in the purified product mixture.

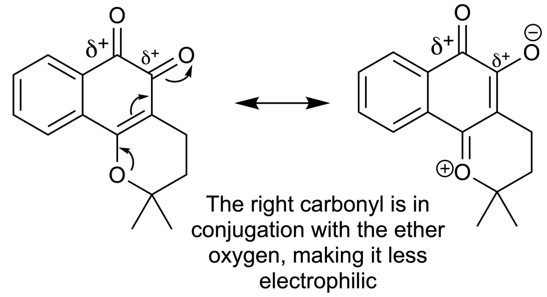

Figure S7. Regioisomer Preference for C-Alkylation of 1. The carbonyl conjugated to the cyclic ether oxygen is hypothesized to be less electrophilic than the carbonyl next to the benzene ring due to the resonance donation of the ether oxygen. This was confirmed by using the Fukui positive (f^+, electrophilic) index. The carbonyl adjacent to the benzene ring has a value of 0.169 eV, while the carbonyl in resonance with the ether oxygen has a lower value of 0.144 eV (calculated using Rowan Scientific).[17,18]

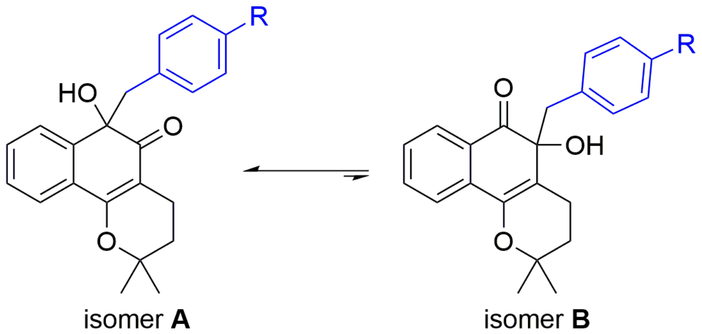

Derivative	Relative stability of isomer B ($\Delta G_{B\text{-}A}$, kcal mol^{-1})	Theoretical ratio A:B
R: OH (PHB)	+7.0	>99:1
R: NH_2 (PAB)	+7.2	>99:1

Figure S8. Equilibrium between isomers A and B for PHB (R = OH) and PAB (R = NH_2) prodrugs. Calculated relative stabilities ($\Delta G_{B\text{–}A}$) indicate that isomer A is strongly favored in both cases, leading to a theoretical A:B ratio of >99:1. The substituent in the *para*-position of the phenyl ring is highlighted in blue.

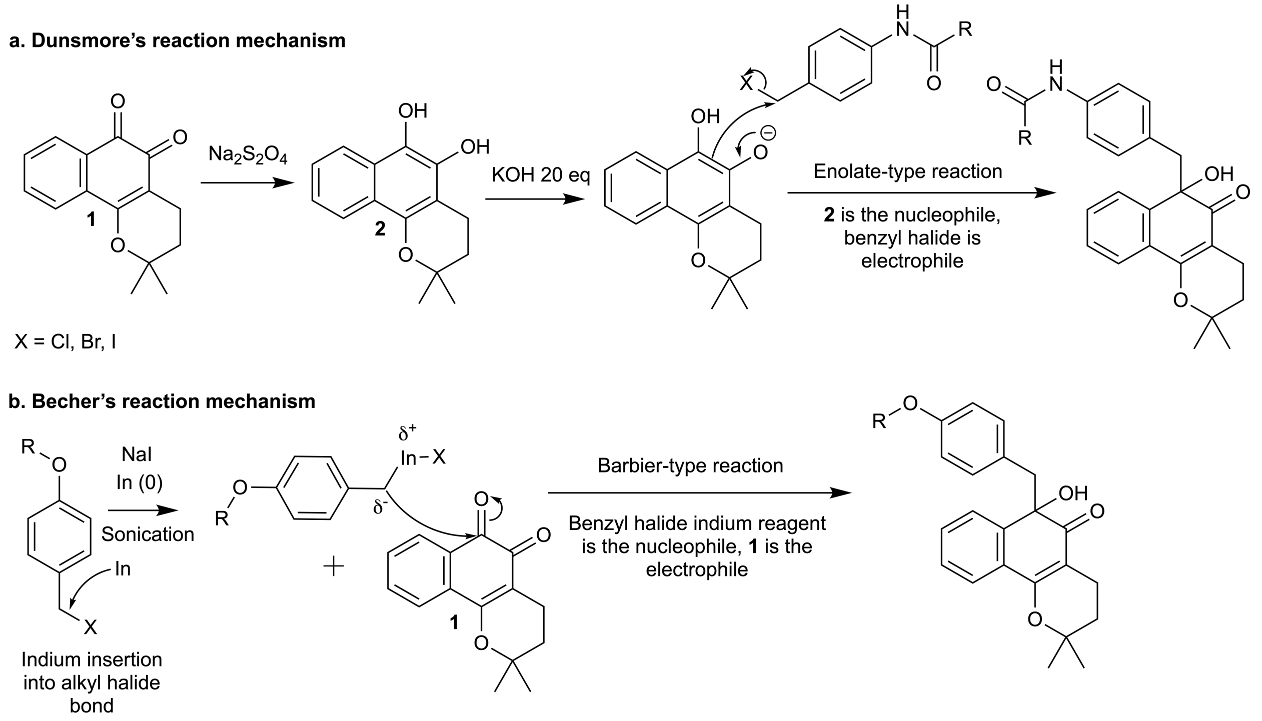

Figure S9. Mechanism comparison between the reaction used by Dunsmore *et al.* and the reaction used by Becher *et al.* to alkylate the carbonyl carbon of the *ortho*-quinone. In Dunsmore's methodology,[8] lapachone is reduced and deprotonated, becoming an enolate-type nucleophile. This then performs an S_N2 attack on the benzyl halide electrophile. In Becher's methodology adapted from Nair *et al.*[19], indium inserts itself into the benzyl halide bond, making the benzylic carbon nucleophilic. This then attacks the lapachone carbonyl electrophile in a Barbier-type reaction.

Table S3.1. Values of Anomeric H Coupling Constants (β = 6-10 Hz[16])

Derivative	Anomeric H (H1") $J_{H1''\text{-}H2''}$ Coupling Constant (Hz)
**16a**	7.4
**16b**	7.3
**16c**	7.5
**16d**	7.2
**16e Major Product**	6.8
**16e Minor Product**	7.5
**16f**	7.3
**16g**	7.4

**16h**	6.6
**16i**	7.6
**16j**	7.3

Section 4. Supplemental figures for release rate comparison of self-immolative linkers

4.1. Representative HPLC traces for each derivative at each pH studied.

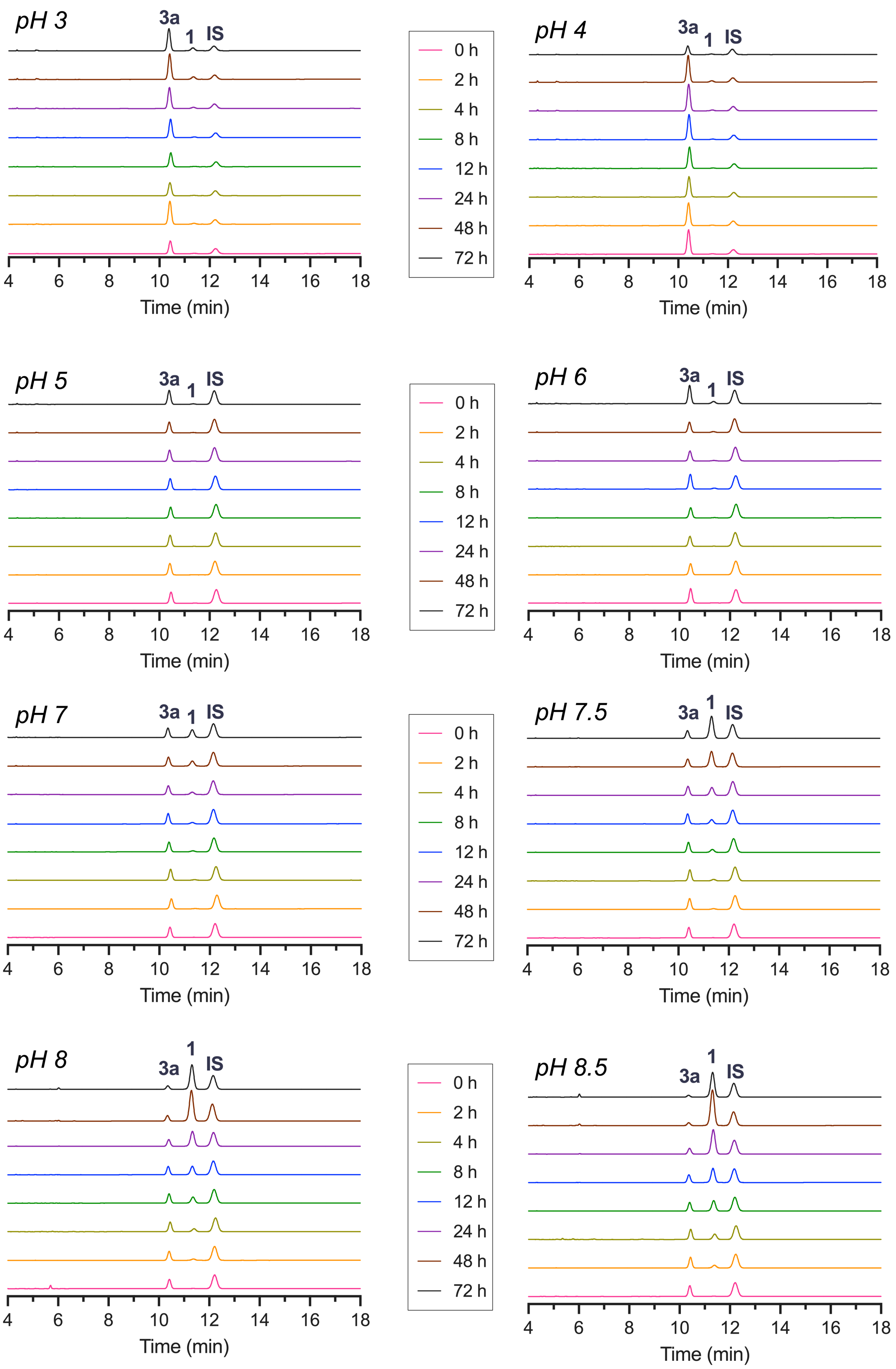

pH 3
pH 4
pH 5
pH 6
pH 7
pH 7.5
pH 8
pH 8.5
3a
1
IS
0 h
2 h
4 h
8 h
12 h
24 h
48 h
72 h
Time (min)

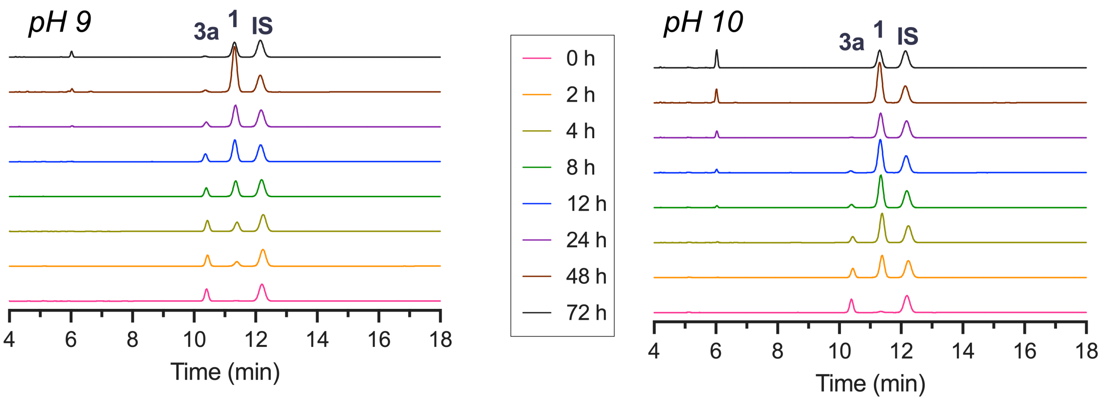

Figure S10. HPLC traces for 16a release experiments. The representative traces shown are one replicate of the triplicate experiments performed. RT **3a** = 10.4 min, RT **1** = 11.3 min, RT warfarin IS 12.2 = min.

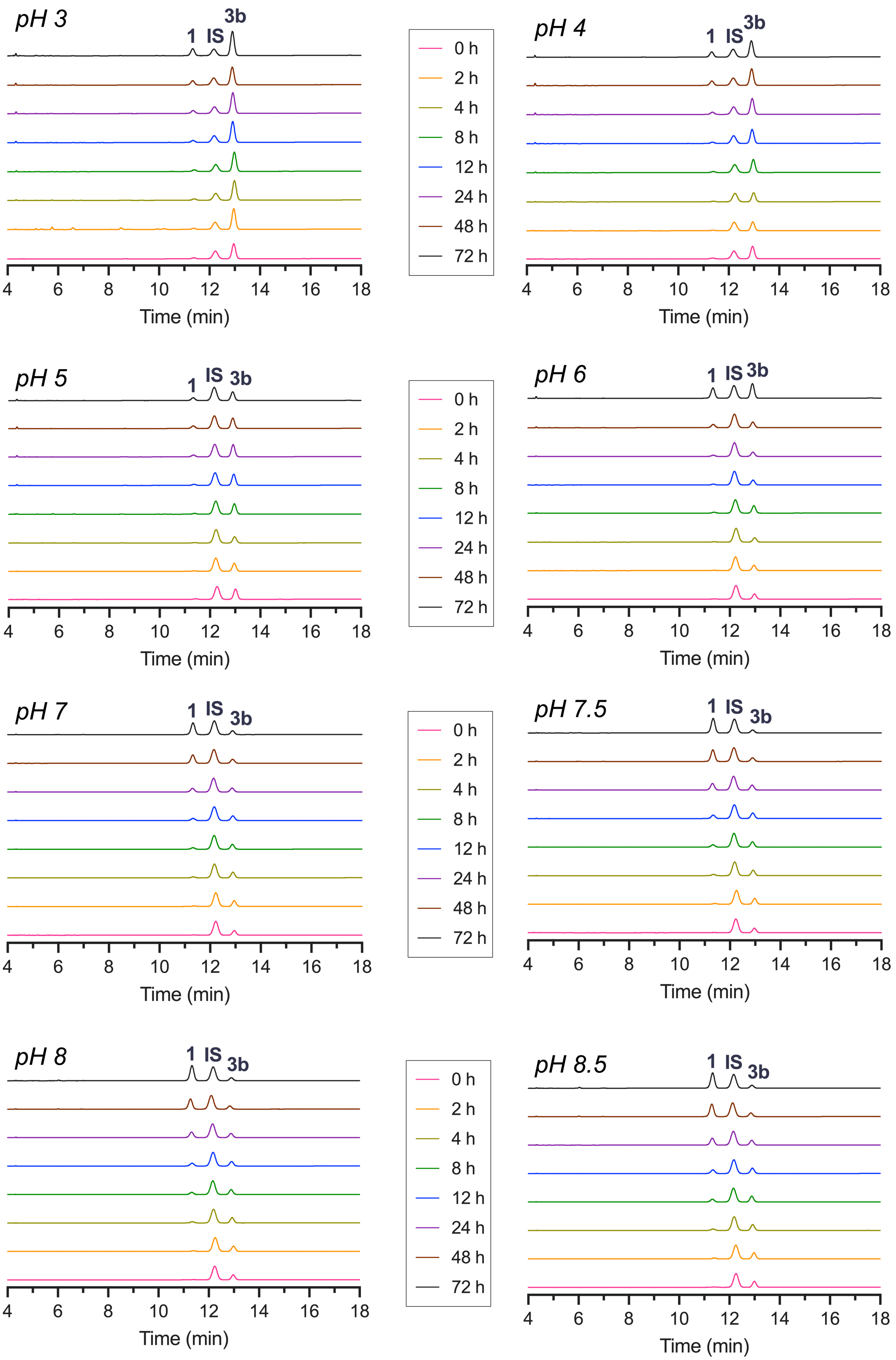
pH 3
1
IS
3b
4 6 8 10 12 14 16 18
Time (min)
0 h
2 h
4 h
8 h
12 h
24 h
48 h
72 h
pH 4
1
IS
3b
4 6 8 10 12 14 16 18
Time (min)
pH 5
1
IS
3b
4 6 8 10 12 14 16 18
Time (min)
0 h
2 h
4 h
8 h
12 h
24 h
48 h
72 h
pH 6
1
IS
3b
4 6 8 10 12 14 16 18
Time (min)
pH 7
1
IS
3b
4 6 8 10 12 14 16 18
Time (min)
0 h
2 h
4 h
8 h
12 h
24 h
48 h
72 h
pH 7.5
1
IS
3b
4 6 8 10 12 14 16 18
Time (min)
pH 8
1
IS
3b
4 6 8 10 12 14 16 18
Time (min)
0 h
2 h
4 h
8 h
12 h
24 h
48 h
72 h
pH 8.5
1
IS
3b
4 6 8 10 12 14 16 18
Time (min)

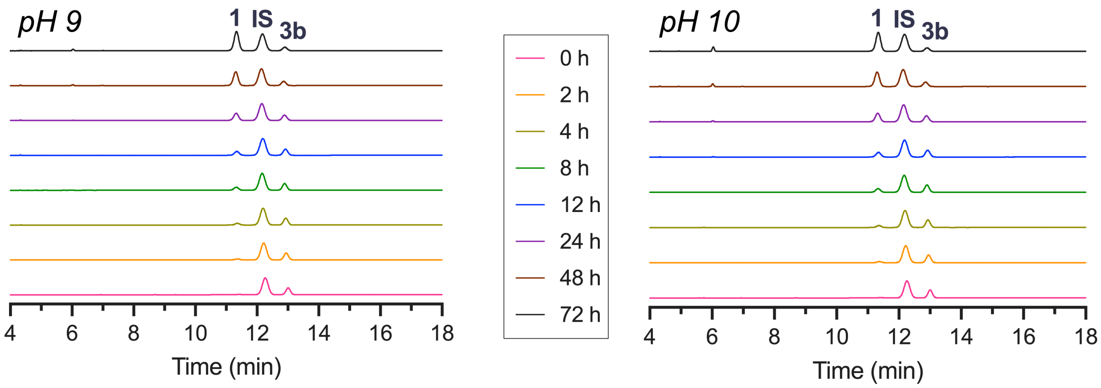

Figure S11. HPLC traces for 16b release experiments. The representative traces shown are one replicate of the triplicate experiments performed. RT **3b** = 12.9 min, RT **1** = 11.3 min, RT warfarin IS 12.2 = min.

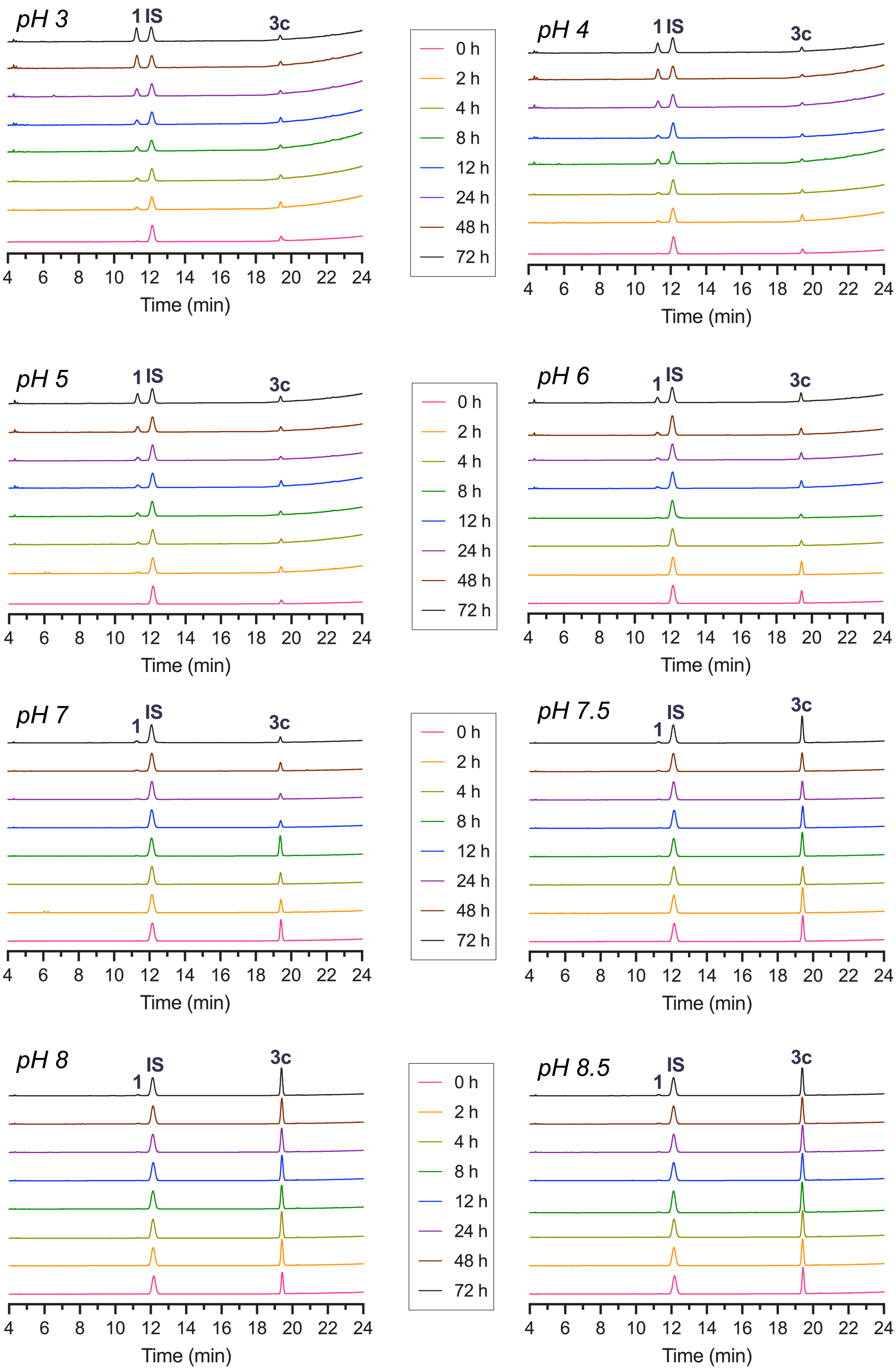
pH 3
1 IS
3c
4 6 8 10 12 14 16 18 20 22 24
Time (min)
0 h
2 h
4 h
8 h
12 h
24 h
48 h
72 h
pH 4
1 IS
3c
4 6 8 10 12 14 16 18 20 22 24
Time (min)
pH 5
1 IS
3c
4 6 8 10 12 14 16 18 20 22 24
Time (min)
0 h
2 h
4 h
8 h
12 h
24 h
48 h
72 h
pH 6
1 IS
3c
4 6 8 10 12 14 16 18 20 22 24
Time (min)
pH 7
1 IS
3c
4 6 8 10 12 14 16 18 20 22 24
Time (min)
0 h
2 h
4 h
8 h
12 h
24 h
48 h
72 h
pH 7.5
1 IS
3c
4 6 8 10 12 14 16 18 20 22 24
Time (min)
pH 8
1 IS
3c
4 6 8 10 12 14 16 18 20 22 24
Time (min)
0 h
2 h
4 h
8 h
12 h
24 h
48 h
72 h
pH 8.5
1 IS
3c
4 6 8 10 12 14 16 18 20 22 24
Time (min)

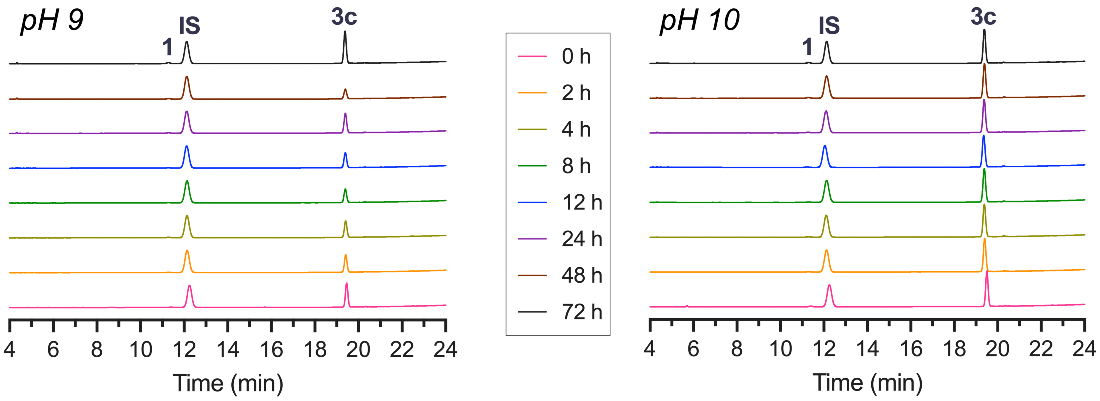

Figure S12. HPLC traces for 16c release experiments. The representative traces shown are one replicate of the triplicate experiments performed. RT **3c** = 19.4 min, RT **1** = 11.3 min, RT warfarin IS 12.2 = min.

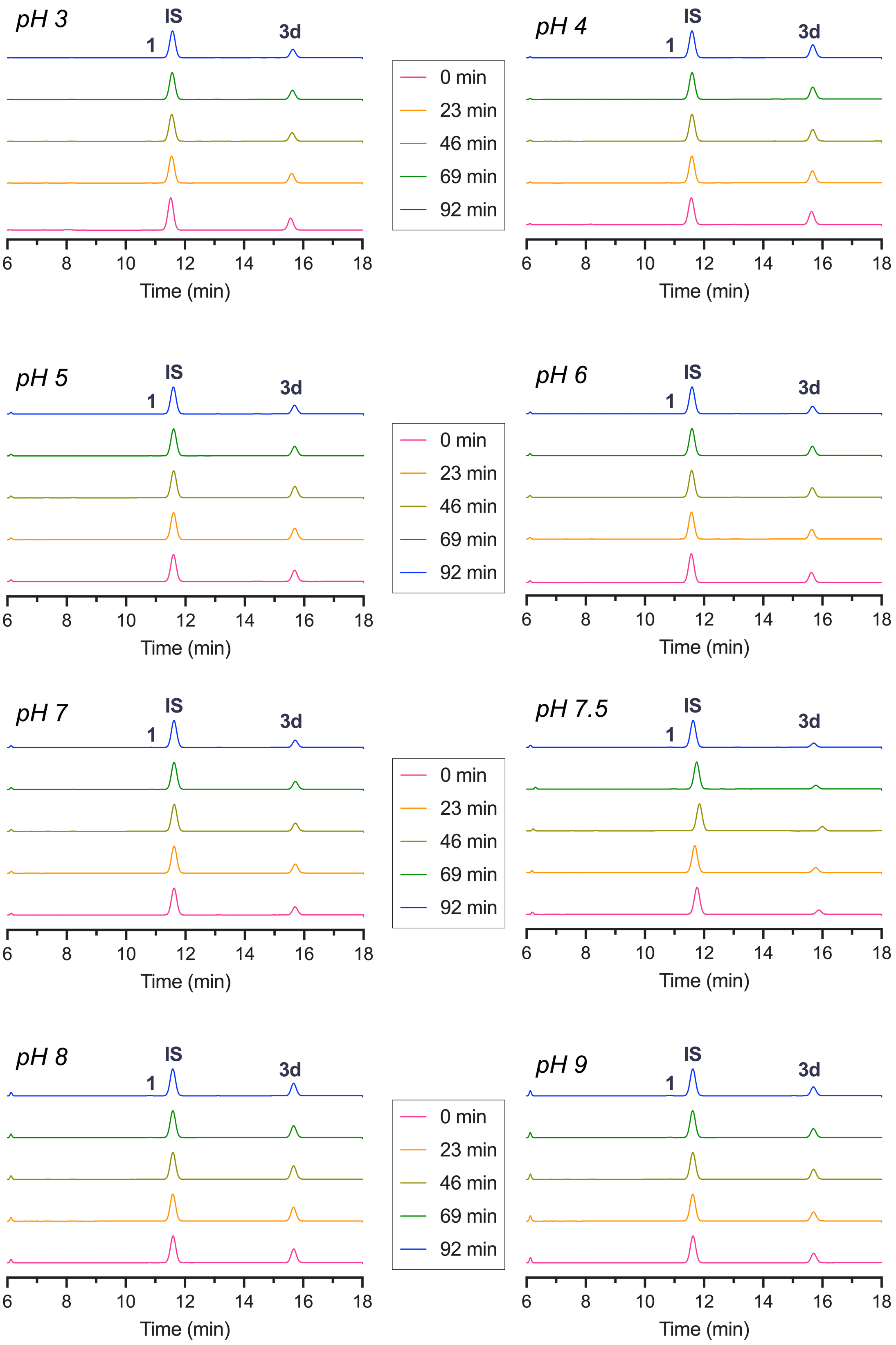
pH 3
IS
1
3d
6 8 10 12 14 16 18
Time (min)
0 min
23 min
46 min
69 min
92 min
pH 4
IS
1
3d
6 8 10 12 14 16 18
Time (min)
pH 5
IS
1
3d
6 8 10 12 14 16 18
Time (min)
0 min
23 min
46 min
69 min
92 min
pH 6
IS
1
3d
6 8 10 12 14 16 18
Time (min)
pH 7
IS
1
3d
6 8 10 12 14 16 18
Time (min)
0 min
23 min
46 min
69 min
92 min
pH 7.5
IS
1
3d
6 8 10 12 14 16 18
Time (min)
pH 8
IS
1
3d
6 8 10 12 14 16 18
Time (min)
0 min
23 min
46 min
69 min
92 min
pH 9
IS
1
3d
6 8 10 12 14 16 18
Time (min)

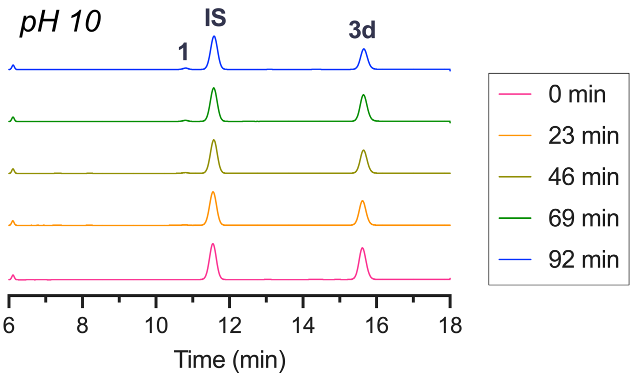

Figure S13. HPLC traces for 16d release experiments. The representative traces shown are one replicate of the triplicate experiments performed. RT **3d** = 15.8 min, RT **1** = 10.9 min, RT warfarin IS 11.7 = min.

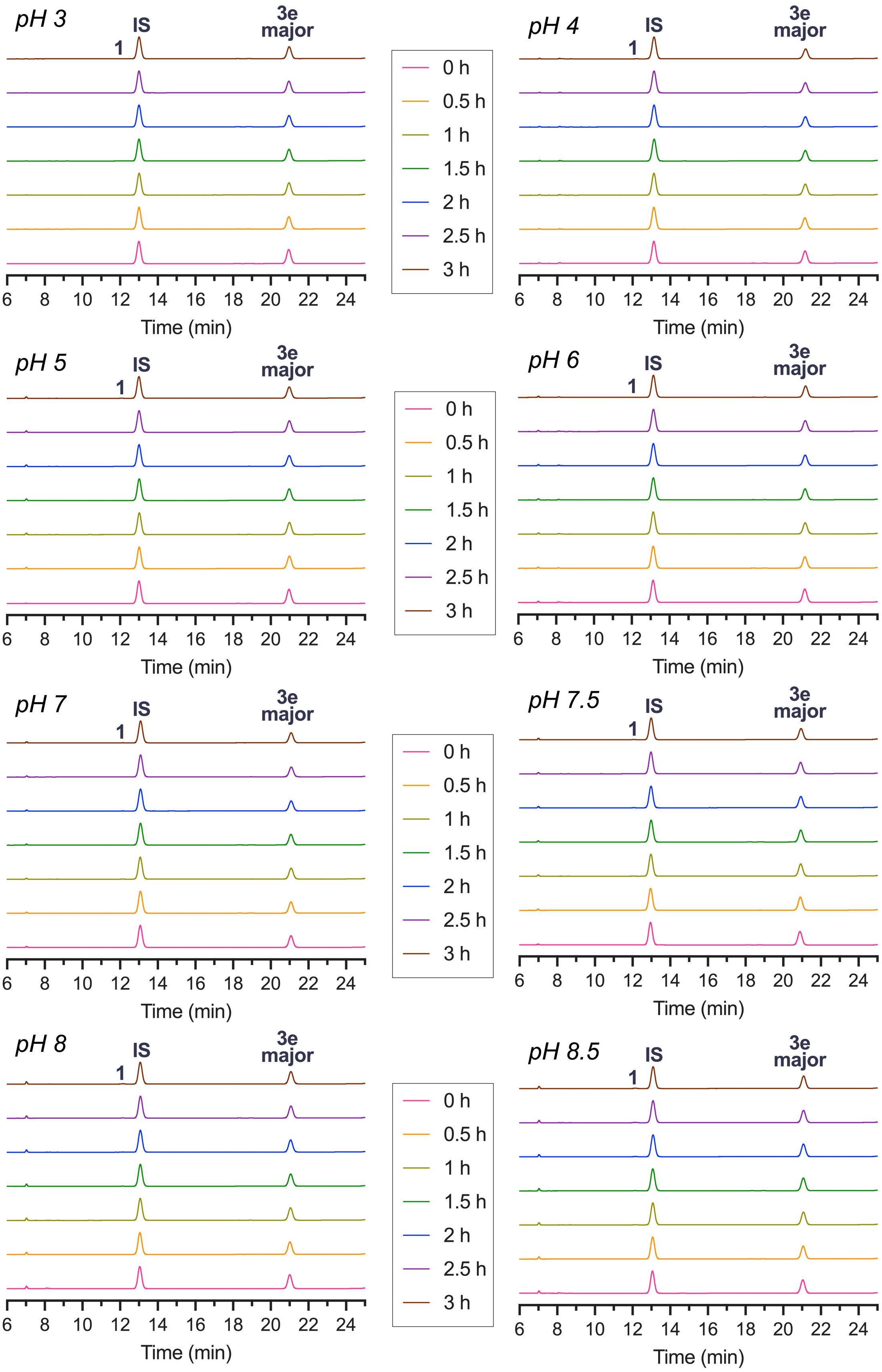
pH 3
pH 4
pH 5
pH 6
pH 7
pH 7.5
pH 8
pH 8.5
IS
1
3e
major
0 h
0.5 h
1 h
1.5 h
2 h
2.5 h
3 h
6
8
10
12
14
16
18
20
22
24
Time (min)

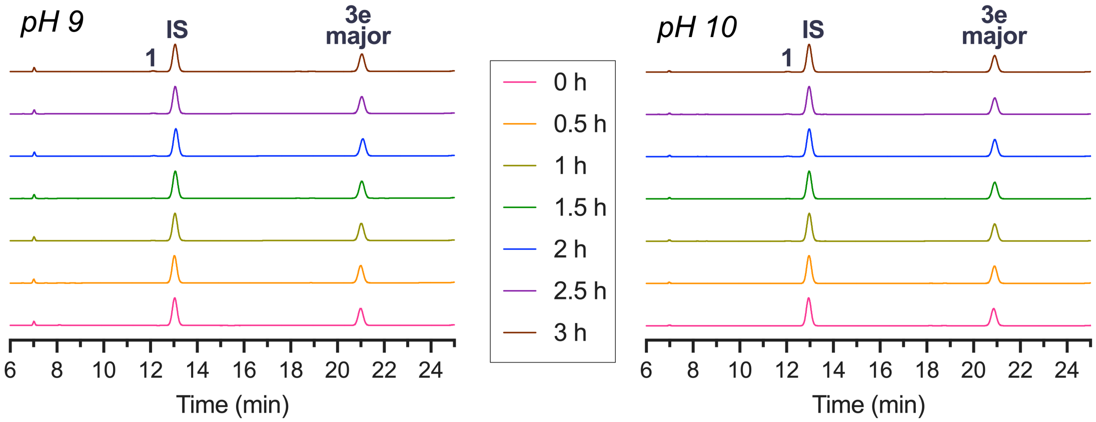

Figure S14. HPLC traces for 16e major product release experiments. The representative traces shown are one replicate of the triplicate experiments performed. RT **3e** = 20.8 min, RT **1** = 12.1 min, RT warfarin IS 12.9 = min.

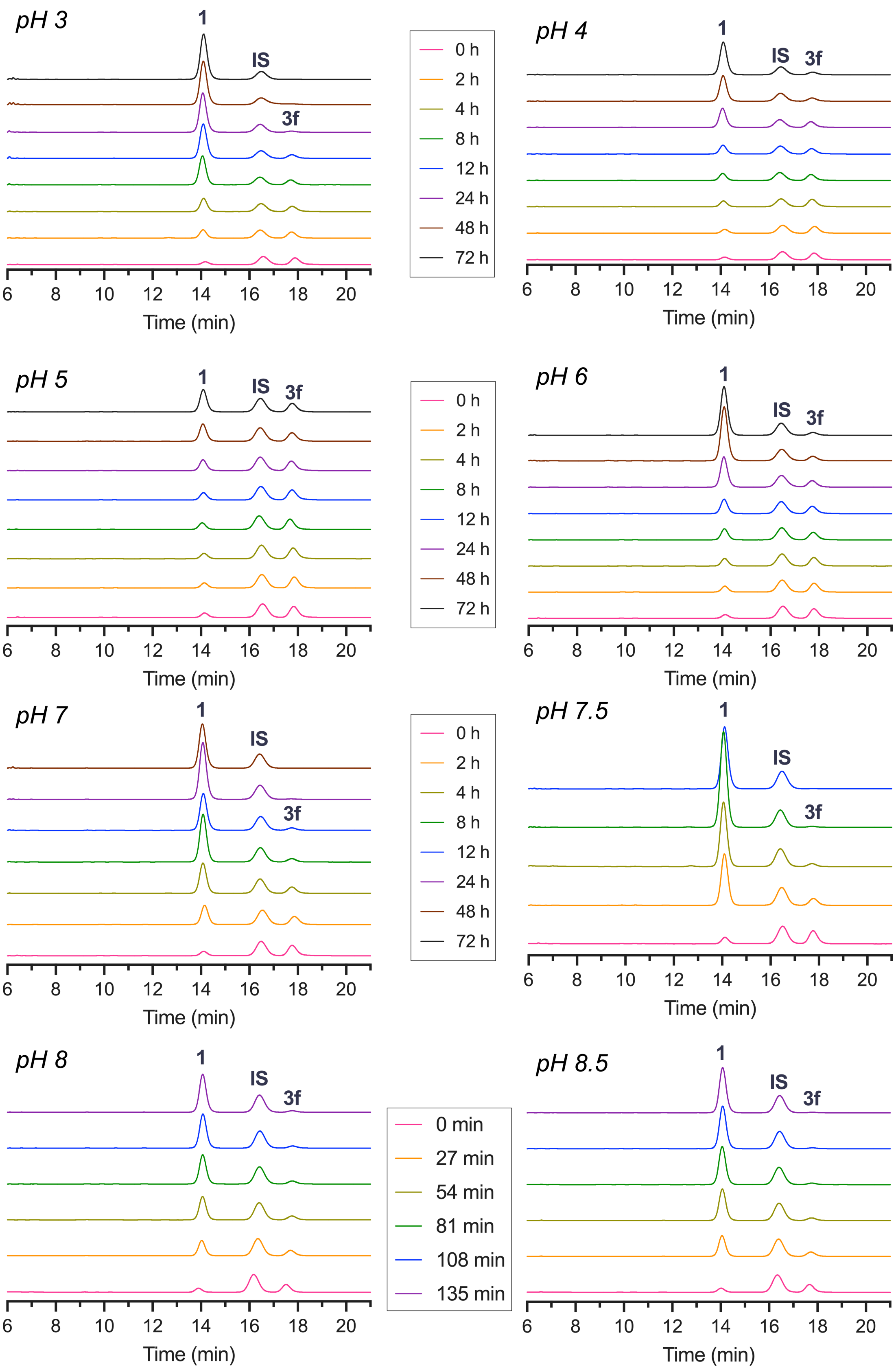

pH 3
1
IS
3f
0 h
2 h
4 h
8 h
12 h
24 h
48 h
72 h
pH 4
1
IS
3f
pH 5
1
IS
3f
pH 6
1
IS
3f
pH 7
1
IS
3f
pH 7.5
1
IS
3f
pH 8
1
IS
3f
0 min
27 min
54 min
81 min
108 min
135 min
pH 8.5
1
IS
3f
6 8 10 12 14 16 18 20
Time (min)

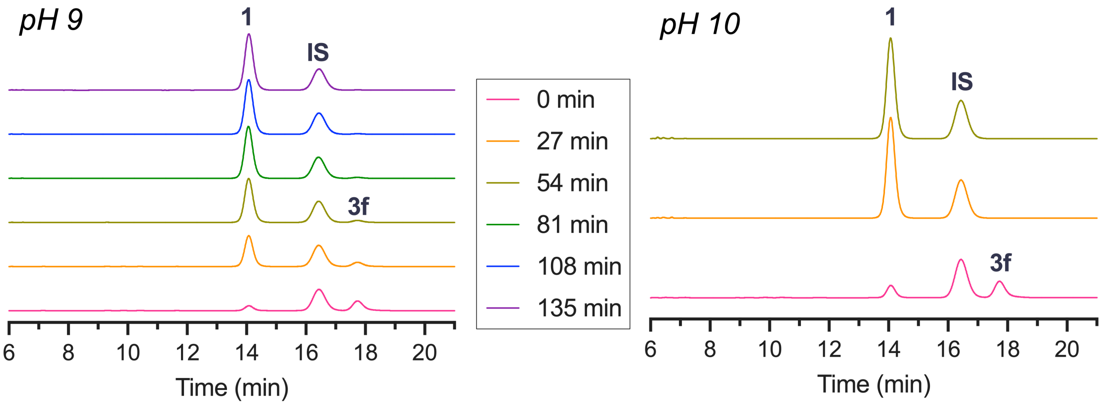

Figure S15. HPLC traces for 16f release experiments. The representative traces shown are one replicate of the triplicate experiments performed. RT **3f** = 17.8 min, RT **1** = 14.1 min, RT warfarin IS 16.5 = min.

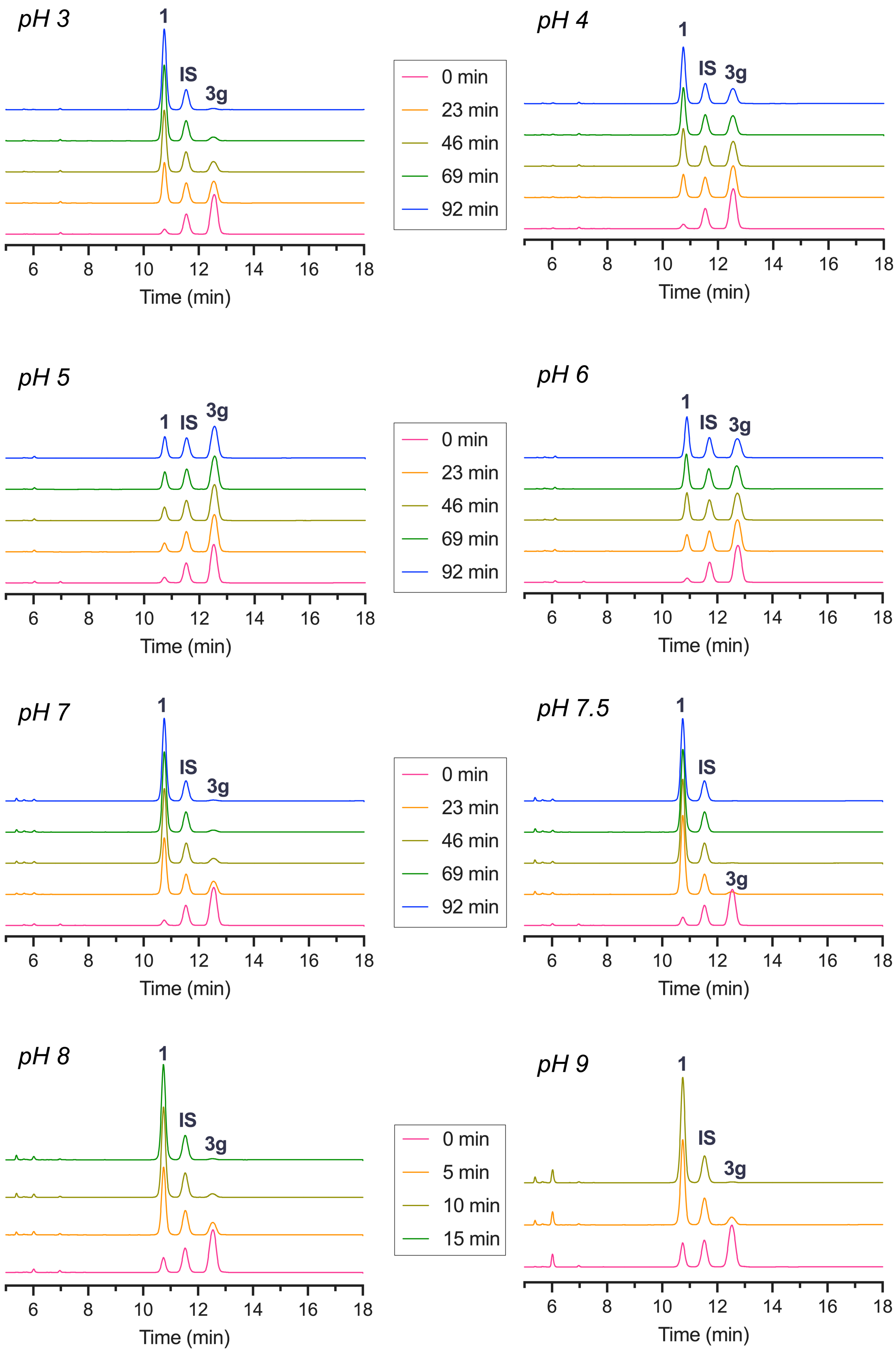
pH 3
1
IS
3g
6
8
10
12
14
16
18
Time (min)
0 min
23 min
46 min
69 min
92 min
pH 4
1
IS
3g
Time (min)
pH 5
1
IS
3g
Time (min)
0 min
23 min
46 min
69 min
92 min
pH 6
1
IS
3g
Time (min)
pH 7
1
IS
3g
Time (min)
0 min
23 min
46 min
69 min
92 min
pH 7.5
1
IS
3g
Time (min)
pH 8
1
IS
3g
Time (min)
0 min
5 min
10 min
15 min
pH 9
1
IS
3g
Time (min)

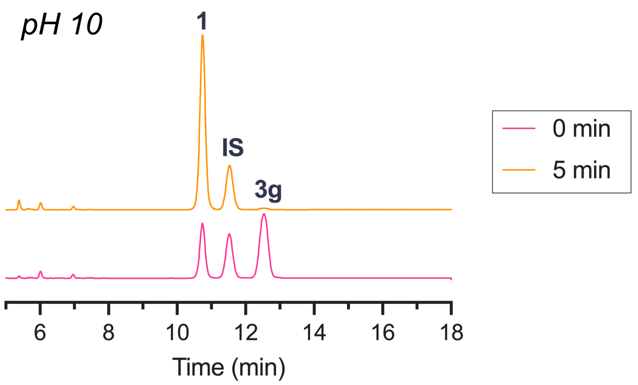

Figure S16. HPLC traces for 16g release experiments. The representative traces shown are one replicate of the triplicate experiments performed. RT **3g** = 12.5 min, RT **1** = 10.8 min, RT warfarin IS 11.6 = min.

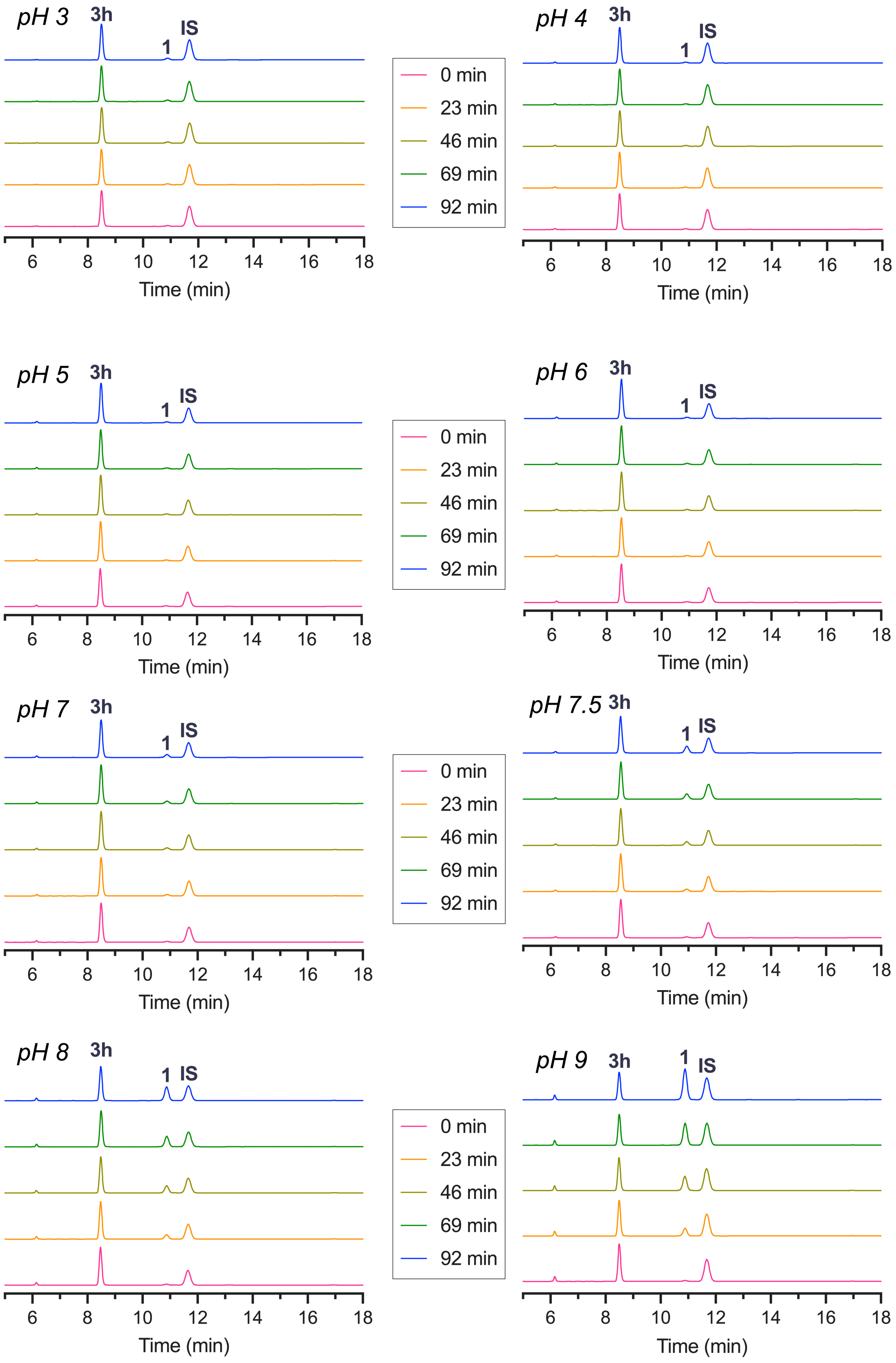
pH 3
3h
1
IS
6 8 10 12 14 16 18
Time (min)
0 min
23 min
46 min
69 min
92 min
pH 4
3h
1
IS
6 8 10 12 14 16 18
Time (min)
pH 5
3h
1
IS
6 8 10 12 14 16 18
Time (min)
0 min
23 min
46 min
69 min
92 min
pH 6
3h
1
IS
6 8 10 12 14 16 18
Time (min)
pH 7
3h
1
IS
6 8 10 12 14 16 18
Time (min)
0 min
23 min
46 min
69 min
92 min
pH 7.5
3h
1
IS
6 8 10 12 14 16 18
Time (min)
pH 8
3h
1
IS
6 8 10 12 14 16 18
Time (min)
0 min
23 min
46 min
69 min
92 min
pH 9
3h
1
IS
6 8 10 12 14 16 18
Time (min)

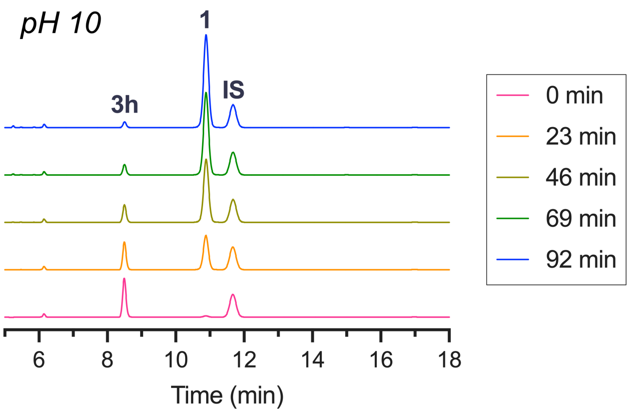

Figure S17. HPLC traces for 16h release experiments. The representative traces shown are one replicate of the triplicate experiments performed. RT **3h** = 8.5 min, RT **1** = 10.9 min, RT warfarin IS 11.7 = min.

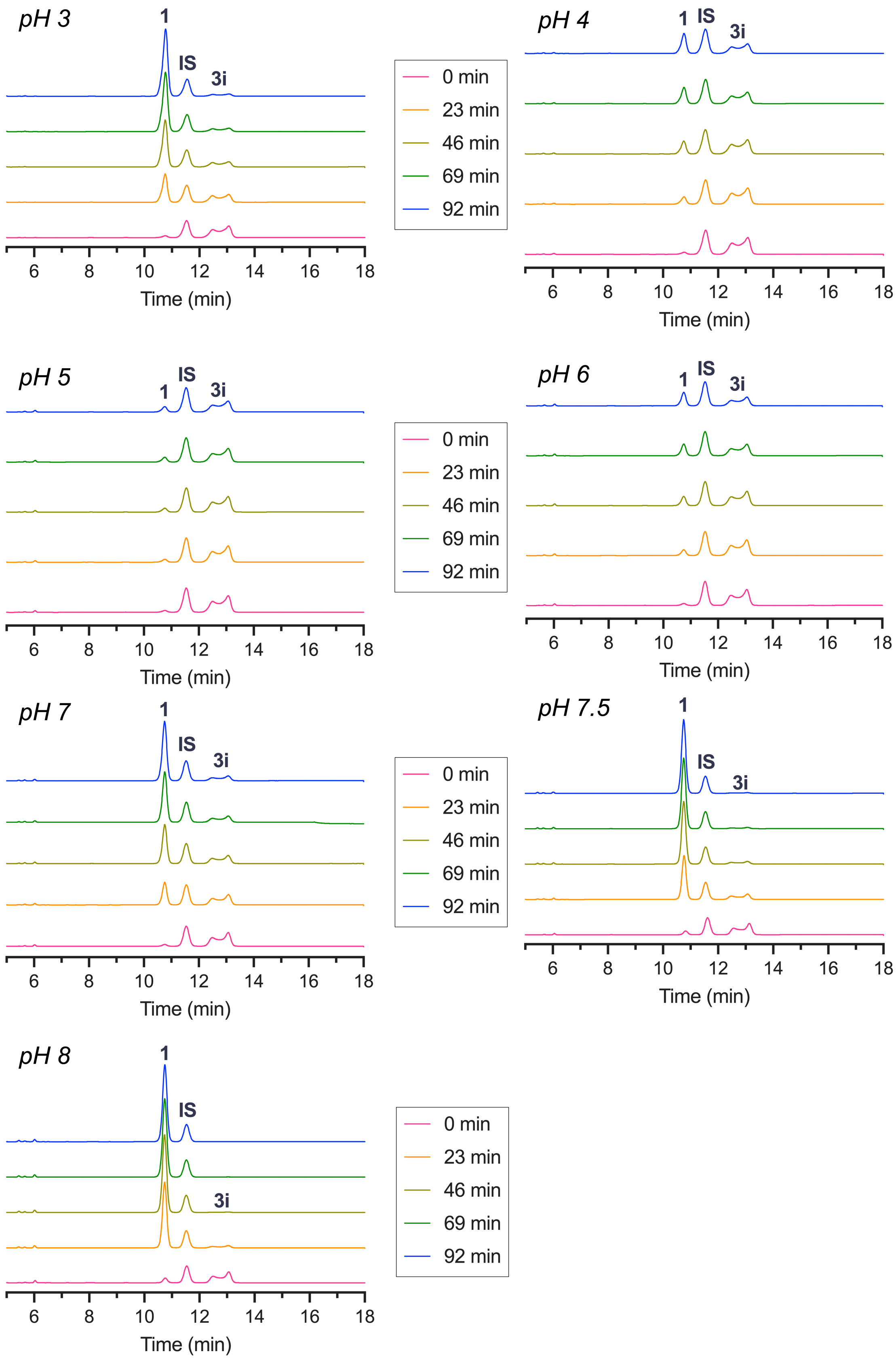
pH 3
1
IS
3i
pH 4
1
IS
3i
pH 5
IS
1
3i
pH 6
1
IS
3i
pH 7
1
IS
3i
pH 7.5
1
IS
3i
pH 8
1
IS
3i
0 min
23 min
46 min
69 min
92 min
6 8 10 12 14 16 18
Time (min)

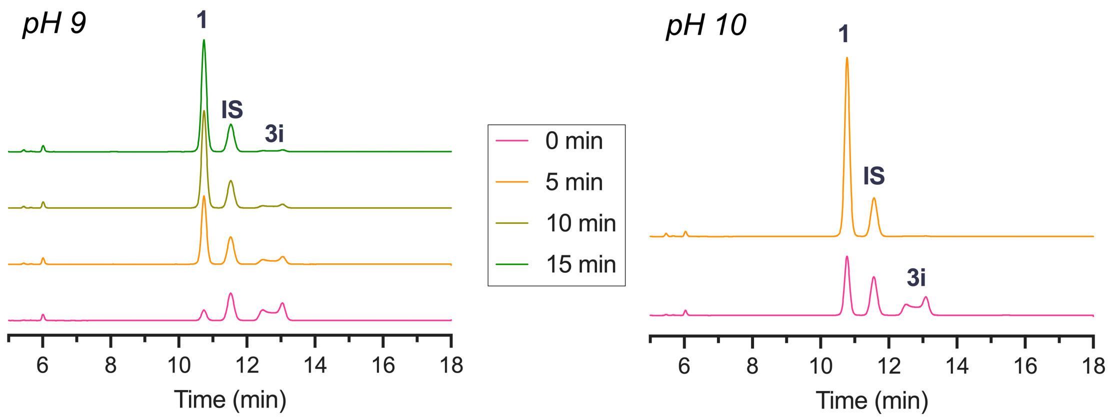

Figure S18. HPLC traces for 16i release experiments. The representative traces shown are one replicate of the triplicate experiments performed. RT **3i** = 12.5-13.0 min, RT **1** = 10.8 min, RT warfarin IS 11.6 = min.

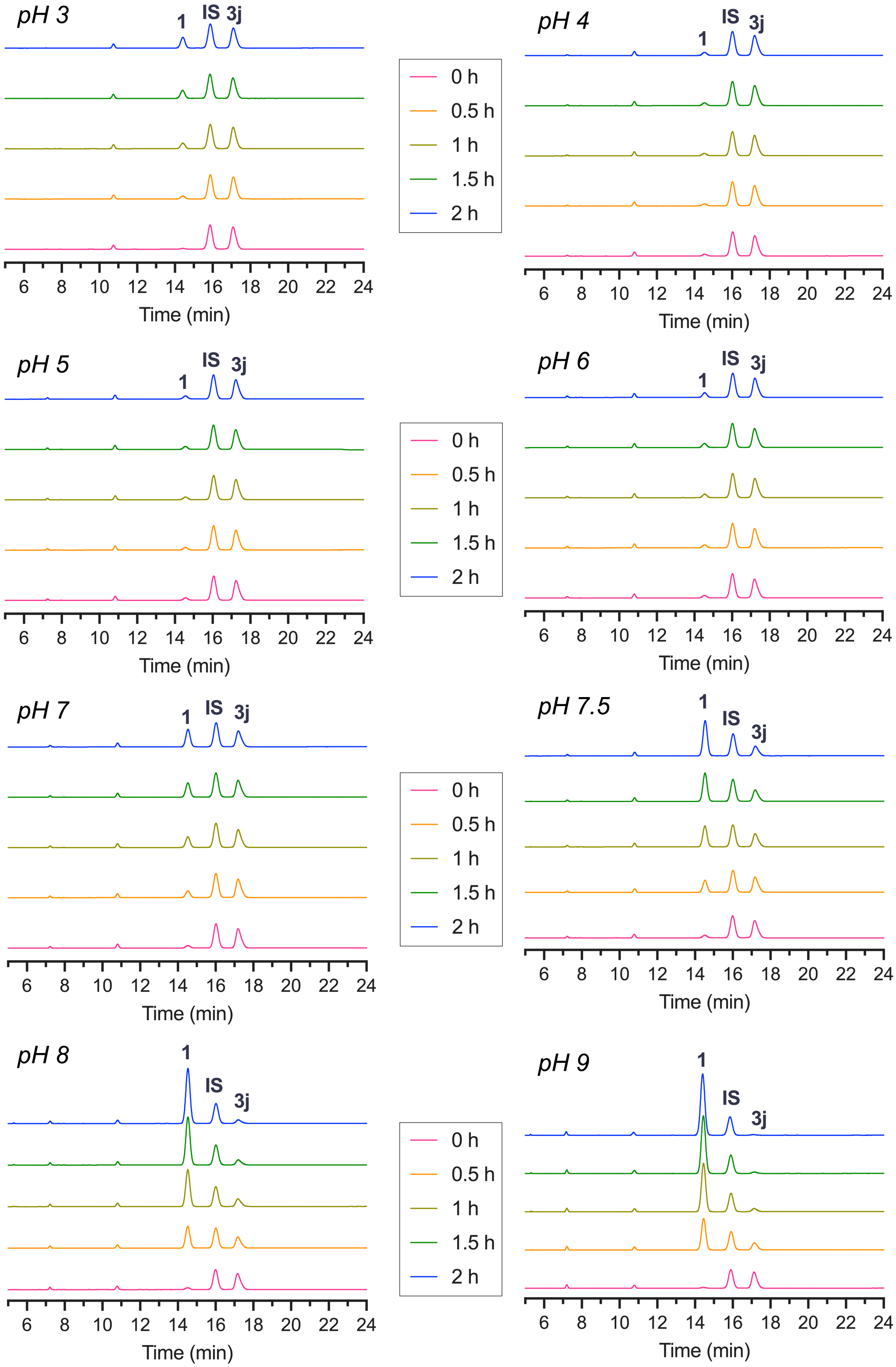
pH 3
1
IS
3j
0 h
0.5 h
1 h
1.5 h
2 h
6 8 10 12 14 16 18 20 22 24
Time (min)
pH 4
pH 5
pH 6
pH 7
pH 7.5
pH 8
pH 9

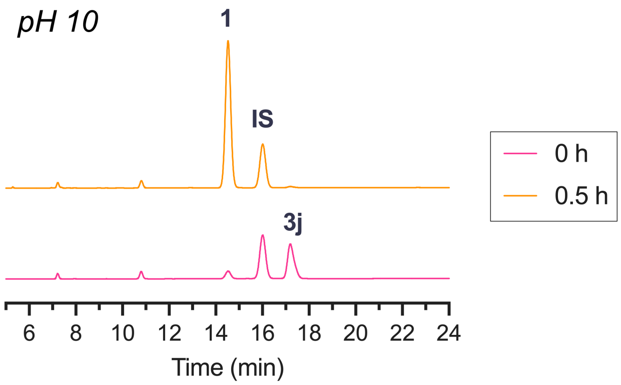

Figure S19. HPLC traces for 16j release experiments. The representative traces shown are one replicate of the triplicate experiments performed. RT **3j** = 17.2 min, RT **1** = 14.6 min, RT warfarin IS 16.1 = min.

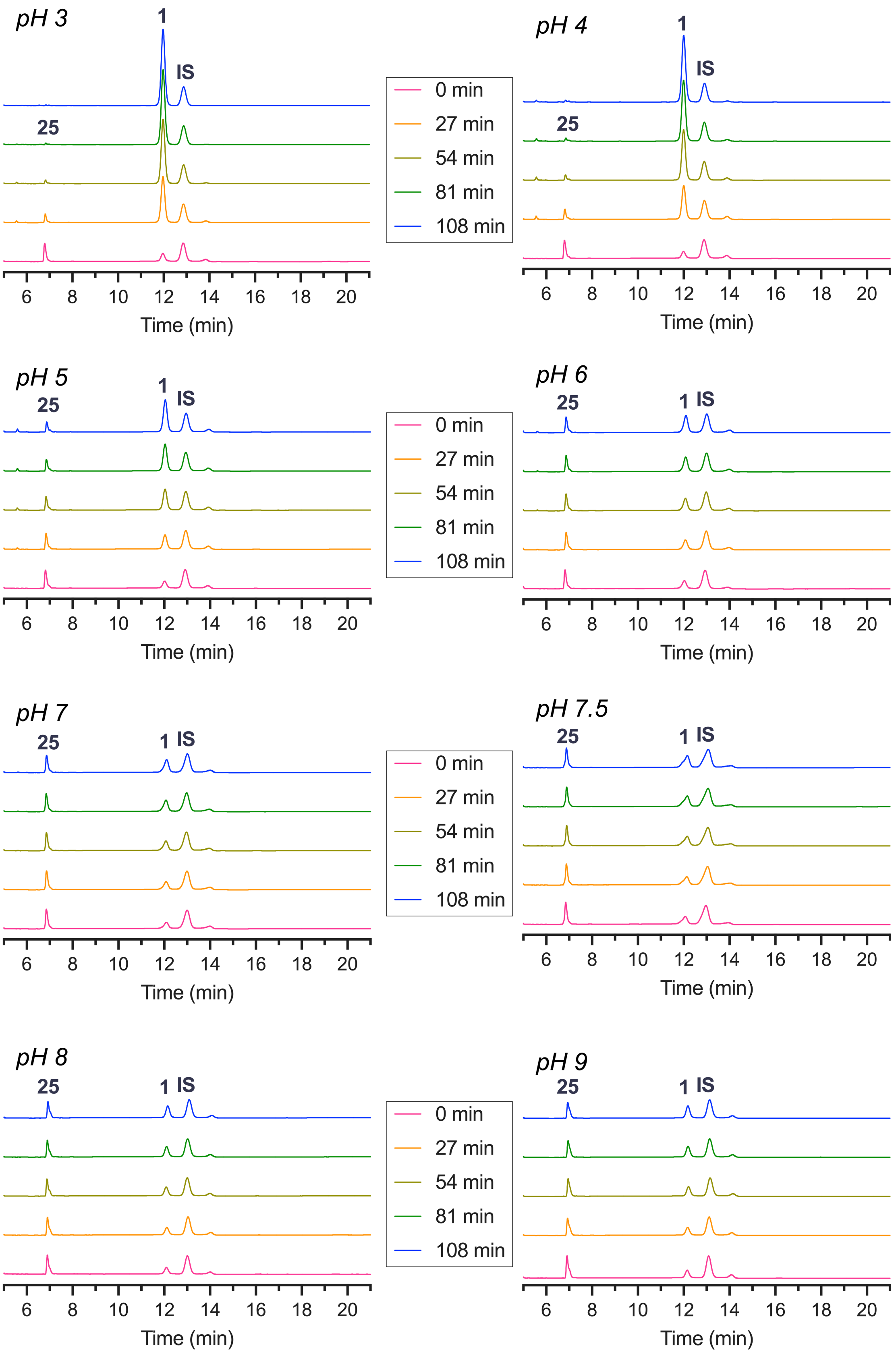
pH 3
1
IS
25
pH 4
1
IS
25
0 min
27 min
54 min
81 min
108 min
pH 5
25
1
IS
pH 6
25
1 IS
pH 7
25
1 IS
pH 7.5
25
1 IS
pH 8
25
1 IS
pH 9
25
1 IS
6 8 10 12 14 16 18 20
Time (min)

Figure S20. HPLC traces for 22 release experiments. The representative traces shown are one replicate of the triplicate experiments performed. RT **25** = 6.8 min, RT **1** = 12.0 min, RT warfarin IS 12.9 = min.

4.2. Linearized equations and fitting of kinetic data for each derivative.

$$\mathbf{3} \xrightarrow{k_{obs}} \mathbf{1}$$

$$A \rightarrow B$$

$$Rate = -\frac{d[A]}{dt} = k_{obs}[A]^1$$

$$\frac{d[A]}{[A]} = -k_{obs}\, dt$$

$$\int_{[A]_0}^{[A]_t} \frac{d[A]}{[A]} = -\int_{t_0}^{t} k_{obs} dt$$

$$\ln[A]_t - \ln[A]_0 = -k_{obs}t$$

$$\ln[A]_t = -k_{obs}t + \ln[A]_0$$

$$y = mx + b$$

Therefore, graph of $\ln[A]_t$ vs t is linear if first order and has the slope $-k_{obs}$

Putting the integrated rate law in terms of B:

$$[A]_t = [A]_0 - [B]_t$$

$$[A]_0 = [B]_\infty$$

$$[A]_t = [B]_\infty - [B]_t$$

$$\ln([B]_\infty - [B]_t) = -k_{obs}t + \ln[B]_\infty$$

Therefore, a graph of $\ln([B]_\infty - [B]_t)$ vs t is linear if first order and has the slope $-k_{obs}$

Figure S21. Derivation of the First Order Integrated Rate Laws in terms of [A] (PHB-β-Lapachone, 3) and [B] (β-lapachone, 1).

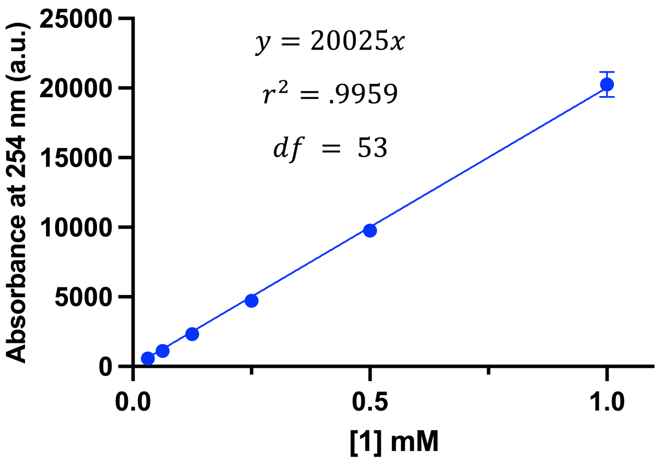

Figure S22. Lapachone Standard Curve. The standard curve for β-lapachone on the analytical Agilent 1260 HPLC is shown. Three separate serial dilutions were performed to give six different concentrations. DMSO was used as the solvent since **1** has low solubility in water. Each sample was then injected three times using three different gradients for a total of 54 injections. The line of best fit was then calculated using GraphPad Prism 10 software. Error bars represent ± SD.

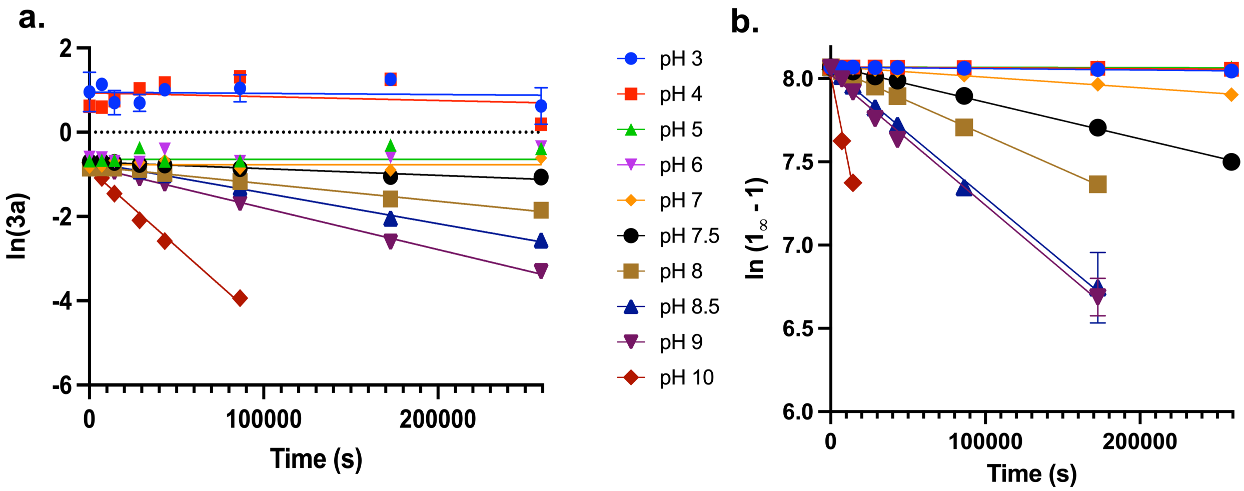

Figure S23. First order linearized kinetics graphs for the release of 1 from 16a. (a) The ln of the normalized peak area of **3a** vs time is plotted. Error bars represent ± SD for the triplicate experiments. The linear relationship confirms the drug release kinetics are first order. The slopes of this graph were used to calculate k_{obs} for pH 8-10. From pH 3-7.5, very little change in the peak area of **3a** was detected over the assay period, so determining the release kinetics using this graph was less accurate. (b) Instead, the graph of ln (**1**$_{\infty}$**-1**) vs time was plotted. The absorbance of **1** was the peak area of **1** at the timepoint of interest, and the theoretical absorbance of **1**$_{\infty}$ was calculated based on the known value of [**3a**] at t = 0 and the lapachone standard curve. Error bars represent ± SD for the triplicate experiments. The slopes of this graph were used to calculate k_{obs} for pH 3-7.5. All slopes were calculated using GraphPad Prism 10.

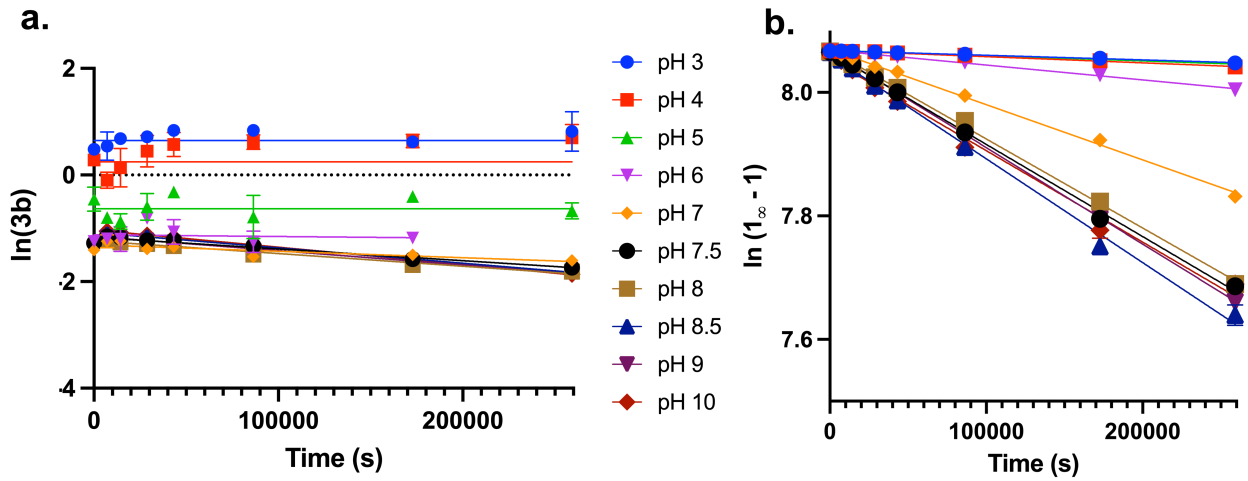

Figure S24. First order linearized kinetics graphs for the release of 1 from 16b. (a) The ln of the normalized peak area of **3b** vs time is plotted. Error bars represent ± SD for the triplicate experiments. The linear relationship confirms the drug release kinetics are first order. The slopes of this graph were used to calculate k_{obs} for pH 8-10. From pH 3-7.5, very little change in the peak area of **3b** was detected over the assay period, so determining the release kinetics using this graph was less accurate. (b) Instead, the graph of ln (**1**$_\infty$-**1**) vs time was plotted. The absorbance of **1** was the peak area of **1** at the timepoint of interest, and the theoretical absorbance of **1**$_\infty$ was calculated based on the known value of [**3b**] at t = 0 and the lapachone standard curve. Error bars represent ± SD for the triplicate experiments. The slopes of this graph were used to calculate k_{obs} for pH 3-7.5. All slopes were calculated using GraphPad Prism 10.

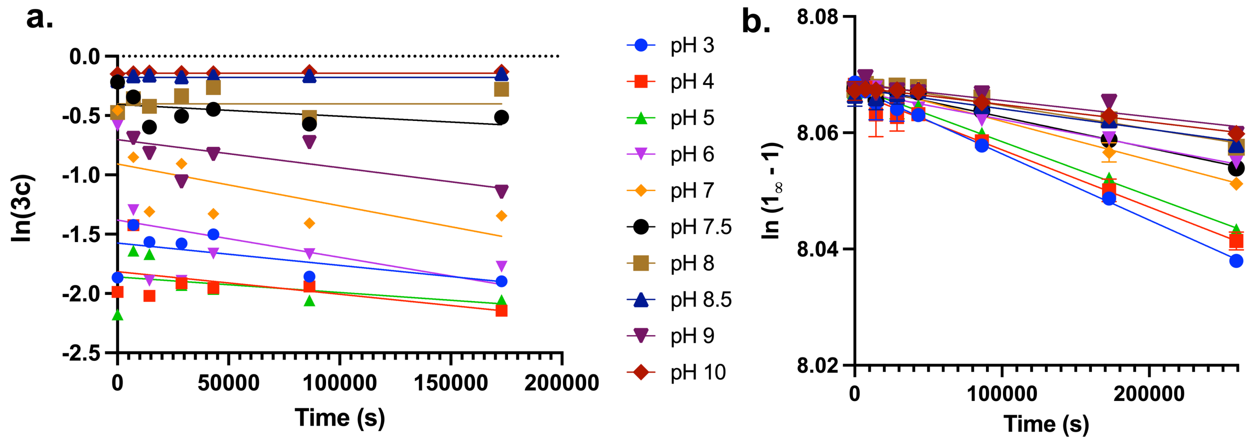

Figure S25. First order linearized kinetics graphs for the release of 1 from 16c. (a) The ln of the normalized peak area of **3c** vs time is plotted. Error bars represent ± SD for the triplicate experiments. From pH 3-10, very little change in the peak area of **3c** was detected over the assay period, so determining the release kinetics using this graph was less accurate. (b) Instead, the graph of ln (**1**$_\infty$-**1**) vs time was plotted. The absorbance of **1** was the peak area of **1** at the timepoint of interest, and the theoretical absorbance of **1**$_\infty$ was calculated based on the known value of [**3c**] at t = 0 and the lapachone standard curve. Error bars represent ± SD for the

triplicate experiments. The linear relationship confirms the drug release kinetics are first order. The slopes of this graph were used to calculate k_{obs} for pH 3-10. All slopes were calculated using GraphPad Prism 10.

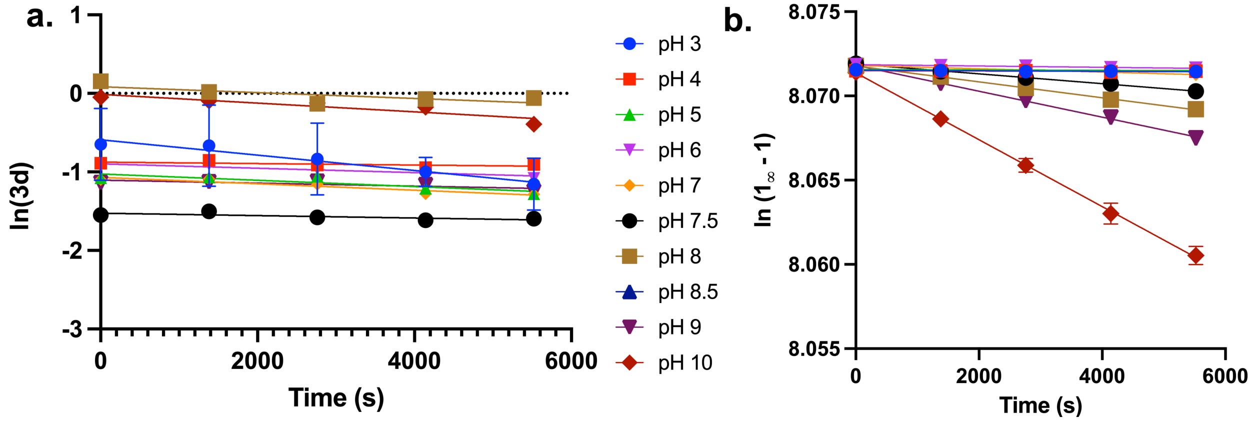

Figure S26. First order linearized kinetics graphs for the release of 1 from 16d. (a) The ln of the normalized peak area of **3d** vs time is plotted. Error bars represent ± SD for the triplicate experiments. From pH 3-10, very little change in the peak area of **3d** was detected over the assay period, so determining the release kinetics using this graph was less accurate. (b) Instead, the graph of ln (**1_∞-1**) vs time was plotted. The absorbance of **1** was the peak area of **1** at the timepoint of interest, and the theoretical absorbance of **1_∞** was calculated based on the known value of [**3d**] at t = 0 and the lapachone standard curve. Error bars represent ± SD for the triplicate experiments. The linear relationship confirms the drug release kinetics are first order. The slopes of this graph were used to calculate k_{obs} for pH 3-10. All slopes were calculated using GraphPad Prism 10.

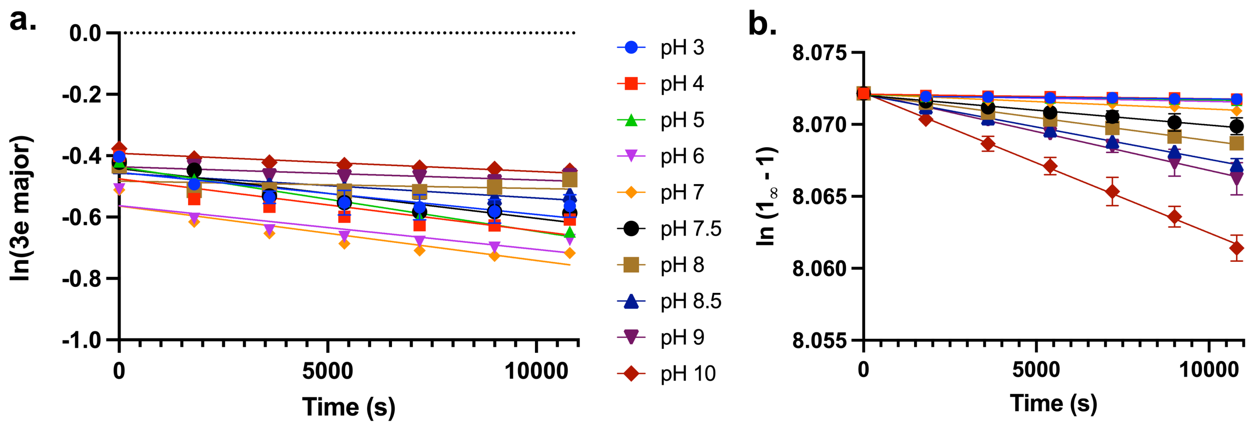

Figure S27. First order linearized kinetics graphs for the release of 1 from the 16e major product. (a) The ln of the normalized peak area of **3e** vs time is plotted. Error bars represent ± SD for the triplicate experiments. From pH 3-10, very little change in the peak area of **3e** was detected over the assay period, so determining the release kinetics using this graph was less accurate. (b) Instead, the graph of ln (**1_∞-1**) vs time was plotted. The absorbance of **1** was the

peak area of **1** at the timepoint of interest, and the theoretical absorbance of $\mathbf{1}_{\infty}$ was calculated based on the known value of [**3e**] at t = 0 and the lapachone standard curve. Error bars represent ± SD for the triplicate experiments. The linear relationship confirms the drug release kinetics are first order. The slopes of this graph were used to calculate k_{obs} for pH 3-10. All slopes were calculated using GraphPad Prism 10.

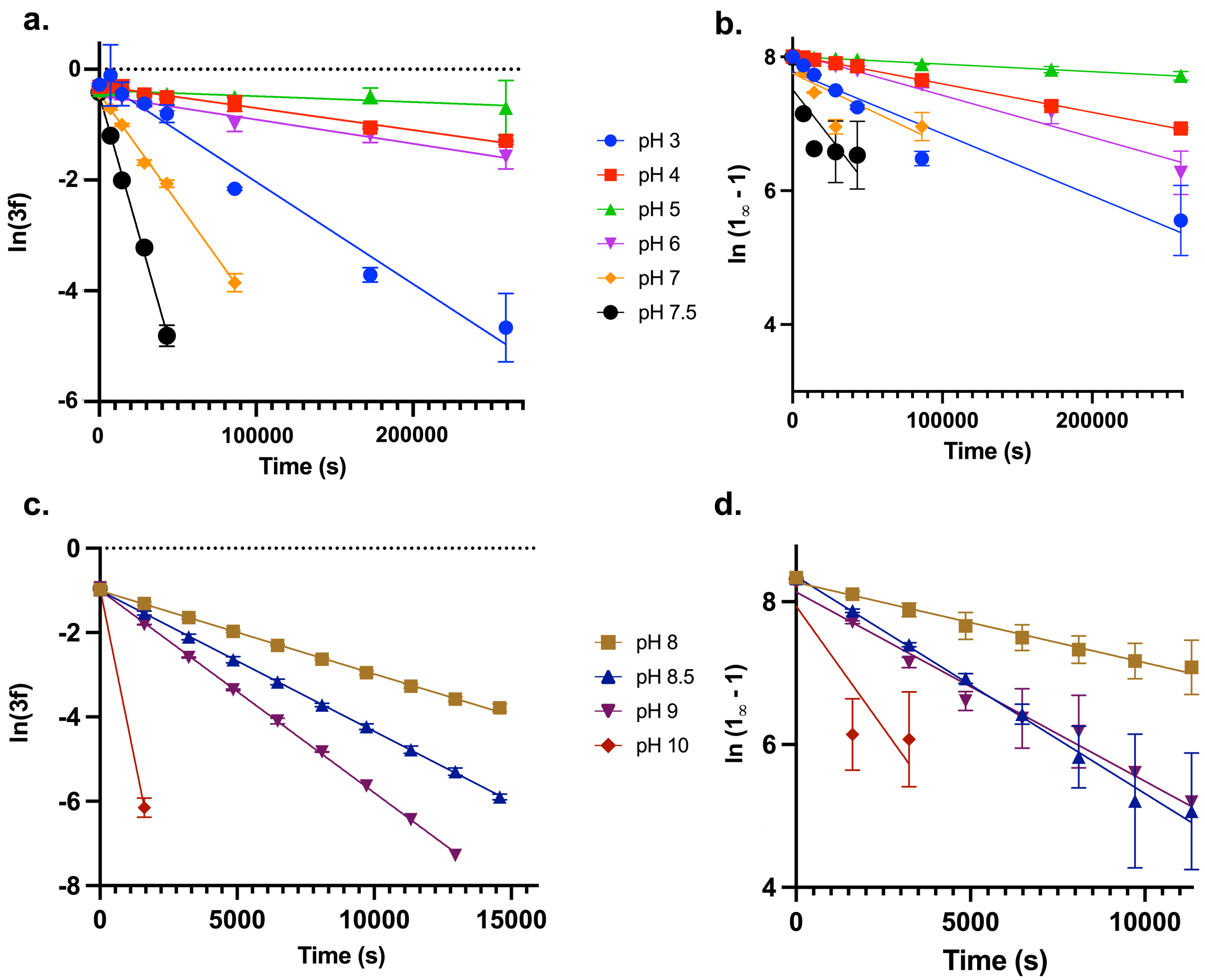

Figure S28. First order linearized kinetics graphs for the release of 1 from 16f. (a,c) The ln of the normalized peak area of **3f** vs time is plotted. Error bars represent ± SD for the triplicate experiments. The linear relationship confirms the drug release kinetics are first order. The slopes of this graph were used to calculate k_{obs} for pH 3 and 7-10. From pH 4-6, very little change in the peak area of **3f** was detected over the assay period, so determining the release kinetics using this graph was less accurate. (b,d) Instead, the graph of ln ($\mathbf{1}_{\infty}$**-1**) vs time was plotted. The absorbance of **1** was the peak area of **1** at the timepoint of interest, and the theoretical absorbance of $\mathbf{1}_{\infty}$ was calculated based on the known value of [**3f**] at t = 0 and the lapachone standard curve. Error bars represent ± SD for the triplicate experiments. The slopes of this graph were used to calculate k_{obs} for pH 4-6. All slopes were calculated using GraphPad Prism 10.

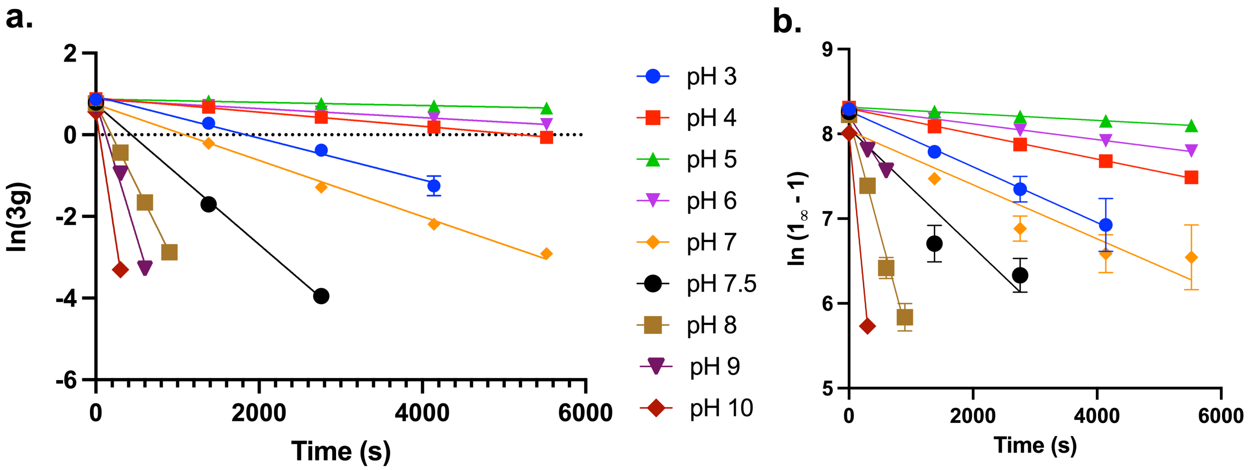

Figure S29. First order linearized kinetics graphs for the release of 1 from 16g. (a) The ln of the normalized peak area of **3g** vs time is plotted. Error bars represent ± SD for the triplicate experiments. The linear relationship confirms the drug release kinetics are first order. The slopes of this graph were used to calculate k_{obs} for pH 3 and 7-10. From pH 4-6, very little change in the peak area of **3g** was detected over the assay period, so determining the release kinetics using this graph was less accurate. (b) Instead, the graph of ln (**1_∞-1**) vs time was plotted. The absorbance of **1** was the peak area of **1** at the timepoint of interest, and the theoretical absorbance of **1_∞** was calculated based on the known value of [**3g**] at t = 0 and the lapachone standard curve. Error bars represent ± SD for the triplicate experiments. The slopes of this graph were used to calculate k_{obs} for pH 4-6. All slopes were calculated using GraphPad Prism 10.

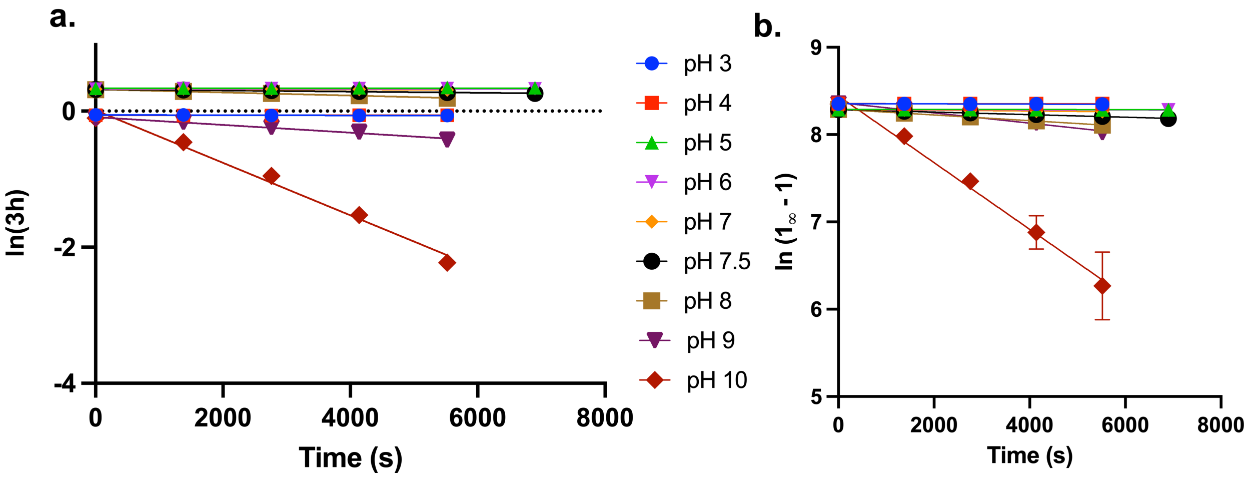

Figure S30. First order linearized kinetics graphs for the release of 1 from 16h. (a) The ln of the normalized peak area of **3h** vs time is plotted. Error bars represent ± SD for the triplicate experiments. The linear relationship confirms the drug release kinetics are first order. The slopes of this graph were used to calculate k_{obs} for pH 8-10. From pH 3-7.5, very little change in the peak area of **3h** was detected over the assay period, so determining the release kinetics using this graph was less accurate. (b) Instead, the graph of ln (**1_∞-1**) vs time was plotted. The absorbance

of **1** was the peak area of **1** at the timepoint of interest, and the theoretical absorbance of $\mathbf{1}_\infty$ was calculated based on the known value of [**3h**] at t = 0 and the lapachone standard curve. Error bars represent ± SD for the triplicate experiments. The slopes of this graph were used to calculate k_{obs} for pH 3-7.5. All slopes were calculated using GraphPad Prism 10.

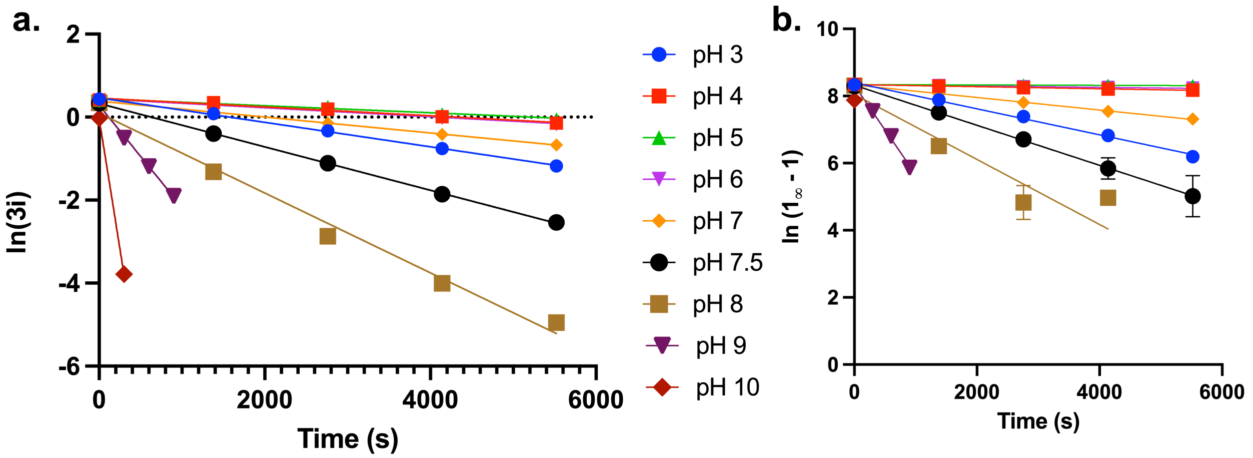

Figure S31. First order linearized kinetics graphs for the release of 1 from 16i. (a) The ln of the normalized peak area of **3i** vs time is plotted. Error bars represent ± SD for the triplicate experiments. The linear relationship confirms the drug release kinetics are first order. The slopes of this graph were used to calculate k_{obs} for pH 3 and 7-10. From pH 4-6, very little change in the peak area of **3i** was detected over the assay period, so determining the release kinetics using this graph was less accurate. (b) Instead, the graph of ln ($\mathbf{1}_\infty$**-1**) vs time was plotted. The absorbance of **1** was the peak area of **1** at the timepoint of interest, and the theoretical absorbance of $\mathbf{1}_\infty$ was calculated based on the known value of [**3i**] at t = 0 and the lapachone standard curve. Error bars represent ± SD for the triplicate experiments. The slopes of this graph were used to calculate k_{obs} for pH 4-6. All slopes were calculated using GraphPad Prism 10.

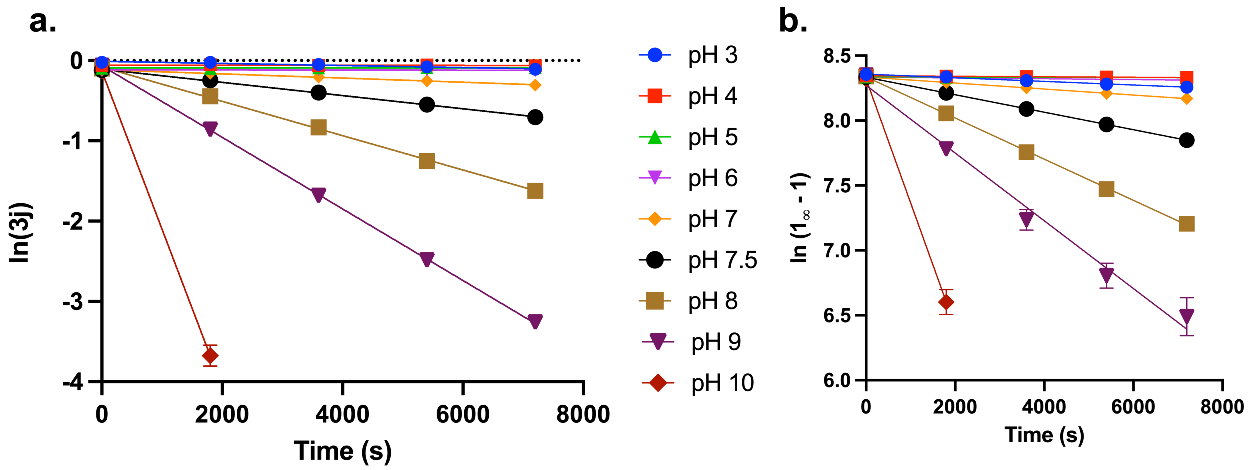

Figure S32. First order linearized kinetics graphs for the release of 1 from 16j. (a) The ln of the normalized peak area of **3j** vs time is plotted. Error bars represent ± SD for the triplicate

experiments. The linear relationship confirms the drug release kinetics are first order. The slopes of this graph were used to calculate k_{obs} for pH 7-10. From pH 3-6, very little change in the peak area of **3j** was detected over the assay period, so determining the release kinetics using this graph was less accurate. (b) Instead, the graph of ln (**1**$_\infty$**-1**) vs time was plotted. The absorbance of **1** was the peak area of **1** at the timepoint of interest, and the theoretical absorbance of **1**$_\infty$ was calculated based on the known value of [**3j**] at t = 0 and the lapachone standard curve. Error bars represent ± SD for the triplicate experiments. The slopes of this graph were used to calculate k_{obs} for pH 3-6. All slopes were calculated using GraphPad Prism 10.

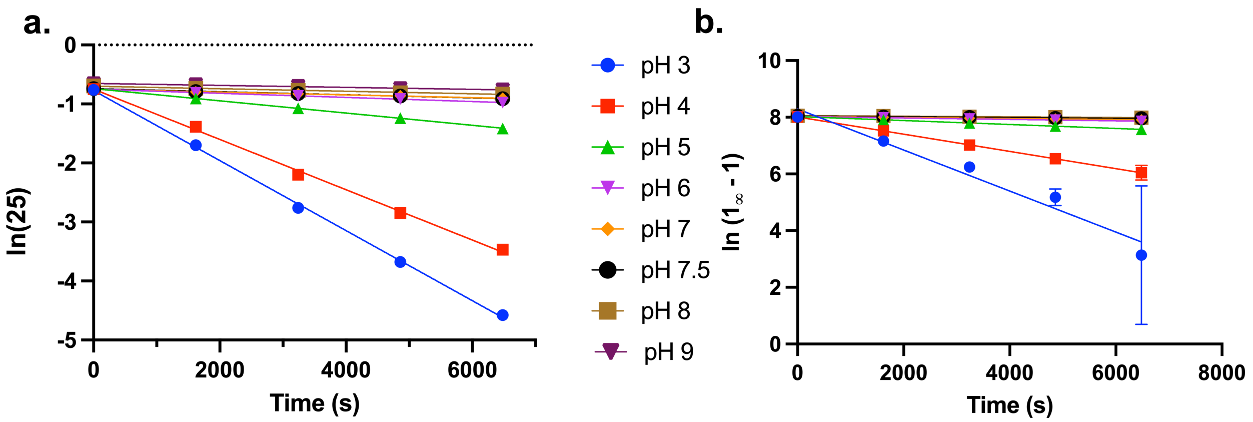

Figure S33. First order linearized kinetics graphs for the release of 1 from 22. (a) The ln of the normalized peak area of **25** vs time is plotted. Error bars represent ± SD for the triplicate experiments. The linear relationship confirms the drug release kinetics are first order. The slopes of this graph were used to calculate k_{obs} for pH 3-6. From pH 7-9, very little change in the peak area of **25** was detected over the assay period, so determining the release kinetics using this graph was less accurate. (b) Instead, the graph of ln (**1**$_\infty$**-1**) vs time was plotted. The absorbance of **1** was the peak area of **1** at the timepoint of interest, and the theoretical absorbance of **1**$_\infty$ was calculated based on the known value of [**25**] at t = 0 and the lapachone standard curve. Error bars represent ± SD for the triplicate experiments. The slopes of this graph were used to calculate k_{obs} for pH 7-9. All slopes were calculated using GraphPad Prism 10.

4.3. Retention time comparison to known standards for peak identification.

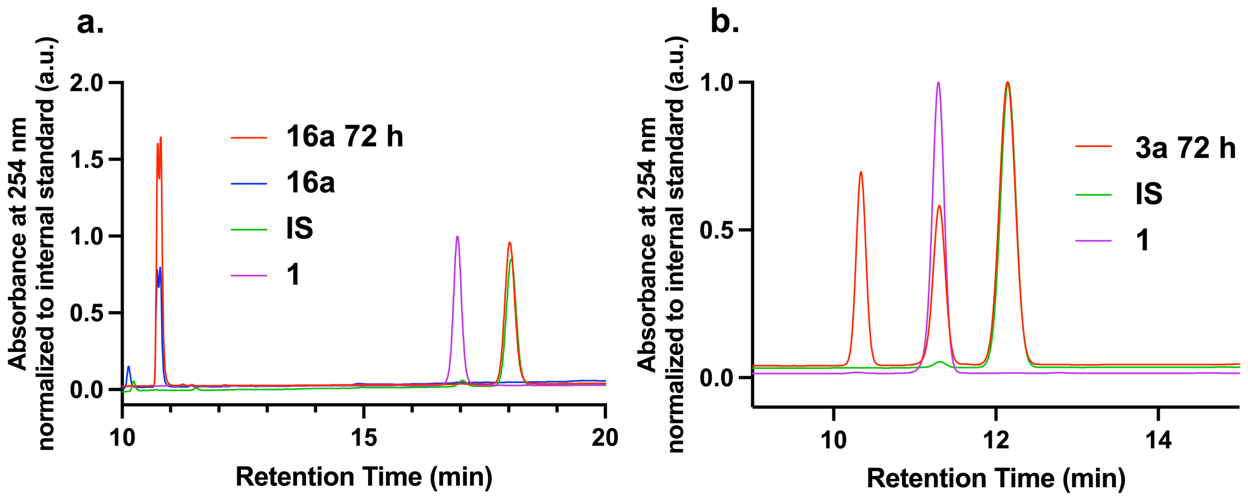

Figure S34. Peak Assignment for Stability and Release Assays for 16a. (a) A representative HPLC trace from the stability assay for **16a** after incubation for 72 h at 37°C in PBS buffer (red) is shown overlaid with the trace for **16a** (blue), **1** (purple), and the warfarin IS (green). (b) A representative HPLC trace from the release assay for **3a** after incubation for 72 h at 37°C in pH 7 buffer (red) is shown overlaid with the trace for **1** (purple) and the warfarin IS (green).

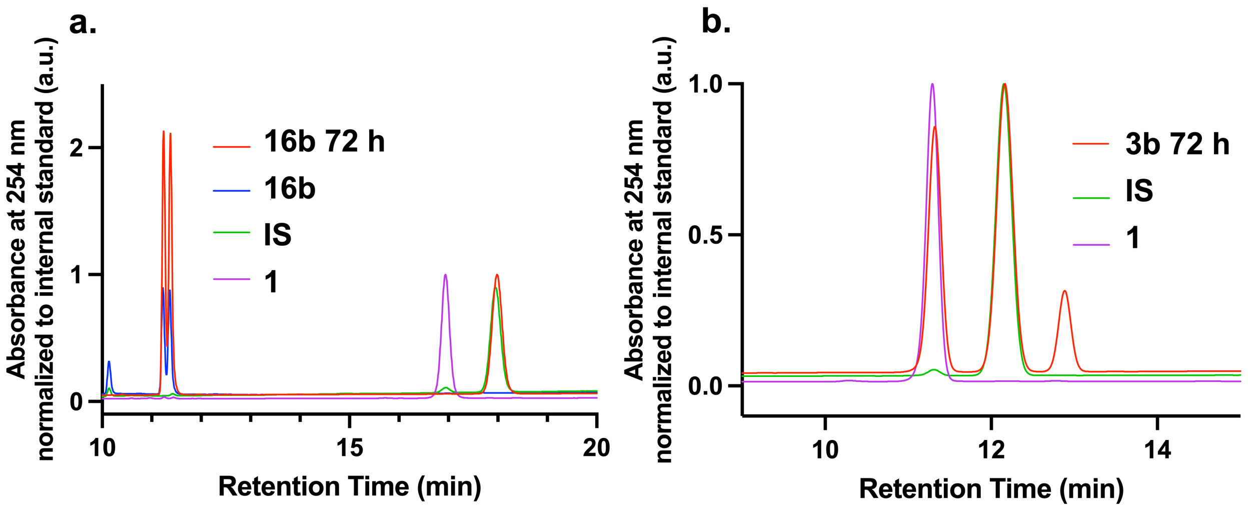

Figure S35. Peak Assignment for Stability and Release Assays for 16b. (a) A representative HPLC trace from the stability assay for **16b** after incubation for 72 h at 37°C in PBS buffer (red) is shown overlaid with the trace for **16b** (blue), **1** (purple), and the warfarin IS (green). (b) A representative HPLC trace from the release assay for **3b** after incubation for 72 h at 37°C in pH 7 buffer (red) is shown overlaid with the trace for **1** (purple) and the warfarin IS (green).

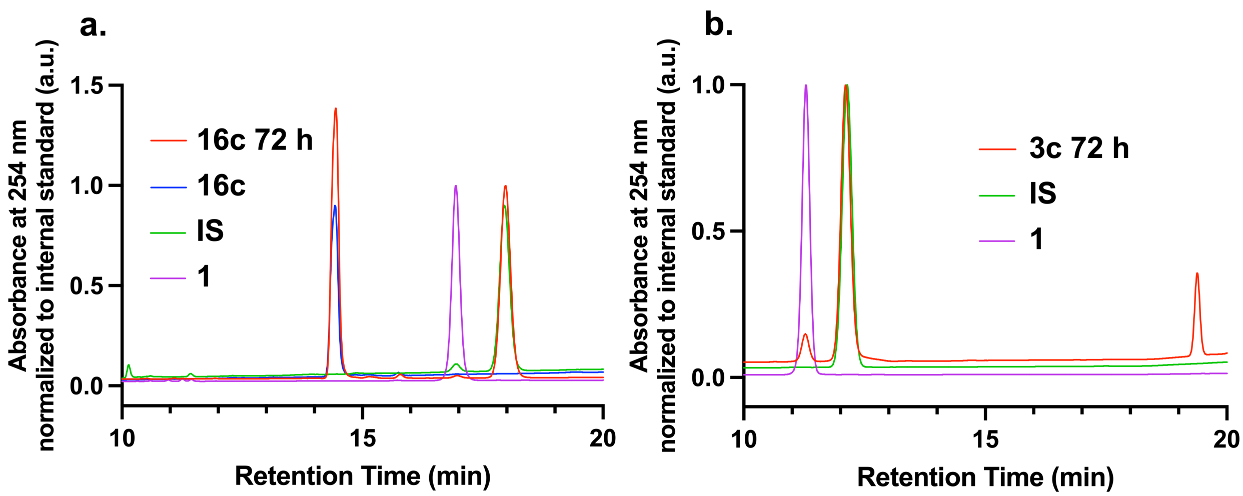

Figure S36. Peak Assignment for Stability and Release Assays for 16c. (a) A representative HPLC trace from the stability assay for **16c** after incubation for 72 h at 37°C in PBS buffer (red) is shown overlaid with the trace for **16c** (blue), **1** (purple), and the warfarin IS (green). (b) A representative HPLC trace from the release assay for **3c** after incubation for 72 h at 37°C in pH 7 buffer (red) is shown overlaid with the trace for **1** (purple) and the warfarin IS (green).

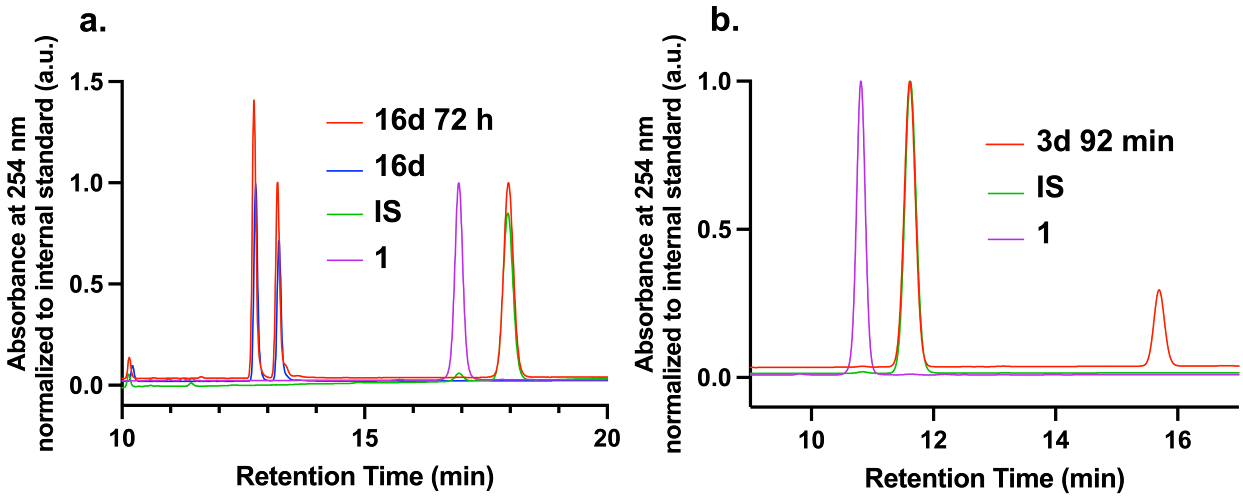

Figure S37. Peak Assignment for Stability and Release Assays for 16d. (a) A representative HPLC trace from the stability assay for **16d** after incubation for 72 h at 37°C in PBS buffer (red) is shown overlaid with the trace for **16d** (blue), **1** (purple), and the warfarin IS (green). (b) A representative HPLC trace from the release assay for **3d** after incubation for 92 min at 37°C in pH 7 buffer (red) is shown overlaid with the trace for **1** (purple) and the warfarin IS (green).

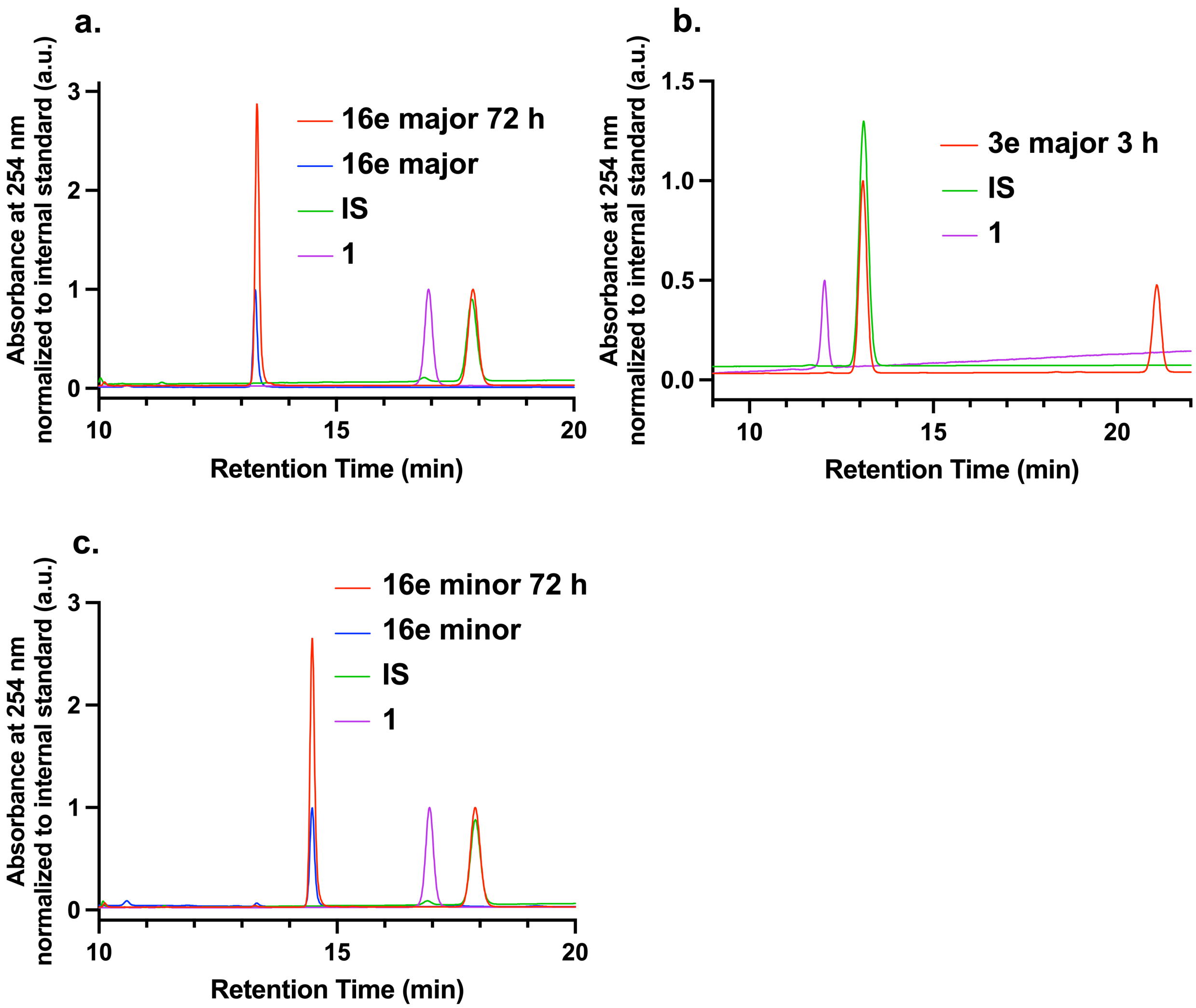

Figure S38. Peak Assignment for Stability and Release Assays for 16e. (a) A representative HPLC trace from the stability assay for the **16e major product** after incubation for 72 h at 37°C in PBS buffer (red) is shown overlaid with the trace for the **16e major product** (blue), **1** (purple), and the warfarin IS (green). (b) A representative HPLC trace from the release assay for the **3e major product** after incubation for 3 h at 37°C in pH 7 buffer (red) is shown overlaid with the trace for **1** (purple) and the warfarin IS (green). (c) A representative HPLC trace from the stability assay for the **16e minor product** after incubation for 72 h at 37°C in PBS buffer (red) is shown overlaid with the trace for the **16e minor product** (blue), **1** (purple), and the warfarin IS (green).

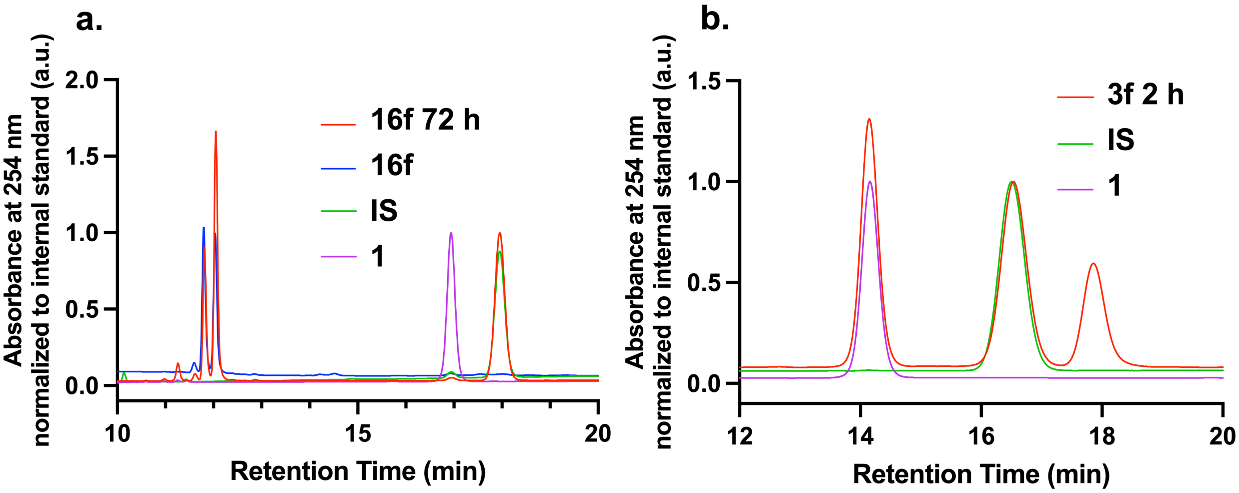

Figure S39. Peak Assignment for Stability and Release Assays for 16f. (a) A representative HPLC trace from the stability assay for **16f** after incubation for 72 h at 37°C in PBS buffer (red) is shown overlaid with the trace for **16f** (blue), **1** (purple), and the warfarin IS (green). (b) A representative HPLC trace from the release assay for **3f** after incubation for 2 h at 37°C in pH 7 buffer (red) is shown overlaid with the trace for **1** (purple) and the warfarin IS (green).

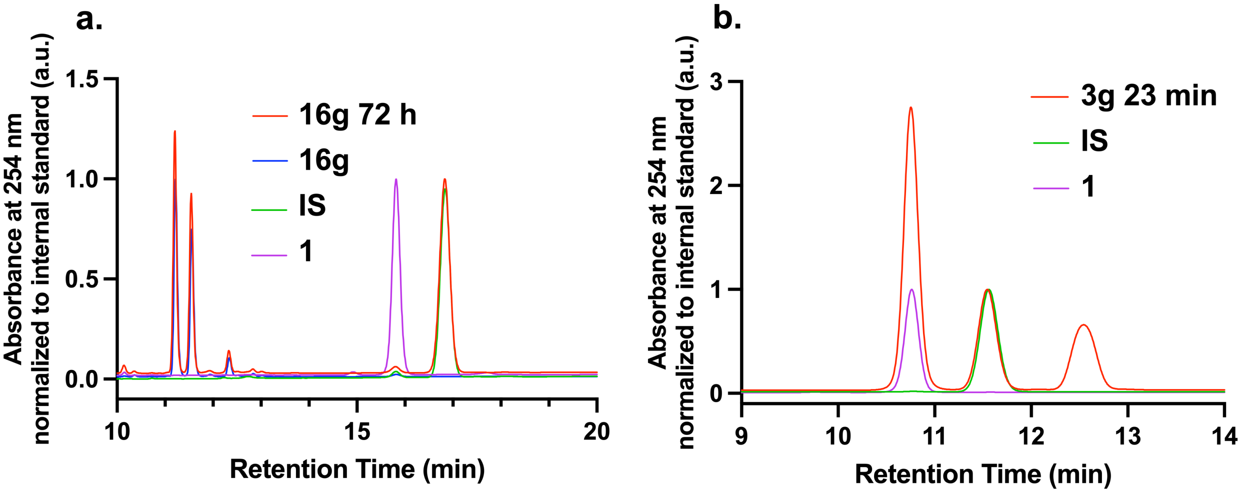

Figure S40. Peak Assignment for Stability and Release Assays for 16g. (a) A representative HPLC trace from the stability assay for **16g** after incubation for 72 h at 37°C in PBS buffer (red) is shown overlaid with the trace for **16g** (blue), **1** (purple), and the warfarin IS (green). (b) A representative HPLC trace from the release assay for **3g** after incubation for 23 min at 37°C in pH 7 buffer (red) is shown overlaid with the trace for **1** (purple) and the warfarin IS (green).

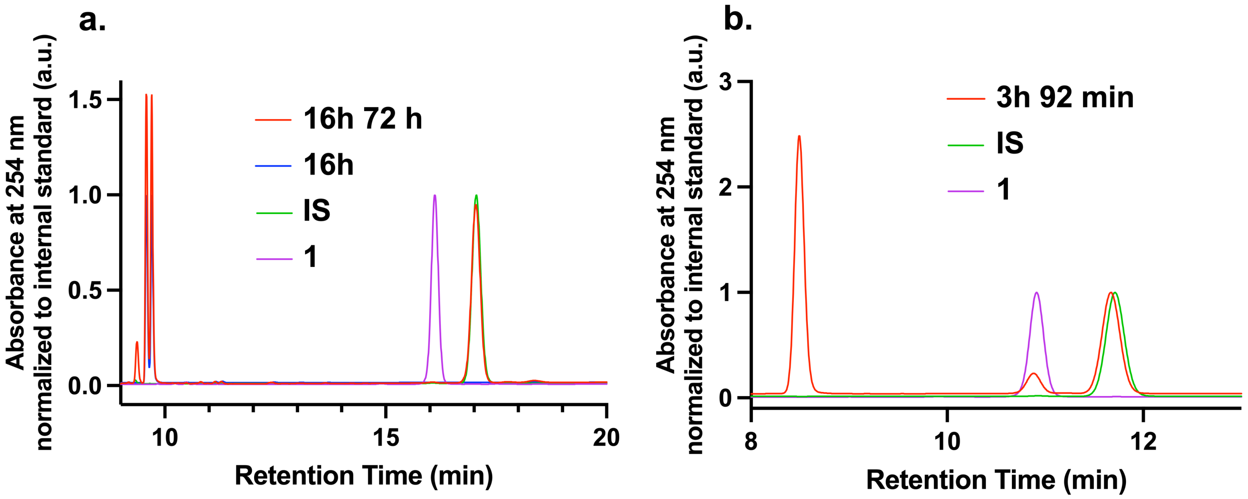

Figure S41. Peak Assignment for Stability and Release Assays for 16h. (a) A representative HPLC trace from the stability assay for **16h** after incubation for 72 h at 37°C in PBS buffer (red) is shown overlaid with the trace for **16h** (blue), **1** (purple), and the warfarin IS (green). (b) A representative HPLC trace from the release assay for **3h** after incubation for 92 min at 37°C in pH 7 buffer (red) is shown overlaid with the trace for **1** (purple) and the warfarin IS (green).

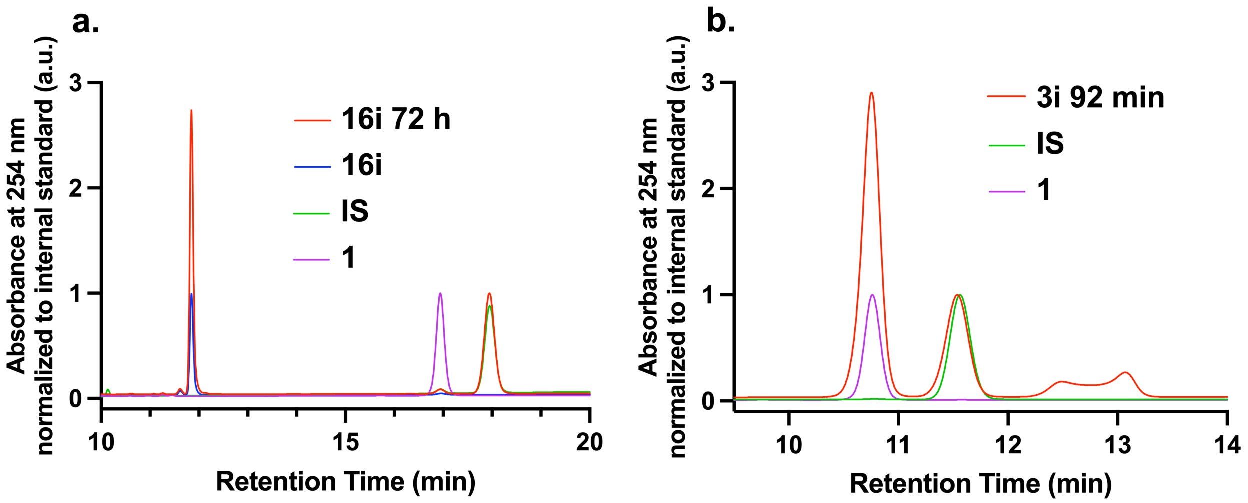

Figure S42. Peak Assignment for Stability and Release Assays for 16i. (a) A representative HPLC trace from the stability assay for **16i** after incubation for 72 h at 37°C in PBS buffer (red) is shown overlaid with the trace for **16i** (blue), **1** (purple), and the warfarin IS (green). (b) A representative HPLC trace from the release assay for **3i** after incubation for 92 min at 37°C in pH 7 buffer (red) is shown overlaid with the trace for **1** (purple) and the warfarin IS (green).

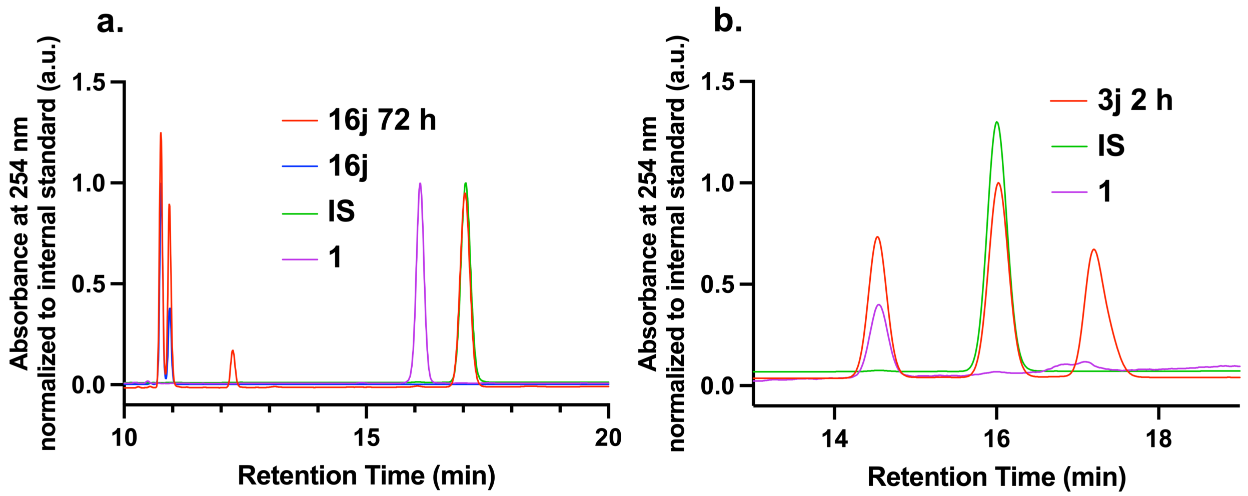

Figure S43. Peak Assignment for Stability and Release Assays for 16j. (a) A representative HPLC trace from the stability assay for **16j** after incubation for 72 h at 37°C in PBS buffer (red) is shown overlaid with the trace for **16j** (blue), **1** (purple), and the warfarin IS (green). (b) A representative HPLC trace from the release assay for **3j** after incubation for 2 h at 37°C in pH 7 buffer (red) is shown overlaid with the trace for **1** (purple) and the warfarin IS (green).

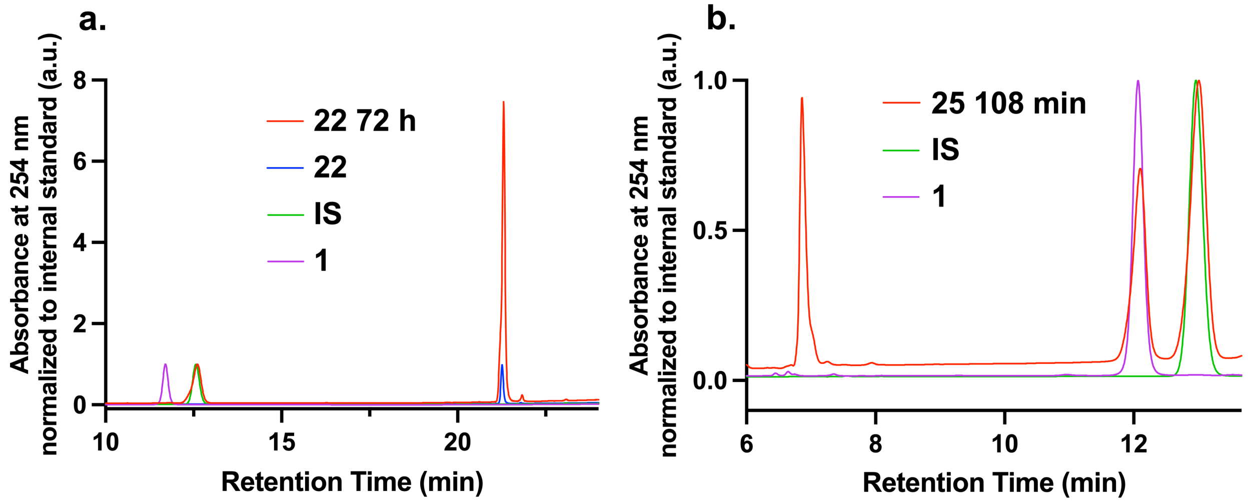

Figure S44. Peak Assignment for Stability and Release Assays for 22. (a) A representative HPLC trace from the stability assay for **22** after incubation for 72 h at 37°C in PBS buffer (red) is shown overlaid with the trace for **22** (blue), **1** (purple), and the warfarin IS (green). (b) A representative HPLC trace from the release assay for **25** after incubation for 108 min at 37°C in pH 7 buffer (red) is shown overlaid with the trace for **1** (purple) and the warfarin IS (green).

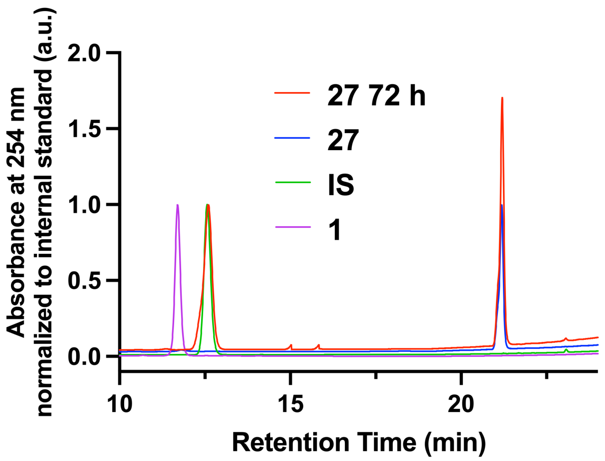

Figure S45. Peak Assignment for Stability Assay for 27. A representative HPLC trace from the stability assay for **27** after incubation for 72 h at 37°C in PBS buffer (red) is shown overlaid with the trace for **27** (blue), **1** (purple), and the warfarin IS (green).

4.4. Graphs of calculated k_{obs} on a non-logarithmic scale

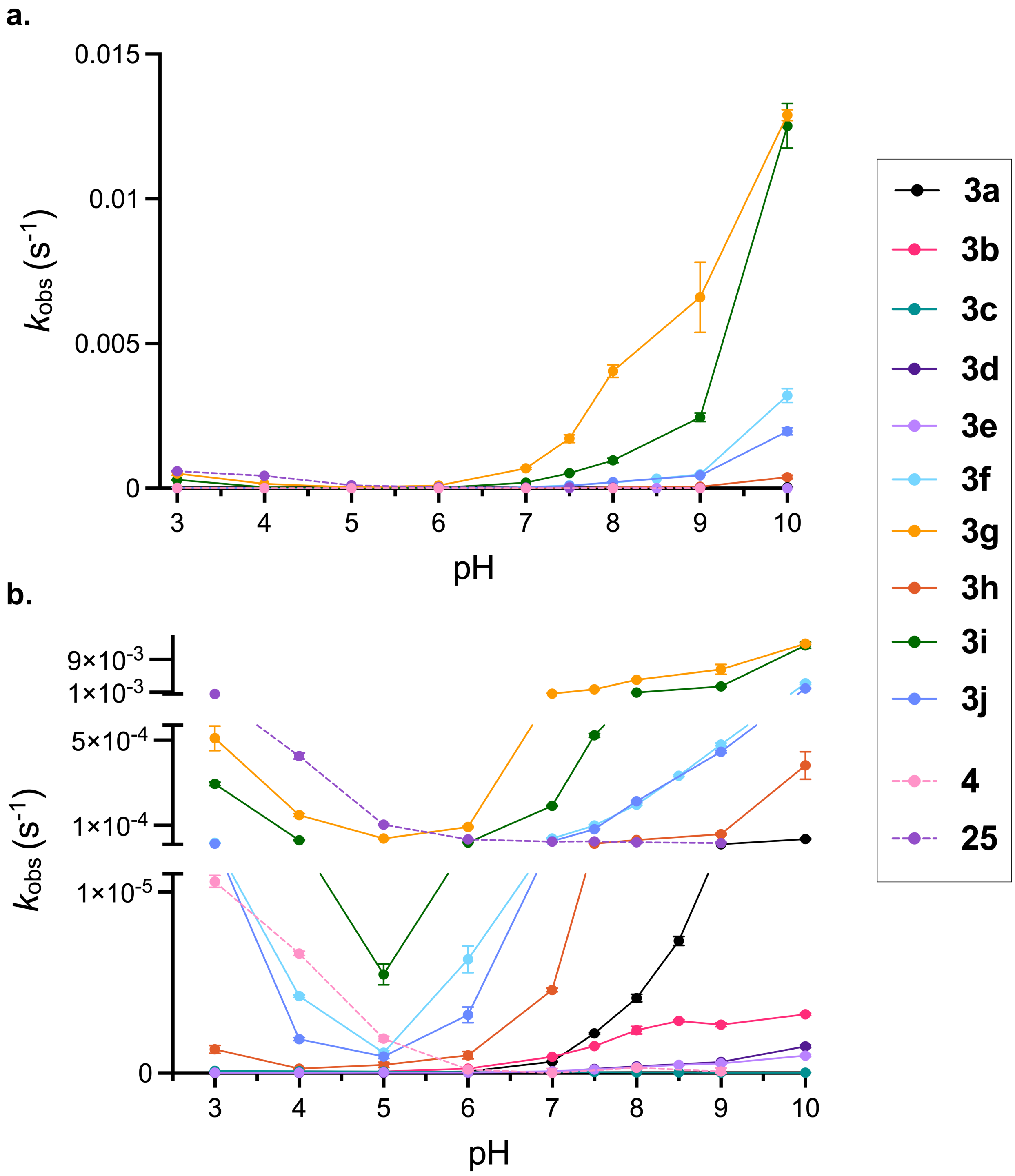

Figure S46. First Order Rate Constants for PHB and PAB Derivatives. The calculated k_{obs} values for the studied PHB and PAB prodrugs are plotted vs pH. (a) These values are plotted on a non-logarithmic scale. (b) A zoomed in view of (a) is shown such that the relative k_{obs} for all compounds from pH 3-7 can be examined. Error bars represent ± 95% CI.

4.5. Tables of kinetics data

Table S4.1. Kinetic parameters derived for β-lapachone release from 3a.

pH	k_{obs} (s^{-1})	95% CI	$t_{1/2}$ (hr)	95% CI
**3**	8.39×10^{-8}	$9.09 \times 10^{-8} - 7.69 \times 10^{-8}$	2294.07	2117.70 – 2502.48
**4**	4.90×10^{-8}	$5.22 \times 10^{-8} - 4.57 \times 10^{-8}$	3933.42	3687.82 – 4214.07
**5**	1.74×10^{-8}	$2.15 \times 10^{-8} - 1.34 \times 10^{-8}$	11040.19	8938.76 – 14422.54
**6**	8.12×10^{-8}	$8.86 \times 10^{-8} - 7.38 \times 10^{-8}$	2370.61	2173.39 – 2607.54
**7**	6.39×10^{-7}	$6.55 \times 10^{-7} - 6.22 \times 10^{-7}$	301.55	294.14 – 309.40
**7.4**	8.68×10^{-7}	$9.37 \times 10^{-7} - 7.99 \times 10^{-7}$	221.80	205.46 – 240.98
**7.5**	2.19×10^{-6}	$2.23 \times 10^{-6} - 2.15 \times 10^{-6}$	87.88	86.23 – 89.55
**8**	4.15×10^{-6}	$4.36 \times 10^{-6} - 3.94 \times 10^{-6}$	46.43	44.21 – 48.87
**8.5**	7.30×10^{-6}	$7.54 \times 10^{-6} - 7.06 \times 10^{-6}$	26.38	25.53 – 27.29
**9**	1.16×10^{-5}	$1.17 \times 10^{-5} - 1.14 \times 10^{-5}$	16.67	16.41 – 16.93
**10**	3.68×10^{-5}	$3.98 \times 10^{-5} - 3.37 \times 10^{-5}$	5.24	4.83 – 5.72

Table S4.2. Kinetic parameters derived for β-lapachone release from 3b.

pH	k_{obs} (s^{-1})	95% CI	$t_{1/2}$ (hr)	95% CI
**3**	7.49×10^{-8}	$7.85 \times 10^{-8} - 7.14 \times 10^{-8}$	2569.95	2454.00 – 2697.41
**4**	1.00×10^{-7}	$1.05 \times 10^{-7} - 9.56 \times 10^{-8}$	1919.65	1833.72 – 2014.45
**5**	8.60×10^{-8}	$8.85 \times 10^{-8} - 8.36 \times 10^{-8}$	2237.81	2176.83 – 2302.57
**6**	2.41×10^{-7}	$2.52 \times 10^{-7} - 2.29 \times 10^{-7}$	800.59	763.75 – 841.16
**7**	8.94×10^{-7}	$9.22 \times 10^{-7} - 8.66 \times 10^{-7}$	215.32	208.78 – 222.26
**7.5**	1.49×10^{-6}	$1.52 \times 10^{-6} - 1.46 \times 10^{-6}$	128.96	126.34 – 131.70

**8**	2.38×10^{-6}	$2.58 \times 10^{-6} – 2.17 \times 10^{-6}$	81.00	74.54 – 88.69
**8.5**	2.88×10^{-6}	$2.94 \times 10^{-6} – 2.82 \times 10^{-6}$	66.85	65.40 – 68.35
**9**	2.67×10^{-6}	$2.78 \times 10^{-6} – 2.56 \times 10^{-6}$	72.06	69.26 – 75.09
**10**	3.25×10^{-6}	$3.31 \times 10^{-6} – 3.19 \times 10^{-6}$	59.30	58.19 – 60.45

Table S4.3. Kinetic parameters derived for β-lapachone release from 3c.

pH	k_{obs} (s^{-1})	95% CI	$t_{1/2}$ (hr)	95% CI
**3**	1.14×10^{-7}	$1.20 \times 10^{-7} – 1.08 \times 10^{-7}$	1690.44	1600.51 – 1791.08
**4**	9.85×10^{-8}	$1.09 \times 10^{-7} – 8.85 \times 10^{-8}$	1954.93	1774.57 – 2174.87
**5**	9.34×10^{-8}	$9.89 \times 10^{-8} – 8.78 \times 10^{-8}$	2061.91	1946.63 – 2191.95
**6**	4.82×10^{-8}	$5.29 \times 10^{-8} – 4.34 \times 10^{-8}$	3998.77	3639.71 – 4436.43
**7**	6.67×10^{-8}	$7.26x 10^{-8} – 6.07 \times 10^{-8}$	2888.40	2652.81 – 3169.92
**7.5**	5.34×10^{-8}	$5.84 \times 10^{-8} – 4.84 \times 10^{-8}$	3606.31	3295.24 – 3981.41
**8**	4.04×10^{-8}	$4.51 \times 10^{-8} – 3.56 \times 10^{-8}$	4771.77	4269.20 – 5409.97
**8.5**	3.48×10^{-8}	$4.14 \times 10^{-8} – 2.82 \times 10^{-8}$	5531.19	4652.99 – 6818.02
**9**	2.80×10^{-8}	$3.45 \times 10^{-8} – 2.15 \times 10^{-8}$	6874.01	5579.28 – 8951.23
**10**	2.99×10^{-8}	$3.39 \times 10^{-8} – 2.60 \times 10^{-8}$	6430.89	5681.35 – 7405.42

Table S4.4. Kinetic parameters derived for β-lapachone release from 3d.

pH	k_{obs} (s^{-1})	95% CI	$t_{1/2}$ (hr)	95% CI
**3**	1.25×10^{-8}	$2.52 \times 10^{-8} – 0.00$	15440.33	7637.48 – ∞
**4**	6.50×10^{-9}	$1.89 \times 10^{-8} – 0.00$	29608.01	10176.58 – ∞
**5**	8.27×10^{-9}	$1.66 \times 10^{-8} – 0.00$	23284.66	11619.85 – ∞
**6**	3.14×10^{-8}	$4.11 \times 10^{-8} – 2.16 \times 10^{-8}$	6137.74	4682.41 – 8909.80

**7**	7.76×10^{-8}	$8.73 \times 10^{-8} – 6.79 \times 10^{-8}$	2480.88	2204.75 – 2836.07
**7.5**	2.31×10^{-7}	$2.55 \times 10^{-7} – 2.07 \times 10^{-7}$	834.96	756.25 – 931.95
**8**	3.74×10^{-7}	$3.94 \times 10^{-7} – 3.53 \times 10^{-7}$	515.50	488.19 – 546.22
**9**	6.09×10^{-7}	$6.41 \times 10^{-7} – 5.77 \times 10^{-7}$	316.21	300.24 – 333.98
**10**	1.48×10^{-6}	$1.56 \times 10^{-6} – 1.40 \times 10^{-6}$	129.92	123.27 – 137.24

Table S4.5. Kinetic parameters derived for β-lapachone release from 3e (obtained from the 16e major diastereomer).

pH	k_{obs} (s^{-1})	95% CI	$t_{1/2}$ (hr)	95% CI
**3**	1.99×10^{-8}	$2.41 \times 10^{-8} – 1.57 \times 10^{-8}$	9665.71	7976.01 – 12255.94
**4**	3.16×10^{-8}	$3.81 \times 10^{-8} – 2.52 \times 10^{-8}$	6091.14	5057.55 – 7655.70
**5**	3.73×10^{-8}	$4.40 \times 10^{-8} – 3.06 \times 10^{-8}$	5167.50	4378.91 – 6300.42
**6**	4.50×10^{-8}	$5.31 \times 10^{-8} – 3.68 \times 10^{-8}$	4283.45	3626.69 – 5230.67
**7**	1.03×10^{-7}	$1.11 \times 10^{-7} – 9.44 \times 10^{-8}$	1876.62	1739.30 – 2038.98
**7.5**	2.07×10^{-7}	$2.49 \times 10^{-7} – 1.65 \times 10^{-7}$	929.70	773.57 – 1165.50
**8**	3.20×10^{-7}	$3.37 \times 10^{-7} – 3.03 \times 10^{-7}$	601.50	571.51 – 634.82
**8.5**	4.50×10^{-7}	$4.73 \times 10^{-7} – 4.26 \times 10^{-7}$	428.15	406.72 – 451.87
**9**	5.40×10^{-7}	$6.13 \times 10^{-7} – 4.66 \times 10^{-7}$	356.89	313.89 – 413.53
**10**	9.74×10^{-7}	$1.05 \times 10^{-6} – 8.99 \times 10^{-7}$	197.72	183.55 – 214.24

Table S4.6. Kinetic parameters derived for β-lapachone release from 3f.

pH	k_{obs} (s^{-1})	95% CI	$t_{1/2}$ (hr)	95% CI
**3**	1.84×10^{-5}	$2.02 \times 10^{-5} – 1.67 \times 10^{-5}$	10.44	9.54 – 11.54
**4**	4.25×10^{-6}	$4.33 \times 10^{-6} – 4.18 \times 10^{-6}$	45.29	44.52 – 46.10

**5**	1.13×10^{-6}	$1.25 \times 10^{-6} – 1.00 \times 10^{-6}$	170.84	153.79 – 191.96
**6**	6.28×10^{-6}	$7.02 \times 10^{-6} – 5.54 \times 10^{-6}$	30.66	27.45 – 34.74
**7**	3.95×10^{-5}	$4.10 \times 10^{-5} – 3.79 \times 10^{-5}$	4.88	4.70 – 5.08
**7.5**	9.98×10^{-5}	$1.04 \times 10^{-4} – 9.55 \times 10^{-5}$	1.93	1.85 – 2.02
**8**	1.97×10^{-4}	$2.02 \times 10^{-4} – 1.93 \times 10^{-4}$	0.975	0.955 – 0.997
**8.5**	3.34×10^{-4}	$3.39 \times 10^{-4} – 3.29 \times 10^{-4}$	0.577	0.569 – 0.586
**9**	4.80×10^{-4}	$4.86 \times 10^{-4} – 4.74 \times 10^{-4}$	0.401	0.396 – 0.406
**10**	3.21×10^{-3}	$3.44 \times 10^{-3} – 2.97 \times 10^{-3}$	0.060	0.056 – 0.065

Table S4.7. Kinetic parameters derived for β-lapachone release from 3g.

**pH**	k_{obs} **(s^{-1})**	**95% CI**	$t_{1/2}$ **(hr)**	**95% CI**
**3**	5.09×10^{-4}	$4.51 \times 10^{-4} – 5.67 \times 10^{-4}$	0.378	0.427 – 0.339
**4**	1.49×10^{-4}	$1.42 \times 10^{-4} – 1.56 \times 10^{-4}$	1.29	1.36 – 1.24
**5**	3.90×10^{-5}	$3.56 \times 10^{-5} – 4.24 \times 10^{-5}$	4.94	5.40 – 4.55
**6**	9.27×10^{-5}	$8.93 \times 10^{-5} – 9.62 \times 10^{-5}$	2.08	2.16 – 2.00
**7**	6.87×10^{-4}	$6.51 \times 10^{-4} – 7.23 \times 10^{-4}$	0.280	0.296 – 0.266
**7.5**	1.72×10^{-3}	$1.58 \times 10^{-3} – 1.85 \times 10^{-3}$	0.112	0.122 – 0.104
**8**	4.05×10^{-3}	$3.83 \times 10^{-3} – 4.27 \times 10^{-3}$	0.048	0.050 – 0.045
**9**	6.60×10^{-3}	$7.82 \times 10^{-3} – 5.38 \times 10^{-3}$	0.029	0.025 – 0.036
**10**	1.29×10^{-2}	$1.31 \times 10^{-2} – 1.27 \times 10^{-2}$	0.0149	0.0147 – 0.0151

Table S4.8. Kinetic parameters derived for β-lapachone release from 3h.

**pH**	k_{obs} **(s^{-1})**	**95% CI**	$t_{1/2}$ **(hr)**	**95% CI**
**3**	1.31×10^{-6}	$1.53 \times 10^{-6} – 1.09 \times 10^{-6}$	146.98	125.93 – 176.32

**4**	2.41×10^{-7}	$2.76 \times 10^{-7} – 2.06 \times 10^{-7}$	800.25	698.37 – 936.48
**5**	4.56×10^{-7}	$6.03 \times 10^{-7} – 3.08 \times 10^{-7}$	422.61	319.15 – 625.13
**6**	9.82×10^{-7}	$1.18 \times 10^{-6} – 7.83 \times 10^{-7}$	196.01	162.89 – 246.00
**7**	4.59×10^{-6}	$4.69 \times 10^{-6} – 4.50 \times 10^{-6}$	41.95	41.09 – 42.83
**7.5**	1.47×10^{-5}	$1.52 \times 10^{-5} – 1.42 \times 10^{-5}$	13.10	12.66 – 13.57
**8**	3.27×10^{-5}	$3.42 \times 10^{-5} – 3.12 \times 10^{-5}$	5.89	5.63 – 6.18
**9**	5.89×10^{-5}	$6.31 \times 10^{-5} – 5.47 \times 10^{-5}$	3.27	3.05 – 3.52
**10**	3.82×10^{-4}	$4.47 \times 10^{-4} – 3.17 \times 10^{-4}$	0.504	0.431 – 0.607

Table S4.9. Kinetic parameters derived for β-lapachone release from 3i.

pH	k_{obs} (s^{-1})	95% CI	$t_{1/2}$ (hr)	95% CI
**3**	2.95×10^{-4}	$3.03 \times 10^{-4} – 2.87 \times 10^{-4}$	0.653	0.636 – 0.671
**4**	3.10×10^{-5}	$3.44 \times 10^{-5} – 2.76 \times 10^{-5}$	6.22	5.60 – 6.98
**5**	5.46×10^{-6}	$6.03 \times 10^{-6} – 4.88 \times 10^{-6}$	35.28	31.91 – 39.44
**6**	1.96×10^{-5}	$2.14 \times 10^{-5} – 1.78 \times 10^{-5}$	9.83	9.02 – 10.81
**7**	1.92×10^{-4}	$1.95 \times 10^{-4} – 1.89 \times 10^{-4}$	1.004	0.987 – 1.021
**7.5**	5.23×10^{-4}	$5.31 \times 10^{-4} – 5.14 \times 10^{-4}$	0.368	0.363 – 0.374
**8**	9.62×10^{-4}	$1.04 \times 10^{-3} – 8.86 \times 10^{-4}$	0.200	0.186 – 0.217
**9**	2.45×10^{-3}	$2.60 \times 10^{-3} – 2.31 \times 10^{-3}$	0.0785	0.0741 – 0.0835
**10**	1.25×10^{-2}	$1.33 \times 10^{-2} – 1.18 \times 10^{-2}$	0.0154	0.0145 – 0.0164

Table S4.10. Kinetic parameters derived for β-lapachone release from 3j.

pH	k_{obs} (s^{-1})	95% CI	$t_{1/2}$ (hr)	95% CI
**3**	1.39×10^{-5}	$1.49 \times 10^{-5} – 1.30 \times 10^{-5}$	13.82	12.97 – 14.80

**4**	1.87×10^{-6}	$1.95 \times 10^{-6} – 1.79 \times 10^{-6}$	102.85	98.59 – 107.50
**5**	9.20×10^{-7}	$1.04 \times 10^{-6} – 8.03 \times 10^{-7}$	209.28	185.67 – 239.84
**6**	3.22×10^{-6}	$3.66 \times 10^{-6} – 2.78 \times 10^{-6}$	59.81	52.66 – 69.21
**7**	2.59×10^{-5}	$2.72 \times 10^{-5} – 2.47 \times 10^{-5}$	7.42	7.09 – 7.79
**7.5**	8.23×10^{-5}	$8.49 \times 10^{-5} – 7.97 \times 10^{-5}$	2.34	2.27 – 2.42
**8**	2.14×10^{-4}	$2.18 \times 10^{-4} – 2.09 \times 10^{-4}$	0.901	0.882 – 0.919
**9**	4.46×10^{-4}	$4.51 \times 10^{-4} – 4.41 \times 10^{-4}$	0.432	0.427 – 0.437
**10**	1.97×10^{-3}	$2.09 \times 10^{-3} – 1.85 \times 10^{-3}$	0.098	0.092 – 0.104

Table S4.11. Kinetic parameters derived for β-lapachone release from 25.

**pH**	k_{obs} **(s^{-1})**	**95% CI**	$t_{1/2}$ **(hr)**	**95% CI**
**3**	5.94×10^{-4}	$6.08 \times 10^{-4} – 5.79 \times 10^{-4}$	0.324	0.317 – 0.332
**4**	4.26×10^{-4}	$4.40 \times 10^{-4} – 4.12 \times 10^{-4}$	0.452	0.438 – 0.467
**5**	1.04×10^{-4}	$1.07 \times 10^{-4} – 1.00 \times 10^{-4}$	1.86	1.80 – 1.92
**6**	3.49×10^{-5}	$3.63 \times 10^{-5} – 3.35 \times 10^{-5}$	5.51	5.30 – 5.74
**7**	2.33×10^{-5}	$2.43 \times 10^{-5} – 2.22 \times 10^{-5}$	8.27	7.92 – 8.66
**7.5**	2.56×10^{-5}	$2.94 \times 10^{-5} – 2.18 \times 10^{-5}$	7.52	6.54 – 8.83
**8**	2.13×10^{-5}	$2.70 \times 10^{-5} – 1.57 \times 10^{-5}$	9.03	7.14 – 12.27
**9**	1.67×10^{-5}	$2.15 \times 10^{-5} – 1.20 \times 10^{-5}$	11.50	8.95 – 16.10

Table S4.12. Kinetic parameters derived for β-lapachone release from 3a (obtained from 16a) in the presence of hydrogen peroxide.

**pH**	k_{obs} **(s^{-1})**	**95% CI**	$t_{1/2}$ **(hr)**	**95% CI**
**7.4**	9.95×10^{-7}	$1.06 \times 10^{-6} – 9.36 \times 10^{-7}$	193.45	182.50 – 205.75

**9**	1.17×10^{-5}	$1.21 \times 10^{-5} – 1.14 \times 10^{-5}$	16.41	15.98 – 16.87

Table S4.13. Kinetic parameters derived for β-lapachone release from 3a (obtained from 24 (26)) in the presence of hydrogen peroxide (k_2).

pH	k_2 (s^{-1})	95% CI	$t_{1/2}$ (hr)	95% CI
**5**	1.88×10^{-7}	$8.49 \times 10^{-8} – 2.91 \times 10^{-7}$	1025.79	662.56 – 2267.59
**7.4**	1.49×10^{-6}	$1.44 \times 10^{-6} – 1.56 \times 10^{-6}$	128.62	123.58 – 133.99
**9**	2.11×10^{-5}	$2.00 \times 10^{-5} – 2.23 \times 10^{-5}$	9.09	8.63 – 9.58

Table S4.14. Kinetic parameters derived for conversion of 26 into 3a (k_1).

pH	k_1 (s^{-1})	95% CI	$t_{1/2}$ (hr)	95% CI
**5**	1.78×10^{-5}	$1.87 \times 10^{-5} – 1.69 \times 10^{-5}$	10.82	10.28 – 11.42
**7.4**	6.81×10^{-4}	$7.41 \times 10^{-4} – 6.20 \times 10^{-4}$	0.283	0.260 – 0.310
**9**	1.52×10^{-2}	$1.52 \times 10^{-2} – 1.51 \times 10^{-2}$	0.01269	0.01266 – 0.01273

Section 5. Supplemental figures for mechanistic discussion and quantum mechanical calculations

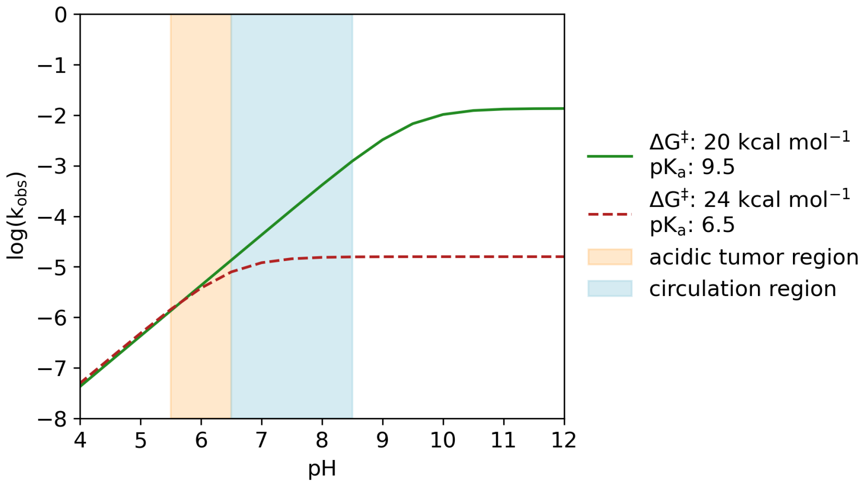

Figure S47. pH-Dependence of the observed rate constant (k_{obs}, logarithmic scale; calculated with equation 3) for two hypothetical reactions characterized by different intrinsic activation free energies ($\Delta G^{\ddagger}$ = 20 and 24 kcal mol^{-1}; solid and dashed lines, respectively) and apparent pK_a values (6.5 or 9.5; red and green lines, respectively). At acidic pH values corresponding to the tumor microenvironment (TME; 5.5–6.5, orange region), the rate increase from lowering the apparent pK_a by 3 units is completely counterbalanced by a 4 kcal mol^{-1} increase in $\Delta G^{\ddagger}$. By contrast, under near-neutral conditions representative of physiological circulation (pH 6.5–8.5, blue region), the differences in k_{obs} are maximized.

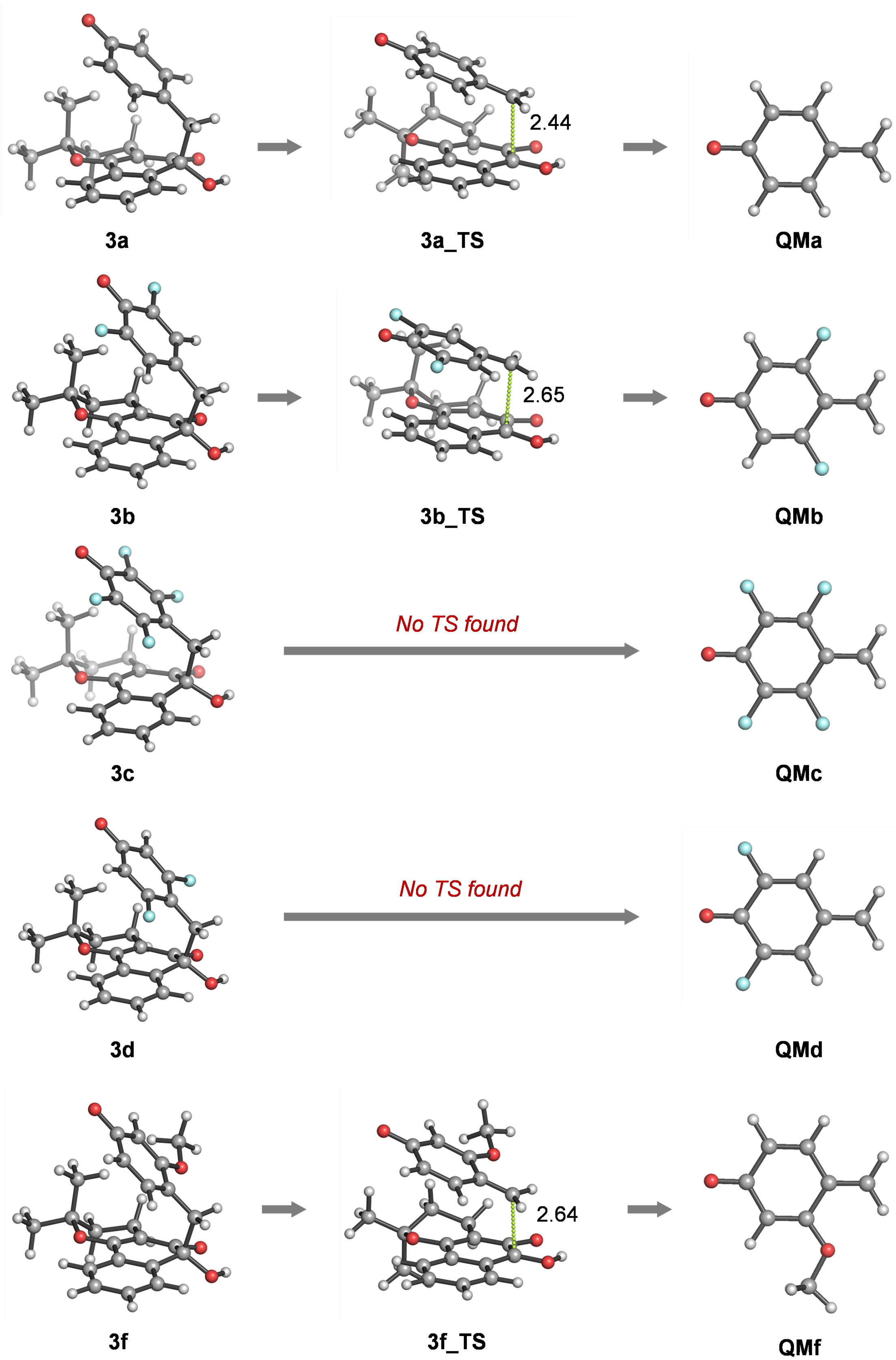

2.44
3a
3a_TS
QMa
2.65
3b
3b_TS
QMb
No TS found
3c
QMc
No TS found
3d
QMd
2.64
3f
3f_TS
QMf

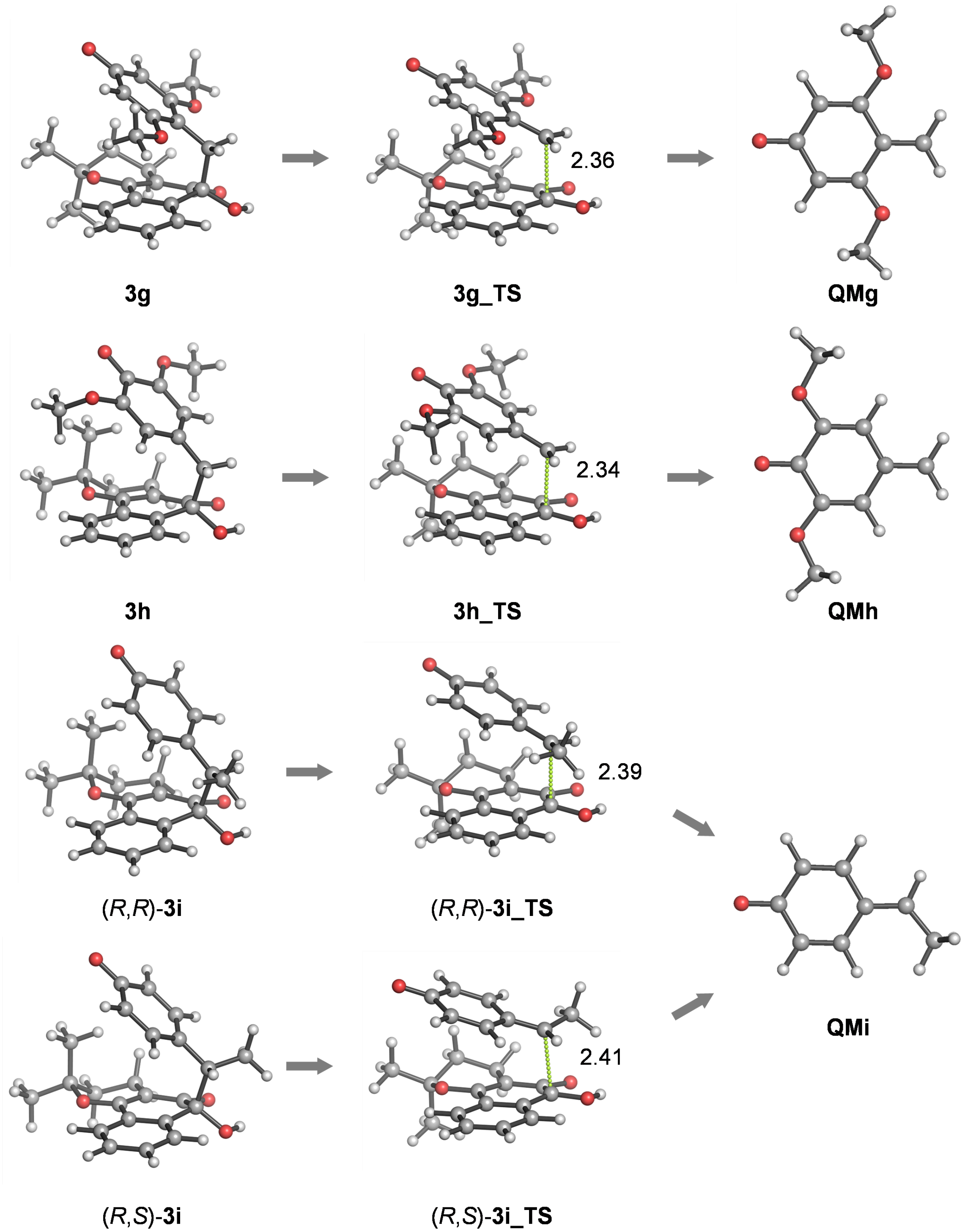

Figure S48. Lowest-energy structures of the PHB phenolates, transition state structures (TS), and quinone methide (QM) products for the 1,6-elimination reactions calculated at the PCM(H_2O)/M06-2X/6-31+G(d,p) level. Breaking bonds are represented with green dotted lines. Distances are given in angstrom.

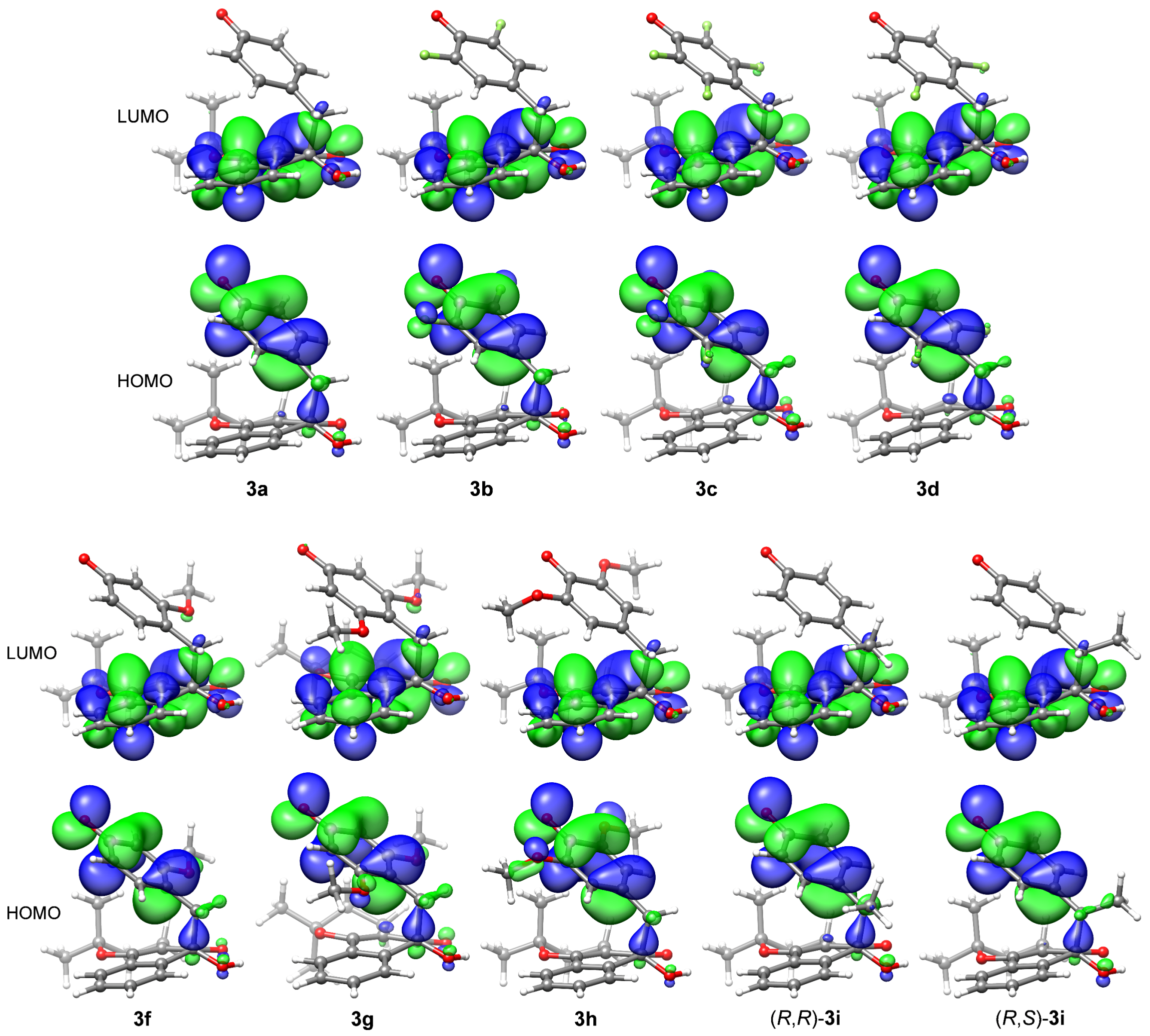

	E_{HOMO} (eV)	E_{LUMO} (eV)	$\Delta E_{HOMO–LUMO}$ (eV)
**3a**	–5.8	–1.1	**4.7**
**3b**	–6.1	–1.1	**5.0**
**3c**	–6.4	–1.1	**5.3**
**3d**	–6.2	–1.1	**5.1**
**3f**	–5.8	–1.0	**4.8**
**3g**	–5.8	–0.9	**4.8**
**3h**	–5.6	–1.1	**4.5**
(*R*,*R*)-**3i**	–5.8	–1.0	**4.7**
(*R*,S)-**3i**	–5.8	–1.0	**4.8**

Figure S49. HOMO and LUMO of phenolates 3a-i calculated at the PCM(H_2O)/M06-2X/6-31+G(d,p) level.

Table S5.1. Energies, entropies, and lowest frequencies of the lowest energy calculated structures.[a]

Structure	E_{elec} (Hartree)	E_{elec} + ZPE (Hartree)	H (Hartree)	S (cal mol^{-1} K^{-1})	G (Hartree)	Lowest freq. (cm^{-1})	# of imag freq.
**3a**	–1152.118454	–1151.721287	–1151.698089	154.1	–1151.768084	31.6	0
**3a_isomerB**	–1152.106960	–1151.710225	–1151.686888	156.1	–1151.756978	20.2	0
**3a$_{PhO-}$**	–1151.638124	–1151.254397	–1151.231626	152.4	–1151.300987	30.8	0
**3a_TS**	–1151.604016	–1151.223553	–1151.200450	154.4	–1151.270616	–105.0	1
**QMa**	–345.413358	–345.303533	–345.296111	80.1	–345.334100	94.4	0
**3a$_{Zw1}$**	–1152.065786	–1151.668982	–1151.645933	154.1	–1151.715704	23.4	0
**3a$_{Zw1}$_TS**	–1152.054571	–1151.659991	–1151.636890	152.4	–1151.706754	–447.1	1
**3b**	–1350.065272	–1349.697620	–1349.673025	161.4	–1349.745800	25.0	0
**3b_TS**	–1350.022842	–1349.658354	–1349.633687	160.0	–1349.706828	–69.5	1
**QMb**	–543.828526	–543.734676	–543.725652	88.5	–543.767654	94.0	0
**3c**	–1548.478884	–1548.127153	–1548.100661	170.0	–1548.177189	17.5	0
**QMc**	–742.231955	–742.153761	–742.142995	97.1	–742.189076	94.9	0
**3d**	–1350.070016	–1349.702425	–1349.677893	160.4	–1349.750603	26.3	0
**QMd**	–543.834811	–543.740826	–543.731883	88.2	–543.773722	91.2	0
**3f**	–1266.125369	–1265.708313	–1265.683087	162.9	–1265.757222	26.5	0
**3f_TS**	–1266.095536	–1265.681270	–1265.655952	162.2	–1265.730290	–166.5	1
**QMf**	–459.904465	–459.761013	–459.751232	91.7	–459.794665	86.3	0
**3g**	–1380.612301	–1380.162018	–1380.134387	173.4	–1380.212991	17.6	0
**3g_TS**	–1380.585454	–1380.137328	–1380.109902	169.4	–1380.188231	–195.8	1
**QMg**	–574.394594	–574.217764	–574.205498	103.8	–574.254295	56.8	0
**3h**	–1380.602174	–1380.152342	–1380.124440	175.7	–1380.203653	22.2	0
**3h_TS**	–1380.572061	–1380.124609	–1380.096790	174.2	–1380.175858	–244.7	1

**QMh**	–574.383492	–574.207016	–574.194660	104.2	–574.243586	54.1	0
(*R*,*R*)-**3i**	–1190.933125	–1190.520890	–1190.496839	156.6	–1190.568563	34.2	0
(*R*,*R*)-**3i_TS**	–1190.903085	–1190.493739	–1190.469499	158.0	–1190.541699	–244.7	1
(*R*,*S*)-**3i**	–1190.931699	–1190.519284	–1190.495253	156.7	–1190.566919	33.6	0
(*R*,*S*)-**3i_TS**	–1190.904175	–1190.494939	–1190.470634	157.7	–1190.543051	–211.0	1
**QMi**	–384.715082	–384.577017	–384.567972	88.3	–384.609667	74.6	0
**4xH_2O**	–305.640683	–305.544130	–305.531796	102.7	–305.578540	30.9	0
**H_3O^+_3xH_2O**	–306.074128	–305.965778	–305.953928	102.6	–306.000299	43.8	0
**2**	–806.646064	–806.363684	–806.347027	122.2	–806.404398	63.4	0
**2_anion**	–806.174551	–805.905172	–805.889234	119.3	–805.945215	62.0	0

[a]Energy values calculated at the PCM(H_2O)/M06-2X/6-31+G(d,p) level. 1 Hartree = 627.51 kcal mol^{-1}. Thermal corrections at 298.15 K.

5.1. Cartesian coordinates of the lowest energy structures calculated at the PCM(H2O)/M06-2X/6-31+G(d,p) level

Structure **3a**

C	3.539210	-0.724298	2.236270
C	3.425609	-0.192181	0.953226
C	2.252509	-0.364440	0.220201
C	1.185167	-1.079191	0.778458
C	1.296294	-1.601131	2.073035
C	2.469665	-1.422819	2.799025
C	2.097762	0.305827	-1.120391
C	-0.038438	-1.291058	-0.017667
C	-0.150353	-0.937585	-1.332349
C	0.957520	-0.263658	-1.969101
C	-1.361030	-1.294681	-2.147407
H	-1.039044	-1.662954	-3.125697
C	-2.180778	-2.355487	-1.417830
C	-2.333784	-2.014913	0.064114
H	4.458194	-0.589800	2.797695
H	4.249170	0.361637	0.512801
H	0.462482	-2.146507	2.499897
H	2.551387	-1.830684	3.801139
H	-3.175287	-2.459749	-1.861162
O	1.006878	-0.051188	-3.182537
O	3.310112	0.192899	-1.839875
H	3.081606	0.337697	-2.772626
C	1.795096	1.828022	-0.919779
H	2.634088	2.227461	-0.340428
H	1.831820	2.274617	-1.919794
C	0.478809	2.153935	-0.261730
C	-0.634959	2.493612	-1.035664
C	0.322889	2.102157	1.128845
C	-1.868793	2.772267	-0.451521
H	-0.540082	2.541010	-2.118231
C	-0.904781	2.367560	1.728859
H	1.175281	1.847270	1.754976
C	-2.003411	2.702119	0.935621
H	-2.723582	3.033270	-1.069867
H	-1.023690	2.323441	2.806736
O	-1.006046	-1.912865	0.662551
H	-1.679975	-3.327809	-1.493895
H	-1.961068	-0.394485	-2.339172
C	-3.013098	-3.138152	0.828573
H	-2.478285	-4.080459	0.681820
H	-3.040406	-2.908287	1.897247
H	-4.039426	-3.257379	0.471232
C	-3.035208	-0.679244	0.288452
H	-3.138316	-0.484529	1.359890
H	-2.469899	0.144388	-0.156726
H	-4.031660	-0.702888	-0.162852
O	-3.186725	2.944410	1.570117
H	-3.872454	3.168730	0.927536

Structure **3a_isomerB**

C	-2.344616	0.538051	2.990277
C	-1.953417	1.520073	2.086563
C	-0.841217	1.311210	1.269863
C	-0.081842	0.131236	1.371451
C	-0.472411	-0.842580	2.295587
C	-1.602477	-0.642188	3.086968
C	-0.444608	2.330742	0.281040
C	1.118812	-0.038230	0.531174
C	1.418253	0.788423	-0.486845
C	0.471400	1.897466	-0.861813
C	2.615260	0.550980	-1.365567
H	3.385809	1.304572	-1.158806
C	3.155531	-0.861041	-1.147045
C	3.202015	-1.214789	0.340621
H	-3.220318	0.685439	3.613213
H	-2.512500	2.445010	1.984121
H	0.110755	-1.751392	2.389346
H	-1.903438	-1.411937	3.790725
H	2.506660	-1.590625	-1.646309
O	1.221443	3.013856	-1.299983
O	-0.861852	3.479373	0.296272
C	-0.468345	1.452519	-2.041418
H	-1.091922	2.320378	-2.286292
H	0.194907	1.272167	-2.893343
C	-1.321072	0.242895	-1.763037
C	-2.618029	0.369689	-1.252886
C	-0.810994	-1.045766	-1.943529
C	-3.374697	-0.745907	-0.908834
H	-3.041512	1.361402	-1.106329
C	-1.551948	-2.176051	-1.605009
H	0.188979	-1.172569	-2.351606
C	-2.835399	-2.022702	-1.077445
H	-4.377640	-0.642344	-0.507314
H	-1.136016	-3.169277	-1.750980
O	1.863446	-1.121822	0.887555
H	4.157920	-0.966564	-1.572633
H	2.339543	0.689565	-2.417652
C	4.108076	-0.271764	1.130386
H	4.149880	-0.585529	2.176982
H	5.120852	-0.294269	0.717022
H	3.741905	0.757738	1.092385
C	3.608911	-2.664611	0.548112
H	3.560332	-2.923360	1.609654
H	2.944470	-3.331436	-0.008685
H	4.633812	-2.817325	0.198817

H	0.652860	3.792679	-1.190703
O	-3.613605	-3.084430	-0.718707
H	-3.143894	-3.914086	-0.874388

Structure **3a**$_{\mathbf{PhO-}}$

C	-3.541864	-0.913420	-2.161062
C	-3.423928	-0.318110	-0.906467
C	-2.234801	-0.419491	-0.185787
C	-1.155040	-1.127140	-0.728944
C	-1.270544	-1.712757	-1.995619
C	-2.460181	-1.604890	-2.709221
C	-2.080375	0.315909	1.118971
C	0.084879	-1.268088	0.057423
C	0.204976	-0.850255	1.351472
C	-0.914984	-0.182014	1.973993
C	1.439688	-1.128465	2.160384
H	1.147807	-1.445930	3.165588
C	2.272197	-2.209107	1.476305
C	2.395769	-1.946676	-0.024191
H	-4.473641	-0.833655	-2.711994
H	-4.257063	0.230725	-0.477901
H	-0.427079	-2.252289	-2.410972
H	-2.545034	-2.062368	-3.689516
H	3.275814	-2.267351	1.907815
O	-0.954853	0.076543	3.179755
O	-3.281057	0.199685	1.862332
H	-3.042645	0.403735	2.781161
C	-1.819856	1.836039	0.841794
H	-2.680719	2.176644	0.254550
H	-1.873036	2.321642	1.824248
C	-0.518797	2.180404	0.168113
C	0.604014	2.554724	0.921223
C	-0.351840	2.101191	-1.221921
C	1.829125	2.836092	0.330315
H	0.510491	2.624134	2.005671
C	0.868181	2.372515	-1.832831
H	-1.202828	1.813654	-1.840746
C	2.029862	2.750223	-1.085061
H	2.678334	3.123540	0.947153
H	0.962313	2.301362	-2.914726
O	1.058619	-1.900097	-0.607148
H	1.794637	-3.186545	1.613867
H	2.018774	-0.202849	2.283539
C	3.082558	-3.100187	-0.735966
H	2.568523	-4.042313	-0.526452
H	3.086915	-2.931139	-1.816337
H	4.117257	-3.180510	-0.391951
C	3.073640	-0.615944	-0.332953
H	3.168021	-0.487664	-1.415270
H	2.502135	0.230165	0.060870
H	4.074393	-0.599942	0.109953
O	3.167360	2.994322	-1.639735

Structure **3a_TS**

C	3.077161	1.277359	2.228241
C	3.251581	0.335634	1.236811
C	2.139276	-0.368955	0.703268
C	0.835808	-0.066393	1.195987
C	0.689777	0.894378	2.221707
C	1.785968	1.558912	2.732825
C	2.261692	-1.304583	-0.344811
C	-0.288381	-0.767620	0.638100
C	-0.144634	-1.737671	-0.328492
C	1.164887	-2.059585	-0.835764
C	-1.336942	-2.440854	-0.915822
C	-2.627613	-1.707336	-0.559598
C	-2.631301	-1.259750	0.903015
H	3.938337	1.804867	2.628458
H	4.242318	0.117072	0.849791
H	-0.306739	1.109536	2.593584
H	1.659466	2.297893	3.517797
O	1.373437	-2.949352	-1.738383
O	3.498996	-1.643230	-0.831698
H	3.302084	-2.348442	-1.478298
C	1.691303	0.446048	-2.092632
H	2.720267	0.691017	-1.847554
H	1.528114	-0.378197	-2.782006
C	0.664628	1.271084	-1.720171
C	0.889733	2.417667	-0.870148
C	-0.698097	1.007741	-2.123190
C	-0.122580	3.237393	-0.489913
H	1.906614	2.602629	-0.527314
C	-1.722709	1.815988	-1.753244
H	-0.882898	0.130253	-2.741421
C	-1.500300	2.988353	-0.908225
H	0.053030	4.095996	0.151738
H	-2.745741	1.615424	-2.059520
O	-1.501055	-0.389950	1.139076
C	-2.537612	-2.441964	1.867069
H	-2.582794	-2.085765	2.900242
H	-3.369413	-3.132581	1.697772
H	-1.600526	-2.989243	1.730775
C	-3.849499	-0.401517	1.206363
H	-3.805964	-0.028441	2.233859
H	-3.889389	0.452352	0.522843
H	-4.762992	-0.991161	1.087534
H	-1.371870	-3.481881	-0.565949
H	-1.214296	-2.499023	-2.003298
H	-2.728945	-0.810640	-1.181006
H	-3.503252	-2.338700	-0.742568
O	-2.438666	3.732666	-0.560709

Structure **QMa**

C	2.745571	0.000000	-0.000181

H	3.307790	0.928816	-0.000236
H	3.307791	-0.928815	-0.000237
C	1.398035	-0.000000	-0.000063
C	0.639313	1.249834	-0.000015
C	0.639313	-1.249835	-0.000016
C	-0.706412	1.257026	0.000059
H	1.200167	2.180850	-0.000041
C	-0.706412	-1.257026	0.000058
H	1.200166	-2.180851	-0.000043
C	-1.475829	0.000000	0.000109
H	-1.276781	2.180443	0.000087
H	-1.276783	-2.180442	0.000087
O	-2.707979	-0.000000	0.000084

Structure **3a**$_{Zw1}$

C	-3.448256	-0.405108	-2.415062
C	-3.403097	-0.116900	-1.052721
C	-2.236794	-0.353759	-0.331308
C	-1.114404	-0.881351	-0.982900
C	-1.156712	-1.156585	-2.356645
C	-2.323431	-0.915910	-3.069196
C	-2.114006	0.084915	1.100328
C	0.085656	-1.174537	-0.197799
C	0.110937	-1.118750	1.209290
C	-1.018300	-0.633147	1.827406
C	1.290476	-1.635864	1.988427
H	0.934193	-2.188835	2.861175
C	2.135296	-2.538631	1.094409
C	2.402468	-1.894840	-0.261955
H	-4.363544	-0.228392	-2.970232
H	-4.277148	0.287433	-0.552216
H	-0.278919	-1.558114	-2.849774
H	-2.362248	-1.129955	-4.131539
H	3.096158	-2.761047	1.565565
O	-1.103167	-0.662168	3.136984
O	-3.294803	-0.104333	1.852654
H	-3.800686	0.719099	1.899597
C	-1.737918	1.635108	1.128224
H	-2.584268	2.113207	0.619100
H	-1.770805	1.920378	2.186659
C	-0.436723	2.045926	0.517077
C	-0.286807	2.243204	-0.866103
C	0.695303	2.263044	1.321536
C	0.924814	2.624556	-1.423370
H	-1.146836	2.091595	-1.519501
C	1.910052	2.659998	0.786660
H	0.611492	2.122339	2.399836
C	2.094396	2.854741	-0.624074
H	1.008446	2.767971	-2.498293
H	2.766401	2.824684	1.436878
O	1.101447	-1.586516	-0.897561
H	1.617348	-3.490529	0.931369
H	1.879589	-0.791321	2.366207
C	3.063546	-2.861358	-1.227485
H	3.166942	-2.402999	-2.214124
H	4.059177	-3.114696	-0.854408
H	2.477987	-3.779718	-1.318093
C	3.154997	-0.575297	-0.168195
H	3.326443	-0.168528	-1.168312
H	2.597758	0.168167	0.408928
H	4.122116	-0.745307	0.313533
O	3.218680	3.208172	-1.128505
H	-2.005814	-0.393407	3.399784

Structure **3a**$_{Zw1}$_TS

C	3.184378	1.387809	2.215077
C	3.321811	0.557202	1.115523
C	2.202637	-0.124912	0.612970
C	0.944832	0.049816	1.221123
C	0.819674	0.902394	2.336738
C	1.929359	1.563887	2.826416
C	2.265733	-0.903954	-0.610121
C	-0.191446	-0.675759	0.694544
C	-0.057756	-1.667818	-0.282385
C	1.202474	-1.826874	-0.851079
C	-1.245450	-2.475595	-0.743993
C	-2.543492	-1.769390	-0.355123
C	-2.505726	-1.255995	1.080575
H	4.052422	1.906908	2.608518
H	4.284746	0.423982	0.634327
H	-0.153428	1.031882	2.796648
H	1.834118	2.219270	3.685598
O	1.477921	-2.735390	-1.797264
O	3.530094	-1.241190	-1.034157
H	3.466835	-1.869114	-1.768762
C	1.725783	0.451106	-1.930354
H	2.668939	0.977753	-1.795063
H	1.719554	-0.209027	-2.798352
C	0.532282	1.176843	-1.655353
C	0.542462	2.327994	-0.813565
C	-0.727096	0.781669	-2.192361
C	-0.596814	3.033171	-0.532342
H	1.491761	2.640605	-0.378140
C	-1.878670	1.478612	-1.933767
H	-0.762466	-0.096943	-2.837319
C	-1.884748	2.649098	-1.078833
H	-0.565941	3.908102	0.111427
H	-2.827174	1.169182	-2.365136
O	-1.356080	-0.364859	1.246439
C	-2.350133	-2.375335	2.104907
H	-2.336640	-1.959119	3.115422
H	-3.190547	-3.069998	2.021559
H	-1.422581	-2.934154	1.949065
C	-3.707841	-0.377987	1.380477

H	-3.633955	0.037012	2.389213
H	-3.764509	0.443621	0.660321
H	-4.622780	-0.972324	1.312539
O	-2.939415	3.294501	-0.831041
H	0.706534	-3.270099	-2.035746
H	-3.393620	-2.448465	-0.462438
H	-2.708238	-0.914710	-1.017592
H	-1.208600	-3.485456	-0.317387
H	-1.228098	-2.589301	-1.834952

Structure **3b**

C	3.795730	-0.557884	-2.096906
C	3.528757	-0.818773	-0.753935
C	2.456201	-0.199044	-0.114825
C	1.645285	0.692042	-0.829246
C	1.906734	0.942322	-2.181826
C	2.978340	0.318106	-2.812956
C	2.107588	-0.569885	1.302556
C	0.538893	1.377333	-0.134721
C	0.330611	1.293142	1.212205
C	1.190650	0.438338	1.997209
C	-0.719421	2.115772	1.903443
H	-0.304479	2.523613	2.829710
C	-1.183527	3.240369	0.981981
C	-1.434274	2.727101	-0.435526
H	4.636421	-1.040830	-2.584705
H	4.153428	-1.507246	-0.192910
H	1.269651	1.627916	-2.728714
H	3.177786	0.517109	-3.860822
H	-2.100466	3.704683	1.356621
O	1.177648	0.423992	3.230530
O	3.298139	-0.706801	2.055817
H	3.033027	-0.625995	2.986532
C	1.360512	-1.946798	1.320859
H	2.049993	-2.661883	0.858586
H	1.260734	-2.213163	2.379738
C	0.011247	-1.979131	0.654820
C	-1.160176	-1.811633	1.404772
C	-0.124001	-2.125209	-0.731680
C	-2.391494	-1.798091	0.778676
C	-1.377972	-2.098739	-1.313335
C	-2.613477	-1.938441	-0.621861
O	-0.201552	2.136978	-0.948495
H	-0.413596	4.019419	0.931627
H	-1.561083	1.474090	2.198768
C	-1.741037	3.864565	-1.394873
H	-0.945398	4.614165	-1.368762
H	-1.837385	3.484774	-2.415749
H	-2.682685	4.341704	-1.110360
C	-2.511142	1.648425	-0.484837
H	-2.667828	1.321324	-1.516793
H	-2.228198	0.774712	0.109487
H	-3.452706	2.047444	-0.095706
O	-3.765296	-1.920072	-1.180885
F	-3.503140	-1.624389	1.552771
F	-1.457981	-2.244510	-2.669023
H	-1.120149	-1.683748	2.484461
H	0.747355	-2.254092	-1.370442

Structure **3b_TS**

C	-2.381118	1.418852	2.200618
C	-1.614510	2.270748	1.433494
C	-0.374310	1.842057	0.890143
C	0.067749	0.509513	1.157437
C	-0.749279	-0.346497	1.923823
C	-1.957588	0.092649	2.434550
C	0.393334	2.644614	0.028062
C	1.352317	0.097111	0.654973
C	2.147399	0.934241	-0.092319
C	1.671996	2.251248	-0.453330
C	3.545176	0.540534	-0.482352
H	4.204342	1.404300	-0.348964
C	4.014431	-0.631259	0.376382
C	2.942054	-1.718793	0.463148
H	-3.327090	1.762733	2.608839
H	-1.949374	3.283483	1.229493
H	-0.414380	-1.363315	2.103577
H	-2.578651	-0.581341	3.016681
H	4.936217	-1.069638	-0.019185
O	2.350496	3.071050	-1.164922
O	-0.000297	3.923984	-0.266805
H	0.739115	4.276047	-0.798729
C	-0.450524	1.419312	-2.150648
H	-0.861456	2.423096	-2.193269
H	0.527632	1.257178	-2.592231
C	-1.205131	0.365631	-1.711017
C	-0.680961	-0.978674	-1.696126
C	-2.512268	0.580865	-1.138956
C	-1.393401	-1.981682	-1.138933
C	-3.187163	-0.446482	-0.581418
C	-2.698899	-1.817826	-0.513057
O	1.735618	-1.158969	1.032077
H	4.224804	-0.280493	1.394309
H	3.596506	0.290974	-1.550928
C	3.348471	-2.819018	1.432379
H	3.553512	-2.398214	2.420837
H	2.547592	-3.558742	1.523816
H	4.249071	-3.323280	1.071126
C	2.617774	-2.304147	-0.909457
H	1.797307	-3.022606	-0.826911
H	2.328706	-1.522930	-1.616399
H	3.497495	-2.815814	-1.311654
O	-3.336099	-2.749871	-0.000593
F	-0.905604	-3.242999	-1.123706

F	-4.404162	-0.244185	-0.031877
H	0.290139	-1.180955	-2.134562
H	-2.944631	1.576642	-1.138034

Structure **QMb**

C	3.060808	-0.000000	-0.000008
H	3.621151	0.929180	0.000006
H	3.621151	-0.929180	0.000016
C	1.712727	-0.000000	0.000025
C	0.964844	1.252928	0.000011
C	0.964844	-1.252929	0.000022
C	-0.374460	1.234046	0.000048
H	1.500468	2.196836	-0.000066
C	-0.374460	-1.234046	0.000059
H	1.500468	-2.196837	-0.000046
C	-1.180863	0.000000	0.000241
O	-2.402520	0.000000	-0.000112
F	-1.092428	2.366030	-0.000089
F	-1.092429	-2.366030	-0.000067

Structure **3c**

C	3.873383	-0.327974	-1.900452
C	3.612017	-0.232369	-0.535039
C	2.411492	0.313268	-0.086688
C	1.467702	0.776093	-1.013246
C	1.723700	0.658647	-2.384779
C	2.922369	0.105825	-2.825452
C	2.076513	0.314927	1.380997
C	0.246301	1.448031	-0.525979
C	0.033714	1.760268	0.788140
C	1.021311	1.356074	1.763002
C	-1.125488	2.617002	1.214510
H	-0.781074	3.334951	1.964278
C	-1.698637	3.344500	0.001236
C	-1.876170	2.396678	-1.184451
H	4.814381	-0.746826	-2.242720
H	4.338823	-0.581806	0.191747
H	0.986787	1.013694	-3.096114
H	3.118218	0.021742	-3.889420
H	-2.665275	3.800414	0.234393
O	1.032334	1.764535	2.926024
O	3.243218	0.542154	2.142705
H	2.940104	0.933491	2.979110
C	1.517285	-1.095145	1.787495
H	2.337706	-1.795347	1.604917
H	1.341703	-1.053135	2.867576
C	0.275256	-1.555685	1.083248
C	-1.004811	-1.284166	1.567886
C	0.290739	-2.263655	-0.118865
C	-2.161331	-1.683435	0.932131
C	-0.854527	-2.660177	-0.781563
C	-2.173581	-2.395740	-0.305519
O	-0.585146	1.797338	-1.511040
H	-1.017149	4.147934	-0.302561
H	-1.891172	2.001987	1.705567
C	-2.275953	3.147224	-2.443881
H	-1.553823	3.937897	-2.664879
H	-2.323899	2.462426	-3.295027
H	-3.261383	3.599821	-2.304905
C	-2.845924	1.259525	-0.883551
H	-2.937335	0.603447	-1.753873
H	-2.502124	0.659374	-0.037583
H	-3.832138	1.668254	-0.644562
O	-3.234994	-2.749652	-0.909729
F	-3.352841	-1.352011	1.487382
F	-1.125824	-0.578365	2.716790
F	1.480041	-2.596251	-0.668122
F	-0.722245	-3.344666	-1.944351

Structure **QMc**

C	0.000000	2.770808	0.000422
H	0.931640	3.324282	0.000501
H	-0.931640	3.324282	0.000490
C	0.000000	1.428277	0.000217
C	1.233186	0.650166	0.000107
C	-1.233186	0.650166	0.000094
C	1.242443	-0.690863	-0.000105
C	-1.242443	-0.690863	-0.000119
C	0.000000	-1.481848	-0.000267
O	-0.000000	-2.700894	-0.000420
F	-2.375289	1.331984	0.000206
F	2.375289	1.331984	0.000232
F	2.382544	-1.379566	-0.000190
F	-2.382544	-1.379566	-0.000216

Structure **3d**

C	-3.584837	-1.229855	-1.932979
C	-3.416558	-0.705573	-0.653216
C	-2.163198	-0.723936	-0.044931
C	-1.070550	-1.281184	-0.721405
C	-1.238327	-1.789899	-2.015273
C	-2.491474	-1.761535	-2.619058
C	-1.958044	-0.062810	1.292017
C	0.231961	-1.377865	-0.033464
C	0.412951	-1.056711	1.282392
C	-0.720458	-0.567282	2.035946
C	1.715675	-1.320603	1.983309
H	1.511689	-1.717611	2.981765
C	2.544599	-2.311225	1.170489
C	2.564805	-1.938165	-0.311585
H	-4.565947	-1.218546	-2.397196
H	-4.258524	-0.277519	-0.117977
H	-0.386706	-2.214599	-2.534412
H	-2.617176	-2.160909	-3.620182

H	3.575069	-2.357156	1.534921
O	-0.728964	-0.481246	3.265762
O	-3.097508	-0.262492	2.103946
H	-2.781507	-0.195639	3.020452
C	-1.785355	1.485198	1.088400
H	-2.710663	1.818155	0.608113
H	-1.753930	1.914124	2.095610
C	-0.590490	1.938245	0.303931
C	0.631984	2.261644	0.901051
C	-0.563233	2.066196	-1.086984
C	1.765559	2.674967	0.240548
C	0.529195	2.453834	-1.833782
C	1.771534	2.778082	-1.192385
O	1.193442	-1.889270	-0.808647
H	2.114387	-3.315464	1.264268
H	2.260253	-0.377777	2.129190
C	3.234976	-3.016839	-1.146534
H	2.758819	-3.986349	-0.976371
H	3.168012	-2.770277	-2.209767
H	4.290785	-3.090921	-0.872203
C	3.187095	-0.569667	-0.565149
H	3.206794	-0.361428	-1.638860
H	2.618123	0.225300	-0.074981
H	4.213262	-0.552591	-0.185412
O	2.810176	3.131970	-1.847046
F	0.695737	2.151719	2.259022
F	-1.720200	1.799100	-1.754114
H	0.446506	2.521396	-2.913583
H	2.666882	2.898623	0.802307

Structure **QMd**

C	2.459886	-0.000001	0.000424
H	3.014173	-0.931268	0.000574
H	3.014174	0.931266	0.000564
C	1.117932	-0.000000	0.000190
C	0.322531	-1.226053	0.000041
C	0.322531	1.226053	0.000028
C	-1.014946	-1.261388	-0.000102
C	-1.014945	1.261388	-0.000115
C	-1.776599	-0.000000	0.000005
H	-1.559865	-2.197831	-0.000254
O	-3.004548	0.000001	-0.000548
H	-1.559866	2.197830	-0.000275
F	1.034969	2.359302	0.000043
F	1.034967	-2.359302	0.000063

Structure **3f**

C	-3.935970	-0.459880	-1.773599
C	-3.603210	-0.134103	-0.459124
C	-2.334582	-0.423597	0.038398
C	-1.391542	-1.049645	-0.787923
C	-1.722662	-1.362521	-2.111586
C	-2.990777	-1.065255	-2.602979
C	-1.927292	0.050610	1.406390
C	-0.076212	-1.416443	-0.228264
C	0.236024	-1.296540	1.095347
C	-0.739679	-0.711861	1.987484
C	1.503174	-1.868526	1.663758
H	1.273144	-2.379227	2.603503
C	2.125365	-2.838408	0.663547
C	2.124472	-2.249410	-0.746699
H	-4.928665	-0.236245	-2.151458
H	-4.327645	0.350398	0.188498
H	-0.985176	-1.841112	-2.745912
H	-3.242854	-1.310796	-3.629518
H	3.153332	-3.089240	0.941361
O	-0.645396	-0.762836	3.216363
O	-3.017848	-0.057213	2.301988
H	-2.621185	-0.146206	3.184560
C	-1.533212	1.571176	1.318101
H	-2.482882	2.072168	1.098635
H	-1.227577	1.857970	2.330797
C	-0.503453	1.979589	0.304778
C	0.870548	2.027564	0.613859
C	-0.863138	2.365345	-0.992115
C	1.818007	2.460217	-0.307798
C	0.060664	2.775934	-1.944350
C	1.457513	2.844250	-1.641398
O	0.748154	-1.958700	-1.133789
H	1.549486	-3.771353	0.644006
H	2.199803	-1.058536	1.916668
C	2.607169	-3.259184	-1.773998
H	2.020729	-4.180105	-1.712790
H	2.516822	-2.847507	-2.783000
H	3.657713	-3.498571	-1.588227
C	2.909165	-0.944673	-0.830414
H	2.925832	-0.579318	-1.861430
H	2.453576	-0.170205	-0.207373
H	3.938737	-1.106680	-0.496555
O	2.339535	3.222804	-2.498561
H	-0.266377	3.065252	-2.939962
H	2.872493	2.492498	-0.054563
O	1.196716	1.611054	1.880258
C	2.543914	1.729006	2.296568
H	2.575687	1.367316	3.324501
H	3.211334	1.118499	1.676467
H	2.879029	2.771777	2.261428
H	-1.920349	2.336475	-1.257612

Structure **3f_TS**

C	3.843260	1.309094	1.534915
C	3.709944	0.461582	0.455204
C	2.500899	-0.250643	0.241080
C	1.422571	-0.063584	1.151397

C	1.591525	0.801054	2.255342
C	2.777581	1.481227	2.447956
C	2.291885	-1.074940	-0.887811
C	0.196482	-0.782123	0.919910
C	0.042787	-1.665641	-0.124395
C	1.124761	-1.871309	-1.054829
C	-1.251744	-2.396995	-0.345990
C	-2.369855	-1.746703	0.463017
C	-1.914333	-1.425817	1.886818
H	4.775186	1.845926	1.687382
H	4.526667	0.327392	-0.247635
H	0.764163	0.931554	2.945072
H	2.893045	2.146979	3.297415
O	1.070164	-2.693100	-2.035623
O	3.321235	-1.317431	-1.761316
H	2.953675	-1.991479	-2.365168
C	1.237443	0.735920	-2.139553
H	2.257591	1.099225	-2.071423
H	1.016625	0.007263	-2.912475
C	0.233197	1.400179	-1.482525
C	0.519474	2.453237	-0.539352
C	-1.168227	1.074296	-1.690148
C	-0.455176	3.144265	0.102453
C	-2.165535	1.770112	-1.063391
C	-1.864860	2.838854	-0.126375
H	-0.222991	3.933943	0.810373
H	-3.213527	1.544743	-1.224146
O	-0.790244	-0.518149	1.826650
C	-1.483931	-2.674764	2.654727
H	-1.212854	-2.407757	3.680153
H	-2.304523	-3.398127	2.686251
H	-0.620599	-3.151446	2.182029
C	-2.989647	-0.661675	2.643164
H	-2.622980	-0.365346	3.630311
H	-3.275314	0.237616	2.088646
H	-3.874650	-1.290810	2.774002
H	-1.143307	-3.456687	-0.076371
H	-1.486679	-2.380540	-1.415508
H	-2.658810	-0.797142	-0.002930
H	-3.258167	-2.385734	0.501762
O	-2.774102	3.468787	0.457685
O	-1.378212	0.066401	-2.561146
C	-2.714237	-0.227659	-2.945500
H	-3.190638	0.653097	-3.387727
H	-2.643042	-1.023107	-3.685683
H	-3.304192	-0.573890	-2.090932
H	1.569005	2.674462	-0.350078

Structure **QMf**

C	1.210559	2.320558	-0.000427
H	0.897213	3.359776	-0.000519
H	2.272937	2.105740	-0.000609
C	0.296074	1.335373	-0.000109
C	-1.136660	1.629464	0.000054
C	0.688944	-0.087344	-0.000015
C	-2.063268	0.658153	0.000221
H	-1.424089	2.677264	0.000010
C	-0.241195	-1.075834	0.000140
C	-1.668644	-0.765017	0.000327
H	-3.126882	0.872826	0.000302
O	-2.522551	-1.656650	0.000279
H	0.023609	-2.125844	0.000157
O	2.014880	-0.275820	-0.000120
C	2.499466	-1.615781	-0.000189
H	3.584563	-1.539041	-0.000352
H	2.161322	-2.144199	0.895859
H	2.161035	-2.144197	-0.896133

Structure **3g**

C	-3.841732	-1.134606	-1.372773
C	-3.325807	-1.295857	-0.088129
C	-1.951775	-1.236524	0.125716
C	-1.084205	-1.035099	-0.955959
C	-1.606137	-0.851824	-2.241592
C	-2.982465	-0.895614	-2.446598
C	-1.375605	-1.249262	1.511512
C	0.371507	-1.111276	-0.725616
C	0.919613	-1.556630	0.443341
C	0.039167	-1.814245	1.558634
C	2.402991	-1.719444	0.610409
H	2.697042	-2.773077	0.520681
C	3.112452	-0.864431	-0.435871
C	2.518797	-1.080614	-1.827591
H	-4.914044	-1.185199	-1.535472
H	-3.986320	-1.457521	0.757882
H	-0.929365	-0.690392	-3.073419
H	-3.383777	-0.753456	-3.445096
H	2.993511	0.196149	-0.181843
O	0.396191	-2.392990	2.589240
O	-2.199967	-1.980477	2.393350
H	-1.597955	-2.359466	3.055846
C	-1.313863	0.243744	2.042059
H	-2.366368	0.530522	2.124438
H	-0.894895	0.172670	3.051332
C	-0.561261	1.254555	1.229839
C	0.804794	1.513638	1.450826
C	-1.172838	1.995024	0.198159
C	1.530355	2.436819	0.706383
C	-0.477308	2.916520	-0.578815
C	0.913352	3.172230	-0.355844
O	1.092682	-0.776501	-1.802866
H	4.183586	-1.085971	-0.472990
H	2.680955	-1.406377	1.621412
C	2.659683	-2.522732	-2.306884

H	2.120340	-3.212016	-1.651068
H	2.257104	-2.622191	-3.318482
H	3.715399	-2.809266	-2.318841
C	3.100612	-0.099775	-2.831755
H	4.171225	-0.285664	-2.953351
H	2.613184	-0.215268	-3.803843
H	2.957962	0.927246	-2.482793
O	1.570211	4.019206	-1.065077
H	-0.967878	3.474575	-1.367570
H	2.582047	2.618694	0.896102
O	-2.506817	1.745768	0.025966
O	1.368477	0.767945	2.454413
C	-3.171291	2.369670	-1.055046
H	-4.187774	1.975025	-1.051056
H	-3.198225	3.458639	-0.932141
H	-2.690564	2.120413	-2.007513
C	2.699366	1.058507	2.837993
H	3.404836	0.867918	2.020858
H	2.798057	2.100811	3.160858
H	2.925273	0.392948	3.671479

Structure **3g_TS**

C	3.871655	-0.618304	1.506323
C	3.365066	-1.331794	0.441008
C	1.965937	-1.408595	0.228631
C	1.089789	-0.750964	1.134289
C	1.636190	-0.015401	2.208511
C	3.003015	0.054025	2.394292
C	1.397319	-2.067266	-0.890253
C	-0.331600	-0.898005	0.945440
C	-0.870152	-1.684321	-0.047942
C	-0.000002	-2.335194	-0.992804
C	-2.356563	-1.848978	-0.197444
C	-3.091514	-0.773683	0.597503
C	-2.482913	-0.593944	1.988668
H	4.945641	-0.568939	1.662874
H	4.027800	-1.842134	-0.251386
H	0.961137	0.491719	2.890410
H	3.410843	0.623887	3.223775
O	-0.413357	-3.096704	-1.933508
O	2.205470	-2.754519	-1.759581
H	1.573319	-3.241017	-2.323372
C	1.167816	-0.074048	-2.134617
H	2.250214	-0.107920	-2.107627
H	0.662377	-0.674371	-2.883243
C	0.502551	0.962426	-1.521418
C	1.181306	1.913348	-0.658762
C	-0.920737	1.172448	-1.715040
C	0.522877	2.956115	-0.065957
C	-1.589264	2.217786	-1.143301
C	-0.898565	3.152494	-0.273749
H	1.027128	3.670752	0.572147
H	-2.648639	2.383498	-1.298321
O	-1.092464	-0.219415	1.852821
C	-2.553921	-1.872147	2.822941
H	-2.150632	-1.688202	3.822864
H	-3.592986	-2.201808	2.919034
H	-1.976723	-2.677735	2.360296
C	-3.128322	0.573637	2.718441
H	-2.632535	0.742880	3.678801
H	-3.051293	1.484544	2.116592
H	-4.185238	0.362092	2.903732
H	-2.663813	-2.851172	0.132279
H	-2.611495	-1.790819	-1.260856
H	-3.007831	0.190293	0.082169
H	-4.156081	-1.010129	0.697342
O	-1.510912	4.102283	0.271693
O	-1.496885	0.263121	-2.530349
O	2.502956	1.686592	-0.530719
C	-2.847874	0.467200	-2.917208
H	-2.963938	1.433691	-3.418129
H	-3.085357	-0.340708	-3.607890
H	-3.519318	0.419655	-2.053802
C	3.267987	2.610872	0.227537
H	2.900784	2.667515	1.256139
H	4.286226	2.223932	0.228136
H	3.241446	3.603389	-0.235358

Structure **QMg**

C	-0.000003	-2.375149	0.000048
H	-0.932573	-2.926568	0.000045
H	0.932565	-2.926574	0.000043
C	-0.000000	-1.033903	0.000056
C	-1.257414	-0.259531	0.000024
C	1.257414	-0.259531	0.000024
C	-1.258923	1.093781	0.000026
C	1.258923	1.093781	0.000026
C	-0.000000	1.840828	0.000179
H	-2.168931	1.680098	-0.000037
O	0.000000	3.077769	-0.000121
H	2.168931	1.680099	-0.000038
O	2.350600	-1.035367	-0.000010
C	3.620974	-0.390877	-0.000072
H	4.359437	-1.189695	-0.000110
H	3.736075	0.226226	-0.895918
H	3.736167	0.226222	0.895765
O	-2.350599	-1.035367	-0.000008
C	-3.620974	-0.390875	-0.000052
H	-3.736085	0.226227	-0.895898
H	-4.359436	-1.189694	-0.000080
H	-3.736152	0.226224	0.895786

Structure **3h**

C	4.065732	1.925376	0.140333

C	3.674042	0.910987	-0.732056
C	2.614696	0.067195	-0.402836
C	1.945353	0.240439	0.815565
C	2.331965	1.267291	1.684968
C	3.387461	2.108686	1.346502
C	2.115638	-0.943758	-1.400771
C	0.857624	-0.686703	1.179599
C	0.545896	-1.799670	0.452712
C	1.251552	-2.043740	-0.783621
C	-0.459253	-2.804870	0.937790
H	-0.069978	-3.813094	0.768703
C	-0.741936	-2.578653	2.420359
C	-0.947701	-1.094579	2.721816
H	4.894706	2.574682	-0.122868
H	4.188448	0.770411	-1.677892
H	1.803310	1.397816	2.622533
H	3.683571	2.903005	2.023956
H	-1.629631	-3.131011	2.742299
O	1.139972	-3.093419	-1.422935
O	3.219716	-1.539350	-2.058110
H	2.886912	-2.372601	-2.429489
C	1.234675	-0.224716	-2.481534
H	1.898959	0.510784	-2.949583
H	1.005827	-0.993500	-3.229983
C	-0.032880	0.418016	-1.986926
C	-0.028983	1.676697	-1.390086
C	-1.255246	-0.276683	-2.048985
C	-1.198464	2.229188	-0.871039
C	-2.423613	0.275276	-1.538164
C	-2.464585	1.576193	-0.919259
O	0.253266	-0.364144	2.328243
H	0.105561	-2.934829	3.018068
H	-1.380936	-2.726872	0.344837
C	-1.076795	-0.839943	4.214054
H	-0.215812	-1.248518	4.750272
H	-1.140816	0.233876	4.411291
H	-1.984923	-1.318026	4.591108
C	-2.120557	-0.499857	1.949782
H	-3.040356	-1.034766	2.206062
H	-2.243874	0.555854	2.208777
H	-1.964217	-0.565034	0.868858
O	-3.546787	2.089598	-0.452375
H	0.894226	2.248266	-1.301731
H	-1.266481	-1.265451	-2.499975
O	-3.640255	-0.364779	-1.567180
O	-1.123077	3.490445	-0.296587
C	-3.677769	-1.658850	-2.129876
H	-3.379433	-1.646249	-3.185402
H	-3.023993	-2.351333	-1.584355
H	-4.710290	-1.999440	-2.050591
C	-1.248016	3.444448	1.119651
H	-0.416232	2.874062	1.555613
H	-1.210056	4.474128	1.481508
H	-2.200186	2.984415	1.401676

Structure **3h_TS**

C	4.041286	1.226549	0.995117
C	3.842448	0.252482	0.036564
C	2.636508	-0.489962	0.004843
C	1.627396	-0.204343	0.963921
C	1.857735	0.790179	1.937101
C	3.044501	1.498456	1.956914
C	2.352227	-1.444621	-1.005854
C	0.403135	-0.967307	0.923240
C	0.196831	-1.988916	0.026954
C	1.207867	-2.295222	-0.953236
C	-1.094811	-2.757605	-0.000469
C	-2.168653	-2.034300	0.808636
C	-1.611074	-1.504138	2.129486
H	4.972825	1.784797	1.011728
H	4.606934	0.040731	-0.704855
H	1.080440	0.996617	2.665698
H	3.209777	2.264078	2.708632
O	1.091047	-3.231407	-1.814081
O	3.332331	-1.800160	-1.897740
H	2.946482	-2.556187	-2.379590
C	1.221386	0.063680	-2.387041
H	2.205166	0.518625	-2.449539
H	1.025738	-0.770391	-3.057035
C	0.170940	0.754021	-1.809200
C	0.403622	1.943636	-1.041969
C	-1.172517	0.259840	-1.913201
C	-0.626030	2.601581	-0.431340
C	-2.209398	0.913278	-1.314460
C	-2.005829	2.128117	-0.521815
O	-0.521037	-0.593531	1.849896
C	-1.082729	-2.620969	3.027964
H	-0.724865	-2.202473	3.972927
H	-1.880514	-3.338645	3.241994
H	-0.256079	-3.154848	2.550738
C	-2.644275	-0.656610	2.854535
H	-2.210802	-0.217927	3.758036
H	-2.994914	0.149995	2.203110
H	-3.499911	-1.274137	3.141985
H	-0.939883	-3.775989	0.381173
H	-1.410909	-2.878959	-1.043652
H	-2.544548	-1.171896	0.246318
H	-3.018452	-2.693278	1.015425
O	-2.952399	2.720863	0.031891
O	-3.516587	0.526708	-1.367296
O	-0.499014	3.736127	0.315499
C	-3.823760	-0.573846	-2.204133
H	-3.334137	-1.490669	-1.853618
H	-4.904549	-0.700708	-2.157270

H	-3.517383	-0.375540	-3.237745
C	0.803770	4.279801	0.429405
H	0.708581	5.172545	1.046586
H	1.486681	3.568250	0.908868
H	1.200587	4.554291	-0.555402
H	1.428985	2.290308	-0.947089
H	-1.333435	-0.650065	-2.484550

Structure **QMh**

C	-0.000002	3.145541	-0.000075
H	-0.928953	3.706702	-0.000080
H	0.928947	3.706704	-0.000076
C	-0.000001	1.796323	-0.000068
C	-1.255992	1.055198	-0.000033
C	1.255991	1.055200	-0.000029
C	-1.265023	-0.295517	-0.000126
H	-2.175648	1.629737	0.000086
C	1.265023	-0.295516	-0.000122
H	2.175646	1.629740	0.000094
C	0.000000	-1.076348	-0.000453
O	0.000001	-2.298175	-0.000099
O	2.351845	-1.091186	-0.000030
C	3.617974	-0.445914	0.000340
H	3.735187	0.173496	0.895677
H	4.361420	-1.240560	0.000443
H	3.735623	0.173670	-0.894821
O	-2.351844	-1.091188	-0.000039
C	-3.617972	-0.445916	0.000347
H	-4.361417	-1.240562	0.000460
H	-3.735173	0.173495	0.895685
H	-3.735634	0.173668	-0.894812

Structure (*R*,*R*)-**3i**

C	-3.159725	-1.870997	-2.062825
C	-3.171148	-1.165847	-0.860089
C	-1.984460	-0.921074	-0.169369
C	-0.778119	-1.425625	-0.678069
C	-0.767007	-2.121227	-1.892636
C	-1.953554	-2.335854	-2.587491
C	-1.984832	-0.074120	1.076482
C	0.461267	-1.273520	0.107613
C	0.485018	-0.772569	1.376410
C	-0.747024	-0.288334	1.954707
C	1.736783	-0.783153	2.206539
H	1.488698	-1.087017	3.227582
C	2.750907	-1.743775	1.592384
C	2.858072	-1.543997	0.081115
H	-4.090855	-2.054732	-2.589414
H	-4.106721	-0.802086	-0.448481
H	0.172457	-2.499392	-2.279157
H	-1.938694	-2.876816	-3.528058
H	3.741852	-1.610259	2.036264
O	-0.846002	0.028713	3.143089
O	-3.134408	-0.384220	1.845979
H	-2.930633	-0.092773	2.749651
C	-2.004538	1.472360	0.738035
C	-0.753592	1.975370	0.050226
C	0.249328	2.628610	0.781249
C	-0.519974	1.804239	-1.322784
C	1.417048	3.094615	0.189040
C	0.644403	2.257660	-1.933887
C	1.678724	2.931256	-1.208579
O	1.543326	-1.749653	-0.518659
H	2.440033	-2.778546	1.779777
H	2.147464	0.233413	2.276746
C	3.738892	-2.604148	-0.559007
H	3.381528	-3.605880	-0.304910
H	3.737778	-2.492565	-1.646842
H	4.765588	-2.495429	-0.199066
C	3.317140	-0.138399	-0.290917
H	4.295042	0.060360	0.158590
H	3.407026	-0.050035	-1.377533
H	2.610505	0.622578	0.053930
O	2.759275	3.356266	-1.768931
H	0.790772	2.105750	-3.001686
H	2.169308	3.599191	0.792401
H	-1.264410	1.295196	-1.934829
H	0.106670	2.774228	1.852809
C	-3.287346	1.873733	0.005835
H	-3.292983	1.524126	-1.029935
H	-4.171107	1.474609	0.510433
H	-3.358794	2.965068	-0.010795
H	-2.035631	1.930927	1.736819

Structure (*R*,*R*)-**3i_TS**

C	3.253193	-1.053181	2.425589
C	3.306559	-0.898272	1.054565
C	2.116563	-0.857671	0.286831
C	0.866630	-1.001389	0.950220
C	0.839362	-1.156333	2.351828
C	2.011443	-1.174566	3.084944
C	2.112609	-0.624632	-1.113346
C	-0.335380	-1.049452	0.151378
C	-0.317772	-0.966157	-1.220466
C	0.935034	-0.781692	-1.906813
C	-1.584046	-1.036392	-2.028157
C	-2.812111	-0.895716	-1.131632
C	-2.654611	-1.688562	0.165049
H	4.174093	-1.084182	3.000617
H	4.260693	-0.802540	0.545399
H	-0.120737	-1.263993	2.845707
H	1.978121	-1.293009	4.163277
O	1.032948	-0.704363	-3.176712
O	3.298897	-0.605813	-1.804314

H	3.022627	-0.601356	-2.740721
C	1.774839	1.736467	-0.938004
H	1.813776	1.680857	-2.026846
C	0.530138	2.045031	-0.381147
C	0.309043	2.236891	1.020318
C	-0.611410	2.181817	-1.237665
C	-0.922290	2.563411	1.519357
H	1.140414	2.106055	1.708665
C	-1.841279	2.530190	-0.762872
H	-0.467696	2.016898	-2.305140
C	-2.076874	2.734761	0.657069
H	-1.073778	2.697571	2.587006
H	-2.688825	2.650296	-1.432768
O	-1.480747	-1.213144	0.867618
C	-2.488769	-3.186628	-0.085941
H	-2.405944	-3.718428	0.866223
H	-3.356460	-3.573288	-0.628913
H	-1.591958	-3.392486	-0.677055
C	-3.809509	-1.417319	1.115840
H	-3.650777	-1.935167	2.066267
H	-3.896710	-0.343901	1.308767
H	-4.746097	-1.772669	0.676981
H	-1.616783	-1.979441	-2.590693
H	-1.569287	-0.240632	-2.781893
H	-2.949284	0.157078	-0.860464
H	-3.717477	-1.229451	-1.649029
O	-3.212236	3.037819	1.106563
C	3.080235	2.010116	-0.262435
H	3.040788	1.852480	0.817261
H	3.873853	1.389348	-0.683131
H	3.346979	3.059629	-0.441807

Structure (*R*,*S*)-**3i**

C	-2.764123	-2.185459	-2.306623
C	-2.950798	-1.430803	-1.150098
C	-1.868094	-1.103639	-0.334567
C	-0.585614	-1.545175	-0.682773
C	-0.397104	-2.291833	-1.852412
C	-1.482663	-2.609055	-2.662667
C	-2.077486	-0.207165	0.861947
C	0.550988	-1.235921	0.204445
C	0.409289	-0.629114	1.417534
C	-0.917627	-0.252748	1.858294
C	1.573794	-0.451240	2.349252
H	1.255461	-0.679633	3.370300
C	2.717091	-1.368685	1.925087
C	2.953769	-1.301538	0.416776
H	-3.615363	-2.437906	-2.930926
H	-3.943866	-1.089957	-0.872942
H	0.601175	-2.621213	-2.116847
H	-1.330580	-3.189071	-3.567098
H	3.646503	-1.105991	2.438955

O	-1.160642	0.073796	3.023123
O	-3.260695	-0.616978	1.526468
H	-3.178398	-0.293973	2.438253
C	-2.247397	1.286044	0.355542
H	-3.010185	1.199315	-0.429692
C	-0.991296	1.851282	-0.277431
C	-0.029488	2.556137	0.463417
C	-0.722503	1.669254	-1.640768
C	1.128291	3.057471	-0.117918
C	0.435017	2.155354	-2.241101
C	1.426176	2.881141	-1.507615
O	1.723293	-1.670837	-0.274181
H	2.474427	-2.405405	2.187434
H	1.891798	0.600698	2.350336
C	3.969952	-2.338510	-0.032444
H	4.064887	-2.329836	-1.121828
H	4.946177	-2.109965	0.403738
H	3.666793	-3.338737	0.289106
C	3.338440	0.096746	-0.054194
H	2.542552	0.820965	0.144585
H	4.247767	0.423184	0.459997
H	3.529454	0.087628	-1.131350
O	2.501615	3.340397	-2.050836
H	0.604487	1.994880	-3.304050
H	1.850891	3.597906	0.490993
H	-1.444159	1.123003	-2.249938
H	-0.183812	2.717401	1.529772
C	-2.822825	2.188573	1.452319
H	-2.941103	3.200253	1.054156
H	-3.804408	1.835229	1.776455
H	-2.173805	2.243502	2.330004

Structure (*R*,*R*)-**3i_TS**

C	2.627526	1.677202	2.592162
C	2.973225	0.717446	1.660794
C	1.978608	-0.088867	1.056597
C	0.618130	0.116012	1.409758
C	0.291846	1.096129	2.369440
C	1.277182	1.869575	2.953693
C	2.278416	-1.046398	0.052228
C	-0.389215	-0.709559	0.783396
C	-0.073313	-1.731377	-0.078616
C	1.308731	-1.988616	-0.405935
C	-1.139404	-2.572927	-0.723102
C	-2.501952	-1.898537	-0.585185
C	-2.712165	-1.347065	0.825753
H	3.400981	2.285314	3.052272
H	4.011331	0.565251	1.381359
H	-0.750718	1.241403	2.632184
H	1.014011	2.623724	3.688620
O	1.687257	-2.973110	-1.120844
O	3.589084	-1.345777	-0.223237

H	3.536010	-2.167112	-0.747999
C	1.837449	0.457874	-1.774635
H	2.748658	0.889548	-1.362236
C	0.661963	1.187629	-1.571539
C	0.670613	2.350310	-0.734171
C	-0.592554	0.842704	-2.174031
C	-0.436474	3.129480	-0.550638
H	1.604130	2.611219	-0.235935
C	-1.713383	1.600252	-1.993100
H	-0.653805	-0.052964	-2.788279
C	-1.704860	2.801565	-1.173030
H	-0.401034	4.012404	0.081917
H	-2.657226	1.320402	-2.454314
O	-1.665924	-0.393919	1.129972
C	-2.674453	-2.445955	1.886630
H	-2.867143	-2.018224	2.874617
H	-3.439549	-3.199170	1.675135
H	-1.698844	-2.940025	1.908041
C	-4.001066	-0.544764	0.910513
H	-4.103924	-0.093179	1.901581
H	-4.000275	0.250443	0.158470
H	-4.860776	-1.197217	0.733382
H	-1.153982	-3.578287	-0.280560
H	-0.887278	-2.717296	-1.780211
H	-2.570663	-1.057597	-1.284433
H	-3.313286	-2.595332	-0.819165
O	-2.735788	3.504690	-1.013823
C	2.038379	-0.513674	-2.892611
H	2.884581	-1.172916	-2.691191
H	1.160938	-1.138519	-3.073803
H	2.261468	0.042440	-3.812223

Structure **QMi**

C	2.221494	0.528332	0.000019
H	2.519453	1.576731	0.000032
C	0.888256	0.274313	0.000003
C	0.318489	-1.067736	-0.000012
C	-0.049948	1.391528	0.000003
C	-1.015104	-1.267341	-0.000018
H	0.986822	-1.922390	-0.000015
C	-1.385493	1.207670	-0.000004
H	0.366151	2.396167	0.000009
C	-1.963622	-0.142525	-0.000007
H	-1.441223	-2.265774	-0.000029
H	-2.078878	2.042718	-0.000005
O	-3.184695	-0.328359	-0.000023
C	3.343909	-0.449982	0.000029
H	3.977563	-0.277681	-0.876733
H	3.977405	-0.277835	0.876937
H	3.022379	-1.490617	-0.000089

Structure **4xH_2O**

O	0.399217	0.016219	0.893017
H	1.281759	0.009651	0.467160
O	-1.781094	-1.360666	-0.190827
H	-0.915169	-1.127307	0.191662
H	-1.616682	-2.042865	-0.850682
O	-1.774812	1.498116	-0.295834
H	-0.946637	1.304120	0.170922
H	-2.113697	0.610977	-0.492962
O	2.858662	0.009106	-0.350054
H	3.037042	0.744170	-0.948971
H	3.104464	-0.789648	-0.832041
H	0.553142	-0.011303	1.844495

Structure **H_3O^+_3xH_2O**

O	0.001201	-0.004811	-0.519419
H	-0.769143	-0.616093	-0.230844
O	-1.862679	-1.567618	0.148205
H	-2.334036	-1.389724	0.972605
H	-2.534266	-1.738370	-0.525210
O	-0.287851	2.414749	0.137524
H	-0.658574	2.997121	-0.538477
H	-0.757786	2.607392	0.959460
O	2.237676	-0.953989	0.159656
H	2.659835	-0.593378	0.950596
H	2.913290	-0.976330	-0.530804
H	0.915150	-0.366210	-0.228801
H	-0.141244	0.968944	-0.236253

Structure **2**

C	3.431674	-2.068193	0.020916
C	3.315812	-0.698382	0.042817
C	2.036426	-0.083408	-0.006757
C	0.873666	-0.901344	-0.079615
C	1.025458	-2.312448	-0.097116
C	2.275712	-2.884091	-0.049006
C	1.885292	1.326080	0.029347
C	-0.411940	-0.280340	-0.133463
C	-0.545081	1.093137	-0.126939
C	0.631394	1.882825	-0.024843
C	-1.897680	1.750897	-0.241665
H	-1.854178	2.570581	-0.967847
C	-2.943783	0.731207	-0.688136
C	-2.787277	-0.590755	0.064740
H	4.413784	-2.529086	0.058499
H	4.196339	-0.067075	0.098250
H	0.135651	-2.930180	-0.148752
H	2.379430	-3.964359	-0.063160
H	-3.953610	1.121750	-0.532927
O	0.587914	3.255136	-0.020269
O	3.005515	2.105065	0.119955
H	2.726729	3.030683	0.179400
O	-1.475648	-1.131424	-0.219616

H	-2.826149	0.526309	-1.758424
H	-2.189782	2.195625	0.719962
C	-3.760783	-1.634912	-0.457909
H	-3.645142	-1.758397	-1.538343
H	-3.579464	-2.597420	0.028680
H	-4.788114	-1.325569	-0.246946
C	-2.927524	-0.420050	1.576701
H	-3.898947	0.023275	1.815203
H	-2.856837	-1.394209	2.068190
H	-2.143931	0.226391	1.981724
H	-0.266211	3.580134	0.291495

Structure **2_anion**

C	3.436732	-2.047251	0.025753
C	3.321464	-0.678184	0.048880
C	2.040525	-0.047282	-0.003953
C	0.874829	-0.881160	-0.084981
C	1.032373	-2.289861	-0.102295
C	2.281321	-2.869297	-0.048883
C	1.879519	1.343286	0.026859
C	-0.402664	-0.250639	-0.146695
C	-0.540208	1.123143	-0.128549
C	0.622340	1.970911	-0.024071
C	-1.894580	1.771919	-0.221148
H	-1.842702	2.614715	-0.918384
C	-2.949906	0.763834	-0.667321
C	-2.779992	-0.570501	0.062281
H	4.420492	-2.506774	0.066359
H	4.205808	-0.050809	0.108066
H	0.142074	-2.908412	-0.158569
H	2.384447	-3.949839	-0.062648
H	-3.961074	1.146624	-0.493845
O	0.553066	3.264367	0.015271
O	2.976329	2.176905	0.115718
H	2.570917	3.065327	0.119311
O	-1.477074	-1.106137	-0.246821
H	-2.849932	0.570109	-1.742517
H	-2.166593	2.209621	0.749684
C	-3.764138	-1.607305	-0.457554
H	-3.665475	-1.717226	-1.541446
H	-3.577295	-2.577213	0.012829
H	-4.788492	-1.300311	-0.227736
C	-2.903882	-0.418792	1.578800
H	-3.871144	0.023827	1.836313
H	-2.829143	-1.399273	2.057997
H	-2.112863	0.221622	1.978506

Section 6. Supplemental figures for stability of lapachone prodrugs

6.1. Representative HPLC traces for each derivative at each pH studied.

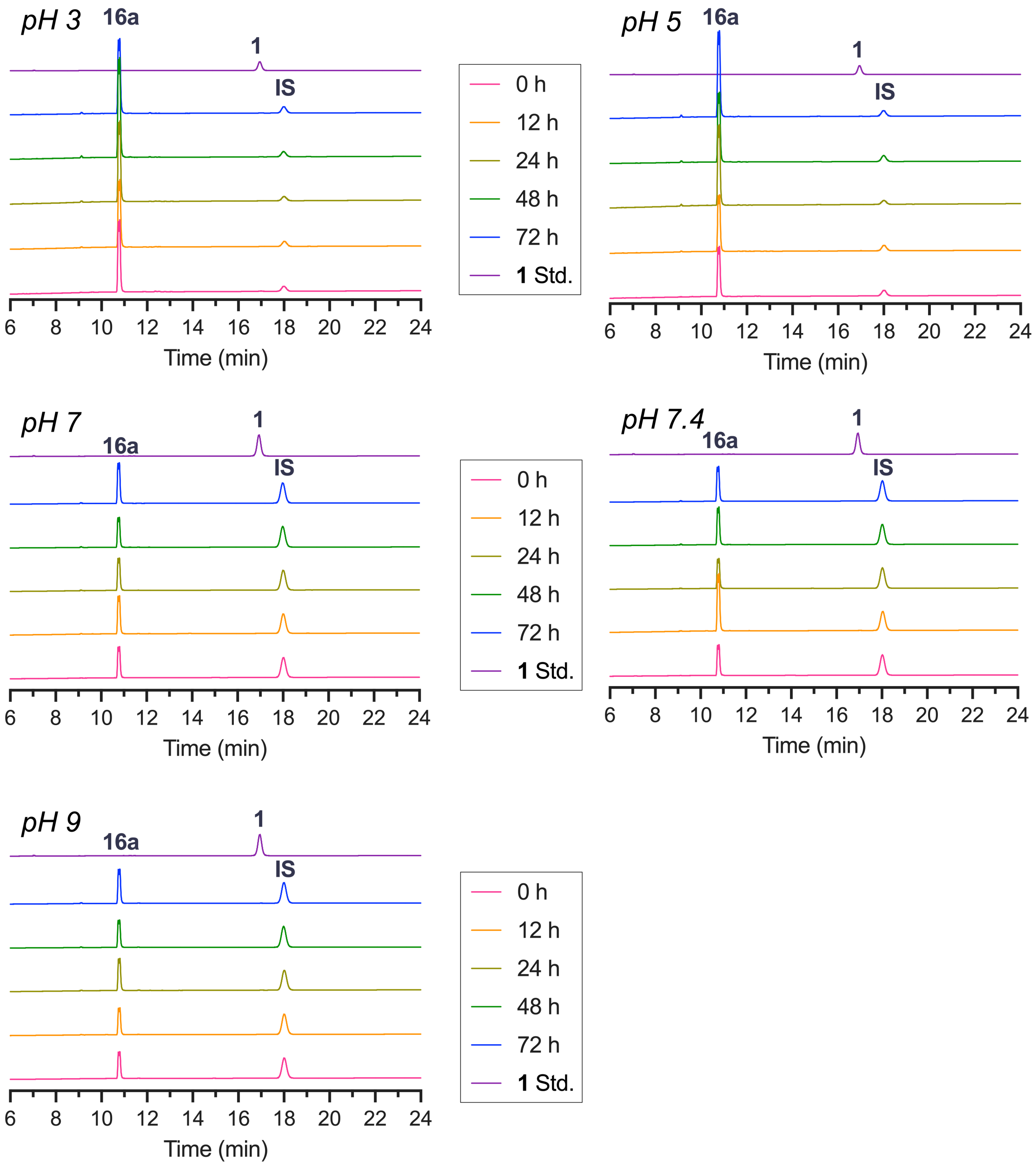

Figure S50. HPLC traces for 16a stability experiments. The representative traces shown are one replicate of the triplicate experiments performed. No lapachone peak was detected in any sample. RT **16a** = 10.7 min, RT **1** = 16.9 min, RT warfarin IS 18.0 = min.

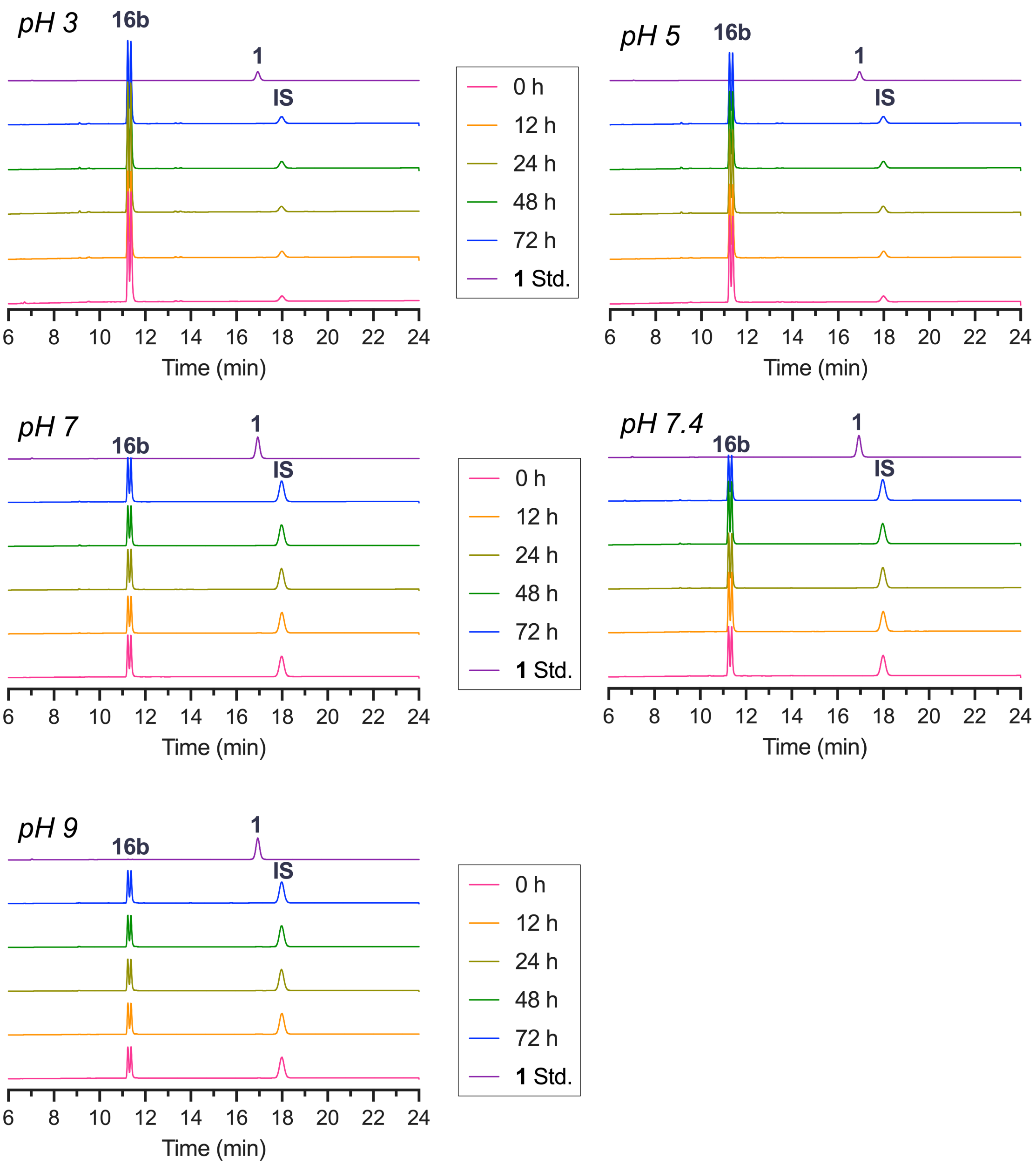

Figure S51. HPLC traces for 16b stability experiments. The representative traces shown are one replicate of the triplicate experiments performed. No lapachone peak was detected in any sample. RT **16b** = 11.2 min, RT **1** = 16.9 min, RT warfarin IS 18.0 = min.

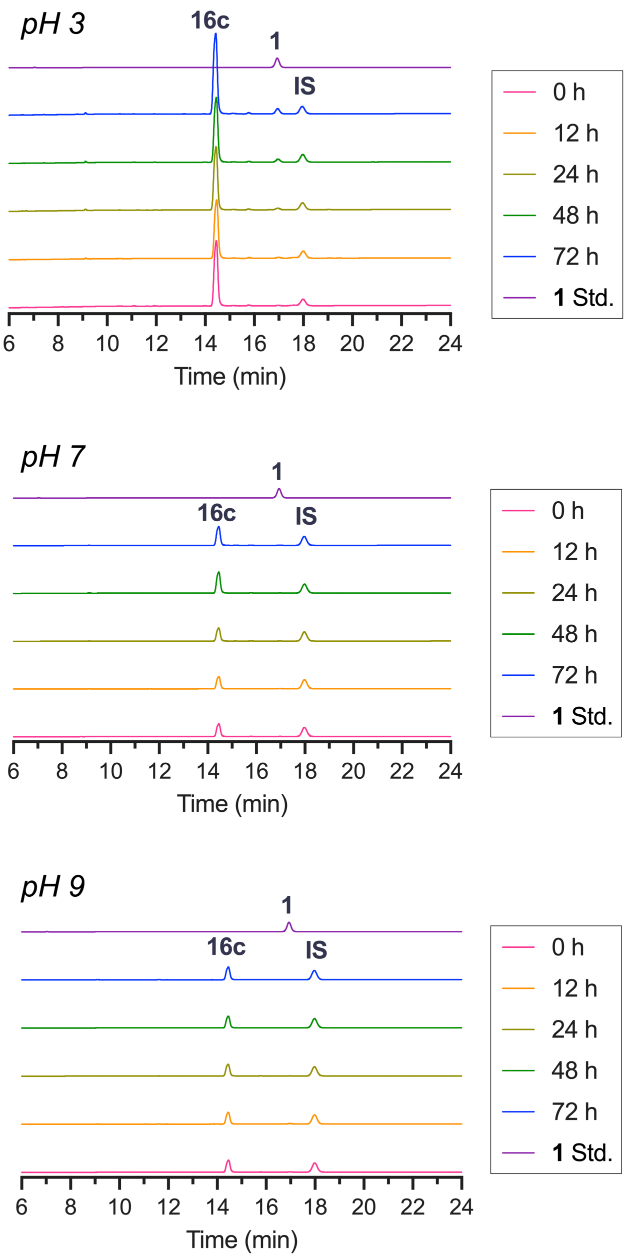

Figure S52. HPLC traces for 16c stability experiments. The representative traces shown are one replicate of the triplicate experiments performed. A minute lapachone peak was detected in some samples. RT **16c** = 14.4 min, RT **1** = 16.9 min, RT warfarin IS 18.0 = min.

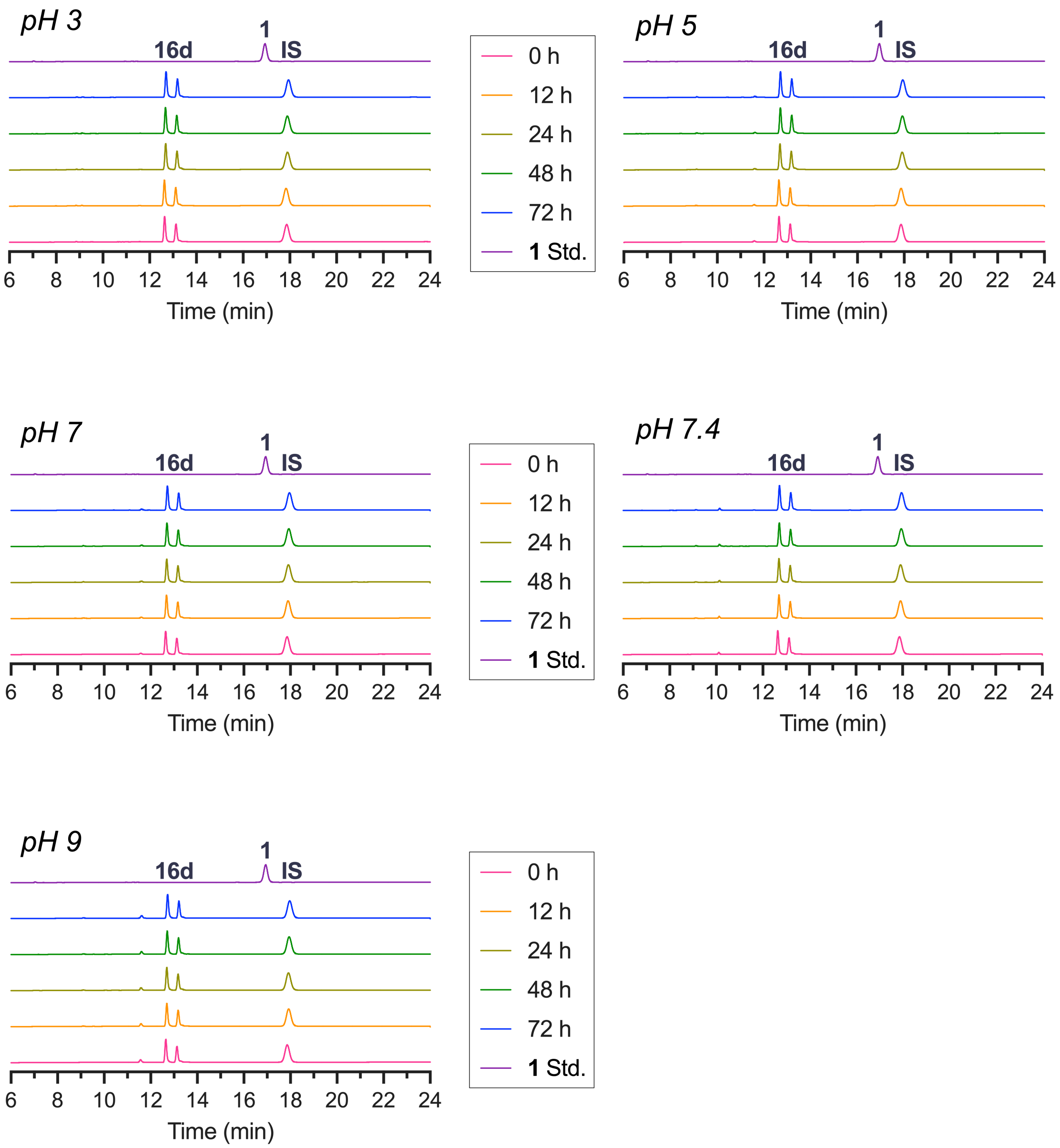

Figure S53. HPLC traces for 16d stability experiments. The representative traces shown are one replicate of the triplicate experiments performed. No lapachone peak was detected in any sample. RT **16d** = 12.6, 13.1 min, RT **1** = 16.9 min, RT warfarin IS 18.0 = min.

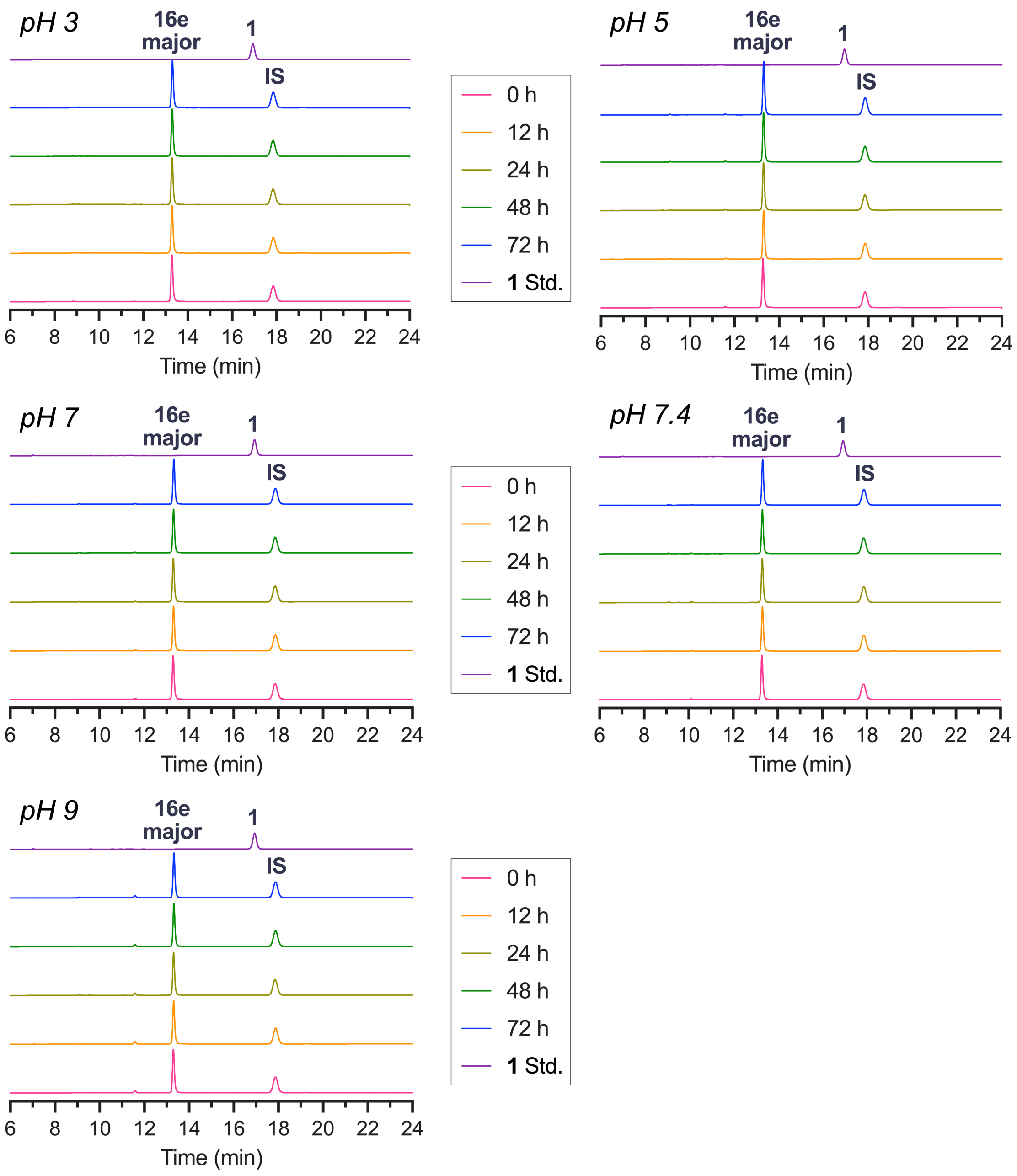

Figure S54. HPLC traces for the 16e major product stability experiments. The representative traces shown are one replicate of the triplicate experiments performed. No lapachone peak was detected in any sample. RT **16e** = 13.3 min, RT **1** = 16.9 min, RT warfarin IS 18.0 = min.

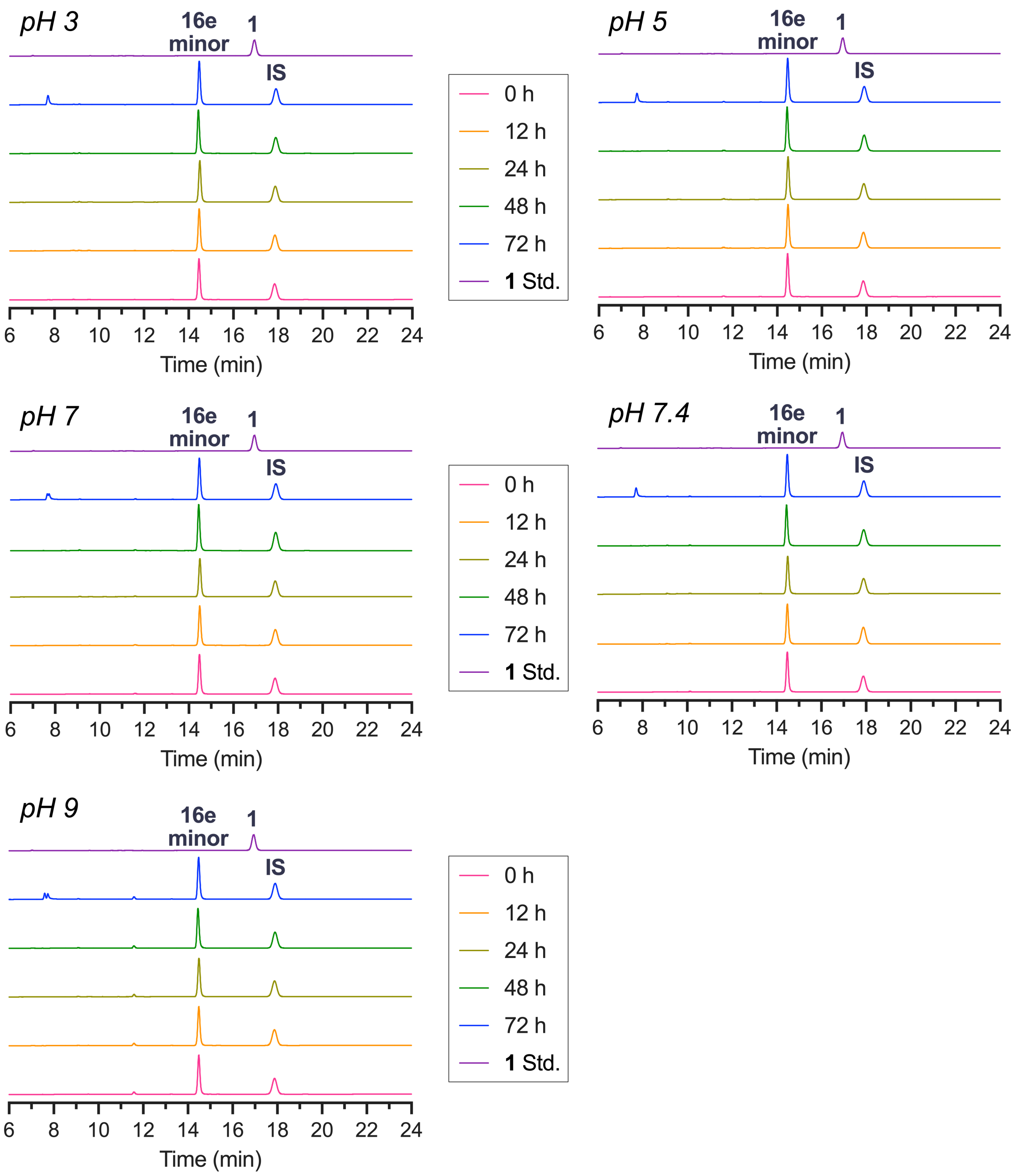

Figure S55. HPLC traces for the 16e minor product stability experiments. The representative traces shown are one replicate of the triplicate experiments performed. No lapachone peak was detected in any sample. RT **16e** = 14.4 min, RT **1** = 16.9 min, RT warfarin IS 18.0 = min.

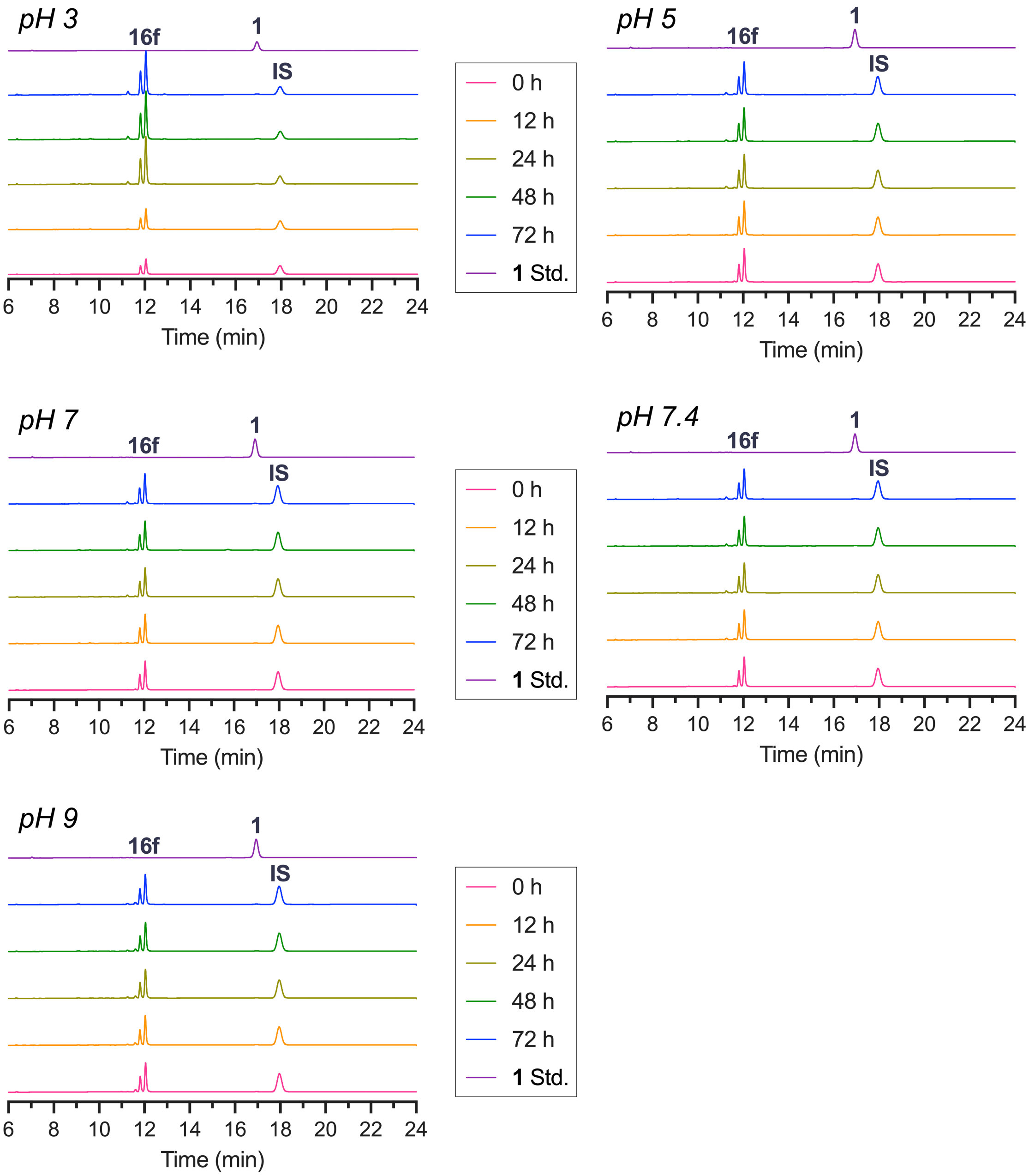

Figure S56. HPLC traces for 16f stability experiments. The representative traces shown are one replicate of the triplicate experiments performed. A minute lapachone peak was detected in some samples. RT **16f** = 11.7, 12.0 min, RT **1** = 16.9 min, RT warfarin IS 18.0 = min.

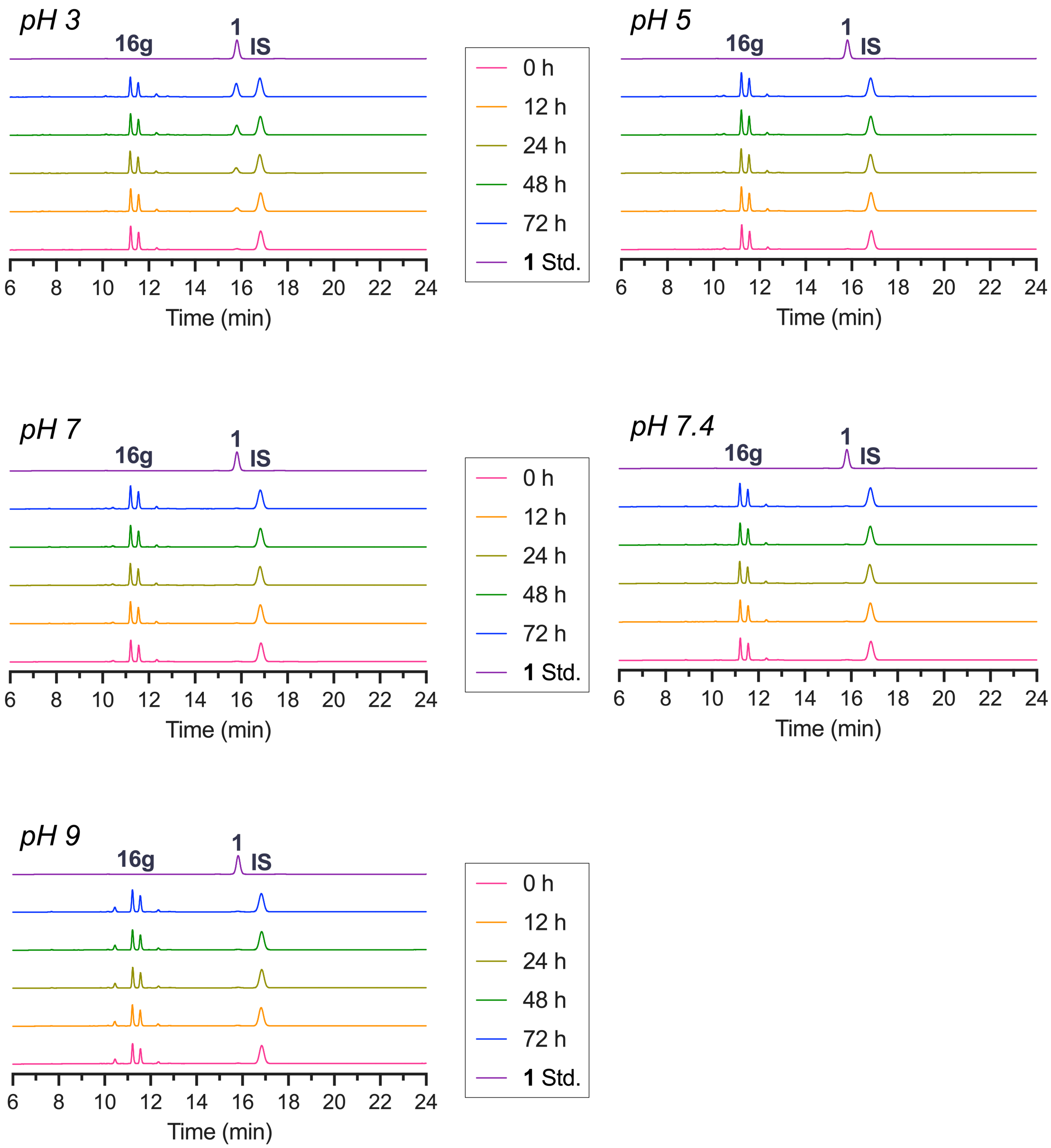

Figure S57. HPLC traces for 16g stability experiments. The representative traces shown are one replicate of the triplicate experiments performed. A minute lapachone peak was detected in some samples. RT **16g** = 11.2, 11.5 min, RT **1** = 15.8 min, RT warfarin IS 16.8 = min.

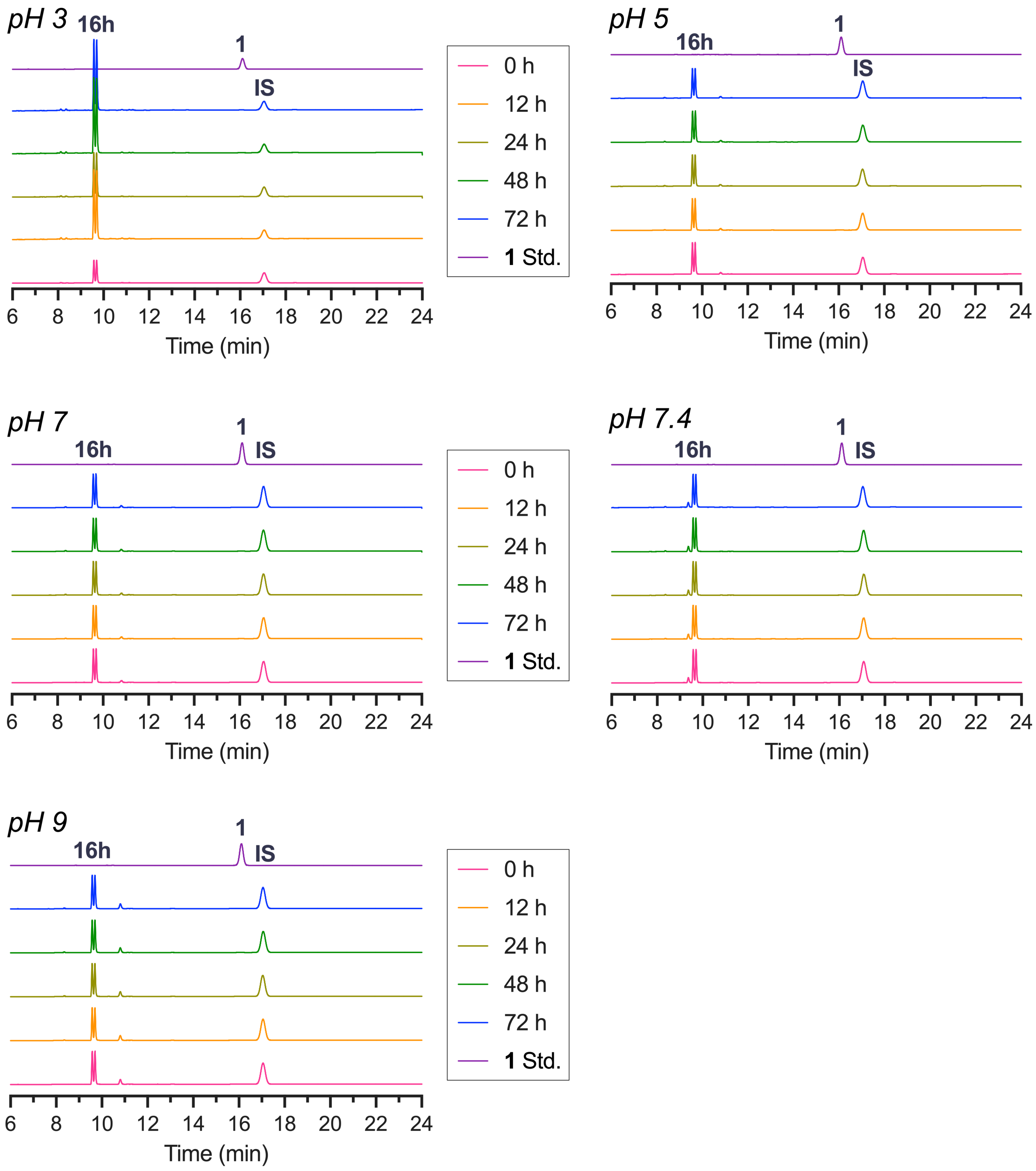

Figure S58. HPLC traces for 16h stability experiments. The representative traces shown are one replicate of the triplicate experiments performed. No lapachone peak was detected in any sample. RT **16h** = 9.6, 9.7 min, RT **1** = 16.0 min, RT warfarin IS 17.0 = min.

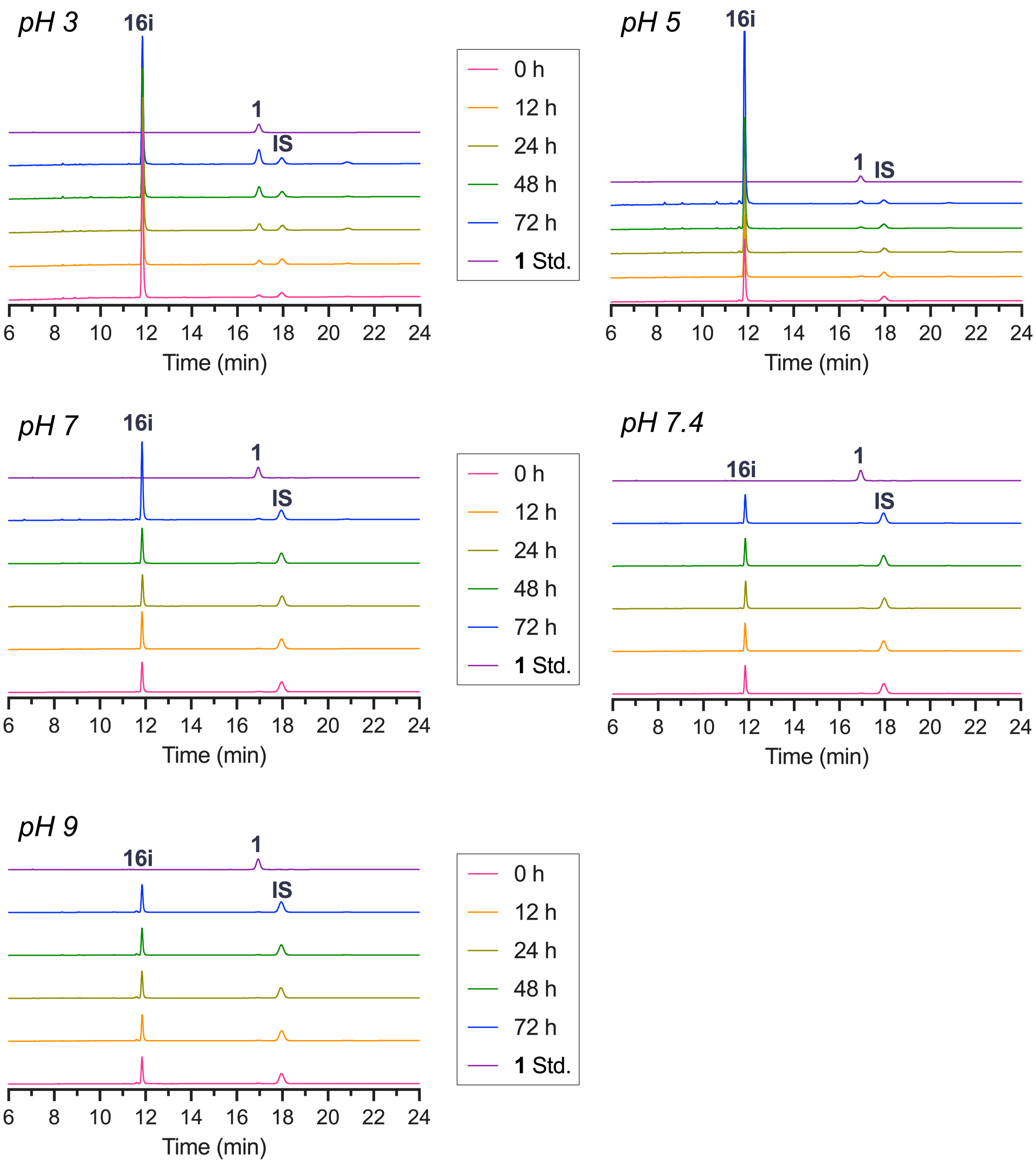

Figure S59. HPLC traces for 16i stability experiments. The representative traces shown are one replicate of the triplicate experiments performed. A minute lapachone peak was detected in some samples. RT **16i** = 11.8 min, RT **1** = 16.9 min, RT warfarin IS 18.0 = min.

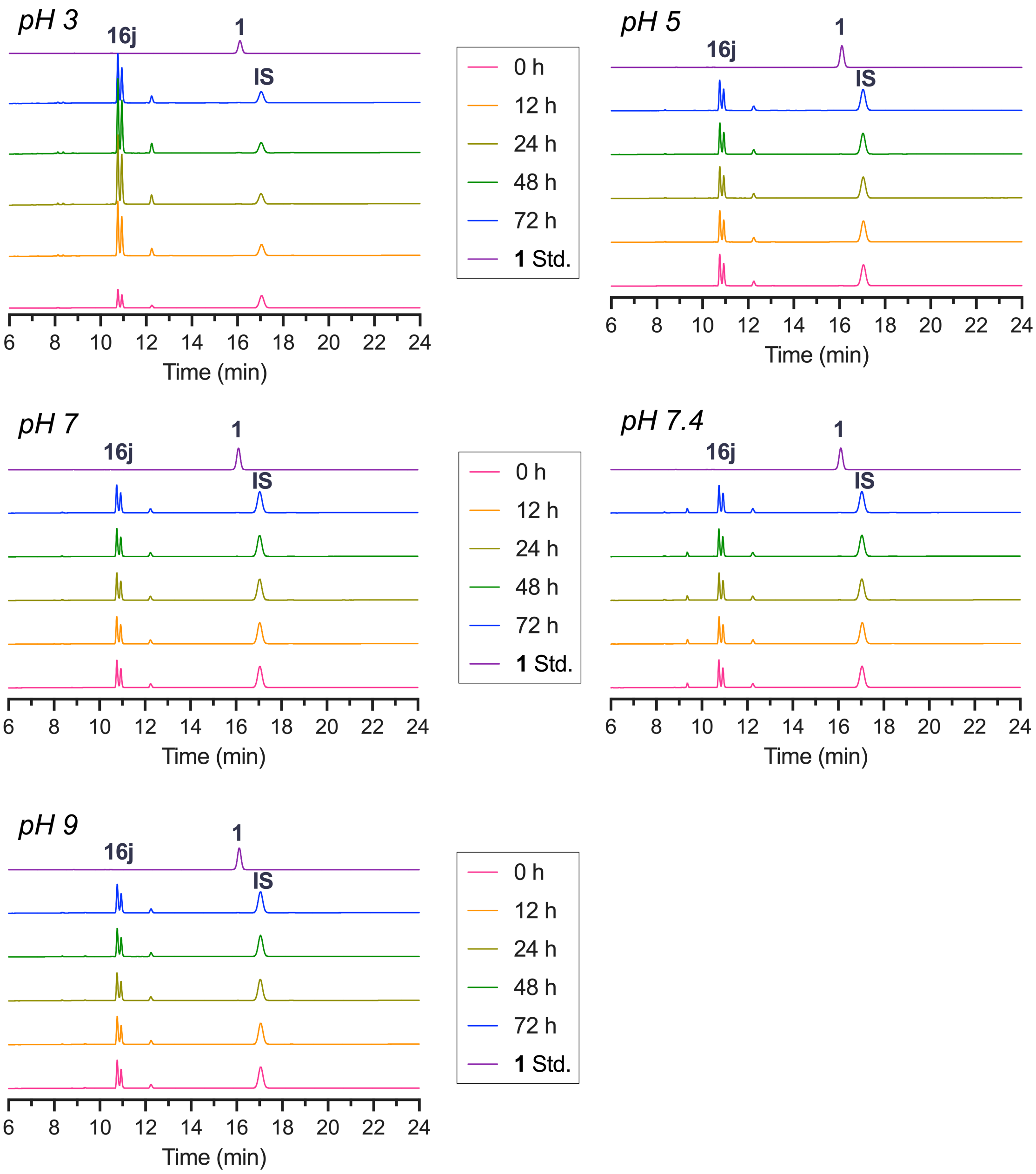

Figure S60. HPLC traces for 16j stability experiments. The representative traces shown are one replicate of the triplicate experiments performed. No lapachone peak was detected in any sample. RT **16j** = 10.8, 10.9 min, RT **1** = 16.0 min, RT warfarin IS 17.0 = min.

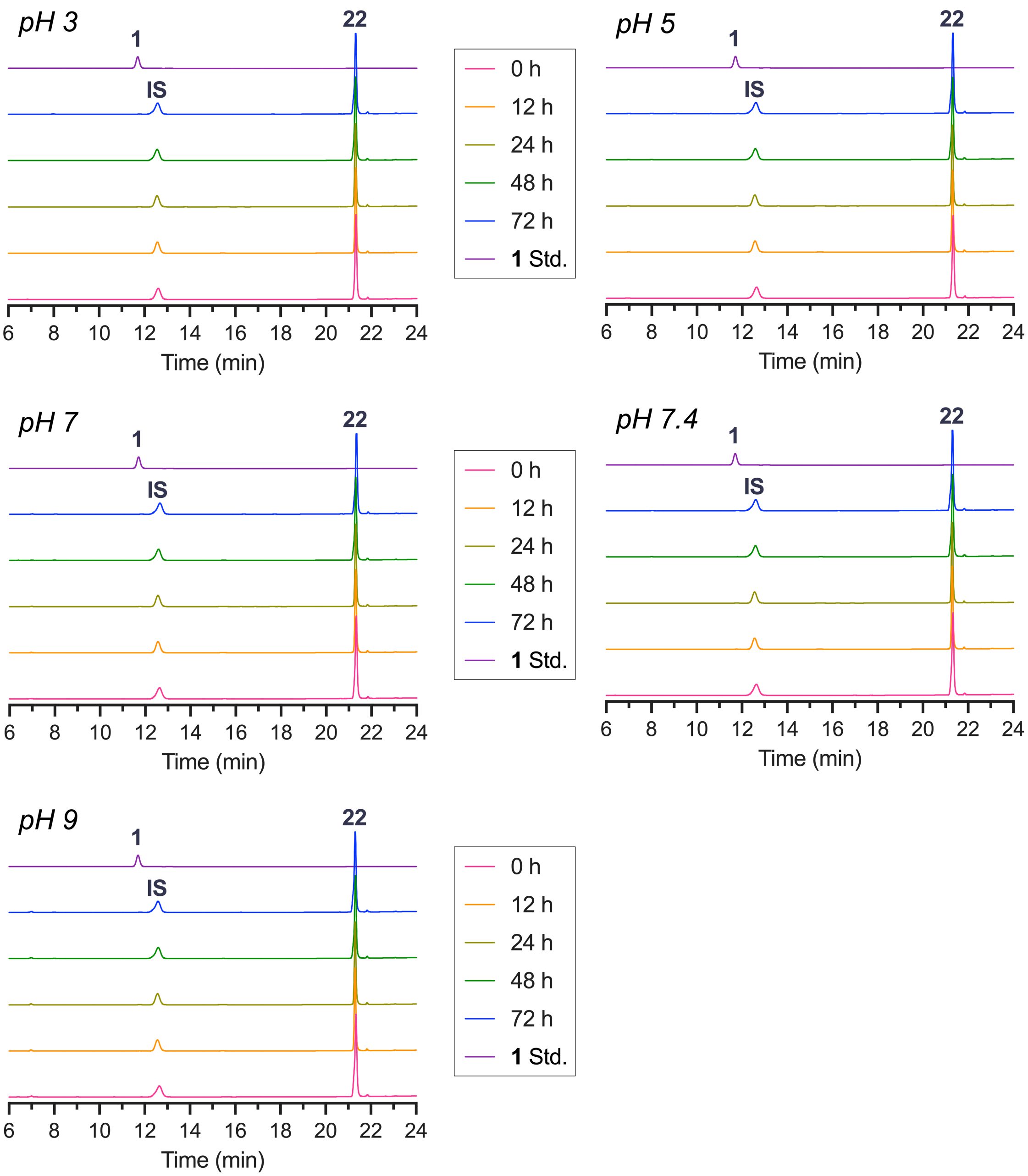

Figure S61. HPLC traces for 22 stability experiments. The representative traces shown are one replicate of the triplicate experiments performed. A minute lapachone peak was detected in some samples. RT **22** = 21.3 min, RT **1** = 11.6 min, RT warfarin IS 12.5 = min.

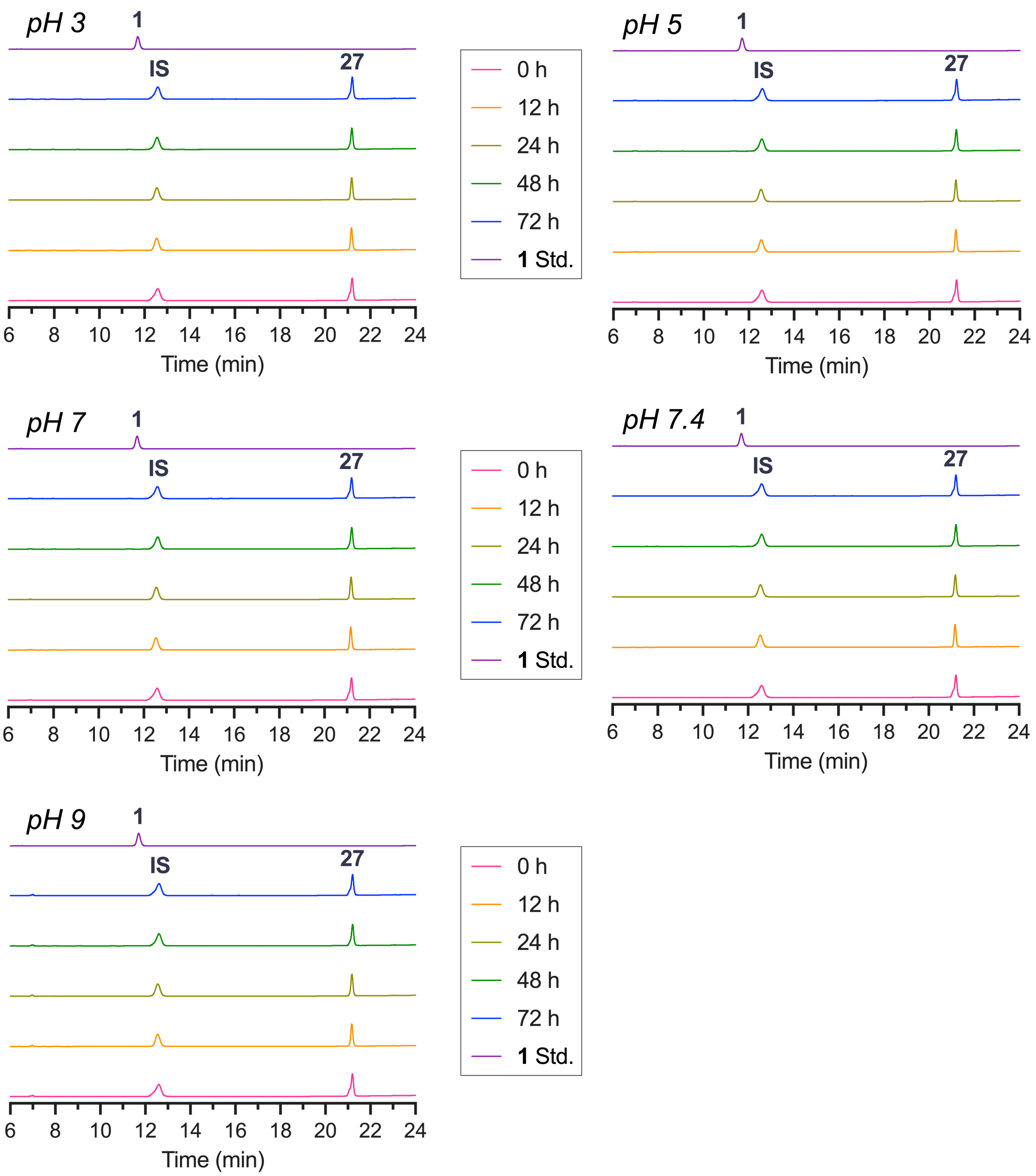

Figure S62. HPLC traces for 27 stability experiments. The representative traces shown are one replicate of the triplicate experiments performed. No lapachone peak was detected in any sample. RT **27** = 21.2 min, RT **1** = 11.7 min, RT warfarin IS 12.6 = min.

6.2. Prodrug and lapachone peak area graphs for each derivative

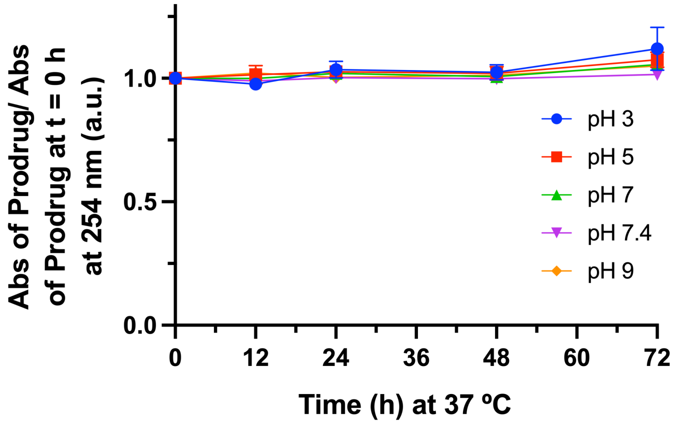

Figure S63. Prodrug peak area of 16a. The peak area of **16a** divided by the starting peak area of **16a** is plotted for each pH. No changes in the peak area were detected over the 72 h incubation period for pH 3-9. No lapachone peak was detected in any sample. Error bars represent ± SD of the triplicate experiments.

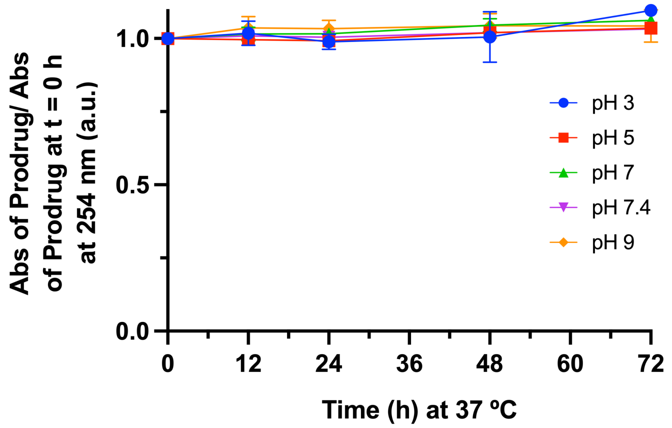

Figure S64. Prodrug peak area of 16b. The peak area of **16b** divided by the starting peak area of **16b** is plotted for each pH. No changes in the peak area were detected over the 72 h incubation period for pH 3-9. No lapachone peak was detected in any sample. Error bars represent ± SD of the triplicate experiments.

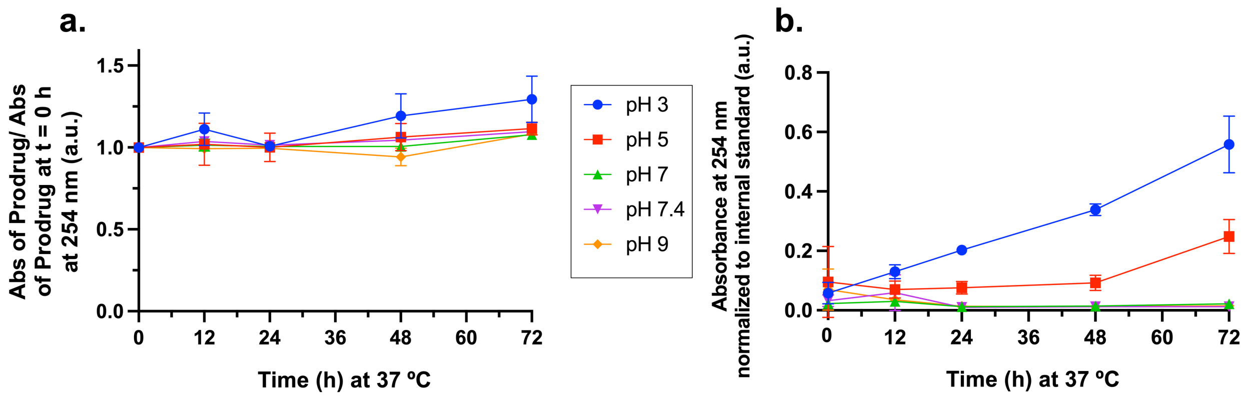

Figure S65. Prodrug peak area of 16c. (a) The peak area of **16c** divided by the starting peak area of **16c** is plotted for each pH. No changes in the peak area were detected over the 72 h incubation period for pH 3-9. (b) The area of the β-lapachone peak is plotted over time. The peak remained extremely small for pH 7-9, but increased over time for pH 3 and 5. Error bars represent ± SD of the triplicate experiments.

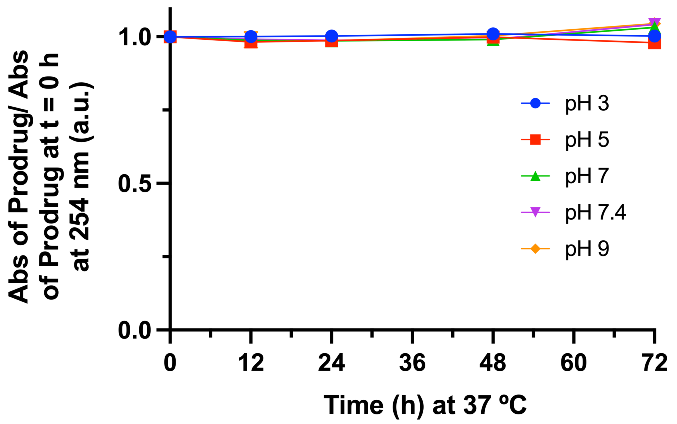

Figure S66. Prodrug peak area of 16d. The peak area of **16d** divided by the starting peak area of **16d** is plotted for each pH. No changes in the peak area were detected over the 72 h incubation period for pH 3-9. No lapachone peak was detected in any sample. Error bars represent ± SD of the triplicate experiments.

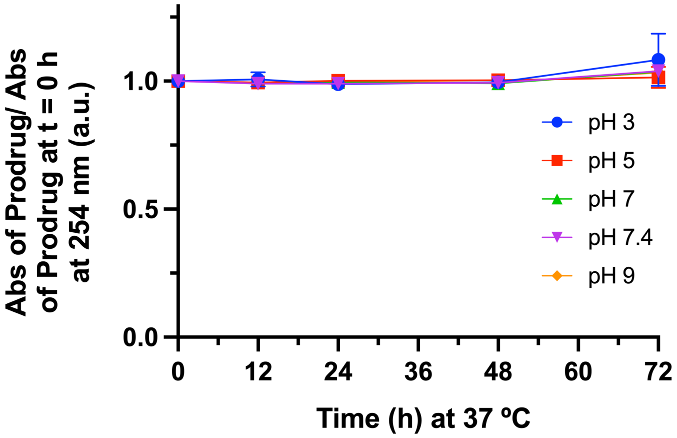

Figure S67. Prodrug peak area of the 16e major product. The peak area of **16e** divided by the starting peak area of **16e** is plotted for each pH. No changes in the peak area were detected over the 72 h incubation period for pH 3-9. No lapachone peak was detected in any sample. Error bars represent ± SD of the triplicate experiments.

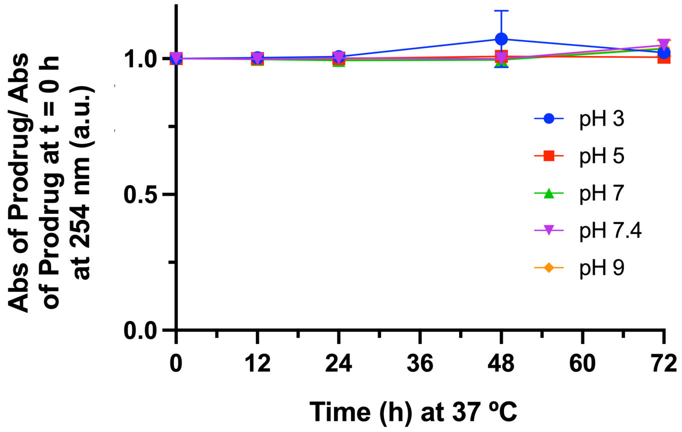

Figure S68. Prodrug peak area of the 16e minor product. The peak area of **16e** divided by the starting peak area of **16e** is plotted for each pH. No changes in the peak area were detected over the 72 h incubation period for pH 3-9. No lapachone peak was detected in any sample. Error bars represent ± SD of the triplicate experiments.

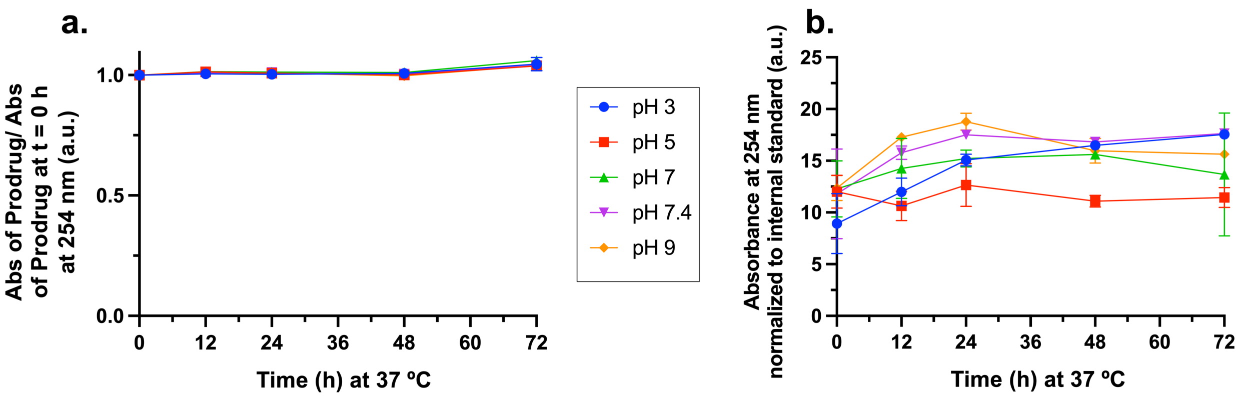

Figure S69. Prodrug peak area of 16f. (a) The peak area of **16f** divided by the starting peak area of **16f** is plotted for each pH. No changes in the peak area were detected over the 72 h incubation period for pH 3-9. (b) The area of the β-lapachone peak is plotted over time. The peak remained extremely small for pH 3-9. Error bars represent ± SD of the triplicate experiments.

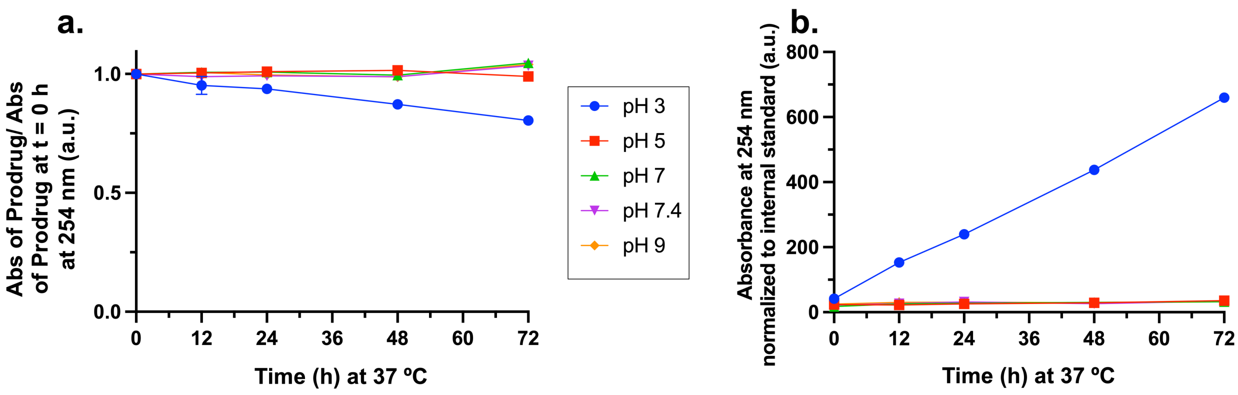

Figure S70. Prodrug peak area of 16g. (a) The peak area of **16g** divided by the starting peak area of **16g** is plotted for each pH. No changes in the peak area were detected over the 72 h incubation period for pH 5-9, but a substantial decrease in the peak area was detected for pH 3. (b) The area of the β-lapachone peak is plotted over time. The peak remained small for pH 5-9, but increased substantially over time for pH 3. Error bars represent ± SD of the triplicate experiments.

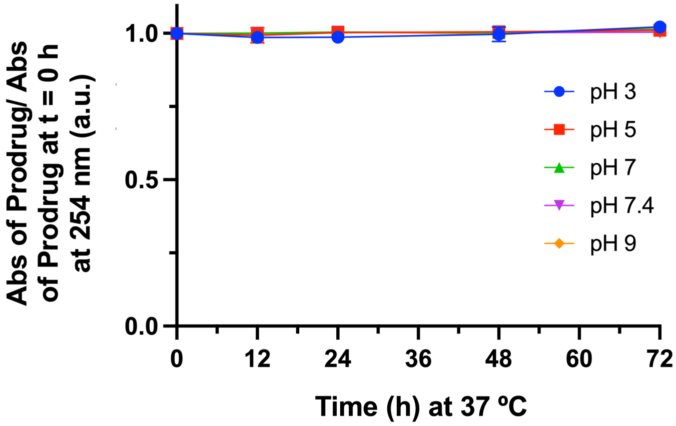

Figure S71. Prodrug peak area of 16h. The peak area of **16h** divided by the starting peak area of **16h** is plotted for each pH. No changes in the peak area were detected over the 72 h incubation period for pH 3-9. No lapachone peak was detected in any sample. Error bars represent ± SD of the triplicate experiments.

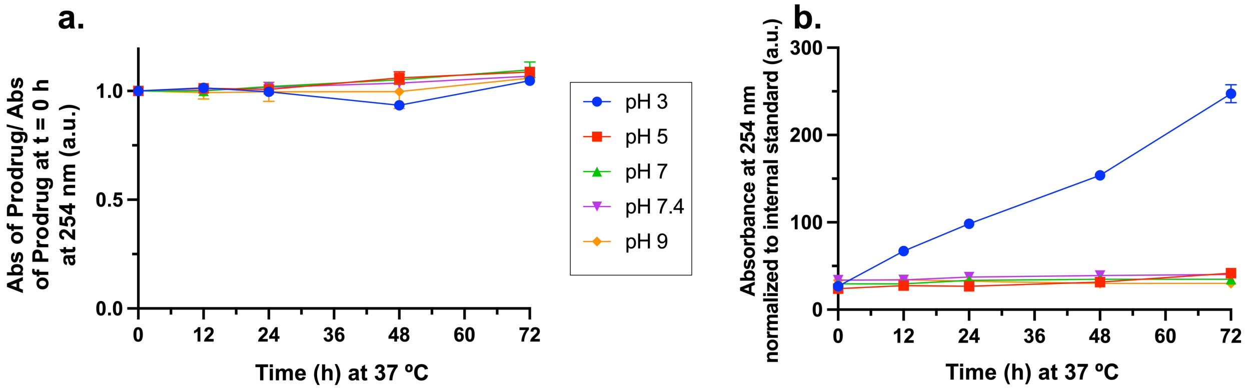

Figure S72. Prodrug peak area of 16i. (a) The peak area of **16i** divided by the starting peak area of **16i** is plotted for each pH. No changes in the peak area were detected over the 72 h incubation period for pH 3-9. (b) The area of the β-lapachone peak is plotted over time. The peak remained small for pH 5-9, but increased substantially over time for pH 3. Error bars represent ± SD of the triplicate experiments.

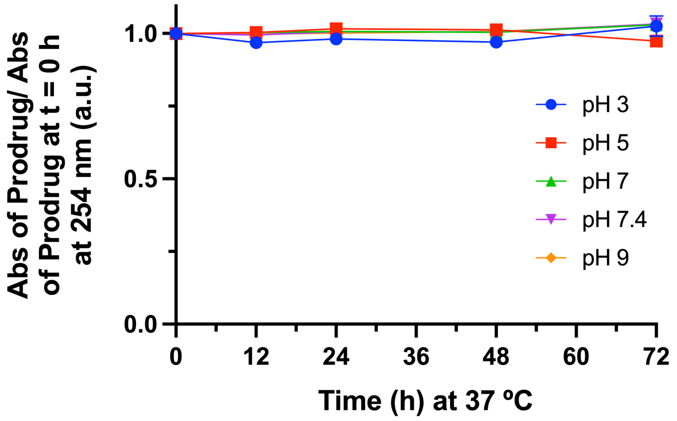

Figure S73. Prodrug peak area of 16j. The peak area of **16j** divided by the starting peak area of **16j** is plotted for each pH. No changes in the peak area were detected over the 72 h incubation period for pH 3-9. No lapachone peak was detected in any sample. Error bars represent ± SD of the triplicate experiments.

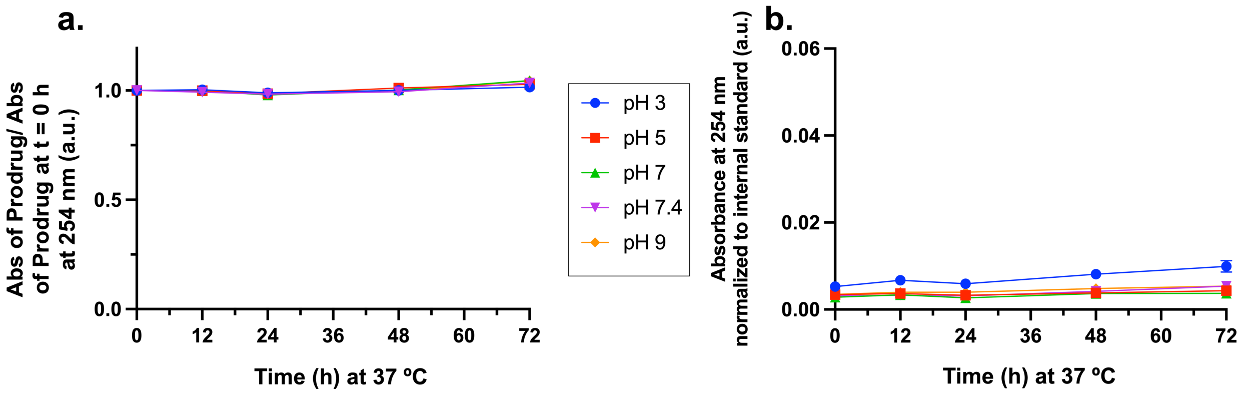

Figure S74. Prodrug peak area of 22. (a) The peak area of **22** divided by the starting peak area of **22** is plotted for each pH. No changes in the peak area were detected over the 72 h incubation period for pH 3-9. (b) The area of the β-lapachone peak is plotted over time. The peak remained extremely small for pH 3-9. Error bars represent ± SD of the triplicate experiments.

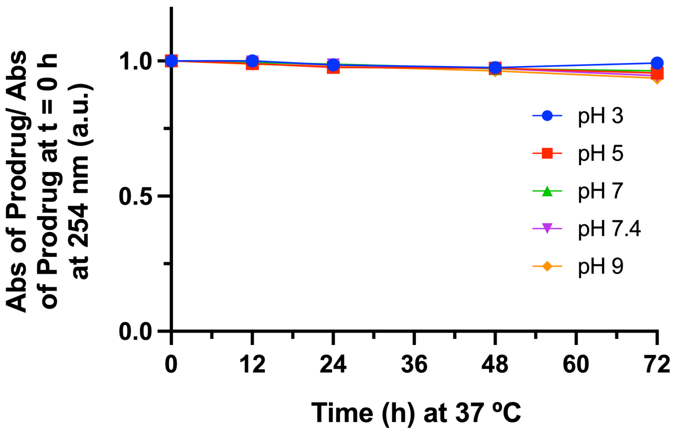

Figure S75. Prodrug peak area of 27. The peak area of **27** divided by the starting peak area of **27** is plotted for each pH. No changes in the peak area were detected over the 72 h incubation period for pH 3-9. No lapachone peak was detected in any sample. Error bars represent ± SD of the triplicate experiments.

6.3. Quantified β-lapachone released in prodrug stability assays

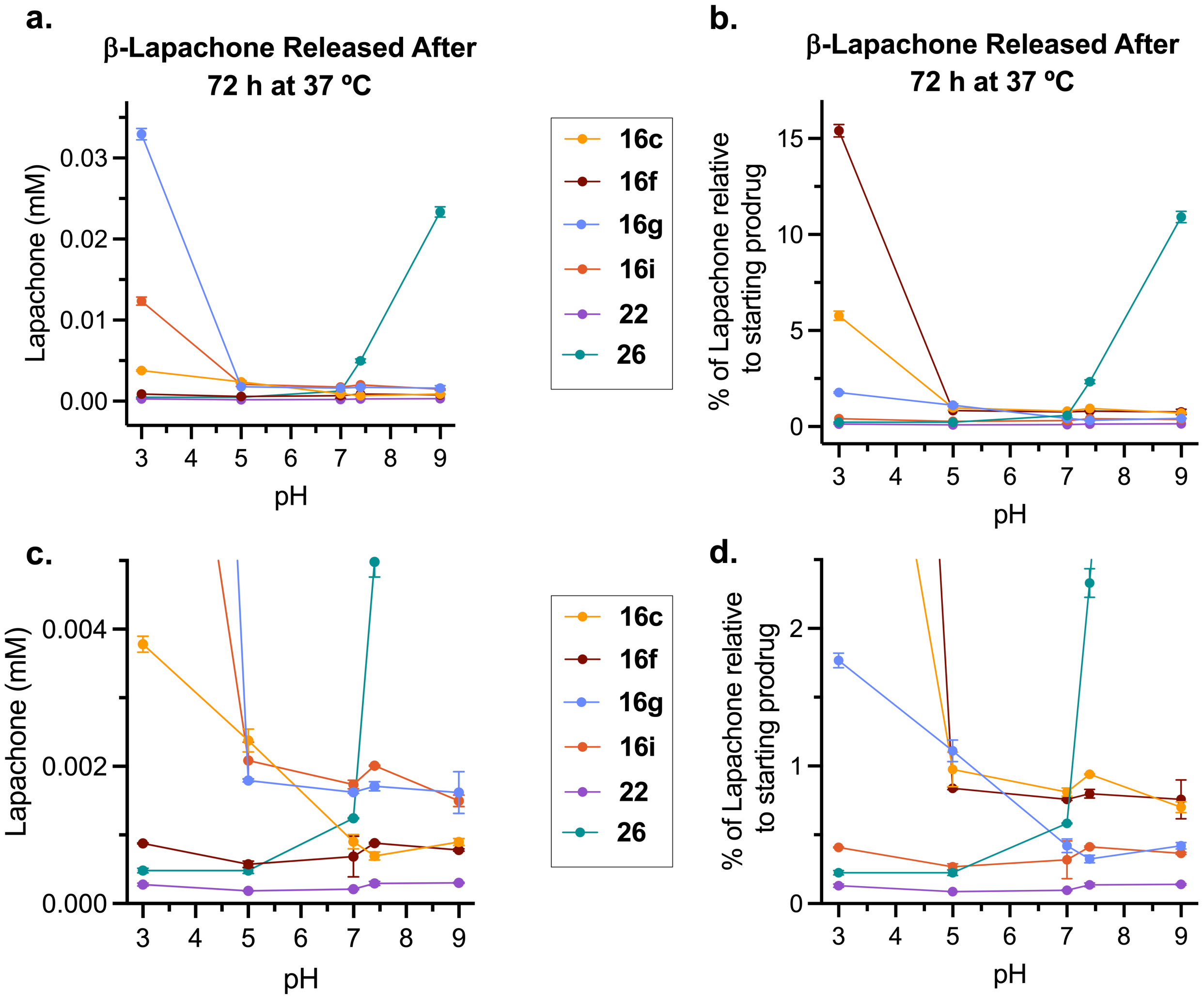

Figure S76. Quantified lapachone released during stability assays. (a) The lapachone released during the stability assays after 72 h incubation at 37 ºC was quantified using a standard curve. (b) This lapachone was then calculated as a percentage of starting prodrug concentration, such that it represents the percentage of lapachone that was prematurely released from the prodrug after 72 h. Panels (c) and (d) are zoomed in views of (a) and (b) respectively. Error bars represent ± SD.

6.4. Tables of stability data

Table S6.1. Concentration of Lapachone Released from Prodrug after 72 h at 37 °C (mM)

Derivative	pH 3	pH 5	pH 7	pH 7.4	pH 9
**16a**	0	0	0	0	0
**16b**	0	0	0	0	0
**16c**	0.004 ± 0.0001	0.002 ± 0.0002	0.0009 ±	0.0007 ±	0.0009 ±

			0.0001	0.00006	0.00005
**16d**	0	0	0	0	0
**16e major product**	0	0	0	0	0
**16e minor product**	0	0	0	0	0
**16f**	0.0009 ± 0.000009	0.0006 ± 0.00005	0.0007 ± 0.0003	0.0009 ± 0.00001	0.0008 ± 0.00002
**16g**	0.033 ± 0.0007	0.002 ± 0.00002	0.002 ± 0.00002	0.002 ± 0.00007	0.002 ± 0.0003
**16h**	0	0	0	0	0
**16i**	0.012 ± 0.0005	0.002 ± 0.0003	0.002 ± 0.00006	0.002 ± 0.00001	0.002 ± 0.00008
**16j**	0	0	0	0	0
**22**	0.0003 ± 0.00002	0.0002 ± 0.000004	0.0002 ± 0.000009	0.0003 ± 0.00003	0.0003 ± 0.00001
**24/26**	0.0005 ± 0.00003	0.0005 ± 0.00004	0.001 ± 0.000009	0.005 ± 0.0002	0.023 ± 0.0006

*Mean ± SD of Triplicate Experiments

Table S6.2. Percentage of Lapachone Released from Prodrug after 72 h at 37 °C*

Derivative	pH 3	pH 5	pH 7	pH 7.4	pH 9
**16a**	0	0	0	0	0
**16b**	0	0	0	0	0
**16c**	1.77 ± 0.05	1.11 ± 0.08	0.42 ± 0.05	0.32 ± 0.03	0.42 ± 0.02
**16d**	0	0	0	0	0
**16e major product**	0	0	0	0	0
**16e minor product**	0	0	0	0	0
**16f**	0.41 ± 0.005	0.27 ± 0.02	0.32 ± 0.14	0.41 ± 0.006	0.36 ± 0.009
**16g**	15.40 ± 0.33	0.84 ± 0.009	0.76 ± 0.008	0.80 ± 0.03	0.76 ± 0.14
**16h**	0	0	0	0	0
**16i**	5.78 ± 0.24	0.98 ± 0.13	0.81 ± 0.03	0.94 ± 0.005	0.70 ± 0.04
**16j**	0	0	0	0	0
**22**	0.13 ± 0.01	0.09 ± 0.002	0.09 ± 0.004	0.14 ± 0.01	0.14 ± 0.006
**24/26**	0.22 ± 0.02	0.22 ± 0.02	0.58 ± 0.004	2.33 ± 0.10	10.92 ± 0.29

*Mean ± SD of Triplicate Experiments

Section 7. Supplemental figures for comparison to boronate ester prodrug

7.1. Kinetics figures for boronate ester

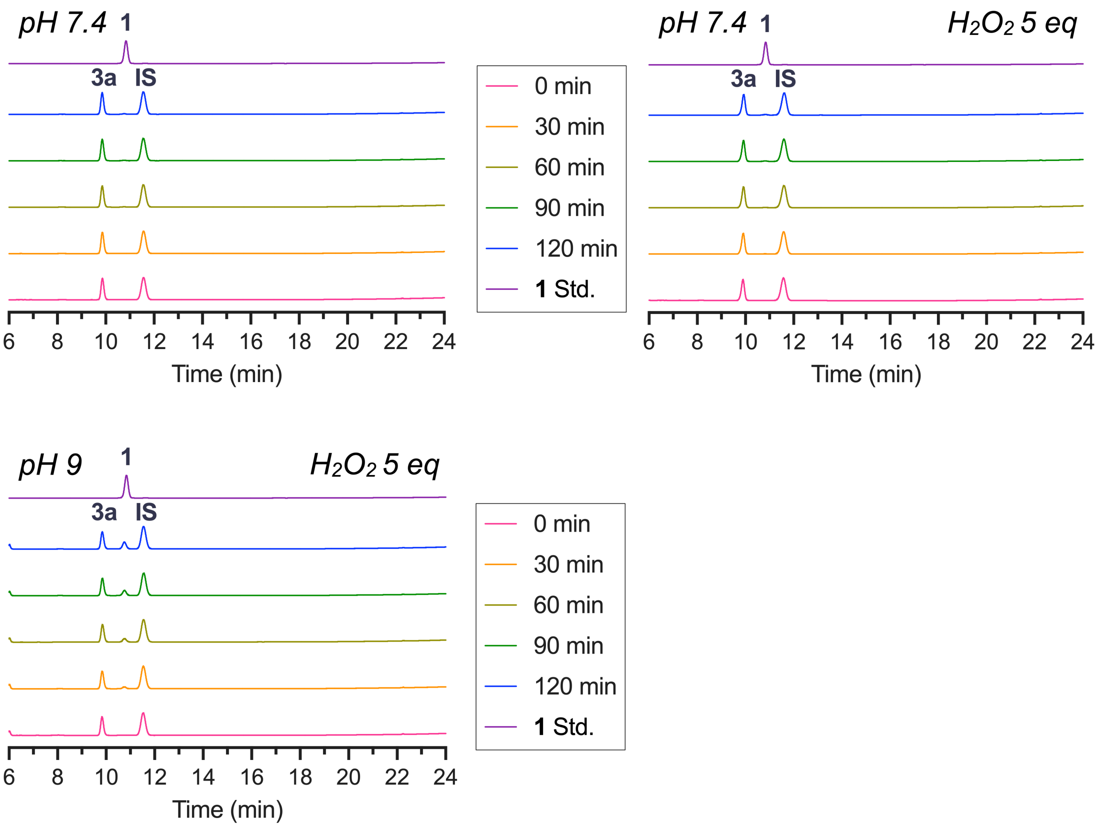

Figure S77. HPLC traces for 16a peroxide release experiments. The representative traces shown are one replicate of the triplicate experiments performed. RT **3a** = 9.9 min, RT **1** = 10.8 min, RT warfarin IS 11.6 = min.

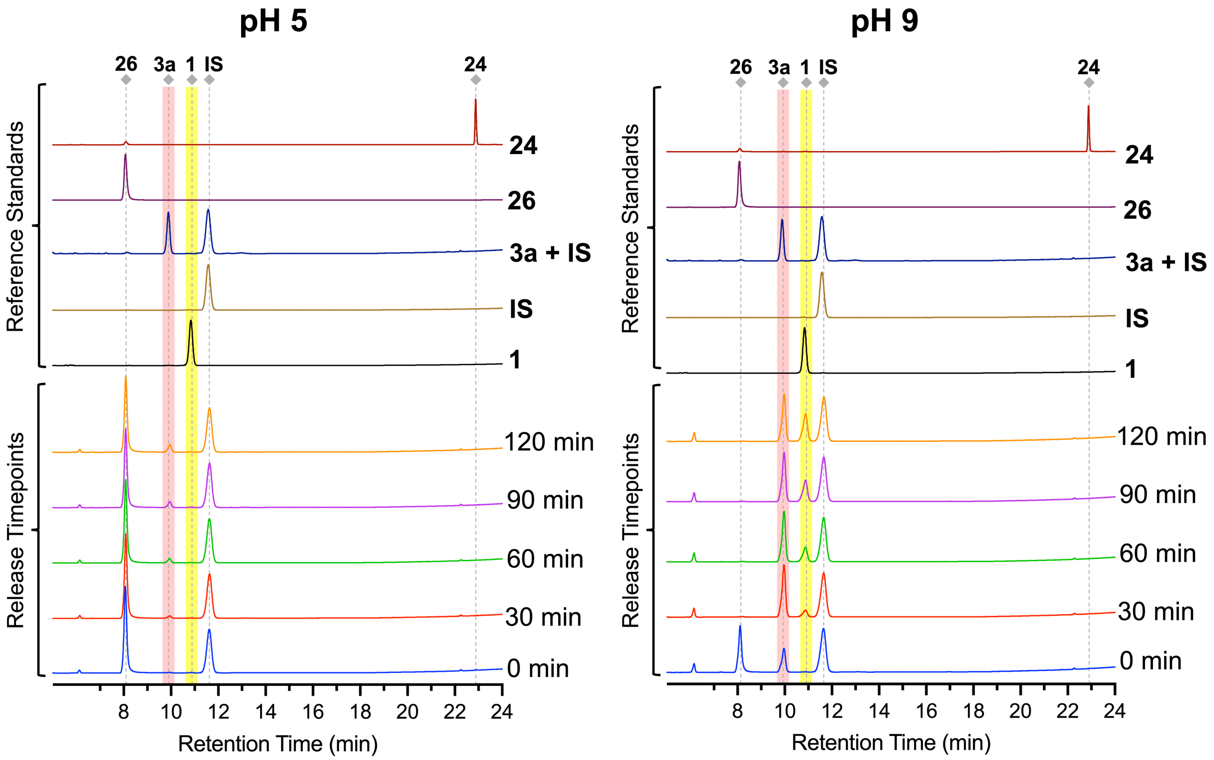

Figure S78. HPLC traces for 24 release experiments. The representative traces shown are one replicate of the triplicate experiments performed. The peak at 7.9 min was confirmed to be **26**. The peak at 9.9 min was confirmed to be **3a**. The peak at 10.8 min was confirmed to be **1**. The peak at 11.7 min was the warfarin IS. The peak at 22.4 min was confirmed to be **24**.

$$\mathbf{26} \overset{k_1}{\rightarrow} \mathbf{3a} \overset{k_2}{\rightarrow} \mathbf{1}$$

$$A \overset{k_1}{\rightarrow} I \overset{k_2}{\rightarrow} P$$

$$\frac{d[A]}{dt} = -k_1[A]$$

$$\frac{d[I]}{dt} = k_1[A] - k_2[I]$$

$$\frac{d[P]}{dt} = k_2[I]$$

Integrate [A] with respect to t:

$$[A] = [A]_0 e^{-k_1 t}$$

Plug the expression for [A] into the equation for d[I]/dt:

$$\frac{d[I]}{dt} = k_1[A]_0 e^{-k_1 t} - k_2[I]$$

Rearrange and integrate:

$$[I] = \frac{k_1[A]_0}{k_2 - k_1}(e^{-k_1 t} - e^{-k_2 t})$$

Plug the expression for [I] into the equation for d[P]/dt:

$$\frac{d[P]}{dt} = k_2\left(\frac{k_1[A]_0}{k_2 - k_1}(e^{-k_1 t} - e^{-k_2 t})\right)$$

Rearrange and integrate:

$$[P] = [A]_0\left[1 + \frac{1}{k_1 - k_2}(k_2 e^{-k_1 t} - k_1 e^{-k_2 t})\right]$$

If $k_1 >>> k_2$:

$$[P] \approx [A]_0\left[1 + \frac{1}{k_1}(-k_1 e^{-k_2 t})\right]$$

$$[P] \approx [A]_0[1 - e^{-k_2 t}]$$

Figure S79. Integrated Kinetic Model for Two Irreversible Consecutive Reactions. An expression for the production of [**1**] ([P]) can be derived from two-step consecutive reaction kinetic model. If the assumption is then applied that $k_1>>>k_2$, a simplified version of this expression can be written.[20] This is the equation for a first order process. As such, it can be linearized into the same form as shown in Figure S21. Experimental data were fitted to this simplified linear expression using GraphPad Prism 10 software to derive approximate k_2 values.

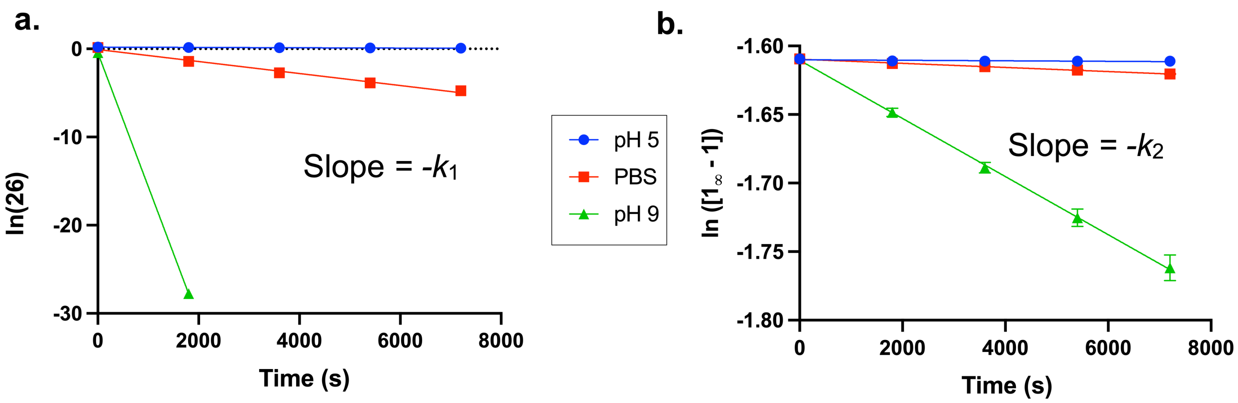

Figure S80. First order linearized kinetics graphs for the release of 1 from 24. (a) ln (**26**) vs time was plotted. Error bars represent ± SD for the triplicate experiments. For pH 9, **26** was consumed before the third time point. The slope of this graph was used to determine k_1. (b) ln (**1**$_\infty$-**1**) vs time was plotted using the concentrations of lapachone calculated from the peak area absorbances and the lapachone standard curve. The slope of this graph was used to calculate k_2. Error bars represent ± SD for the triplicate experiments.

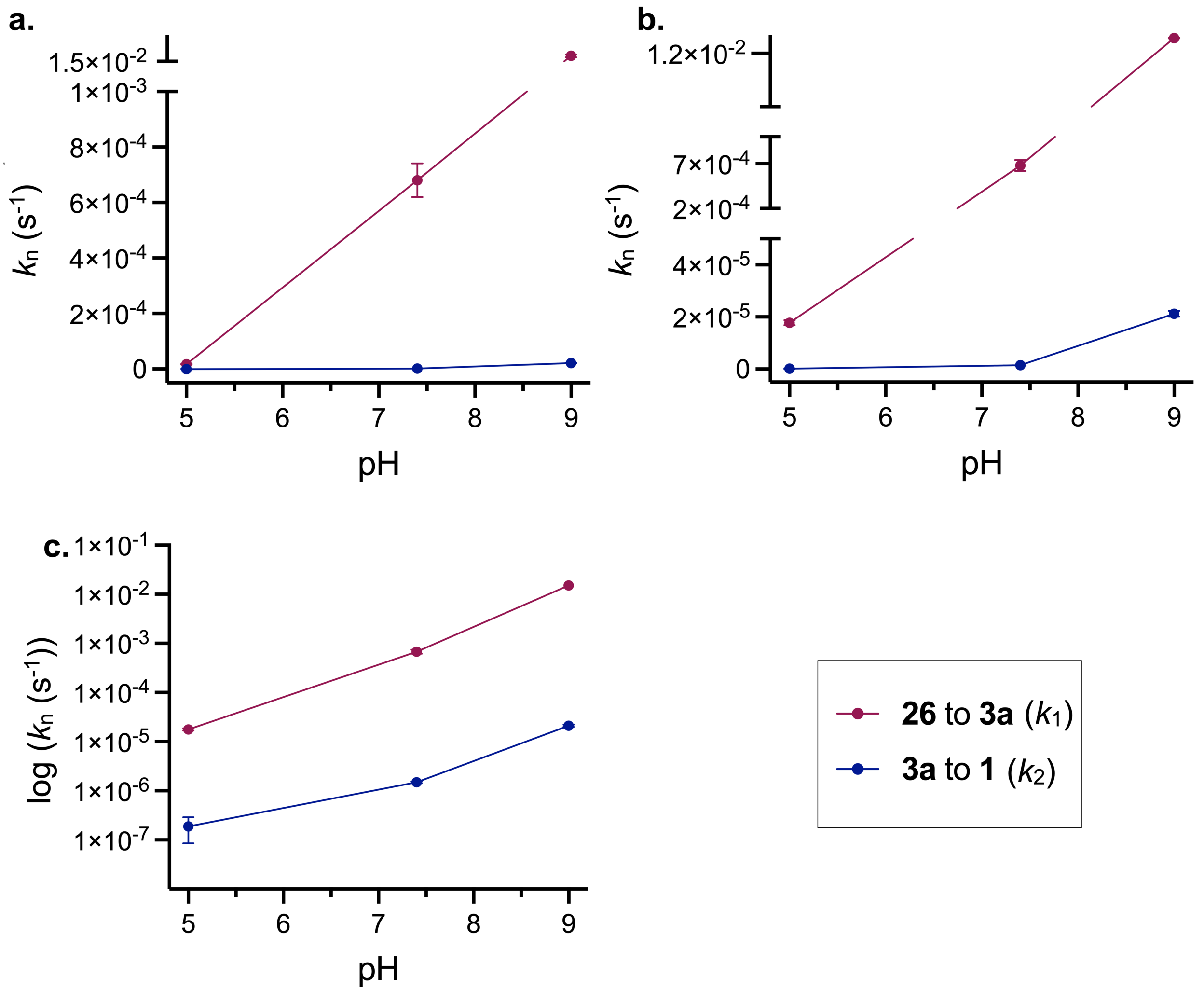

Figure S81. Comparison of k_1 and k_2. To confirm that the assumption that $k_1 >>> k_2$ is accurate, the calculated values of k_1 and k_2 were plotted. (a) These values were plotted with a partly zoomed y axis, (b) a more zoomed y axis (to see pH 5), and (c) in a logarithmic scale. The comparison confirmed that $k_1 >>> k_2$ for all three pHs studied, and thus applying the first order kinetics equations derived in Figure S21 and Figure S79 is suitable for this experiment. Error bars represent ± 95% CI. All k_{obs} values were calculated using GraphPad Prism 10.

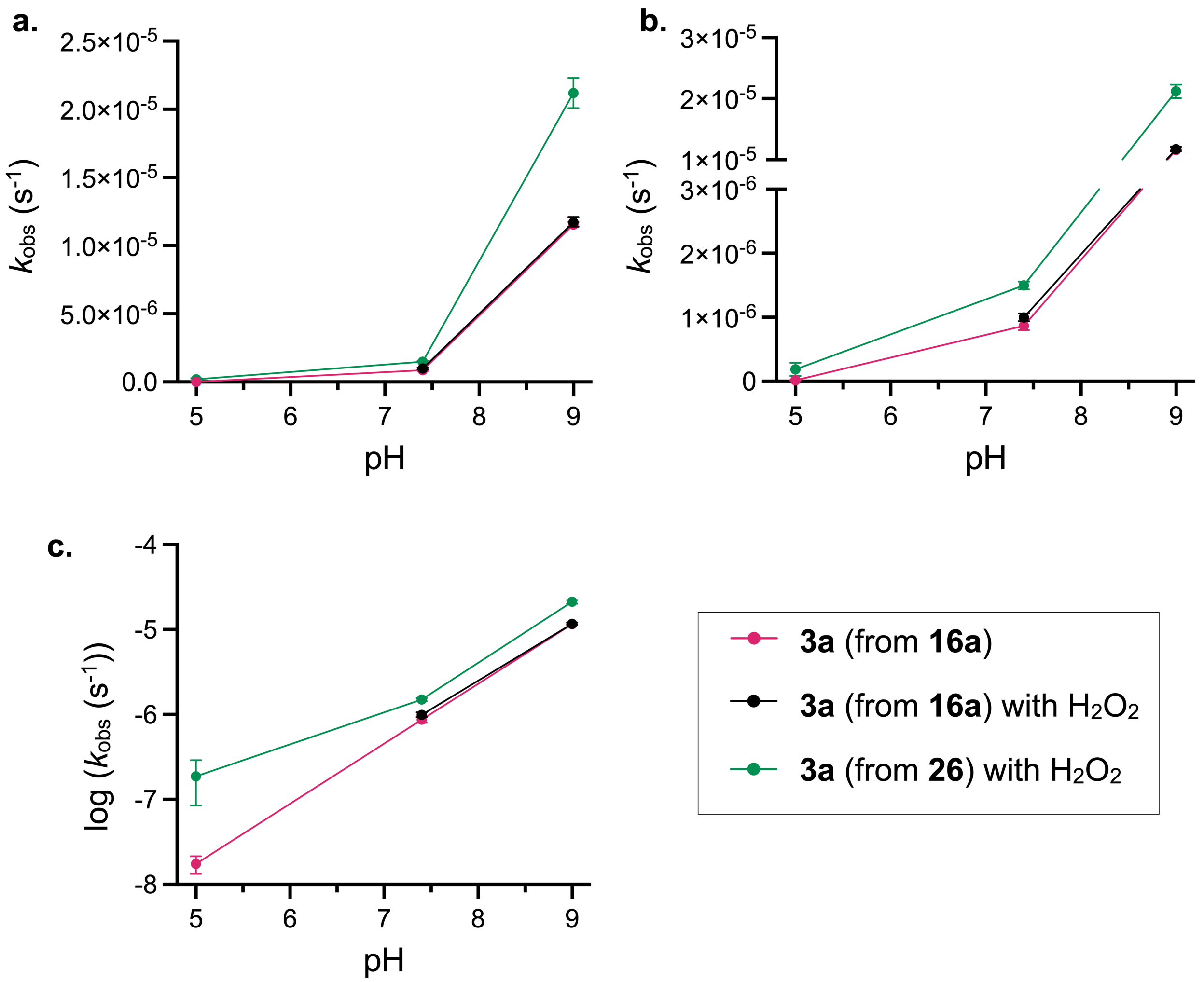

Figure S82. Comparison of lapachone release rates from boronate ester and glucuronide prodrugs. The derived values of the k_{obs} of lapachone release vs. pH were plotted for **3a** derived from the boronate prodrug **26** (teal) and compared to the release rate of lapachone from **3a** derived from the glucuronide prodrug **16a** with and without H_2O_2 (black and red respectively). (a) These values were plotted with a standard y axis, (b) a more zoomed y axis (to see pH 7.4 and 5), and (c) with a logarithmic scale. Error bars represent ± 95% CI. All k_{obs} values were calculated using GraphPad Prism 10.

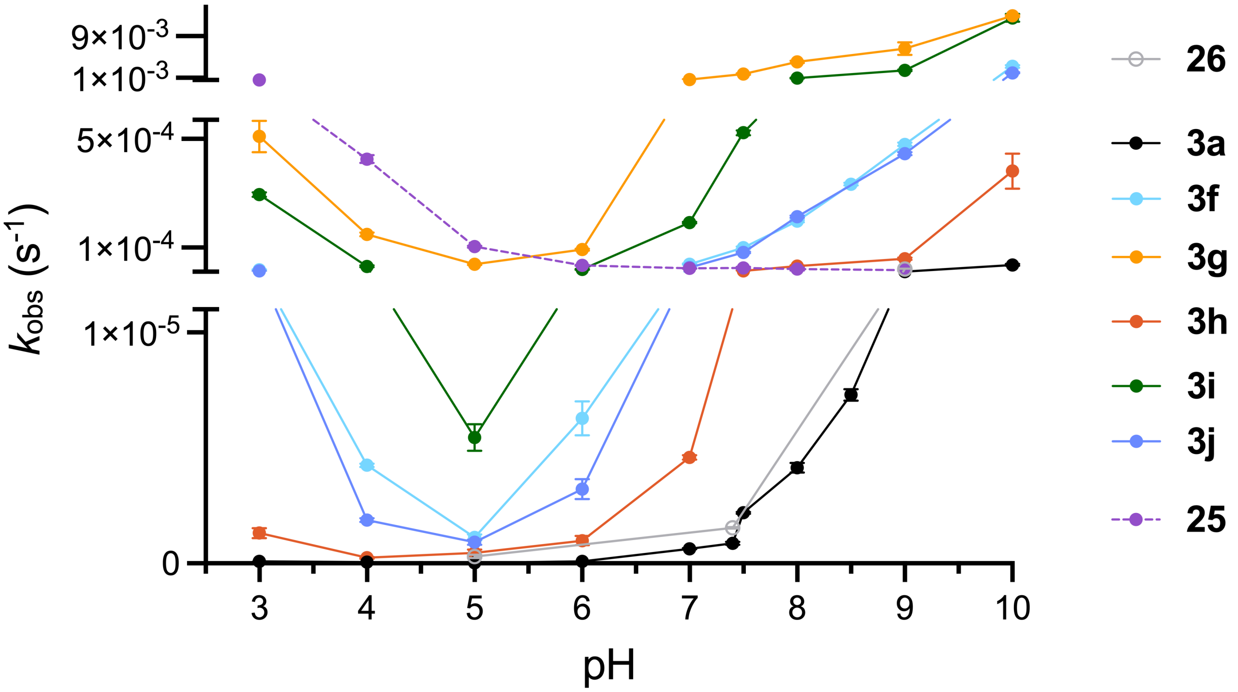

Figure S83. First Order Rate Constants for Improved PHB and PAB Derivatives Compared to Boronate Ester Prodrug. The calculated k_{obs} values for the improved PHB and PAB prodrugs, as well as **3a**, are plotted vs pH in comparison to the boronate prodrug. These values are shown plotted without a logarithmic scale, zoomed in such that the relative k_{obs} for all compounds from pH 3-7 can be examined. Error bars represent ± 95% CI.

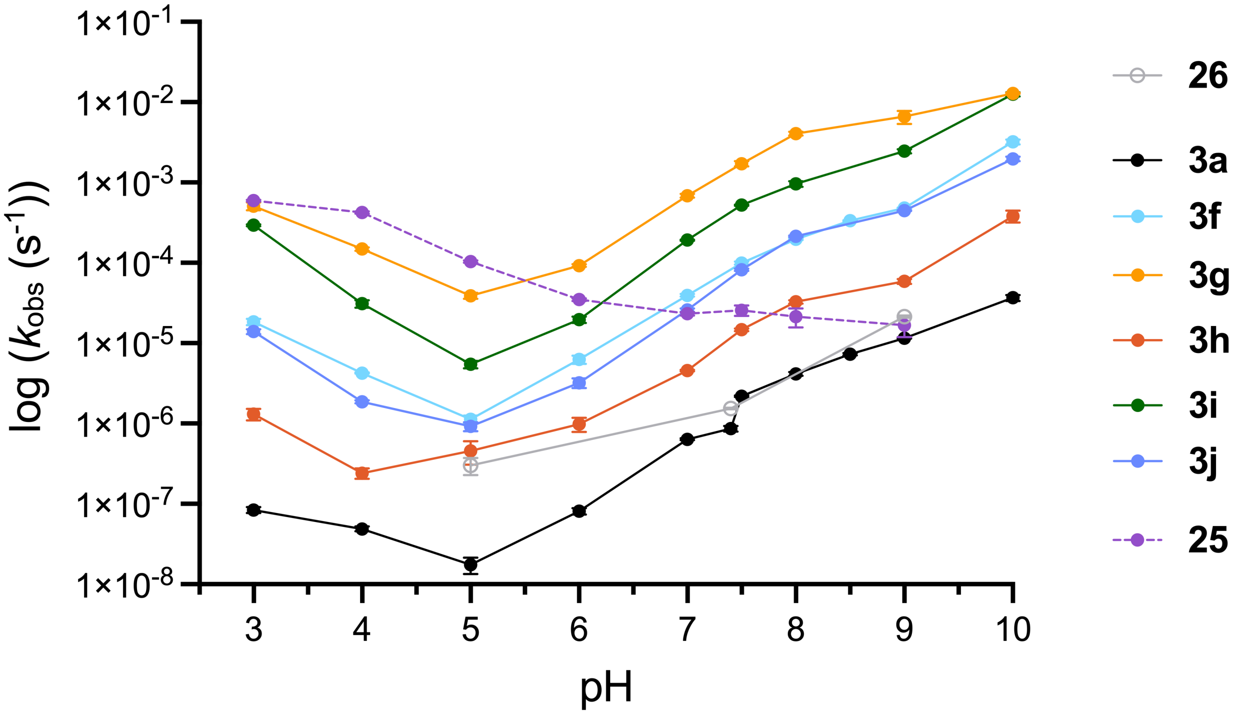

Figure S84. First Order Rate Constants for Improved PHB and PAB Derivatives Compared to Boronate Ester Prodrug. The calculated k_{obs} values for the improved PHB and PAB prodrugs, as well as **3a**, are plotted vs pH in comparison to the boronate prodrug. These values are shown plotted with a logarithmic scale. Error bars represent ± 95% CI.

7.2. LC-MS peak identification for boronate ester experiments.

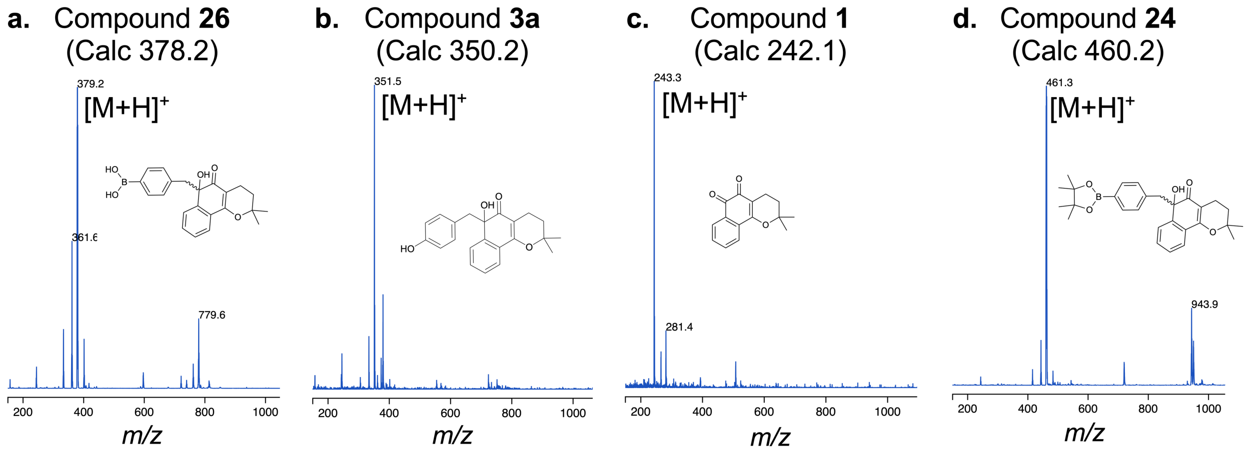

Figure S85. Peak identification by m/z for boronate ester experiments. After incubation with 5 equiv. of H_2O_2 at pH 7.4 for 30 min, each compound in the mixture was purified by semi-preparative HPLC. The analytical HPLC retention time of each isolated compound was established (Figure S78). Each isolated compound was then analyzed by LC-MS. The reported retention times (RT) correspond to those shown in Figure S78. (a) The peak at RT = 7.9 min was

confirmed to be **26**. (b) The peak at RT = 9.9 min was confirmed to be **3a**. (c) The peak at RT = 10.8 was confirmed to be **1**. (d) The identity of a reference standard in DMSO of **24** (RT = 22.4 min) was confirmed.

7.3. Stability of Boronate Prodrug

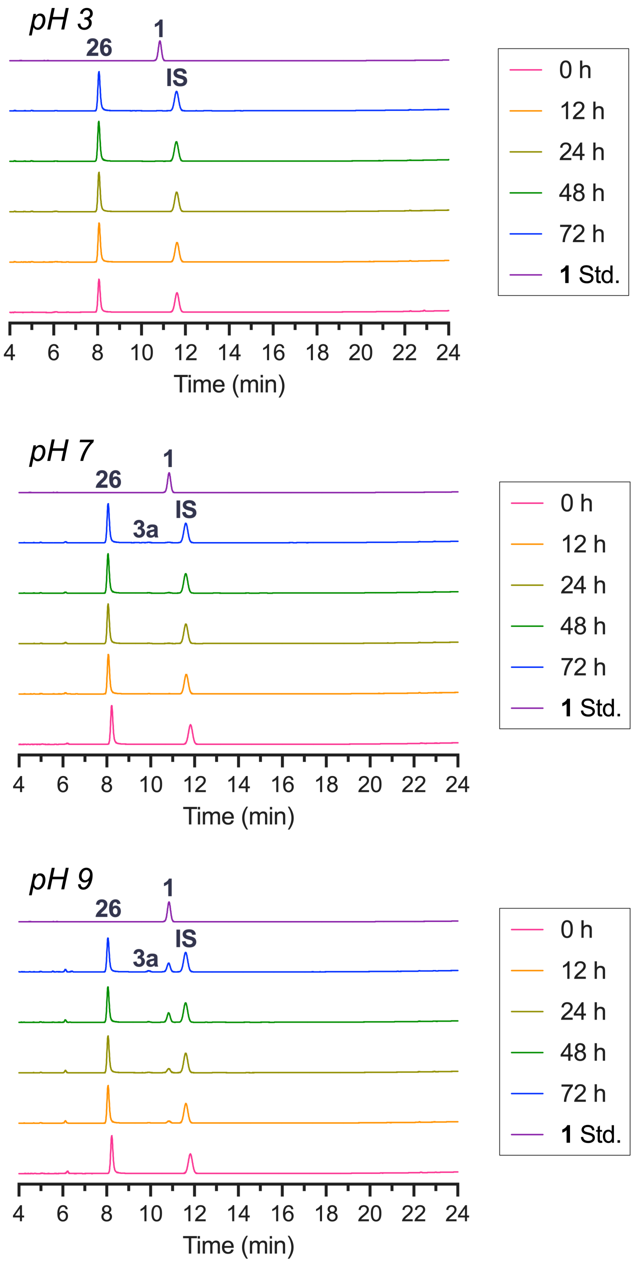

Figure S86. HPLC traces for 24 (26) stability experiments. The representative traces shown are one replicate of the triplicate experiments performed. Minute lapachone and **3a** peaks were detected in some samples. RT **24** = 22.8 min, RT **26** = 7.9 min, RT **1** = 10.8 min, RT **3a** = 9.9 min RT warfarin IS 11.6 = min.

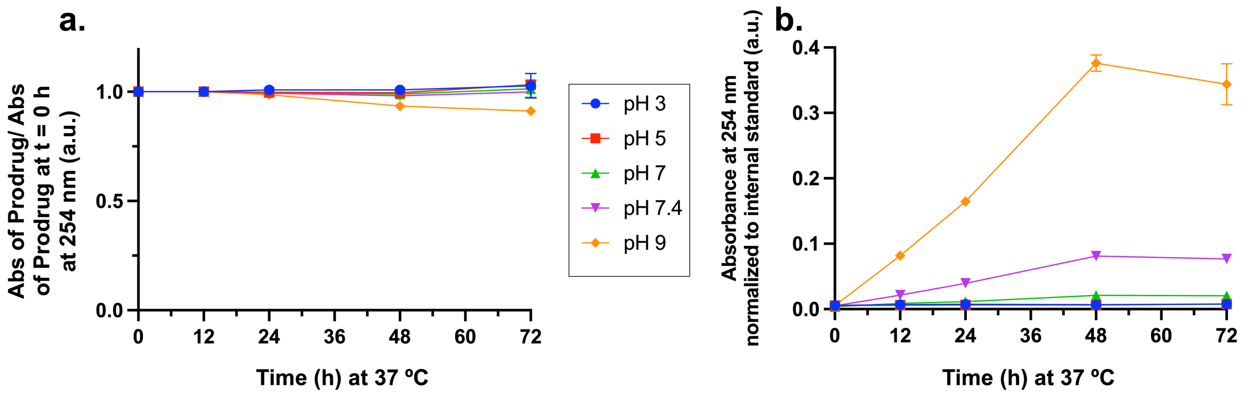

Figure S87. Prodrug peak area of 26. (a) The peak area of **26** divided by the starting peak area of **26** is plotted for each pH. No changes in the peak area were detected over the 72 h incubation period for pH 3-7.4, but a substantial decrease in the peak area was detected for pH 9. (b) The area of the β-lapachone peak is plotted over time. The peak remained small for pH 3-5, but increased substantially over time for pH 7-9. Error bars represent ± SD of the triplicate experiments.

Section 8. Supplemental figures for *in vitro* PDAC cellular efficacy

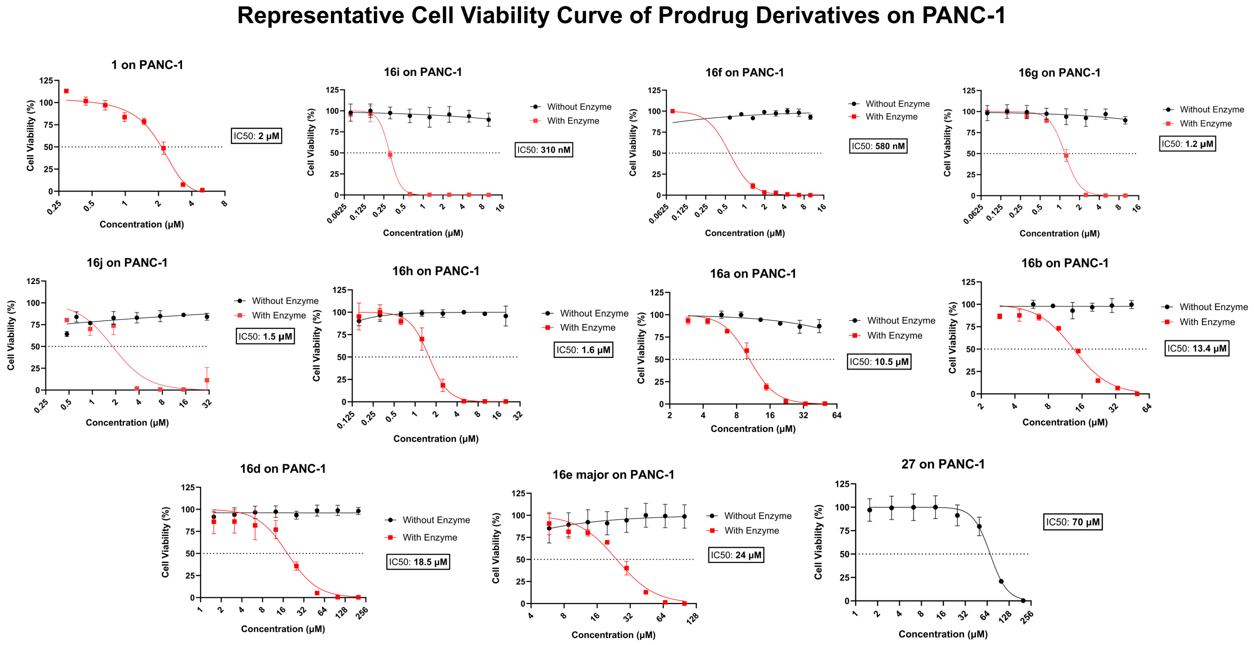

Figure S88. Representative Cell Viability Curves of Prodrug Derivatives on PANC-1 Cell Line. PANC-1 PDAC cell line viability with prodrug treatment. Representative PANC-1 cell viability data of all β-lapachone prodrug derivatives after 72 h treatment with or without exogenous β-glucuronidase added. The IC_{50}'s are listed for the representative curve and the error bars represent the SD of three technical replicates.

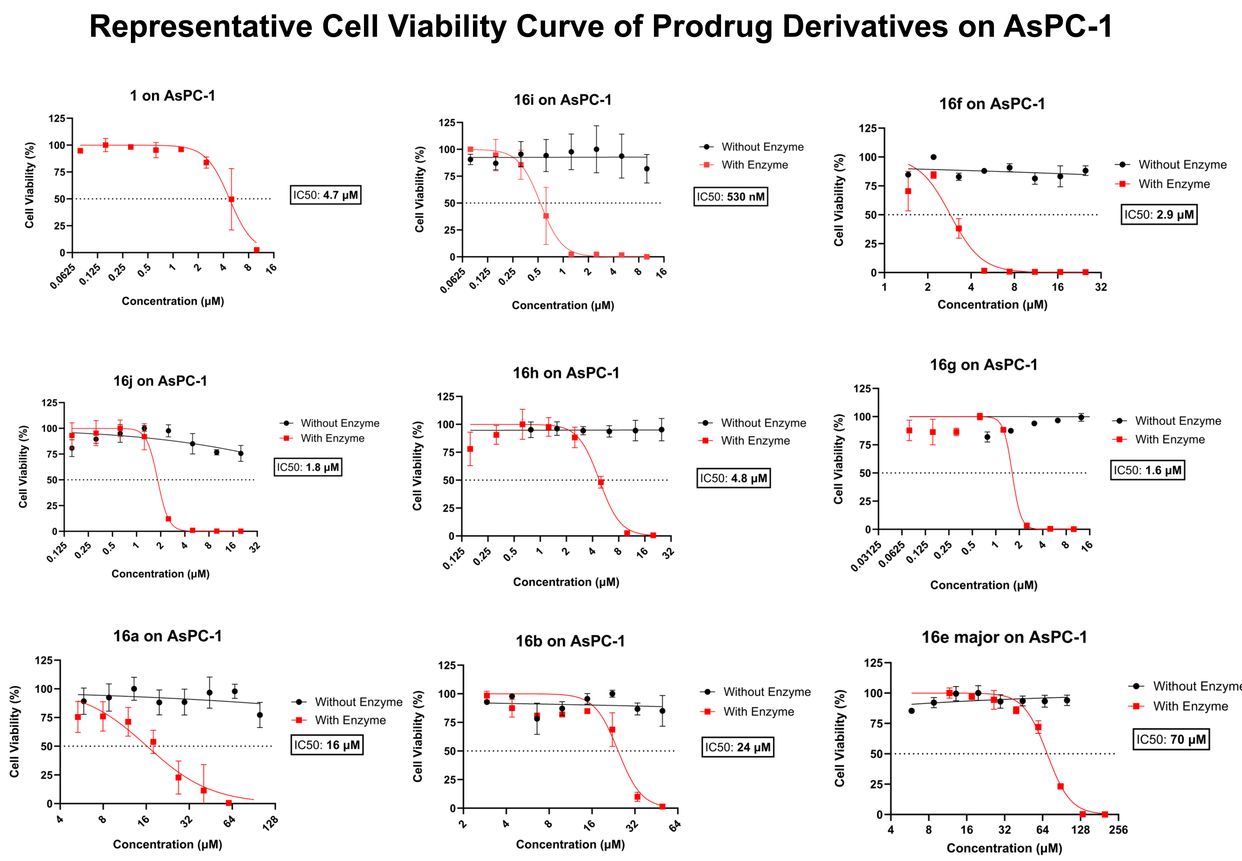

Figure S89. Representative Cell Viability Curves of Prodrug Derivatives on AsPC-1 Cell Line. AsPC-1 PDAC cell line viability with prodrug treatment. Representative data of all β-lapachone prodrug derivatives after 72 h treatment with or without exogenous β-glucuronidase added. The IC_{50}'s are listed for the representative curve and the error bars represent the SD of three technical replicates.

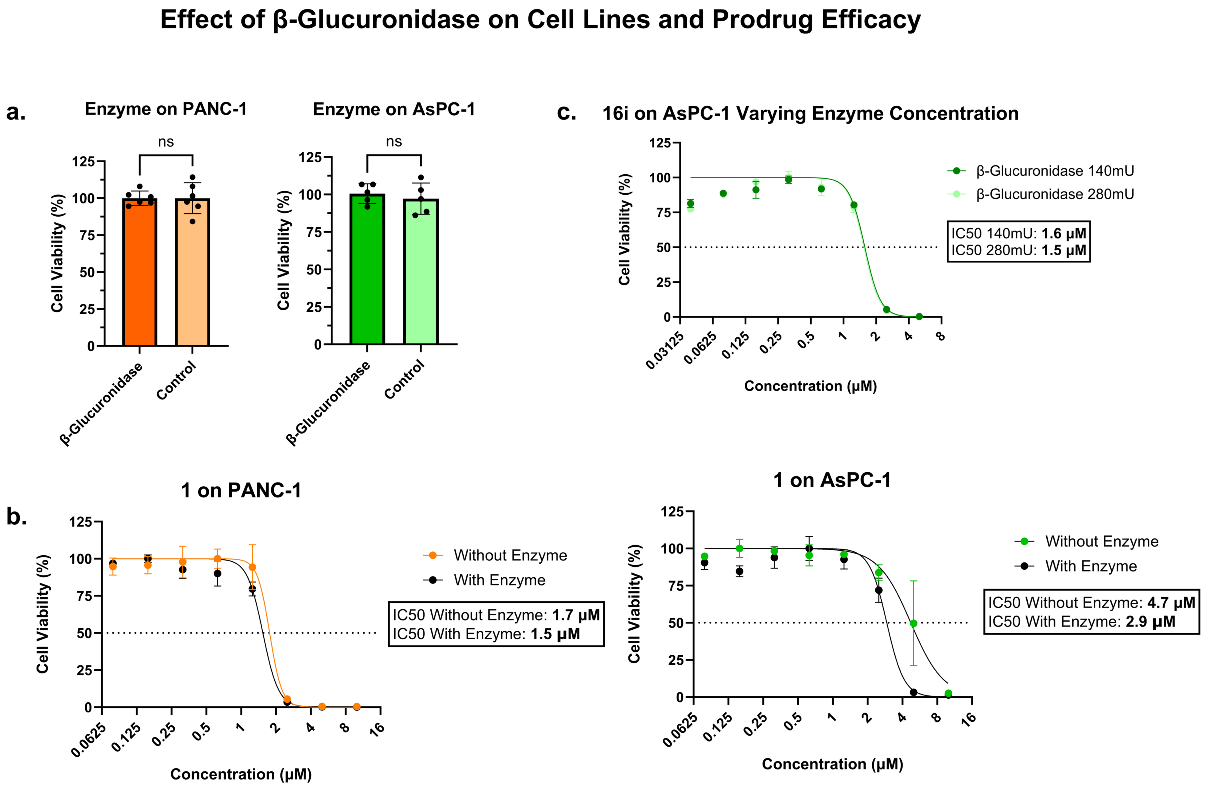

Figure S90. Effect of β-Glucuronidase on Cell Lines and Prodrug Efficacy. (a) AsPC-1 and PANC-1 cell viability after 72 h incubation with 140mU (1 μL) of only β-glucuronidase enzyme, compared to a without enzyme control. (b) PANC-1 and AsPC-1 cell viability curves of β-lapachone with and without β-glucuronidase enzyme. (c) AsPC-1 cell viability after 72 h incubation with varied β-glucuronidase enzyme concentration of prodrug **16i**. No statistically significant differences are seen. For all *in vitro* assays, 1 μL of sterile β-glucuronidase enzyme (from *E. coli* K-12 Roche #03707580001, 140 U/mL) was used in order to practically conduct these experiments. In an *in vivo* setting, tumors have heterogenous β-glucuronidase expression, so the exogenous β-glucuronidase in these assays is not meant reflect the exact enzyme amounts that may be present in any one tumor.

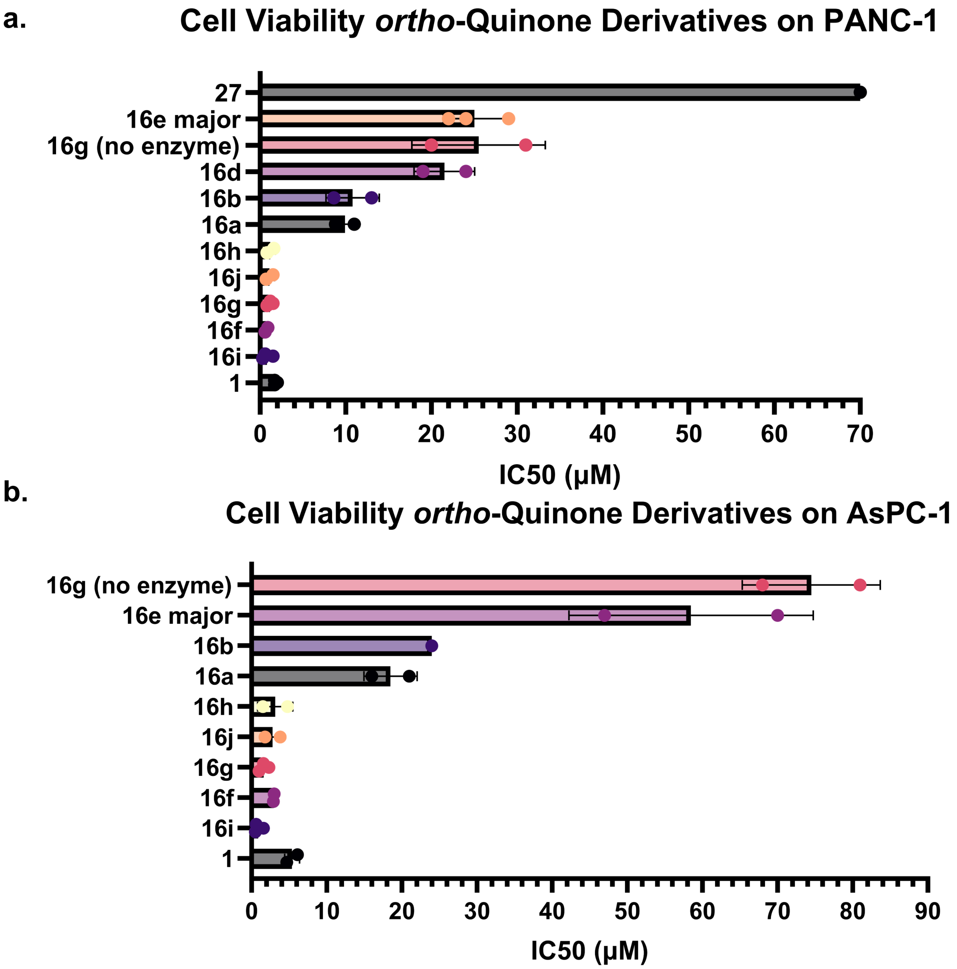

Figure S91. PDAC Cell Viability with Prodrug Treatment for All Derivatives. Mean IC_{50} values ± standard deviation of at least two biological replicates for each prodrug on the PANC-1 (a) and AsPC-1 (b) cell lines are shown. All derivatives were tested on the PANC-1 cell line, and those that performed better than **16a** were prioritized for testing in the AsPC-1 cell line. Values with no error bars represent only one biological replicate.

Table 8.1. Raw IC_{50} Values of Biological Replicates in Figure S91 (µM).

Derivative	PANC-1 Replicate 1	PANC-1 Replicate 2	PANC-1 Replicate 3	AsPC-1 Replicate 1	AsPC-1 Replicate 2	AsPC-1 Replicate 3
**1**	1.7	2	1.7	4.7	6.1	-
**16i**	0.3	1.5	0.6	0.5	0.6	1.6
**16f**	0.6	0.9	-	2.9	3	-
**16g**	1.2	1.5	0.8	2.3	1.6	1
**16j**	0.7	1.5	-	1.8	3.8	-
**16h**	1.6	0.8	-	4.8	1.5	-
**16a**	8.8	11	-	21	16	-
**16b**	8.6	13	-	24	-	-
**16d**	19	24	-	-	-	-
**16g No Enzyme**	20	31	-	68	81	-
**16e Major Product**	29	24	22	47	70	-
**27**	70	-	-	-	-	-

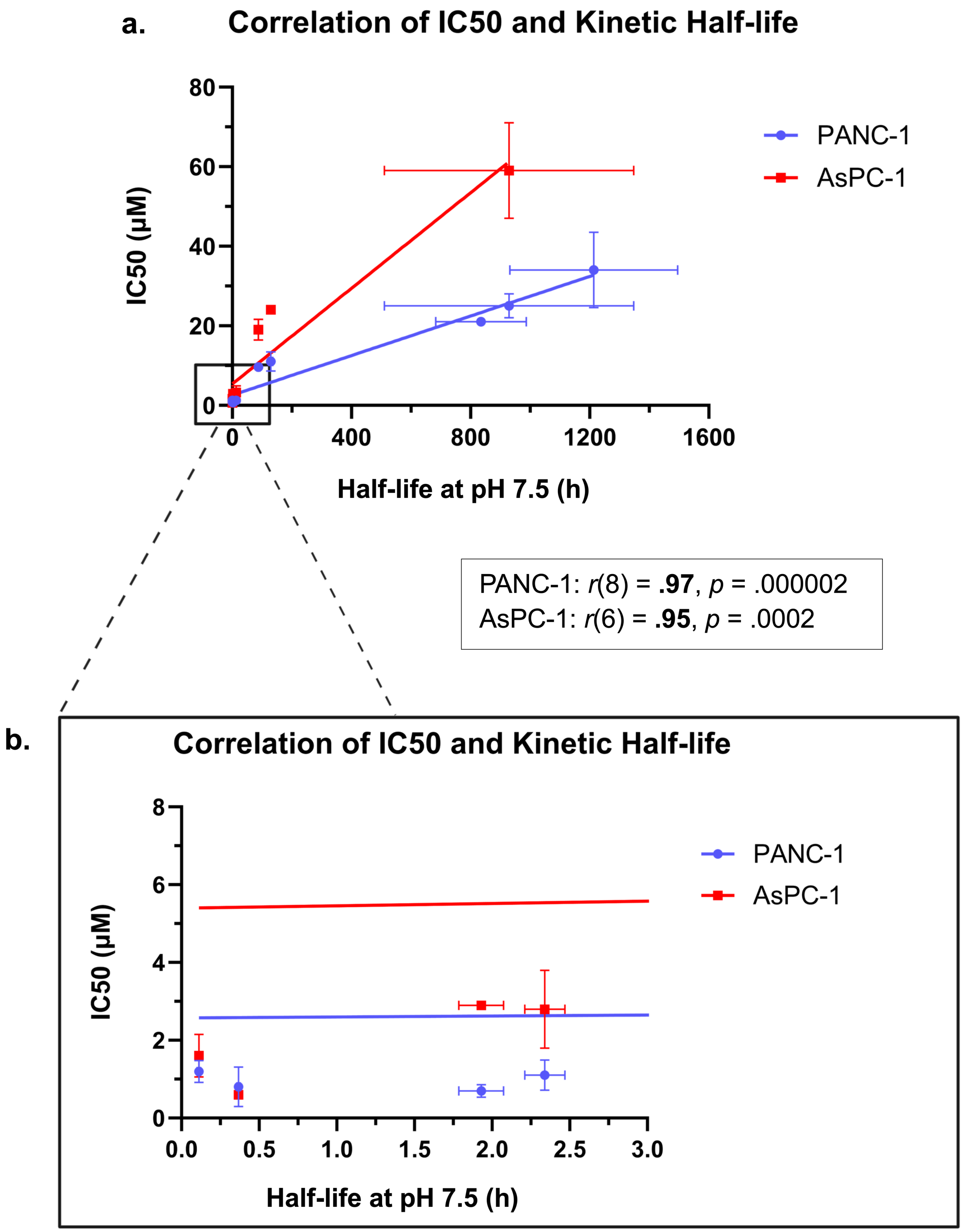

Figure S92. Correlation of IC_{50} and Kinetic Half-Life for Prodrug Derivatives. Mean half-life values at pH 7.5 ± standard deviation calculated from 95% CI compared against IC_{50} values ± standard deviation of at least two biological replicates for all prodrug derivatives. Panel (a) shows the full range of derivatives and panel (b) shows data points close to the origin with adjusted axes for clarity. A Pearson correlation coefficient was calculated from this trend and is stated.

Section 9. Supplemental figures for cellular mechanism of prodrug

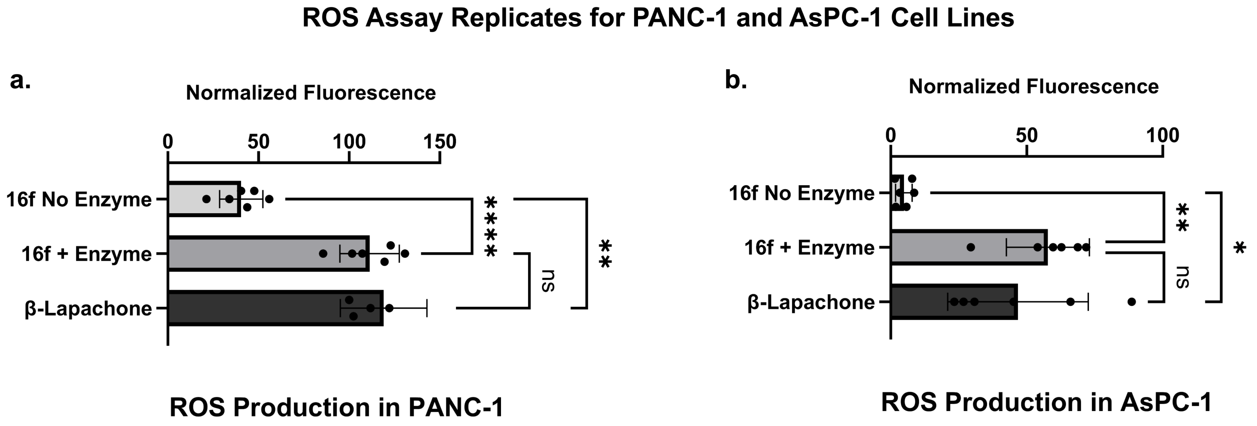

Figure S93. ROS Assay Replicate on PANC-1 and AsPC-1 Cell Lines. Biological replicate of reactive oxygen species generation in the PANC-1 (a) and AsPC-1 (b) cell lines after 24 h treatment with **16f** with and without β-glucuronidase enzyme, compared to β-lapachone. Results were normalized to baseline cellular ROS activity as 0 and positive control *tert*-butyl hydrogen peroxide (TBHP) as 100. Adjusted *p*-values of < 0.0284 (*), < 0.0015 (**), and < 0.0001 (****) are shown for statistically significant comparisons, calculated using GraphPad Prism 10 software.

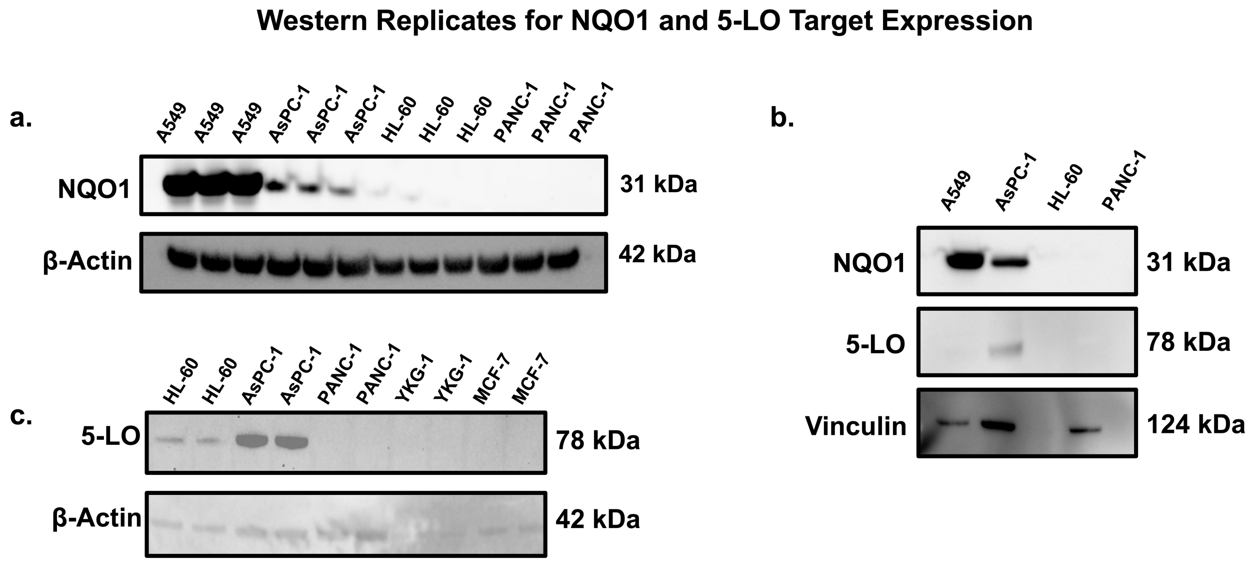

Figure S94. Western Blot Replicate for NQO1 and 5-LO Targets on PANC-1 and AsPC-1 Cell Lysates. Panel (a) shows replicate 1 western blot bands of NQO1 expression from whole cell lysates of PANC-1 and AsPC-1 and β-Actin as a loading control. Panel (b) shows replicate 2 western blot bands of NQO1 and 5-LO expression from whole cell lysates of PANC-1 and AsPC-1 and Vinculin as a loading control. Panel (c) shows replicate 1 western blot bands of 5-LO expression from whole cell lysates of PANC-1 and AsPC-1 and β-Actin as a loading control. The A549 cell line was used as a positive control for NQO1 enzyme expression, and the HL-60 cell

line was used as a positive control for 5-LO enzyme expression. The YKG-1 and MCF-7 cell lines were used as negative controls.

a.

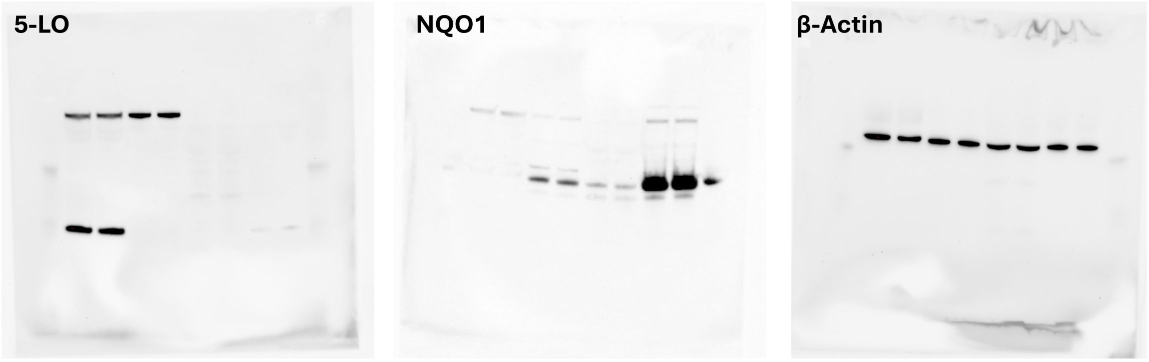

b.

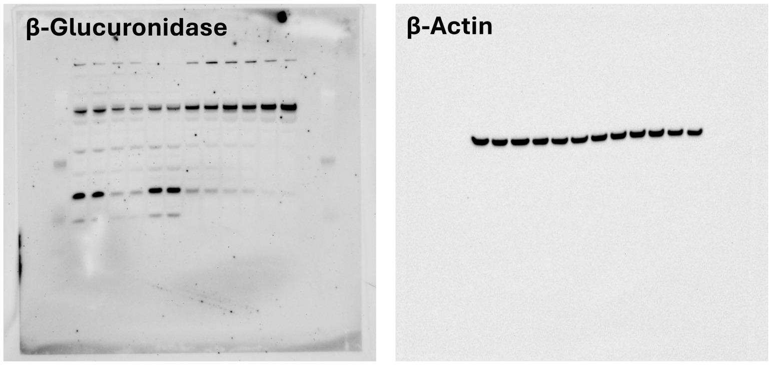

Figure S95. Full Blots for Representative Westerns in Figure 8a. Panel row (a) shows full blots for representative western blot bands of NQO1 and 5-LO expression from Figure 8a. Panel row (b) shows full blots for representative western blot bands of β-glucuronidase expression from Figure 8a.

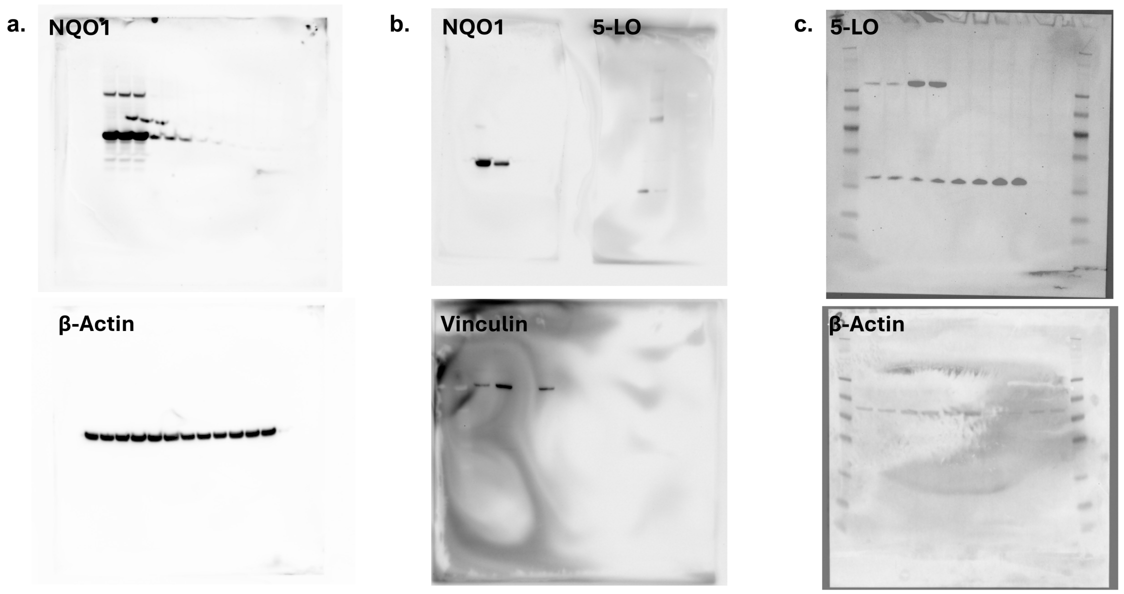

Figure S96. Full Blots for Replicate Westerns in Figure S94. Panel column (a) shows full blots for replicate western blot bands of NQO1 expression from Figure S94a. Panel column (b) shows full blots for replicate western blot bands of NQO1 and 5-LO expression from Figure S94b. Panel column (c) shows full blots for replicate western blot bands of 5-LO expression from Figure S94c.

Section 10. Materials and Methods

10.1. HPLC Prodrug Stability Assays

β-Glucuronide prodrug was dissolved in DMSO to a concentration of 10 mM. A 100 mM stock solution of warfarin internal standard in DMSO was also prepared. A stock solution was prepared with prodrug (49.7 μL of 10 mM DMSO stock) and warfarin (5 μL of 100 mM DMSO stock) in glucuronidase buffer (255.3 μL of 75 mM potassium phosphate buffer, pH 6.8). This resulted in stock concentrations of 1.6 mM prodrug and 1.6 mM warfarin internal standard in 310 μL total volume with 17.6% DMSO. For the less soluble **22**, **24**, and **27**, 255.3 μL of DMSO was added in place of the glucuronidase buffer. For a reaction, 20 μL of the stock solution was added to 130 μL of citrate-phosphate buffer at the specified pH and incubated at 37 °C. For **22**, **24**, and **27**, 20 μL of the stock solution was added to 75 μL of citrate-phosphate buffer at the specified pH and 55 μL DMSO to give a 50% DMSO solution. At the recorded time points, 20 μL aliquots of each sample were flash frozen and thawed immediately before injection into the HPLC (254 nm, Agilent 1260 Infinity) for analysis.

HPLC conditions: column Agilent InfinityLab Poroshell 120 SB-C18 2.7 μm, 50 × 4.6 mm; column compartment = 30 °C; solvent system A = H_2O + 0.1% TFA, B = acetonitrile + 0.1% TFA; flow = 1 mL/min. Gradient for **16a-j**: t = 0–1 min 5% B, t = 1–8 min 5–30% B, t = 8–23 min 30–40% B, t = 23–23.1 min 40–100% B, t = 23.1–24 min 100–100% B, t = 24–29 min 5% B. Gradient for **22**, **24/26** and **27**: t = 0–1 min 5% B, t = 1–2 min 5–30% B, t = 2–17 min 30–40% B, t = 17–24 min 40–90% B, t = 24–24.1 min 90–100% B, t = 24.1–25 min 100–100% B, t = 25–30 min 5% B. Peak assignments were confirmed by retention time comparison to known standard solutions. Relative peak area represents raw peak area (mAU*s) normalized to raw peak area of the warfarin internal standard. Lapachone release was quantified using the lapachone raw peak area and a lapachone standard curve. Values recorded are averages of three independent reactions and error bars display standard deviation (SD).

10.2. HPLC Drug Release Kinetics Assays

β-Glucuronide prodrug was dissolved in DMSO to a concentration of 10 mM. A 100 mM stock solution of warfarin internal standard in DMSO was also prepared. To obtain deprotected *para*-hydroxybenzyl ketol species **3**, a concentrated stock solution was prepared combining the prodrug **16** (83.4 μL of the 10mM stock), warfarin (8.6 μL of the 100 mM stock), and glucuronidase buffer (398 μL of 75 mM potassium phosphate buffer, pH 6.8). To this, 30 μL of enzyme (β-glucuronidase from *E. coli* K-12 Roche #03707580001, 140 U/mL) was added. This resulted in stock concentrations of 1.6 mM prodrug and 1.6 mM warfarin internal standard in 520 μL total volume with 17.6% DMSO. The stock was incubated at 37°C with shaking for 10-20 min until LC-MS analysis indicated complete removal of the glucuronide moiety from the prodrug. Reactions were then set up immediately. For a reaction, 20 μL of the hydroxybenzyl ketol / internal standard stock was added to 130 μL of citrate-phosphate buffer at the specified

pH, resulting in a reaction concentration of 0.21 mM prodrug and 2.3% DMSO. The reactions were incubated in either a 37 °C water bath or a 37 °C shaking air incubator. At the recorded time points, 20 μL aliquots of the reactions were analysed by HPLC (254 nm, Agilent 1260 Infinity). Depending on the speed of the release, the samples were either analyzed live time as the release reaction proceeded, or flash frozen in liquid nitrogen and then thawed before injection.

For those compounds (**3i**, **3g**) that were extremely fast releasing, instead of one large deprotected stock of **3** being made via enzyme addition, each sample was prepared separately. A stock of prodrug (93 μL of the 10 mM stock) and warfarin (9.3 μL of the 100 mM stock) in glucuronidase buffer (477.7 μL of 75 mM potassium phosphate buffer, pH 6.8) was prepared. This resulted in stock concentrations of 1.6 mM prodrug and 1.6 mM warfarin internal standard in 580 μL total volume with 17.6% DMSO. For each reaction, 20 μL of this stock was warmed to 37°C and 5 μL of enzyme (β-glucuronidase from *E. coli* K-12 Roche #03707580001, 140 U/mL) was added. This was incubated for two min at 37°C with mixing to ensure total removal of the glucuronide sugar moiety from the prodrug. Then 125 μL of citrate-phosphate buffer at the specified pH was added to the vial and mixed well, resulting in a reaction concentration of 0.21 mM prodrug and 2.3% DMSO. The reactions were incubated in a 37 °C water bath. At the recorded time points, 20 μL aliquots of the reactions were analysed by HPLC (254 nm, Agilent 1260 Infinity). Depending on the speed of the release, the samples were either analyzed live time as the release reaction proceeded, or flash frozen in liquid nitrogen and then thawed before injection.

For the H_2O_2 assays performed with **24/26** and **3a**, a commercial 50% wt/v solution of H_2O_2 (17.6 M) was diluted into citrate-phosphate buffer of the desired pH to a concentration of 1.15 mM H_2O_2. This peroxide buffer solution was freshly prepared immediately before each reaction to ensure minimal degradation of the peroxide. A stock of prodrug (30 μL of 10 mM DMSO stock) and warfarin (3 μL of 100 mM DMSO stock) in glucuronidase buffer (100 μL of 75 mM potassium phosphate buffer, pH 6.8) and DMSO (67 μL) was also prepared. This resulted in stock concentrations of 1.5 mM prodrug and 1.5 mM warfarin internal standard in 200 μL total volume with 50% DMSO. Lower DMSO concentrations proved insufficient at dissolving **24**. For **3a**, 47 μL of glucuronidase buffer and 20 μL of enzyme were added in place of the 67 μL of DMSO, and incubated at 37 °C for 15 min until removal of the glucuronide moiety was complete. For each reaction, 20 μL of this stock was dissolved into 130 μL of the peroxide citrate-phosphate buffer, resulting in 0.2 mM prodrug, 0.2 mM warfarin, and 1 mM hydrogen peroxide with 6.7% DMSO. The ratio of prodrug to peroxide was 1:5 in each reaction. The reactions were incubated at 37 °C in a water bath and 20 μL aliquots were injected into the HPLC for analysis at the designated time points. To confirm peak assignments, two samples were incubated using the peroxide conditions for 5 or 30 min. They were then purified using semi-preparative HPLC to isolate **26**, **3a**, and **1** (semi-prep conditions described in synthetic

methods). These isolated compounds were then analyzed by LC-MS and NMR (^{1}H, ^{13}C, COSY, DEPT, HSQC, HMBC) to confirm their identities and structures.

HPLC conditions: column Agilent InfinityLab Poroshell 120 SB-C18 2.7 μm, 50 × 4.6 mm; column compartment = 30 °C; solvent system A = H_2O + 0.1% TFA, B = acetonitrile + 0.1% TFA; flow = 1 mL/min. Gradient for **3a**, **3b**, **3d**, **3g**, **3h**, **3i**, **24/26**: t = 0–1 min 0% B, t = 1–2 min 0–30% B, t = 2–17 min 30–40% B, t = 17–17.1 min 40-100% B, t = 17.1–18 min 100% B, t = 18–23 min 0% B. Gradient for **25**: t = 0–1 min 0% B, t = 1–2 min 0–30% B, t = 2–20 min 30–40% B, t = 20–20.1 min 40-100% B, t = 20.1–22 min 100% B, t = 22–27 min 0% B. Gradient for **3c**: t = 0–1 min 5% B, t = 1–2 min 5–30% B, t = 2–17 min 30–40% B, t = 17–24 min 40–90% B, t = 24–24.1 min 90–100% B, t = 24.1–25 min 100–100% B, t = 25–30 min 5% B. Gradient for **3j**: t = 0–1 min 0% B, t = 1–2 min 0–25% B, t = 2–24 min 25–35% B, t = 24–24.1 min 35–100% B, t = 24.1–25 min 100–100% B, t = 25–30 min 0% B, column compartment = 45 °C. Gradient for **3e**: t = 0–1 min 0% B, t = 1–2 min 0–30% B, t = 2–20 min 30–40% B, t = 20–23 min 40–45% B, t = 23–23.1 min 45–100% B, t = 23.1–25 min 100–100% B, t = 25–30 min 0–0% B. Gradient for **3f**: t = 0–1 min 5% B, t = 1–2 min 5–30% B, t = 2–20 min 30–30% B, t = 20–20.1 min 30–100% B, t = 20.1–22 min 100–100% B, t = 22–27 min 0–0% B. Peak assignments were confirmed by retention time comparison to known standard solutions. Relative peak area represents raw peak area (mAU*s) normalized to raw peak area of the warfarin internal standard. Lapachone release was quantified using the lapachone raw peak area and a lapachone standard curve. Values recorded are averages of three independent reactions and error bars display standard deviation (SD). Values were fitted to first-order kinetics using GraphPad Prism 10 software, with the 95% profile likelihood confidence interval calculated and graphed as the error bars for each slope. A positive control experiment was performed under identical conditions with known substrate 4-nitrophenyl-β-D-glucuronide (Sigma Aldrich) to confirm enzyme activity.

10.3. Cell Viability Assays

Cells were cultured in DMEM or RPMI media (according to ATCC guidelines), supplemented with 10% FBS, 5% L-glutamine, and 5% Pen-Strep antibiotics. 10,000 cells were plated in clear tissue culture treated plates for adhesion at 37 °C, 5% CO_2 overnight. Compounds were filtered with 0.22 μm syringe filters. Stock solutions of appropriate concentrations were made with respective cell media and serial dilutions were prepared, with DMSO levels not exceeding 0.1%. 1 μL of sterile β-glucuronidase enzyme (from *E. coli* K-12 Roche #03707580001, 140 U/mL) was added to treatment wells at the time of compound treatment. This enzyme amount was used in order to practically conduct these experiments. In an *in vivo* setting, tumors have heterogenous β-glucuronidase expression, so the exogenous β-glucuronidase in these assays is not meant reflect the exact enzyme amounts that may be present in any one tumor. Control wells of cells included 0.1% DMSO, 1 μL β-glucuronidase enzyme, or β-lapachone at 10 μM. Experiments were also conducted without β-glucuronidase present to exhibit the therapeutic window and toxicity of the compounds. Cells were treated with

compounds for 72 h at 37 °C incubation. After 72 h, cells were treated with CellTiter-Glo® Luminescence Viability Assay solution shaking on an orbital shaker for 15 min. Wells were then transferred to a white 96-well plate for luminescence reading in a plate reader. Values were normalized with the positive β-lapachone control set to 0, and largest control values recorded (0.1% DMSO or 0.1% DMSO with enzyme) set to 100. Graphs were plotted in GraphPad Prism 10.

10.4. Reactive Oxygen Species Assays

Cells were cultured in DMEM or RPMI media (according to ATCC guidelines), supplemented with 10% FBS, 5% L-glutamine, and 5% Pen-Strep antibiotics. 10,000 cells were plated in clear tissue culture treated plates for adhesion at 37 °C, 5% CO_2 overnight. To measure ROS levels, a DCFDA ROS assay kit (Abcam: ab113851) was used. Media was removed from the wells and 100 µL of 1x buffer was added to wash. Buffer was removed and 100 µL of 20 µM DCFDA in 1x buffer was added to cells which were left to incubate at 37 °C for 45 min. After incubation, wells were washed with 100 µL of 1x buffer. Next, cells were treated with 100 µL of 0.22 µm syringe-filtered compounds at appropriate concentrations. Treatment included nothing, 50 µM prodrug with and without enzyme, 50 µM of β-lapachone, and 50 µM of sterile *tert*-butyl hydrogen peroxide as a positive control. The concentration of TBHP was optimized to each cell line and ranged from 100 µM – 2 mM. The treatment was for 24hr - 48hr at 37 °C incubation. After incubation, plates were read on the plate reader at excitation 485 nm and emission 535 nm. Values were normalized with 0 set to baseline ROS levels for the cell line (no treatment) and 100 set to the TBHP positive control. Results were graphed and analyzed statistically in GraphPad Prism 10.

10.5. Western Blot for Target Expression

About 300,000 cells in a pellet were resuspended in RIPA lysis buffer supplemented with EDTA and protease inhibitor. Lysate was kept on ice and protein levels were quantified using the Pierce 660 protein assay. A standard curve was made using known amounts of BA protein, serially diluted. The standards and sample were plated in duplicates in a clear 96-well plate. The Pierce 660 reagent was added, the plate was covered in aluminium foil, and put on the rocker for 5 min before absorbance was measured on the plate reader at 660 nm. The standard values were used to make a standard curve in Excel and concentrations were found for sample values from the standard equation. The sample with the lowest concentration was loaded to the maximum possible volume depending on gel size. The other samples were normalized to the least concentrated sample such that the final protein concentrations of all samples were the same. 4-12% gradient gels were loaded with 25% loading buffer dye, 10% reducing buffer, and the remaining 65% a combination of sample and MQ water (sample volume depending on concentration). Samples were heated at 100 °C for 3 min before being loaded and run on a gel at 200 V for 35 min. Gels were then transferred onto PVDF membranes using the iBlot transfer system for 7 min. Transferred blots were blocked in 5% wt/v powdered skim milk dissolved in

TBST for 1 h at room temperature. Primary incubation took place overnight at 4 °C with primary antibody concentration ranging from 1:250 – 1:1000 dilution depending on recommended values. The loading control primary antibody beta-actin was used on the samples separately in addition to the antibody of interest. Membranes were then washed with TBST 3x for 8 min each before incubation at room temperature for 1 h with secondary antibody (either rabbit or mouse respectively) at a concentration ranging from 1:5000 – 1:10000 dilution. After incubation with secondary antibody, the membranes were washed 3x with TBST for 8 min each. After washing, the membranes were imaged on the BioRad imager by treating the membrane with ECL reagent just prior to imaging. Exposure times were set manually in the chemiluminescence setting and the images with the most ideal exposure times for both β-actin control and protein of interest were chosen. Bands were quantified with ImageJ.

10.6. LC-MS Analysis Methodology

LC-MS analysis of protein samples was carried out using a Waters SQD2 mass spectrometer using inlet method B for small molecules, in combination with an Acquity UPLC BEH C18 column (130 Å1.7 µm, 2.1 × 50 mm). The SQD2 mass spectrometer mobile phase consisted of solvent A (0.1% formic acid in Milli-Q water), solvent B (0.1% formic acid in ACN). Gradient methods were as follows: Inlet method B: 5% B for 0.5 mins, followed by a gradient from 5% to 90% B over 5.5 mins, then 90% B for 2.5 min, followed by a gradient from 90% to 5% B over 0.25 min and finally, 5% B for 3.25 min. The capillary voltage of the electrospray source for the Waters SQD2 mass spectrometer was 3.0 kV with a cone voltage of 30 V and the desolvation gas used was nitrogen, with a flow rate of 800 L h−1. The ion series was obtained through integration of the major peaks of the chromatogram. Following this, the total mass spectra were reconstructed using the MaxEnt1 algorithm on the MassLynx software (v. 4.1), according to manufacturer's guidelines.

Section 11. Synthetic Procedures

11.1. General Synthetic Information

All reagents and solvents, unless otherwise stated, were purchased from commercial suppliers or obtained from departmental anhydrous stills and used as received. Water used experimentally was deionized and purified using a MQ system on site. Merck silica gel 60 was used for flash column chromatography as indicated, and thin-layer chromatography (TLC) analysis was performed using silica gel 60 F254 plates. Compounds were visualized using UV at 254 nm (short wave) or 365 nm (long wave) or by staining with known indicator solutions. NMR spectra were recorded on a 400 MHz Avance III HD (400 MHz for ^{1}H, 100 MHz for ^{13}C), a 400 MHz Neo Prodigy (400 MHz for ^{1}H, 100 MHz for ^{13}C), a 500 MHz DCH Cryoprobe (500 MHz for ^{1}H, 126 MHz for ^{13}C), a 700 MHz TXO Cryoprobe (700 MHz for ^{1}H, 176 MHz for ^{13}C), or a 600 MHz Avance 600 BBI (600 MHz for ^{1}H, 151 MHz for ^{13}C) spectrometer in the solvents indicated. Chemical shifts are given in ppm (δ-scale). Spectra are calibrated to the residual solvent peak. Multiplicities are described as s (singlet), d (doublet), t (triplet), q (quartet), m (multiplet), dd (double doublet) etc. Coupling constants (*J*) are reported in hertz (Hz) using MestReNova software version 14.1.2 for signal processing. Structural assignments were made with the aid of literature, or COSY, HSQC and HMBC experiments. Assignments can be seen in the experimental writeups. High-resolution mass spectra were measured on a Waters LCT Premier spectrometer, an Agilent 6230 LC TOF, or a Waters Vion IMS Qtof using the electrospray ionisation (ESI) method.

11.2. General Synthetic Methods

Glycosylation

Acetobromo-α-D-glucuronic acid methyl ester (**S2**) (1 eq) and aldehyde/ketone starting material (**11**) (1.5 eq) were combined in anhydrous acetonitrile (15 mL) under N_2 in the dark and stirred for 30 min. Ag_2O (4 eq) and activated molecular sieves were added and the reaction was stirred at room temperature overnight in the dark. Thin-layer chromatography was used to monitor reaction progress. The reaction was filtered through celite, the celite washed, and the solvent removed *in vacuo*. The residue was dissolved in ethyl acetate, washed with sat. $NaHCO_3$ (3 × 10 mL), water (10 mL), and brine (10 mL). It was then dried (sodium sulfate), and the solvent was removed *in vacuo*. The product was purified by flash column chromatography on silica gel 60 to give the glycosylated linker **12**.

Reduction

12 (1 eq) was dissolved in chloroform (5 mL) and isopropanol (1 mL) with silica gel (equal mass to **12**) and stirred at 0°C under N_2 for 15 min. $NaBH_4$ (2.0 eq) was added and the reaction was stirred for 1 h. The reaction was monitored by thin-layer chromatography (1:1 ethyl acetate: Pet ether). Upon completion, the reaction was quenched with acetone (10 mL) and allowed to stir for 30 min. The reaction was diluted with dichloromethane (10 mL), filtered over

celite, and washed with dichloromethane (10 mL). The filtrate was washed with brine (10 mL), dried (magnesium sulfate), and the solvent removed *in vacuo* to give the reduced product **13**.

Chlorination

Compound **13** (1 eq) was dissolved in anhydrous dichloromethane (20 mL) at 0°C under N_2. $SOCl_2$ (6 eq) was added and the reaction was stirred under N_2 overnight. Thin-layer chromatography was used to monitor the reaction (1:1 ethyl acetate: Pet ether) to completion. The reaction was quenched with cold sat. $NaHCO_3$ (10 mL), washed with brine (10 mL), dried (magnesium sulfate), and the solvent removed *in vacuo* to give the chlorinated product **14**.

Bromination

Compound **13** (1 eq) was dissolved in anhydrous dichloromethane or ether (30 mL) at 0°C under N_2. Neat PBr_3 (2 eq) was added and the reaction was stirred under N_2 for 1-5 h. Thin-layer chromatography then showed reaction completion. The reaction was quenched with cold sat. $NaHCO_3$ (10 mL), extracted into DCM (3 × 50 mL) washed with brine (10 mL), dried (sodium sulfate), and the solvent removed *in vacuo* to give **14**. Due to the instability of the compound, it was used immediately in the next reaction without further purification or characterization.

Indium Barbier Reaction

14 (1.55 eq), NaI (4 eq), β-lapachone (**1**) (1 eq) and indium(0) powder (1.1 eq) were added to anhydrous dimethylformamide (3 mL). The solution was heated to 40°C and sonicated overnight, while monitoring by thin-layer chromatography and LC-MS. Water (10 mL) was added to quench the reaction, and it was extracted with ethyl acetate (3 × 150 mL). The organic layers were combined and washed with brine (10 mL), dried (magnesium sulfate), and the solvent removed *in vacuo*. The product was purified by flash column chromatography on silica gel 60 to give the C-alkylated construct **15.**

Sugar Deprotection

The acetyl-protected glucuronide β-lapachone prodrug **15** (1 eq) was dissolved in tetrahydrofuran (1 mL) and methanol (1 mL) and stirred at 0°C. A separate solution of LiOH monohydrate (6 eq) in water (1 mL) was prepared and added to the solution of **15** dropwise. The reaction was stirred for 1.5 h and product formation was monitored by LC-MS. After complete conversion, glacial acetic acid (6 eq) was added and the solvent removed *in vacuo*. Crude product **16** was purified by semi-preparative HPLC.

11.3. Synthetic Procedures for 1 and 27

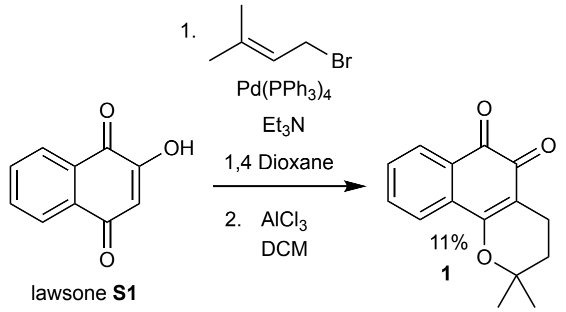

Scheme S1. Synthesis of 1 from lawsone in two steps.

β-lapachone (**1**)[21]

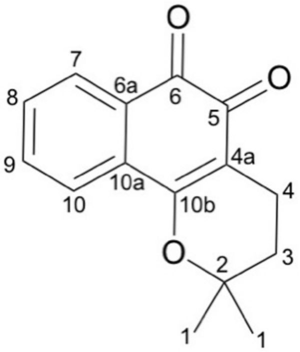

Lawsone (**S1**) (2.006 g, 11.5 mmol, 1 eq) and tetrakis(triphenylphosphine)palladium (32.5 mg, 0.028 mmol, cat.) were combined in 1,4-dioxane (50 mL) under N_2. To the resulting solution, triethylamine (1920 µL, 13.7 mmol, 1.2 eq) was added. 1-bromo-3-methyl-2-butene (1600 µL, 13.8 mmol, 1.2 eq) was then added and the reaction was stirred vigorously at room temperature for 4 h. The reaction was monitored by thin-layer chromatography (2:1 Pet ether: ethyl acetate). The reaction was stopped prior to full conversion as previous experiments suggested the product degrades if allowed to react for longer. Water (50 mL) quenched the reaction and it was extracted into dichloromethane (3 × 250 mL), dried (magnesium sulfate), filtered, and the solvent was removed *in vacuo*. The crude red solid (5.585 g) was immediately taken forward to the next step without further characterization. R_f: 0.65 (2:1 Pet ether: ethyl acetate).

The residue (5.585 g) was dissolved in anhydrous dichloromethane (60 mL) and $AlCl_3$ (4.656 g, 34.9 mmol, 3 eq) was added. The solution was stirred at room temperature for 1.5 h. Thin-layer chromatography (2:1 Pet ether: ethyl acetate) was used to monitor the reaction progress. After 1.5 h, the reaction was quenched with cold water (60 mL), extracted into dichloromethane (3 × 150 mL), dried (magnesium sulfate), and the solvent removed *in vacuo*. Purification by flash chromatography on silica gel 60 (20% ethyl acetate /Pet ether) yielded β-lapachone (**1**) as red solid (0.314 g, 1.3 mmol, 11.2%). Recrystallization from absolute ethanol was also effective for product purification.[22]

^{1}H NMR (600 MHz, $CDCl_3$) δ 8.04 (dd, *J* = 7.6, 1.4 Hz, 1H, H7), 7.80 (dd, *J* = 7.9, 1.2 Hz, 1H, H10), 7.63 (td, *J* = 7.6, 1.4 Hz, 1H, H9), 7.49 (td, *J* = 7.6, 1.2 Hz, 1H, H8), 2.56 (t, *J* = 6.7 Hz, 2H, H4), 1.84 (t, *J* = 6.7 Hz, 2H, H3), 1.46 (s, 6H, H1).

^{13}C NMR (151 MHz, $CDCl_3$) δ 179.9 (C6), 178.6 (C5), 162.1 (C10b), 134.8 (C6a), 132.7 (C8), 130.7 (C10a), 130.2 (C9), 128.6 (C7), 124.1 (C10), 112.8 (C4a), 79.3 (C2), 31.7 (C3), 26.8 (C1), 16.2 (C4).

HRMS (ESI$^+$): *m/z* calcd. for $[C_{15}H_{14}O_3+H]^+$ calcd. 243.1021 found 243.1015.

NMR spectra were in alignment with the literature procedure.[21] Numbering is based off of Di Chenna *et al.*[23]

Benzyl β-lapa-ketol[19]

6-benzyl-6-hydroxy-2,2-dimethyl-2,3,4,6-tetrahydro-5H-benzo[h]chromen-5-one (**27**)

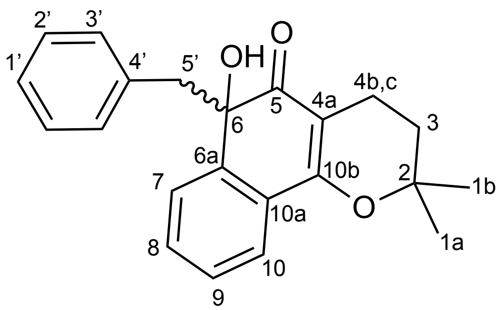

NaI (0.2936 g, 1.9 mmol, 4.5 eq), β-lapachone (**1**) (0.1012 g, 0.4 mmol, 1 eq) and indium(0) powder (0.0732 g, 0.6 mmol, 1.5 eq) were added to anhydrous dimethylformamide (3 mL). Then benzyl bromide (0.1 mL, 0.84 mmol, 2 eq) was added. The solution was sonicated while monitoring by thin-layer chromatography (25% ethyl acetate/Pet ether) and LC-MS. After 1 h, the reaction was complete. 1M HCl (~0.5 mL) was added to quench the reaction and it was extracted with ethyl acetate (3 × 150 mL). The organic layers were combined and washed with brine (10 mL), dried (sodium sulfate), and the solvent removed *in vacuo*. A portion of the product was purified by flash column chromatography on silica gel 60 (10-40% ethyl acetate/Pet ether) and then further purified by semi-preparative HPLC for kinetics studies to give 6-benzyl-6-hydroxy-2,2-dimethyl-2,3,4,6-tetrahydro-5*H*-benzo[*h*]chromen-5-one (**3**) as an off-white solid after lyophilization (0.042 g, 0.126 mmol, 30%). HPLC Purification Method: Column = Agilent

InfinityLab ZORBAX 5 Eclipse Plus C18 21.2 × 250 mm; mobile phases: A = H_2O, B = ACN; gradient: t = 0-5 min 5% B, t = 5-35 min 5-95% B, t = 35-40 min 95% B. Retention time of **27** = 31.3 min.

^{1}H NMR (400 MHz, CD_3CN) δ 7.62 (ddd, *J* = 7.1, 5.3, 1.4 Hz, 2H, H7, H10), 7.48 (td, *J* = 7.5, 1.4 Hz, 1H, H8), 7.37 (td, *J* = 7.6, 1.3 Hz, 1H, H9), 7.14 – 7.00 (m, 3H, H1', H2'), 6.58 (d, *J* = 6.6 Hz, 2H, H3'), 4.14 (s, 1H, O<u>H</u>), 3.10 – 2.98 (m, 2H, H5'), 2.43 (dt, *J* = 17.3, 5.8 Hz, 1H, H4b,c), 2.11 (ddd, *J* = 17.3, 8.0, 6.8 Hz, 1H, H4b,c), 1.70 – 1.60 (m, 2H, H3), 1.33 (s, 3H, H1a/H1b), 1.07 (s, 3H, H1a/H1b).

^{13}C NMR (101 MHz, CD_3CN) δ 200.9 (C5), 162.2 (C10b), 142.8 (C6a), 136.2 (C4'), 130.7 (C8), 130.5 (C3'), 128.7 (C1'), 128.4 (C2', C10a), 127.5 (C9), 126.9 (C7), 123.4 (C10), 108.3 (C4a), 78.9 (C6), 78.8 (C2), 54.6 (C5'), 32.0 (C3), 27.5 (C1a/C1b), 25.9 (C1a/C1b), 16.4 (C4b,c).

HRMS (ESI$^+$): *m/z* calcd. for $[C_{22}H_{22}O_3+H]^+$ calcd. 335.1642 found 335.1639.

11.4. Synthetic Procedures for 16a

Aceto-β-glucuronic acid methyl ester *para*-hydroxybenzaldehyde[24]

(2S,3S,4R,5R,6S)-6-(4-formylphenoxy)-5-hydroxy-2-(methoxycarbonyl)tetrahydro-2H-pyran-3,4-diyl diacetate (**12a**)

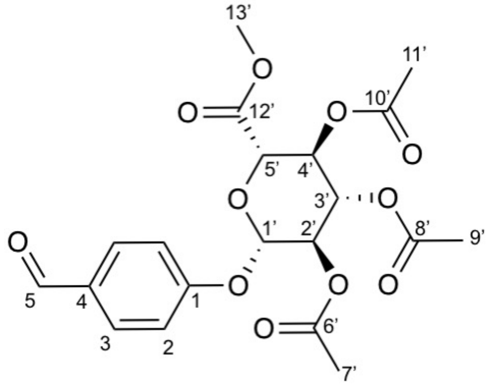

Acetobromo-α-D-glucuronic acid methyl ester (**S2**) (0.504 g, 1.27 mmol, 1 eq) and 4-hydroxybenzaldehyde (**11a**) (0.261 g, 2.1 mmol, 1.67 eq) were combined in anhydrous acetonitrile (15 mL) under N_2 in the dark and stirred for 30 min. Ag_2O (1.294 g, 5.59 mmol, 4.43 eq) and activated molecular sieves (1.27 g) were added and the reaction was stirred at room temperature overnight in the dark. Thin-layer chromatography monitored reaction progress (25% ethyl acetate/Pet ether). The reaction was filtered through celite and the solvent removed *in vacuo*. The residue was dissolved in ethyl acetate, washed with sat. $NaHCO_3$ (3 × 10 mL), water

(10 mL), and brine (10 mL). It was then dried (magnesium sulfate), and the solvent was removed *in vacuo*. The product was purified by flash column chromatography on silica gel 60 (25-40% ethyl acetate/Pet ether) to give (2*S*,3*S*,4*R*,5*R*,6*S*)-6-(4-formylphenoxy)-5-hydroxy-2-(methoxycarbonyl)tetrahydro-2H-pyran-3,4-diyl diacetate (**12a**) as white circular crystals (0.272 g, 0.62 mmol, 49%).

^{1}H NMR (600 MHz, $CDCl_3$) δ 9.17 (s, 1H, H5), 7.10 (d, *J* = 8.7 Hz, 2H, H3), 6.37 (d, *J* = 8.7 Hz, 2H, H2), 4.66 – 4.53 (m, 4H, H1', H2', H3', H4'), 3.58 – 3.51 (m, 1H, H5'), 2.96 (s, 3H, H13'), 1.33 – 1.28 (m, 9H, H11', H9', H7').

^{13}C NMR (151 MHz, $CDCl_3$) δ 190.7 (C5), 170.0 (C8'), 169.3 (C10'), 169.2 (C6'), 166.7 (C12'), 162.5 (C1), 161.0 (C4), 131.9 (C3), 131.8 (C3), 116.8 (C2), 98.0 (C1'), 72.7 (C5'), 71.6 (C3'), 70.9 (C2'), 68.9 (C4'), 53.0 (C13'), 20.6 (C7'), 20.6 (C9'), 20.5 (C11').

HRMS (ESI$^+$): *m/z* calcd. for $[C_{20}H_{22}O_{11}+Na]^+$ calcd. 461.1060 found 461.1053.

NMR spectra were in alignment with the literature procedure.[24]

Aceto-β-glucuronic acid methyl ester *para*-hydroxybenzyl alcohol[24]

(2S,3R,4S,5S,6S)-2-(4-(hydroxymethyl)phenoxy)-6-(methoxycarbonyl)tetrahydro-2H-pyran-3,4,5-triyl triacetate (**13a**)

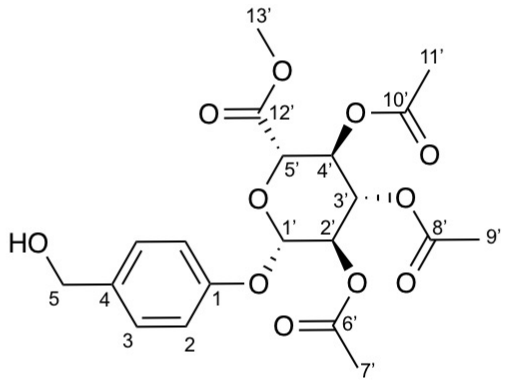

(2*S*,3*S*,4*R*,5*R*,6*S*)-6-(4-formylphenoxy)-5-hydroxy-2-(methoxycarbonyl) tetrahydro-2H-pyran-3,4-diyl diacetate (**12a**) (0.272 g, 0.62 mmol, 1 eq) was dissolved in chloroform (5 mL) and isopropanol (1 mL) with silica gel (0.272 g) and stirred at 0°C under N_2 for 15 min. $NaBH_4$ (0.048 g, 1.27 mmol, 2.0 eq) was added and the reaction was stirred for 45 min. It was monitored by thin-layer chromatography (1:1 ethyl acetate: Pet ether). The reaction was diluted with

dichloromethane (10 mL), filtered over celite, and washed with dichloromethane (10 mL). The filtrate was washed with brine (10 mL), dried (magnesium sulfate), and the solvent removed *in vacuo* to give (2*S*,3*R*,4*S*,5*S*,6*S*)-2-(4-(hydroxymethyl)phenoxy)-6-(methoxycarbonyl)tetrahydro-2*H*-pyran-3,4,5-triyl triacetate (**13a**) as an off-white solid (0.252 g, 0.57 mmol, 92%).

^{1}H NMR (600 MHz, $CDCl_3$) δ 7.23 (d, *J* = 8.6 Hz, 2H, H3), 6.92 (d, *J* = 8.6 Hz, 2H, H2), 5.31 (t, *J* = 9.3 Hz, 1H, H2'), 5.27 – 5.20 (m, 2H, H3', H4'), 5.10 (d, *J* = 7.6 Hz, 1H, H1'), 4.54 (s, 2H, H5), 4.18 (d, *J* = 9.6 Hz, 1H, H5'), 3.67 (s, 3H, H13'), 2.89 (s, 1H, OH), 2.05 – 1.95 (m, 9H, H11', H9', H7').

^{13}C NMR (151 MHz, $CDCl_3$) δ 170.1 (C6'), 169.5 (C8'), 169.3 (C10'), 166.9 (C12'), 155.9 (C1), 136.2 (C4), 128.3 (C3), 116.8 (C2), 98.9 (C1'), 72.2 (C5'), 71.7 (C3'), 70.8 (C2'), 69.0 (C4'), 64.2 (C5), 52.9 (C13'), 20.5 (C11'), 20.5 (C9'), 20.4 (C7').

R_f: 0.15 (1:1 ethyl acetate: Pet ether).

HRMS (ESI$^+$): *m/z* calcd. for $[C_{20}H_{24}O_{11}+Na]^+$ calcd. 463.1216 found 463.1213.

NMR spectra were in alignment with the literature procedure.[24]

Aceto-β-glucuronic acid methyl ester *para*-hydroxybenzyl chloride[25]

(2S,3R,4S,5S,6S)-2-(4-(chloromethyl)phenoxy)-6-(methoxycarbonyl)tetrahydro-2H-pyran-3,4,5-triyl triacetate (**14a**)

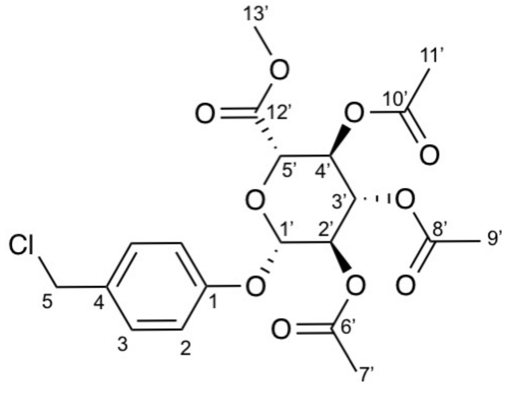

(2*S*,3*R*,4*S*,5*S*,6*S*)-2-(4-(hydroxymethyl)phenoxy)-6-(methoxycarbonyl)tetrahydro-2H-pyran-3,4,5-triyl triacetate (**13a**) (0.179 g, 0.41 mmol, 1 eq) was dissolved in anhydrous dichloromethane (20 mL) at 0°C under N_2. $SOCl_2$ (0.2 mL, 2.74 mmol, 6.7 eq) was added and the reaction was stirred under N_2 overnight. Thin-layer chromatography monitored the reaction

(1:1 ethyl acetate: Pet ether) to completion. The reaction was quenched with sat. $NaHCO_3$ (10 mL), washed with brine (10 mL), dried (magnesium sulfate), and the solvent removed *in vacuo* to give (2*S*,3*R*,4*S*,5*S*,6*S*)-2-(4-(chloromethyl)phenoxy)-6-(methoxy carbonyl)tetrahydro-2*H*-pyran-3,4,5-triyl triacetate (**14a**) as an off-white solid (0.165 g, 0.36 mmol, 90%).

^{1}H NMR (400 MHz, $CDCl_3$) δ 7.29 (d, *J* = 8.7 Hz, 2H, H3), 6.95 (d, *J* = 8.6 Hz, 2H, H2), 5.34 (q, *J* = 9.3 Hz, 2H, H3', H4'), 5.30 – 5.21 (m, 1H, H2'), 5.16 (d, *J* = 7.4 Hz, 1H, H1'), 4.52 (s, 2H, H5), 4.23 (d, *J* = 9.0 Hz, 1H, H5'), 3.69 (s, 3H, H13'), 2.02 (s, 9H, H11', H9', H7').

^{13}C NMR (100 MHz, $CDCl_3$) δ 169.9 (C6'), 169.3 (C8'), 169.1 (C10'), 166.8 (C12'), 156.5 (C1), 132.5 (C4), 130.0 (C3), 117.0 (C2), 98.7 (C1'), 72.4 (C5'), 71.7 (C3'), 70.9 (C2'), 69.0 (C4'), 52.9 (C13'), 45.7 (C5), 20.5 (C11'), 20.5 (C9'), 20.4 (C7').

HRMS (ESI$^+$): *m/z* calcd. for $[C_{20}H_{23}O_{10}Cl+Na]^+$ calcd. 481.0877 found 481.0876.

Aceto-β-glucuronic acid methyl ester *para*-hydroxybenzyl β-lapa-ketol[19]

(2S,3R,4S,5S,6S)-2-(4-((6-hydroxy-2,2-dimethyl-5-oxo-3,4,5,6-tetrahydro-2H-benzo[h]chromen-6-yl)methyl)phenoxy)-6-(methoxycarbonyl)tetrahydro-2H-pyran-3,4,5-triyl triacetate (**15a**)

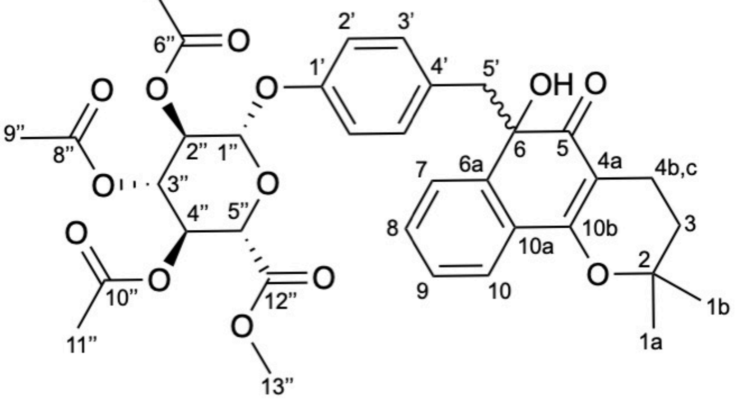

(2*S*,3*R*,4*S*,5*S*,6*S*)-2-(4-(chloromethyl)phenoxy)-6-(methoxycarbonyl)tetrahydro-2H-pyran-3,4,5-triyl triacetate (**14a**) (0.231 g, 0.50 mmol, 1.2 eq), NaI (0.255 g, 1.7 mmol, 4.2 eq), β-lapachone (**1**) (0.098 g , 0.40 mmol, 1 eq) and indium(0) powder (0.058 g, 0.51 mmol, 1.2 eq) were added to anhydrous dimethylformamide (6 mL). The solution was heated to 40°C and sonicated for 45 min, while monitoring by thin-layer chromatography (2:3 Pet ether: ethyl acetate). Excess In(0) powder was added and the reaction was left stirring at room temperature overnight. The next day, it was again sonicated at 40°C for 1.5 h. Water (10 mL) and 7 drops 1M

HCl were added to quench the reaction and it was extracted with ethyl acetate (3 × 150 mL). The organic layers were combined and washed with brine (10 mL), dried (magnesium sulfate), and the solvent removed *in vacuo*. The product was purified by flash column chromatography on silica gel 60 (2% methanol:dichloromethane) to give (2*S*,3*R*,4*S*,5*S*,6*S*)-2-(4-((6-hydroxy-2,2-dimethyl-5-oxo-3,4,5,6-tetrahydro-2*H*-benzo[*h*]chromen-6-yl)methyl)phenoxy)-6-(methoxy carbonyl)tetrahydro-2*H*-pyran-3,4,5-triyl triacetate (**15a**) as an off-white solid (0.099 g, 0.15 mmol, 37%).

^{1}H NMR (400 MHz, $CDCl_3$) δ 7.69 – 7.63 (m, 1H, H10), 7.57 (ddd, *J* = 15.7, 7.7, 1.3 Hz, 1H, H7), 7.43 (tdd, *J* = 7.6, 4.7, 1.4 Hz, 1H, H8), 7.33 (td, *J* = 7.6, 1.3 Hz, 1H, H9), 6.70 (dd, *J* = 8.5, 6.1 Hz, 2H, H2'), 6.54 (t, *J* = 8.6 Hz, 2H, H3'), 5.32 – 5.26 (m, 2H, H3'', H4''), 5.25 – 5.19 (m, 1H, H2''), 5.02 (t, *J* = 7.0 Hz, 1H, H1''), 4.15 – 4.10 (m, 1H, H5''), 3.93 (d, *J* = 2.1 Hz, 1H, OH), 3.72 (d, *J* = 4.6 Hz, 3H, H13''), 3.00 (d, *J* = 2.3 Hz, 2H, H5'), 2.54 (dtd, *J* = 17.3, 5.7, 2.5 Hz, 1H, H4b,c), 2.13 – 2.08 (m, 1H, H4b,c), 2.05 – 1.96 (m, 9H, H7", H9", H11"), 1.65 (qd, *J* = 8.3, 7.6, 3.8 Hz, 2H, H3), 1.36 (d, *J* = 1.8 Hz, 3H, H1a/H1b), 1.11 (d, *J* = 8.7 Hz, 3H, H1a/H1b).

^{13}C NMR (100 MHz, $CDCl_3$) δ 200.5 (C5), 200.5 (C5), 170.2 (C8''), 169.4 (C10''), 169.2 (C6''), 169.2 (C6''), 166.9 (C12''), 166.9 (C12''), 162.2 (C10b), 162.2 (C10b), 155.8 (C1'), 155.7 (C1'), 141.5 (C6a), 130.9 (C3'), 130.9 (C3'), 130.3 (C4'), 130.3 (C4'), 130.2 (C8), 130.1 (C8), 127.6 (C9), 127.5 (C10a), 127.4 (C10a), 125.7 (C7), 125.7 (C7), 123.1 (C10), 123.1 (C10), 116.2 (C2'), 116.1 (C2'), 106.7 (C4a), 106.7 (C4a), 99.2 (C1''), 99.0 (C1''), 78.1 (C6), 78.1 (C6), 78.0 (C2), 72.7 (C5''), 72.7 (C5''), 72.0 (C3''), 71.1 (C2''), 71.0 (C2''), 69.2 (C4''), 69.2 (C4''), 53.5 (C5'), 53.4 (C5'), 53.0 (C13''), 53.0 (C13''), 31.7 (C3), 31.7 (C3), 27.6 (C1a, C1b), 27.6 (C1a, C1b), 25.7 (C1a, C1b), 20.7 (C11'', C9'', C7''), 20.6 (C11'', C9'', C7''), 15.7 (C4b,c), 15.7 (C4b,c).

HRMS (ESI^{+}): *m/z* calcd. for $[C_{35}H_{38}O_{13}+H]^{+}$ calcd. 667.2391 found 667.2403.

β-glucuronide-*para*-hydroxybenzyl β-lapa-ketol[26]

(2S,3S,4S,5R,6S)-3,4,5-trihydroxy-6-(4-((6-hydroxy-2,2-dimethyl-5-oxo-3,4,5,6-tetrahydro-2H-benzo[h]chromen-6-yl)methyl)phenoxy)tetrahydro-2H-pyran-2-carboxylic acid (**16a**)

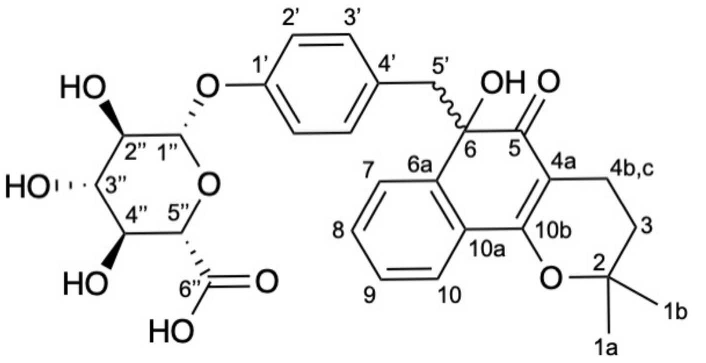

The acetyl-protected glucuronide β-lapachone prodrug (**15a**) (0.066 g, 0.1 mmol, 1 eq) was dissolved in tetrahydrofuran (1 mL) and methanol (1 mL) and stirred at 0°C. A separate solution of LiOH monohydrate (0.025 g, 0.6 mmol, 6 eq) in water (1 mL) was prepared and added to the solution of **15a** dropwise. The reaction was stirred for 1.5 h and product formation was monitored by LC-MS. After complete conversion, glacial acetic acid (33.9 µL, 0.6 mmol, 6 eq) was added and the solvent removed *in vacuo*. Crude product **16a** was purified by semi-preparative HPLC to give (2*S*,3*S*,4*S*,5*R*,6*S*)-3,4,5-trihydroxy-6-(4-((6-hydroxy-2,2-dimethyl-5-oxo-3,4,5,6-tetrahydro-2*H*-benzo[*h*]chromen-6-yl)methyl)phenoxy)tetrahydro-2*H*-pyran-2-carboxylic acid (**16a**) as a white solid (0.054 g, 0.10 mmol, 82%). HPLC Purification Method: Column = YMC Pack Pro C18 5 µm 250×10 mm 120Å; mobile phases: A = H_2O + 0.1 % formic acid, B = ACN + 0.1 % formic acid; gradient: t = 0-1 min 0% B, t = 1-10 min 0-100% B, t = 10-12 min 100% B. Retention time of **16a** = 8.06 min.

^{1}H NMR (500 MHz, CD_3CN) δ 8.03 (s, 1H, COO<u>H</u>), 7.62 (ddt, *J* = 7.4, 2.6, 1.5 Hz, 2H, H10, H7), 7.48 (tdd, *J* = 7.6, 3.2, 1.3 Hz, 1H, H8), 7.38 (tdd, *J* = 7.6, 3.0, 1.4 Hz, 1H, H9), 6.72 (dd, *J* = 8.6, 3.6 Hz, 2H, H2'), 6.50 (dd, *J* = 8.7, 2.8 Hz, 2H, H3'), 4.86 (d, *J* = 7.4 Hz, 0.5H, H1''), 4.83 (d, *J* = 7.4 Hz, 0.5H, H1''), 4.11 (s, 1H, O<u>H</u>), 3.93 (dd, *J* = 9.8, 6.6 Hz, 1H, H3''), 3.54 (ddd, *J* = 9.8, 8.7, 1.0 Hz, 1H, H4''), 3.48 – 3.33 (m, 2H, H5'', H2''), 3.05 – 2.94 (m, 2H, H5'), 2.44 (dtd, *J* = 17.3, 5.7, 3.3 Hz, 1H, H4b,c), 2.22 (s, 2H, Gluc 2x O<u>H</u>'s), 2.11 (dddd, *J* = 17.4, 8.9, 6.5, 4.3 Hz, 2H, Gluc O<u>H</u>, H4b,c), 1.72 – 1.58 (m, 2H, H3), 1.34 (d, *J* = 4.4 Hz, 3H, H1a, H1b), 1.09 (d, *J* = 3.7 Hz, 3H, H1a, H1b).

^{1}H NMR (400 MHz, MeOD) δ 7.72 (ddd, *J* = 7.7, 6.0, 1.3 Hz, 1H, H10), 7.59 (ddd, *J* = 7.8, 5.3, 1.3 Hz, 1H, H7), 7.51 (tt, *J* = 7.6, 1.5 Hz, 1H, H8), 7.36 (td, *J* = 7.6, 1.3 Hz, 1H, H9), 6.71 (dd, *J* = 11.1, 8.6 Hz, 2H, H2'), 6.37 (dd, *J* = 8.6, 5.0 Hz, 2H, H3'), 4.82 (d, *J* = 7.3 Hz, 0.5H, H1''), 4.77 (d, *J* = 7.7 Hz, 0.5H, H1''), 3.85 (dd, *J* = 9.7, 6.6 Hz, 1H, H3''), 3.57 (td, *J* = 9.4, 3.0 Hz, 1H, H4''), 3.50 – 3.38 (m, 2H, H5'', H2''), 3.13 (dd, *J* = 12.1, 3.8 Hz, 1H, H5'), 3.00 (dd, *J* = 12.1, 4.3 Hz, 1H, H5'), 2.47 – 2.32 (m, 1H, H4b,c), 2.11 – 2.00 (m, 1H, H4b,c), 1.64 – 1.54 (m, 2H, H3), 1.37 – 1.24 (m, 3H, H1a, H1b), 1.00 (d, *J* = 2.2 Hz, 3H, H1a, H1b).

^{13}C NMR (126 MHz, CD_3CN) δ 200.9 (C5), 200.8 (C5), 170.0 (C6’’), 170.0 (C6’’), 162.2 (C10b), 162.2 (C10b), 157.0 (C1’), 156.9 (C1’), 142.7 (C6a), 142.7 (C6a), 131.5 (C3’), 131.5 (C3’), 130.7 (C8), 130.7 (C8), 130.3 (C4’), 130.3 (C4’), 128.6 (C10a), 128.4 (C9), 128.3 (C9), 126.8 (C7), 126.7 (C7), 123.3 (C10), 123.3 (C10), 116.2 (C2’), 116.2 (C2’), 108.3 (C4a), 108.2 (C4a), 101.3 (C1’’), 101.1 (C1’’), 78.9 (C6), 78.8 (C6), 78.7 (C2), 78.7 (C2), 76.5 (C5’’), 75.2 (C3’’), 75.2 (C3’’), 73.8 (C2’’), 72.2 (C4’’), 72.2 (C4’’), 53.8 (C5’), 53.7 (C5’), 32.0 (C3), 32.0 (C3), 27.6 (C1a, C1b), 27.5 (C1a, C1b), 25.9 (C1a, C1b), 25.8 (C1a, C1b), 16.3 (C4b,c), 16.3 (C4b,c).

HRMS (ESI^+): *m/z* calcd. for $[C_{28}H_{30}O_{10}+H]^+$ calcd. 527.1917 found 527.1914.

11.5. Synthetic Procedures for 16b

Aceto-β-glucuronic acid methyl ester 3,5-difluoro-*para*-hydroxybenzaldehyde[24]

(2S,3R,4S,5S,6S)-2-(2,6-difluoro-4-formylphenoxy)-6-(methoxycarbonyl)tetrahydro-2H-pyran-3,4,5-triyl triacetate (**12b**)[24]

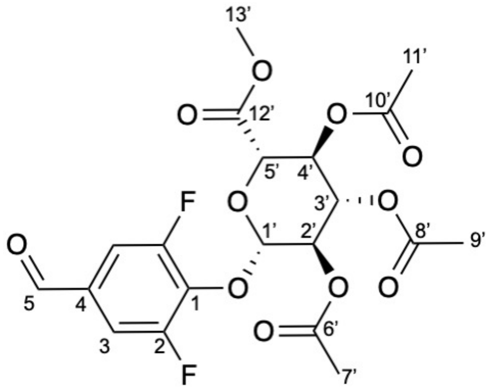

Acetobromo-α-D-glucuronic acid methyl ester (**S2**) (1.009 g, 2.5 mmol, 1 eq) and 3,5-difluoro-4-hydroxybenzaldehyde (**11b**) (0.669 g, 4.2 mmol, 1.67 eq) were combined in anhydrous acetonitrile (15 mL) under N_2 in the dark and stirred for 30 min. Ag_2O (2.07 g, 8.9 mmol, 3.6 eq) and activated molecular sieves (2 g) were added and the reaction was stirred at room temperature overnight in the dark. Thin-layer chromatography monitored reaction progress (1:2 ethyl acetate/Pet ether). The reaction was filtered through celite and the solvent removed *in vacuo*. The residue was dissolved in ethyl acetate, washed with sat. Na_2CO_3 (10 mL), water (10 mL), and brine (10 mL). It was then dried (magnesium sulfate), and the solvent was removed *in vacuo*. The product was purified by flash column chromatography on silica gel 60 (20-50% ethyl acetate/Pet ether) to give (2*S*,3*R*,4S,5*S*,6*S*)-2-(2,6-difluoro-4-formylphenoxy)-6-(methoxycarbonyl)tetrahydro-2*H*-pyran-3,4,5-triyl triacetate (**12b**) as an off white solid (0.756 g, 1.6 mmol, 63%).

^{1}H NMR (400 MHz, $CDCl_3$) δ 9.81 (s, 1H, H5), 7.43 (d, *J* = 7.3 Hz, 2H, H3), 5.75 – 4.65 (m, 4H, H1', H2', H3', H4'), 4.07 (dd, *J* = 10.8, 6.6 Hz, 1H, H5'), 3.66 (s, 3H, H13'), 2.04 (s, 3H, H11'/H9'/H7'), 2.00 (s, 3H, H11'/H9'/H7'), 1.97 (s, 3H, H11'/H9'/H7').

^{13}C NMR (101 MHz, $CDCl_3$) δ 188.7 (t, *J* = 2.2 Hz) (C5), 169.9 (C8'), 169.3 (C10'), 169.1 (C6'), 166.5 (C12'), 155.7 (dd, *J* = 254.1, 4.2 Hz) (C2), 137.4 (t, *J* = 14.3 Hz) (C1), 132.7 (t, *J* = 6.6 Hz) (C4), 113.3 (dd, *J* = 17.5, 6.0 Hz) (C3), 101.1 (t, *J* = 2.9 Hz) (C1'), 72.7 (C5'), 71.5 (C3'), 71.1 (C2'), 68.9 (C4'), 52.9 (C13'), 20.5 (C7'), 20.5 (C9'), 20.4 (C11').

HRMS (ESI^+): *m/z* calcd. for $[C_{20}H_{20}O_{11}F_2+Na]^+$ calcd. 497.0866 found 497.0855.

Aceto-β-glucuronic acid methyl ester 3,5-difluoro-*para*-hydroxybenzyl alcohol[24]

(2S,3R,4S,5S,6S)-2-(2,6-difluoro-4-(hydroxymethyl)phenoxy)-6-(methoxycarbonyl)tetrahydro-2H-pyran-3,4,5-triyl triacetate (**13b**)[24]

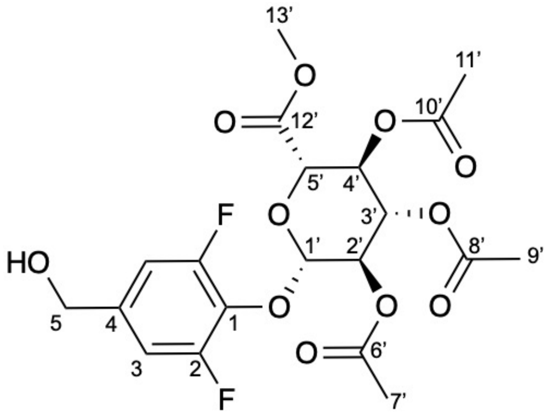

(2*S*,3*R*,4S,5*S*,6*S*)-2-(2,6-difluoro-4-formylphenoxy)-6-(methoxycarbonyl) tetrahydro-2*H*-pyran-3,4,5-triyl triacetate (**12b**) (0.756 g, 1.6 mmol, 1 eq) was dissolved in chloroform (8 mL) and isopropanol (2 mL) with silica gel (0.750 g) and stirred at 0°C under N_2 for 15 min. $NaBH_4$ (0.120 g, 3.2 mmol, 2 eq) was added and the reaction was stirred for 45 min. It was monitored by thin-layer chromatography (1:2 ethyl acetate: Pet ether). The reaction was diluted with dichloromethane (10 mL), filtered over celite, and washed with dichloromethane (10 mL). The filtrate was washed with brine (10 mL), dried (magnesium sulfate), and the solvent removed *in vacuo* to give (2*S*,3*R*,4*S*,5*S*,6*S*)-2-(2,6-difluoro-4-(hydroxymethyl)phenoxy)-6-(methoxy carbonyl) tetrahydro-2*H*-pyran-3,4,5-triyl triacetate (**13b**) as an off-white solid (0.604 g, 1.26 mmol, 80%).

^{1}H NMR (400 MHz, $CDCl_3$) δ 6.86 (d, *J* = 8.5 Hz, 2H, H3), 5.32 – 5.18 (m, 3H, H2', H3', H4'), 4.98 (d, *J* = 6.8 Hz, 1H, H1'), 4.54 (s, 2H, H5), 4.00 (d, *J* = 9.4 Hz, 1H, H5'), 3.66 (s, 3H, H13'), 3.23 (brs, 1H, O<u>H</u>), 2.02 (s, 3H, H11'/H9'/H7'), 1.97 (s, 3H, H11'/H9'/H7'), 1.95 (s, 3H, H11'/H9'/H7').

^{13}C NMR (101 MHz, $CDCl_3$) δ 170.1 (C6'), 169.5 (C8'), 169.4 (C10'), 166.8 (C12'), 155.5 (dd, *J* = 250.4, 4.6 Hz) (C2), 139.6 (t, *J* = 7.8 Hz) (C4), 131.2 (t, *J* = 14.8 Hz) (C1), 110.0 (dd, *J* = 16.7, 5.3 Hz) (C3), 102.0 (C1'), 72.5 (C5'), 71.7 (C3'), 71.1 (C2'), 69.2 (C4'), 63.2 (t, *J* = 1.7 Hz) (C5), 52.9 (C13'), 20.5 (C11'), 20.4 (C9'), 20.4 (C7').

HRMS (ESI$^-$): neutral mass calcd. for [$C_{20}H_{22}O_{11}F_2$] calcd. 476.11302 found 476.1136.

Aceto-β-glucuronic acid methyl ester 3,5-difluoro-*para*-hydroxybenzyl chloride[25]

(2S,3R,4S,5S,6S)-2-(4-(chloromethyl)-2,6-difluorophenoxy)-6-(methoxycarbonyl)tetrahydro-2H-pyran-3,4,5-triyl triacetate (**14b**)

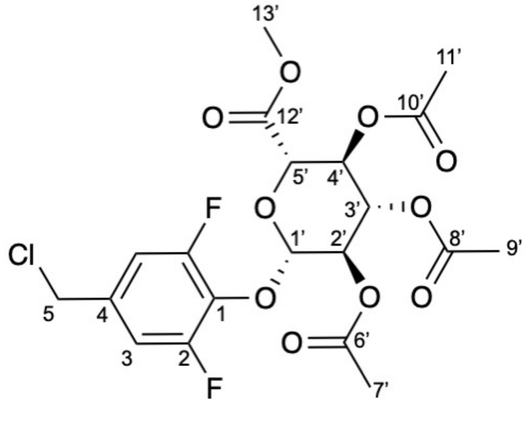

(2*S*,3*R*,4*S*,5*S*,6*S*)-2-(2,6-difluoro-4-(hydroxymethyl)phenoxy)-6-(methoxycarbonyl) tetrahydro-2*H*-pyran-3,4,5-triyl triacetate (**13b**) (0.604 g, 1.26 mmol, 1 eq) was dissolved in anhydrous dichloromethane (25 mL) at 0°C under N_2. $SOCl_2$ (0.62 mL, 8.5 mmol, 6.7 eq) was added and the reaction was stirred under N_2 overnight. Thin-layer chromatography (1:1 ethyl acetate: Pet ether) and LC-MS monitored the reaction to completion. The reaction was quenched with sat. $NaHCO_3$ (10 mL), washed with brine (10 mL), dried (magnesium sulfate), and the solvent removed *in vacuo* to give (2*S*,3*R*,4*S*,5*S*,6*S*)-2-(4-(chloromethyl)-2,6-difluorophenoxy)-6-(methoxycarbonyl) tetrahydro-2*H*-pyran-3,4,5-triyl triacetate (**14b**) as an off-white solid (0.588 g, 1.19 mmol, 94%).

^{1}H NMR (400 MHz, $CDCl_3$) δ 6.94 (d, *J* = 8.1 Hz, 2H, H3), 5.34 – 5.22 (m, 3H, H2', H3', H4'), 5.04 (d, *J* = 7.0 Hz, 1H, H1'), 4.45 (s, 2H, H5), 4.07 – 3.98 (m, 1H, H5'), 3.69 (s, 3H, H13'), 2.05 (s, 3H, H11'/H9'/H7'), 2.00 (s, 3H, H11'/H9'/H7'), 1.98 (s, 3H, H11'/H9'/H7').

^{13}C NMR (101 MHz, $CDCl_3$) δ 170.0 (C6'), 169.3 (C8'), 169.3 (C10'), 166.6 (C12'), 155.5 (dd, *J* = 251.5, 4.9 Hz) (C2), 135.1 (t, *J* = 8.6 Hz) (C4), 132.4 (t, *J* = 14.5 Hz) (C1), 112.5 (dd, *J* = 17.5, 6.5 Hz) (C3), 101.9 (t, *J* = 2.2 Hz) (C1'), 72.7 (C5'), 71.7 (C3'), 71.1 (C2'), 69.1 (C4'), 52.9 (C13'), 44.5 (t, *J* = 1.9 Hz) (C5), 20.6 (C11'), 20.5 (C9'), 20.4 (C7').

HRMS (ESI^+): *m/z* calcd. for $[C_{20}H_{21}O_{10}ClF_2+Na]^+$ calcd. 517.0684 found 517.0668.

Aceto-β-glucuronic acid methyl ester 3,5-difluoro-*para*-hydroxybenzyl β-lapa-ketol[19]

(2S,3R,4S,5S,6S)-2-(2,6-difluoro-4-((6-hydroxy-2,2-dimethyl-5-oxo-3,4,5,6-tetrahydro-2H-benzo[h]chromen-6-yl)methyl)phenoxy)-6-(methoxycarbonyl)tetrahydro-2H-pyran-3,4,5-triyl triacetate (**15b**)

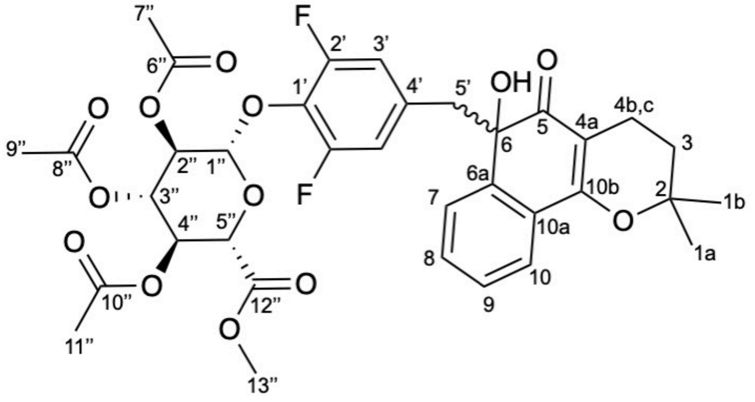

(2*S*,3*R*,4*S*,5*S*,6*S*)-2-(4-(chloromethyl)-2,6-difluorophenoxy)-6-(methoxycarbonyl) tetrahydro-2*H*-pyran-3,4,5-triyl triacetate (**14b**) (0.588 g, 1.2 mmol, 2.1 eq), NaI (0.503 g, 3.4 mmol, 6 eq), β-lapachone (**1**) (0.139 g, 0.57 mmol, 1 eq) and indium(0) powder (0.139 g, 1.2 mmol, 2.1 eq) were added to anhydrous dimethylformamide (6 mL). The solution was heated to 40°C and sonicated for 4 h, while monitoring by thin-layer chromatography (1:1 Pet ether: ethyl acetate) and LC-MS. The reaction was left stirring at room temperature overnight. Water (10 mL) was added to quench the reaction, and it was extracted with ethyl acetate (3 × 150 mL). The organic layers were combined and washed with brine (10 mL), dried (magnesium sulfate), and the solvent removed *in vacuo*. The product was purified by flash column chromatography on silica gel 60 (40-60% ethyl acetate/Pet ether) to give (2*S*,3*R*,4*S*,5*S*,6*S*)-2-(2,6-difluoro-4-((6-hydroxy-2,2-dimethyl-5-oxo-3,4,5,6-tetrahydro-2*H*-benzo[*h*] chromen-6-yl)methyl)phenoxy)-6-

(methoxycarbonyl)tetrahydro-2*H*-pyran-3,4,5-triyl triacetate (**15b**) as an off-white solid (0.529 g, 0.75 mmol, 70%).

^{1}H NMR (400 MHz, $CDCl_3$) δ 7.67 (td, *J* = 7.5, 1.4 Hz, 1H, H10), 7.47 (ddd, *J* = 12.9, 7.7, 1.4 Hz, 1H, H7), 7.39 (tdd, *J* = 7.5, 5.2, 1.4 Hz, 1H, H8), 7.31 (td, *J* = 7.6, 1.4 Hz, 1H, H9), 6.20 (t, *J* = 8.2 Hz, 2H, H3'), 5.32 – 5.19 (m, 3H, H2'', H3'', H4''), 4.93 (dd, *J* = 7.0, 3.1 Hz, 1H, H1''), 4.04 (s, 1H, O<u>H</u>), 3.99 (d, *J* = 9.2 Hz, 1H, H5''), 3.69 (d, *J* = 2.7 Hz, 3H, H13''), 2.91 (d, *J* = 6.0 Hz, 2H, H5'), 2.53 (dt, *J* = 17.4, 5.8 Hz, 1H, H4b,c), 2.26 – 2.15 (m, 1H, H4b,c), 2.08 – 1.96 (m, 9H, H7", H9", H11"), 1.77 – 1.60 (m, 2H, H3), 1.36 (s, 3H, H1a/H1b), 1.19 (d, *J* = 6.8 Hz, 3H, H1a/H1b).

^{13}C NMR (101 MHz, $CDCl_3$) δ 199.9 (d, *J* = 7.4 Hz) (C5), 170.0 (d, *J* = 1.0 Hz) (C8"), 169.3 (C10"), 169.2 (d, *J* = 4.5 Hz) (C6"), 166.6 (d, *J* = 1.9 Hz) (C12''), 162.2 (d, *J* = 2.0 Hz) (C10b), 154.2 (ddd, *J* = 250.2, 4.9, 2.6 Hz) (C2'), 140.9 (C6a), 133.2 (td, *J* = 8.0, 4.9 Hz) (C4'), 131.4 (t, *J* = 14.5 Hz) (C1'), 130.2 (d, *J* = 4.8 Hz) (C8), 127.8 (d, *J* = 1.4 Hz) (C9), 127.0 (d, *J* = 4.8 Hz) (C10a), 125.6 (d, *J* = 2.7 Hz) (C7), 123.3 (d, *J* = 2.8 Hz) (C10), 113.6 (dd, *J* = 22.3, 5.1 Hz) (C3'), 106.4 (d, *J* = 8.1 Hz) (C4a), 102.1 (C1"), 78.2 (d, *J* = 1.3 Hz) (C6), 77.5 (C2), 72.7 (d, *J* = 1.5 Hz) (C5"), 71.7 (d, *J* = 2.2 Hz) (C3"), 71.1 (d, *J* = 2.5 Hz) (C2"), 69.2 (C4"), 52.9 (C5'), 52.8 (C13"), 31.5 (C3), 27.4 (d, *J* = 2.9 Hz) (C1a/C1b), 25.5 (d, *J* = 7.1 Hz) (C1a/C1b), 20.6 (C11''/C9''/C7''), 20.5 (C11''/C9''/C7''), 20.4 (C11''/C9''/C7''), 15.7 (d, *J* = 2.4 Hz) (C4b,c).

HRMS (ESI^{+}): *m/z* calcd. for $[C_{35}H_{36}O_{13}F_2+H]^+$ calcd. 703.2197 found 703.2195.

β-glucuronide-3,5-difluoro-*para*-hydroxybenzyl β-lapa-ketol[26]

(2S,3S,4S,5R,6S)-6-(2,6-difluoro-4-((6-hydroxy-2,2-dimethyl-5-oxo-3,4,5,6-tetrahydro-2H-benzo[h]chromen-6-yl)methyl)phenoxy)-3,4,5-trihydroxytetrahydro-2H-pyran-2-carboxylic acid (**16b**)

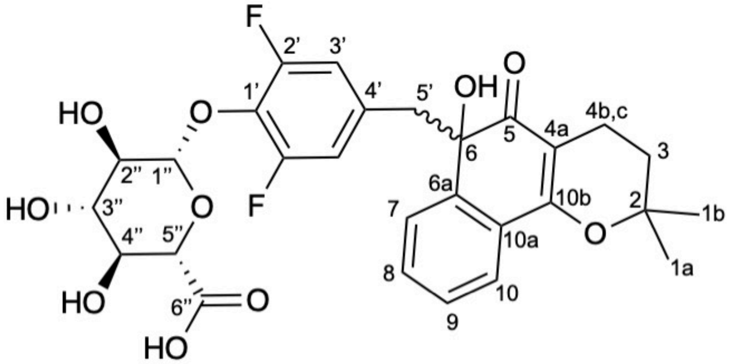

The acetyl-protected glucuronide β-lapachone prodrug (**15b**) (0.529 g, 0.75 mmol, 1 eq) was dissolved in tetrahydrofuran (1 mL) and methanol (1 mL) and stirred at 0°C. A separate solution of LiOH monohydrate (0.189 g, 4.5 mmol, 6 eq) in water (1 mL) was prepared and added to the solution of **15b** dropwise. The reaction was stirred for 1.5 h and product formation was monitored by LC-MS. After complete conversion, glacial acetic acid (258.5 µL, 4.5 mmol, 6 eq) was added and the solvent removed *in vacuo*. Crude product **16b** was purified by semi-preparative HPLC to give (2*S*,3*S*,4*S*,5*R*,6*S*)-6-(2,6-difluoro-4-((6-hydroxy-2,2-dimethyl-5-oxo-3,4,5,6-tetrahydro-2*H*-benzo[*h*]chromen-6-yl)methyl) phenoxy)-3,4,5-trihydroxy tetrahydro-2*H*-pyran-2-carboxylic acid (**16b**) as an off-white solid (0.347 g, 0.616 mmol, 98%). HPLC Purification Method: Column = YMC Pack Pro C18 5 µm 250×10 mm 120Å; mobile phases: A = H_2O + 0.1 % formic acid, B = ACN + 0.1 % formic acid; gradient: t = 0-1 min 0% B, t = 1-10 min 0-100% B, t = 10-12 min 100% B. Retention time of **16b** = 8.48 min.

^{1}H NMR (500 MHz, MeOD) δ 8.12 (s, 1H, COOH), 7.67 (tdd, *J* = 8.5, 6.8, 1.3 Hz, 2H, H10, H7), 7.52 (tt, *J* = 7.6, 1.5 Hz, 1H, H8), 7.44 – 7.37 (m, 1H, H9), 6.12 (dd, *J* = 9.2, 6.7 Hz, 2H, H3'), 4.83 (dd, *J* = 7.3, 2.2 Hz, 1H, H1''), 3.70 (dd, *J* = 9.8, 8.1 Hz, 1H, H3"), 3.58 (td, *J* = 9.3, 2.0 Hz, 1H, H4"), 3.50 – 3.38 (m, 2H, H5'', H2''), 3.13 – 2.96 (m, 2H, H5'), 2.49 (dq, *J* = 17.0, 5.6 Hz, 1H, H4b,c), 2.23 – 2.11 (m, 1H, H4b,c), 1.71 (tp, *J* = 7.9, 5.5 Hz, 2H, H3), 1.40 (d, *J* = 1.7 Hz, 3H, H1a, H1b), 1.14 (d, *J* = 10.2 Hz, 3H, H1a, H1b).

^{13}C NMR (126 MHz, MeOD) δ 201.5 (d, *J* = 6.0 Hz, C5), 172.2 (C6"), 163.5 (d, *J* = 8.6 Hz, C10b), 157.1 (dd, *J* = 13.1, 5.5 Hz, C2'), 155.1 (dd, *J* = 12.7, 5.5 Hz, C2'), 143.2 (C6a), 133.7 – 133.4 (m, C4'), 132.9 – 132.5 (m, C1'), 131.3 (d, *J* = 2.4 Hz, C8), 129.1 (d, *J* = 3.1 Hz, C10a), 128.9 (C9), 127.3 (d, *J* = 7.3 Hz, C7), 123.9 (C10), 114.4 – 114.0 (m, C3'), 109.1 (d, *J* = 6.3 Hz, C4a), 105.2 (d, *J* = 21.6 Hz, C1"), 79.3 (C2), 78.3 (d, *J* = 2.9 Hz, C6), 77.2 (d, *J* = 4.6 Hz, C5",

C3”), 75.0, (C2”), 72.9 (d, J = 4.9 Hz, C4”), 52.8 (d, J = 10.5 Hz, C5’), 32.4 (d, J = 5.8 Hz, C3), 27.6 (d, J = 15.0 Hz, C1a, C1b), 25.7 (d, J = 40.7 Hz, C1a, C1b), 16.7 (C4b,c).

HRMS (ESI$^+$): *m/z* calcd. for $[C_{28}H_{28}O_{10}F_2+H]^+$ calcd. 563.1723 found 563.1723.

11.6. Synthetic Procedures for 16c

4-hydroxy-2,3,5,6-tetrafluorobenzyl alcohol[27]

2,3,5,6-tetrafluoro-4-(hydroxymethyl)phenol **(6)**

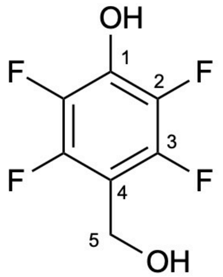

2,3,5,6-tetrafluoro-4-hydroxybenzoic acid (**5**) (3.59 g, 17.1 mmol, 1.0 eq) was dissolved in anhydrous THF (20 mL) and cooled to 0 °C under a N_2 atmosphere. A solution of BH_3•THF (1 M, 85 mL, 85 mmol, 5.0 eq) was added, resulting in gas evolution. The mixture was subsequently refluxed while monitoring by thin-layer chromatography for ~12 h until completion. The reaction was then cooled to 0 °C, and 2M HCl (100 mL) was slowly added, followed by ethyl acetate (200 mL). The organic and aqueous layers were separated, and the aqueous layer was back-extracted with ethyl acetate (100 mL). The organic layers were combined, dried (sodium sulfate), and solvent was removed *in vacuo*. The product was purified by flash column chromatography on silica gel 60 (20-50% ethyl acetate/Pet ether) to give 2,3,5,6-tetrafluoro-4-(hydroxymethyl)phenol (**6**) as a white solid (3.21 g, 16.3 mmol, 96%).

^{1}H NMR (400 MHz, CD_3CN) δ 8.48 (s, 1H, O<u>H</u>), 4.60 – 4.54 (m, 2H, H5).

^{13}C NMR (101 MHz, CD_3CN) δ 147.9 – 147.5 (m), 145.6 – 145.1 (m), 140.3 – 139.9 (m), 137.9 – 137.6 (m), 137.1 – 136.6 (m), 118.5 (C5), 110.8 – 108.8 (m), 53.2 – 51.6 (m).

HRMS (ESI$^+$): *m/z* calcd. for $[C_7H_4O_2F_4]$ calcd. 196.01474 found 196.0146.

NMR spectra were in alignment with the literature procedure.[27]

Aceto-β-glucuronic acid methyl ester 2,3,5,6-tetrafluoro-*para*-hydroxybenzyl alcohol

(2S,3S,4S,5R,6S)-2-(methoxycarbonyl)-6-(2,3,5,6-tetrafluoro-4-(hydroxymethyl)phenoxy)tetrahydro-2H-pyran-3,4,5-triyl triacetate (**13c**)

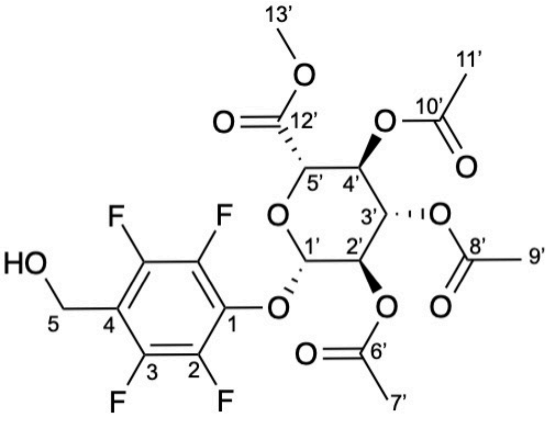

Acetobromo-α-D-glucuronic acid methyl ester (**S2**) (0.127 g, 0.32 mmol, 1 eq) and 2,3,5,6-tetrafluoro-4-(hydroxymethyl)phenol (**6**) (0.100 g, 0.51 mmol, 1.6 eq) were combined in anhydrous acetonitrile (5 mL) under nitrogen in the dark and stirred for 30 min. Ag_2O (0.340 g, 1.46 mmol, 4.5 eq) was added and the reaction was stirred at room temperature overnight in the dark. Thin-layer chromatography monitored reaction progress (3:1 toluene:methanol). The reaction was vacuum filtered through celite, washed with ethyl acetate (100 mL), and the solvent removed *in vacuo*. The residue was dissolved in ethyl acetate (40 mL), washed with sat. Na_2CO_3 (5 × 10 mL), and brine (2 × 10 mL). It was then dried (sodium sulfate), and the solvent was removed *in vacuo* to give pure (2*S*,3*S*,4*S*,5*R*,6*S*)-2-(methoxycarbonyl)-6-(2,3,5,6-tetrafluoro-4-(hydroxymethyl)phenoxy)tetrahydro-2*H*-pyran-3,4,5-triyl triacetate (**13c**) as a pink oil (0.165 g, 0.32 mmol, quantitative).

^{1}H NMR (400 MHz, CD_3CN) δ 5.40 (t, *J* = 9.0 Hz, 1H, H2'), 5.27 (d, *J* = 8.0 Hz, 2H, H1', H3'), 5.19 (t, *J* = 9.7 Hz, 1H, H4'), 4.63 (s, 2H, H5), 4.21 (d, *J* = 9.9 Hz, 1H, H5'), 3.65 (s, 3H, H13'), 2.05 (s, 3H, H11'/H9'/H7'), 1.99 (d, *J* = 2.5 Hz, 6H, H11'/H9'/H7').

^{13}C NMR (101 MHz, CD_3CN) δ 170.6 (C6'/C8'/C10'), 170.5 (C6'/C8'/C10'), 170.2 (C6'/C8'/C10'), 167.8 (C12'), 146.2 (d, *J* = 245.7 Hz, C3), 142.0 (dd, *J* = 247.5, 16.2 Hz, C2), 134.8 (t, *J* = 13.0 Hz, C1), 116.5 (t, *J* = 18.6 Hz, C4), 102.7 (C1'), 72.9 (C5'), 72.0 (C2'), 71.6 (C3'), 69.9 (C4'), 53.4 (C13'), 52.1 (C5), 20.7 (C7'/C9'/C11'), 20.7 (C7'/C9'/C11'), 20.6 (C7'/C9'/C11').

HRMS (ESI$^+$): *m/z* calcd. for [$C_{20}H_{20}O_{11}F_4$] calcd. 512.09417 found 512.0953.

Aceto-β-glucuronic acid methyl ester 2,3,5,6-tetrafluoro-*para*-hydroxybenzyl chloride

(2S,3R,4S,5S,6S)-2-(4-(chloromethyl)-2,3,5,6-tetrafluorophenoxy)-6-(methoxycarbonyl)tetrahydro-2H-pyran-3,4,5-triyl triacetate (**14c**)

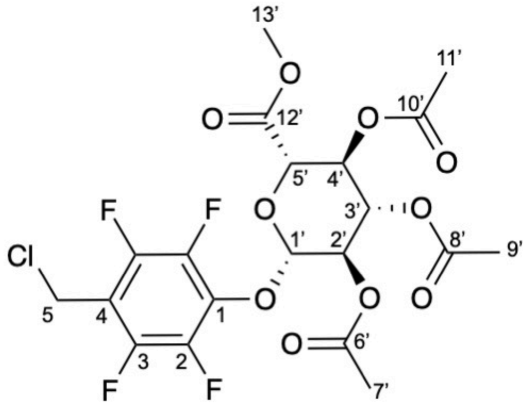

(2*S*,3*S*,4*S*,5*R*,6*S*)-2-(methoxycarbonyl)-6-(2,3,5,6-tetrafluoro-4-(hydroxymethyl) phenoxy)tetrahydro-2*H*-pyran-3,4,5-triyl triacetate (**13c**) (0.165 g, 0.32 mmol, 1 eq) was dissolved in anhydrous dichloromethane (10 mL) at 0°C under N_2. $SOCl_2$ (0.5 mL, 6.8 mmol, 21.4 eq) was added and the reaction was stirred under N_2 overnight. Thin-layer chromatography (3:1 toluene:methanol) showed that some starting material remained, so additional $SOCl_2$ (0.5 mL, 6.8 mmol, 21.4 eq) was added and the reaction stirred for 4 h. LC-MS then showed completion. The reaction was quenched with sat. $NaHCO_3$ (10 mL), washed with brine (10 mL), dried (magnesium sulfate), and the solvent removed *in vacuo* to give (2*S*,3*R*,4*S*,5*S*,6*S*)-2-(4-(chloromethyl)-2,3,5,6-tetrafluorophenoxy)-6-(methoxycarbonyl)tetrahydro-2*H*-pyran-3,4,5-triyl triacetate (**14c**) as an off-white solid (0.185 g, 0.32 mmol, quantitative).

^{1}H NMR (400 MHz, $CDCl_3$) δ 5.29 (d, *J* = 8.6 Hz, 3H, H2', H3', H4'), 5.14 (dd, *J* = 14.1, 4.7 Hz, 1H, H1'), 4.78 – 4.60 (m, 2H, H5), 4.12 – 4.00 (m, 1H, H5'), 3.71 (s, 3H, H13'), 2.08 (s, 3H, H7'/H9'/H11'), 2.02 (s, 3H, H7'/H9'/H11'), 2.01 (s, 3H, H7'/H9'/H11').

^{13}C NMR (101 MHz, $CDCl_3$) δ 170.1 (d, *J* = 2.2 Hz, C6'/C8'/C10'), 169.4 (C6'/C8'/C10'), 169.3 (d, *J* = 4.8 Hz, C6'/C8'/C10'), 166.5 (d, *J* = 3.0 Hz, C12'), 145.2 (d, *J* = 245.7 Hz, C3), 141.1 (dd, *J* = 247.5, 16.2 Hz, C2), 134.6 (t, *J* = 13.0 Hz, C1), 113.6 (dt, *J* = 235.4, 18.3 Hz, C4), 101.7 (d, *J* = 14.7 Hz, C1'), 72.8 (C5'), 71.6 (d, *J* = 7.1 Hz, C3'), 71.0 (d, *J* = 2.6 Hz, C2'), 69.0 (d, *J* = 5.5 Hz, C4'), 53.1 (d, *J* = 1.9 Hz, C13'), 30.7 (d, *J* = 196.8 Hz, C5), 20.6 (C7'/C9'/C11'), 20.5 (C7'/C9'/C11'), 20.5 (C7'/C9'/C11').

HRMS (ESI$^+$): *m/z* calcd. for $[C_{20}H_{19}O_{10}ClF_4+Na]^+$ calcd. 553.0495 found 553.0487.

Aceto-β-glucuronic acid methyl ester 2,3,5,6-tetrafluoro-*para*-hydroxybenzyl β-lapa-ketol[19]

(2S,3S,4S,5R,6S)-2-(methoxycarbonyl)-6-(2,3,5,6-tetrafluoro-4-((6-hydroxy-2,2-dimethyl-5-oxo-3,4,5,6-tetrahydro-2H-benzo[h]chromen-6-yl)methyl)phenoxy)tetrahydro-2H-pyran-3,4,5-triyl triacetate (**15c**)

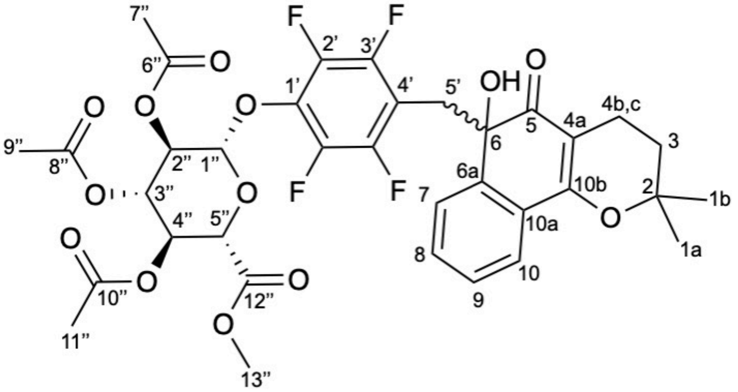

(2*S*,3*R*,4*S*,5*S*,6*S*)-2-(4-(chloromethyl)-2,3,5,6-tetrafluorophenoxy)-6-(methoxycarbonyl) tetrahydro-2*H*-pyran-3,4,5-triyl triacetate (**14c**) (0.185 g, 0.32 mmol, 1.5 eq), NaI (0.153 g, 1.0 mmol, 5 eq), β-lapachone (**1**) (0.05 g , 0.21 mmol, 1 eq) and indium(0) powder (0.047 g, 0.41 mmol, 2 eq) were added to anhydrous dimethylformamide (4 mL). The solution was heated to 40°C and sonicated overnight, while monitoring by thin-layer chromatography (40% ethyl acetate/Pet ether) and LC-MS. Water (10 mL) was added to quench the reaction, and it was extracted with ethyl acetate (3 × 150 mL). The organic layers were combined and washed with brine (10 mL), dried (magnesium sulfate), and the solvent removed *in vacuo*. The product was purified by flash column chromatography on silica gel 60 (40-50% ethyl acetate/Pet ether) to give (2*S*,3*S*,4*S*,5*R*,6*S*)-2-(methoxycarbonyl)-6-(2,3,5,6-tetrafluoro-4-((6-hydroxy-2,2-dimethyl - 5-oxo-3,4,5,6-tetrahydro-2*H*-benzo[*h*]chromen-6-yl)methyl)phenoxy)tetrahydro-2*H*-pyran-3,4,5-triyl triacetate (**15c**) as an off-white solid (0.117 g, 0.15 mmol, 75%).

^{1}H NMR (700 MHz, $CDCl_3$) δ 7.78 – 7.74 (m, 1H, H10), 7.58 (dd, *J* = 11.2, 7.8 Hz, 1H, H7), 7.42 (q, *J* = 6.9 Hz, 1H, H8), 7.36 (t, *J* = 7.6 Hz, 1H, H9), 5.36 – 5.29 (m, 3H, H2”,H3”,H4”), 5.11 (t, *J* = 7.0 Hz, 1H, H1”), 4.79 (s, 1H, O<u>H</u>), 4.07 (d, *J* = 9.2 Hz, 1H, H5”), 3.78 – 3.73 (m, 3H, H13”), 3.11 (dd, *J* = 66.6, 13.4 Hz, 2H, H5’), 2.62 (dt, *J* = 17.4, 6.2 Hz, 1H, H4b,c), 2.38 (dq, *J* = 15.3, 7.3 Hz, 1H, H4b,c), 2.05 (s, 3H, H7”/H9”/H11”), 2.04 (s, 3H, H7”/H9”/H11”), 2.03 (s, 3H, H7”/H9”/H11”), 1.89 – 1.71 (m, 2H, H3), 1.44 (s, 3H, H1a/H1b), 1.40 (d, *J* = 9.0 Hz, 3H, H1a/H1b).

^{13}C NMR (176 MHz, $CDCl_3$) δ 199.4 (C5), 170.1 (C8"), 169.3 (d, J = 23.2 Hz, C10", C6"), 166.5 (d, J = 3.9 Hz, C12''), 161.9 (C10b), 141.3 (C6a), 130.3 (d, J = 6.1 Hz, C8), 128.0 (C9), 126.7 (C10a), 125.4 (C7), 123.6 (C10), 110.1 (C1'), 106.1 (C4a), 101.9 (d, J = 22.1 Hz, C1"), 78.5 (d, J = 3.3 Hz, C6, C2), 73.0 (C5"), 71.8 (d, J = 3.8 Hz, C3"), 71.2 (d, J = 5.5 Hz, C2"), 69.1 (d, J = 8.2 Hz, C4"), 53.0 (C13"), 39.5 (C5'), 31.8 (C3), 27.5 (d, J = 5.8 Hz, C1a/C1b), 25.8 (d, J = 11.6 Hz, C1a/C1b), 20.8 – 19.9 (m, C11''/C9''/C7''), 16.1 (C4b,c).

HRMS (ESI^+): *m/z* calcd. for $[C_{35}H_{34}O_{13}F_4+H]^+$ calcd. 739.2008 found 739.2029.

β-glucuronide-2,3,5,6-tetrafluoro-*para*-hydroxybenzyl β-lapa-ketol[26]

(2S,3S,4S,5R,6S)-3,4,5-trihydroxy-6-(2,3,5,6-tetrafluoro-4-((6-hydroxy-2,2-dimethyl-5-oxo-3,4,5,6-tetrahydro-2H-benzo[h]chromen-6-yl)methyl)phenoxy)tetrahydro-2H-pyran-2-carboxylic acid (**16c**)

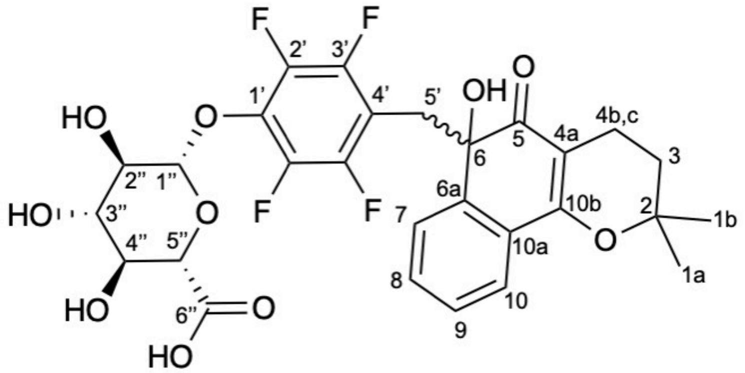

The acetyl-protected glucuronide β-lapachone prodrug (**15c**) (0.117 g, 0.15 mmol, 1 eq) was dissolved in tetrahydrofuran (1 mL) and methanol (1 mL) and stirred at 0°C. A separate solution of LiOH monohydrate (0.039 g, 0.95 mmol, 6 eq) in water (1 mL) was prepared and added to the solution of **15c** dropwise. The reaction was stirred for 1.5 h and product formation was monitored by LC-MS. After complete conversion, glacial acetic acid (54 μL, 0.95 mmol, 6 eq) was added and the solvent removed *in vacuo*. Crude product **16c** was purified by semi-preparative HPLC to give (2*S*,3*S*,4*S*,5*R*,6*S*)-3,4,5-trihydroxy-6-(2,3,5,6-tetrafluoro-4-((6-hydroxy-2,2-dimethyl-5-oxo-3,4,5,6-tetrahydro-2*H*-benzo[*h*]chromen-6-yl)methyl)phenoxy) tetrahydro-2*H*-pyran-2-carboxylic acid (**16c**) as an off-white solid (0.024 g, 0.04 mmol, 27%). HPLC Purification Method: Column = YMC Pack Pro C18 5 μm 250×10 mm 120Å; mobile phases: A = H_2O + 0.1 % formic acid, B = ACN + 0.1 % formic acid; gradient: t = 0-1 min 0% B, t = 1-10 min 0-100% B, t = 10-12 min 100% B. Retention time of **16c** = 9.03 min.

^{1}H NMR (700 MHz, MeOD) δ 7.68 (dd, *J* = 15.0, 7.8 Hz, 1H, H10), 7.62 (dd, *J* = 18.8, 7.6 Hz, 1H, H7), 7.44 (tt, *J* = 7.6, 1.4 Hz, 1H, H8), 7.36 (tdd, *J* = 7.6, 3.1, 1.3 Hz, 1H, H9), 4.98 (dd, *J* = 13.5, 7.5 Hz, 1H, H1”), 3.78 (dd, *J* = 12.8, 9.8 Hz, 1H, H3”), 3.61 (t, *J* = 9.3 Hz, 1H, H4”), 3.50 (t, *J* = 7.6 Hz, 1H, H5”), 3.46 (td, *J* = 9.0, 4.5 Hz, 1H, H2”), 3.19 (dd, *J* = 13.5, 4.3 Hz, 1H, H5’), 3.09 (dd, *J* = 13.5, 6.3 Hz, 1H, H5’), 2.55 (dtd, *J* = 17.2, 5.8, 2.8 Hz, 1H, H4b,c), 2.32 (dddd, *J* = 17.2, 8.5, 6.2, 2.0 Hz, 1H, H4b,c), 1.83 (dtd, *J* = 13.6, 6.0, 1.6 Hz, 1H, H3), 1.75 (ddd, *J* = 14.2, 8.7, 6.1 Hz, 1H, H3), 1.43 (d, *J* = 4.8 Hz, 3H, H1a/H1b), 1.32 (d, *J* = 2.8 Hz, 3H, H1a/H1b).

^{13}C NMR (176 MHz, MeOD) δ 201.1 (d, *J* = 3.4 Hz, C5), 172.0 (C6”), 163.1 (d, *J* = 3.7 Hz, C10b), 147.7 (d, *J* = 243.6 Hz, 3’), 142.9 (d, *J* = 12.0 Hz, C6a), 142.6 (C1’), 138.1 (d, *J* = 1119.5 Hz, C2’), 131.1 (d, *J* = 2.4 Hz, C8), 128.9 (d, *J* = 4.0 Hz, C10a), 128.3 (d, *J* = 2.1 Hz, C9), 126.9 (d, *J* = 7.4 Hz, C7), 124.0 (d, *J* = 9.2 Hz, C10), 110.5 (d, *J* = 18.8 Hz, C4’), 108.2 (d, *J* = 17.0 Hz, C4a), 105.2 (d, *J* = 21.3 Hz, C1”), 79.5 (d, *J* = 4.9 Hz, C2), 78.5 (C6), 77.2 (d, *J* = 5.5 Hz, C5”, C3”), 74.9 (d, *J* = 3.2 Hz, C2”), 72.9 (d, *J* = 7.4 Hz, C4”), 39.7 (d, *J* = 5.6 Hz, C5’), 32.6 (d, *J* = 4.9 Hz, C3), 27.6 (d, *J* = 9.9 Hz, C1a, C1b), 25.9 (d, *J* = 8.4 Hz, C1a, C1b), 17.0 (C4b,c).

HRMS (ESI^{+}): *m/z* calcd. for $[C_{28}H_{26}O_{10}F_4+H]^{+}$ calcd. 599.1535 found 599.1529.

11.7. Synthetic Procedures for 16d

Aceto-β-glucuronic acid methyl ester 2,6-difluoro-*para*-hydroxybenzaldehyde[24]

(2S,3R,4S,5S,6S)-2-(3,5-difluoro-4-formylphenoxy)-6-(methoxycarbonyl)tetrahydro-2H-pyran-3,4,5-triyl triacetate (**12d**)

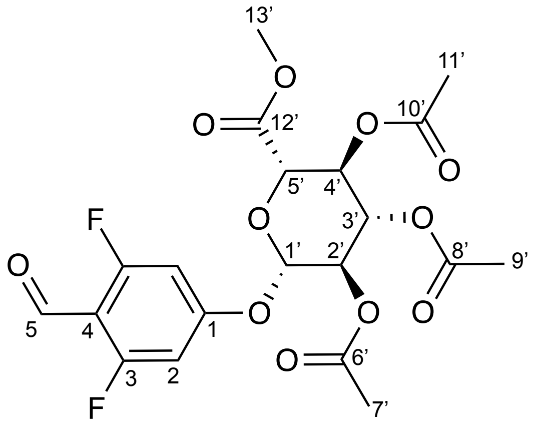

Acetobromo-α-D-glucuronic acid methyl ester (**S2**) (0.503 g, 1.2 mmol, 1 eq) and 2,6-difluoro-4-hydroxybenzaldehyde (**11d**) (0.3345 g, 2.1 mmol, 1.7 eq) were combined in anhydrous acetonitrile (10 mL) under N_2 in the dark and stirred for 30 min. Ag_2O (1.597 g, 6.9 mmol, 5.7 eq) was added and the reaction was stirred at room temperature for 48 h in the dark.

Thin-layer chromatography monitored reaction progress (50% ethyl acetate/Pet ether). The reaction was then filtered through celite and the solvent removed *in vacuo*. The residue was dissolved in ethyl acetate, washed with sat. Na_2CO_3 (10 mL), water (10 mL), and brine (10 mL). It was then dried (sodium sulfate), and the solvent was removed *in vacuo*. The product was purified by flash column chromatography on silica gel 60 (0-100% ethyl acetate/Pet ether) to give (2*S*,3*R*,4*S*,5*S*,6*S*)-2-(3,5-difluoro-4-formylphenoxy)-6-(methoxycarbonyl) tetrahydro-2*H*-pyran-3,4,5-triyl triacetate (**12d**) as an off-white solid (0.3541 g, 0.746 mmol, 59%).

^{1}H NMR (400 MHz, $CDCl_3$) δ 10.15 (s, 1H, H5), 6.60 (d, *J* = 10.2 Hz, 2H, H2), 5.37 – 5.15 (m, 4H, H1', H2', H3', H4'), 4.30 (d, *J* = 8.6 Hz, 1H, H5'), 3.69 (s, 3H, H13'), 2.09 – 1.91 (m, 9H, H7', H9', H11').

^{13}C NMR (101 MHz, $CDCl_3$) δ 183.3 (t, *J* = 4.2 Hz, C5), 169.9 (C8'), 169.4 (C10'), 169.1 (C6'), 166.6 (C12'), 165.5 (d, *J* = 8.6 Hz, C3), 162.9 (d, *J* = 8.6 Hz, C3), 161.9 (t, *J* = 15.1 Hz, C1), 109.9 (t, *J* = 11.1 Hz, C4), 101.1 (d, *J* = 28.8 Hz, C2), 97.6 (C1'), 72.6 (C5'), 71.2 (C3'), 70.6 (C2'), 68.6 (C4'), 53.1 (C13'), 20.5 (C7'), 20.5 (C9'), 20.4 (C11').

HRMS (ESI^{+}): *m/z* calcd. for $[C_{20}H_{20}F_2O_{11}+Na]^+$ calcd. 497.0866 found 497.0870.

Aceto-β-glucuronic acid methyl ester 2,6-difluoro-*para*-hydroxybenzyl alcohol[24]

(2S,3R,4S,5S,6S)-2-(3,5-difluoro-4-(hydroxymethyl)phenoxy)-6-(methoxycarbonyl)tetrahydro-2H-pyran-3,4,5-triyl triacetate (**13d**)

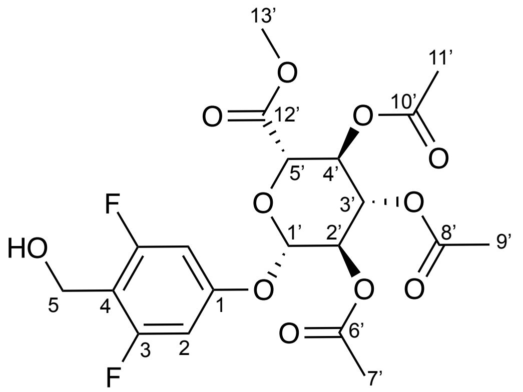

Compound **12d** (0.3541 g, 0.746 mmol, 1 eq) was dissolved in chloroform (5 mL) and isopropanol (1 mL) with silica gel (0.27 g) and stirred at 0°C under N_2 for 15 min. $NaBH_4$ (0.056 g, 1.49 mmol, 2 eq) was added and the reaction was stirred for 60 min. It was monitored by thin-

layer chromatography (1:1 ethyl acetate: Pet ether). The reaction was then quenched with acetone, diluted with dichloromethane (10 mL), filtered over celite, and washed with dichloromethane (10 mL). The filtrate was washed with brine (10 mL), dried (sodium sulfate), and the solvent removed *in vacuo*. NMR of the crude revealed the reduction had over reacted and removed some sugar protecting groups, so the crude was purified by flash column chromatography on silica gel 60 (0-100% ethyl acetate/Pet ether) to give to give (2*S*,3*R*,4*S*,5*S*,6*S*)-2-(3,5-difluoro-4-(hydroxymethyl)phenoxy)-6-(methoxycarbonyl) tetrahydro-2*H*-pyran-3,4,5-triyl triacetate (**13d**) as an off-white solid (0.1284 g, 0.27 mmol, 36%).

^{1}H NMR (400 MHz, MeOD) δ 6.66 (d, *J* = 8.6 Hz, 2H, H2), 5.50 – 5.38 (m, 2H, H1', H3'), 5.30 – 5.18 (m, 2H, H2', H4'), 4.61 – 4.53 (m, 3H, H5, H5'), 3.72 (s, 3H, H13'), 2.08 – 1.96 (m, 9H, H7', H9', H11').

^{13}C NMR (101 MHz, MeOD) δ 171.1 (C8'), 170.9 (C10'), 170.7 (C6'), 168.5 (C12'), 164.3 (d, *J* = 11.2 Hz, C3), 161.8 (d, *J* = 11.3 Hz, C3), 158.4 (t, *J* = 14.4 Hz, C1), 112.3 (t, *J* = 20.4 Hz, C4), 101.2 (d, *J* = 30.6 Hz, C2), 98.7 (C1'), 72.8 (C3'), 72.8 (C5'), 71.9 (C2'), 70.2 (C4'), 53.4 (C13'), 51.9 (t, *J* = 3.9 Hz, C5), 20.5 (C11'), 20.5 (C9'), 20.5 (C7').

HRMS (ESI^{+}): *m/z* calcd. for $[C_{20}H_{22}F_2O_{11}+Na]^+$ calcd. 499.1028 found 499.1053.

Aceto-β-glucuronic acid methyl ester 2,6-difluoro-*para*-hydroxybenzyl bromide[28]

(2S,3R,4S,5S,6S)-2-(4-(bromomethyl)-3,5-difluorophenoxy)-6-(methoxycarbonyl)tetrahydro-2H-pyran-3,4,5-triyl triacetate (**14d**)

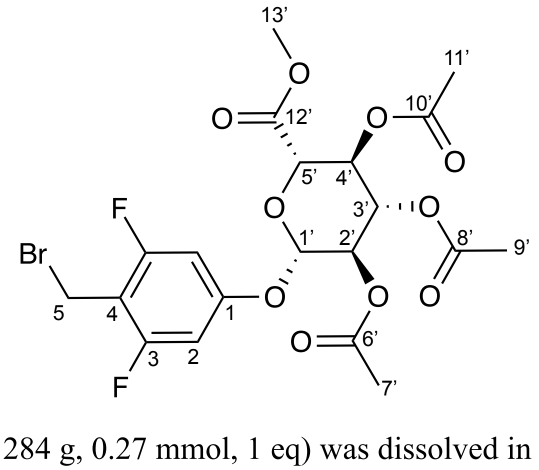

Compound **13d** (0.1284 g, 0.27 mmol, 1 eq) was dissolved in anhydrous dichloromethane (30 mL) at 0°C under N_2. Neat PBr_3 (0.05 mL, 0.54 mmol, 2 eq) was added and the reaction was

stirred under N_2 for 1 h. Thin-layer chromatography (50% ethyl acetate/Pet ether) then showed reaction completion. The reaction was quenched with cold sat. $NaHCO_3$ (10 mL), extracted into DCM (3 × 50 mL) washed with brine (10 mL), dried (sodium sulfate), and the solvent removed *in vacuo* to give (2*S*,3*R*,4*S*,5*S*,6*S*)-2-(4-(bromomethyl)-3,5-difluorophenoxy)-6-(methoxycarbonyl)tetrahydro-2*H*-pyran-3,4,5-triyl triacetate (**14d**) as an unstable yellow solid (0.145 g, 0.27 mmol, quantitative). Due to the instability of the compound, it was used immediately in the next reaction without further purification or characterization.

Aceto-β-glucuronic acid methyl ester 2,6-difluoro-*para*-hydroxybenzyl β-lapa-ketol[19]

(2S,3R,4S,5S,6S)-2-(3,5-difluoro-4-((6-hydroxy-2,2-dimethyl-5-oxo-3,4,5,6-tetrahydro-2H-benzo[h]chromen-6-yl)methyl)phenoxy)-6-(methoxycarbonyl)tetrahydro-2H-pyran-3,4,5-triyl triacetate (**15d**)

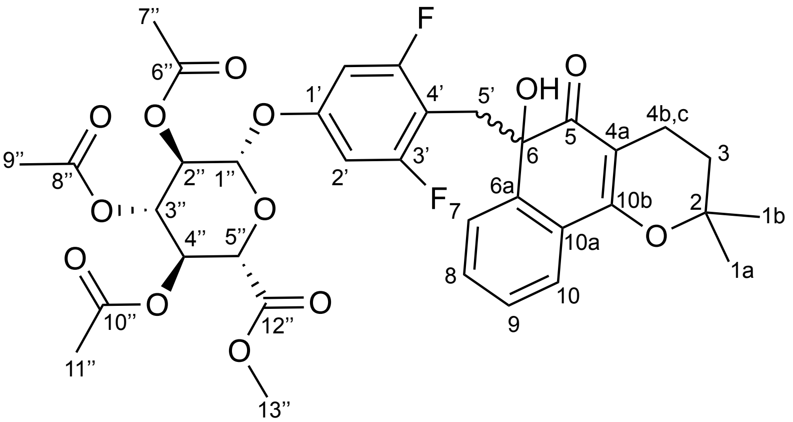

Compound **14d** (0.145 g, 0.27 mmol, 1 eq), NaI (0.1995 g, 1.3 mmol, 4.9 eq), β-lapachone (**1**) (0.071 g, 0.29 mmol, 1.08 eq) and indium(0) powder (0.050 g, 0.43 mmol, 1.6 eq) were added to anhydrous dimethylformamide (3 mL). The solution was sonicated overnight, while monitoring by thin-layer chromatography (40% ethyl acetate/Pet ether) and LC-MS. 1M HCl (~0.5 mL) was added to quench the reaction, and it was extracted with ethyl acetate (3 × 150 mL). The organic layers were combined and washed with brine (10 mL), dried (sodium sulfate), and the solvent removed *in vacuo*. The product was purified by flash column chromatography on silica gel 60 (20-80% ethyl acetate/Pet ether) to give (2*S*,3*R*,4*S*,5*S*,6*S*)-2-(3,5-difluoro-4-((6-hydroxy-2,2-dimethyl-5-oxo-3,4,5,6-tetrahydro-2*H*-benzo[*h*]chromen-6-yl)methyl)phenoxy)-6-(methoxycarbonyl)tetrahydro-2*H*-pyran-3,4,5-triyl triacetate (**15d**) as a peach colored sticky solid (0.0875 g, 0.124 mmol, 46%).

^{1}H NMR (400 MHz, MeOD, mixture of diastereomers 1:1.2) δ 7.63 (dddd, *J* = 13.7, 11.9, 7.8, 1.3 Hz, 2H, H7, H10), 7.41 (tt, *J* = 7.6, 1.3 Hz, 1H, H8), 7.32 (tdd, *J* = 7.5, 3.7, 1.3 Hz, 1H, H9),

6.45 (t, *J* = 8.4 Hz, 2H, H2'), 5.47 – 5.32 (m, 2H, H1", H3"), 5.24 – 5.12 (m, 2H, H2", H4"), 4.53 (dd, *J* = 9.9, 2.9 Hz, 1H, H5"), 3.71 (d, *J* = 2.2 Hz, 3H, H13"), 3.04 (qd, *J* = 13.4, 3.1 Hz, 2H, H5'), 2.51 (dtd, *J* = 17.2, 5.8, 3.8 Hz, 1H, H4b,c), 2.26 (dddd, *J* = 17.4, 9.0, 6.1, 3.2 Hz, 1H, H4b,c), 2.06 – 1.96 (m, 9H, H7", H9", H11"), 1.84 – 1.62 (m, 2H, H3), 1.39 (d, *J* = 6.1 Hz, 3H, H1a/H1b), 1.24 (d, *J* = 4.4 Hz, 3H, H1a/H1b).

^{13}C NMR (101 MHz, MeOD, mixture of diastereomers 1:1.2) δ 201.5 (C5), 201.5 (C5), 171.3 (C8"), 171.1 (C6"), 170.8 (C10"), 170.8 (C10"), 168.7 (C12"), 168.7 (C12"), 164.6 (d, *J* = 11.2 Hz, C3'), 163.1 (C10b), 163.0 (C10b), 162.1 (d, *J* = 11.1 Hz, C3'), 157.8 (td, *J* = 14.3, 3.6 Hz, C1'), 143.3 (C6a), 143.2 (C6a), 131.0 (C8), 131.0 (C8), 128.6 (C9), 128.4 (C10a), 128.4 (C10a), 127.0 (C7), 126.9 (C7), 123.8 (C10), 123.8 (C10), 108.2 (C4a), 108.1 (C4a), 107.1 (td, *J* = 20.6, 9.9 Hz, C4'), 100.7 (dd, *J* = 29.7, 17.6 Hz, C2'), 98.8 (C1"), 98.6 (C1"), 79.2 (C2), 79.2 (C2), 78.7 (C6), 73.1 (C3"), 73.0 (C5"), 72.1 (C2"), 72.1 (C2"), 70.5 (C4"), 70.4 (C4"), 53.4 (C13"), 53.4 (C13"), 39.8 (C5'), 39.8 (C5'), 32.5 (C3), 32.5 (C3), 27.7 (C1a/C1b), 26.1 (C1a/C1b), 20.5 (C7"/C9"/C11"), 20.5 (C7"/C9"/C11"), 20.5 (C7"/C9"/C11"), 20.4 (C7"/C9"/C11"), 16.9 (C4b,c).

HRMS (ESI$^+$): *m/z* calcd. for $[C_{35}H_{36}F_2O_{13}+H]^+$ calcd. 703.2197 found 703.2192.

β-glucuronide-2,6-difluoro-*para*-hydroxybenzyl β-lapa-ketol[26]

(2S,3S,4S,5R,6S)-6-(3,5-difluoro-4-((6-hydroxy-2,2-dimethyl-5-oxo-3,4,5,6-tetrahydro-2H-benzo[h]chromen-6-yl)methyl)phenoxy)-3,4,5-trihydroxytetrahydro-2H-pyran-2-carboxylic acid (**16d**)

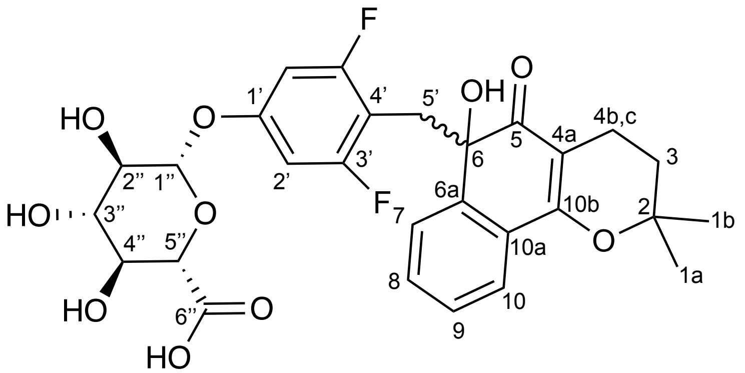

The acetyl-protected glucuronide β-lapachone prodrug (**15d**) (0.0875 g, 0.124 mmol, 1 eq) was dissolved in tetrahydrofuran (2 mL) and methanol (2 mL) and stirred at 0°C. A separate solution of LiOH monohydrate (0.0313 g, 0.744 mmol, 6 eq) in water (1 mL) was prepared and added to the solution of **15d** dropwise. The reaction was stirred for 1.5 h and product formation

was monitored by LC-MS. After complete conversion, glacial acetic acid (42.7 µL, 0.744 mmol, 6 eq) was added and the solvent removed *in vacuo*. Crude product **16d** was purified by semi-preparative HPLC to give (2*S*,3*S*,4*S*,5*R*,6*S*)-6-(3,5-difluoro-4-((6-hydroxy-2,2-dimethyl-5-oxo-3,4,5,6-tetrahydro-2*H*-benzo[*h*] chromen-6-yl)methyl)phenoxy)-3,4,5-trihydroxytetrahydro-2*H*-pyran-2-carboxylic acid (**16d**) as an off-white solid after lyophilization (0.0379 g, 0.067 mmol, 54%). HPLC Purification Method: Column = Agilent InfinityLab ZORBAX 5 Eclipse Plus C18 21.2 × 250 mm; mobile phases: A = H_2O + 0.1% formic acid, B = ACN + 0.1% formic acid**; gradient: t = 0-5 min 5% B, t = 5-35 min 5-95% B, t = 35-40 min 95% B. Retention time of **16d** = 19.0 min.

**Initial purification attempts included 0.1% TFA, but this caused product degradation so 0.1% formic acid was then used.

^{1}H NMR (400 MHz, MeOD, mixture of diastereomers 1:1.2) δ 7.73 – 7.55 (m, 2H, H7, H10), 7.43 (tt, *J* = 7.6, 1.4 Hz, 1H, H8), 7.32 (tt, *J* = 7.6, 1.5 Hz, 1H, H9), 6.47 (dd, *J* = 16.0, 9.2 Hz, 2H, H2'), 4.92 (d, *J* = 7.2 Hz, 1H, H1"), 3.97 (dd, *J* = 9.7, 5.1 Hz, 1H, H5"), 3.59 (td, *J* = 9.3, 2.8 Hz, 1H, H4"), 3.52 – 3.38 (m, 2H, H2", H3"), 3.14 (dd, *J* = 13.1, 2.9 Hz, 1H, H5'), 3.03 (dd, *J* = 13.0, 4.2 Hz, 1H, H5'), 2.51 (ddt, *J* = 16.7, 11.4, 5.9 Hz, 1H, H4b,c), 2.34 – 2.22 (m, 1H, H4b,c), 1.84 – 1.62 (m, 2H, H3), 1.38 (d, *J* = 11.4 Hz, 3H, H1a/H1b), 1.24 (d, *J* = 14.3 Hz, 3H, H1a/H1b).

^{13}C NMR (101 MHz, MeOD, mixture of diastereomers 1:1.2) δ 201.8 (C5), 201.7 (C5), 172.0 (C6"), 164.6 (d, *J* = 9.3 Hz, C3'), 163.4 (C10b), 163.3 (C10b), 162.1 (d, *J* = 9.3 Hz, C3'), 158.8 (t, *J* = 12.5 Hz, C1'), 143.3 (C6a), 143.2 (C6a), 131.0 (C8), 128.6 (C9), 128.6 (C10a), 128.5 (C10a), 127.1 (C7), 127.0 (C7), 123.8 (C10), 123.7 (C10), 108.4 (C4a), 108.3 (C4a), 106.2 (t, *J* = 20.4 Hz, C4'), 102.0 (C1"), 101.6 (C1"), 100.8 (dd, *J* = 36.3, 30.6 Hz, C2'), 79.4 (C2), 79.3 (C2), 78.8 (C6), 77.1 (C3"), 77.0 (C3"), 76.5 (C5"), 74.3 (C2"), 74.2 (C2"), 72.8 (C4"), 39.9 (C5'), 39.8 (C5'), 32.6 (C3), 32.5 (C3), 27.6 (C1a/C1b), 27.5 (C1a/C1b), 26.3 (C1a/C1b), 26.1 (C1a/C1b), 17.0 (C4b,c).

HRMS (ESI$^-$): *m/z* calcd. for $[C_{28}H_{28}F_2O_{10}\text{-}H]^-$ calcd. 561.1578 found 561.1559.

11.8. Synthetic Procedures for 16e

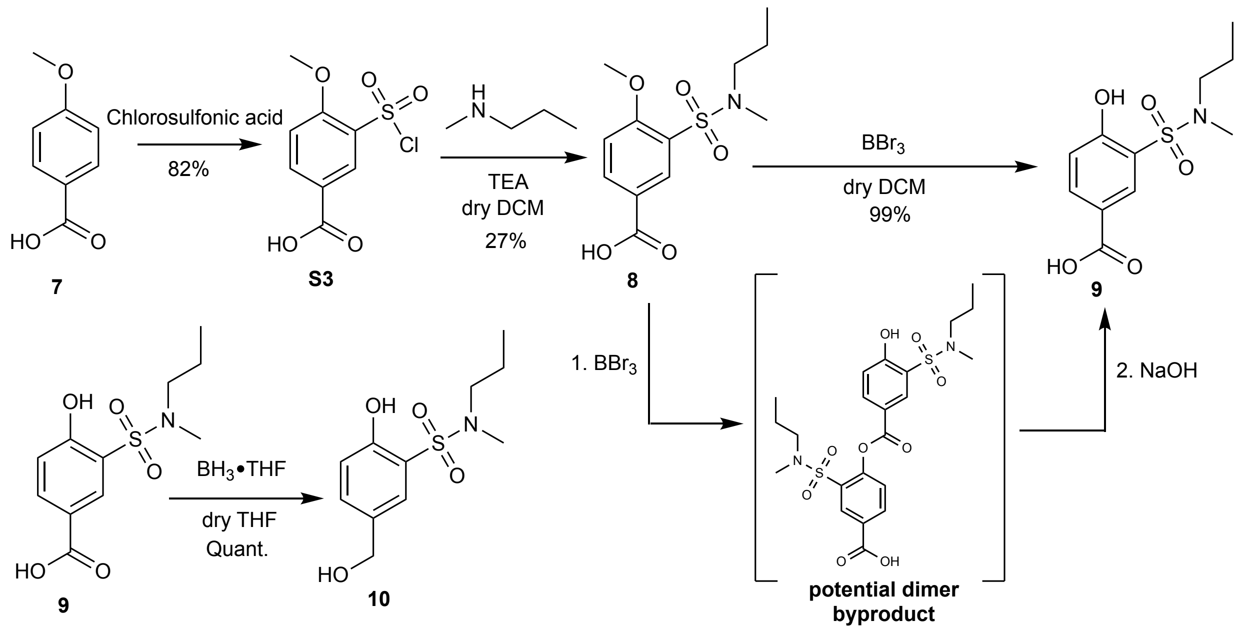

Scheme S2. Synthesis of 10 from 7.

4-methoxy-3-(*N*-methyl-*N*-propylsulfamoyl)benzoic acid[29] (**8**)

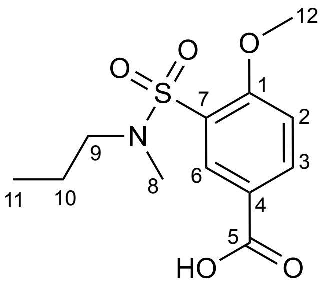

4-Methoxybenzoic acid (**7**) (2 g, 13.2 mmol, 1 eq) was dissolved into chlorosulfonic acid (neat, 6 mL, excess) dropwise at 0 ºC under a nitrogen atmosphere. The yellow solution was heated to 65 ºC for 2 h until it had turned to a dark greenish-brown. It was then cooled to room temperature and poured slowly onto ice in multiple portions, resulting in a white precipitate. The precipitate was collected via vacuum filtration and washed once with ice cold water (5 ml). The crude off-white solid was then redissolved into dichloromethane, dried with sodium sulfate, and the solvent removed *in vacuo* to give 3-(chlorosulfonyl)-4-methoxybenzoic acid as a brown-white solid (2.7 g, 10.8 mmol, 82%). Due to instability, this crude compound **S3** was used immediately in the next step without further purification or characterization.

Crude compound **S3** (2.7 g, 10.8 mmol, 1 eq) was dissolved in dry dichloromethane (15 mL) under a nitrogen atmosphere. *N*-methylpropyl amine (2.1 mL, 20.4 mmol, 1.9 eq) was dissolved in a separate solution of dry dichloromethane (5 mL) under a nitrogen atmosphere. Triethylamine (3.8 mL, 27.2 mmol, 2.5 eq) was added dropwise to the amine solution. Both

solutions were cooled to 0 ºC. The amine solution was then added dropwise to the sulfonyl chloride solution over 10 min, followed by an additional portion of triethylamine (3.8 mL, 27.2 mmol, 2.5 eq). The solution was then warmed to room temperature and stirred overnight until LC-MS showed full conversion. The solvent was removed *in vacuo*, and the crude product was redissolved in ethyl acetate. The solution was washed with HCl (1M, 3 × 10 mL), back-extracted into ethyl acetate (2 × 20 mL), dried with sodium sulfate, and the solvent removed *in vacuo*. The product was purified by flash column chromatography on silica gel 60 (60-100% ethyl acetate/Pet ether + 1% formic acid) to give 4-methoxy-3-(*N*-methyl-*N*-propylsulfamoyl) benzoic acid (**8**) as an off-white solid (1.029 g, 3.58 mmol, 27%).

^{1}H NMR (400 MHz, $CDCl_3$) δ 12.09 (s, 1H, COOH), 8.59 (d, *J* = 2.2 Hz, 1H, H6), 8.20 (dd, *J* = 8.7, 2.2 Hz, 1H, H3), 7.06 (d, *J* = 8.7 Hz, 1H, H2), 3.98 (s, 3H, H12), 3.10 (t, *J* = 7.3 Hz, 2H, H9), 2.82 (s, 3H, H8), 1.54 (h, *J* = 7.4 Hz, 2H, H10), 0.86 (t, *J* = 7.4 Hz, 3H, H11).

^{13}C NMR (101 MHz, $CDCl_3$) δ 170.7 (C5), 160.7 (C1), 136.5 (C3), 133.8 (C6), 127.9 (C7), 121.4 (C4), 112.1 (C2), 56.4 (C12), 51.7 (C9), 34.5 (C8), 21.1 (C10), 10.9 (C11).

HRMS (ESI$^+$): *m/z* calcd. for $[C_{12}H_{17}NO_5S+Na]^+$ calcd. 310.0720 found 310.0723.

3-*N*-methyl-*N*-propylsulfonamide-*para*-hydroxybenzoic acid[30]

4-hydroxy-3-(N-methyl-N-propylsulfamoyl)benzoic acid (**9**)

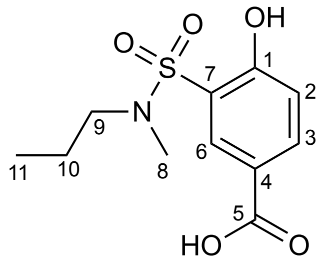

Compound **8** (1.029 g, 3.58 mmol, 1 eq) was dissolved in dry dichloromethane (20 mL) under a nitrogen atmosphere and cooled to -78 ºC. BBr_3 (1M in DCM, 7.2 mL, 7.2 mmol, 2 eq) was added dropwise, and the solution was warmed to room temperature and stirred for 5 h. When LC-MS indicated starting material was still present, and additional 2 eq of BBr_3 were added. The solution was stirred overnight. Once LC-MS indicated completion, the reaction was quenched with water at 0 ºC, extracted with dichloromethane (2 × 20 mL), acidified with HCl (3M, 20 mL), and extracted with dichloromethane (3 × 20 mL). The organic layers were combined, dried

with sodium sulfate, and the solvent removed *in vacuo*. NMR analysis of the crude product indicated the formation of a dimer byproduct, likely due to the formation of an acid bromide during deprotection. An aliquot of the reaction solution dissolved in methanol contained a methyl ester byproduct from reaction of the acid bromide with the methanol, further supporting this conclusion. Therefore, the crude product was stirred in a 10% NaOH solution at 55 ºC for 2 h. The solution was then acidified with HCl (3M) and extracted with dichloromethane (3 × 20 mL). The organic layers were combined, dried with sodium sulfate, and the solvent removed *in vacuo*. This resulted in 4-hydroxy-3-(*N*-methyl-*N*-propylsulfamoyl)benzoic acid (**9**) as an off-white solid (0.9674 g, 3.54 mmol, 99%) without further purification necessary.

^{1}H NMR (400 MHz, $CDCl_3$) δ 8.31 (d, *J* = 2.1 Hz, 1H, H6), 8.16 (dd, *J* = 8.8, 2.1 Hz, 1H, H3), 7.11 (d, *J* = 8.7 Hz, 1H, H2), 3.08 (t, *J* = 6.8 Hz, 2H, H9), 2.81 (s, 3H, H8), 1.59 (h, *J* = 7.3 Hz, 2H, H10), 0.93 (t, *J* = 7.4 Hz, 3H, H11).

^{13}C NMR (101 MHz, $CDCl_3$) δ 170.7 (C5), 159.8 (C1), 136.5 (C3), 131.3 (C6), 121.7 (C4), 121.4 (C7), 119.2 (C2), 51.8 (C9), 34.4 (C8), 20.8 (C10), 11.0 (C11).

HRMS (ESI$^-$): *m/z* calcd. for $[C_{11}H_{15}NO_5S\text{-}H]^-$ calcd. 272.0598 found 272.0594.

3-*N*-methyl-*N*-propylsulfonamide-*para*-hydroxybenzyl alcohol[31]

2-hydroxy-5-(hydroxymethyl)-N-methyl-N-propylbenzenesulfonamide (**10**)

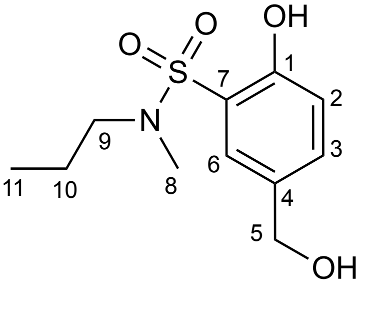

Compound **9** (0.967 g, 3.54 mmol, 1 eq) was dissolved in dry THF (5 mL) under a nitrogen atmosphere. The solution was cooled to 0 ºC. BH_3•THF (1M, 17.7 mL, 17.7 mmol, 5 eq) was added dropwise over 15 min. Once the bubbling had slowed down, the solution was warmed to room temperature and stirred for 4 h until LC-MS analysis indicated no starting material was left. The solution was cooled back down to 0 ºC and slowly quenched with ice cold water in three portions. The solution was then acidified with HCl (3M, 10 mL) and extracted

with dichloromethane (3 × 20 mL). The organic layers were combined, dried with sodium sulfate, and the solvent removed *in vacuo*. This resulted in 2-hydroxy-5-(hydroxymethyl)-*N*-methyl-*N*-propylbenzenesulfonamide (**10**) as a gooey yellow oil (0.92 g, 3.54 mmol, quantitative). This oil was difficult to dry fully due to its viscosity and thus was used as a mostly pure crude with trace THF and DCM still present.

^{1}H NMR (400 MHz, $CDCl_3$) δ 7.47 (d, *J* = 2.2 Hz, 1H, H6), 7.38 (dd, *J* = 8.5, 2.2 Hz, 1H, H3), 6.93 (d, *J* = 8.5 Hz, 1H, H2), 4.54 (s, 2H, H5), 2.97 (t, *J* = 7.2 Hz, 2H, H9), 2.71 (s, 3H, H8), 1.51 (h, *J* = 7.2 Hz, 2H, H10), 0.86 (t, *J* = 7.4 Hz, 3H, H11).

^{13}C NMR (101 MHz, $CDCl_3$) δ 154.3 (C1), 133.7 (C3), 133.2 (C4), 126.3 (C6), 120.3 (C7), 118.5 (C2), 63.4 (C5), 51.4 (C9), 34.1 (C8), 20.5 (C10), 10.7 (C11).

HRMS (ESI$^-$): *m/z* calcd. for $[C_{11}H_{17}NO_4S\text{-}H]^-$ calcd. 258.0806 found 258.0802.

Aceto-β-glucuronic acid methyl ester 3-*N*-methyl-*N*-propylsulfonamide-*para*-hydroxybenzyl alcohol[24]

(2S,3R,4S,5S,6S)-2-(4-(hydroxymethyl)-2-(N-methyl-N-propylsulfamoyl)phenoxy)-6-(methoxycarbonyl)tetrahydro-2H-pyran-3,4,5-triyl triacetate (**13e**)

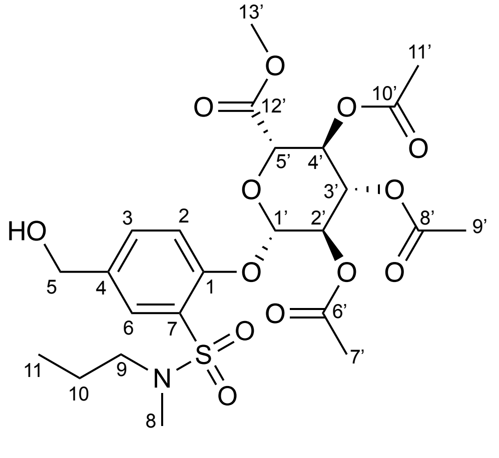

Acetobromo-α-D-glucuronic acid methyl ester (**S2**) (0.615 g, 1.5 mmol, 1 eq) and compound **10** (0.413 g, 1.6 mmol, 1.1 eq) were combined in anhydrous acetonitrile (5 mL) under N_2 in the dark and stirred for 30 min. Ag_2O (0.892 g, 3.75 mmol, 2.5 eq) was added and the reaction was stirred at room temperature for 48 h in the dark. Thin-layer chromatography monitored reaction progress (80% ethyl acetate/Pet ether). The reaction was filtered through

celite, and the solvent removed *in vacuo*. The residue was dissolved in ethyl acetate, washed with sat. Na_2CO_3 (10 mL), water (10 mL), and brine (10 mL). It was then dried (sodium sulfate), and the solvent was removed *in vacuo*. The product was purified by flash column chromatography on silica gel 60 (50-80% ethyl acetate/Pet ether) to give (2*S*,3*R*,4*S*,5*S*,6*S*)-2-(4-(hydroxymethyl)-2-(*N*-methyl-*N*-propylsulfamoyl)phenoxy)-6-(methoxycarbonyl)tetrahydro-2*H*-pyran-3,4,5-triyl triacetate (**13e**) as an off-white sticky solid (0.512 g, 0.88 mmol, 57%).

^{1}H NMR (400 MHz, $CDCl_3$) δ 7.69 (d, *J* = 2.2 Hz, 1H, H6), 7.37 (dd, *J* = 8.6, 2.2 Hz, 1H, H3), 7.09 (d, *J* = 8.5 Hz, 1H, H2), 5.28 – 5.11 (m, 4H, H1', H2', H3', H4'), 4.47 (s, 2H, H5), 4.28 (d, *J* = 9.5 Hz, 1H, H5'), 3.62 (s, 3H, H13'), 3.40 (s, 1H, OH), 2.99 (dt, *J* = 14.4, 7.4 Hz, 1H, H9), 2.82 (ddd, *J* = 13.5, 7.9, 5.7 Hz, 1H, H9), 2.62 (s, 3H, H8), 1.95 – 1.84 (m, 9H, H11', H9', H7'), 1.46 – 1.28 (m, 2H, H10), 0.74 (t, *J* = 7.4 Hz, 3H, H11).

^{13}C NMR (101 MHz, $CDCl_3$) δ 169.8 (C8'), 169.7 (C10'), 169.3 (C6'), 166.7 (C12'), 152.8 (C1), 136.0 (C4), 132.5 (C3), 129.6 (C6), 127.7 (C7), 115.9 (C2), 98.6 (C1'), 71.9 (C5'), 71.9 (C3'), 70.1 (C2'), 68.7 (C4'), 63.1 (C5), 52.8 (C13'), 51.2 (C9), 34.0 (C8), 20.7 (C10), 20.4 (C7'), 20.3 (C9'), 20.2 (C11'), 10.6 (C11).

HRMS (ESI$^+$): *m/z* calcd. for $[C_{24}H_{33}NO_{13}S+Na]^+$ calcd. 600.1580 found 600.1573.

Aceto-β-glucuronic acid methyl ester 3-*N*-methyl-*N*-propylsulfonamide-*para*-hydroxybenzyl bromide[28]

(2S,3R,4S,5S,6S)-2-(4-(bromomethyl)-2-(N-methyl-N-propylsulfamoyl)phenoxy)-6-(methoxycarbonyl) tetrahydro-2H-pyran-3,4,5-triyl triacetate (**14e**)

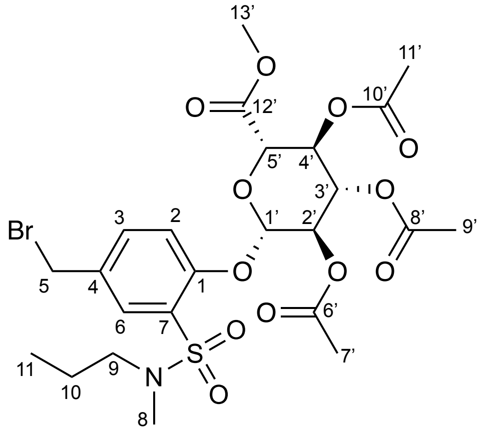

Compound **13e** (0.5116 g, 0.88 mmol, 1 eq) was dissolved in anhydrous dichloromethane (10 mL) at 0°C under N_2. Neat PBr_3 (0.1 mL, 1.0 mmol, 1.2 eq) was added and the reaction was stirred under N_2 for 30 min. Thin-layer chromatography (60% ethyl acetate/Pet ether) then showed reaction completion. The reaction was quenched with cold sat. $NaHCO_3$ (10 mL), extracted into DCM (3 × 50 mL) washed with brine (10 mL), dried (sodium sulfate), and the solvent removed *in vacuo* to give (2*S*,3*R*,4*S*,5*S*,6*S*)-2-(4-(bromomethyl)-2-(*N*-methyl-*N*-propylsulfamoyl)phenoxy)-6-(methoxy carbonyl)tetrahydro-2*H*-pyran-3,4,5-triyl triacetate (**14e**) as an unstable yellow solid (0.562 g, 0.88 mmol, quantitative). Due to the instability of the compound, it was used immediately in the next reaction without further purification or characterization.

Aceto-β-glucuronic acid methyl ester 3-*N*-methyl-*N*-propylsulfonamide-*para*-hydroxybenzyl β-lapa-ketol[19]

(2S,3R,4S,5S,6S)-2-(4-((6-hydroxy-2,2-dimethyl-5-oxo-3,4,5,6-tetrahydro-2H-benzo[h]chromen-6-yl)methyl)-2-(N-methyl-N-propylsulfamoyl)phenoxy)-6-(methoxycarbonyl)tetrahydro-2H-pyran-3,4,5-triyl triacetate (**15e**)

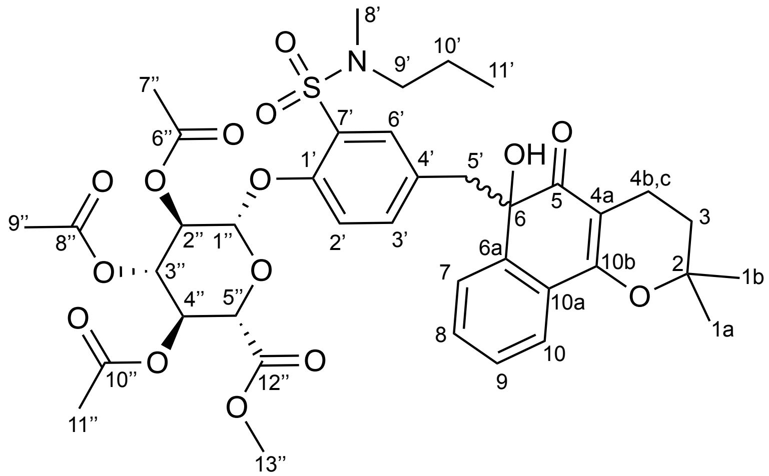

Compound **14e** (0.562 g, 0.88 mmol, 1 eq), NaI (0.544 g, 3.6 mmol, 4 eq), β-lapachone (**1**) (0.214 g, 0.88 mmol, 1 eq) and indium(0) powder (0.155 g, 1.3 mmol, 1.5 eq) were added to anhydrous dimethylformamide (4 mL). The solution was sonicated overnight, while monitoring by thin-layer chromatography (40% ethyl acetate/Pet ether) and LC-MS. 1M HCl (~0.5 mL) was added to quench the reaction, and it was extracted with ethyl acetate (3 × 150 mL). The organic layers were combined and washed with brine (10 mL), dried (sodium sulfate), and the solvent removed *in vacuo*. The product was purified by flash column chromatography on silica gel 60 (40-80% ethyl acetate/Pet ether) to give (2*S*,3*R*,4*S*,5*S*,6*S*)-2-(4-((6-hydroxy-2,2-dimethyl-5-oxo-3,4,5,6-tetrahydro-2*H*-benzo[*h*]chromen-6-yl)methyl)-2-(*N*-methyl-*N*-propylsulfamoyl)phenoxy)

-6-(methoxy carbonyl)tetrahydro-2*H*-pyran-3,4,5-triyl triacetate (**15e**) as an orange sticky solid (0.277 g, 0.345 mmol, 39%).

^{1}H NMR (400 MHz, MeOD, mixture of diastereomers 1:1.6) δ 7.81 – 7.27 (m, 4H, H7, H8, H9, H10), 7.05 (dd, *J* = 39.0, 9.2 Hz, 1H, H2'), 6.98 – 6.69 (m, 2H, H3', H6'), 5.53 – 5.38 (m, 2H, H1", H3"), 5.31 – 5.16 (m, 2H, H2", H4"), 4.67 – 4.54 (m, 1H, H5"), 3.72 (s, 3H, H13"), 3.24 – 2.77 (m, 4H, H5', H9'), 2.64 (d, *J* = 9.5 Hz, 3H, H8'), 2.49 – 2.35 (m, 1H, H4b,c), 2.20 – 2.07 (m, 1H, H4b,c), 2.07 – 1.94 (m, 9H, H7", H9", H11"), 1.73 – 1.41 (m, 4H, H3, H10'), 1.36 (d, *J* = 20.7 Hz, 3H, H1a/H1b), 1.08 (d, *J* = 18.2 Hz, 3H, H1a/H1b), 0.89 (dt, *J* = 16.4, 7.4 Hz, 3H, H11').

^{13}C NMR (101 MHz, MeOD, mixture of diastereomers 1:1.6) δ 201.5 (C5), 201.4 (C5), 171.3 (C6"), 171.2 (C6"), 171.1 (C10"), 171.1 (C10"), 171.0 (C8"), 171.0 (C8"), 168.5 (C12"), 168.4 (C12"), 163.1 (C10b), 163.1 (C10b), 153.9 (C1'), 153.8 (C1'), 143.0 (C6a), 143.0 (C6a), 136.6 (C3'), 136.3 (C3'), 133.3 (C6'), 133.0 (C6'), 131.5 (C8), 131.2 (C8), 131.0 (C4'), 130.5 (C4'), 128.8 (C9), 128.8 (C10a), 128.8 (C10a), 128.7 (C9), 128.1 (C7'), 128.0 (C7'), 127.3 (C7), 127.1 (C7), 124.1 (C10), 123.8 (C10), 115.9 (C2'), 115.6 (C2'), 109.4 (C4a), 108.6 (C4a), 98.8 (C1"), 98.6 (C1"), 79.3 (C2), 79.1 (C2), 78.6 (C6), 78.3 (C6), 73.4 (C3"), 73.4 (C3"), 72.8 (C5"), 72.8 (C5"), 71.7 (C2"), 71.5 (C2"), 70.3 (C4"), 70.2 (C4"), 53.4 (C13"), 53.4 (C13"), 53.0 (C5'), 52.6 (C5'), 52.5 (C9'), 34.9 (C8'), 34.7 (C8'), 32.3 (C3), 27.8 (C1a/C1b), 27.6 (C1a/C1b), 26.5 (C1a/C1b), 25.8 (C1a/C1b), 22.0 (C10'), 21.9 (C10'), 20.9 (C7"/C9"/C11"), 20.8 (C7"/C9"/C11"), 20.5 (C7"/C9"/C11"), 20.5 (C7"/C9"/C11"), 20.5 (C7"/C9"/C11"), 20.4 (C7"/C9"/C11"), 16.6 (C4b,c), 16.5 (C4b,c), 11.3 (C11'), 11.2 (C11').

HRMS (ESI$^+$): *m/z* calcd. for $[C_{39}H_{47}NO_{15}S+Na]^+$ calcd. 825.2591 found 825.2625.

β-glucuronide-3-*N*-methyl-*N*-propylsulfonamide-*para*-hydroxybenzyl β-lapa-ketol[26]

(2S,3S,4S,5R,6S)-3,4,5-trihydroxy-6-(4-((6-hydroxy-2,2-dimethyl-5-oxo-3,4,5,6-tetrahydro-2H-benzo[h]chromen-6-yl)methyl)-2-(N-methyl-N-propylsulfamoyl)phenoxy)tetrahydro-2H-pyran-2-carboxylic acid (**16e**)

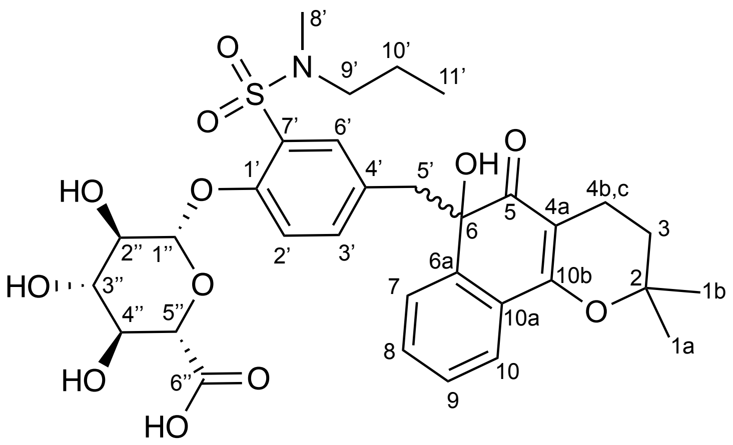

The acetyl-protected glucuronide β-lapachone prodrug (**15e**) (0.277 g, 0.345 mmol, 1 eq) was dissolved in tetrahydrofuran (4 mL) and methanol (4 mL) and stirred at 0°C. A separate solution of LiOH monohydrate (0.086 g, 2.05 mmol, 6 eq) in water (2 mL) was prepared and added to the solution of **15e** dropwise. The reaction was stirred for 1.5 h and product formation was monitored by LC-MS. After complete conversion, glacial acetic acid (118 µL, 2.05 mmol, 6 eq) was added and the solvent removed *in vacuo*. Crude product **16e** was purified by semi-preparative HPLC to give (2*S*,3*S*,4*S*,5*R*,6*S*)-3,4,5-trihydroxy-6-(4-((6-hydroxy-2,2-dimethyl-5-oxo-3,4,5,6-tetrahydro-2*H*-benzo[*h*]chromen-6-yl)methyl)-2-(*N*-methyl-*N*-propylsulfamoyl) phenoxy)tetrahydro-2*H*-pyran-2-carboxylic acid (**16e**) as an off-white solid after lyophilization (0.103 g, 0.156 mmol, 45%, combining both major and minor isomers). HPLC Purification Method: Column = Agilent InfinityLab ZORBAX 5 Eclipse Plus C18 21.2 × 250 mm; mobile phases: A = H_2O + 0.1% formic acid, B = ACN + 0.1% formic acid**; gradient: t = 0-5 min 5% B, t = 5-35 min 5-95% B, t = 35-40 min 95% B. Retention time of major isomer **16e** = 19.1 min. Retention time for minor isomer **16e** = 19.7 min. This was the only prodrug where the two isomers were separable by semi-preparative HPLC.

**Initial purification attempts included 0.1% TFA, but this caused product degradation so 0.1% formic acid was then used.

Major Isomer:

^{1}H NMR (400 MHz, MeOD) δ 7.79 (d, *J* = 7.7 Hz, 1H, H7), 7.57 (t, *J* = 7.1 Hz, 2H, H10, H8), 7.38 (t, *J* = 7.6 Hz, 1H, H9), 7.06 (d, *J* = 8.5 Hz, 1H, H2'), 6.91 (s, 1H, H6'), 6.81 (d, *J* = 8.6 Hz, 1H, H3'), 5.04 (d, *J* = 6.8 Hz, 1H, H1"), 3.99 (d, *J* = 9.6 Hz, 1H, H5"), 3.63 – 3.45 (m, 3H, H2", H3", H4"), 3.23 (d, *J* = 12.2 Hz, 1H, H5'), 3.15 – 3.03 (m, 2H, H5', H9'), 2.90 (dt, *J* = 13.8, 7.0 Hz, 1H, H9'), 2.71 (s, 3H, H8'), 2.43 (dt, *J* = 17.4, 5.9 Hz, 1H, H4b,c), 2.19 (dt, *J* = 17.4, 7.1 Hz, 1H, H4b,c), 1.62 (q, *J* = 6.4 Hz, 2H, H3), 1.55 (dt, *J* = 13.6, 6.8 Hz, 2H, H10'), 1.33 (s, 3H, H1a/H1b), 1.07 (s, 3H, H1a/H1b), 0.95 – 0.89 (m, 3H, H11').

^{13}C NMR (101 MHz, MeOD) δ 201.7 (C5), 171.7 (C6"), 163.4 (C10b), 154.8 (C1'), 143.3 (C6a), 136.7 (C3'), 133.1 (C6'), 131.5 (C8), 130.6 (C4'), 129.1 (C10a), 128.9 (C9), 128.0 (C7'), 127.4 (C7), 123.9 (C10), 116.5 (C2'), 109.6 (C4a), 102.7 (C1"), 79.5 (C2), 78.3 (C6), 76.8 (C5"), 76.7 (C3"), 74.5 (C2"), 72.7 (C4"), 52.9 (C5'), 52.9 (C9'), 35.0 (C8'), 32.4 (C3), 27.3 (C1a/C1b), 26.5 (C1a/C1b), 22.1 (C10'), 16.6 (C4b,c), 11.3 (C11').

HRMS (ESI$^+$): *m/z* calcd. for $[C_{32}H_{39}NO_{12}S+Na]^+$ calcd. 684.2085 found 684.2094.

Minor isomer:

^1H NMR (400 MHz, MeOD) δ 7.67 (ddd, *J* = 11.5, 7.8, 1.3 Hz, 2H, H7, H10), 7.51 (td, *J* = 7.6, 1.4 Hz, 1H, H8), 7.39 (td, *J* = 7.6, 1.3 Hz, 1H, H9), 7.11 (d, *J* = 8.5 Hz, 1H, H2'), 6.92 (dd, *J* = 8.5, 2.3 Hz, 1H, H3'), 6.88 (d, *J* = 2.3 Hz, 1H, H6'), 5.00 (d, *J* = 7.5 Hz, 1H, H1"), 3.98 (d, *J* = 9.6 Hz, 1H, H5"), 3.64 – 3.44 (m, 3H, H2", H3", H4"), 3.16 (d, *J* = 12.5 Hz, 1H, H5'), 3.11 – 3.03 (m, 2H, H5', H9'), 2.98 (dt, *J* = 13.8, 7.0 Hz, 1H, H9'), 2.74 (s, 3H, H8'), 2.45 (dt, *J* = 17.4, 5.8 Hz, 1H, H4b,c), 2.14 (dt, *J* = 17.3, 7.5 Hz, 1H, H4b,c), 1.74 – 1.63 (m, 2H, H3), 1.54 (qd, *J* = 7.3, 3.0 Hz, 2H, H10'), 1.37 (s, 3H, H1a/H1b), 1.12 (s, 3H, H1a/H1b), 0.89 (t, *J* = 7.4 Hz, 3H, H11').

^{13}C NMR (101 MHz, MeOD) δ 201.7 (C5), 171.7 (C6"), 163.4 (C10b), 154.8 (C1'), 143.2 (C6a), 136.8 (C3'), 132.8 (C6'), 131.3 (C8), 130.8 (C4'), 129.0 (C10a), 128.9 (C9), 128.2 (C7'), 127.2 (C10), 124.1 (C7), 116.7 (C2'), 108.9 (C4a), 102.6 (C1"), 79.3 (C2), 78.6 (C6), 76.9 (C3"), 76.6 (C5"), 74.4 (C2"), 72.6 (C4"), 52.8 (C9'), 52.6 (C5'), 34.9 (C8'), 32.5 (C3), 27.6 (C1a/C1b), 26.0 (C1a/C1b), 22.0 (C10'), 16.7 (C4b,c), 11.3 (C11').

HRMS (ESI$^-$): *m/z* calcd. for $[C_{32}H_{39}NO_{12}S-H]^-$ calcd. 660.2120 found 660.2109.

11.9. Synthetic Procedures for 16f

Aceto-β-glucuronic acid methyl ester 2-methoxy-*para*-hydroxybenzaldehyde[24]

(2S,3R,4S,5S,6S)-2-(4-formyl-3-methoxyphenoxy)-6-(methoxycarbonyl)tetrahydro-2H-pyran-3,4,5-triyl triacetate (**12f**)

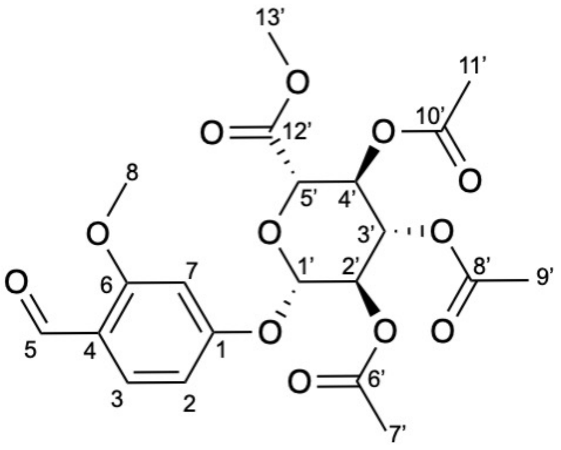

Acetobromo-α-D-glucuronic acid methyl ester (**S2**) (0.503 g, 1.2 mmol, 1 eq) and 2-methoxy-4-hydroxybenzaldehyde (**11f**) (0.307 g, 2.0 mmol, 1.67 eq) were combined in anhydrous acetonitrile (15 mL) under N_2 in the dark and stirred for 30 min. Ag_2O (1.32 g, 5.7 mmol, 4.7 eq) was added and the reaction was stirred at room temperature overnight in the dark. Thin-layer chromatography monitored reaction progress (40% ethyl acetate/Pet ether). The reaction was filtered through celite, and the solvent removed *in vacuo*. The residue was dissolved in ethyl acetate, washed with sat. Na_2CO_3 (10 mL), water (10 mL), and brine (10 mL). It was then dried (magnesium sulfate), and the solvent was removed *in vacuo*. The product was purified by flash column chromatography on silica gel 60 (25-60% ethyl acetate/Pet ether) to give (2*S*,3*R*,4*S*,5*S*,6*S*)-2-(4-formyl-3-methoxyphenoxy)-6-(methoxycarbonyl) tetrahydro-2*H*-pyran-3,4,5-triyl triacetate (**12f**) as a yellow solid (0.403 g, 0.86 mmol, 68%).

^{1}H NMR (400 MHz, $CDCl_3$) δ 10.17 (s, 1H, H5), 7.64 (d, *J* = 8.6 Hz, 1H, H3), 6.53 (d, *J* = 8.6 Hz, 1H, H2), 6.50 (d, *J* = 2.1 Hz, 1H, H7), 5.33 – 5.16 (m, 4H, H1', H2', H3', H4'), 4.27 (d, *J* = 9.3 Hz, 1H, H5'), 3.78 (s, 3H, H8), 3.61 (s, 3H, H13'), 1.97 – 1.93 (m, 9H, H11', H9', H7').

^{13}C NMR (101 MHz, $CDCl_3$) δ 188.1 (C5), 169.8 (C8'), 169.2 (C10'), 169.1 (C6'), 166.6 (C12'), 163.2 (C1), 162.4 (C4), 130.2 (C3), 120.5 (C6), 107.6 (C2), 100.7 (C7), 97.5 (C1'), 72.2 (C5'), 71.5 (C3'), 70.7 (C2'), 68.7 (C4'), 55.6 (C8), 52.8 (C13'), 20.4 (C7'), 20.4 (C9'), 20.3 (C11').

HRMS (ESI$^+$): *m/z* calcd. for $[C_{21}H_{24}O_{12}+H]^+$ calcd. 469.1341 found 469.1381.

Aceto-β-glucuronic acid methyl ester 2-methoxy-*para*-hydroxybenzyl alcohol[24]

(2S,3R,4S,5S,6S)-2-(4-(hydroxymethyl)-3-methoxyphenoxy)-6-(methoxycarbonyl)tetrahydro-2H-pyran-3,4,5-triyl triacetate (**13f**)

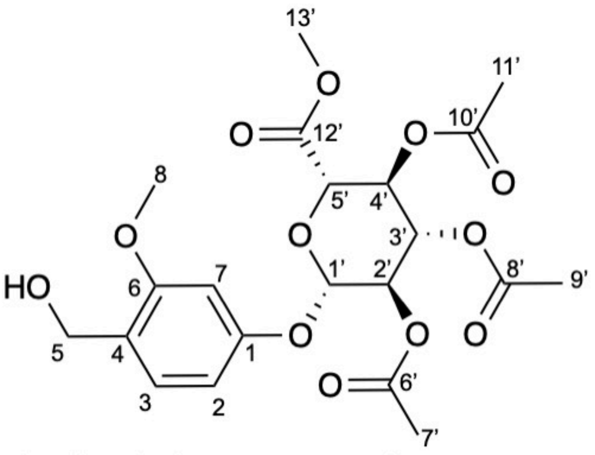

(2*S*,3*R*,4*S*,5*S*,6*S*)-2-(4-formyl-3-methoxyphenoxy)-6-(methoxycarbonyl) tetrahydro-2*H*-pyran -3,4,5-triyl triacetate (**12f**) (0.403 g, 0.86 mmol, 1 eq) was dissolved in chloroform (5 mL) and isopropanol (1 mL) with silica gel (0.4 g) and stirred at 0°C under N_2 for 15 min. $NaBH_4$ (0.065 g, 1.7 mmol, 2 eq) was added and the reaction was stirred for 45 min. It was monitored by thin-layer chromatography (1:1 ethyl acetate: Pet ether). Acetone (10 mL) was added to quench the reaction, and it was stirred for 30 min. The reaction was diluted with dichloromethane (10 mL), filtered over celite, and washed with dichloromethane (10 mL). The filtrate was washed with brine (10 mL), dried (magnesium sulfate), and the solvent removed *in vacuo* to give (2*S*,3*R*,4*S*,5*S*,6*S*)-2-(4-(hydroxymethyl)-3-methoxyphenoxy)-6-(methoxycarbonyl)tetrahydro-2*H*-pyran-3,4,5-triyl triacetate (**13f**) as an off-white solid (0.375 g, 0.79 mmol, 93%).

^{1}H NMR (400 MHz, $CDCl_3$) δ 7.11 (d, *J* = 8.5 Hz, 1H, H3), 6.50 – 6.43 (m, 2H, H2, H7), 5.31 – 5.13 (m, 3H, H2', H3', H4'), 5.10 (d, *J* = 7.5 Hz, 1H, H1'), 4.49 (s, 2H, H5), 4.16 (d, *J* = 9.2 Hz, 1H, H5'), 3.70 (s, 3H, H8), 3.62 (s, 3H, H13'), 2.73 (brs, 1H, O<u>H</u>), 1.96 (d, *J* = 4.1 Hz, 9H, H11', H9', H7').

^{13}C NMR (101 MHz, $CDCl_3$) δ 169.9 (C6'), 169.2 (C8'), 169.1 (C10'), 166.7 (C12'), 157.9 (C1), 157.1 (C4), 128.9 (C3), 124.5 (C6), 107.2 (C2), 100.9 (C7), 98.7 (C1'), 72.2 (C5'), 71.7 (C3'), 70.9 (C2'), 68.9 (C4'), 60.4 (C5), 55.2 (C8), 52.7 (C13'), 20.4 (C11'), 20.4 (C9'), 20.2 (C7').

HRMS (ESI$^+$): *m/z* calcd. for $[C_{21}H_{26}O_{12}+Na]^+$ calcd. 493.1316 found 493.1332.

Aceto-β-glucuronic acid methyl ester 2-methoxy-*para*-hydroxybenzyl bromide[28]

(2S,3R,4S,5S,6S)-2-(4-(bromomethyl)-3-methoxyphenoxy)-6-(methoxycarbonyl)tetrahydro-2H-

pyran-3,4,5-triyl triacetate (**14f**)

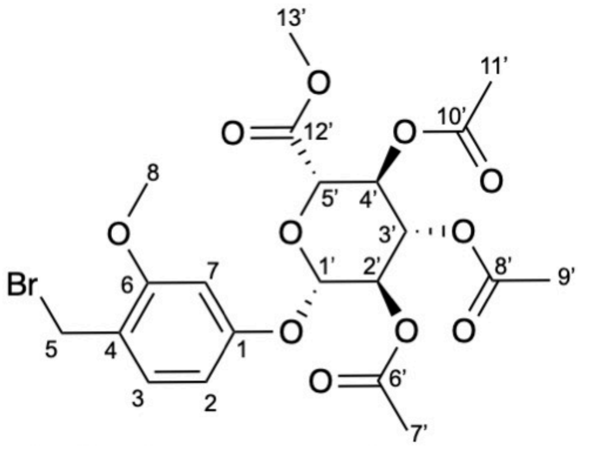

(2*S*,3*R*,4*S*,5*S*,6*S*)-2-(4-(hydroxymethyl)-3-methoxyphenoxy)-6-(methoxycarbonyl) tetrahydro-2*H*-pyran-3,4,5-triyl triacetate (**13f**) (0.375 g, 0.8 mmol, 1 eq) was dissolved in anhydrous diethyl ether (10 mL) at 0°C under N_2. Neat PBr_3 (0.1 mL, 1.0 mmol, 1.3 eq) was added and the reaction was stirred under N_2 for 30 min. Thin-layer chromatography (1:1 Pet ether: ethyl acetate) showed that some starting material remained, so additional PBr_3 (0.05 mL, 0.5 mmol, 0.6 eq) was added and the reaction stirred for 15 min. LC-MS then showed completion. The reaction was quenched with cold water (10 mL), extracted into ether (3 × 50 mL) washed with brine (10 mL), dried (magnesium sulfate), and the solvent removed *in vacuo* to give (2*S*,3*R*,4*S*,5*S*,6*S*)-2-(4-(bromomethyl)-3-methoxyphenoxy)-6-(methoxycarbonyl)tetrahydro -2*H*-pyran-3,4,5-triyl triacetate (**14f**) as an off-white solid (0.401 g, 0.753 mmol, 94%).

^{1}H NMR (400 MHz, $CDCl_3$) δ 7.17 (d, *J* = 8.2 Hz, 1H, H3), 6.53 – 6.47 (m, 2H, H2, H7), 5.34 – 5.18 (m, 3H, H2', H3', H4'), 5.15 (d, *J* = 7.4 Hz, 1H, H1'), 4.45 (d, *J* = 2.8 Hz, 2H, H5), 4.20 (d, *J* = 8.6 Hz, 1H, H5'), 3.78 (s, 3H, H8), 3.66 (s, 3H, H13'), 1.99 (d, *J* = 4.4 Hz, 9H, H11', H9', H7').

^{13}C NMR (101 MHz, $CDCl_3$) δ 169.9 (C6'), 169.3 (C8'), 169.2 (C10'), 166.7 (C12'), 158.4 (C1), 158.1 (C4), 131.5 (C3), 121.3 (C6), 107.6 (C2), 101.4 (C7), 98.5 (C1'), 72.4 (C5'), 71.8 (C3'), 70.9 (C2'), 68.9 (C4'), 55.7 (C8), 52.9 (C13'), 28.8 (C5), 20.5 (C11'), 20.5 (C9'), 20.4 (C7').

HRMS could not be obtained due to compound instability.

Aceto-β-glucuronic acid methyl ester 2-methoxy-*para*-hydroxybenzyl β-lapa-ketol[19]

(2S,3R,4S,5S,6S)-2-(4-((6-hydroxy-2,2-dimethyl-5-oxo-3,4,5,6-tetrahydro-2H-benzo[h]chromen-6-yl)methyl)-3-methoxyphenoxy)-6-(methoxycarbonyl)tetrahydro-2H-pyran-3,4,5-triyl triacetate (**15f**)

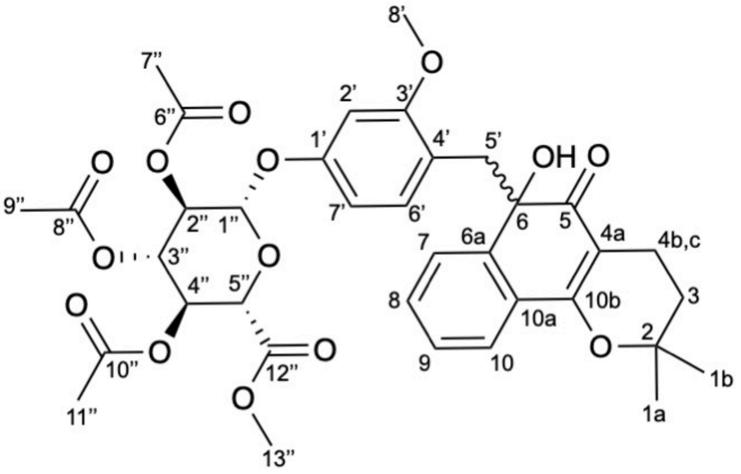

(2*S*,3*R*,4*S*,5*S*,6*S*)-2-(4-(bromomethyl)-3-methoxyphenoxy)-6-(methoxycarbonyl) tetrahydro-2*H*-pyran-3,4,5-triyl triacetate (**14f**) (0.401 g, 0.753 mmol, 1.2 eq), NaI (0.299 g, 2.0 mmol, 3.1 eq), β-lapachone (**1**) (0.154 g , 0.63 mmol, 1 eq) and indium(0) powder (0.087 g, 0.75 mmol, 1.2 eq) were added to anhydrous dimethylformamide (3 mL). The solution was heated to 40°C and sonicated overnight, while monitoring by thin-layer chromatography (50% ethyl acetate/Pet ether) and LC-MS. 1 M HCl (~0.5 mL) was added to quench the reaction, and it was extracted with ethyl acetate (3 × 150 mL). The organic layers were combined and washed with brine (10 mL), dried (magnesium sulfate), and the solvent removed *in vacuo*. The product was purified by flash column chromatography on silica gel 60 (30-70% ethyl acetate/Pet ether) to give (2*S*,3*R*,4*S*,5*S*,6*S*)-2-(4-((6-hydroxy-2,2-dimethyl-5-oxo-3,4,5,6-tetrahydro-2*H*-benzo[*h*] chromen-6-yl)methyl)-3-methoxyphenoxy)-6-(methoxycarbonyl)tetrahydro-2*H*-pyran-3,4,5-triyl triacetate (**15f**) as an orange solid (0.209 g, 0.3 mmol, 47%).

^{1}H NMR (400 MHz, MeOD, mixture of diastereomers 1:1) δ 7.59 (q, *J* = 8.2 Hz, 2H, H7, H10), 7.41 (t, *J* = 7.6 Hz, 1H, H8), 7.29 (t, *J* = 7.6 Hz, 1H, H9), 6.47 (d, *J* = 8.0 Hz, 1H, H6'), 6.41 – 6.25 (m, 2H, H7', H2'), 5.50 – 5.35 (m, 1H, H3"), 5.31 (t, *J* = 8.4 Hz, 1H, H1"), 5.25 – 5.11 (m, 2H, H2", H4"), 4.49 (d, *J* = 4.3 Hz, 1H, H5"), 4.46 (d, *J* = 4.2 Hz, 1H, H5"), 3.69 (d, *J* = 4.6 Hz, 3H, H13"), 3.36 (d, *J* = 7.9 Hz, 3H, H8'), 3.13 (dd, *J* = 99.4, 14.3 Hz, 1H, H8'), 2.95 (t, *J* = 13.2 Hz, 1H, H5'), 2.45 (dt, *J* = 17.4, 5.6 Hz, 1H, H4b,c), 2.11 (ddd, *J* = 17.0, 9.0, 6.5 Hz, 1H, H4b,c), 2.02 (dd, *J* = 7.5, 3.5 Hz, 9H, H7", H9", H11"), 1.74 – 1.55 (m, 2H, H3), 1.34 (d, *J* = 4.1 Hz, 3H, H1a/H1b), 1.10 (d, *J* = 5.9 Hz, 3H, H1a/H1b).

^{13}C NMR (101 MHz, MeOD, mixture of diastereomers 1:1) δ 202.2 (C5), 202.1 (C5), 171.3 (C6"), 171.0 (C10"), 170.8 (C8"), 168.7 (C12"), 168.7 (C12"), 162.9 (C10b), 159.7 (C3'), 158.1

(C1’), 158.0 (C1’), 143.9 (C6a), 143.8 (C6a), 132.9 (C6’), 132.9 (C6’), 130.6 (C8), 128.9 (C10a), 128.8 (C10a), 128.2 (C9), 127.0 (C7/C10), 123.5 (C7/C10), 123.4 (C7/C10), 119.4 (C4’), 119.2 (C4’), 108.5 (C4a), 108.4 (C4a), 107.8 (C2’/C7’), 107.6 (C2’/C7’), 100.8 (C2’/C7’), 100.6 (C2’/C7’), 99.4 (C1”), 99.1 (C1”), 79.1 (C6), 79.1 (C6), 78.9 (C2), 73.3 (C3”), 73.2 (C3”), 72.9 (C5”), 72.3 (C2”), 72.3 (C2”), 70.5 (C4”), 55.6 (C8’), 53.3 (C13”), 46.9 (C5’), 46.8 (C5’), 32.5 (C3), 27.8 (C1a/C1b), 27.8 (C1a/C1b), 26.0 (C1a/C1b), 25.9 (C1a/C1b), 20.6 (C7”/C9”/C11”), 20.5 (C7”/C9”/C11”), 20.5 (C7”/C9”/C11”), 16.8 (C4b,c).

HRMS (ESI$^+$): *m/z* calcd. for $[C_{36}H_{40}O_{14}+Na]^+$ calcd. 719.2310 found 719.2309.

β-glucuronide-2-methoxy-*para*-hydroxybenzyl β-lapa-ketol[26]

(2S,3S,4S,5R,6S)-3,4,5-trihydroxy-6-(4-((6-hydroxy-2,2-dimethyl-5-oxo-3,4,5,6-tetrahydro-2H-benzo[h]chromen-6-yl)methyl)-3-methoxyphenoxy)tetrahydro-2H-pyran-2-carboxylic acid (**16f**)

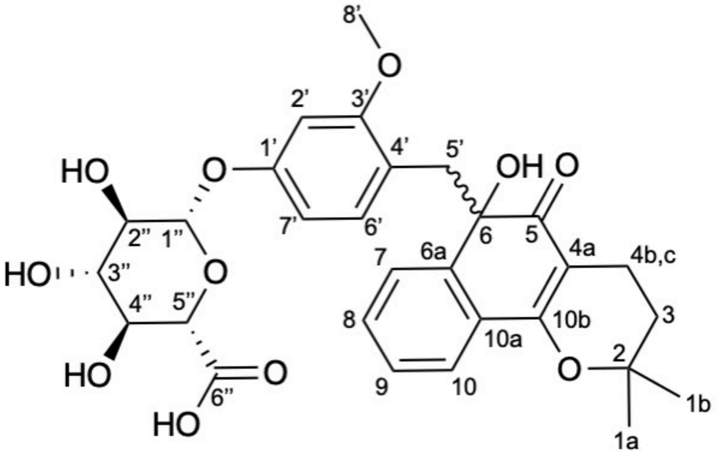

The acetyl-protected glucuronide β-lapachone prodrug (**15f**) (0.209 g, 0.3 mmol, 1 eq) was dissolved in tetrahydrofuran (3 mL) and methanol (3 mL) and stirred at 0°C. A separate solution of LiOH monohydrate (0.075 g, 1.8 mmol, 6 eq) in water (1 mL) was prepared and added to the solution of **15f** dropwise. The reaction was stirred for 1.5 h and product formation was monitored by LC-MS. After complete conversion, glacial acetic acid (103 µL, 1.8 mmol, 6 eq) was added and the solvent removed *in vacuo*. Crude product **16f** was purified by semi-preparative HPLC to give (2*S*,3*S*,4*S*,5*R*,6*S*)-3,4,5-trihydroxy-6-(4-((6-hydroxy-2,2-dimethyl-5-oxo-3,4,5,6-tetrahydro-2*H*-benzo[*h*]chromen-6-yl)methyl)-3-methoxyphenoxy)tetrahydro-2*H*-pyran-2-carboxylic acid (**16f**) as an orange solid after lyophilization (0.123 g, 0.22 mmol, 74%). HPLC Purification Method: Column = YMC Pack Pro C18 5 µm 250×10 mm 120Å; mobile phases: A = H_2O + 0.1 % formic acid, B = ACN + 0.1 % formic acid; gradient: t = 0-1 min 0% B, t = 1-10 min 0-100% B, t = 10-12 min 100% B. Retention time of **16f** = 8.5 min.

^{1}H NMR (400 MHz, MeOD, mixture of diastereomers 1:1) δ 7.66 – 7.53 (m, 2H, H7, H10), 7.42 (tdd, J = 7.6, 3.4, 1.3 Hz, 1H, H8), 7.29 (td, J = 7.6, 1.3 Hz, 1H, H9), 6.49 – 6.30 (m, 3H, H2', H7', H6'), 4.89 (d, J = 7.2 Hz, 1H, H1"), 4.84 (d, J = 7.3 Hz, 1H, H1"), 3.95 (d, J = 4.5 Hz, 1H, H3"), 3.92 (d, J = 4.5 Hz, 1H, H3"), 3.63 (td, J = 9.2, 1.9 Hz, 1H, H4"), 3.56 – 3.43 (m, 2H, H2", H5"), 3.37 (s, 3H, H8'), 3.16 (dd, J = 12.3, 2.3 Hz, 1H, H5'), 2.97 (dd, J = 12.4, 3.0 Hz, 1H, H5'), 2.44 (dq, J = 17.7, 6.0 Hz, 1H, H4b,c), 2.18 – 2.05 (m, 1H, H4b,c), 1.73 – 1.53 (m, 2H, H3), 1.33 (d, J = 8.3 Hz, 3H, H1a/H1b), 1.11 (d, J = 7.8 Hz, 3H, H1a/H1b).

^{13}C NMR (101 MHz, MeOD, mixture of diastereomers 1:1) δ 202.5 (C5), 202.4 (C5), 172.1 (C6"), 172.0 (C6"), 163.2 (C10b), 163.1 (C10b), 159.6 (C3'), 159.6 (C3'), 159.0 (C1'), 158.8 (C1'), 143.9 (C6a), 143.9 (C6a), 132.7 (C6'), 132.6 (C6'), 130.6 (C8), 128.9 (C10a), 128.9 (C10a), 128.2 (C9), 127.0 (C10/C7), 127.0 (C10/C7), 123.4 (C10/C7), 123.4 (C10/C7), 118.7 (C4'), 118.4 (C4'), 108.6 (C4a), 108.6 (C4a), 108.4 (C2'/C7'), 107.9 (C2'/C7'), 102.6 (C1"), 102.0 (C1"), 101.1 (C2'/C7'), 100.6 (C2'/C7'), 79.2 (C6), 79.2 (C6), 79.0 (C2), 78.9 (C2), 77.1 (C2"/C5"), 76.4 (C3"), 74.4 (C2"/C5"), 74.4 (C2"/C5"), 72.8 (C4"), 72.8 (C4"), 55.5 (C8'), 55.5 (C8'), 47.0 (C5'), 46.8 (C5'), 32.5 (C3), 32.4 (C3), 27.7 (C1a/C1b), 27.6 (C1a/C1b), 26.1 (C1a/C1b), 25.9 (C1a/C1b), 16.8 (C4b,c).

HRMS (ESI^{+}): m/z calcd. for $[C_{29}H_{32}O_{11}+Na]^{+}$ calcd. 579.1837 found 579.1860.

11.10. Synthetic Procedures for 16g

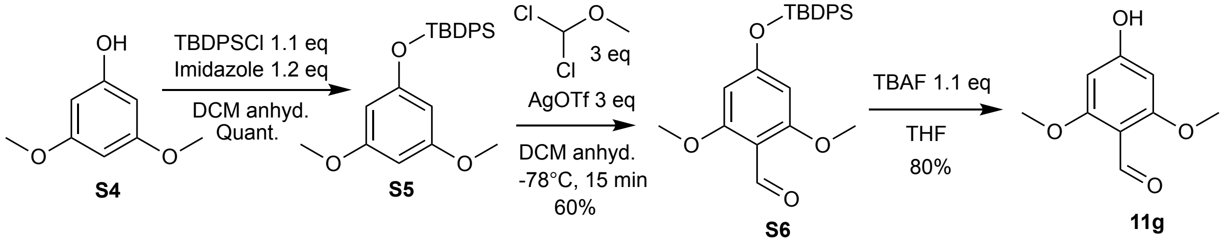

Scheme S3. Synthesis of 11g.

TBDPS-hydroxy-3,5-dimethoxybenzene[32]

tert-butyl(3,5-dimethoxyphenoxy)diphenylsilane (**S5**)

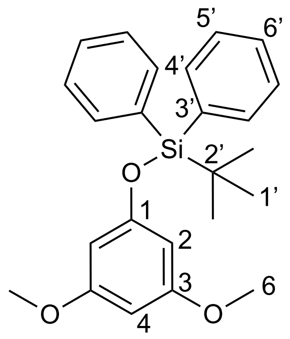

3,5-Dimethoxyphenol (**S4**) (2 g, 13 mmol, 1 eq) and imidazole (2.65 g, 39 mmol, 3 eq) were dissolved in anhydrous DCM (40 mL) under a nitrogen atmosphere and cooled to 0 °C. TBDPSCl (4.1 mL, 15.5 mmol, 1.2 eq) was then added dropwise. The reaction was monitored by thin-layer chromatography (20% ethyl acetate/Pet ether) until completion. Water (10 mL) was then added to quench the reaction, and the product was extracted into DCM (3 × 25 mL). The organic layers were combined, washed with brine (10 mL), dried (sodium sulfate), and the solvent removed *in vacuo*. The product was purified by flash column chromatography on silica gel 60 (10-30% ethyl acetate/Pet ether) to give *tert*-butyl(3,5-dimethoxyphenoxy)diphenylsilane (**S5**) as an off-white solid (5 g, 13 mmol, quantitative).

^{1}H NMR (400 MHz, $CDCl_3$) δ 7.75 – 7.69 (m, 4H, H4'), 7.46 – 7.33 (m, 6H, H5', H6'), 6.01 (t, *J* = 2.2 Hz, 1H, H4), 5.94 (d, *J* = 2.2 Hz, 2H, H2), 3.55 (s, 6H, H6), 1.10 (s, 9H, H1').

^{13}C NMR (101 MHz, $CDCl_3$) δ 161.1 (C3), 157.4 (C1), 135.6 (C4'), 133.1 (C3'), 130.0 (C6'), 127.9 (C5'), 98.6 (C2), 94.1 (C4), 55.2 (C6), 26.7 (C1'), 19.6 (C2').

HRMS (ESI$^+$): *m/z* calcd. for $[C_{24}H_{28}O_3Si+H]^+$ calcd. 393.1880 found 393.1889.

NMR are in accordance with previously reported literature.[33]

4-TBDPS-hydroxy-2,6-dimethoxybenzaldehyde[34]

4-((tert-butyldiphenylsilyl)oxy)-2,6-dimethoxybenzaldehyde (**S6**)

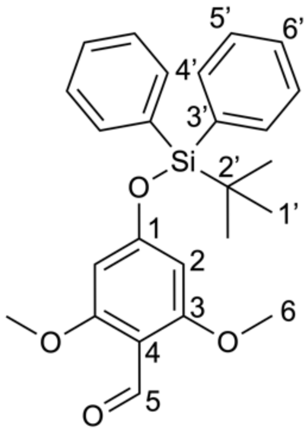

TBDPS-protected 3,5-dimethoxyphenol (**S5**) (0.5 g, 1.27 mmol, 1 eq) and AgOTf (0.985 g, 3.82 mmol, 3 eq) were dissolved in anhydrous dichloromethane (2 mL) under an N_2 atmosphere and cooled to -78 °C. A separate solution of Cl_2CHOMe (0.35 mL, 3.82 mmol, 3 eq) in anhydrous dichloromethane (1 mL) was also prepared and added dropwise to the other solution over 5 min. The reaction was stirred at -78 °C for 15 min and then warmed to 0 °C for 20 min. The reaction was then quenched at 0 °C with saturated $NaHCO_3$ (10 mL) and stirred for an additional 30 min. It was then diluted with DCM, filtered through celite, and extracted with DCM (3 × 20 mL). The combined organic layers were dried (sodium sulfate), and the solvent removed *in vacuo*. The product was purified by flash column chromatography on silica gel 60 (10% ethyl acetate/Pet ether) to give 4-((*tert*-butyldiphenylsilyl)oxy)-2,6-dimethoxy benzaldehyde (**S6**) as an off-white solid (0.267 g, 0.635 mmol, 50%).

^{1}H NMR (400 MHz, $CDCl_3$) δ 10.28 (s, 1H, H5), 7.71 (dd, *J* = 8.0, 1.6 Hz, 4H, H4'), 7.47 – 7.31 (m, 6H, H5', H6'), 5.91 (s, 2H, H2), 3.51 (s, 6H, H6), 1.13 (s, 9H, H1').

^{13}C NMR (101 MHz, $CDCl_3$) δ 187.5 (C5), 163.5 (C3), 162.7 (C1), 135.3 (C4'), 131.9 (C3'), 130.2 (C6'), 127.9 (C5'), 108.9 (C4), 96.1 (C2), 55.5 (C6), 26.4 (C1'), 19.3 (C2').

HRMS (ESI$^+$): *m/z* calcd. for $[C_{25}H_{28}O_4Si+H]^+$ calcd. 421.1830 found 421.1836.

4-hydroxy-2,6-dimethoxybenzaldehyde[35] (**11g**)

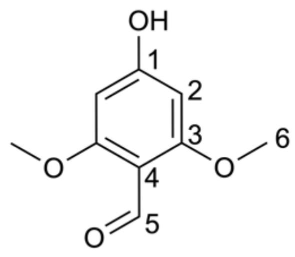

TBDPS-protected 4-hydroxy-2,6-dimethoxybenzaldehyde (**S6**) (0.360 g, 0.85 mmol, 1 eq) was dissolved in anhydrous THF (10 mL). TBAF (1M in THF, 0.94 mL, 0.94 mmol, 1.1 eq) was then added dropwise, causing an immediate color change to orange. The reaction was monitored by thin-layer chromatography (50% ethyl acetate/Pet ether) until completion (1.5 h). Then calcium carbonate (195.4 mg), Dowex resin (586.2 mg), and methanol (1.4 mL) were added and the suspension stirred for 1.5 h at RT. The reaction was then filtered through celite and the celite washed with methanol (3 × 10 mL). The solvent was removed *in vacuo*, and the residue was purified by flash column chromatography on silica gel 60 (50-100% ethyl acetate/Pet ether) to give 4-hydroxy-2,6-dimethoxybenzaldehyde (**11g**) as an off-white, light brown solid (0.22 g, 0.85 mmol, quantitative).

^{1}H NMR (400 MHz, D_2O) δ 10.13 (s, 1H, H5), 6.10 (s, 2H, H2), 3.83 (s, 6H, H6).

^{13}C NMR (101 MHz, D_2O) δ 188.5 (C5), 166.5 (C1), 165.1 (C3), 107.3 (C4), 92.2 (C2), 56.1 (C6).

HRMS (ESI$^-$): *m/z* calcd. for $[C_9H_{10}O_4\text{-}H]^-$ calcd. 181.0506 found 181.0507.

NMR are in accordance with previously reported literature.[36]

Aceto-β-glucuronic acid methyl ester 2,6-dimethoxy-*para*-hydroxybenzaldehyde[24]

(2S,3R,4S,5S,6S)-2-(4-formyl-3,5-dimethoxyphenoxy)-6-(methoxycarbonyl)tetrahydro-2H-pyran-3,4,5-triyl triacetate (**12g**)

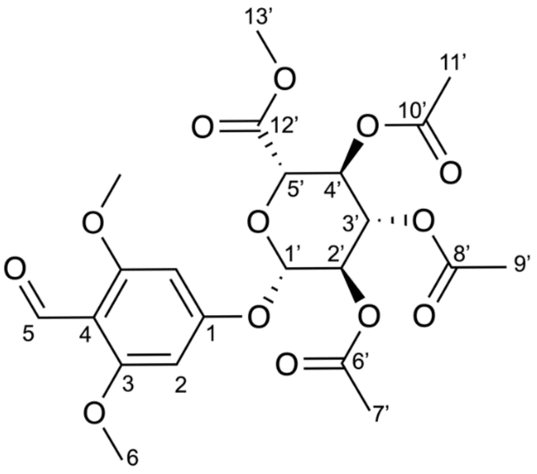

Acetobromo-α-D-glucuronic acid methyl ester (**S2**) (0.627 g, 1.6 mmol, 1 eq) and 4-hydroxy-2,6-dimethoxy-benzaldehyde (**11g**) (0.315 g, 1.7 mmol, 1.1 eq) were combined in anhydrous acetonitrile and anhydrous THF (1:1, 10 mL total) under N_2 in the dark and stirred for 30 min. Ag_2O (0.946 g, 4.0 mmol, 2.5 eq) was added and the reaction was stirred at room temperature overnight in the dark. Thin-layer chromatography monitored reaction progress (50% ethyl acetate/Pet ether). The reaction was filtered through celite, and the solvent removed *in vacuo*. The residue was dissolved in ethyl acetate, washed with sat. Na_2CO_3 (10 mL), water (10 mL), and brine (10 mL). It was then dried (sodium sulfate), and the solvent was removed *in vacuo*. The product was purified by flash column chromatography on silica gel 60 (50% ethyl acetate/Pet ether) to give (2*S*,3*R*,4*S*,5*S*,6*S*)-2-(4-formyl-3,5-dimethoxyphenoxy)-6-(methoxy carbonyl)tetrahydro-2*H*-pyran-3,4,5-triyl triacetate (**12g**) as an off-white solid (0.219 g, 0.44 mmol, 28%).

^{1}H NMR (400 MHz, $CDCl_3$) δ 10.22 (s, 1H, H5), 6.14 (s, 2H, H2), 5.33 – 5.21 (m, 3H, H3', H1', H4'), 5.21 – 5.12 (m, 1H, H2'), 4.30 – 4.20 (m, 1H, H5'), 3.74 (s, 6H, H6), 3.61 (s, 3H, H13'), 1.98 – 1.92 (m, 9H, H11', H9', H7').

^{13}C NMR (101 MHz, $CDCl_3$) δ 187.6 (C5), 169.7 (C8'), 169.1 (C10'), 169.0 (C6'), 166.5 (C12'), 163.6 (C3), 162.4 (C1), 110.1 (C4), 97.6 (C1'), 92.7 (C2), 72.2 (C5'), 71.5 (C3'), 70.8 (C2'), 68.5 (C4'), 55.9 (C6), 52.8 (C13'), 20.4 (C7'), 20.3 (C9'), 20.3 (C11').

HRMS (ESI$^+$): *m/z* calcd. for $[C_{22}H_{26}O_{13}+H]^+$ calcd. 498.13734 found 498.1370.

Aceto-β-glucuronic acid methyl ester 2,6-dimethoxy-*para*-hydroxybenzyl alcohol[24]

(2S,3R,4S,5S,6S)-2-(4-(hydroxymethyl)-3,5-dimethoxyphenoxy)-6-(methoxycarbonyl)tetrahydro-2H-pyran-3,4,5-triyl triacetate (**13g**)

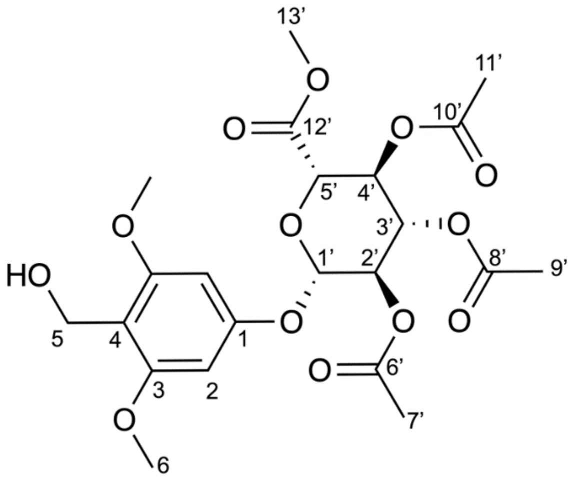

Aceto-β-glucuronic acid methyl ester 2,6-dimethoxy-*para*-hydroxybenzaldehyde (**12g**) (0.286 g, 0.57 mmol, 1 eq) was dissolved in chloroform (5 mL) and isopropanol (1 mL) with silica gel (0.2 g) and stirred at 0°C under N_2 for 15 min. $NaBH_4$ (0.0432 g, 1.1 mmol, 2 eq) was added and the reaction was stirred for 45 min. It was monitored by thin-layer chromatography (1:1 ethyl acetate: Pet ether). The reaction was diluted with dichloromethane (10 mL), filtered over celite, and washed with dichloromethane (10 mL). The filtrate was washed with brine (10 mL), dried (sodium sulfate), and the solvent removed *in vacuo* to give (2*S*,3*R*,4*S*,5*S*,6*S*)-2-(4-(hydroxymethyl)-3,5-dimethoxyphenoxy)-6-(methoxycarbonyl)tetrahydro-2*H*-pyran-3,4,5-triyl triacetate (**13g**) as an off-white solid (0.286 g, 0.57 mmol, 99.5%).

^{1}H NMR (400 MHz, $CDCl_3$) δ 6.20 (s, 2H, H2), 5.31 – 5.26 (m, 2H, H3', H4'), 5.19 (t, *J* = 8.1 Hz, 1H, H2'), 5.12 (d, *J* = 7.4 Hz, 1H, H1'), 4.61 (s, 2H, H5), 4.21 – 4.11 (m, 1H, H5'), 3.73 (s, 6H, H6), 3.66 (s, 3H, H13'), 2.40 (brs, 1H, OH), 2.04 – 1.94 (m, 9H, H11', H9', H7').

^{13}C NMR (101 MHz, $CDCl_3$) δ 170.0 (C6'), 169.2 (C8'), 169.1 (C10'), 166.7 (C12'), 158.9 (C3), 157.7 (C1), 112.5 (C4), 99.1 (C1'), 93.7 (C2), 72.3 (C5'), 71.8 (C3'), 71.0 (C2'), 68.8 (C4'), 55.7 (C6), 53.9 (C5), 52.8 (C13'), 20.5 (C11'), 20.4 (C9'), 20.3 (C7').

HRMS (ESI$^+$): *m/z* calcd. for $[C_{22}H_{28}O_{13}+Na]^+$ calcd. 523.1422 found 523.1439.

Aceto-β-glucuronic acid methyl ester 2,6-dimethoxy-*para*-hydroxybenzyl bromide[37]

(2S,3R,4S,5S,6S)-2-(4-(bromomethyl)-3,5-dimethoxyphenoxy)-6-(methoxycarbonyl)tetrahydro-2H-pyran-3,4,5-triyl triacetate (**14g**)

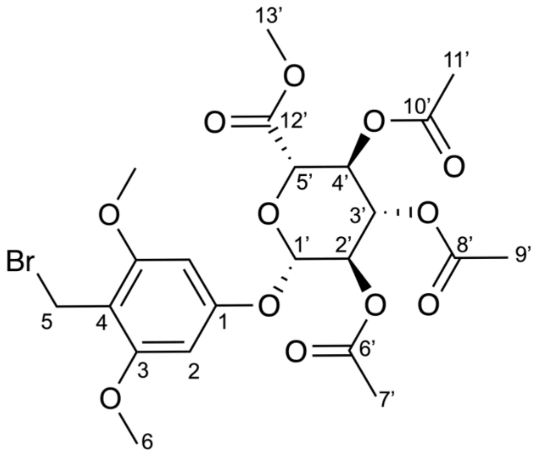

(2*S*,3*R*,4*S*,5*S*,6*S*)-2-(4-(hydroxymethyl)-3,5-dimethoxyphenoxy)-6-(methoxycarbonyl) tetrahydro-2*H*-pyran-3,4,5-triyl triacetate (**13g**) (0.198 g, 0.39 mmol, 1 eq) was dissolved in anhydrous diethyl ether (10 mL) and anhydrous dichloromethane (1 mL) at 0°C under N_2. Neat PBr_3 (0.05 mL, 0.5 mmol, 1.3 eq) was added and the reaction was stirred under N_2 for 30 min. Thin-layer chromatography (1:1 Pet ether: ethyl acetate) showed that some starting material remained, so additional PBr_3 (0.1 mL, 1.0 mmol, 2.6 eq) was added and the reaction stirred for 15 min. Thin-layer chromatography then showed completion. The reaction was quenched with cold water (10 mL), extracted into DCM (3 × 50 mL), washed with brine (10 mL), dried (sodium sulfate), and the solvent removed *in vacuo* to give (2*S*,3*R*,4*S*,5*S*,6*S*)-2-(4-(bromomethyl)-3,5-dimethoxyphenoxy)-6-(methoxycarbonyl)tetrahydro-2*H*-pyran-3,4,5-triyl triacetate (**14g**) as an unstable pink solid (0.223 g, 0.39 mmol, quantitative).

^{1}H NMR (400 MHz, $CDCl_3$) δ 6.18 (s, 2H, H2), 5.36 – 5.27 (m, 2H, H3’, H4’), 5.21 – 5.13 (m, 1H, H2’), 5.10 (dd, *J* = 7.0, 4.9 Hz, 1H, H1’), 4.57 (s, 2H, H5), 4.27 – 4.10 (m, 1H, H5’), 3.79 (s, 6H, H6), 3.67 (s, 3H, H13’), 2.05 – 1.93 (m, 9H, H11’, H9’, H7’).

^{13}C NMR (101 MHz, $CDCl_3$) δ 170.0 (C6’), 169.2 (C8’), 169.2 (C10’), 166.6 (C12’), 159.1 (C3), 158.6 (C1), 109.7 (C4), 98.8 (C1’), 93.5 (C2), 72.4 (C5’), 71.8 (C3’), 71.0 (C2’), 68.8 (C4’), 55.9 (C6), 52.9 (C13’), 23.8 (C5), 20.6 (C11’), 20.5 (C9’), 20.4 (C7’).

HRMS could not be obtained due to compound instability.

Aceto-β-glucuronic acid methyl ester 2,6-dimethoxy-*para*-hydroxybenzyl β-lapa-ketol[19]

(2S,3R,4S,5S,6S)-2-(4-((6-hydroxy-2,2-dimethyl-5-oxo-3,4,5,6-tetrahydro-2H-benzo[h]chromen-6-yl)methyl)-3,5-dimethoxyphenoxy)-6-(methoxycarbonyl)tetrahydro-2H-pyran-3,4,5-triyl triacetate (**15g**)

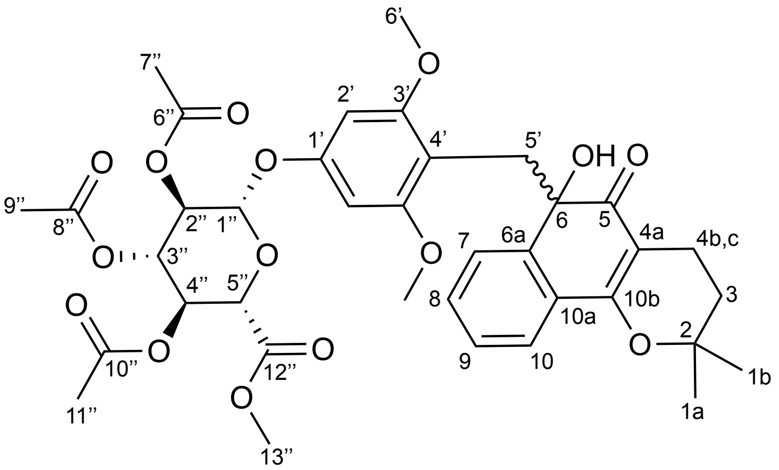

(2*S*,3*R*,4*S*,5*S*,6*S*)-2-(4-(bromomethyl)-3,5-dimethoxyphenoxy)-6-(methoxycarbonyl) tetrahydro-2*H*-pyran-3,4,5-triyl triacetate (**14g**) (0.2662 g, 0.47 mmol, 1 eq), NaI (0.349 g, 2.3 mmol, 4.8 eq), β-lapachone (**1**) (0.1327 g , 0.54 mmol, 1.1 eq) and indium(0) powder (0.0949 g, 0.82 mmol, 1.7 eq) were added to anhydrous dimethylformamide (3 mL). The solution was heated to 40°C and sonicated overnight, while monitoring by thin-layer chromatography (50% ethyl acetate/Pet ether) and LC-MS. 1M HCl (~0.5 mL) was added to quench the reaction, and it was extracted with ethyl acetate (3 × 150 mL). The organic layers were combined and washed with brine (10 mL), dried (sodium sulfate), and the solvent removed *in vacuo*. The product was purified by flash column chromatography on silica gel 60 (50-80% ethyl acetate/Pet ether) to give (2S,3R,4S,5S,6S)-2-(4-((6-hydroxy-2,2-dimethyl-5-oxo-3,4,5,6-tetrahydro-2H-benzo[h] chromen-6-yl)methyl)-3,5-dimethoxyphenoxy)-6-(methoxycarbonyl) tetrahydro-2H-pyran-3,4,5-triyl triacetate (**15g**) as an off-white solid (0.048 g, 0.066 mmol, 14%).

^{1}H NMR (400 MHz, MeOD, mixture of diastereomers 1:1) δ 7.62 (ddd, *J* = 9.4, 7.8, 1.3 Hz, 1H, H7), 7.56 (ddd, *J* = 10.4, 7.8, 1.3 Hz, 1H, H10), 7.38 (tt, *J* = 7.7, 1.2 Hz, 1H, H8), 7.26 (tdd, *J* = 7.5, 3.1, 1.3 Hz, 1H, H9), 6.08 (d, *J* = 6.6 Hz, 2H, H2'), 5.52 – 5.33 (m, 2H, H3", H1"), 5.29 – 5.09 (m, 2H, H2", H4"), 4.57 – 4.44 (m, 1H, H5"), 3.71 (d, *J* = 1.7 Hz, 3H, H13"), 3.40 (d, *J* = 4.2 Hz, 6H, H6'), 3.12 (qd, *J* = 12.5, 1.9 Hz, 2H, H5'), 2.47 (dtd, *J* = 17.1, 5.8, 2.8 Hz, 1H, H4b,c), 2.21 – 2.09 (m, 1H, H4b,c), 2.05 – 1.97 (m, 9H, H7", H9", H11"), 1.78 – 1.56 (m, 2H, H3), 1.35 (d, *J* = 4.9 Hz, 3H, H1a/H1b), 1.16 (d, *J* = 11.6 Hz, 3H, H1a/H1b).

^{13}C NMR (101 MHz, MeOD, mixture of diastereomers 1:1) δ 202.9 (C5), 171.4 (C6”), 171.1 (C6”), 170.9 (C10”), 170.9 (C10”), 168.8 (C8”), 168.7 (C8”), 163.0 (C12”), 163.0 (C12”), 161.4 (C10b), 160.6 (C3’), 160.6 (C3’), 158.5 (C1’), 158.4 (C1’), 144.3 (C6a), 144.3 (C6a), 130.2 (C8), 130.1 (C8), 128.7 (C10a), 128.6 (C10a), 128.0 (C9), 127.3 (C7), 127.3 (C7), 123.2 (C10), 123.2 (C10), 108.1 (C4’), 107.7 (C4a), 107.6 (C4a), 99.6 (C1”), 99.2 (C1”), 93.6 (C2’), 93.3 (C2’), 80.0 (C6), 80.0 (C6), 78.8 (C2), 78.8 (C2), 73.4 (C3”), 73.4 (C3”), 73.0 (C5”), 72.5 (C2”), 72.4 (C2”), 70.6 (C4”), 70.5 (C4”), 55.8 (C6’), 55.8 (C6’), 53.3 (C13”), 40.6 (C5’), 40.5 (C5’), 32.7 (C3), 27.7 (C1a/C1b), 27.7 (C1a/C1b), 26.2 (C1a/C1b), 26.1 (C1a/C1b), 20.6 (C7”/C9”/C11”), 20.5 (C7”/C9”/C11”), 20.4 (C7”/C9”/C11”), 16.9 (C4b,c).

HRMS (ESI$^+$): *m/z* calcd. for $[C_{37}H_{42}O_{15}+Na]^+$ calcd. 726.25237, found 726.2552.

β-glucuronide-2,6-dimethoxy-*para*-hydroxybenzyl β-lapa-ketol[26]

(2S,3S,4S,5R,6S)-3,4,5-trihydroxy-6-(4-((6-hydroxy-2,2-dimethyl-5-oxo-3,4,5,6-tetrahydro-2H-benzo[h]chromen-6-yl)methyl)-3,5-dimethoxyphenoxy)tetrahydro-2H-pyran-2-carboxylic acid (**16g**)

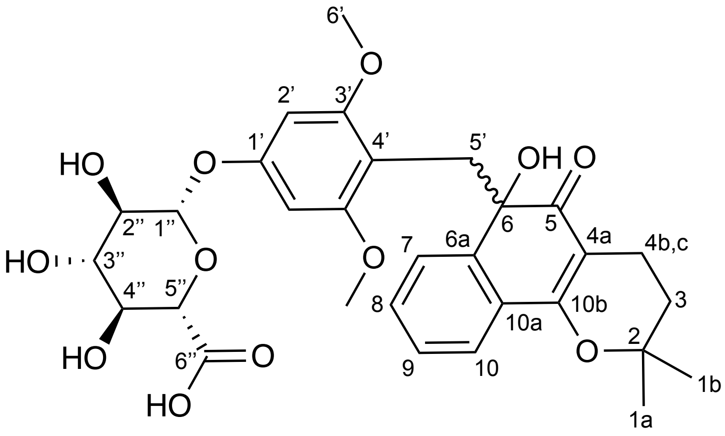

The acetyl-protected glucuronide β-lapachone prodrug (**15g**) (0.048 g, 0.066 mmol, 1 eq) was dissolved in tetrahydrofuran (2 mL) and methanol (2 mL) and stirred at 0°C. A separate solution of LiOH monohydrate (0.016 g, 0.39 mmol, 6 eq) in water (1 mL) was prepared and added to the solution of **15g** dropwise. The reaction was stirred for 1.5 h and product formation was monitored by LC-MS. After complete conversion, glacial acetic acid (22 µL, 0.39 mmol, 6 eq) was added and the solvent removed *in vacuo*. Crude product **16g** was purified by semi-preparative HPLC to give (2*S*,3*S*,4*S*,5*R*,6*S*)-3,4,5-trihydroxy-6-(4-((6-hydroxy-2,2-dimethyl-5-oxo-3,4,5,6-tetrahydro-2*H*-benzo[*h*]chromen-6-yl)methyl)-3,5-dimethoxyphenoxy)tetrahydro-2*H*-pyran-2-carboxylic acid (**16g**) as an orange solid after lyophilization (0.015 g, 0.0255 mmol, 17%). HPLC Purification Method: Column = Agilent InfinityLab ZORBAX 5 Eclipse Plus C18

21.2 × 250 mm; mobile phases: A = H_2O, B = ACN**; gradient: t = 0-5 min 5% B, t = 5-35 min 5-95% B, t = 35-40 min 95% B. Retention time of **16g** = 24.0 min.

**Initial purification attempts included 0.1% formic acid, but this caused product degradation so acid free conditions were then used. Even after immediate flash freezing, trace lapachone was detected in the prodrug stock due to compound instability.

^{1}H NMR (400 MHz, MeOD, mixture of diastereomers 1:1) δ 7.63 (ddd, *J* = 10.4, 7.8, 1.3 Hz, 1H, H7), 7.55 (ddd, *J* = 9.5, 7.8, 1.3 Hz, 1H, H10), 7.39 (tt, *J* = 7.6, 1.6 Hz, 1H, H8), 7.26 (tt, *J* = 7.5, 1.3 Hz, 1H, H9), 6.23 – 6.12 (m, 2H, H2'), 4.89 (d, *J* = 7.4 Hz, 1H, H1"), 3.92 (t, *J* = 9.7 Hz, 1H, H5"), 3.64 – 3.55 (m, 1H, H4"), 3.54 – 3.43 (m, 2H, H3", H2"), 3.41 (d, *J* = 5.1 Hz, 6H, H6'), 3.20 – 3.07 (m, 2H, H5'), 2.47 (ddt, *J* = 17.0, 11.6, 5.9 Hz, 1H, H4b,c), 2.18 (dddd, *J* = 16.9, 8.0, 6.3, 1.6 Hz, 1H, H4b,c), 1.81 – 1.57 (m, 2H, H3), 1.35 (d, *J* = 10.2 Hz, 3H, H1a/H1b), 1.19 (d, *J* = 12.6 Hz, 3H, H1a/H1b).

^{13}C NMR (101 MHz, MeOD, mixture of diastereomers) δ 203.1 (C5), 203.1 (C5), 172.0 (C6"), 163.1 (C10b), 163.0 (C10b), 160.5 (C3'), 160.5 (C3'), 159.5 (C1'), 159.3 (C1'), 144.3 (C6a), 144.3 (C6a), 130.1 (C8), 130.0 (C8), 128.8 (C10a), 128.7 (C10a), 128.0 (C9), 127.3 (C7), 123.2 (C10), 123.2 (C10), 108.2 (C4a), 107.3 (C4'), 107.0 (C4'), 103.1 (C1"), 102.4 (C1"), 94.1 (C2'), 93.5 (C2'), 80.1 (C6), 80.1 (C6), 78.9 (C2), 78.8 (C2), 77.3 (C2"/C3"), 76.5 (C5"), 74.5 (C2"/C3"), 74.5 (C2"/C3"), 72.8 (C4"), 55.7 (C6'), 55.7 (C6'), 40.7 (C5'), 40.6 (C5'), 32.7 (C3), 27.7 (C1a/C1b), 27.6 (C1a/C1b), 26.3 (C1a/C1b), 26.2 (C1a/C1b), 17.0 (C4b,c).

HRMS (ESI$^+$): *m/z* calcd. for $[C_{30}H_{34}O_{12}+Na]^+$ calcd. 609.1942 found 609.1969.

11.11. Synthetic Procedures for 16h

Aceto-β-glucuronic acid methyl ester 3,5-dimethoxy-*para*-hydroxybenzaldehyde[24]

(2S,3R,4S,5S,6S)-2-(4-formyl-2,6-dimethoxyphenoxy)-6-(methoxycarbonyl)tetrahydro-2H-pyran-3,4,5-triyl triacetate (**12h**)

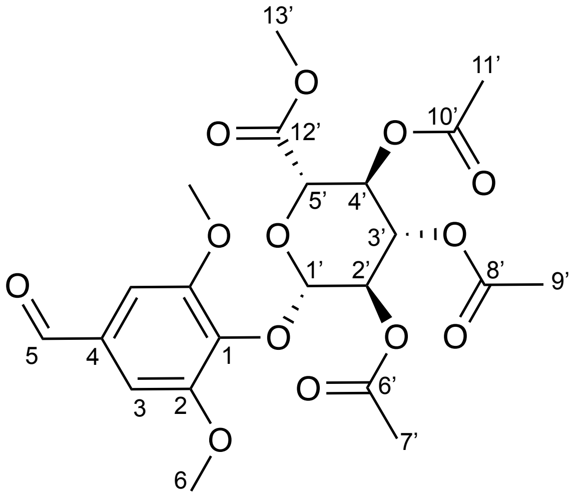

Acetobromo-α-D-glucuronic acid methyl ester (**S2**) (0.652 g, 1.6 mmol, 1 eq) and 3,5-dimethoxy-4-hydroxybenzaldehyde (**11h**) (0.446 g, 2.4 mmol, 1.5 eq) were combined in anhydrous acetonitrile (5 mL) under N_2 in the dark and stirred for 30 min. Ag_2O (0.863 g, 3.7 mmol, 2.3 eq) was added, and the reaction was stirred at room temperature for 48 h in the dark. Thin-layer chromatography monitored reaction progress (50% ethyl acetate/Pet ether). The reaction was then filtered through celite, and the solvent was removed *in vacuo*. The residue was dissolved in ethyl acetate, washed with sat. Na_2CO_3 (10 mL), water (10 mL), and brine (10 mL). It was then dried (sodium sulfate), and the solvent was removed *in vacuo*. The product was purified by flash column chromatography on silica gel 60 (30-100% ethyl acetate/Pet ether) to give (2*S*,3*R*,4*S*,5*S*,6*S*)-2-(4-formyl-2,6-dimethoxyphenoxy)-6-(methoxycarbonyl) tetrahydro-2*H*-pyran-3,4,5-triyl triacetate (**12h**) as an off-white sticky solid (0.4405 g, 0.884 mmol, 54%).

^{1}H NMR (400 MHz, $CDCl_3$) δ 9.84 (s, 1H, H5), 7.09 (s, 2H, H3), 5.60 – 5.48 (m, 1H, H4'), 5.34 – 5.22 (m, 3H, H2', H3', H1'), 4.04 (d, *J* = 10.1 Hz, 1H, H5'), 3.87 (s, 6H, H6), 3.67 (s, 3H, H13'), 2.04 – 1.98 (m, 9H, H7', H9', H11').

^{13}C NMR (101 MHz, $CDCl_3$) δ 191.0 (C5), 170.2 (C8'), 169.4 (C10'), 169.2 (C6'), 167.1 (C12'), 153.6 (C2), 139.0 (C1), 132.9 (C4), 106.7 (C3), 100.1 (C1'), 72.5 (C5'), 72.4 (C3'), 71.9 (C2'), 69.0 (C4'), 56.5 (C6), 52.7 (C13'), 20.7 (C7'), 20.7 (C9'), 20.6 (C11').

HRMS (ESI$^+$): *m/z* calcd. for $[C_{22}H_{26}O_{13}+Na]^+$ calcd. 498.13734 found 498.1373.

Aceto-β-glucuronic acid methyl ester 3,5-dimethoxy-*para*-hydroxybenzyl alcohol[24]

(2S,3R,4S,5S,6S)-2-(4-(hydroxymethyl)-2,6-dimethoxyphenoxy)-6-(methoxycarbonyl)tetrahydro-2H-pyran-3,4,5-triyl triacetate (**13h**)

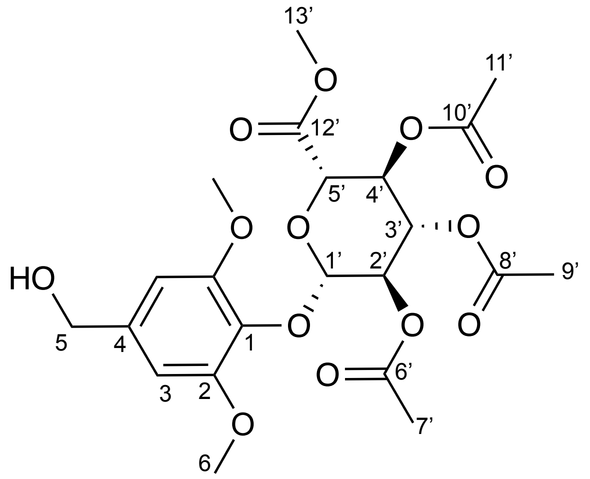

Compound **12h** (0.2118 g, 0.438 mmol, 1 eq) was dissolved in chloroform (10 mL) and isopropanol (1 mL) with silica gel (0.4 g) and stirred at 0°C under N_2 for 15 min. $NaBH_4$ (0.0338 g, 0.88 mmol, 2 eq) was added and the reaction was stirred for 30 min until completion. It was monitored by thin-layer chromatography (1:1 ethyl acetate: Pet ether) and LC-MS. The reaction was then quenched with acetone, diluted with dichloromethane (10 mL), filtered over celite, and washed with dichloromethane (10 mL). The filtrate was washed with brine (10 mL), dried (sodium sulfate), and the solvent removed *in vacuo.* This gave (2*S*,3*R*,4*S*,5*S*,6*S*)-2-(4-(hydroxymethyl)-2,6-dimethoxyphenoxy)-6-(methoxycarbonyl)tetrahydro-2*H*-pyran-3,4,5-triyl triacetate (**13h**) as a yellow solid (0.218 g, 0.436 mmol, 99.5%).

^{1}H NMR (400 MHz, $CDCl_3$) δ 6.50 (s, 2H, H3), 5.36 – 5.30 (m, 1H, H4'), 5.24 – 5.16 (m, 2H, H2', H3'), 5.05 – 5.01 (m, 1H, H1'), 4.51 (s, 2H, H5), 3.93 (d, J = 10.0 Hz, 1H, H5'), 3.71 (s, 6H, H6), 3.60 (s, 3H, H13'), 3.02 (brs, 1H, O<u>H</u>), 1.98 – 1.91 (m, 9H, H7', H9', H11').

^{13}C NMR (101 MHz, $CDCl_3$) δ 170.1 (C8'), 169.4 (C10'), 169.3 (C6'), 167.1 (C12'), 152.8 (C2), 138.4 (C1), 132.9 (C4), 103.7 (C3), 100.8 (C1'), 72.2 (C5'), 72.2 (C3'), 71.6 (C2'), 69.1 (C4'), 64.6 (C5), 56.1 (C6), 52.5 (C13'), 20.5 (C7'), 20.5 (C9'), 20.3 (C11').

HRMS (ESI$^+$): *m/z* calcd. for $[C_{22}H_{28}O_{13}+Na]^+$ calcd. 523.1422 found 523.1429.

Aceto-β-glucuronic acid methyl ester 3,5-dimethoxy-*para*-hydroxybenzyl bromide[28]

(2S,3R,4S,5S,6S)-2-(4-(bromomethyl)-2,6-dimethoxyphenoxy)-6-(methoxycarbonyl)tetrahydro-2H-pyran-3,4,5-triyl triacetate (**14h**)

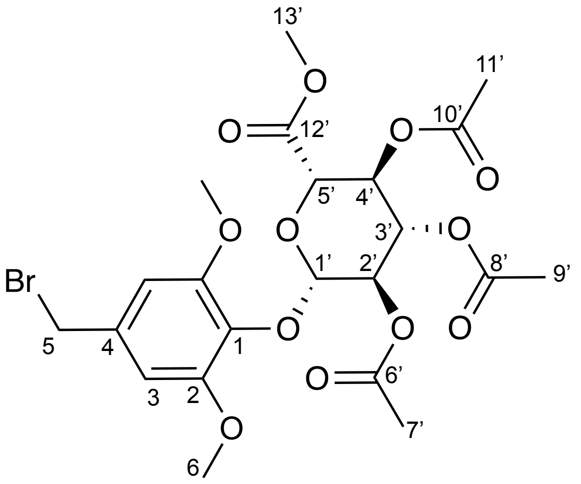

Compound **13h** (0.2185 g, 0.436 mmol, 1 eq) was dissolved in anhydrous dichloromethane (6 mL) and anhydrous THF (6 mL) at 0°C under N_2. Neat PBr_3 (0.1 mL, 1.06 mmol, 2.44 eq) was added and the reaction was stirred under N_2 for 30 min. Thin-layer chromatography (50% ethyl acetate/Pet ether) then showed reaction completion. The reaction was quenched with cold sat. $NaHCO_3$ (10 mL), extracted into DCM (3 × 50 mL), washed with brine (10 mL), dried (sodium sulfate), and the solvent removed *in vacuo* to give (2*S*,3*R*,4*S*,5*S*,6*S*)-2-(4-(bromomethyl)-2,6-dimethoxyphenoxy)-6-(methoxycarbonyl)tetra hydro-2*H*-pyran-3,4,5-triyl triacetate (**14h**) as an unstable cream colored solid (0.245 g, 0.44 mmol, quantitative). Due to the instability of the compound, it was used immediately in the next reaction without further purification or characterization.

Aceto-β-glucuronic acid methyl ester 3,5-dimethoxy-*para*-hydroxybenzyl β-lapa-ketol[19]

(2S,3R,4S,5S,6S)-2-(4-((6-hydroxy-2,2-dimethyl-5-oxo-3,4,5,6-tetrahydro-2H-benzo[h]chromen-6-yl)methyl)-2,6-dimethoxyphenoxy)-6-(methoxycarbonyl)tetrahydro-2H-pyran-3,4,5-triyl triacetate (**15h**)

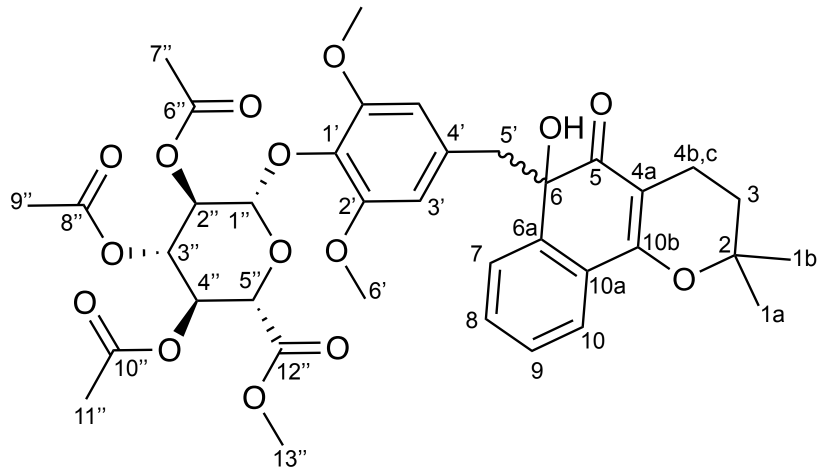

Compound **14h** (0.245 g, 0.436 mmol, 1.02 eq), NaI (0.277 g, 1.8 mmol, 4.3 eq), β-lapachone (**1**) (0.103 g, 0.426 mmol, 1 eq) and indium(0) powder (0.086 g, 0.75 mmol, 1.7 eq) were added to anhydrous dimethylformamide (4 mL). The solution was sonicated overnight, while monitoring by thin-layer chromatography (50% ethyl acetate/Pet ether) and LC-MS. 1M HCl (~0.5 mL) was added to quench the reaction, and it was extracted with ethyl acetate (3 × 150 mL). The organic layers were combined and washed with brine (10 mL), dried (sodium sulfate), and the solvent removed *in vacuo*. The product was purified by flash column chromatography on silica gel 60 (20-80% ethyl acetate/Pet ether) to give (2*S*,3*R*,4*S*,5*S*,6*S*)-2-(4-((6-hydroxy-2,2-dimethyl-5-oxo-3,4,5,6-tetrahydro-2*H*-benzo[*h*]chromen-6-yl)methyl)-2,6-dimethoxyphenoxy)-6-(methoxycarbonyl)tetrahydro-2*H*-pyran-3,4,5-triyl triacetate (**15h**) as an orange colored sticky solid (0.066 g, 0.09 mmol, 21%).

^{1}H NMR (400 MHz, MeOD, mixture of diastereomers 1:1) δ 7.77 (ddd, *J* = 7.8, 3.1, 1.3 Hz, 1H, H7), 7.63 (td, *J* = 7.7, 1.3 Hz, 1H, H10), 7.56 (tt, *J* = 7.6, 1.6 Hz, 1H, H8), 7.42 – 7.36 (m, 1H, H9), 5.69 (d, *J* = 3.9 Hz, 2H, H3’), 5.33 (td, *J* = 9.1, 3.6 Hz, 1H, H3”), 5.21 (td, *J* = 9.6, 3.8 Hz, 1H, H4”), 5.12 (ddd, *J* = 8.9, 7.5, 3.0 Hz, 1H, H2”), 5.06 (dd, *J* = 7.5, 1.6 Hz, 1H, H1”), 4.16 (dd, *J* = 14.7, 10.0 Hz, 1H, H5”), 3.68 (d, *J* = 4.5 Hz, 3H, H13”), 3.52 (d, *J* = 2.1 Hz, 6H, H6’), 3.13 – 2.96 (m, 2H, H5’), 2.43 (dtd, *J* = 17.5, 5.5, 2.8 Hz, 1H, H4b,c), 2.09 – 2.02 (m, 1H, H4b,c), 2.02 – 1.97 (m, 9H, H7”, H9”, H11”), 1.65 – 1.56 (m, 2H, H3), 1.34 (s, 3H, H1a/H1b), 0.97 (d, *J* = 13.1 Hz, 3H, H1a/H1b).

^{13}C NMR (101 MHz, MeOD, mixture of diastereomers 1:1) δ 201.8 (C5), 201.7 (C5), 171.4 (C8”), 171.4 (C8”), 171.1 (C6”), 171.0 (C10”), 170.9 (C10”), 168.9 (C12”), 168.9 (C12”), 163.4 (C10b), 163.3 (C10b), 153.3 (C2’), 153.1 (C2’), 143.7 (C6a), 134.3 (C1’), 134.2 (C1’), 133.4 (C4’), 133.2 (C4’), 131.3 (C8), 131.3 (C8), 129.8 (C10a), 128.7 (C9), 128.6 (C9), 127.5 (C7), 127.4 (C7), 123.6 (C10), 123.6 (C10), 109.4 (C4a), 109.4 (C4a), 107.9 (C3’), 107.6 (C3’), 102.2 (C1”), 102.0 (C1”), 79.2 (C2), 79.1 (C2), 78.7 (C6), 78.7 (C6), 73.4 (C3”), 73.4 (C3”), 73.2

(C5”), 73.2 (C5”), 73.1 (C2”), 73.1 (C2”), 70.8 (C4”), 56.6 (C6’), 56.4 (C6’), 54.4 (C5’), 53.1 (C13”), 53.1 (C13”), 32.4 (C3), 32.3 (C3), 27.7 (C1a/C1b), 27.6 (C1a/C1b), 25.6 (C1a/C1b), 25.4 (C1a/C1b), 20.8 (C7”/C9”/C11”), 20.7 (C7”/C9”/C11”), 20.5 (C7”/C9”/C11”), 20.4 (C7”/C9”/C11”), 16.8 (C4b,c), 16.8 (C4b,c).

HRMS (ESI$^+$): *m/z* calcd. for $[C_{37}H_{42}O_{15}+H]^+$ calcd. 727.2596 found 727.2593.

β-glucuronide-3,5-dimethoxy-*para*-hydroxybenzyl β-lapa-ketol[26]

(2S,3S,4S,5R,6S)-3,4,5-trihydroxy-6-(4-((6-hydroxy-2,2-dimethyl-5-oxo-3,4,5,6-tetrahydro-2H-benzo[h]chromen-6-yl)methyl)-2,6-dimethoxyphenoxy)tetrahydro-2H-pyran-2-carboxylic acid (**16h**)

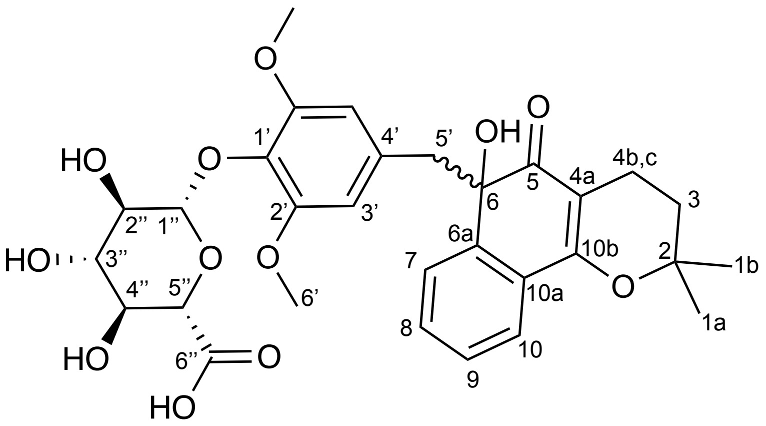

The acetyl-protected glucuronide β-lapachone prodrug (**15h**) (0.066 g, 0.09 mmol, 1 eq) was dissolved in tetrahydrofuran (2 mL) and methanol (2 mL) and stirred at 0°C. A separate solution of LiOH monohydrate (0.0227 g, 0.54 mmol, 6 eq) in water (1 mL) was prepared and added to the solution of **15h** dropwise. The reaction was stirred for 1.5 h and product formation was monitored by LC-MS. After complete conversion, glacial acetic acid (20.7 µL, 0.54 mmol, 6 eq) was added and the solvent removed *in vacuo*. Crude product **16h** was purified by semi-preparative HPLC to give (2*S*,3*S*,4*S*,5*R*,6*S*)-3,4,5-trihydroxy-6-(4-((6-hydroxy-2,2-dimethyl-5-oxo-3,4,5,6-tetrahydro-2*H*-benzo[*h*]chromen-6-yl)methyl)-2,6-dimethoxyphenoxy)tetrahydro-2*H*-pyran-2-carboxylic acid (**16h**) as an off-white solid after lyophilization (0.0366 g, 0.062 mmol, 69%). HPLC Purification Method: Column = Agilent InfinityLab ZORBAX 5 Eclipse Plus C18 21.2 × 250 mm; mobile phases: A = H_2O, B = ACN; gradient: t = 0-5 min 5% B, t = 5-35 min 5-95% B, t = 35-40 min 95% B. Retention time of **16h** = 11.6 min.

^{1}H NMR (400 MHz, MeOD, mixture of diastereomers 1:1) δ 7.78 (ddd, *J* = 7.8, 3.5, 1.3 Hz, 1H, H7), 7.65 (ddd, *J* = 7.9, 4.2, 1.3 Hz, 1H, H10), 7.56 (ddd, *J* = 8.4, 7.4, 1.3 Hz, 1H, H8), 7.40 (tdd, *J* = 7.5, 2.5, 1.3 Hz, 1H, H9), 5.72 (s, 2H, H3'), 4.64 (dd, *J* = 7.6, 6.6 Hz, 1H, H1"), 3.55 (s, 6H, H6'), 3.53 – 3.37 (m, 4H, H2", H3", H4", H5"), 3.15 – 2.96 (m, 2H, H5'), 2.44 (dtd, *J* = 17.3, 5.6, 2.6 Hz, 1H, H4b,c), 2.06 (dt, *J* = 17.3, 7.3 Hz, 1H, H4b,c), 1.66 – 1.57 (m, 2H, H3), 1.35 (s, 3H, H1a/H1b), 1.03 (d, *J* = 4.4 Hz, 3H, H1a/H1b).

^{13}C NMR (101 MHz, MeOD, mixture of diastereomers 1:1) δ 201.9 (C5), 176.1 (C6"), 163.5 (C10b), 163.4 (C10b), 153.3 (C2'), 153.3 (C2'), 143.8 (C6a), 143.8 (C6a), 135.6 (C1'), 135.5 (C1'), 132.7 (C4'), 132.6 (C4'), 131.3 (C8), 129.8 (C10a), 128.7 (C9), 127.5 (C7), 127.5 (C7), 123.7 (C10), 123.7 (C10), 109.5 (C4a), 109.5 (C4a), 108.5 (C3'), 108.2 (C3'), 105.7 (C1"), 105.5 (C1"), 79.3 (C2), 79.2 (C2), 78.7 (C6), 78.7 (C6), 77.6 (C5"), 77.5 (C3"), 77.5 (C3"), 75.5 (C2"), 73.5 (C4"), 56.8 (C6'), 56.7 (C6'), 54.3 (C5'), 32.4 (C3), 27.6 (C1a/C1b), 27.6 (C1a/C1b), 25.9 (C1a/C1b), 25.7 (C1a/C1b), 16.9 (C4b,c).

HRMS (ESI^{+}): *m/z* calcd. for $[C_{30}H_{34}O_{12}+H]^{+}$ calcd. 587.2123 found 587.2142.

11.12. Synthetic Procedures for 16i

Aceto-β-glucuronic acid methyl ester *para*-hydroxybenzyl ketone[24]

(2S,3R,4S,5S,6S)-2-(4-acetylphenoxy)-6-(methoxycarbonyl)tetrahydro-2H-pyran-3,4,5-triyl triacetate (**12i**)

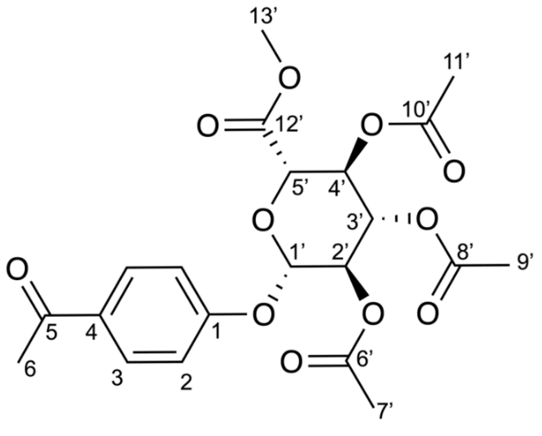

Acetobromo-α-D-glucuronic acid methyl ester (**S2**) (0.501 g, 1.2 mmol, 1 eq) and 4-hydroxyacetophenone (**11i**) (0.289 g, 2.1 mmol, 1.7 eq) were combined in anhydrous acetonitrile (12 mL) under N_2 in the dark and stirred for 30 min. Ag_2O (0.609 g, 2.6 mmol, 2.2 eq) was added, and the reaction was stirred at room temperature overnight in the dark. Thin-layer chromatography monitored reaction progress (40% ethyl acetate/Pet ether). The reaction was filtered through celite, and the solvent was removed *in vacuo*. The residue was dissolved in ethyl

acetate, washed with sat. Na_2CO_3 (10 mL), water (10 mL), and brine (10 mL). It was then dried (sodium sulfate), and the solvent was removed *in vacuo*. The product was purified by flash column chromatography on silica gel 60 (30-40% ethyl acetate/Pet ether) to give (2*S*,3*R*,4*S*,5*S*,6*S*)-2-(4-acetylphenoxy)-6-(methoxycarbonyl)tetrahydro-2*H*-pyran-3,4,5-triyl triacetate (**12i**) as a yellow solid (0.392 g, 0.86 mmol, 74%).

^{1}H NMR (400 MHz, $CDCl_3$) δ 7.80 (d, *J* = 8.9 Hz, 2H, H2), 6.94 (d, *J* = 8.9 Hz, 2H, H3), 5.36 – 5.09 (m, 4H, H1', H2', H3', H4'), 4.27 (d, *J* = 9.4 Hz, 1H, H5'), 3.60 (s, 3H, H13'), 2.43 (s, 3H, H6), 1.94 (m, 9H, H11', H9', H7').

^{13}C NMR (101 MHz, $CDCl_3$) δ 196.4 (C5), 169.7 (C8'), 169.2 (C10'), 169.0 (C6'), 166.7 (C12'), 159.9 (C1), 132.2 (C4), 130.3 (C3), 116.1 (C2), 97.7 (C1'), 72.2 (C5'), 71.5 (C3'), 70.7 (C2'), 68.8 (C4'), 52.7 (C13'), 26.2 (C6), 20.3 (C7'), 20.3 (C9'), 20.2 (C11').

HRMS (ESI$^+$): *m/z* calcd. for $[C_{21}H_{24}O_{11}+Na]^+$ calcd. 475.1216 found 475.1219.

Aceto-β-glucuronic acid methyl ester *para*-hydroxybenzyl methyl alcohol[38]

(2S,3R,4S,5S,6S)-2-(4-(1-hydroxyethyl)phenoxy)-6-(methoxycarbonyl)tetrahydro-2H-pyran-3,4,5-triyl triacetate (**13i**)

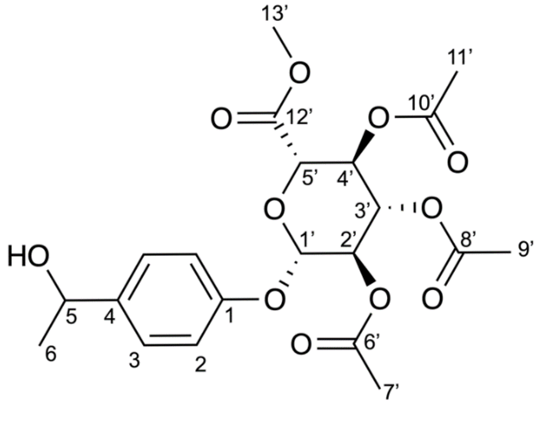

(2*S*,3*R*,4*S*,5*S*,6*S*)-2-(4-acetylphenoxy)-6-(methoxycarbonyl)tetrahydro-2*H*-pyran-3,4,5-triyl triacetate (**12i**) (0.1 g, 0.2 mmol, 1 eq) was dissolved in methanol (3 mL) and tetrahydrofuran (3 mL) and stirred at 0°C under N_2 for 15 min. $NaBH_4$ (0.017 g, 0.4 mmol, 2 eq) was added, and the reaction was stirred for 2 h. It was monitored by LC-MS until no starting material was detected. The reaction was quenched with acetone and sat. NH_4Cl (5 mL) and extracted with dichloromethane (3 × 20 mL). The organic layers were combined and washed

with brine (10 mL), dried (sodium sulfate), and the solvent removed *in vacuo* to give (2*S*,3*R*,4*S*,5*S*,6*S*)-2-(4-(1-hydroxyethyl) phenoxy)-6-(methoxycarbonyl) tetrahydro-2*H*-pyran-3,4,5-triyl triacetate (**13i**) as an off-white solid (0.104 g, 0.2 mmol, quantitative).

^{1}H NMR (400 MHz, $CDCl_3$, mixture of diastereomers 1:1) δ 7.21 (d, *J* = 8.3 Hz, 2H, H3), 6.89 (d, *J* = 8.6 Hz, 2H, H2), 5.32 – 5.14 (m, 3H, H2', H3', H4'), 5.08 (dd, *J* = 7.5, 3.0 Hz, 1H, H1'), 4.75 (qd, *J* = 6.4, 1.9 Hz, 1H, H5), 4.16 (d, *J* = 9.2 Hz, 1H, H5'), 3.64 (s, 3H, H13'), 2.70 (brs, 1H, OH), 1.99 – 1.92 (m, 9H, H11', H9', H7'), 1.35 (d, *J* = 6.5 Hz, 3H, H6).

^{13}C NMR (101 MHz, $CDCl_3$, mixture of diastereomers 1:1) δ 170.0 (C6'), 169.3 (C8'), 169.2 (C10'), 166.8 (C12'), 155.7 (C1), 141.2 (C4), 126.6 (C3), 126.6 (C3), 116.8 (C2), 116.8 (C2), 98.9 (C1'), 98.9 (C1'), 72.3 (H5'), 71.8 (C3'), 70.9 (C2'), 69.4 (C5), 69.3 (C5), 69.0 (C4'), 52.8 (C13'), 25.1 (C6), 20.4 (C11'), 20.4 (C9'), 20.3 (C7').

HRMS (ESI^+): *m/z* calcd. for $[C_{21}H_{26}O_{11}+Na]^+$ calcd. 477.1373 found 477.1363.

Aceto-β-glucuronic acid methyl ester *para*-hydroxybenzyl methyl bromide[37]

(2S,3R,4S,5S,6S)-2-(4-(1-bromoethyl)phenoxy)-6-(methoxycarbonyl)tetrahydro-2H-pyran-3,4,5-triyl triacetate (**14i**)

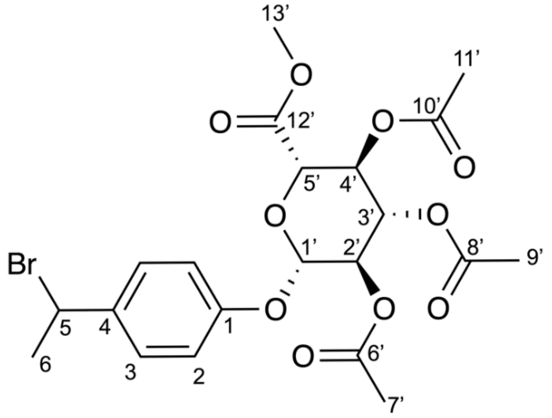

(2*S*,3*R*,4*S*,5*S*,6*S*)-2-(4-(1-hydroxyethyl)phenoxy)-6-(methoxycarbonyl)tetrahydro-2*H*-pyran-3,4,5-triyl triacetate (**13i**) (0.249 g, 0.55 mmol, 1 eq) was dissolved in anhydrous diethyl ether (10 mL) and anhydrous dichloromethane (2 mL) at 0°C under N_2. Neat PBr_3 (0.05 mL, 0.55 mmol, 1 eq) was added and the reaction was stirred under N_2 for 30 min. Thin-layer chromatography (1:1 Pet ether: ethyl acetate) showed that some starting material remained, so additional PBr_3 (0.1 mL, 1.1 mmol, 2 eq) was added and the reaction stirred for 15 min. Thin-

layer chromatography then showed completion. The reaction was quenched with cold water (10 mL), extracted into DCM (3 × 50 mL), washed with brine (10 mL), dried (sodium sulfate), and the solvent removed *in vacuo* to give (2*S*,3*R*,4*S*,5*S*,6*S*)-2-(4-(1-bromoethyl)phenoxy)-6-(methoxy carbonyl)tetrahydro-2*H*-pyran-3,4,5-triyl triacetate (**14i**) as an off-white solid (0.341 g, 0.660 mmol, quantitative).

^{1}H NMR (400 MHz, $CDCl_3$, mixture of diastereomers 1:1) δ 7.31 (d, *J* = 8.8 Hz, 2H. H3), 6.91 (d, *J* = 8.7 Hz, 2H, H2), 5.37 – 5.19 (m, 3H, H2', H3', H4'), 5.15 (m, 2H, H1', H5), 4.27 – 4.14 (m, 1H, H5'), 3.66 (s, 3H, H13'), 1.98 (s, 9H, H11', H9', H7'), 1.94 (d, *J* = 6.9 Hz, 3H, H6).

^{13}C NMR (101 MHz, $CDCl_3$, mixture of diastereomers 1:1) δ 169.8 (C6'), 169.2 (C8'), 169.0 (C10'), 166.8 (C12'), 156.2 (C4), 138.2 (C1), 128.1 (C3), 116.8 (C2), 98.6 (C1'), 72.3 (C5'), 71.7 (C3'), 70.9 (C2'), 69.0 (C4'), 52.8 (C13'), 49.0 (C5), 26.7 (C6), 26.7 (C6), 20.4 (C9', C11'), 20.3 (C7').

HRMS (ESI$^+$): *m/z* calcd. for $[C_{21}H_{25}O_{10}Br+Na]^+$ calcd. 539.0529 found 539.0541.

Aceto-β-glucuronic acid methyl ester *para*-hydroxybenzyl methyl β-lapa-ketol[19]

(2S,3R,4S,5S,6S)-2-(4-(1-(6-hydroxy-2,2-dimethyl-5-oxo-3,4,5,6-tetrahydro-2H-benzo[h]chromen-6-yl)ethyl)phenoxy)-6-(methoxycarbonyl)tetrahydro-2H-pyran-3,4,5-triyl triacetate (**15i**)

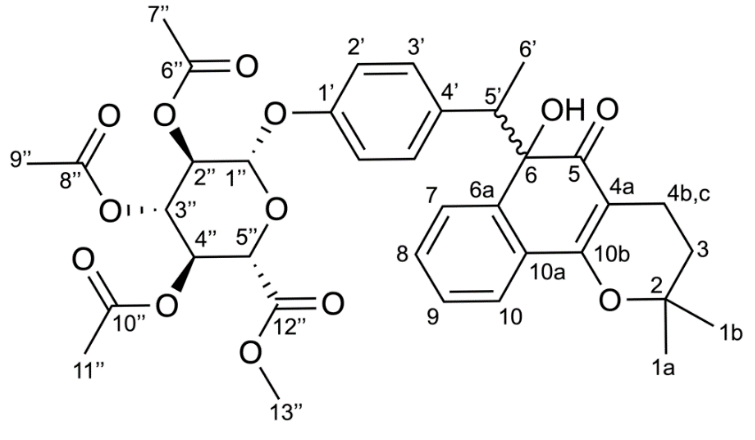

(2*S*,3*R*,4*S*,5*S*,6*S*)-2-(4-(1-bromoethyl)phenoxy)-6-(methoxycarbonyl)tetrahydro-2*H*-pyran-3,4,5-triyl triacetate (**14i**) (0.341 g, 0.66 mmol, 1.1 eq), NaI (0.257 g, 1.7 mmol, 2.8 eq), β-lapachone (**1**) (0.145 g, 0.6 mmol, 1 eq) and indium(0) powder (0.076 g, 0.66 mmol, 1.1 eq)

were added to anhydrous dimethylformamide (3 mL). The solution was heated to 40°C and sonicated overnight, while monitoring by thin-layer chromatography (50% ethyl acetate/Pet ether) and LC-MS. 1M HCl (~0.5 mL) was added to quench the reaction, and it was extracted with ethyl acetate (3 × 150 mL). The organic layers were combined and washed with brine (10 mL), dried (sodium sulfate), and the solvent removed *in vacuo*. The product was purified by flash column chromatography on silica gel 60 (40-60% ethyl acetate/Pet ether) to give two diastereomers of (2*S*,3*R*,4*S*,5*S*,6*S*)-2-(4-(1-(6-hydroxy-2,2-dimethyl-5-oxo-3,4,5,6-tetrahydro-2*H*-benzo[*h*]chromen-6-yl)ethyl)phenoxy)-6-(methoxycarbonyl)tetrahydro-2*H*-pyran-3,4,5-triyl triacetate (**15i**) as an off-white solid (0.0168 g, 0.024 mmol, 4%).**

**Yield would likely be higher if the other 2 diastereomers were isolated as well.

^{1}H NMR (400 MHz, MeOD, mixture of 2 diastereomers 1:1) δ 7.67 – 7.61 (m, 2H, H7, H10), 7.47 (td, *J* = 7.5, 1.5 Hz, 1H, H8), 7.44 – 7.36 (m, 1H, H9), 6.66 (dd, *J* = 8.9, 1.8 Hz, 2H, H2'), 6.37 (dd, *J* = 8.7, 2.3 Hz, 2H, H3'), 5.41 (td, *J* = 9.5, 2.3 Hz, 1H, H3"), 5.32 (dd, *J* = 11.3, 7.9 Hz, 1H, H1"), 5.20 – 5.09 (m, 2H, H2", H4"), 4.46 (dd, *J* = 9.9, 2.5 Hz, 1H, H5"), 3.71 (d, *J* = 9.7 Hz, 3H, H13"), 3.10 (qd, *J* = 7.0, 2.3 Hz, 1H, H5'), 2.34 (dq, *J* = 16.8, 5.6 Hz, 1H, H4b,c), 2.06 – 1.98 (m, 9H, H7", H9", H11"), 1.92 (dddd, *J* = 17.1, 8.6, 6.4, 2.0 Hz, 1H, H4b,c), 1.61 – 1.41 (m, 2H, H3), 1.33 – 1.26 (m, 6H, H6', H1a/H1b), 0.96 (d, *J* = 11.5 Hz, 3H, H1a/H1b).

^{13}C NMR (101 MHz, MeOD, mixture of 2 diastereomers 1:1) δ 202.2 (C5), 202.1 (C5), 170.0 (C6"), 170.0 (C6"), 169.7 (C10"), 169.5 (C8"), 169.5 (C8"), 167.5 (C12"), 167.5 (C12"), 161.9 (C10b), 161.9 (C10b), 155.6 (C1'), 155.5 (C1'), 139.0 (C6a), 139.0 (C6a), 135.8 (C4'), 135.6 (C4'), 128.8 (C10a), 128.7 (C10a), 128.4 (C3', C8), 128.3 (C3', C8), 127.3 (C7/C10), 127.3 (C7/C10), 127.3 (C9), 127.2 (C9), 122.4 (C7/C10), 115.2 (C2'), 115.1 (C2'), 107.3 (C4a), 107.3 (C4a), 97.7 (C1"), 97.5 (C1"), 79.6 (C6), 77.6 (C2), 77.6 (C2), 71.9 (C3"), 71.8 (C3"), 71.5 (C5"), 71.5 (C5"), 71.0 (C2"), 69.3 (C4"), 52.6 (C5'), 52.6 (C5'), 51.9 (C13"), 51.9 (C13"), 31.0 (C3), 30.9 (C3), 26.2 (C1a/C1b), 26.0 (C1a/C1b), 25.0 (C1a/C1b), 24.8 (C1a/C1b), 19.1 (C7"/C9"/C11"), 19.1 (C7"/C9"/C11"), 19.0 (C7"/C9"/C11"), 19.0 (C7"/C9"/C11"), 19.0 (C7"/C9"/C11"), 15.0 (C4b,c), 14.6 (C6'), 14.5 (C6').

HRMS (ESI^{+}): *m/z* calcd. for $[C_{36}H_{40}O_{13}+Na]^{+}$ calcd. 703.2367 found 703.2363.

β-glucuronide-*para*-hydroxybenzyl methyl β-lapa-ketol[26]

(2S,3S,4S,5R,6S)-3,4,5-trihydroxy-6-(4-(1-(6-hydroxy-2,2-dimethyl-5-oxo-3,4,5,6-tetrahydro-2H-benzo[h]chromen-6-yl)ethyl)phenoxy)tetrahydro-2H-pyran-2-carboxylic acid (**16i**)

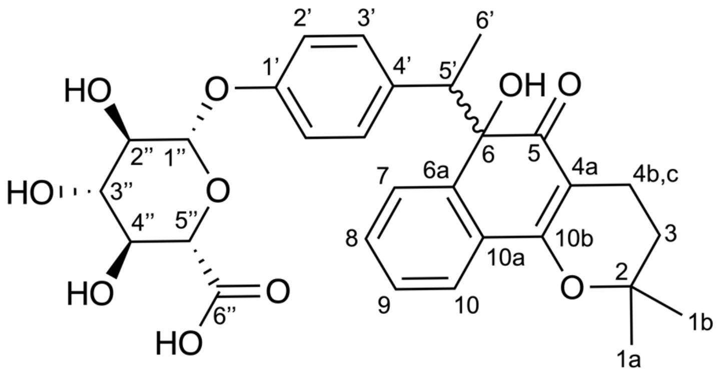

The acetyl-protected glucuronide β-lapachone prodrug (**15i**) (0.168 g, 0.025 mmol, 1 eq) was dissolved in tetrahydrofuran (2 mL) and methanol (2 mL) and stirred at 0°C. A separate solution of LiOH monohydrate (0.0063 g, 0.15 mmol, 6 eq) in water (1 mL) was prepared and added to the solution of **15i** dropwise. The reaction was stirred for 1.5 h and product formation was monitored by LC-MS. After complete conversion, glacial acetic acid (8.4 µL, 0.15 mmol, 6 eq) was added and the solvent removed *in vacuo*. Crude product **16i** was purified by semi-preparative HPLC to give two diastereomers of (2*S*,3*S*,4*S*,5*R*,6*S*)-3,4,5-trihydroxy-6-(4-(1-(6-hydroxy-2,2-dimethyl-5-oxo-3,4,5,6-tetrahydro-2*H*-benzo[*h*]chromen-6-yl)ethyl)phenoxy) tetrahydro-2*H*-pyran-2-carboxylic acid (**16i**) as an orange solid after lyophilization (0.0041 g, 0.0076 mmol, 31%). HPLC Purification Method: Column = YMC Pack Pro C18 5 µm 250×10 mm 120Å; mobile phases: A = H_2O + 0.1 % formic acid, B = ACN + 0.1 % formic acid; gradient: t = 0-1 min 0% B, t = 1-10 min 0-100% B, t = 10-12 min 100% B. Retention time of **16i** = 8.5 min.

^{1}H NMR (400 MHz, MeOD, mixture of 2 diastereomers 1:1) δ 7.64 (dtd, *J* = 7.4, 5.3, 1.4 Hz, 2H, H7, H10), 7.46 (tt, *J* = 7.5, 2.0 Hz, 1H, H8), 7.39 (tt, *J* = 7.5, 1.3 Hz, 1H, H9), 6.73 (t, *J* = 8.9 Hz, 2H, H2'), 6.36 (dd, *J* = 8.4, 5.4 Hz, 2H, H3'), 4.81 (dd, *J* = 7.6, 3.7 Hz, 1H, H1"), 3.88 (dd, *J* = 9.7, 2.6 Hz, 1H, H5"), 3.66 – 3.52 (m, 1H, H4"), 3.49 – 3.39 (m, 2H, H3", H2"), 3.10 (qd, *J* = 7.1, 2.7 Hz, 1H, H5'), 2.33 (ddt, *J* = 16.7, 10.7, 5.9 Hz, 1H, H4b,c), 2.06 – 1.87 (m, 1H, H4b,c), 1.62 – 1.42 (m, 2H, H3), 1.36 – 1.22 (m, 6H, H6', H1a/H1b), 1.00 (d, *J* = 3.8 Hz, 3H, H1a/H1b).

^{13}C NMR (101 MHz, MeOD, mixture of 2 diastereomers 1:1) δ 202.3 (C5), 202.2 (C5), 171.5 (C6"), 162.0 (C10b), 156.5 (C1'), 156.3 (C1'), 139.1 (C6a), 139.0 (C6a), 135.1 (C4'), 134.8 (C4'), 128.8 (C8), 128.8 (C8), 128.3 (C10a), 128.1 (C3'), 128.1 (C3'), 127.3 (C10/C7), 127.2 (C9), 122.4 (C10/C7), 115.5 (C2'), 115.2 (C2'), 107.3 (C4a), 107.3 (C4a), 100.9 (C1"), 100.4 (C1"), 79.7 (C6), 77.7 (C2), 77.7 (C2), 75.9 (C2"/C3"), 75.1 (C5"), 73.0 (C2"/C3"), 71.6 (C4"),

52.7 (C5'), 52.6 (C5'), 31.0 (C3), 30.9 (C3), 26.1(C1a/C1b), 25.9 (C1a/C1b), 25.2 (C1a/C1b), 24.9 (C1a/C1b), 15.1 (C4b,c), 14.7 (C6'), 14.4 (C6').

HRMS (ESI$^+$): *m/z* calcd. for $[C_{29}H_{32}O_{10}+Na]^+$ calcd. 563.1888 found 563.1885.

11.13. Synthetic Procedures for 16j

Aceto-β-glucuronic acid methyl ester 2-hydroxy-*para*-hydroxybenzaldehyde[24,39,40]

(2S,3R,4S,5S,6S)-2-(4-formyl-3-hydroxyphenoxy)-6-(methoxycarbonyl)tetrahydro-2H-pyran-3,4,5-triyl triacetate (**12j**)

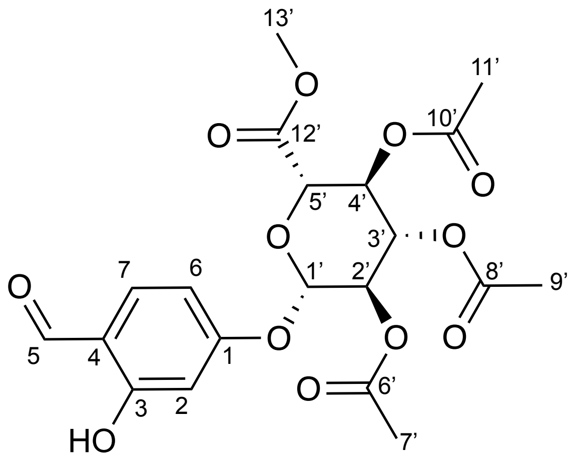

Acetobromo-α-D-glucuronic acid methyl ester (**S2**) (0.547 g, 1.37 mmol, 1 eq) and 2,4-dihydroxybenzaldehyde (**11j**) (0.274 g, 1.98 mmol, 1.4 eq) were combined in anhydrous acetonitrile (5 mL) under N_2 in the dark and stirred for 30 min. Ag_2O (0.787 g, 3.4 mmol, 2.5 eq) was added and the reaction was stirred at room temperature for 48 h in the dark. Thin-layer chromatography monitored reaction progress (50% ethyl acetate/Pet ether). The reaction was then filtered through celite, and the solvent was removed *in vacuo*. The residue was dissolved in ethyl acetate, washed with sat. Na_2CO_3 (10 mL), water (10 mL), and brine (10 mL). It was then dried (sodium sulfate), and the solvent was removed *in vacuo*. The product was purified by flash column chromatography on silica gel 60 (30-100% ethyl acetate/Pet ether) to give (2*S*,3*R*,4*S*,5*S*,6*S*)-2-(4-formyl-3-hydroxyphenoxy)-6-(methoxycarbonyl)tetrahydro-2*H*-pyran-3,4,5-triyl triacetate (**12j**) as an off-white sticky solid (0.241 g, 0.53 mmol, 41%)*

*This product contained trace sugar starting material that proved impossible to remove (possibly due to hydrogen bonding with the product) and thus was used going forward with these impurities present. The presence of the phenol O<u>H</u> peak at 11.27 ppm in the ^{1}H NMR spectrum confirmed the glycosylation had occurred at the *para* position, as this high chemical shift is indicative of an H experiencing hydrogen bonding with the aldehyde. NOESY was also used to confirm this structure.

^{1}H NMR (400 MHz, $CDCl_3$) δ 11.27 (s, 1H, OH), 9.69 (s, 1H, H5), 7.43 (d, J = 8.6 Hz, 1H, H7), 6.55 (dd, J = 8.6, 2.3 Hz, 1H, H6), 6.48 (d, J = 2.3 Hz, 1H, H2), 5.35 – 5.21 (m, 4H, H1', H2', H3', H4'), 4.26 (d, J = 9.2 Hz, 1H, H5'), 3.66 (s, 3H, H13'), 2.01 – 1.94 (m, 9H, H7', H9', H11').

^{13}C NMR (101 MHz, $CDCl_3$) δ 194.9 (C5), 170.0 (C8'), 169.3 (C10'), 169.1 (C6'), 166.7 (C12'), 163.8 (C3), 163.0 (C1), 135.5 (C7), 116.7 (C4), 109.3 (C6), 103.7 (C2), 97.4 (C1'), 72.5 (C5'), 71.5 (C3'), 70.7 (C2'), 68.8 (C4'), 53.0 (C13'), 20.5 (C7'), 20.5 (C9'), 20.4 (C11').

HRMS (ESI^-): *m/z* calcd. for $[C_{20}H_{22}O_{12}-H]^-$ calcd. 453.1038 found 453.1038.

Aceto-β-glucuronic acid methyl ester 2-methoxyethoxy-*para*-hydroxybenzaldehyde[41]

(2S,3R,4S,5S,6S)-2-(4-formyl-3-(2-methoxyethoxy)phenoxy)-6-(methoxycarbonyl)tetrahydro-2H-pyran-3,4,5-triyl triacetate (**17**)

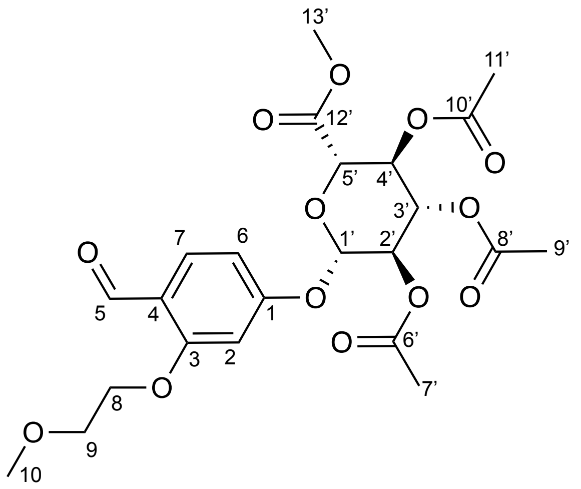

Ag$_2$O (0.274 g, 1.18 mmol, 2.2 eq) and anhydrous potassium carbonate (0.28 g, 2.02 mmol, 3.8 eq) were suspended in anhydrous dimethylformamide (3 mL) and dried over molecular sieves under a nitrogen atmosphere. Compound **12j** (0.2408 g, 0.53 mmol, 1 eq) was dissolved separately in anhydrous DCM (4 mL) and added in four portions to the silver suspension. 2-Bromoethyl methyl ether (0.05 mL, 5.3 mmol, 10 eq) was then added dropwise to the suspension. The mixture was heated to 55 ºC and stirred in the dark overnight until LC-MS indicated reaction completion. The reaction was then filtered through celite, and the solvent was removed *in vacuo*. The residue was dissolved in DCM, washed with sat. Na_2CO_3 (10 mL), water

(10 mL), and brine (10 mL). It was then dried (sodium sulfate), and the solvent was removed *in vacuo*. The product was purified by flash column chromatography on silica gel 60 (30-100% ethyl acetate/Pet ether) to give (2*S*,3*R*,4*S*,5*S*,6*S*)-2-(4-formyl-3-(2-methoxyethoxy) phenoxy)-6-(methoxycarbonyl)tetrahydro-2*H*-pyran-3,4,5-triyl triacetate (**17**) as an off-white sticky solid (0.1089 g, 0.21 mmol, 40%).

^{1}H NMR (400 MHz, $CDCl_3$) δ 10.32 (s, 1H, H5), 7.76 (d, *J* = 8.4 Hz, 1H, H7), 6.62 – 6.55 (m, 2H, H2, H6), 5.38 – 5.20 (m, 4H, H1', H2', H3', H4'), 4.26 – 4.20 (m, 1H, H5'), 4.15 (td, *J* = 4.3, 2.0 Hz, 2H, H8), 3.76 (dt, *J* = 6.2, 2.3 Hz, 2H, H9), 3.68 (s, 3H, H13'), 3.41 (s, 3H, H10), 2.05 – 1.97 (m, 9H, H7', H9', H11').

^{13}C NMR (101 MHz, $CDCl_3$) δ 188.3 (C5), 170.0 (C8'), 169.3 (C10'), 169.2 (C6'), 166.7 (C12'), 162.7 (C3), 162.3 (C1), 130.2 (C7), 121.0 (C4), 108.2 (C6), 101.9 (C2), 97.9 (C1'), 72.6 (C5'), 71.6 (C3'), 70.9 (C2'), 70.7 (C9), 68.8 (C4'), 68.4 (C8), 59.3 (C10), 53.0 (C13'), 20.6 (C7'), 20.6 (C9'), 20.5 (C11').

HRMS (ESI$^+$): *m/z* calcd. for $[C_{23}H_{28}O_{13}+H]^+$ calcd. 513.1603 found 513.1608.

Aceto-β-glucuronic acid methyl ester 2-methoxyethoxy-*para*-hydroxybenzyl alcohol[24,40]

(2S,3R,4S,5S,6S)-2-(4-(hydroxymethyl)-3-(2-methoxyethoxy)phenoxy)-6-(methoxycarbonyl)tetrahydro-2H-pyran-3,4,5-triyl triacetate (**13j**)

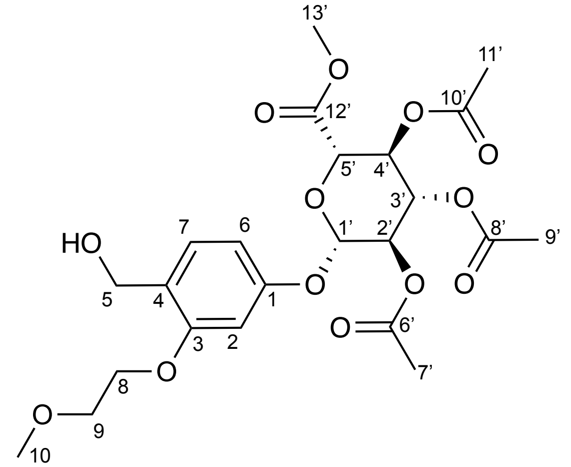

Compound **17** (0.084 g, 0.165 mmol, 1 eq) was dissolved in chloroform (10 mL) and isopropanol (2 mL) with silica gel (0.2 g) and stirred at 0°C under N_2 for 15 min. $NaBH_4$ (0.016

g, 0.42, 2.5 eq) was added, and the reaction was stirred for 30 min. It was monitored by thin-layer chromatography (1:1 ethyl acetate: Pet ether) and LC-MS. When the reaction was not complete, an additional portion of $NaBH_4$ (0.008 g, 0.21, 1.2 eq) was then added and stirred for 15 min until consumption of the starting material was complete. The reaction was then quenched with acetone, diluted with dichloromethane (10 mL), filtered over celite, and washed with dichloromethane (10 mL). The filtrate was washed with brine (10 mL), dried (sodium sulfate), and the solvent removed *in vacuo.* This gave (2*S*,3*R*,4*S*,5*S*,6*S*)-2-(4-(hydroxymethyl)-3-(2-methoxyethoxy)phenoxy)-6-(methoxycarbonyl) tetrahydro-2*H*-pyran-3,4,5-triyl triacetate (**13j**) as a gooey yellow oil (0.085 g, 0.165 mmol, quantitative).

^{1}H NMR (400 MHz, $CDCl_3$) δ 7.13 (d, *J* = 7.9 Hz, 1H, H7), 6.57 – 6.49 (m, 2H, H2, H6), 5.35 – 5.18 (m, 3H, H2', H3', H4'), 5.12 (d, *J* = 7.4 Hz, 1H, H1'), 4.56 (s, 2H, H5), 4.18 – 4.13 (m, 1H, H5'), 4.09 (d, *J* = 4.7 Hz, 2H, H8), 3.79 – 3.64 (m, 5H, H9, H13'), 3.38 (s, 3H, H10), 3.18 (brs, 1H, O<u>H</u>), 2.05 – 1.98 (m, 9H, H7', H9', H11').

^{13}C NMR (101 MHz, $CDCl_3$) δ 170.1 (C8'), 169.3 (C10'), 169.3 (C6'), 166.8 (C12'), 157.8 (C3), 157.2 (C1), 129.7 (C7), 125.8 (C4), 108.2 (C6), 103.2 (C2), 99.1 (C1'), 72.5 (C5'), 71.9 (C3'), 71.1 (C2'), 70.7 (C9), 69.1 (C4'), 68.0 (C8), 61.4 (C5), 59.0 (C10), 52.9 (C13'), 20.6 (C7'), 20.6 (C9'), 20.5 (C11').

HRMS (ESI$^+$): *m/z* calcd. for $[C_{23}H_{30}O_{13}]^+$ calcd. 514.1681 found 514.1684.

Aceto-β-glucuronic acid methyl ester 2-methoxyethoxy-*para*-hydroxybenzyl bromide[28]

(2S,3R,4S,5S,6S)-2-(4-(bromomethyl)-3-(2-methoxyethoxy)phenoxy)-6-(methoxycarbonyl)tetrahydro-2H-pyran-3,4,5-triyl triacetate (**14j**)

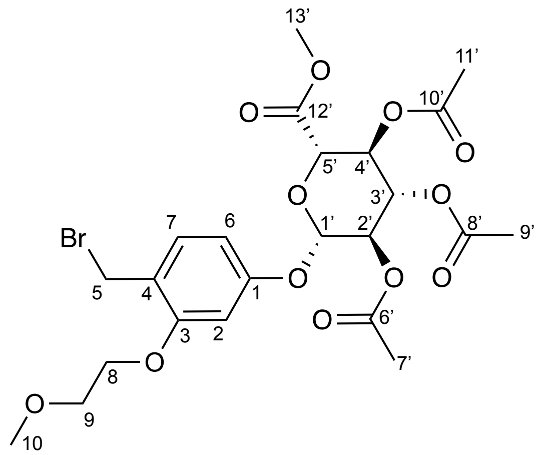

Compound **13j** (0.085 g, 0.165 mmol, 1 eq) was dissolved in anhydrous dichloromethane (6 mL) and anhydrous THF (6 mL) at 0°C under N_2. Neat PBr_3 (0.04 mL, 0.42 mmol, 2.5 eq) was added and the reaction was stirred under N_2 for 30 min. Thin-layer chromatography (50% ethyl acetate/Pet ether) then showed reaction completion. The reaction was quenched with cold sat. $NaHCO_3$ (10 mL), extracted into DCM (3 × 50 mL) washed with brine (10 mL), dried (sodium sulfate), and the solvent removed *in vacuo* to give (2*S*,3*R*,4*S*,5*S*,6*S*)-2-(4-(bromomethyl)-3-(2-methoxyethoxy)phenoxy)-6-(methoxycarbonyl) tetrahydro-2*H*-pyran-3,4,5-triyl triacetate (**14j**) as an unstable off-white solid (0.095 g, 0.165 mmol, quantitative). Due to the instability of the compound, it was used immediately in the next reaction without further purification or characterization.

Aceto-β-glucuronic acid methyl ester 2-methoxyethoxy-*para*-hydroxybenzyl β-lapa-ketol[19]

(2S,3R,4S,5S,6S)-2-(4-((6-hydroxy-2,2-dimethyl-5-oxo-3,4,5,6-tetrahydro-2H-benzo[h]chromen-6-yl)methyl)-3-(2-methoxyethoxy)phenoxy)-6-(methoxycarbonyl)tetrahydro-2H-pyran-3,4,5-triyl triacetate (**15j**)

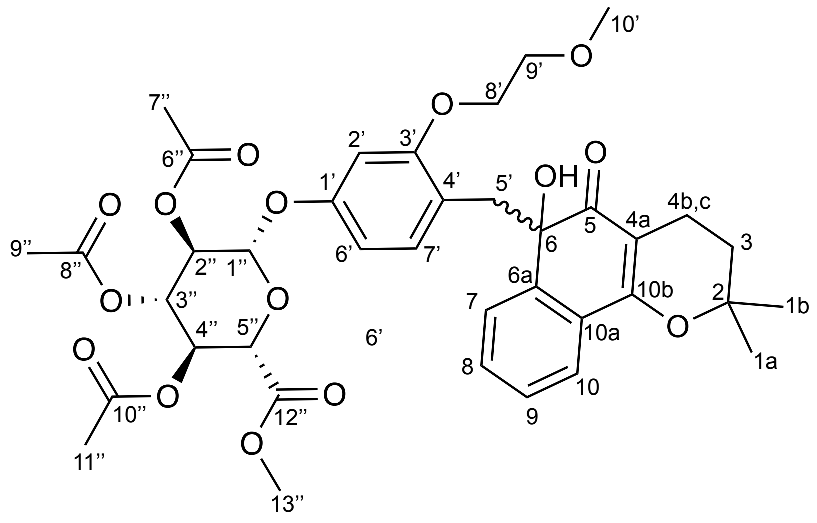

Compound **14j** (0.095 g, 0.165 mmol, 1 eq), NaI (0.113 g, 0.75 mmol, 4.5 eq), β-lapachone (**1**) (0.055 g, 0.22 mmol, 1.3 eq) and indium(0) powder (0.043 g, 0.178 mmol, 1.08 eq) were added to anhydrous dimethylformamide (3 mL). The solution was sonicated overnight, while monitoring by thin-layer chromatography (50% ethyl acetate/Pet ether) and LC-MS. 1M HCl (~0.5 mL) was added to quench the reaction, and it was extracted with ethyl acetate (3 × 150 mL). The organic layers were combined and washed with brine (10 mL), dried (sodium sulfate), and the solvent removed *in vacuo*. The product was purified by flash column chromatography on silica gel 60 (20-80% ethyl acetate/Pet ether) to give (2*S*,3*R*,4*S*,5*S*,6*S*)-2-(4-((6-hydroxy-2,2-dimethyl-5-oxo-3,4,5,6-tetrahydro-2*H*-benzo[*h*]chromen-6-yl)methyl)-3-(2-methoxyethoxy)phenoxy)-6-(methoxycarbonyl) tetrahydro-2*H*-pyran-3,4,5-triyl triacetate (**15j**) as an orange colored sticky solid (0.0627 g, 0.085 mmol, 37%).

^{1}H NMR (400 MHz, MeOD, mixture of diastereomers 1:1) δ 7.61 (ddd, *J* = 9.3, 7.7, 1.4 Hz, 1H, H7), 7.46 (ddd, *J* = 17.8, 7.7, 1.4 Hz, 1H, H10), 7.37 (tdd, *J* = 7.7, 4.3, 1.4 Hz, 1H, H8), 7.30 (td, *J* = 7.6, 1.3 Hz, 1H, H9), 6.44 – 6.26 (m, 3H, H2', H6', H7'), 5.42 (td, *J* = 9.5, 1.4 Hz, 1H, H3"), 5.32 (t, *J* = 8.1 Hz, 1H, H1"), 5.25 – 5.10 (m, 2H, H2", H4"), 4.46 (dd, *J* = 9.9, 1.8 Hz, 1H, H5"), 3.77 – 3.63 (m, 5H, H8', H13"), 3.60 – 3.50 (m, 2H, H9'), 3.39 (d, *J* = 2.2 Hz, 3H, H10'), 3.25 (dd, *J* = 12.5, 10.9 Hz, 1H, H5'), 2.90 (dd, *J* = 12.5, 8.3 Hz, 1H, H5'), 2.49 (dtd, *J* = 17.2, 5.6, 2.6 Hz, 1H, H4b,c), 2.16 (dddd, *J* = 17.4, 9.3, 6.9, 3.4 Hz, 1H, H4b,c), 2.05 – 1.98 (m, 9H, H7", H9", H11"), 1.76 – 1.58 (m, 2H, H3), 1.37 (d, *J* = 4.6 Hz, 3H, H1a/H1b), 1.14 (d, *J* = 8.9 Hz, 3H, H1a/H1b).

^{13}C NMR (101 MHz, MeOD, mixture of diastereomers 1:1) δ 202.6 (C5), 202.6 (C5), 171.4 (C8"), 171.1 (C6"), 170.9 (C10"), 170.9 (C10"), 168.9 (C12"), 168.8 (C12"), 163.0 (C10b), 163.0 (C10b), 159.1 (C3'), 159.0 (C3'), 158.1 (C1'), 158.0 (C1'), 143.6 (C6a), 133.1 (C7'), 133.0 (C7'), 130.5 (C8), 129.1 (C10a), 129.0 (C10a), 128.2 (C9), 127.2 (C7), 127.2 (C7), 123.6

(C10), 123.6 (C10), 119.9 (C4’), 119.7 (C4’), 108.5 (C4a), 108.4 (C4a), 108.3 (C6’), 108.0 (C6’), 101.9 (C2’), 101.8 (C2’), 99.4 (C1”), 99.2 (C1”), 79.3 (C6), 79.2 (C6), 79.0 (C2), 78.9 (C2), 73.3 (C3”), 73.3 (C3”), 73.0 (C5”), 72.9 (C5”), 72.4 (C2”), 72.4 (C2”), 71.8 (C9’), 70.6 (C4”), 68.5 (C8’), 59.4 (C10’), 53.3 (C13”), 53.3 (C13”), 46.2 (C5’), 46.0 (C5’), 32.6 (C3), 27.7 (C1a/C1b), 26.1 (C1a/C1b), 26.1 (C1a/C1b), 20.6 (C7”/C9”/C11”), 20.6 (C7”/C9”/C11”), 20.5 (C7”/C9”/C11”), 20.4 (C7”/C9”/C11”), 16.9 (C4b,c).

HRMS (ESI$^+$): *m/z* calcd. for $[C_{38}H_{44}O_{15}+H]^+$ calcd. 741.2753 found 741.2760.

β-glucuronide-2-methoxyethoxy-*para*-hydroxybenzyl β-lapa-ketol[26]

(2S,3S,4S,5R,6S)-3,4,5-trihydroxy-6-(4-((6-hydroxy-2,2-dimethyl-5-oxo-3,4,5,6-tetrahydro-2H-benzo[h]chromen-6-yl)methyl)-3-(2-methoxyethoxy)phenoxy)tetrahydro-2H-pyran-2-carboxylic acid (**16j**)

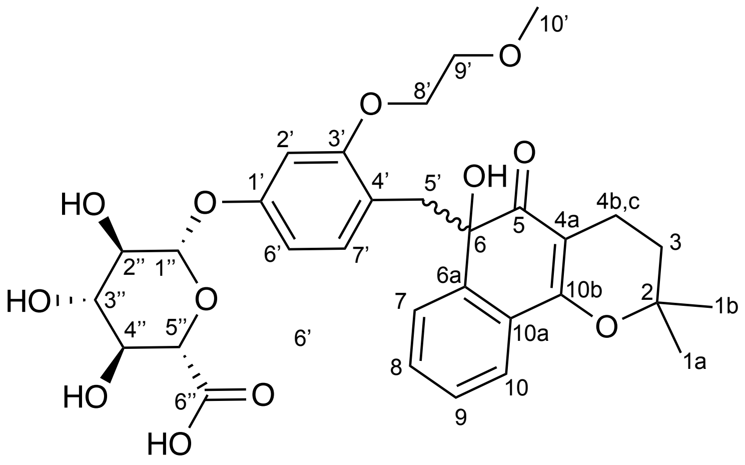

The acetyl-protected glucuronide β-lapachone prodrug (**15j**) (0.0627 g, 0.085 mmol, 1 eq) was dissolved in tetrahydrofuran (2 mL) and methanol (2 mL) and stirred at 0°C. A separate solution of LiOH monohydrate (0.0213 g, 0.5 mmol, 6 eq) in water (1 mL) was prepared and added to the solution of **15j** dropwise. The reaction was stirred for 1.5 h and product formation was monitored by LC-MS. After complete conversion, glacial acetic acid (14.5 µL, 0.5 mmol, 6 eq) was added and the solvent removed *in vacuo*. Crude product **16j** was purified by semi-preparative HPLC to give (2*S*,3*S*,4*S*,5*R*,6*S*)-3,4,5-trihydroxy-6-(4-((6-hydroxy-2,2-dimethyl-5-oxo-3,4,5,6-tetrahydro-2*H*-benzo[*h*]chromen-6-yl)methyl)-3-(2-methoxyethoxy)phenoxy) tetrahydro-2*H*-pyran-2-carboxylic acid (**16j**) as an off-white solid after lyophilization (0.041 g, 0.068 mmol, 80%). HPLC Purification Method: Column = Agilent InfinityLab ZORBAX 5 Eclipse Plus C18 21.2 × 250 mm; mobile phases: A = H_2O, B = ACN; gradient: t = 0-5 min 5% B, t = 5-35 min 5-35% B, t = 35-40 min 35-95% B. Retention time of **16j** = 8.4 min.

^{1}H NMR (400 MHz, MeOD, mixture of diastereomers 1:1) δ 7.61 (dt, J = 7.7, 1.8 Hz, 1H, H7), 7.44 (dd, J = 7.7, 1.5 Hz, 1H, H10), 7.36 (tdd, J = 7.6, 2.7, 1.5 Hz, 1H, H8), 7.29 (tdd, J = 7.4, 2.5, 1.4 Hz, 1H, H9), 6.55 (dd, J = 8.6, 2.3 Hz, 1H, H2'), 6.45 (ddd, J = 9.5, 8.3, 2.3 Hz, 1H, H6'), 6.38 (dd, J = 12.8, 8.3 Hz, 1H, H7'), 4.76 (dd, J = 16.7, 7.3 Hz, 1H, H1"), 3.81 – 3.69 (m, 2H, H8'), 3.67 (d, J = 9.6 Hz, 1H, H5"), 3.56 (ddt, J = 6.5, 4.3, 2.1 Hz, 2H, H9'), 3.54 – 3.43 (m, 3H, H2", H3", H4"), 3.40 (s, 3H, H10'), 3.25 (dd, J = 12.6, 6.4 Hz, 1H, H5'), 2.92 (dd, J = 12.6, 10.2 Hz, 1H, H5'), 2.49 (dt, J = 17.2, 5.7 Hz, 1H, H4b,c), 2.27 – 2.13 (m, 1H, H4b,c), 1.80 – 1.60 (m, 2H, H3), 1.38 (d, J = 3.1 Hz, 3H, H1a/H1b), 1.20 (s, 3H, H1a/H1b).

^{13}C NMR (101 MHz, MeOD, mixture of diastereomers 1:1) δ 202.9 (C5), 176.3 (C6"), 176.3 (C6"), 163.1 (C10b), 159.6 (C3'), 159.5 (C3'), 158.9 (C1'), 143.6 (C6a), 132.9 (C7'), 130.5 (C8), 129.1 (C10a), 129.0 (C10a), 128.2 (C9), 127.2 (C7), 123.6 (C10), 119.0 (C4'), 118.8 (C4'), 109.3 (C6'), 108.9 (C6'), 108.5 (C4a), 108.5 (C4a), 103.2 (C1"), 102.8 (C1"), 102.4 (C2'), 102.3 (C2'), 79.4 (C6), 79.3 (C6), 79.1 (C2), 79.0 (C2), 77.7 (C3"), 76.7 (C5"), 74.7 (C2"), 73.6 (C4"), 71.9 (C9'), 68.3 (C8'), 59.3 (C10'), 59.3 (C10'), 46.1 (C5'), 32.6 (C3), 27.6 (C1a/C1b), 26.2 (C1a/C1b), 26.2 (C1a/C1b), 16.9 (C4b,c).

HRMS (ESI^{+}): m/z calcd. for $[C_{31}H_{36}O_{12}+H]^{+}$ calcd. 601.2280 found 601.2306.

11.14. Synthetic Procedures for 22

2-phenyl-acetyl-2-methoxy-*para*-amino-methyl benzoate[42]

methyl 2-methoxy-4-(2-phenylacetamido)benzoate (**19**)

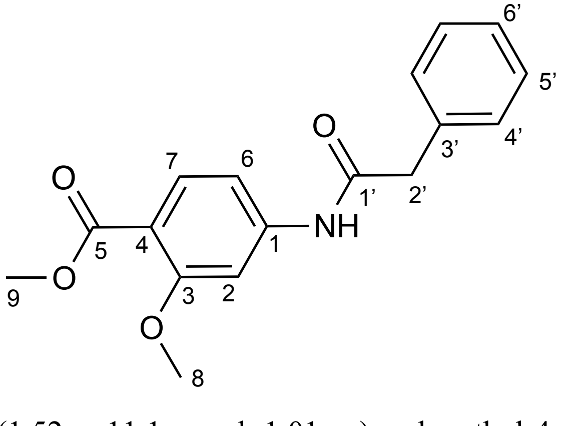

Phenyl acetic acid (1.52 g, 11.1 mmol, 1.01 eq) and methyl-4-amino-2-methoxybenzoate (**18**) (1.99 g, 11 mmol, 1 eq) were suspended in anhydrous DCM (20 mL) under a nitrogen atmosphere. EEDQ (3 g, 12.1 mmol, 1.1 eq) was then added in portions and the reaction was stirred overnight at room temperature. The next day, the peach-colored clear solution was

washed with 1M HCl (3 × 100 mL) and back extracted with DCM (3 × 50 mL). The organic layers were combined and washed with brine (10 mL), dried (sodium sulfate), and the solvent removed *in vacuo*. The product was purified by flash column chromatography on silica gel 60 (40% ethyl acetate/Pet ether) to give methyl 2-methoxy-4-(2-phenylacetamido)benzoate (**19**) as an off-white solid (1.016 g, 3.4 mmol, 31%).

^{1}H NMR (400 MHz, $CDCl_3$) δ 9.14 (s, 1H, NH), 7.79 (d, *J* = 8.5 Hz, 1H, H7), 7.64 (d, *J* = 1.9 Hz, 1H, H2), 7.33 – 7.20 (m, 5H, H4', H5', H6'), 7.00 (dd, *J* = 8.6, 1.9 Hz, 1H, H6), 3.87 (s, 3H, H9), 3.67 (s, 5H, H8, H2').

^{13}C NMR (101 MHz, $CDCl_3$) δ 170.4 (C1'), 166.2 (C5), 160.3 (C3), 143.7 (C1), 134.3 (C3'), 132.6 (C7), 129.0 (C4'), 128.6 (C5'), 127.1 (C6'), 114.0 (C4), 110.6 (C6), 102.9 (C2), 55.4 (C8), 51.8 (C9), 44.2 (C2').

HRMS (ESI$^+$): *m/z* calcd. for $[C_{17}H_{17}NO_4+H]^+$ calcd. 300.1230 found 300.1228.

2-phenyl-acetyl-2-methoxy-*para*-aminobenzyl alcohol[43]

N-(4-(hydroxymethyl)-3-methoxyphenyl)-2-phenylacetamide (**20**)

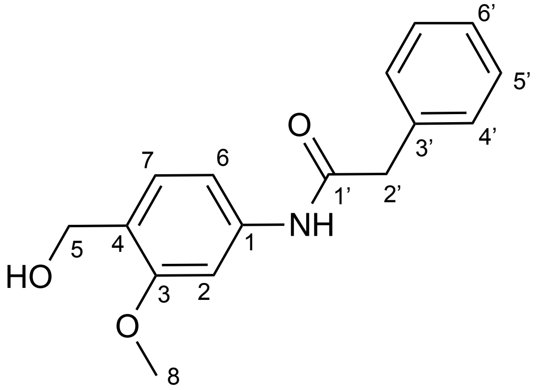

Compound **19** (1 g, 3.4 mmol, 1 eq) was dissolved in dry THF (9 mL) and dry MeOH (1 mL) under a nitrogen atmosphere. The solution was cooled to 0 ºC. $LiBH_4$ (2M in THF, 2.5 mL, 1.5 eq) was added dropwise, and the solution was allowed to warm to room temperature. It was stirred at room temperature for 14 h. LC-MS indicated starting material was remaining, so an additional portion of $LiBH_4$ (2M in THF, 7.5 mL, 4.4 eq) was added at 0 ºC. The reaction was then heated to 50 ºC and left to stir for an additional 24 h. Once LC-MS indicated reaction completion, the solution was cooled to 0 ºC and slowly quenched with HCl (3M, 10 mL). The

product was then extracted with DCM (3 × 50 mL), and the organic layers were combined, washed with brine (10 mL), and dried (sodium sulfate). The solvent was removed *in vacuo*. The product was purified by flash column chromatography on silica gel 60 (60% ethyl acetate/Pet ether) to give *N*-(4-(hydroxymethyl)-3-methoxyphenyl)-2-phenylacetamide (**20**) as an off-white solid (0.6452 g, 2.38 mmol, 70%).

^{1}H NMR (400 MHz, MeOD) δ 7.37 – 7.19 (m, 7H, H4', H5', H6', H2, H7), 7.02 (dd, *J* = 8.1, 2.0 Hz, 1H, H6), 4.57 (s, 2H, H5), 3.75 (s, 3H, H8), 3.64 (s, 2H, H2').

^{13}C NMR (101 MHz, MeOD) δ 171.9 (C1'), 158.2 (C3), 140.0 (C1), 136.3 (C3'), 129.8 (C4'), 129.3 (C5'), 129.2 (C7), 127.7 (C6'), 126.0 (C4), 112.4 (C6), 103.6 (C2), 60.0 (C5), 55.6 (C8), 44.5 (C2').

HRMS (ESI$^+$): *m/z* calcd. for $[C_{16}H_{17}NO_3+H]^+$ calcd. 272.1281 found 272.1285.

2-phenyl-acetyl-2-methoxy-*para*-aminobenzyl bromide[28]

N-(4-(bromomethyl)-3-methoxyphenyl)-2-phenylacetamide (**21**)

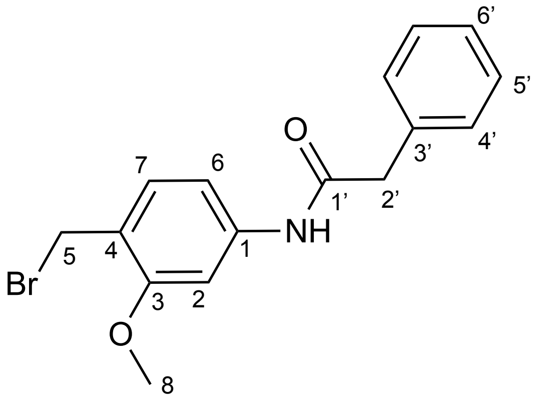

Compound **20** (0.2 g, 0.737 mmol, 1 eq) was dissolved in anhydrous dichloromethane (20 mL) at 0°C under N_2. Neat PBr_3 (0.075 mL, 0.797 mmol, 1.1 eq) was added and the reaction was stirred under N_2 for 30 min. Thin-layer chromatography (60% ethyl acetate/Pet ether) then showed the reaction was not complete so an additional portion of PBr_3 (0.05 mL, 0.53 mmol, 0.7 eq) was added and stirred for 15 min. The reaction was then quenched with cold sat. $NaHCO_3$ (10 mL), extracted into DCM (3 × 50 mL), washed with brine (10 mL), dried (sodium sulfate), and the solvent removed *in vacuo* to give *N*-(4-(bromomethyl)-3-methoxyphenyl)-2-phenylacetamide (**21**) as an unstable off-white pinkish solid (0.25 g, 0.74 mmol, quantitative).

Due to the instability of the compound, it was used immediately in the next reaction without further purification or characterization.

2-phenyl-acetyl-2-methoxy-*para*-aminobenzyl β-lapa-ketol[19]

N-(4-((6-hydroxy-2,2-dimethyl-5-oxo-3,4,5,6-tetrahydro-2H-benzo[h]chromen-6-yl)methyl)-3-methoxyphenyl)-2-phenylacetamide (**22**)

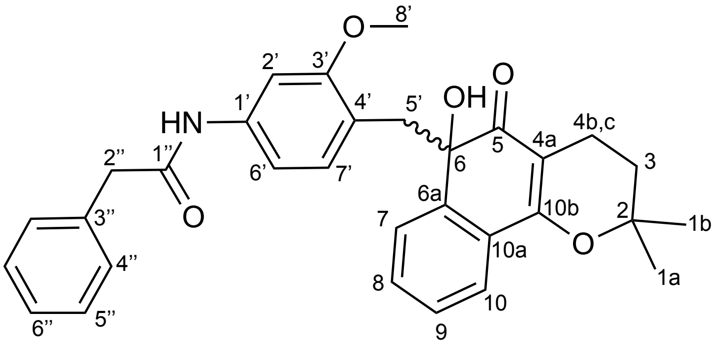

Compound **21** (0.25 g, 0.74 mmol, 1.01 eq), NaI (0.434 g, 2.9 mmol, 4 eq), β-lapachone (**1**) (0.176 g, 0.728 mmol, 1 eq) and indium(0) powder (0.127 g, 1.1 mmol, 1.5 eq) were added to anhydrous dimethylformamide (3 mL). The solution was sonicated overnight, while monitoring by thin-layer chromatography (40% ethyl acetate/Pet ether) and LC-MS. 1M HCl (~0.5 mL) was added to quench the reaction, and it was extracted with ethyl acetate (3 × 150 mL). The organic layers were combined and washed with brine (10 mL), dried (sodium sulfate), and the solvent removed *in vacuo*. The product was purified by flash column chromatography on silica gel 60 (20-80% ethyl acetate/Pet ether) and then further purified by semi-preparative HPLC for kinetics studies to give *N*-(4-((6-hydroxy-2,2-dimethyl-5-oxo-3,4,5,6-tetrahydro-2*H*-benzo[*h*]chromen-6-yl)methyl)-3-methoxyphenyl)-2-phenyl acetamide (**22**) as a peach colored solid after lyophilization (0.039 g, 0.079 mmol, 11%). HPLC Purification Method: Column = Agilent InfinityLab ZORBAX 5 Eclipse Plus C18 21.2 × 250 mm; mobile phases: A = H_2O, B = ACN; gradient: t = 0-5 min 5% B, t = 5-7 min 5-30% B, t = 7-35 min 30-95% B, t = 35-45 min 95% B. Retention time of **22** = 22.2 min.

^{1}H NMR (400 MHz, CD_3CN) δ 8.37 (brs, 1H, N<u>H</u>), 7.63 – 7.54 (m, 2H, H7, H10), 7.42 (td, *J* = 7.5, 1.4 Hz, 1H, H8), 7.36 – 7.30 (m, 5H, H9, H4", H5"), 7.29 – 7.23 (m, 1H, H6"), 7.14 (d, *J* = 2.0 Hz, 1H, H2'), 6.78 (dd, *J* = 8.1, 2.0 Hz, 1H, H6'), 6.51 (d, *J* = 8.2 Hz, 1H, H7'), 4.09 (s, 1H, O<u>H</u>), 3.59 (s, 2H, H2"), 3.43 (s, 3H, H8'), 2.98 (q, *J* = 12.9 Hz, 2H, H5'), 2.44 (dt, *J* = 17.3, 5.9 Hz, 1H, H4b,c), 2.17 – 2.09 (m, 1H, H4b,c), 1.76 – 1.58 (m, 2H, H3), 1.33 (s, 3H, H1a/H1b), 1.14 (s, 3H, H1a/H1b).

^{13}C NMR (101 MHz, CD_3CN) δ 200.7 (C5), 170.2 (C1”), 161.5 (C10b), 158.7 (C3’), 143.2 (C6a), 140.0 (C1’), 136.8 (C3”), 132.4 (C7’), 130.3 (C8), 130.1 (C4”), 129.4 (C5”), 128.4 (C10a), 128.1 (C9), 127.7 (C6”), 126.6 (C7), 123.1 (C10), 119.5 (C4’), 111.0 (C6’), 107.9 (C4a), 102.4 (C2’), 78.8 (C6), 78.6 (C2), 55.7 (C8’), 47.2 (C5’), 44.6 (C2”), 32.1 (C3), 27.5 (C1a/C1b), 25.9 (C1a/C1b), 16.5 (C4b,c).

HRMS (ESI$^+$): *m/z* calcd. for $[C_{31}H_{31}NO_5+Na]^+$ calcd. 520.2094 found 520.2124.

11.15. Synthetic Procedures for 24 and 26

para-boronate pinacol ester benzyl β-lapa-ketol[19]

6-hydroxy-2,2-dimethyl-6-(4-(4,4,5,5-tetramethyl-1,3,2-dioxaborolan-2-yl)benzyl)-2,3,4,6-tetrahydro-5H-benzo[h]chromen-5-one (**24**)

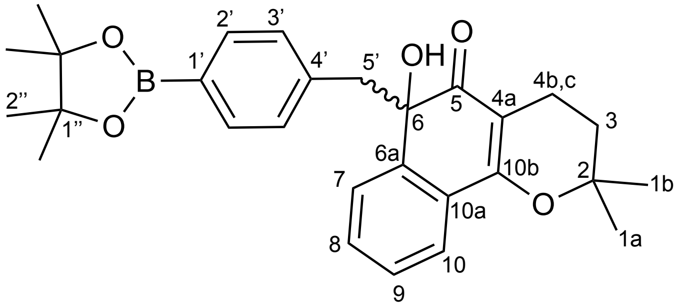

Commercial compound **23** (0.046 g, 0.154 mmol, 1.5 eq), NaI (0.062 g, 0.41 mmol, 4 eq), β-lapachone (**1**) (0.025 g, 0.1 mmol, 1 eq) and indium(0) powder (0.018 g, 0.16 mmol, 1.5 eq) were added to anhydrous dimethylformamide (3 mL). The solution was sonicated, while monitoring by thin-layer chromatography (25% ethyl acetate/Pet ether) and LC-MS. LC-MS indicated reaction completion after 1 h. 1M HCl (~0.5 mL) was added to quench the reaction, and it was extracted with ethyl acetate (3 × 150 mL). The organic layers were combined and washed with brine (10 mL), dried (sodium sulfate), and the solvent removed *in vacuo*. The product was purified by flash column chromatography on silica gel 60 (5-40% ethyl acetate/Pet ether) to give 6-(4-bromobenzyl)-6-hydroxy-2,2-dimethyl-2,3,4,6-tetrahydro-5*H*-benzo[*h*] chromen-5-one (**24**) as a beige solid (0.042 g, 0.091 mmol, 87.7%).

^{1}H NMR (400 MHz, $CDCl_3$) δ 7.64 (td, J = 7.1, 1.3 Hz, 2H, H7, H10), 7.53 – 7.42 (m, 3H, H8, H2’), 7.33 (td, J = 7.6, 1.3 Hz, 1H, H9), 6.57 (d, J = 7.9 Hz, 2H, H3’), 4.00 (s, 1H, O<u>H</u>), 3.09 (s, 2H, H5’), 2.51 (dt, J = 17.3, 5.6 Hz, 1H, H4b,c), 2.08 (dt, J = 17.1, 7.5 Hz, 1H, H4b,c), 1.59 (dd, J = 7.3, 5.4 Hz, 2H, H3), 1.38 – 1.21 (m, 15H, H2”, H1a/H1b), 0.98 (s, 3H, H1a/H1b).

^{13}C NMR (101 MHz, $CDCl_3$) δ 200.3 (C5), 162.2 (C10b), 141.3 (C6a), 138.4 (C4'), 134.1 (C2'), 130.2 (C8), 129.0 (C3'), 127.6 (C10a, C1'), 127.5 (C9), 125.7 (C7), 123.0 (C10), 107.0 (C4a), 83.7 (C1"), 78.2 (C6), 77.9 (C2), 54.8 (C5'), 31.6 (C3), 27.7 (C1a/C1b), 25.6 (C1a/C1b), 24.9 (C2"), 24.8 (C2"), 15.5 (C4b,c).

HRMS (ESI$^+$): *m/z* calcd. for $[C_{28}H_{33}BO_5+H]^+$ calcd. 460.2530 found 460.2557.

NMR are in accordance with previously reported literature.[44]

para-boronic acid benzyl β-lapa-ketol

(4-((6-hydroxy-2,2-dimethyl-5-oxo-3,4,5,6-tetrahydro-2H-benzo[h]chromen-6-yl)methyl) phenyl)boronic acid (**26**)

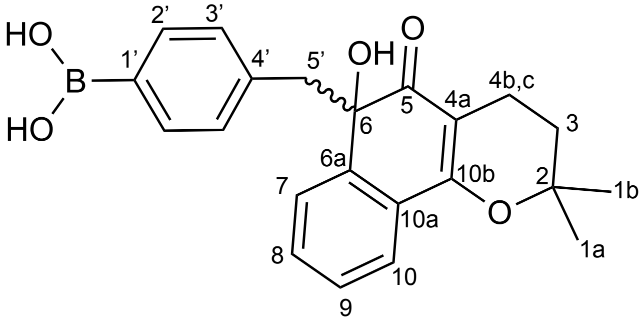

A sample of **24** was incubated with H_2O_2 for 5 min according to the conditions described in the methods section. It was then purified by semi-preparative HPLC to give (4-((6-hydroxy-2,2-dimethyl-5-oxo-3,4,5,6-tetrahydro-2*H*-benzo[*h*] chromen-6-yl)methyl)phenyl) boronic acid (**26**) as an off-white solid after lyophilization. HPLC Purification Method: Column = Agilent InfinityLab ZORBAX 5 Eclipse Plus C18 21.2 × 250 mm; mobile phases: A = H_2O + 0.1% TFA, B = ACN + 0.1% TFA; gradient: t = 0-5 min 5% B, t = 5-35 min 5-95% B, t = 35-40 min 95% B. Retention time of **26** = 23.4 min.

^1H NMR (400 MHz, 1:1 D_2O:CD_3CN) δ 7.70 – 7.65 (m, 1H, H7), 7.57 – 7.48 (m, 2H, H10, H8), 7.40 – 7.33 (m, 3H, H9, H2'), 6.42 (d, *J* = 8.1 Hz, 2H, H3'), 3.07 (dd, *J* = 30.1, 12.3 Hz, 2H, H5'), 2.31 (dt, *J* = 17.3, 5.7 Hz, 1H, H4b,c), 2.02 – 1.97 (m, 1H, H4b,c), 1.52 (ddd, *J* = 6.8, 5.4, 3.9 Hz, 2H, H3), 1.24 (s, 3H, H1a/H1b), 0.85 (s, 3H, H1a/1b).

^{13}C NMR (101 MHz, 1:1 $D_2O:CD_3CN$) δ 201.6 (C5), 163.5 (C10b), 142.5 (C6a), 138.1 (C4'), 134.1 (C2'), 131.3 (C8), 129.6 (C3'), 128.7 (C10a, C1'), 128.7 (C9), 127.0 (C7), 123.3 (C10), 109.1 (C4a), 79.4 (C6), 78.6 (C2), 54.0 (C5'), 31.7 (C3), 27.4 (C1a/C1b), 25.7 (C1a/C1b), 16.1 (C4b,c).

HRMS (ESI$^+$): *m/z* calcd. for $[C_{22}H_{23}BO_5+Na]^+$ calcd. 401.1535 found 401.1518.

NMR are in accordance with previously reported literature.[44]

para-hydroxybenzyl β-lapa-ketol

(R)-6-hydroxy-6-(4-hydroxybenzyl)-2,2-dimethyl-2,3,4,6-tetrahydro-5H-benzo[h]chromen-5-one (**3a**)

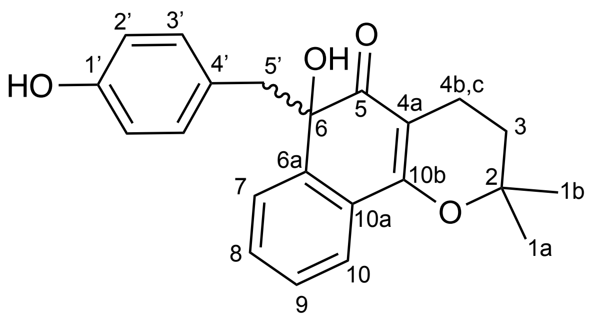

A sample of **24** was incubated with H_2O_2 for 30 min according to the conditions described in the methods section. It was then purified by semi-preparative HPLC to give (*R*)-6-hydroxy-6-(4-hydroxybenzyl)-2,2-dimethyl-2,3,4,6-tetrahydro-5*H*-benzo[*h*]chromen-5-one (**3a**) as a peach colored solid after lyophilization. HPLC Purification Method: Column = Agilent InfinityLab ZORBAX 5 Eclipse Plus C18 21.2 × 250 mm; mobile phases: A = H_2O + 0.1% TFA, B = ACN + 0.1% TFA; gradient: t = 0-5 min 5% B, t = 5-35 min 5-95% B, t = 35-40 min 95% B. Retention time of **3a** = 25.1 min.

^1H NMR (400 MHz, MeOD) δ 7.72 (ddd, J = 7.8, 1.3, 0.5 Hz, 1H, H7), 7.61 (ddd, J = 7.9, 1.4, 0.5 Hz, 1H, H10), 7.51 (td, J = 7.6, 1.4 Hz, 1H, H8), 7.36 (ddd, J = 7.8, 7.4, 1.3 Hz, 1H, H9), 6.39 (d, J = 8.6 Hz, 2H, H2'), 6.25 (d, J = 8.5 Hz, 2H, H3'), 3.12 – 2.92 (m, 2H, H5'), 2.42 (dt, J = 17.3, 5.6 Hz, 1H, H4b,c), 2.06 (ddd, J = 17.3, 8.4, 6.8 Hz, 1H, H4b,c), 1.68 – 1.56 (m, 2H, H3), 1.35 (s, 3H, H1a/H1b), 1.03 (s, 3H, H1a/H1b).

^{13}C NMR (101 MHz, MeOD) δ 202.4 (C5), 163.5 (C10b), 157.4 (C1'), 143.9 (C6a), 131.2 (C3'), 131.0 (C8), 129.6 (C10a), 128.5 (C9), 127.2 (C7), 126.8 (C4'), 123.6 (C10), 115.3 (C2'), 109.3

(C4a), 79.0 (C6), 79.0 (C2), 53.7 (C5'), 32.5 (C3), 27.7 (C1a/C1b), 25.8 (C1a/C1b), 16.6 (C4b,c).

HRMS (ESI$^+$): neutral mass calcd. for [$C_{22}H_{22}O_4$] calcd. 350.1518 found 350.1524.

Section 12. NMR Spectra of Synthesized Compounds

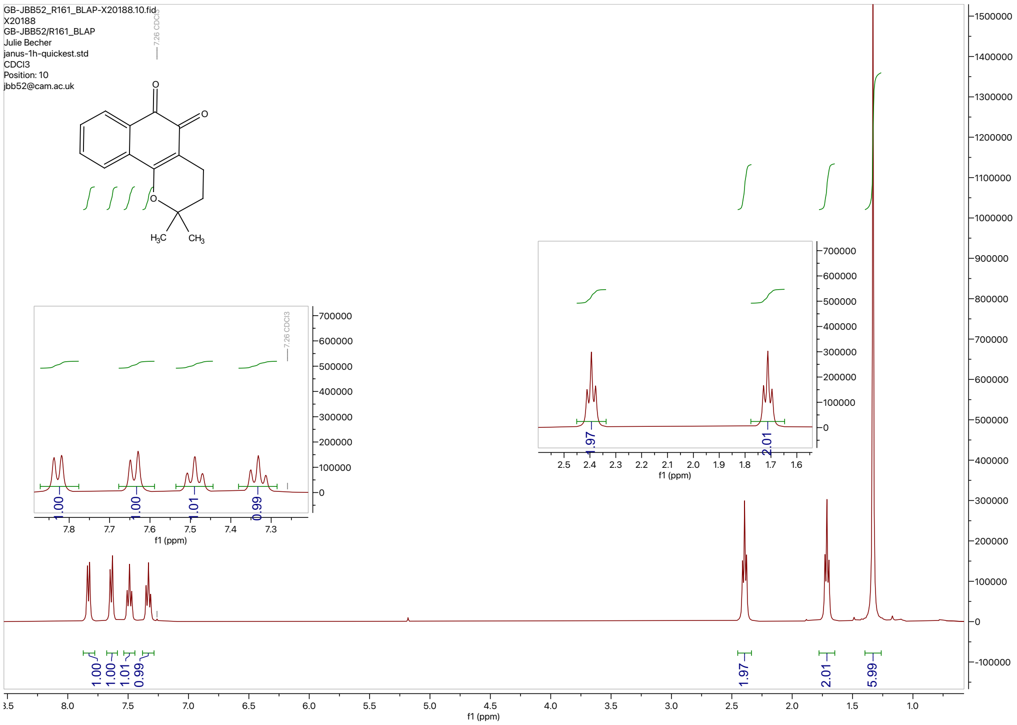

^{1}H NMR (600 MHz, CDCl$_3$) of β-Lapachone 1

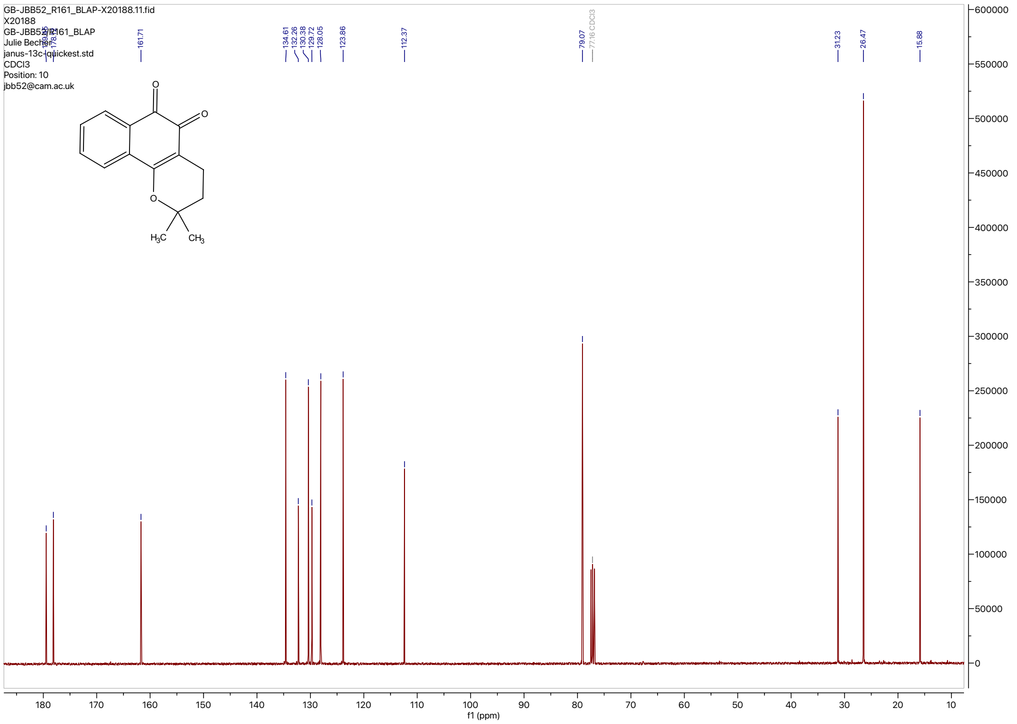

^{13}C NMR (151 MHz, CDCl$_3$) of β-Lapachone 1.

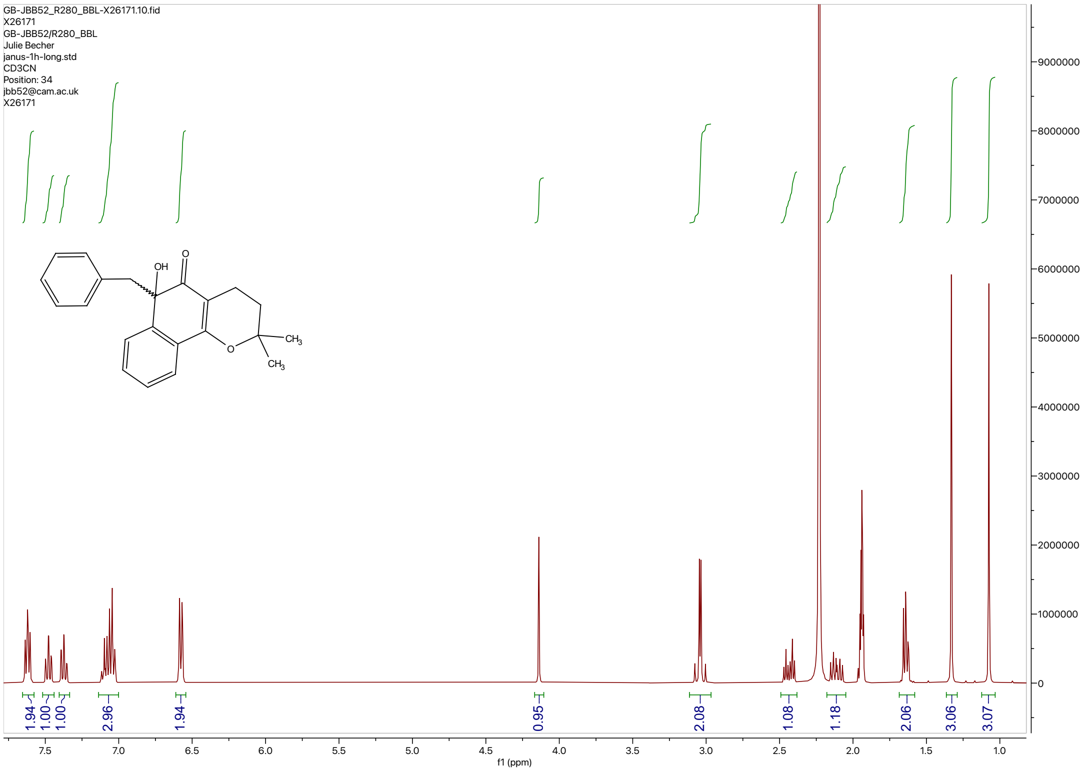

^{1}H NMR (400 MHz, CD_3CN) of 27.

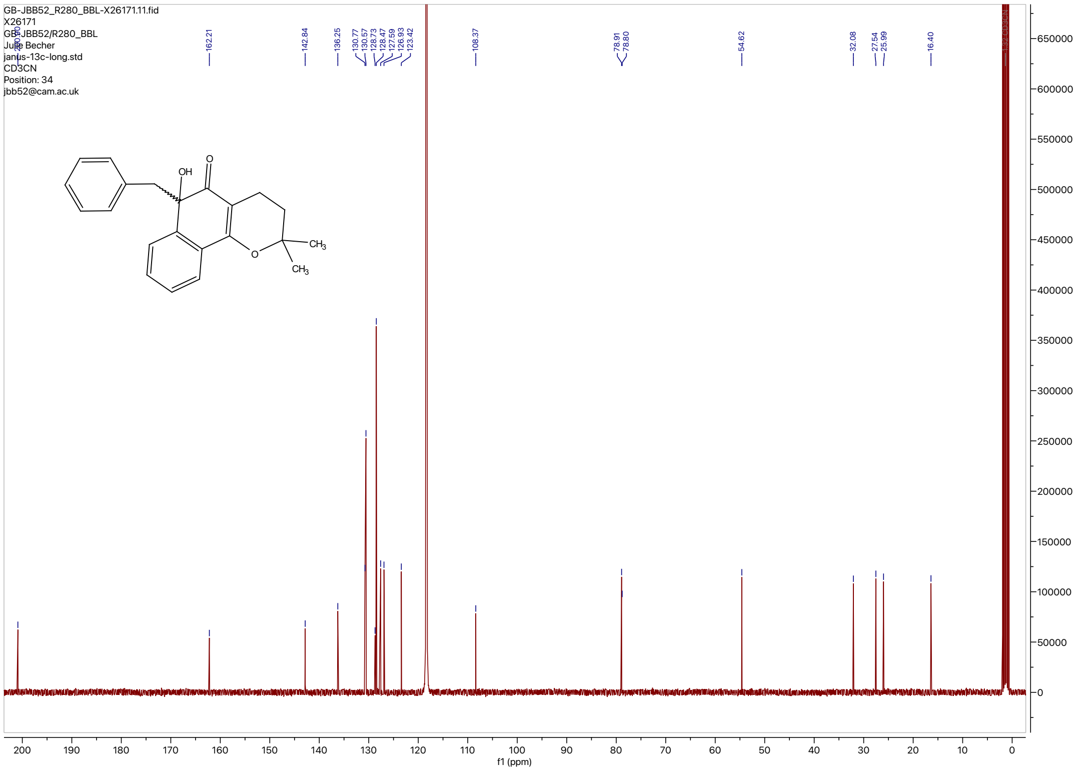

^{13}C NMR (101 MHz, CD_3CN) of 27.

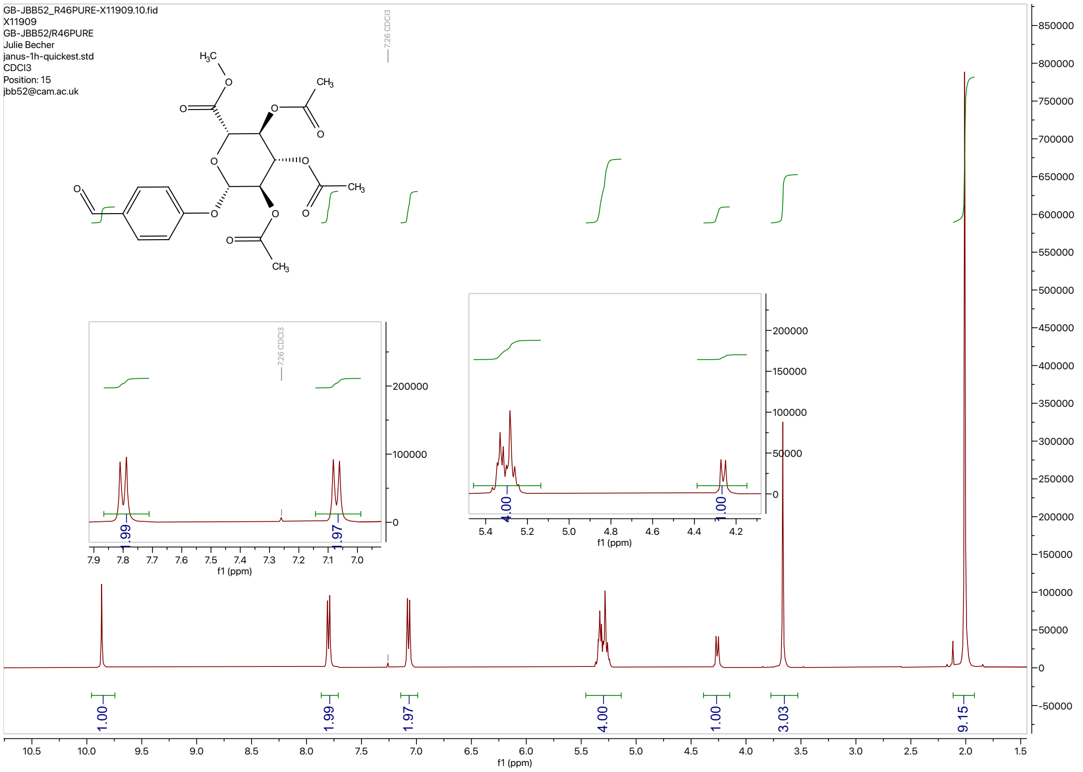

^{1}H NMR (600 MHz, $CDCl_3$) of 12a.

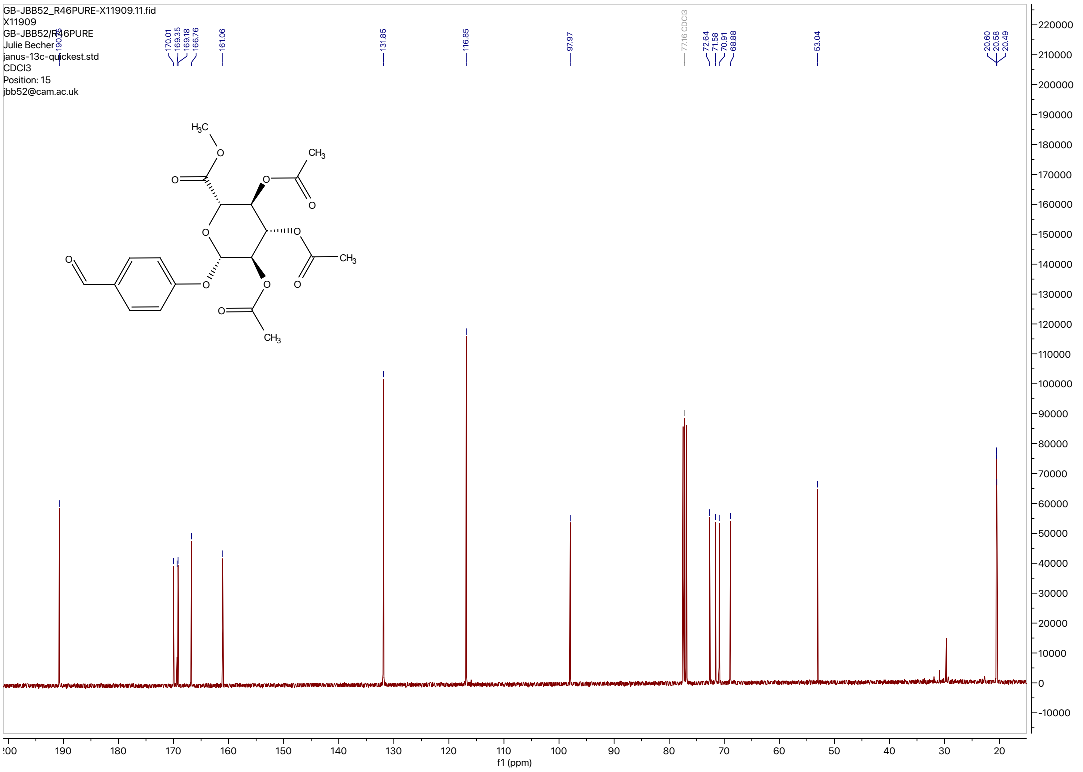

^{13}C NMR (151 MHz, $CDCl_3$) of 12a.

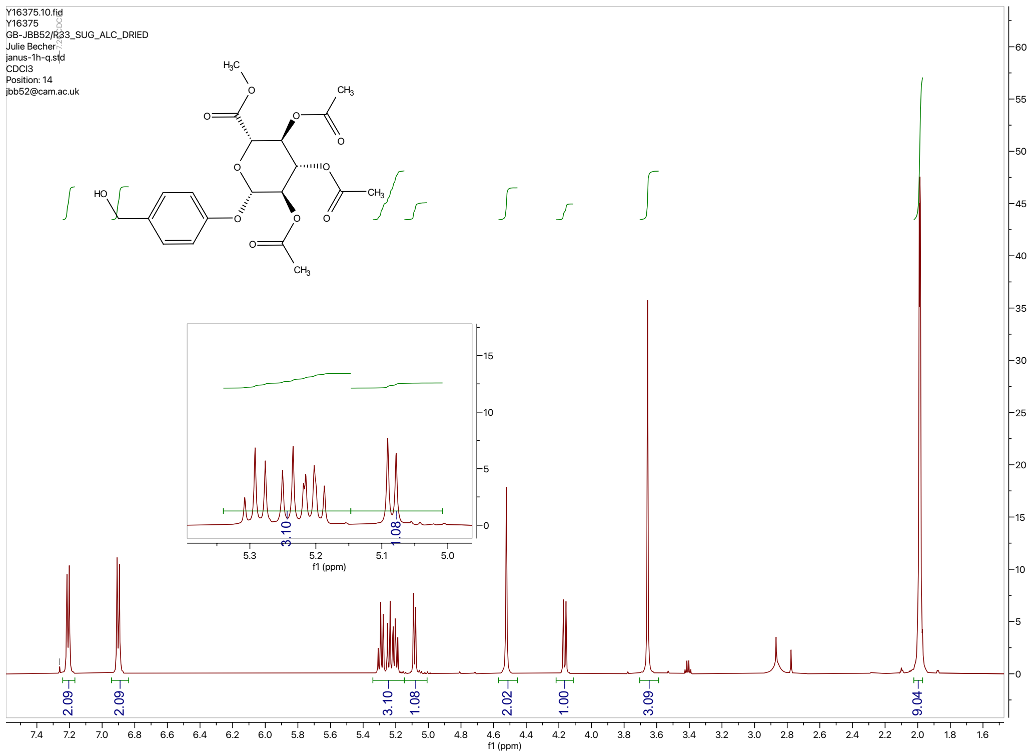

^{1}H NMR (600 MHz, $CDCl_3$) of 13a.

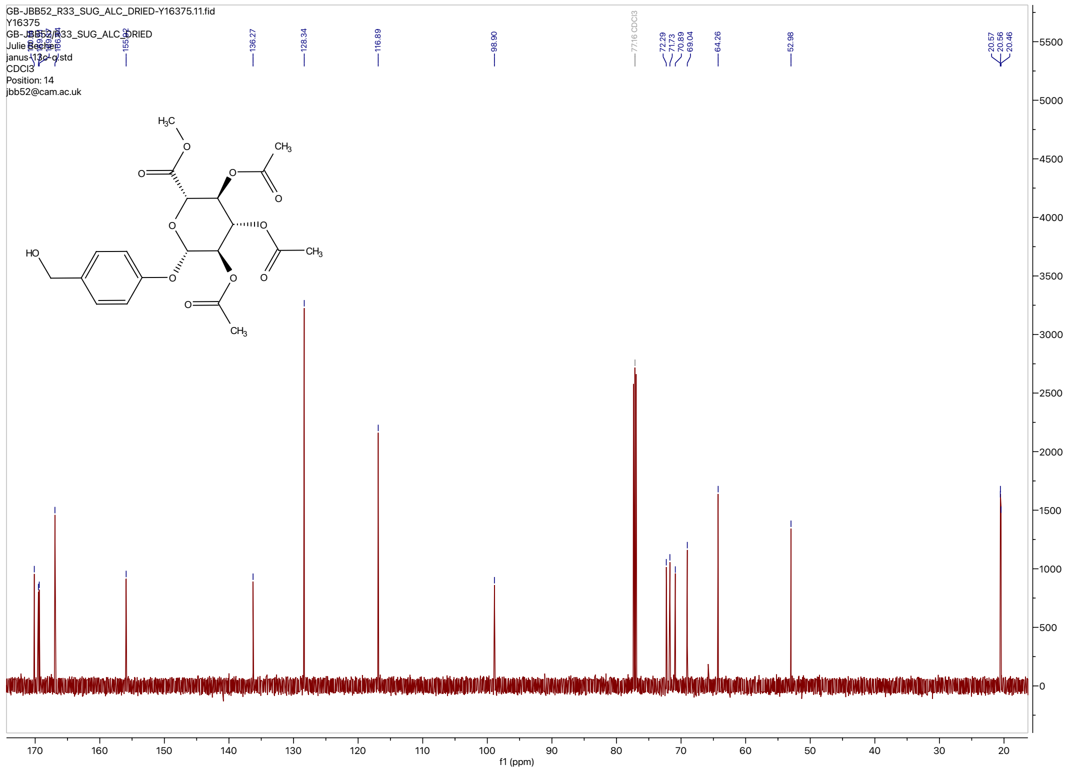

^{13}C NMR (151 MHz, $CDCl_3$) of 13a.

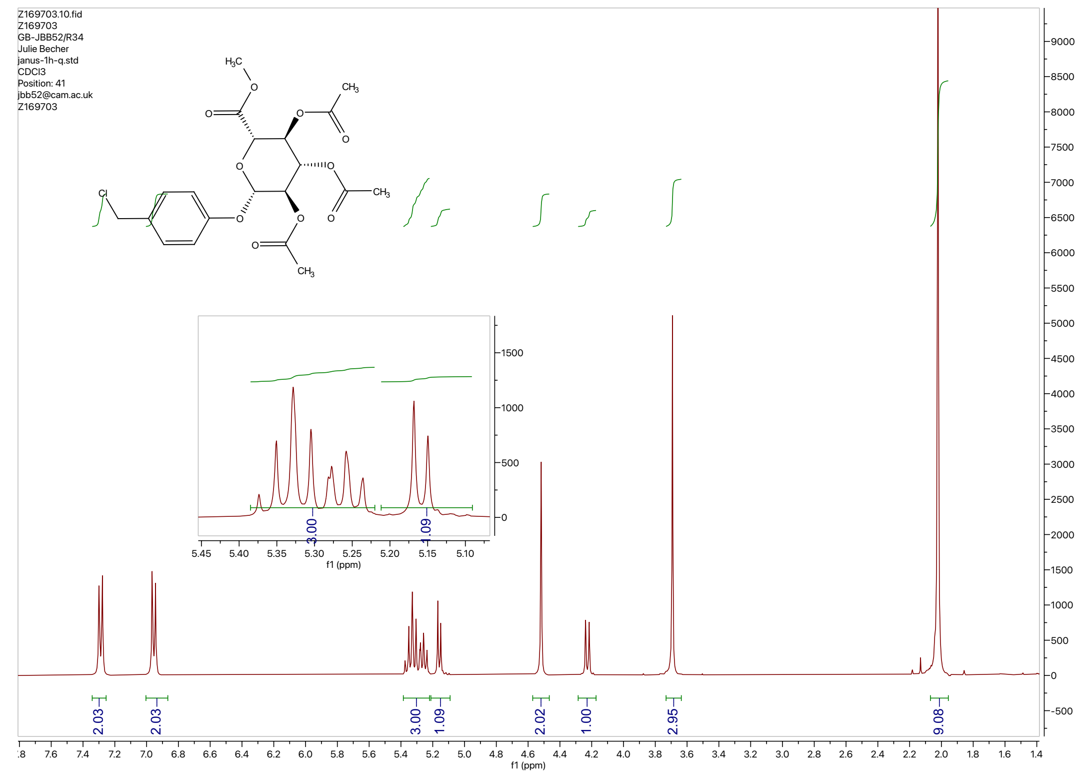

^{1}H NMR (400 MHz, $CDCl_3$) of 14a.

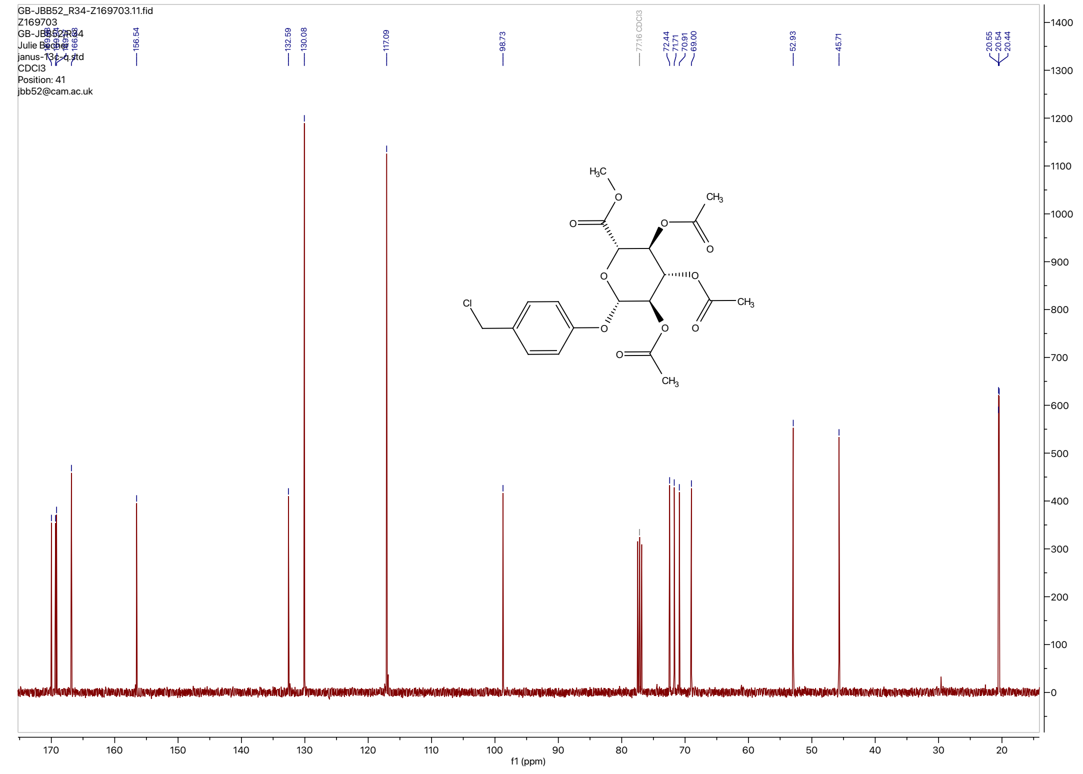

^{13}C NMR (100 MHz, $CDCl_3$) of 14a.

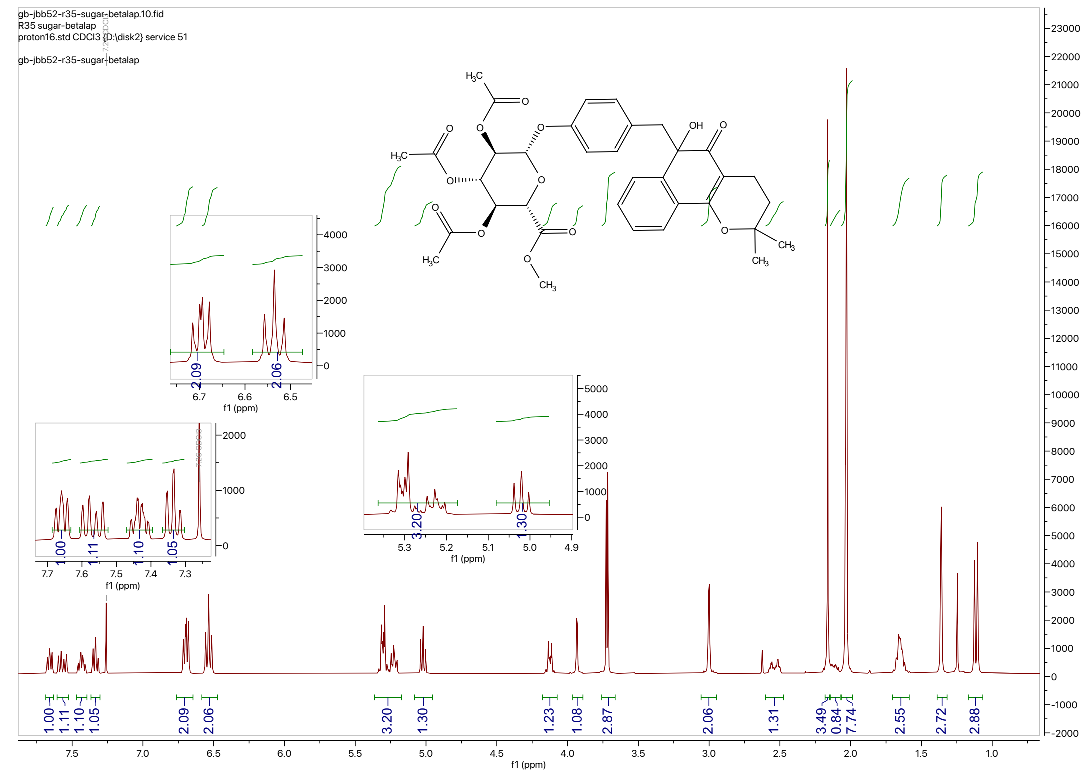

^{1}H NMR (400 MHz, CDCl$_3$) of 15a.

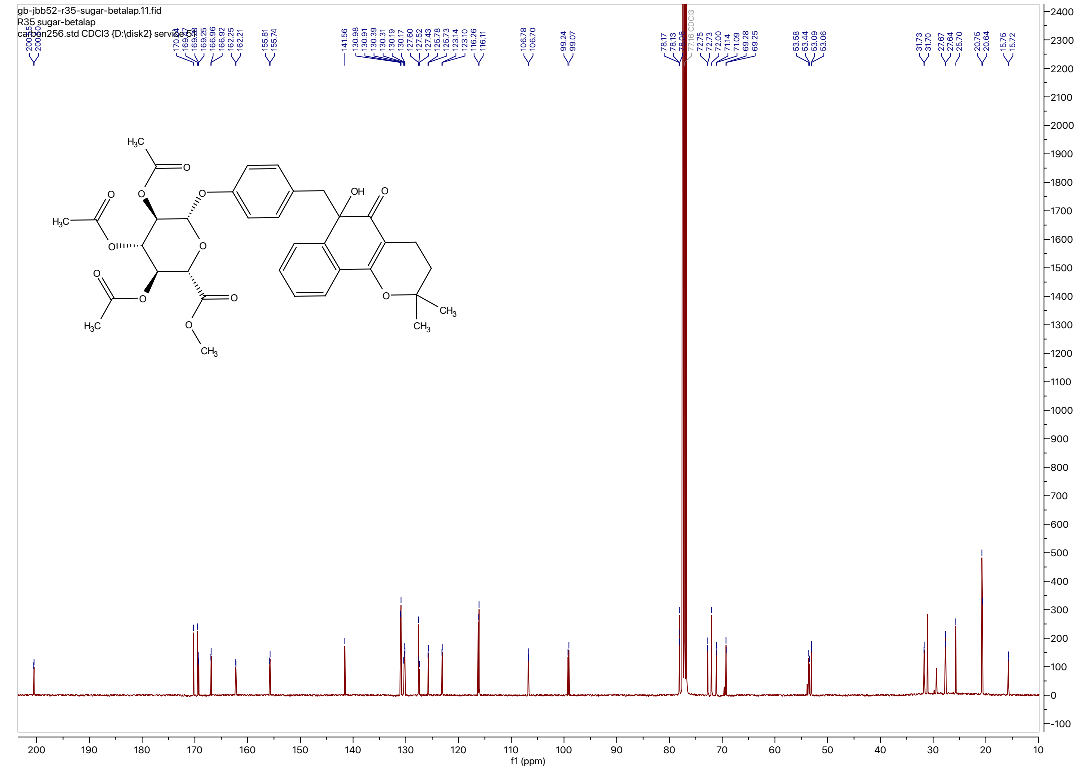

^{13}C NMR (100 MHz, CDCl$_3$) of 15a.

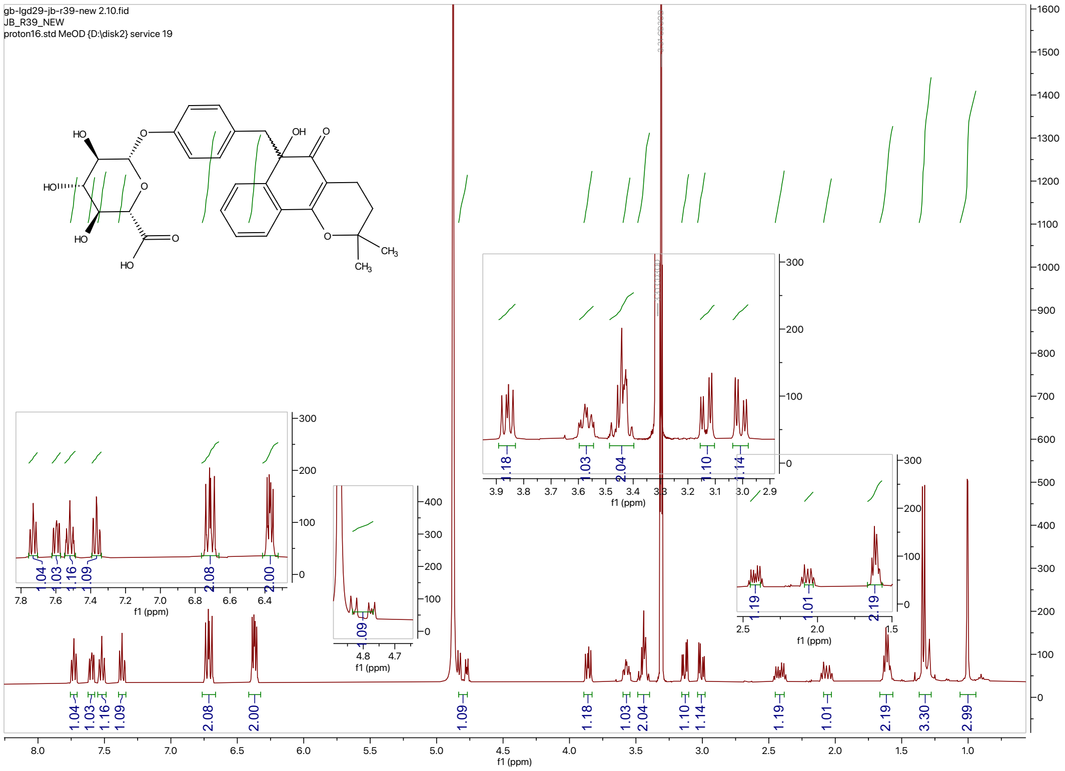

^{1}H NMR (400 MHz, MeOD) of 16a.

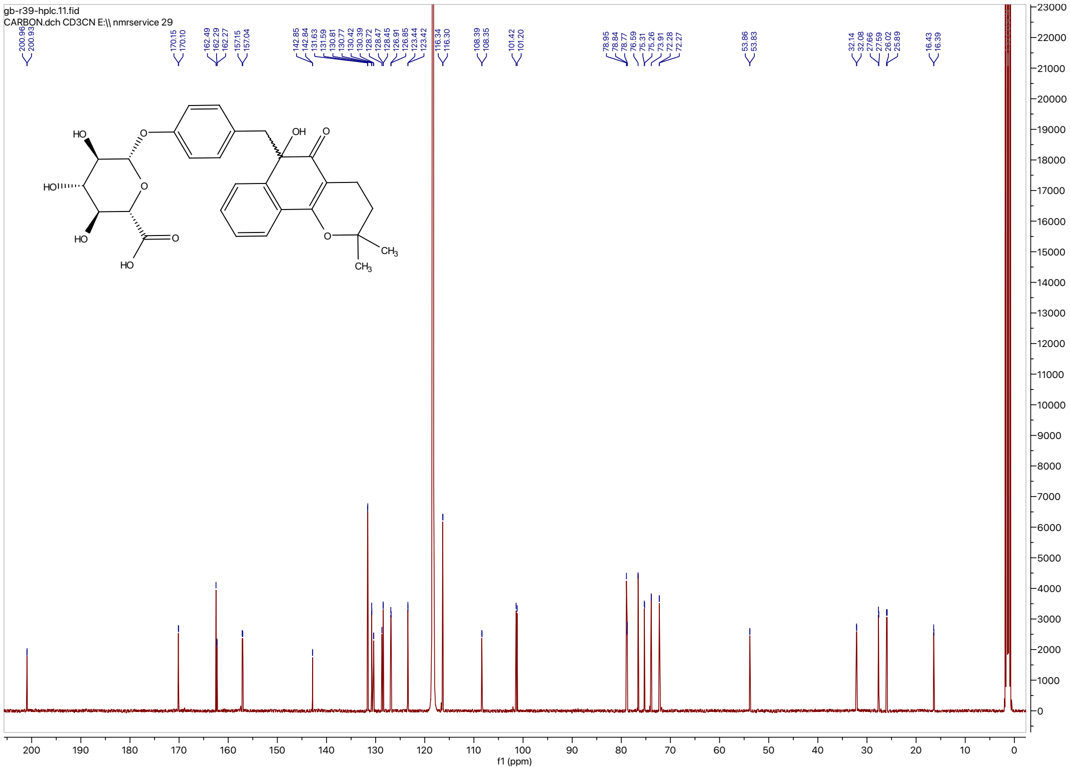

^{13}C NMR (126 MHz, CD_3CN) of 16a.

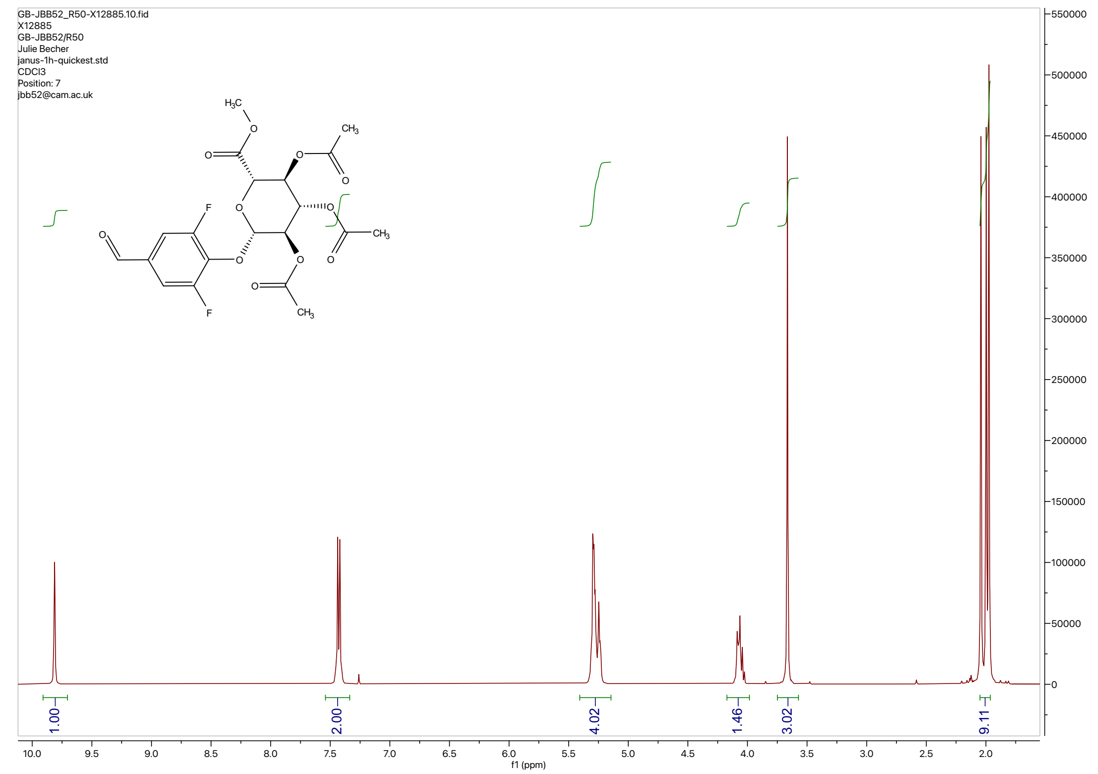

^{1}H NMR (400 MHz, $CDCl_3$) of 12b.

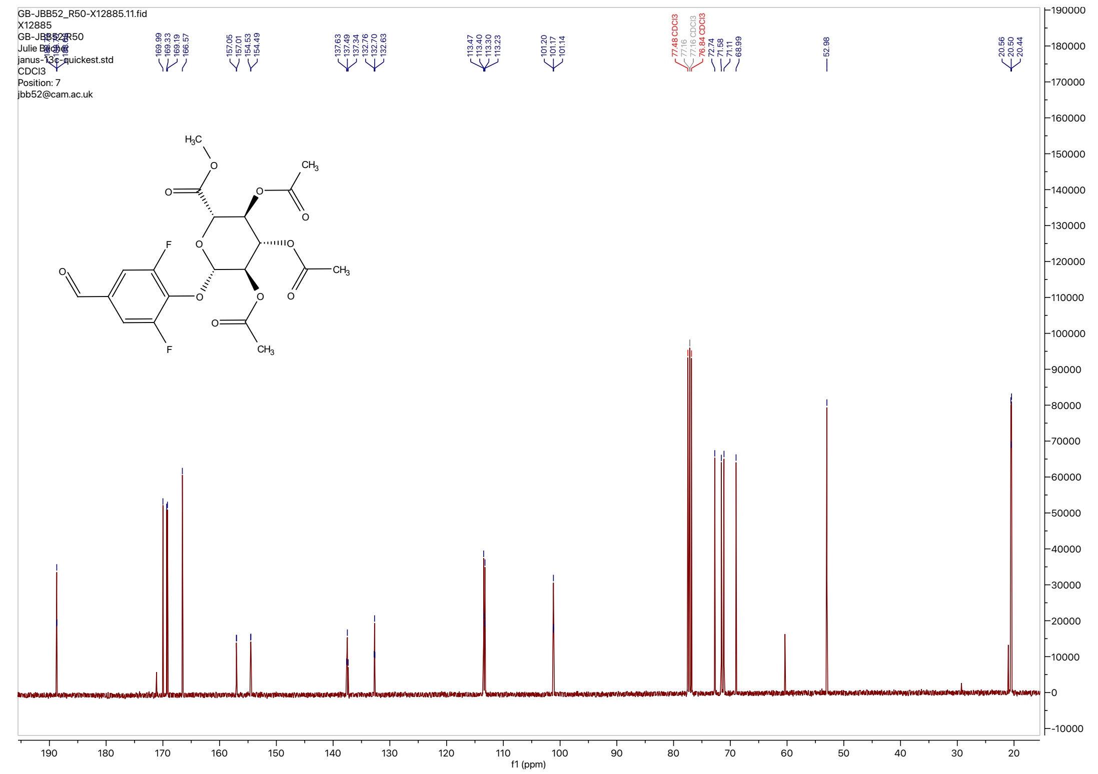

^{13}C NMR (101 MHz, $CDCl_3$) of 12b.

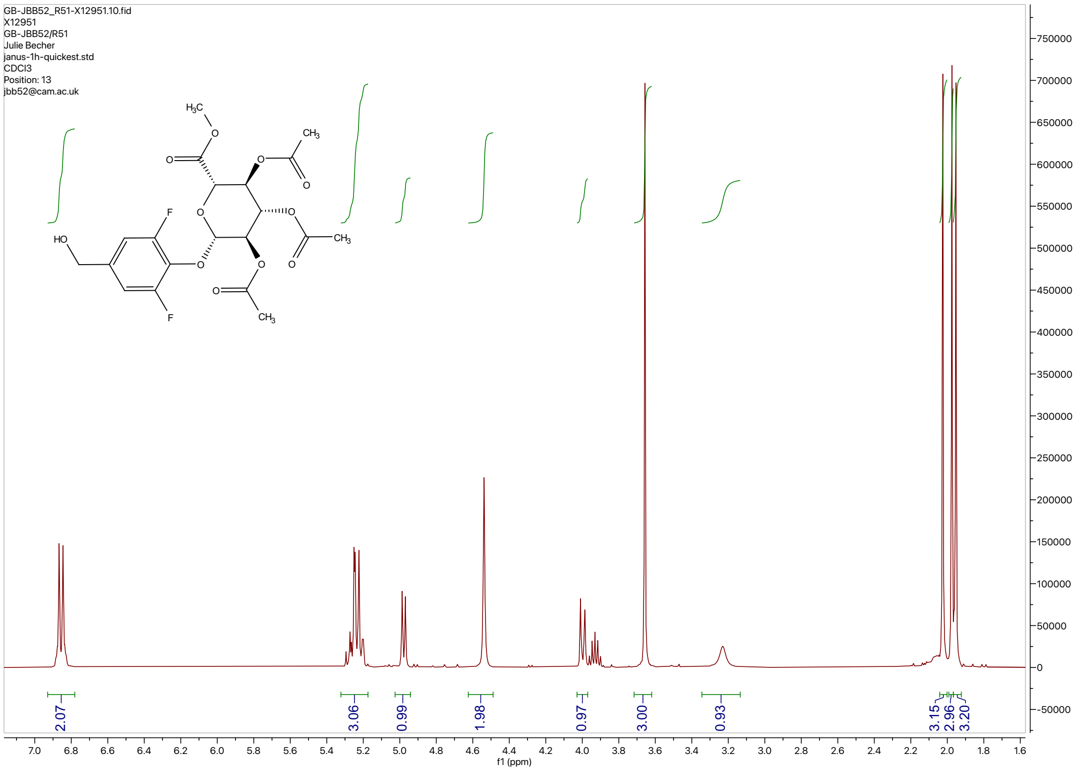

^{1}H NMR (400 MHz, CDCl$_3$) of 13b.

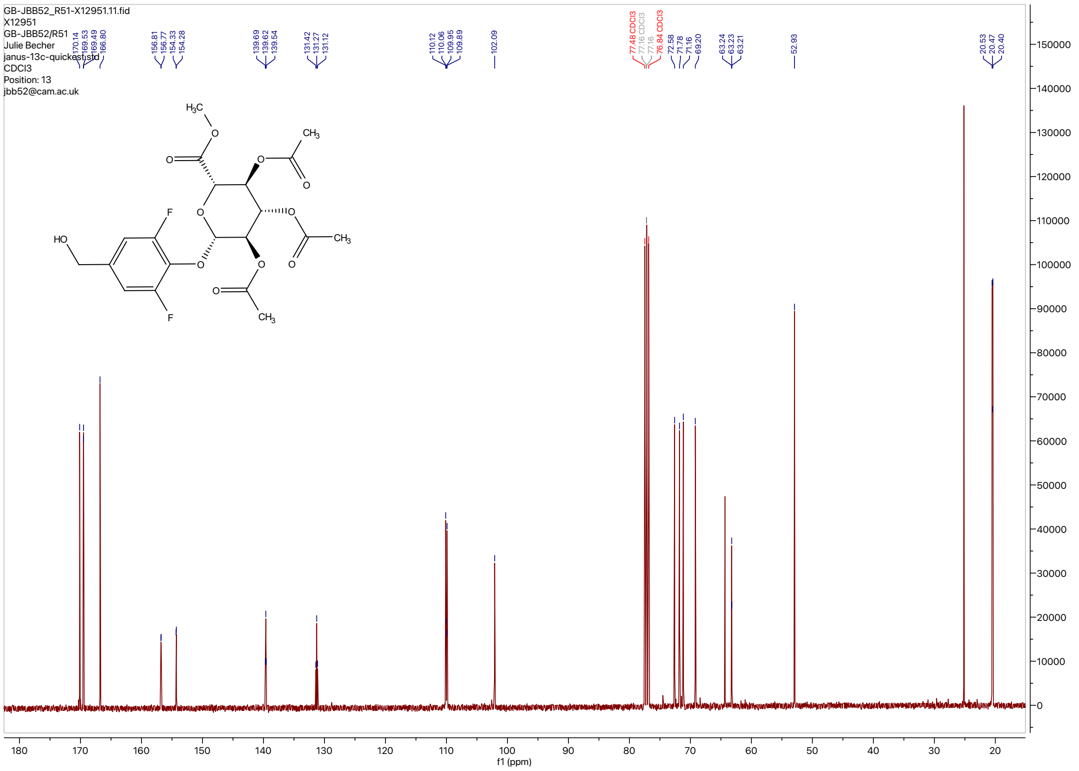

^{13}C NMR (101 MHz, CDCl$_3$) of 13b.

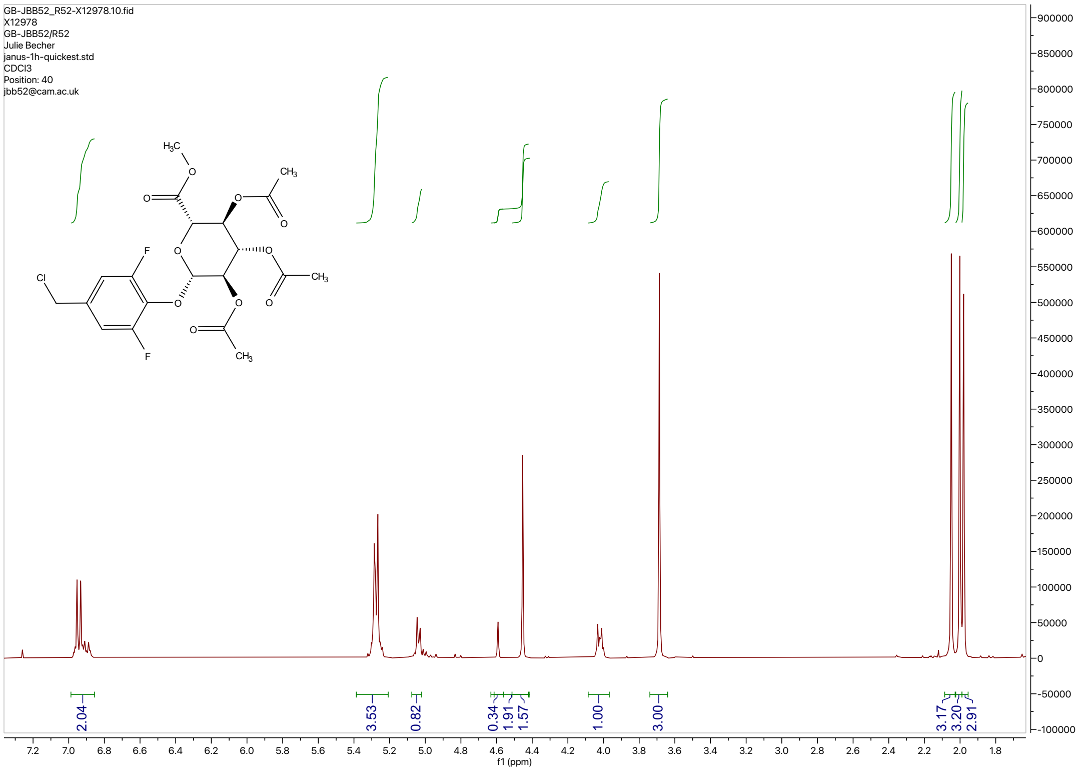

^{1}H NMR (400 MHz, $CDCl_3$) of 14b.

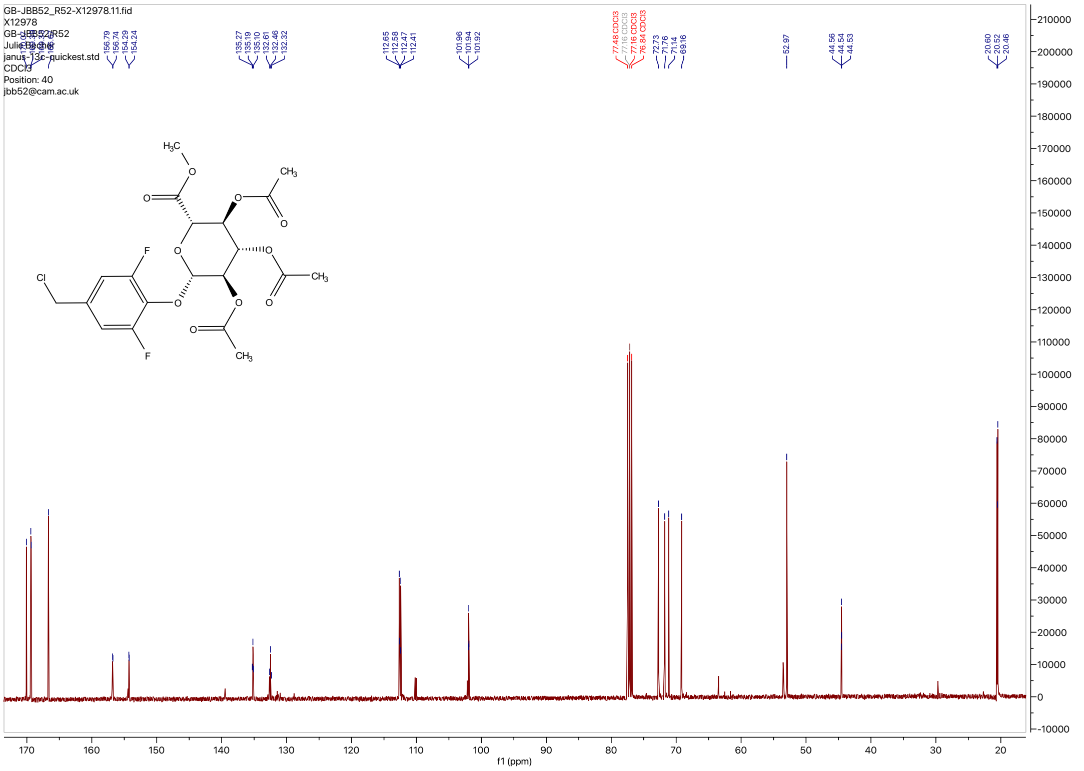

^{13}C NMR (101 MHz, $CDCl_3$) of 14b.

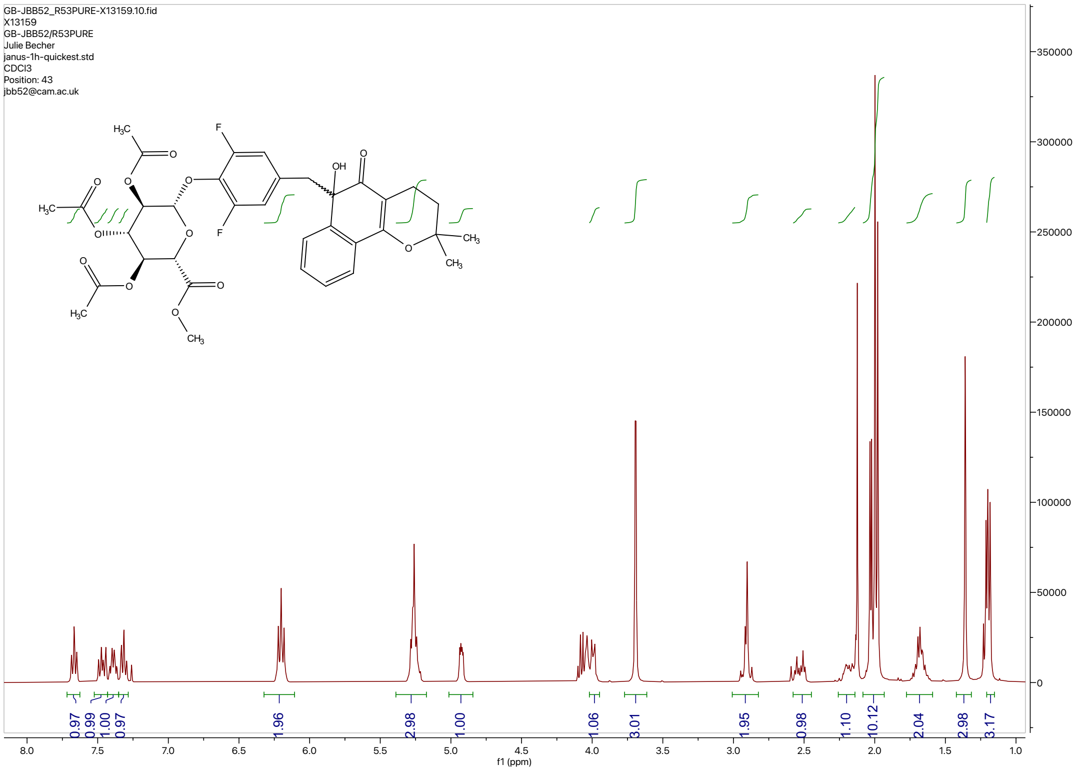

^{1}H NMR (400 MHz, $CDCl_3$) of 15b.

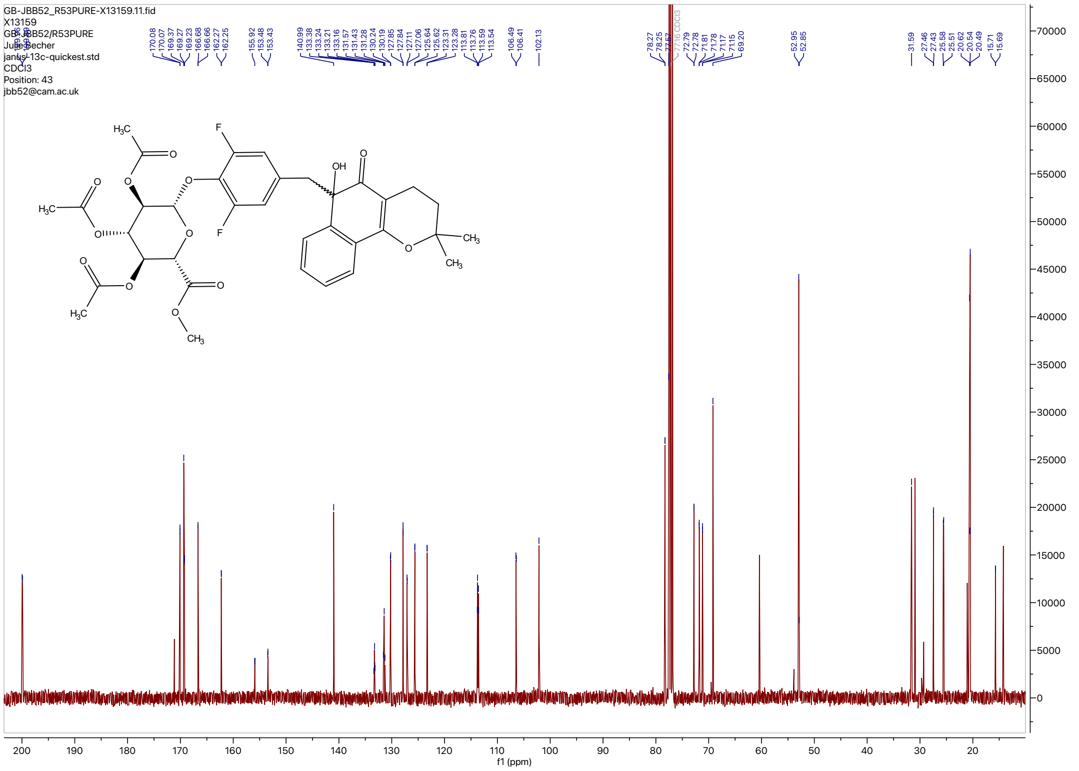

^{13}C NMR (101 MHz, $CDCl_3$) of 15b.

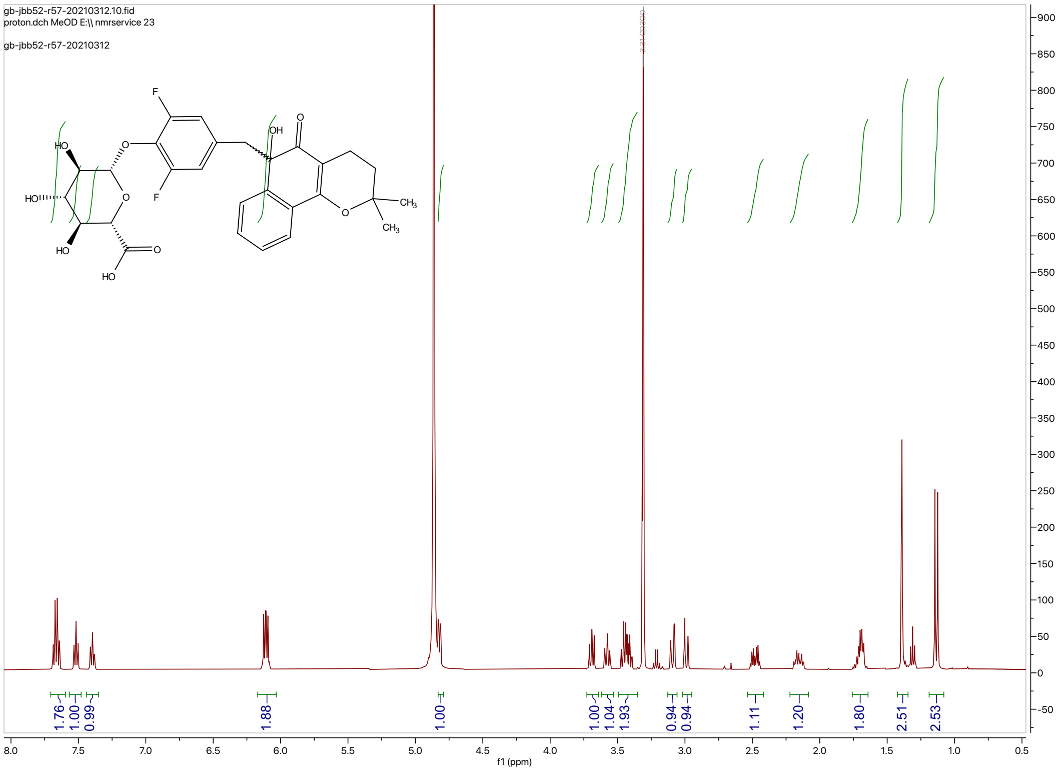

^{1}H NMR (500 MHz, MeOD) of 16b.

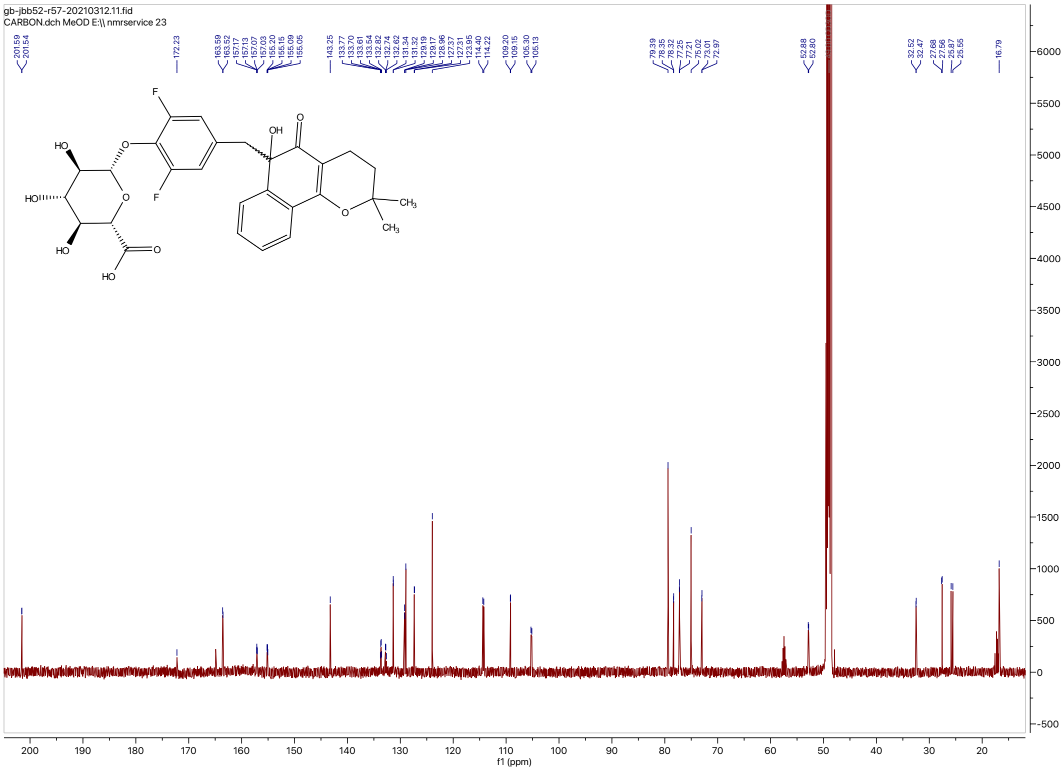

^{13}C NMR (126 MHz, MeOD) of 16b.

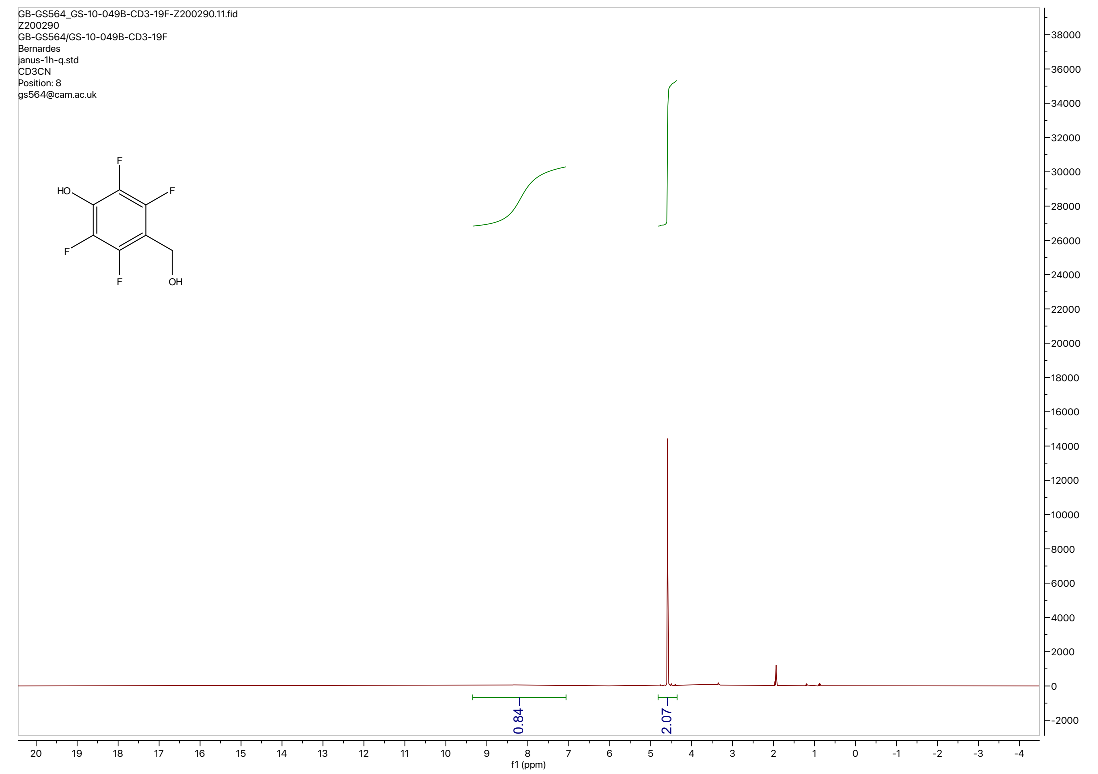

^{1}H NMR (400 MHz, CD_3CN) of 6.

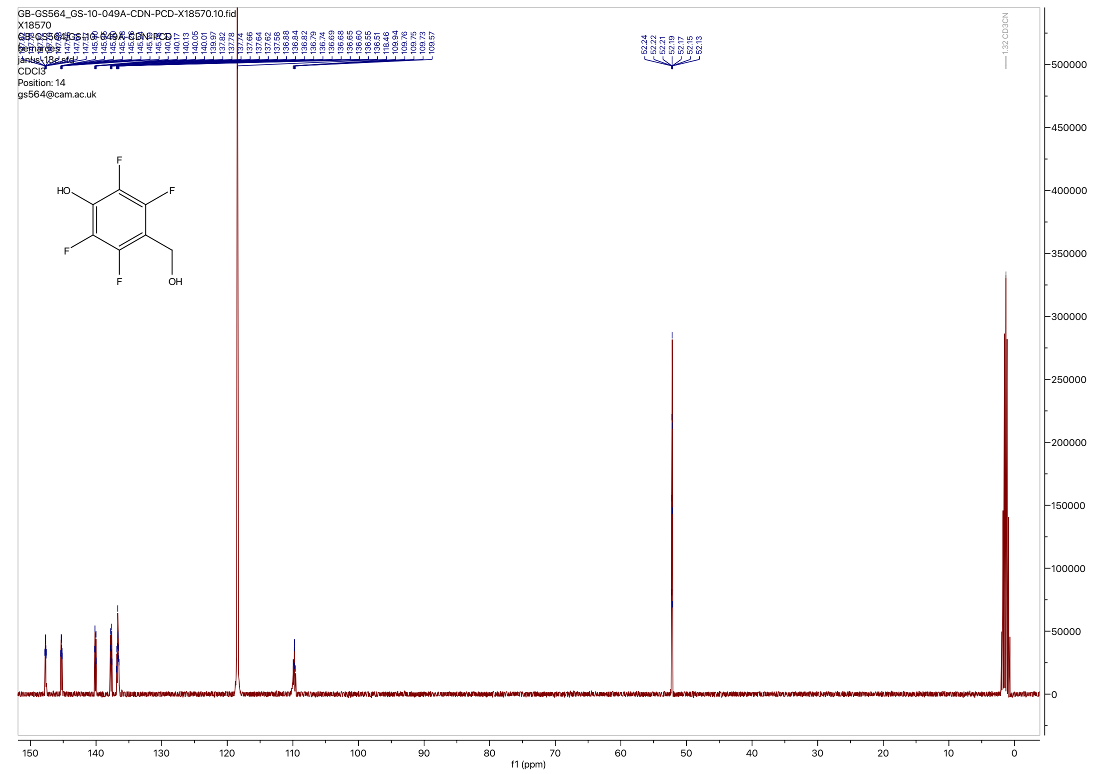

^{13}C NMR (101 MHz, $CDCl_3$) of 6.

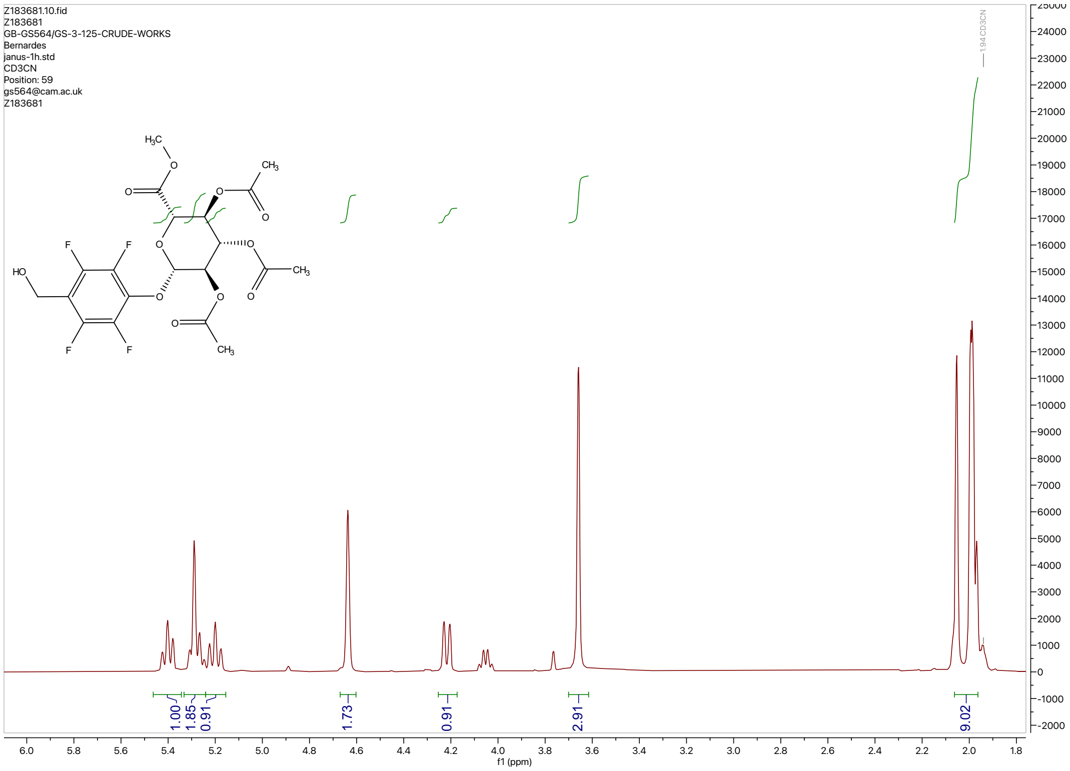

^{1}H NMR (400 MHz, CD_3CN) of 13c.

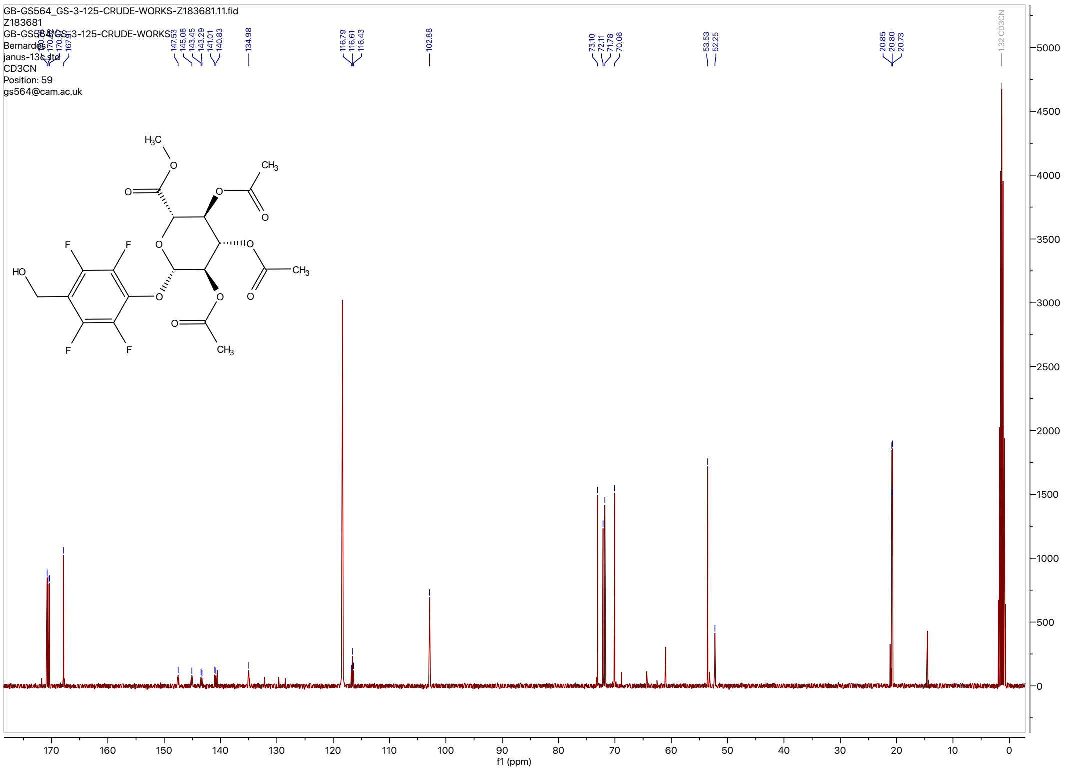

^{13}C NMR (101 MHz, CD_3CN) of 13c.

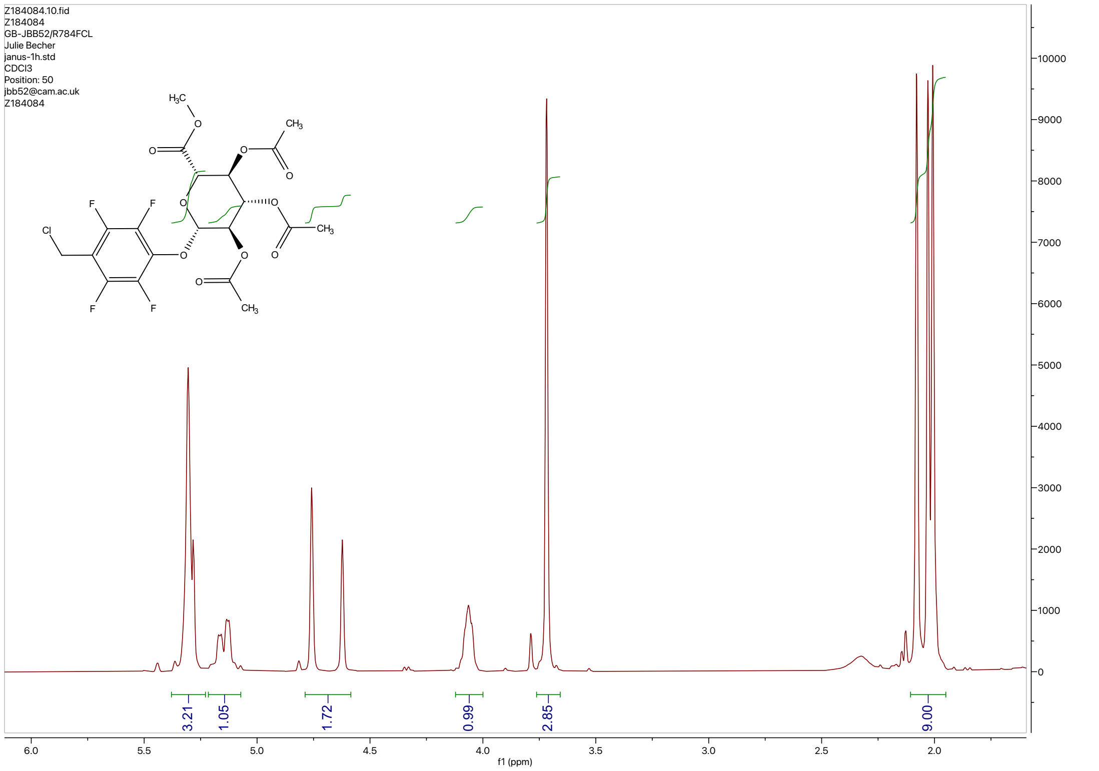

^{1}H NMR (400 MHz, CDCl$_3$) of 14c.

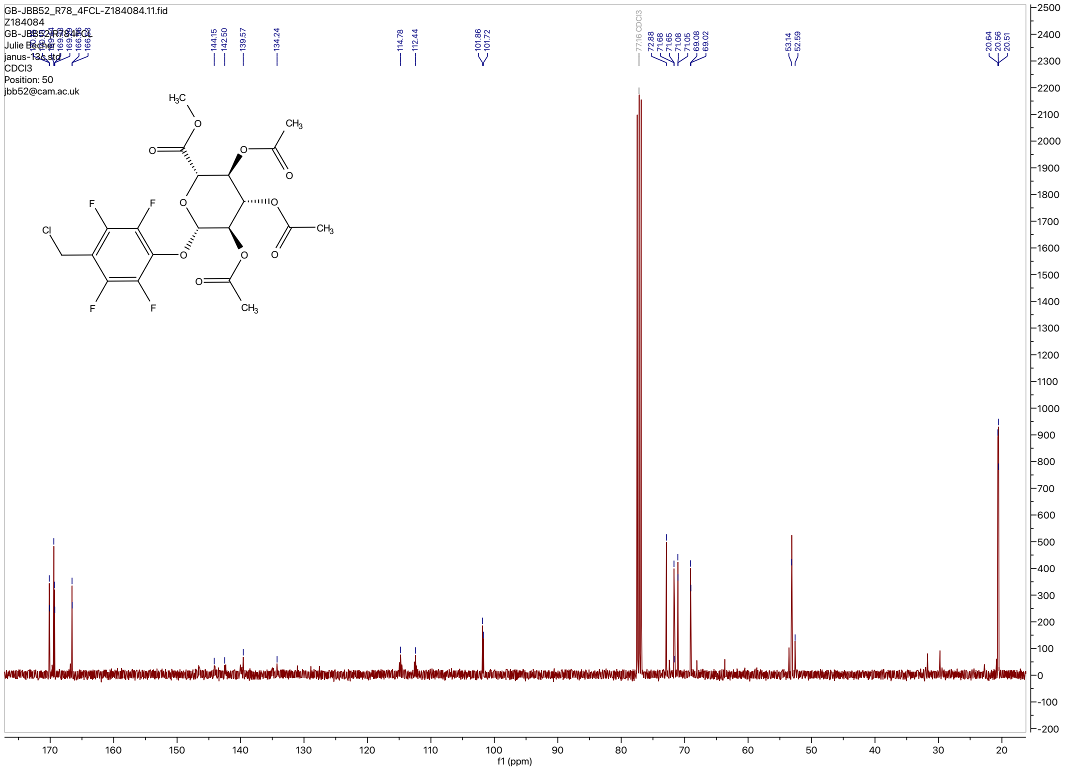

^{13}C NMR (101 MHz, CDCl$_3$) of 14c.

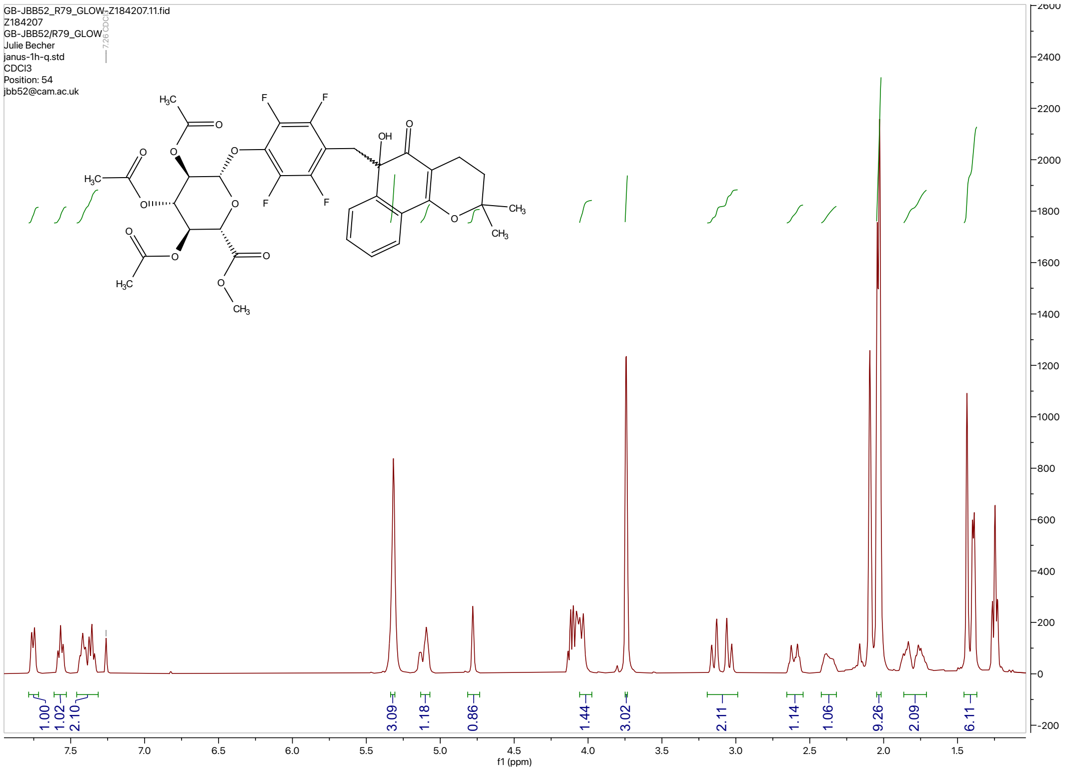

^{1}H NMR (700 MHz, $CDCl_3$) of 15c.

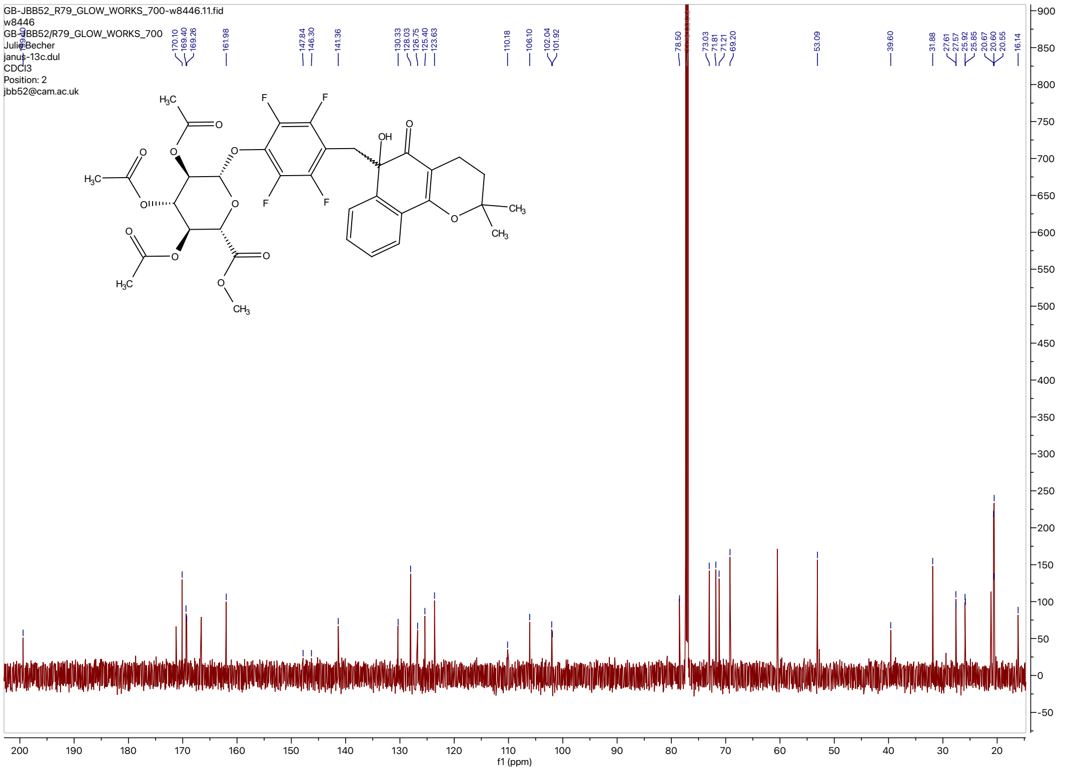

^{13}C NMR (176 MHz, $CDCl_3$) of 15c.

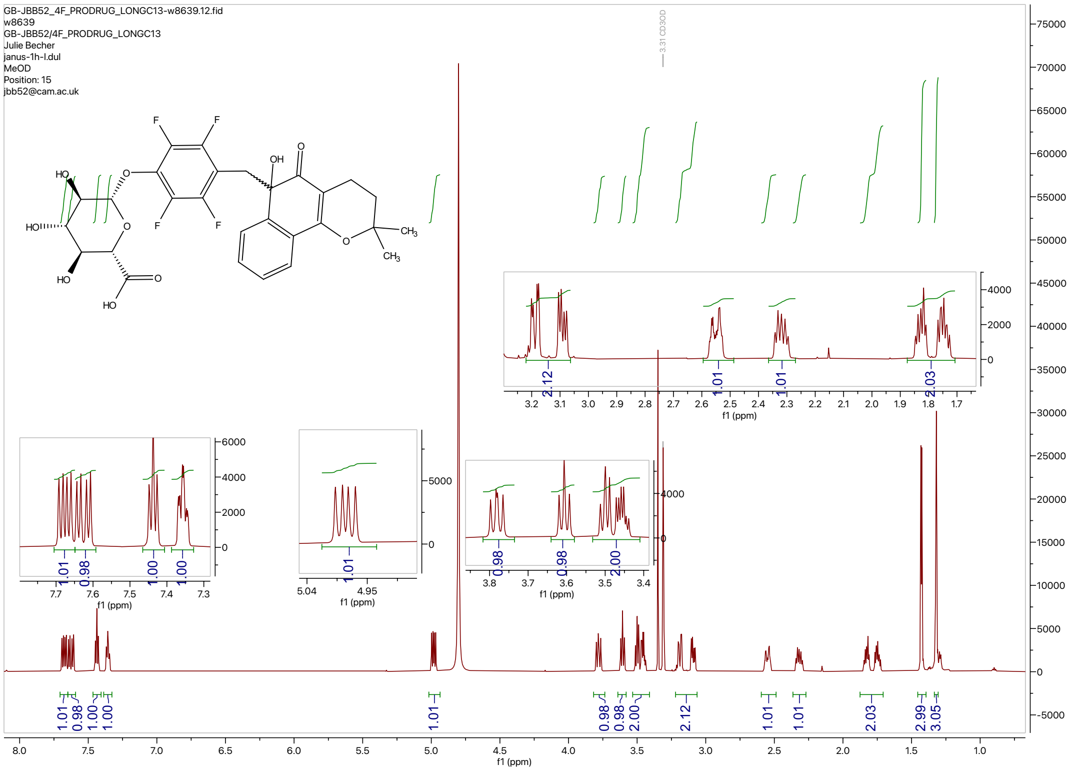

^{1}H NMR (700 MHz, MeOD) of 16c.

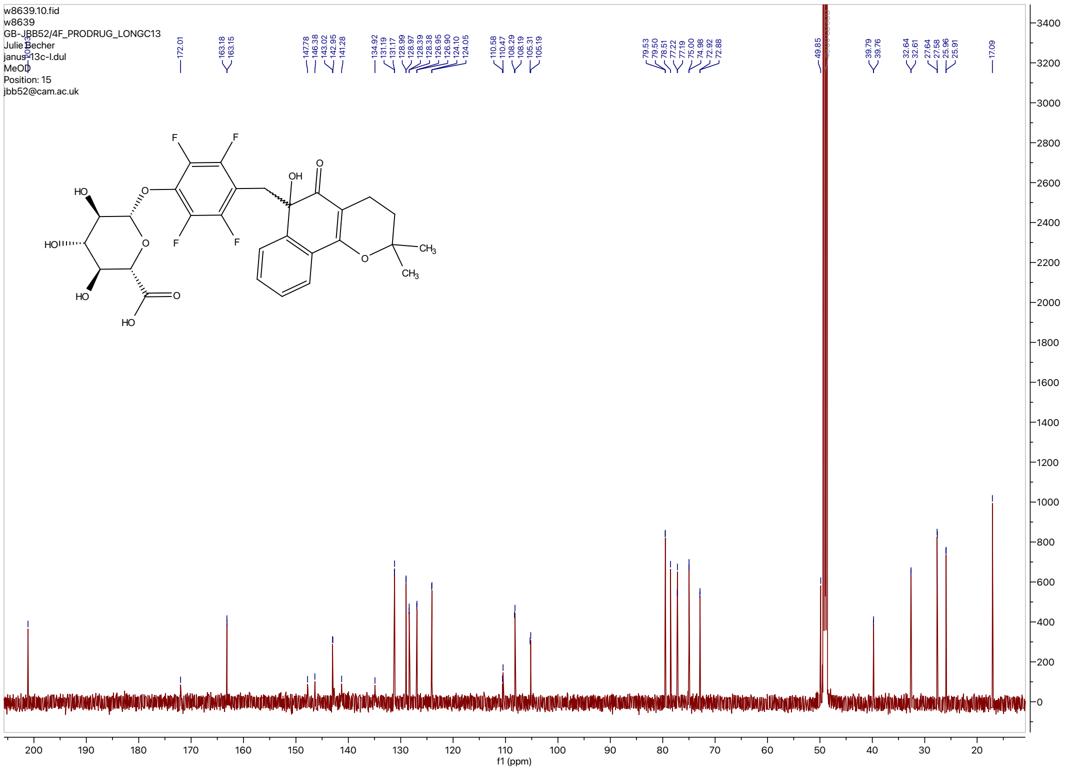

^{13}C NMR (176 MHz, MeOD) of 16c.

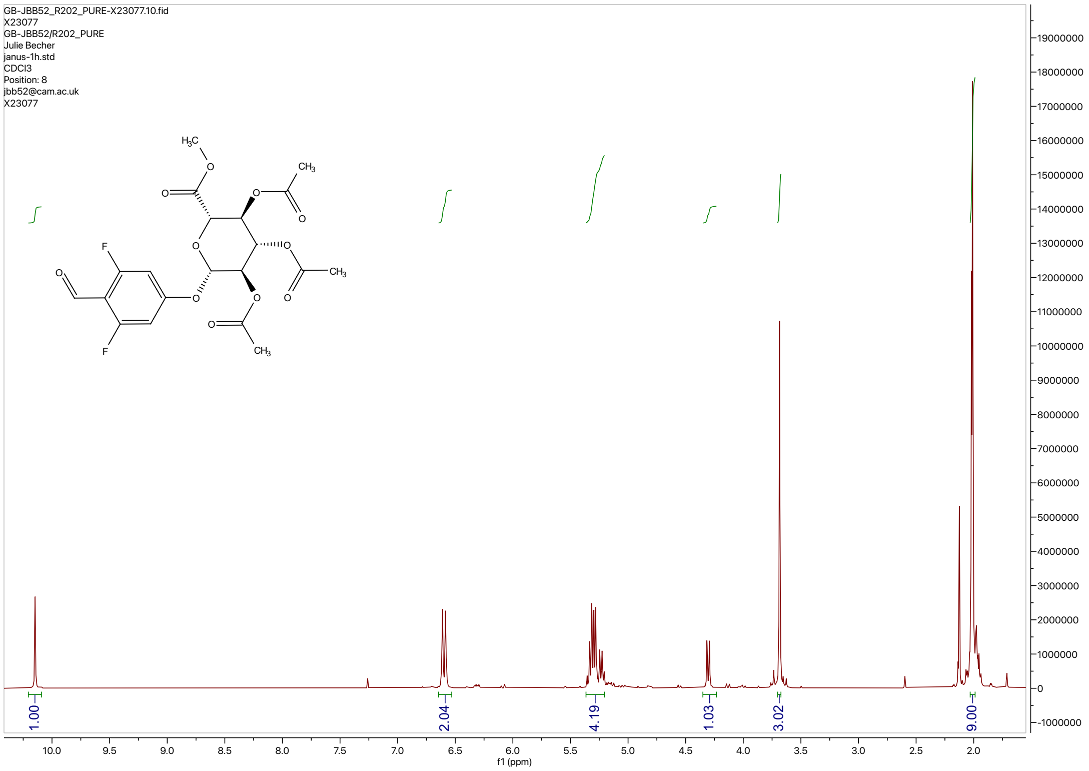

^1H NMR (400 MHz, $CDCl_3$) of 12d.

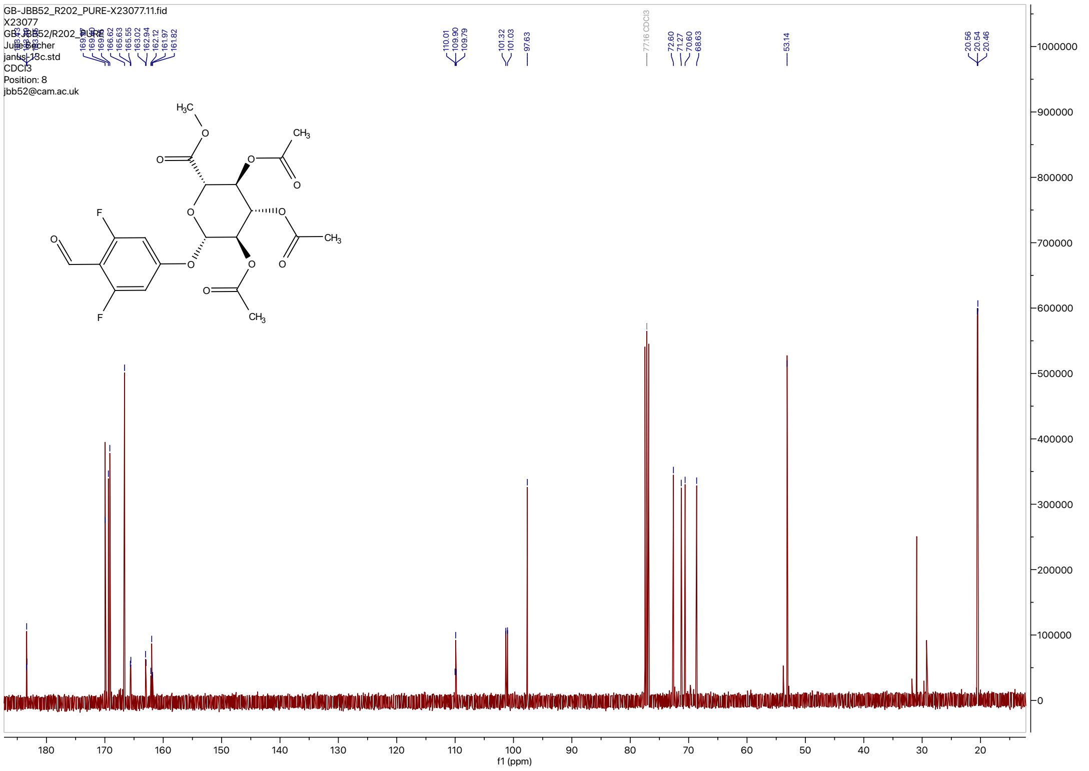

^{13}C NMR (101 MHz, $CDCl_3$) of 12d.

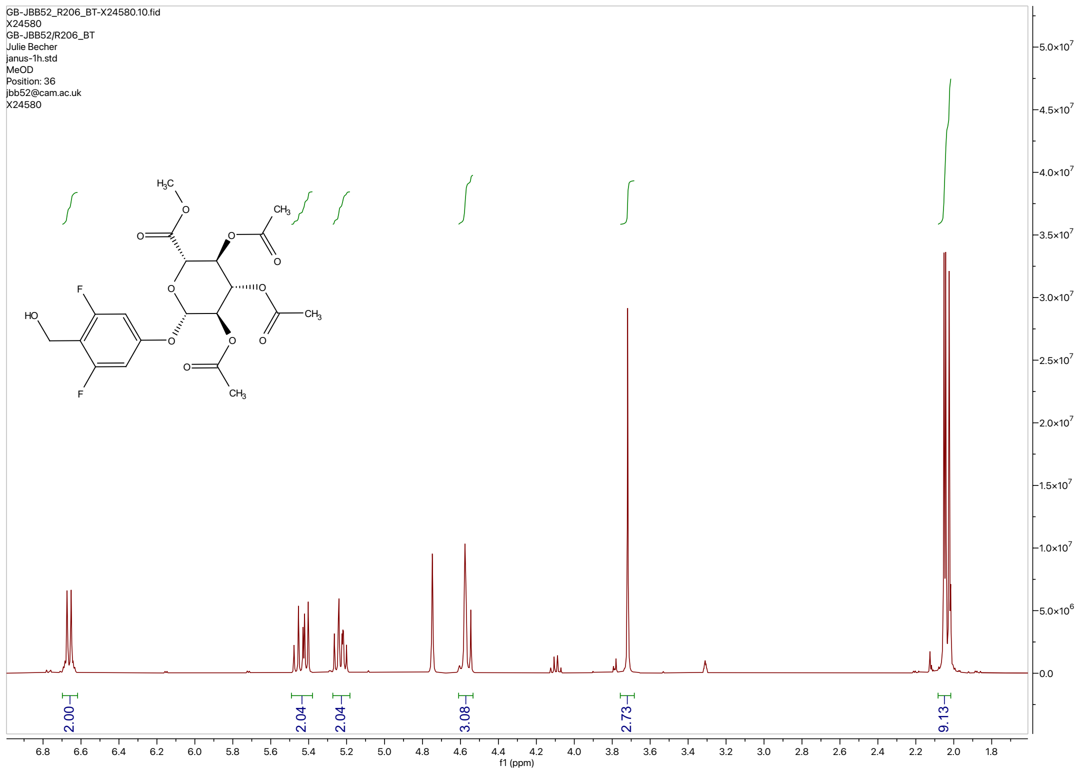

^{1}H NMR (400 MHz, MeOD) of 13d.

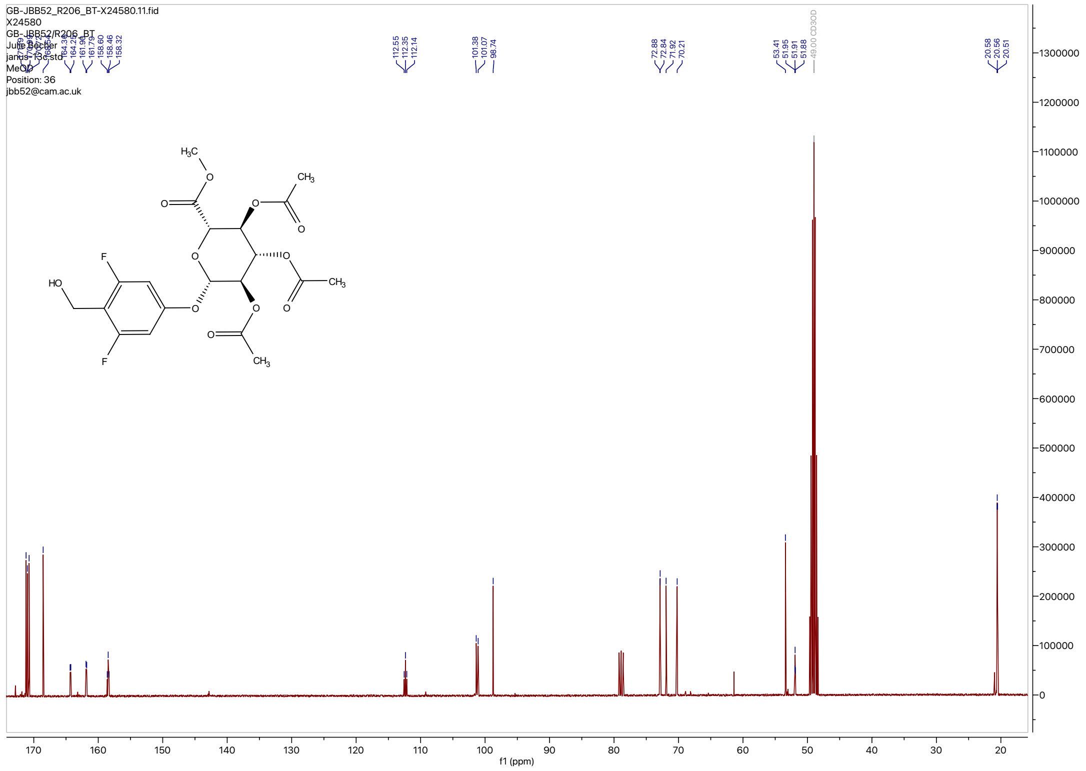

^{13}C NMR (101 MHz, MeOD) of 13d.

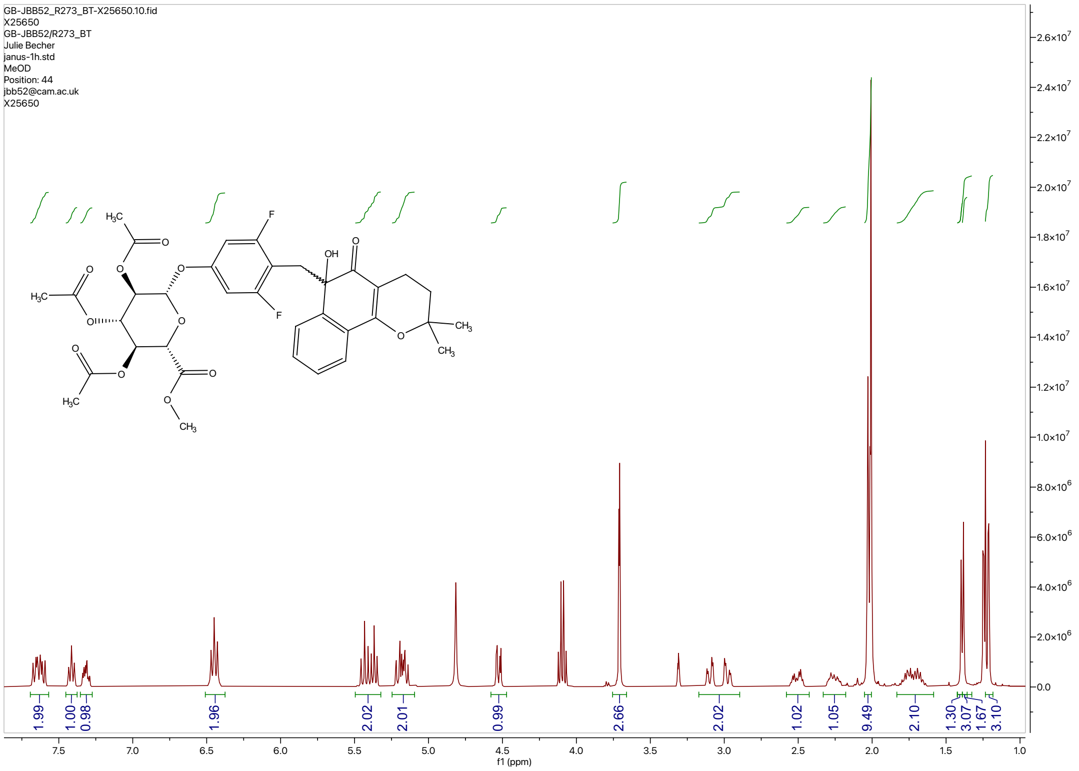

^{1}H NMR (400 MHz, MeOD, mixture of diastereomers 1:1.2) of 15d.

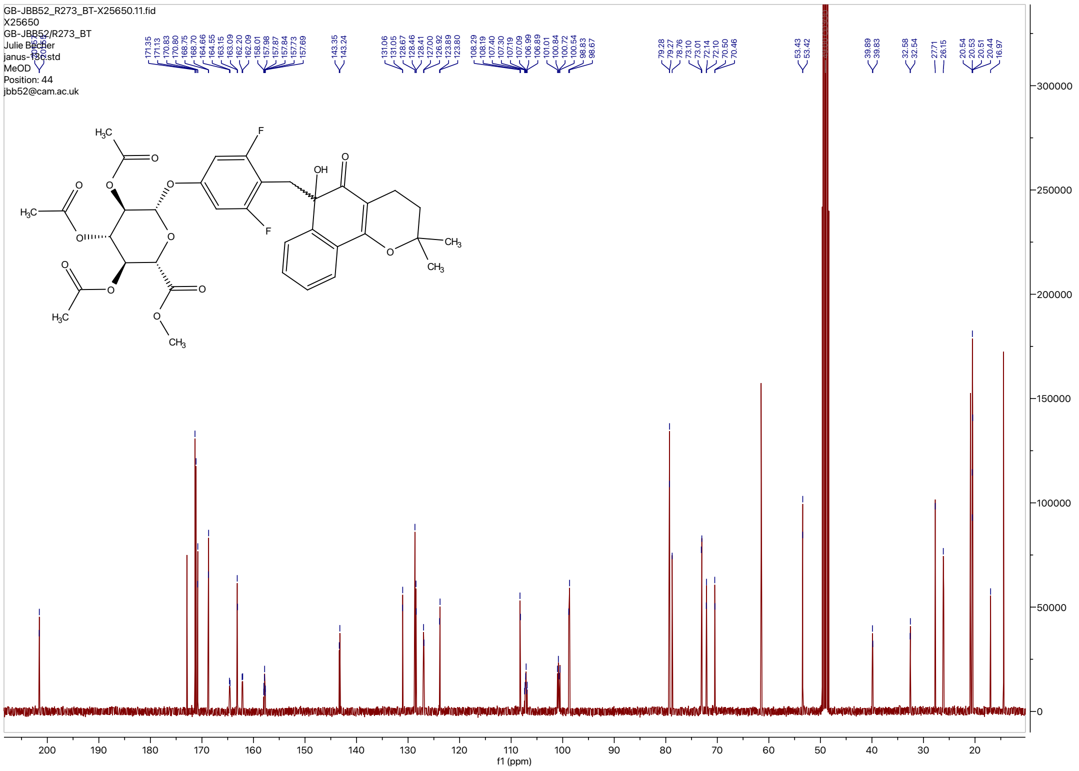

^{13}C NMR (101 MHz, MeOD, mixture of diastereomers 1:1.2) of 15d.

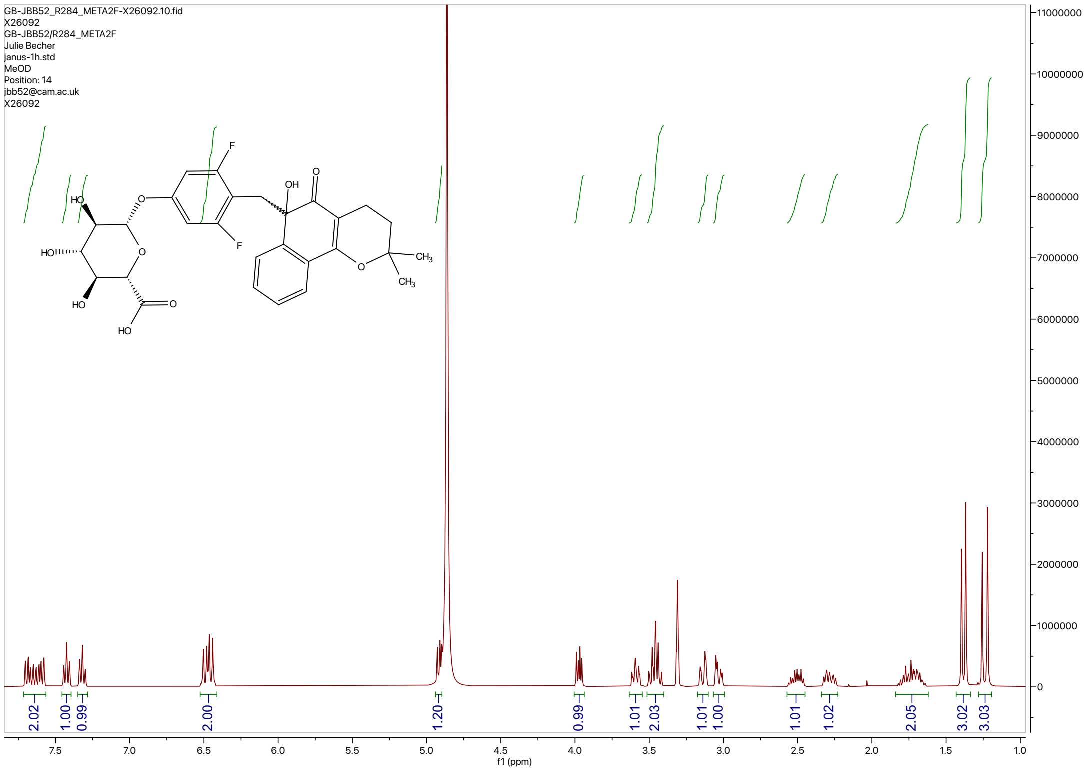

^{1}H NMR (400 MHz, MeOD, mixture of diastereomers 1:1.2) of 16d.

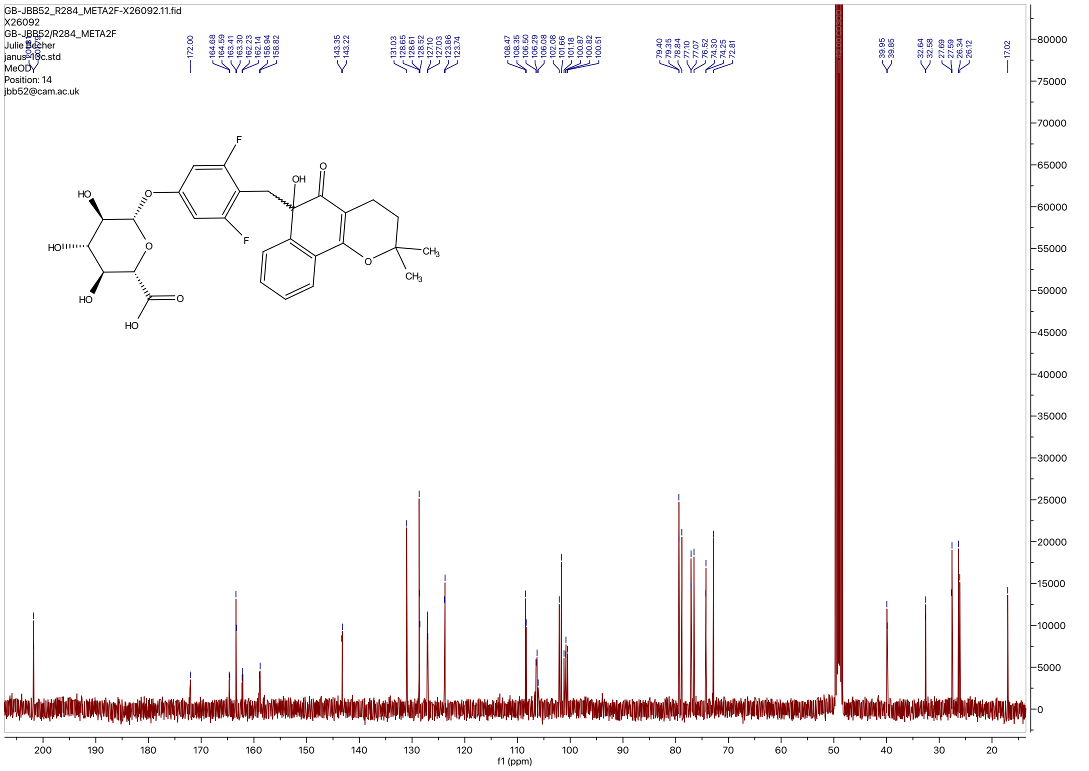

^{13}C NMR (101 MHz, MeOD, mixture of diastereomers 1:1.2) of 16d.

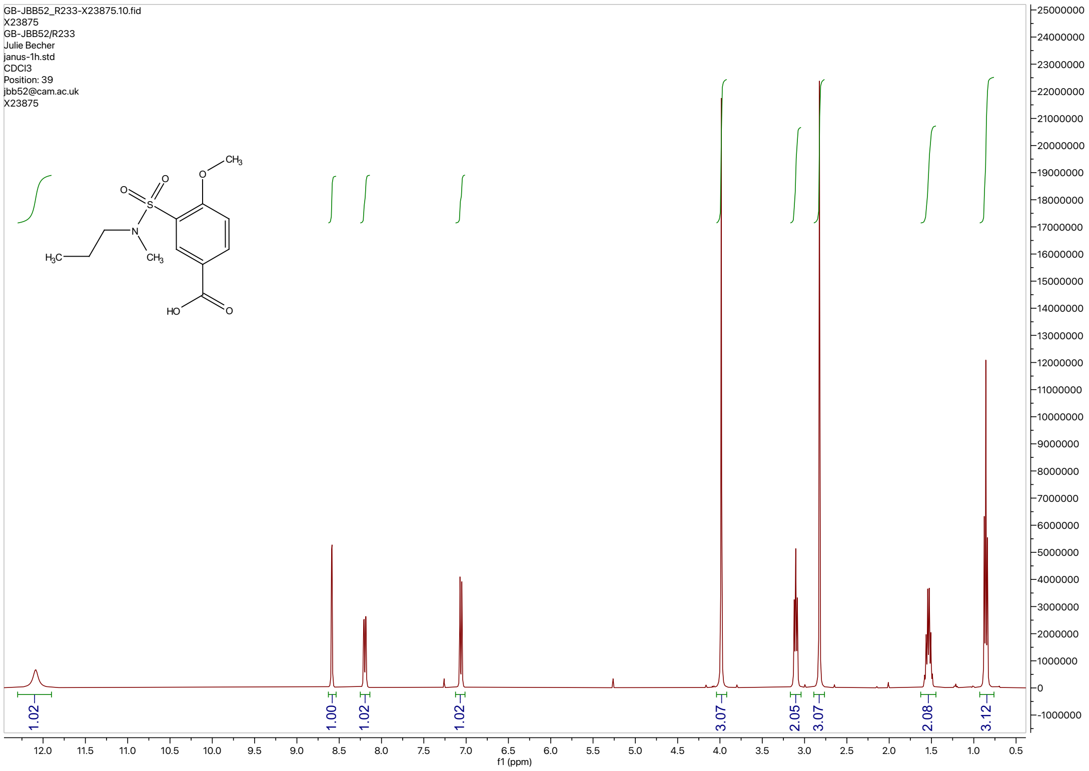

^{1}H NMR (400 MHz, CDCl$_3$) of 8.

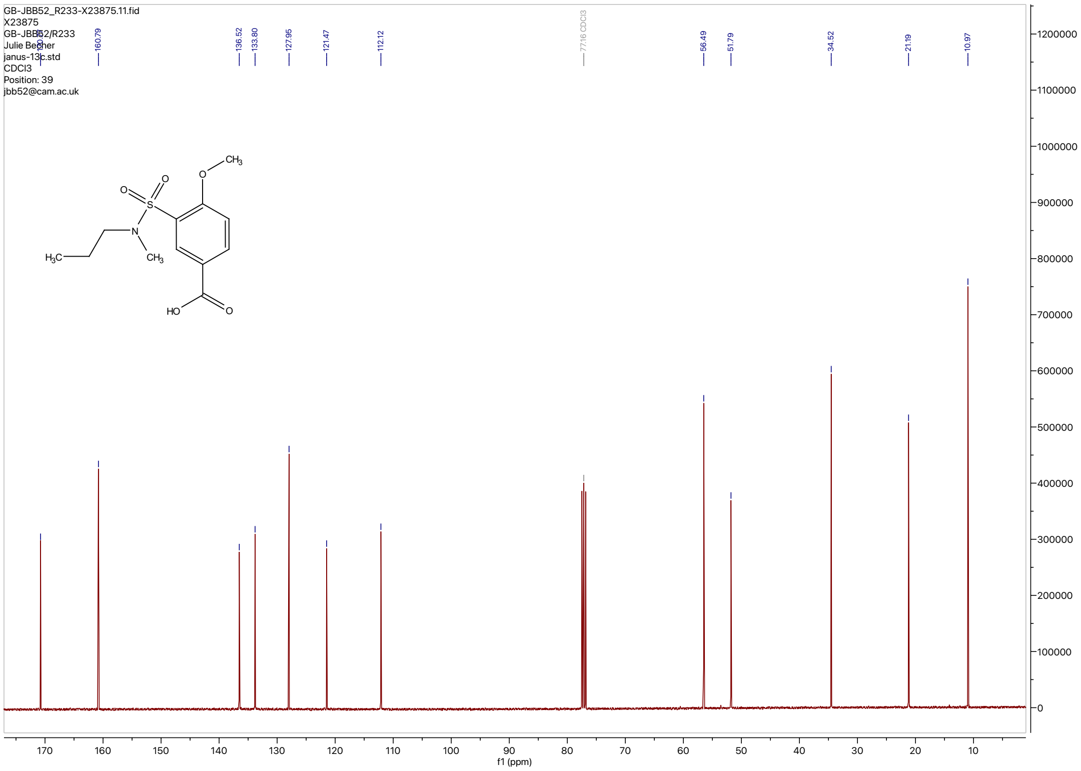

^{13}C NMR (101 MHz, CDCl$_3$) of 8.

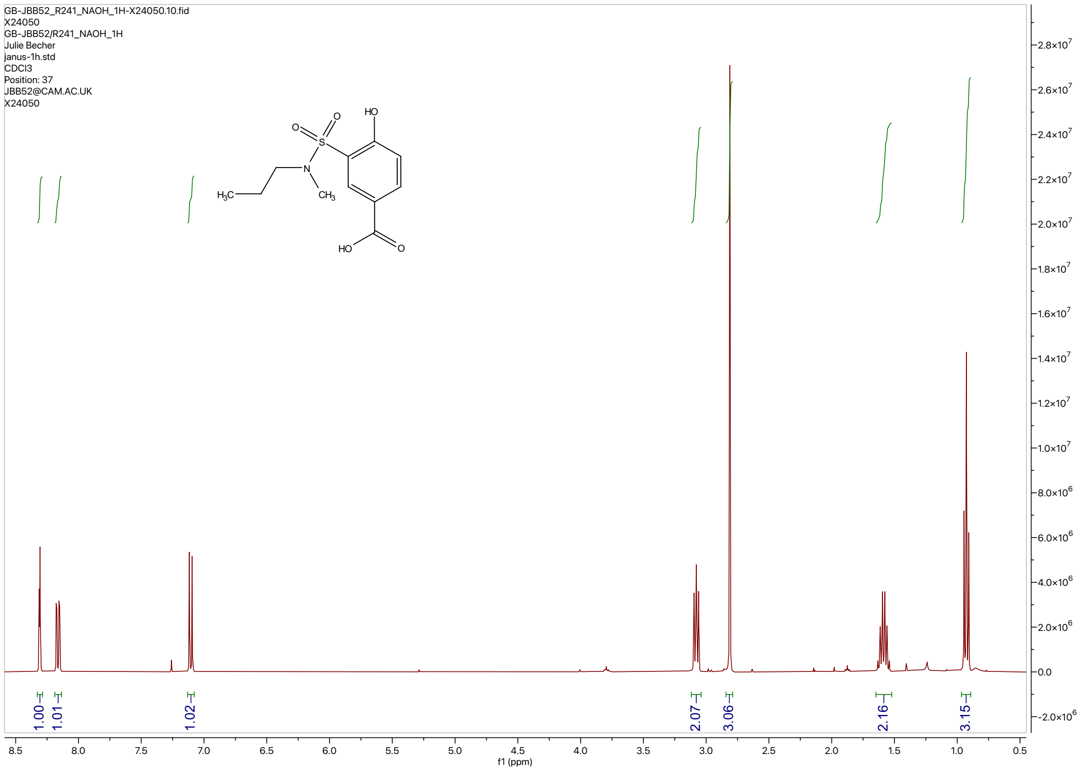

^{1}H NMR (400 MHz, $CDCl_3$) of 9.

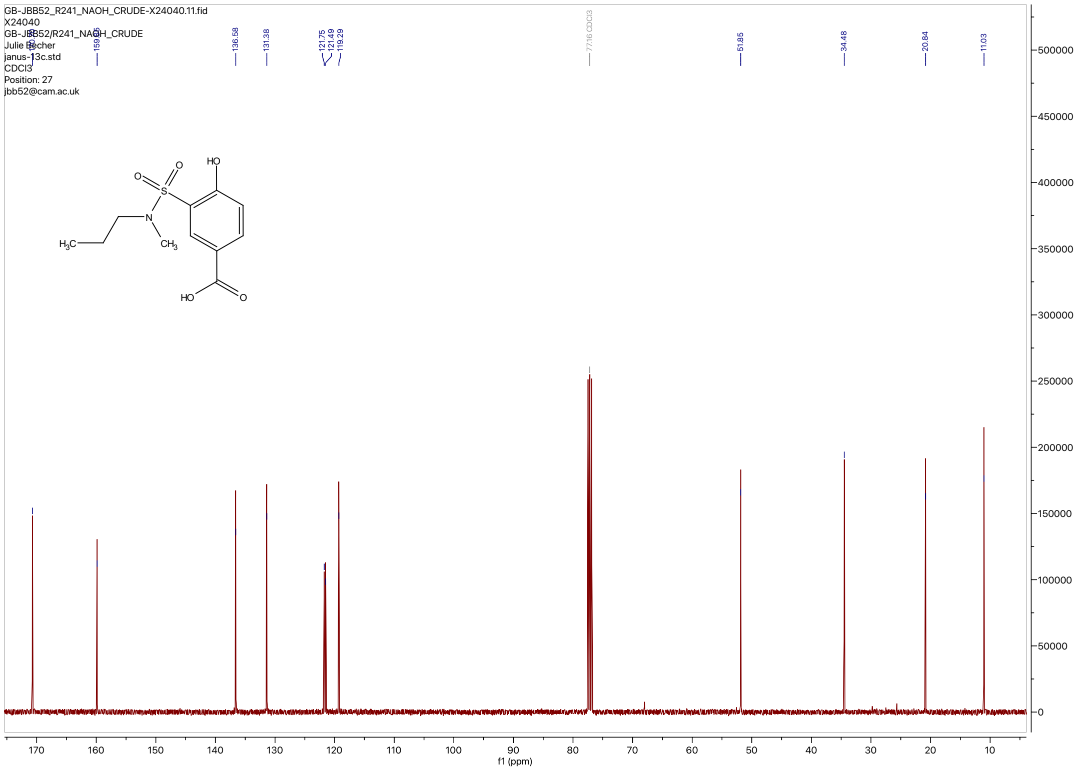

^{13}C NMR (101 MHz, $CDCl_3$) of 9.

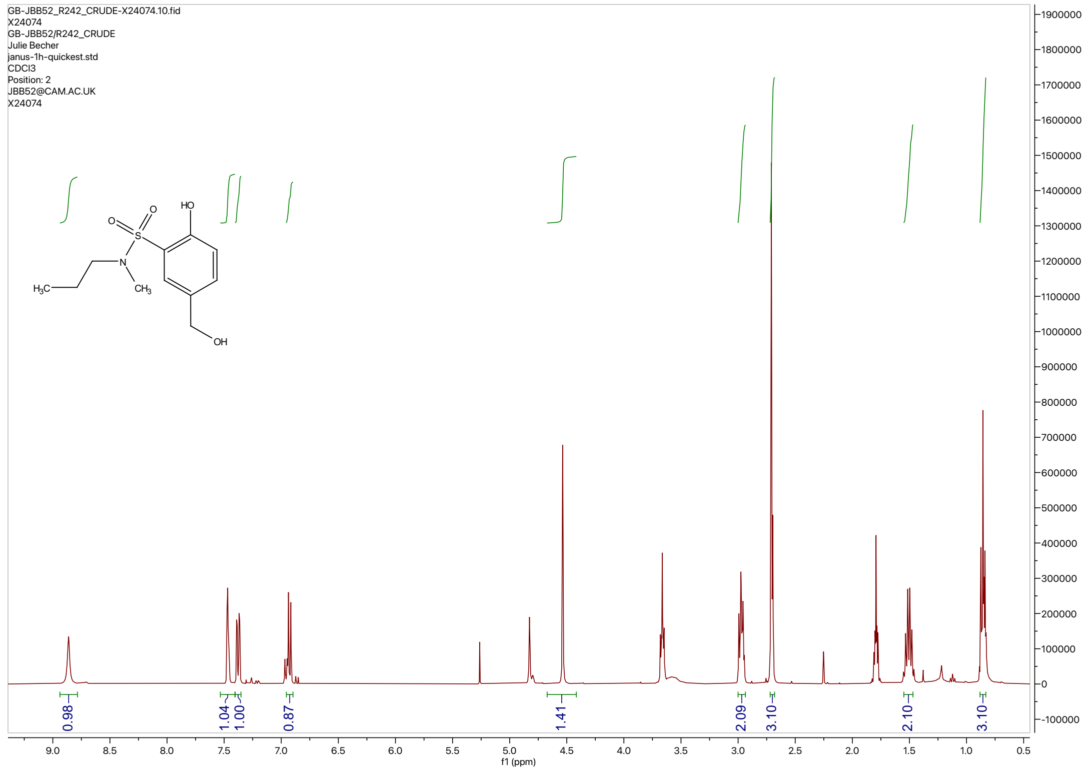

^{1}H NMR (400 MHz, CDCl$_3$) of 10.

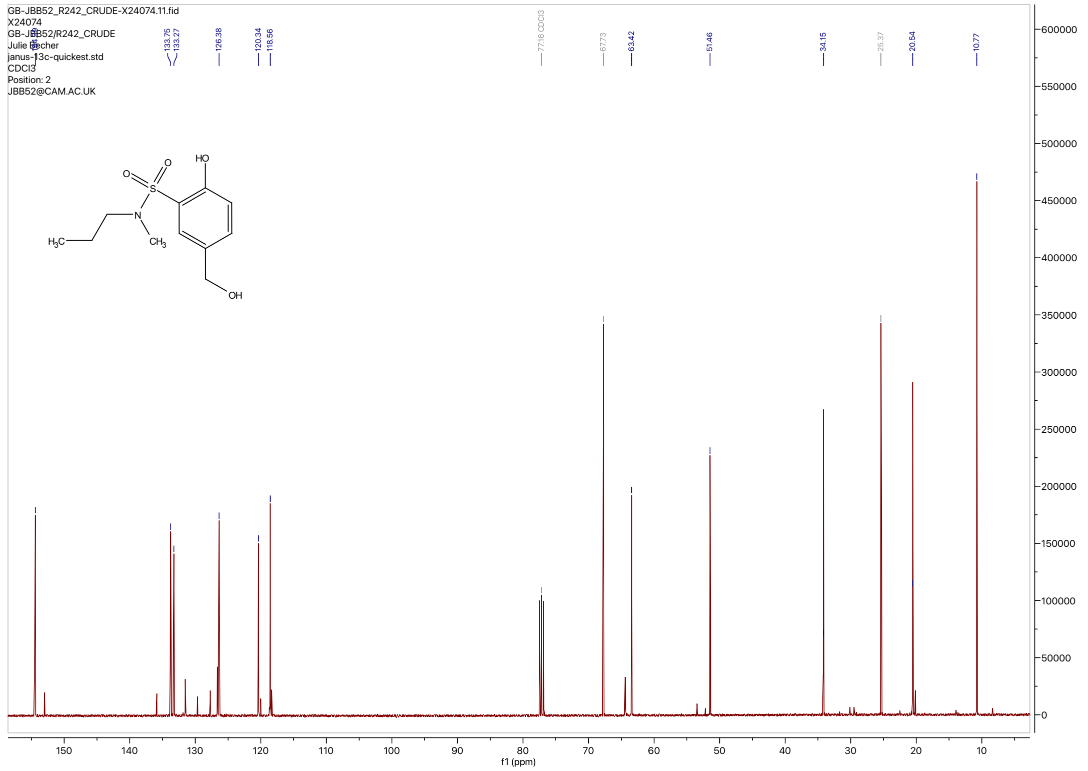

^{13}C NMR (101 MHz, CDCl$_3$) of 10.

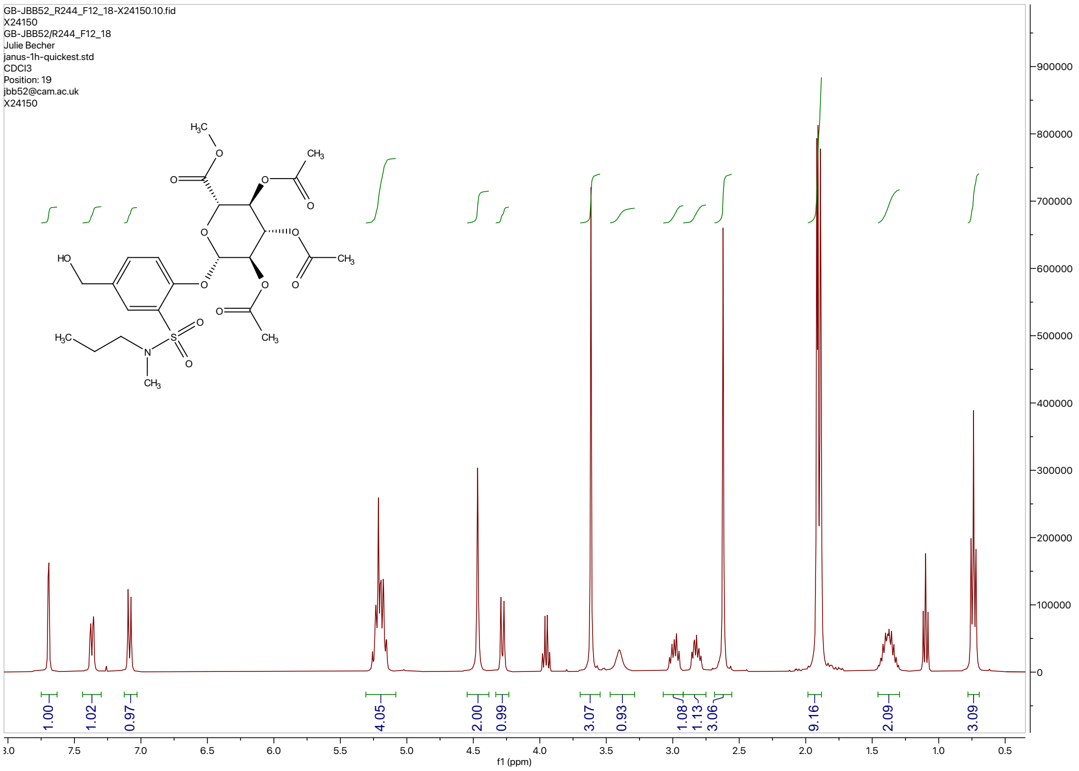

^{1}H NMR (400 MHz, $CDCl_3$) of 13e.

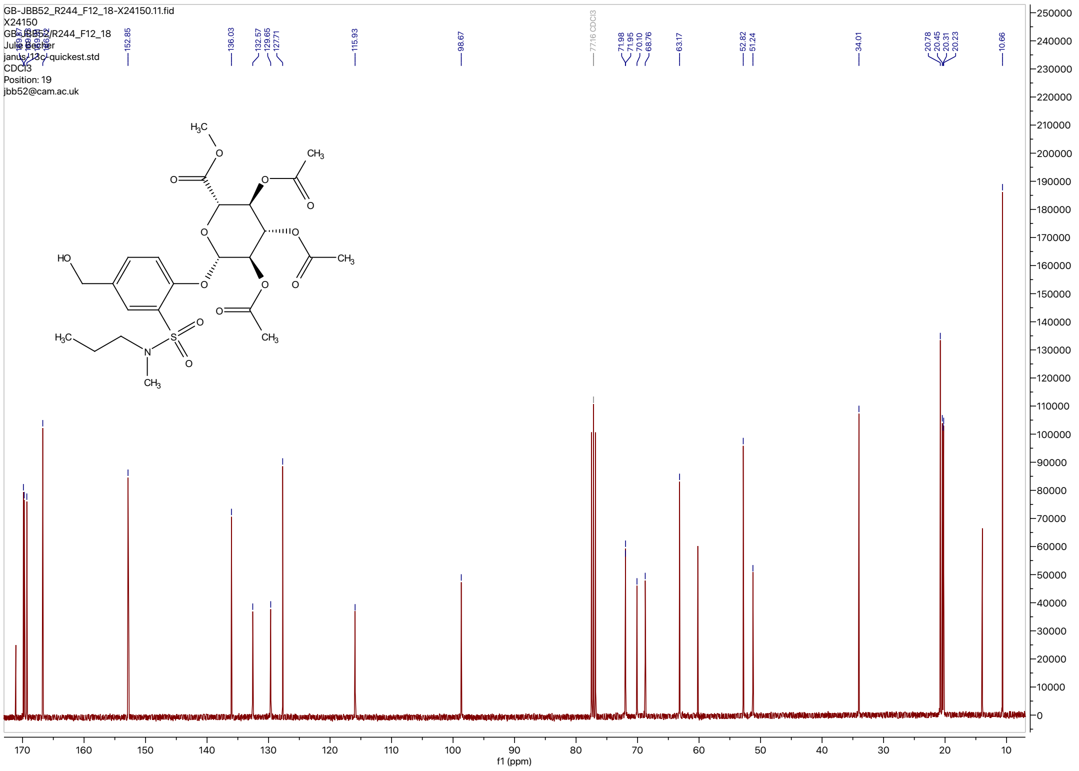

^{13}C NMR (101 MHz, $CDCl_3$) of 13e.

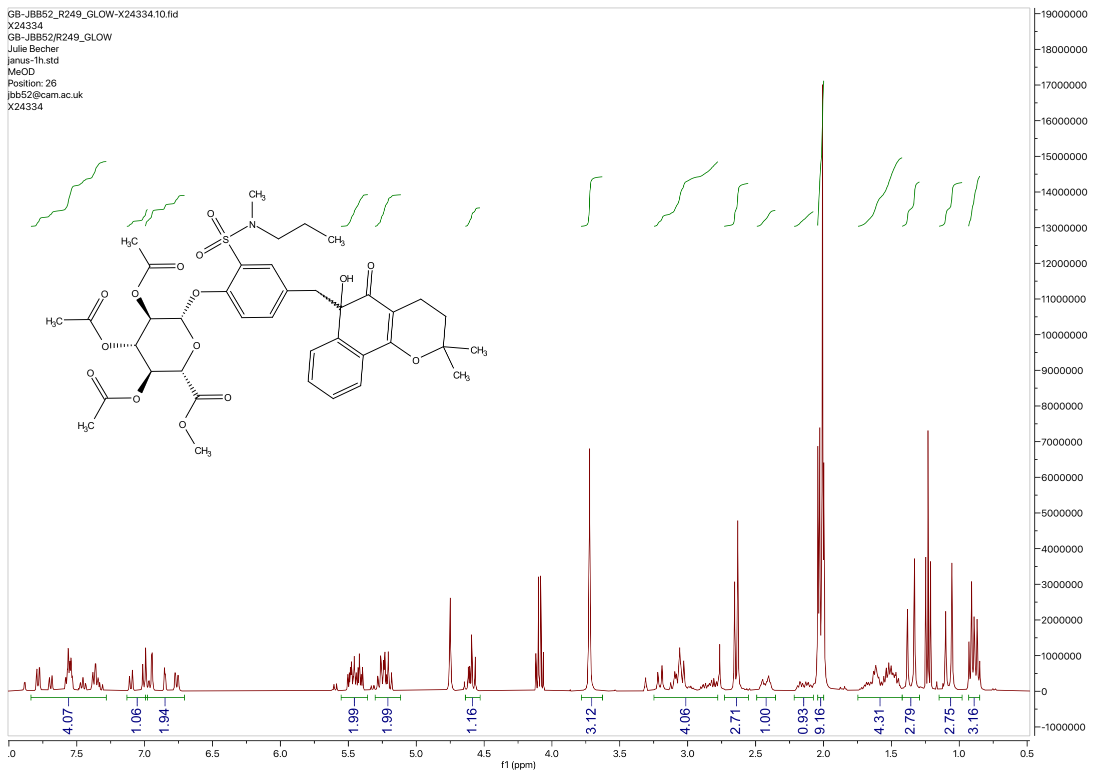

^{1}H NMR (400 MHz, MeOD, mixture of diastereomers 1:1.6) of 15e.

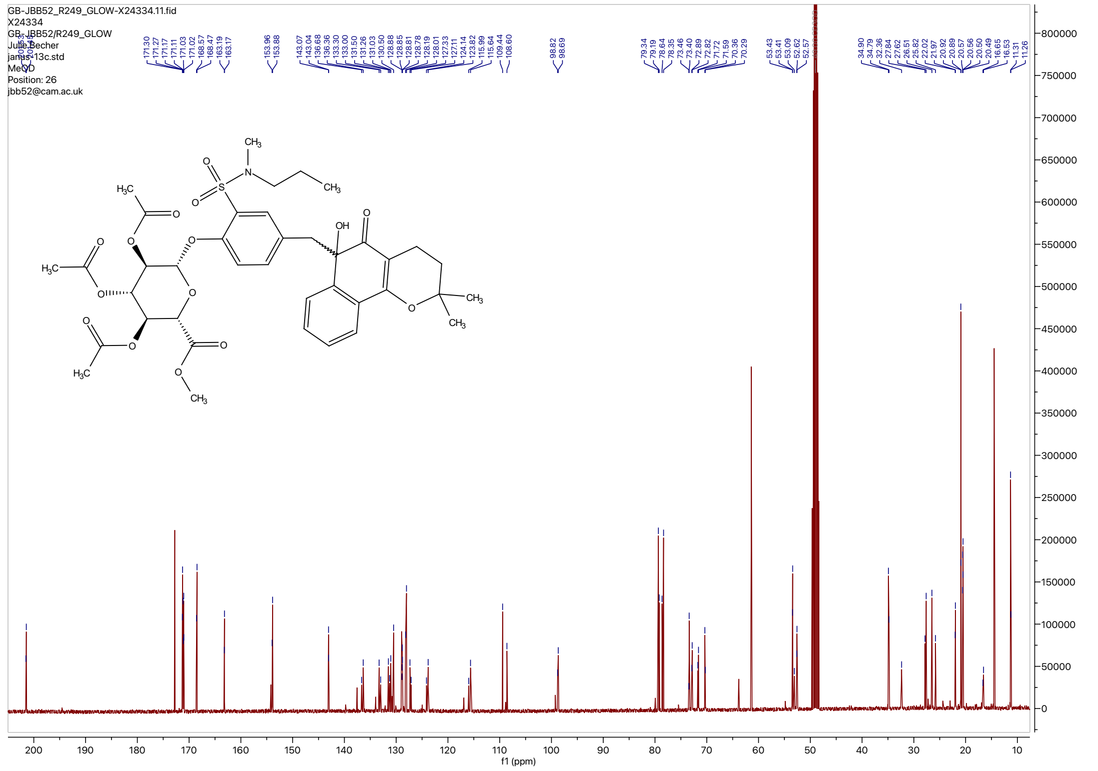

^{13}C NMR (101 MHz, MeOD, mixture of diastereomers 1:1.6) of 15e.

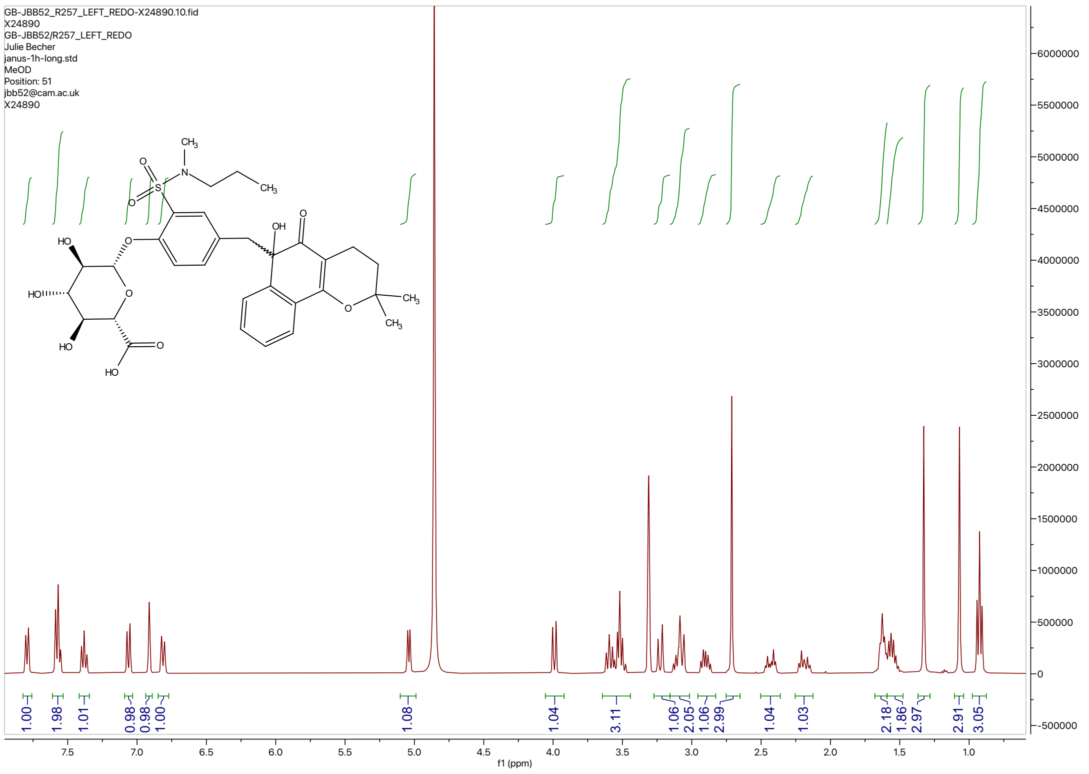

^{1}H NMR (400 MHz, MeOD) of 16e Major Product.

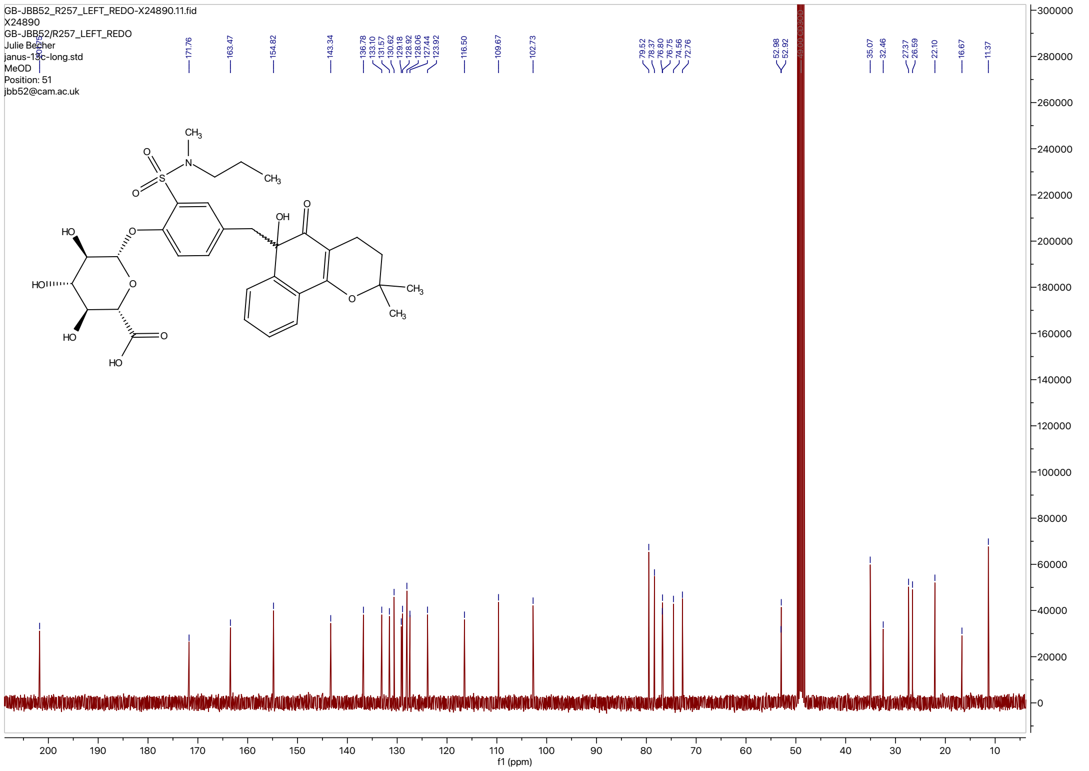

^{13}C NMR (101 MHz, MeOD) of 16e Major Product.

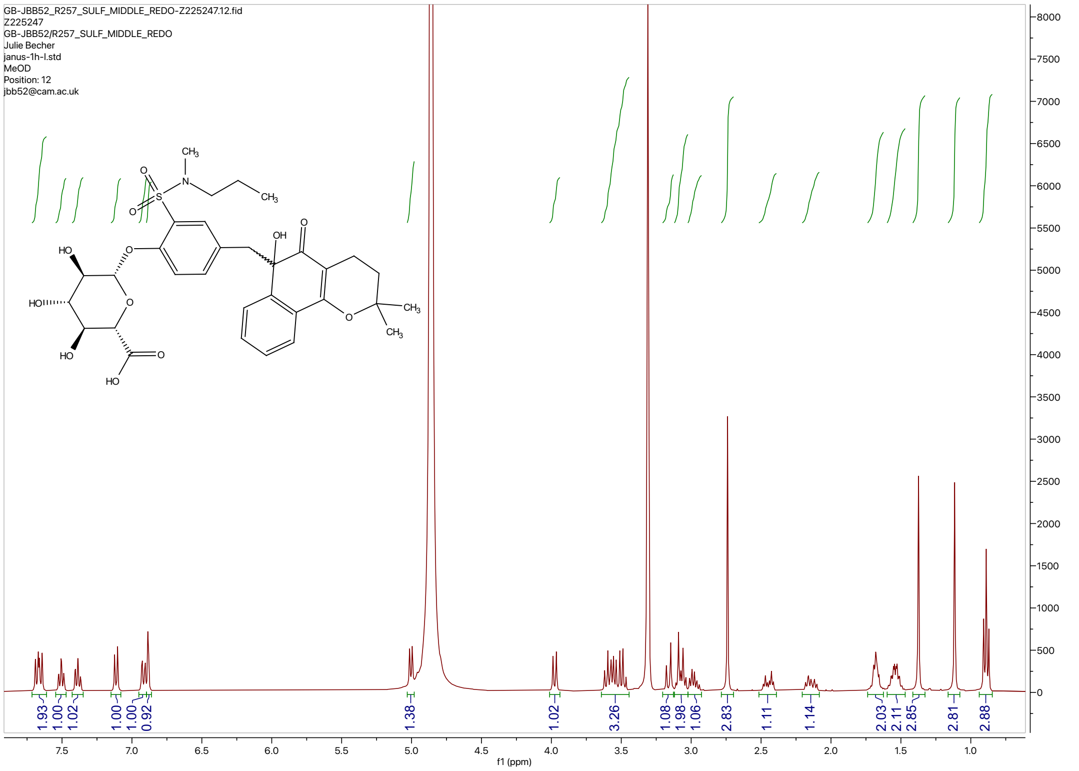

^{1}H NMR (400 MHz, MeOD) of 16e Minor Product.

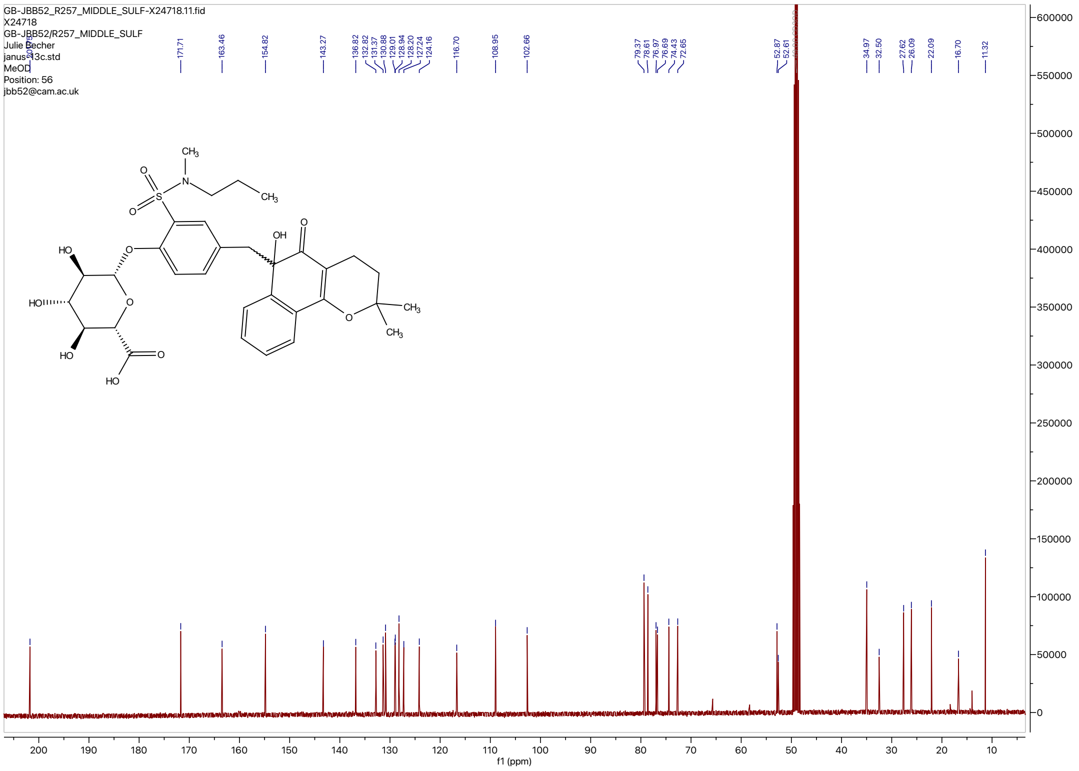

^{13}C NMR (101 MHz, MeOD) of 16e Minor Product.

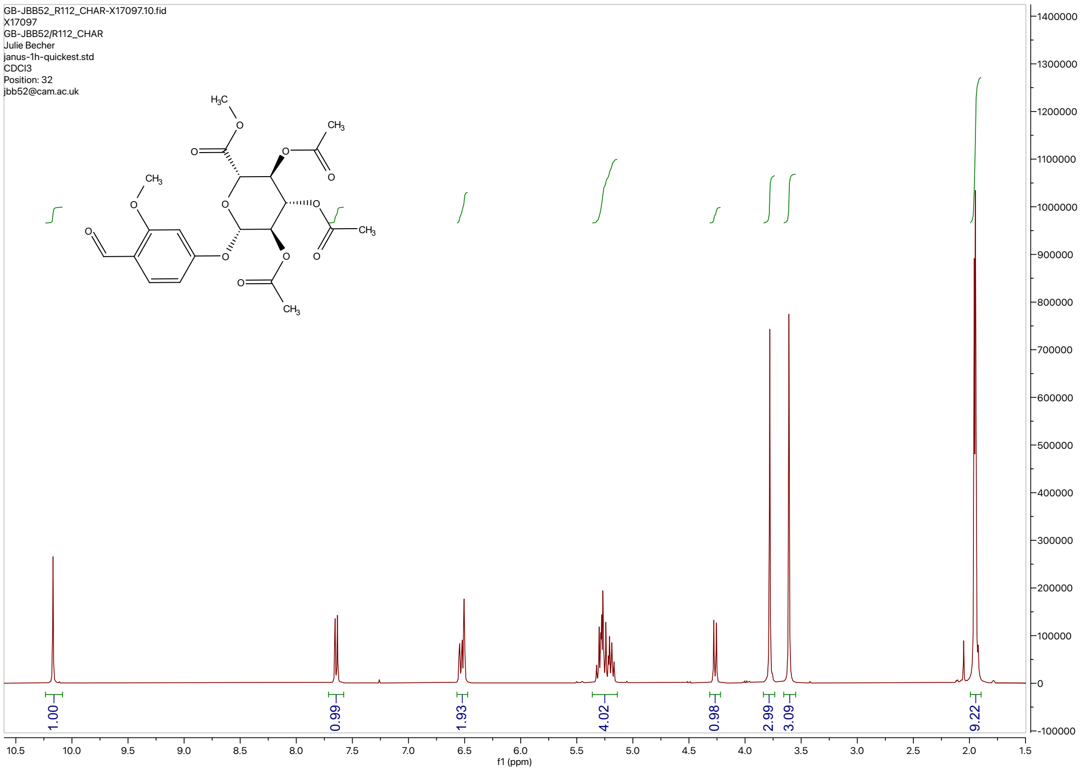

^{1}H NMR (400 MHz, $CDCl_3$) of 12f.

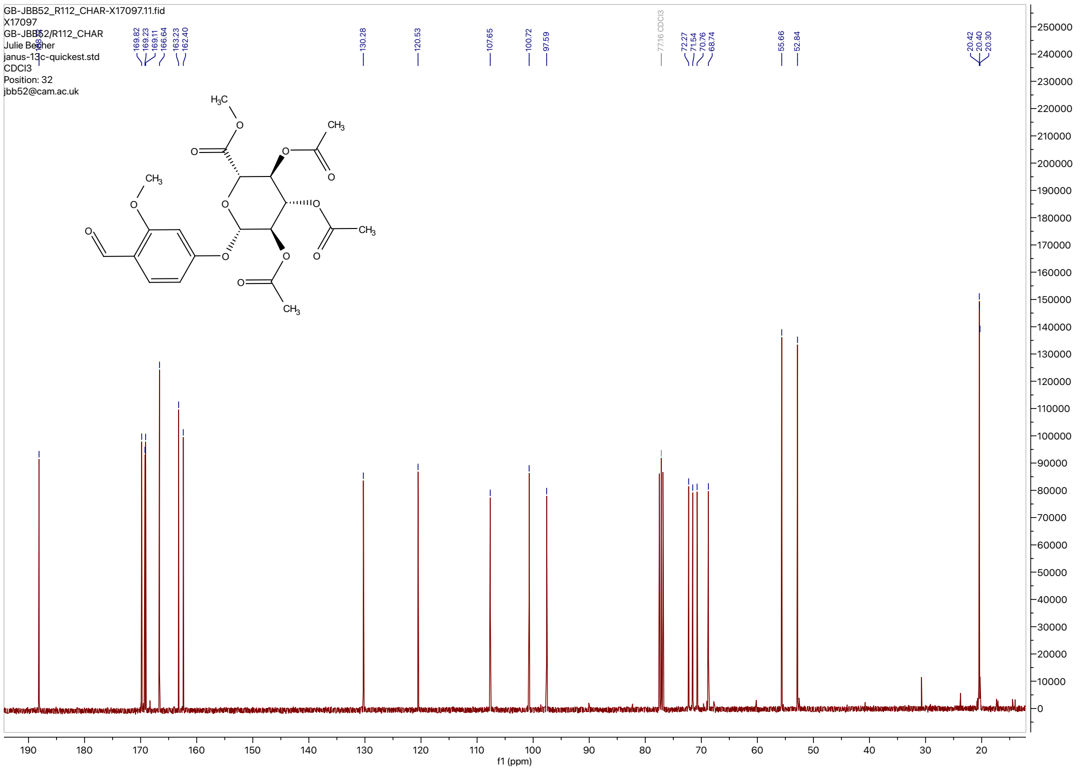

^{13}C NMR (101 MHz, $CDCl_3$) of 12f.

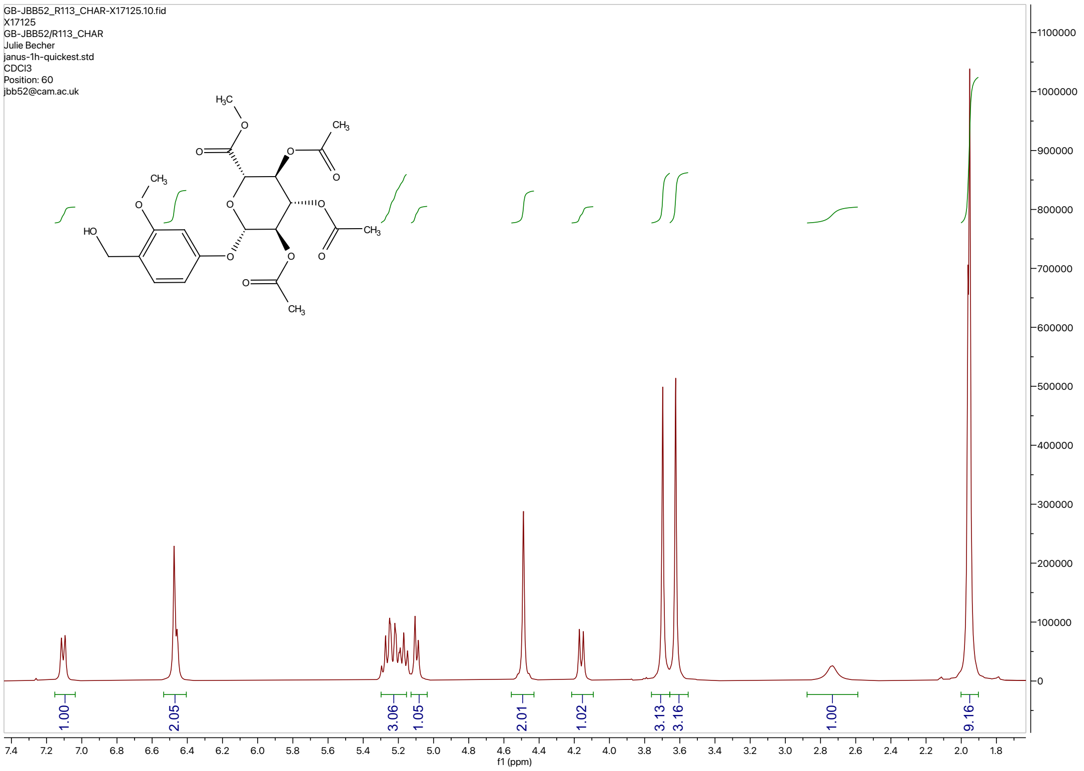

^{1}H NMR (400 MHz, CDCl$_3$) of 13f.

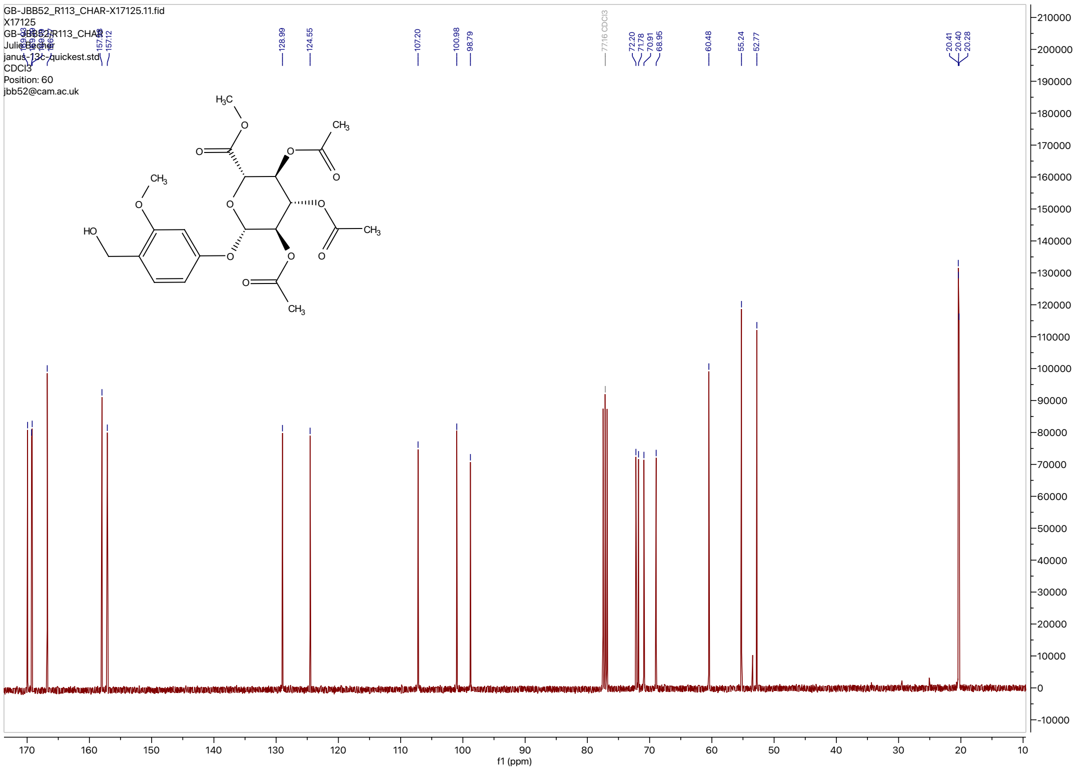

^{13}C NMR (101 MHz, CDCl$_3$) of 13f.

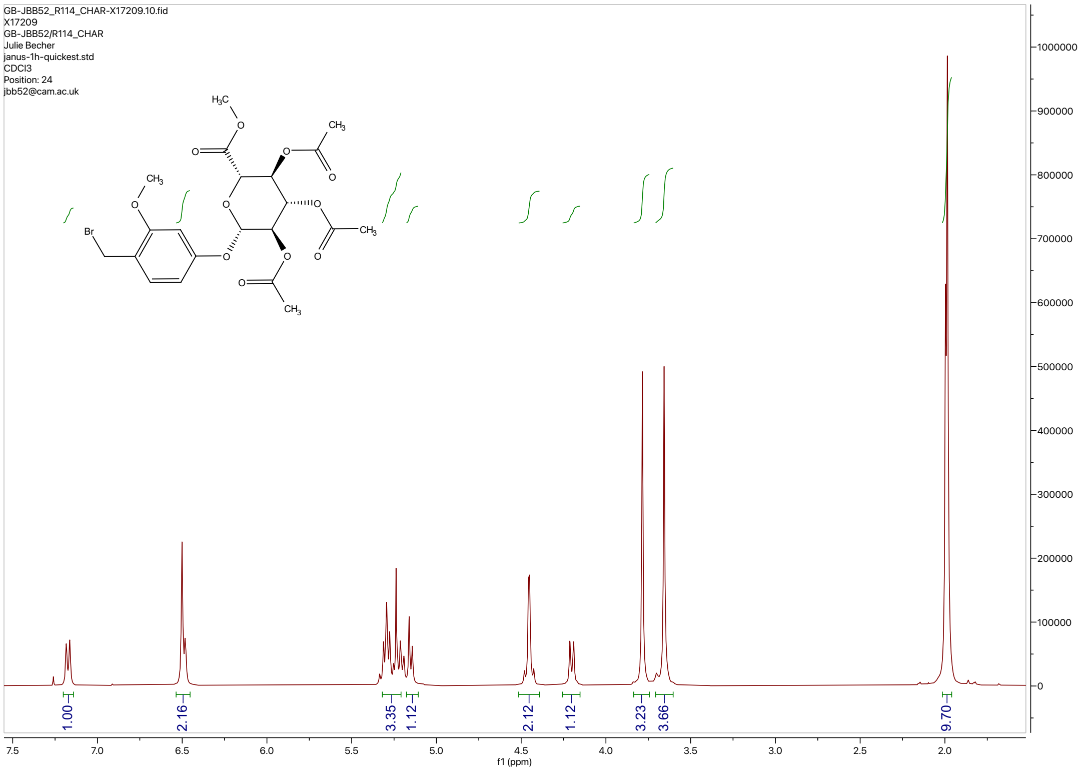

^{1}H NMR (400 MHz, $CDCl_3$) of 14f.

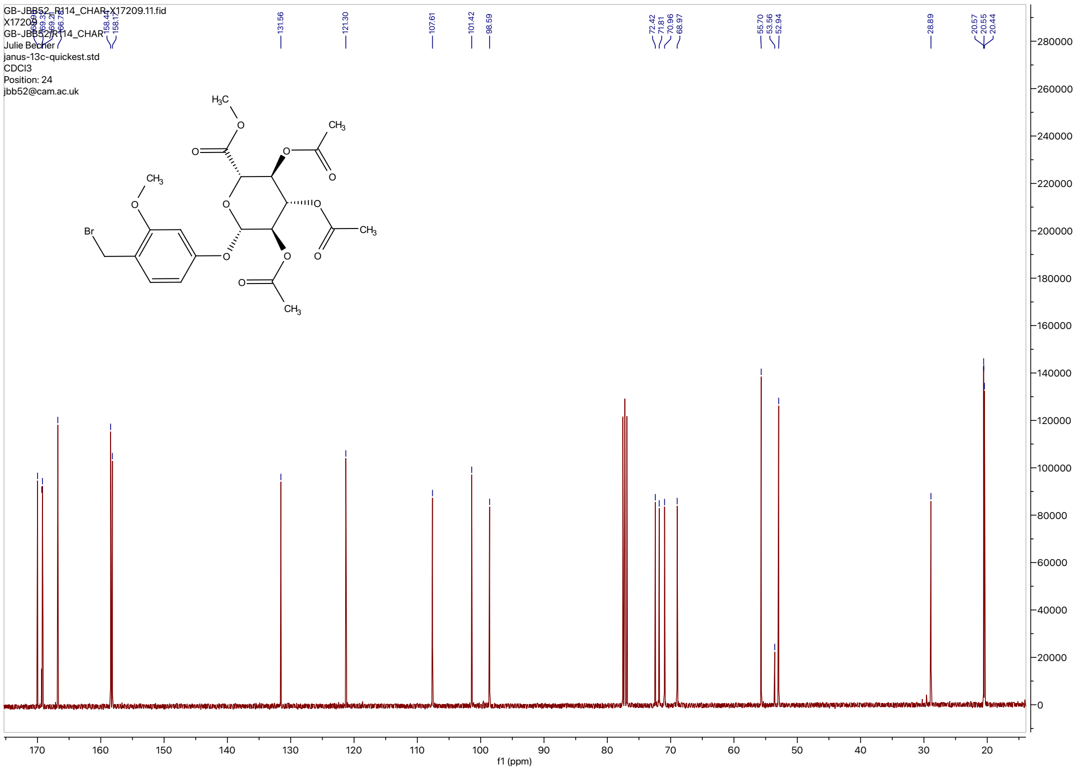

^{13}C NMR (101 MHz, $CDCl_3$) of 14f.

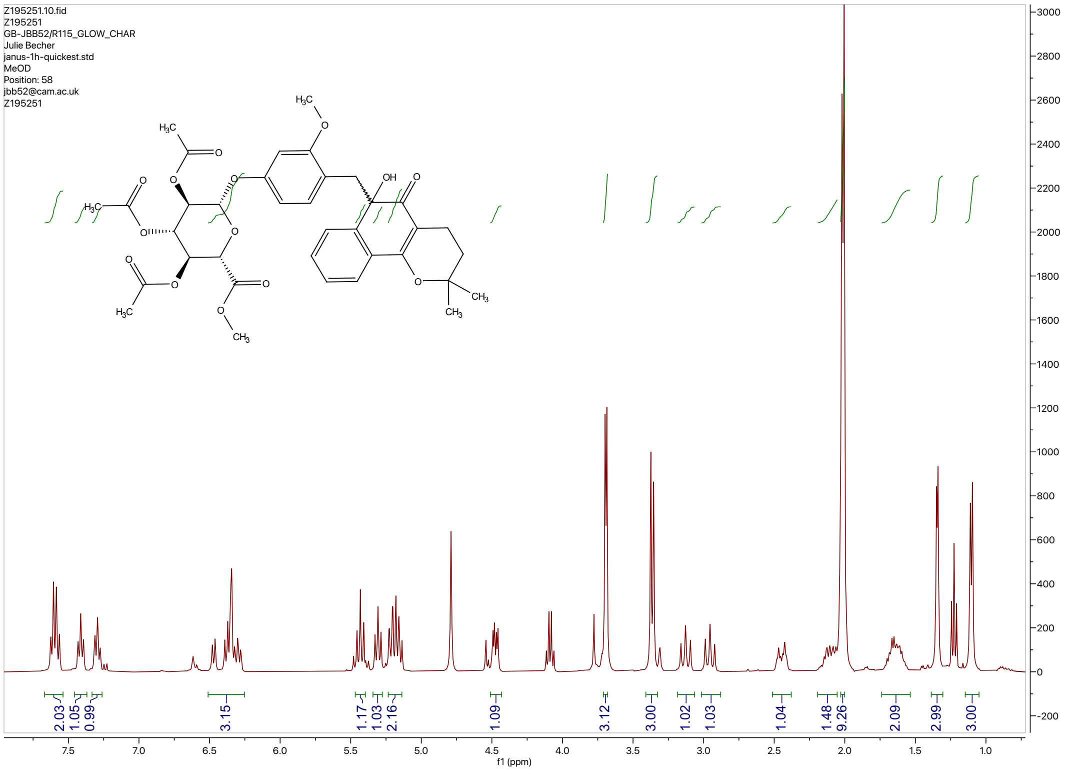

^{1}H NMR (400 MHz, MeOD, mixture of diastereomers 1:1) of 15f.

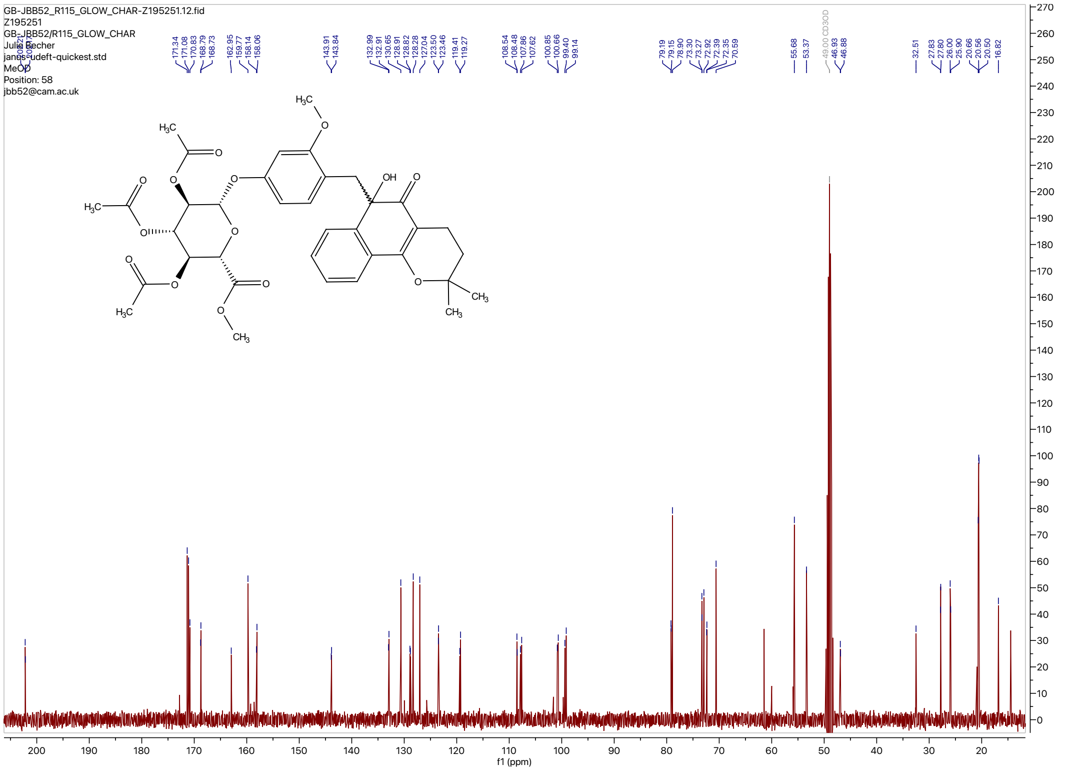

^{13}C NMR (101 MHz, MeOD, mixture of diastereomers 1:1) of 15f.

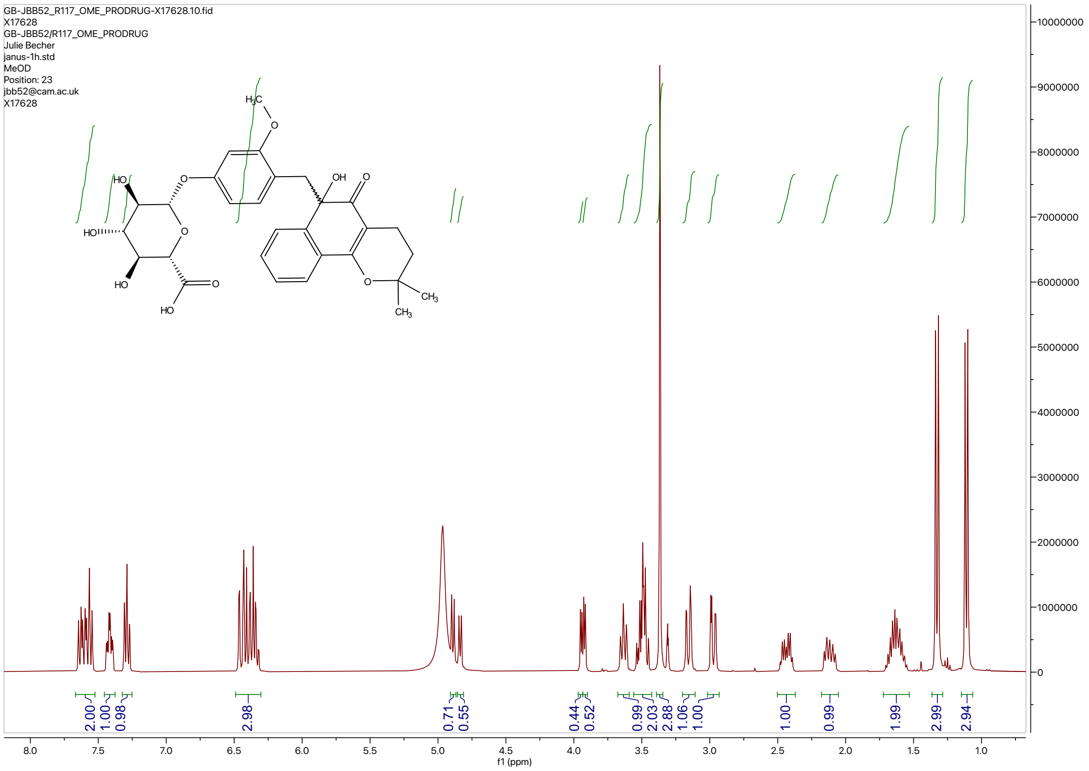

^{1}H NMR (400 MHz, MeOD, mixture of diastereomers 1:1) of 16f.

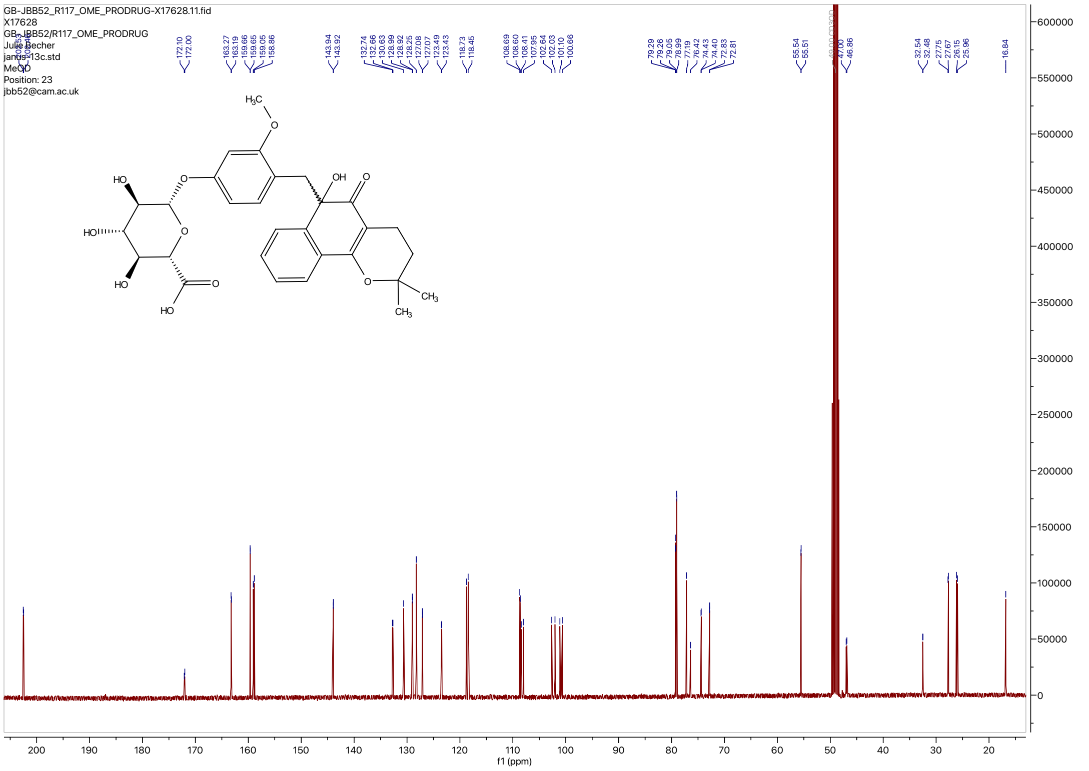

^{13}C NMR (101 MHz, MeOD, mixture of diastereomers 1:1) of 16f.

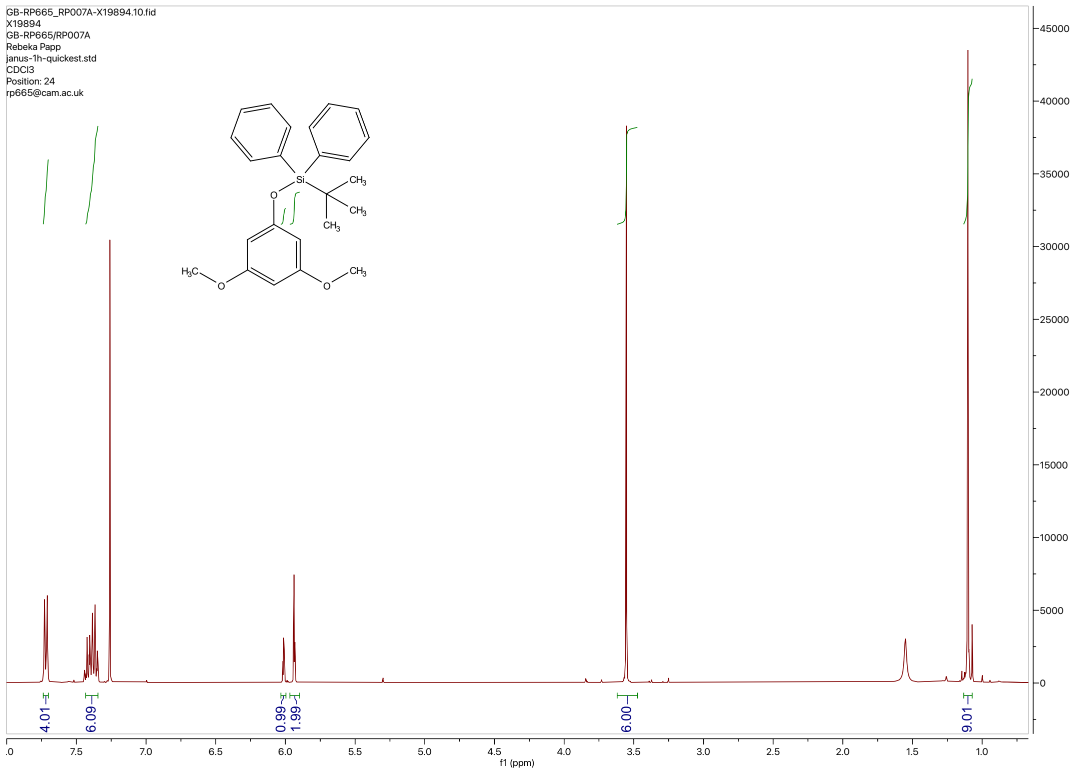

^{1}H NMR (400 MHz, $CDCl_3$) of S5.

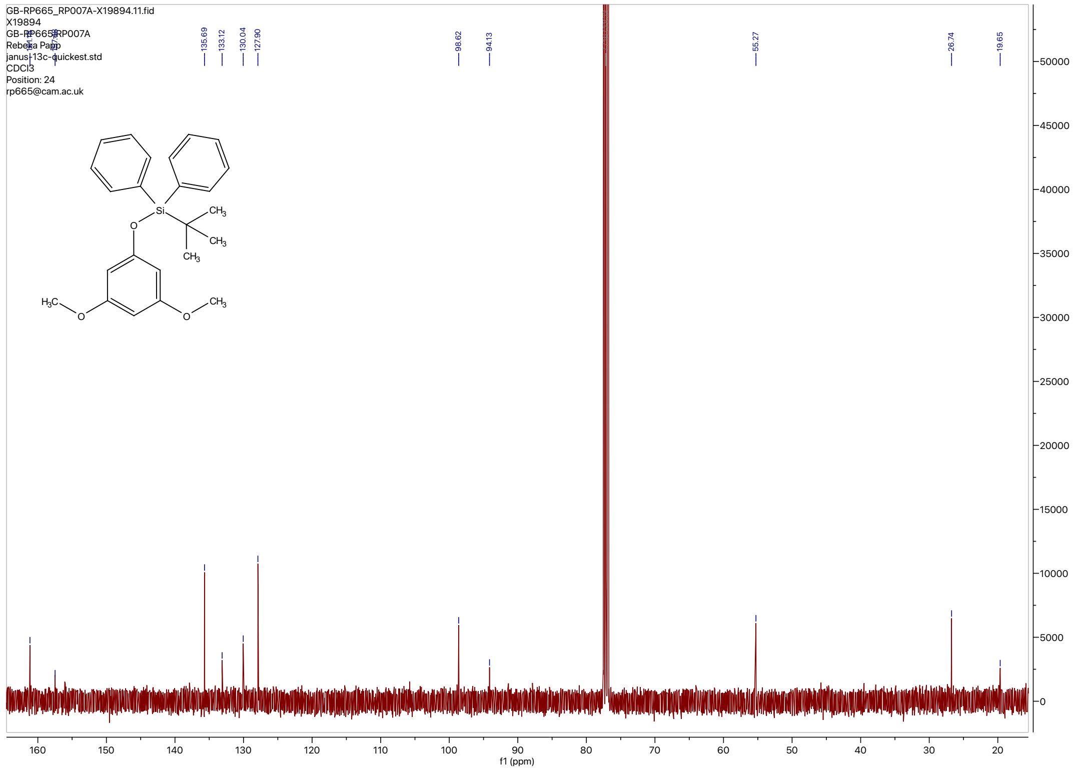

^{13}C NMR (101 MHz, $CDCl_3$) of S5.

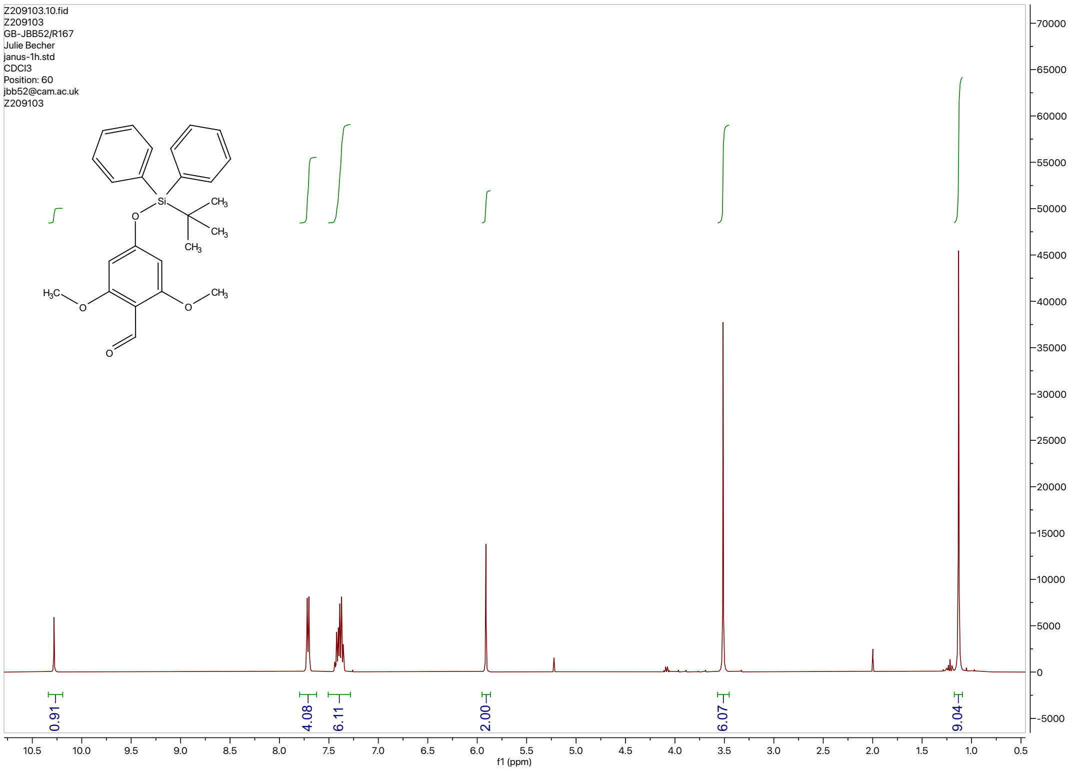

^{1}H NMR (400 MHz, $CDCl_3$) of S6.

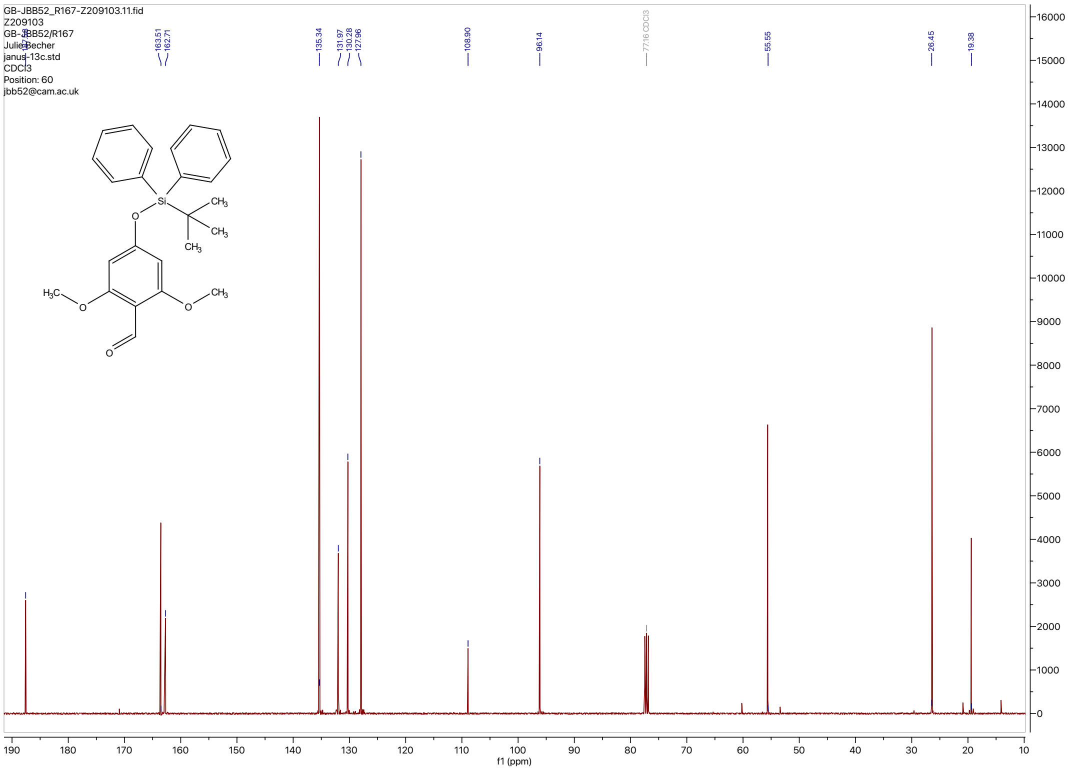

^{13}C NMR (101 MHz, $CDCl_3$) of S6.

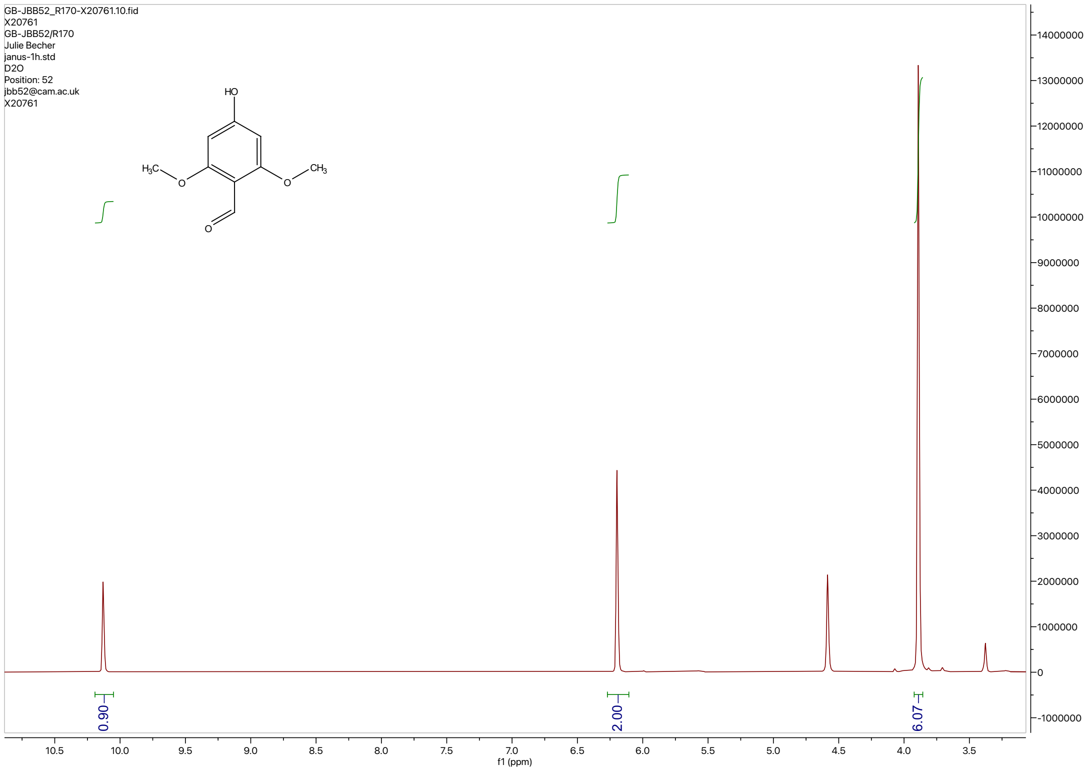

^{1}H NMR (400 MHz, D_2O) of 11g.

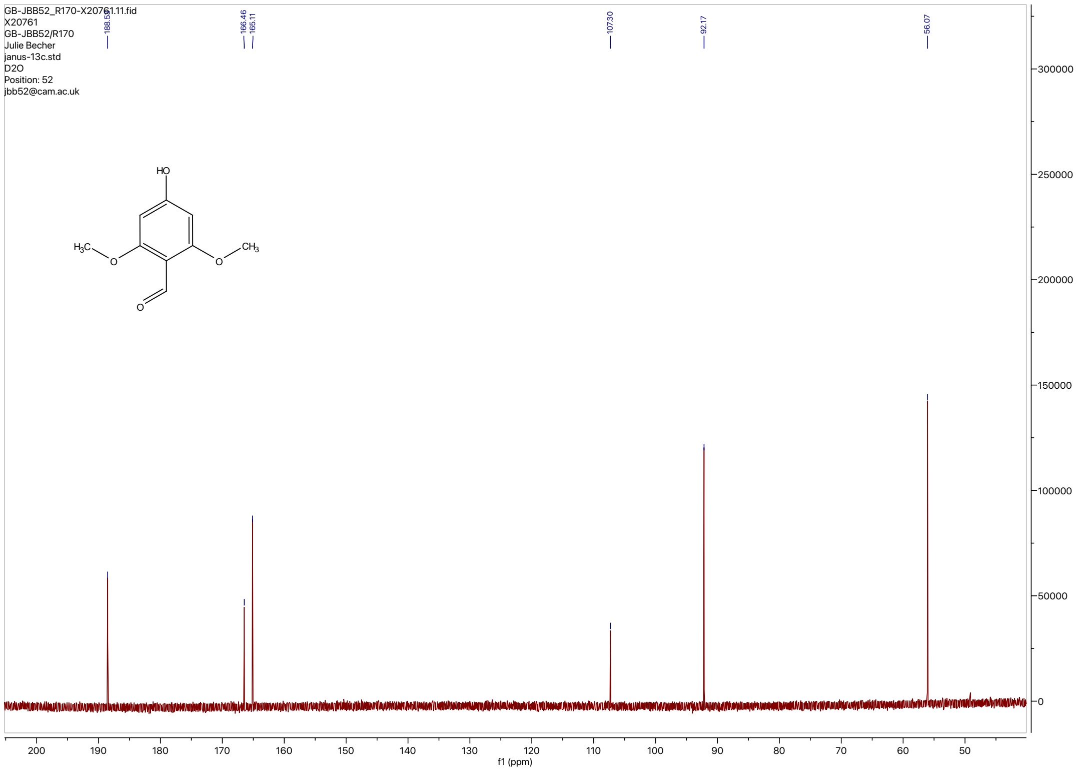

^{13}C NMR (101 MHz, D_2O) of 11g.

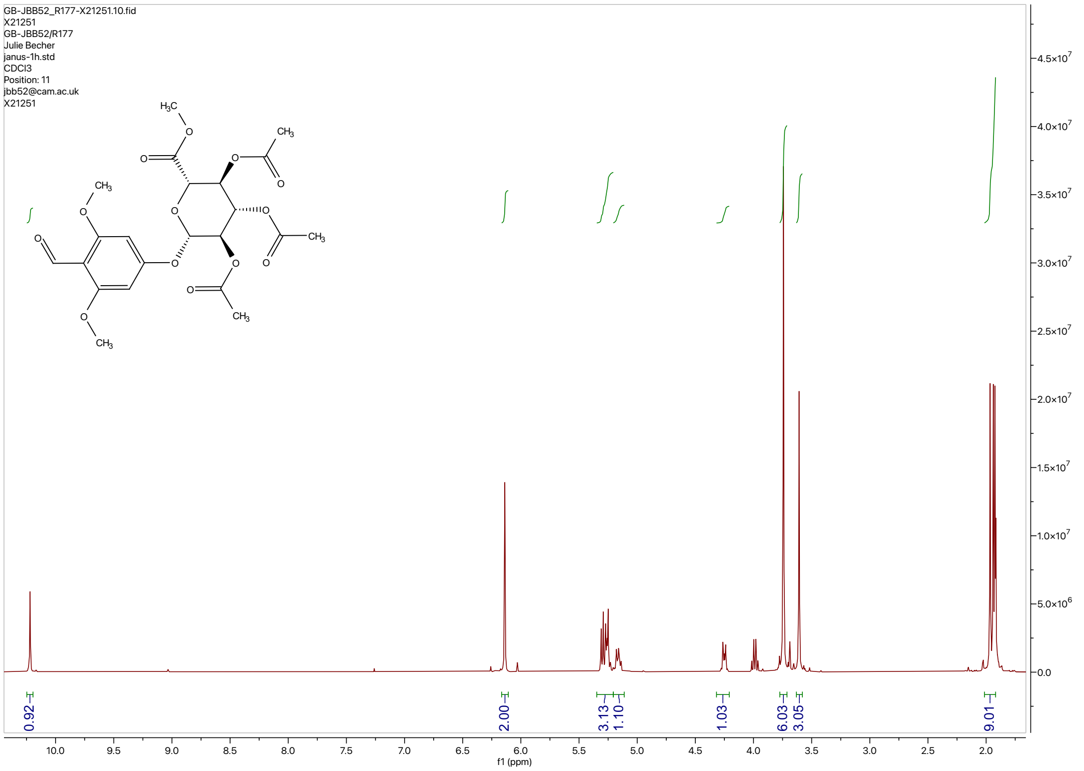

^{1}H NMR (400 MHz, $CDCl_3$) of 12g.

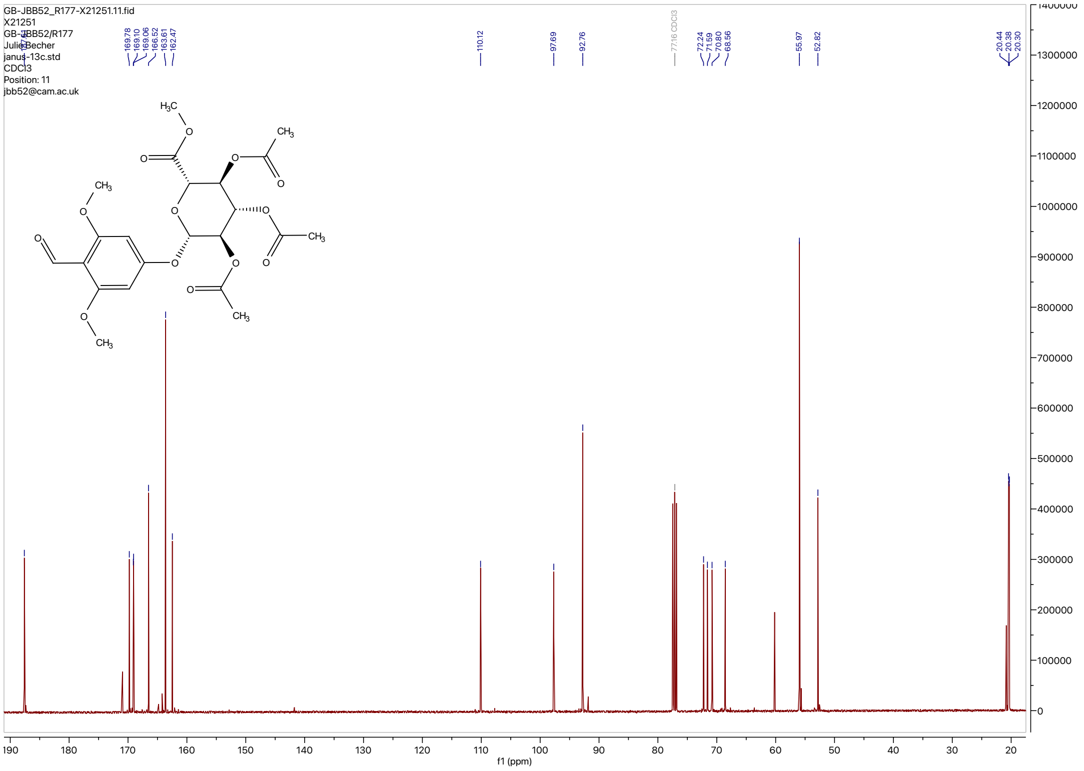

^{13}C NMR (101 MHz, $CDCl_3$) of 12g.

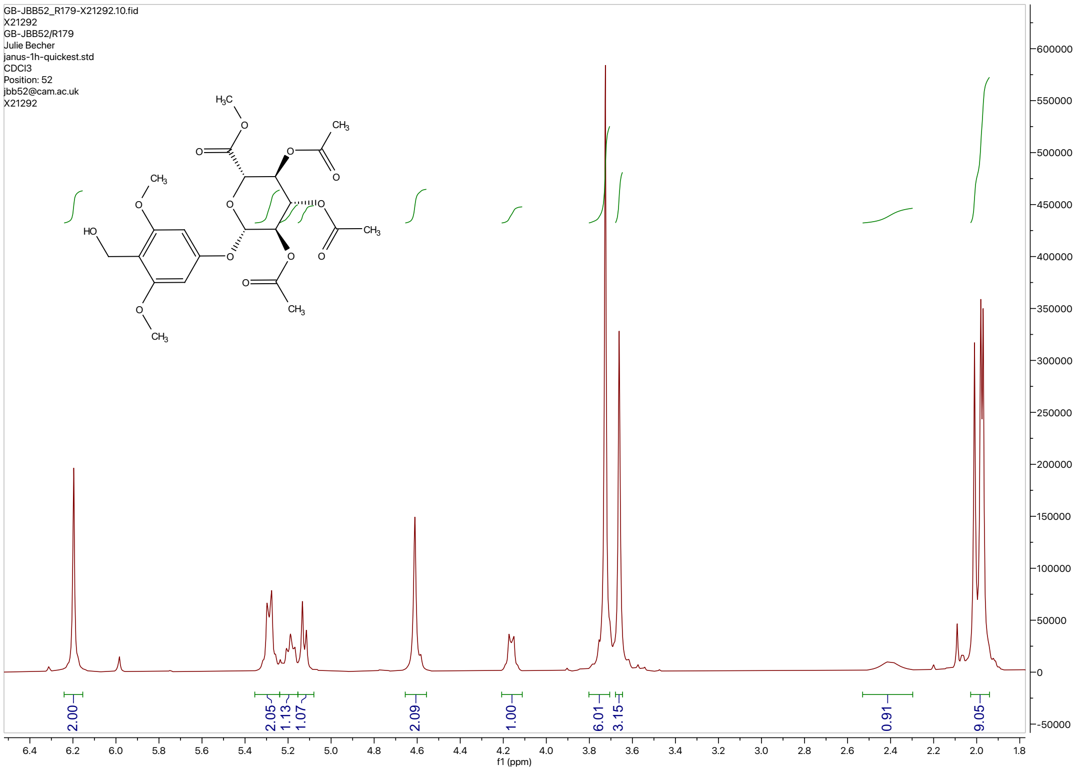

^{1}**H NMR (400 MHz, CDCl$_3$) of 13g.**

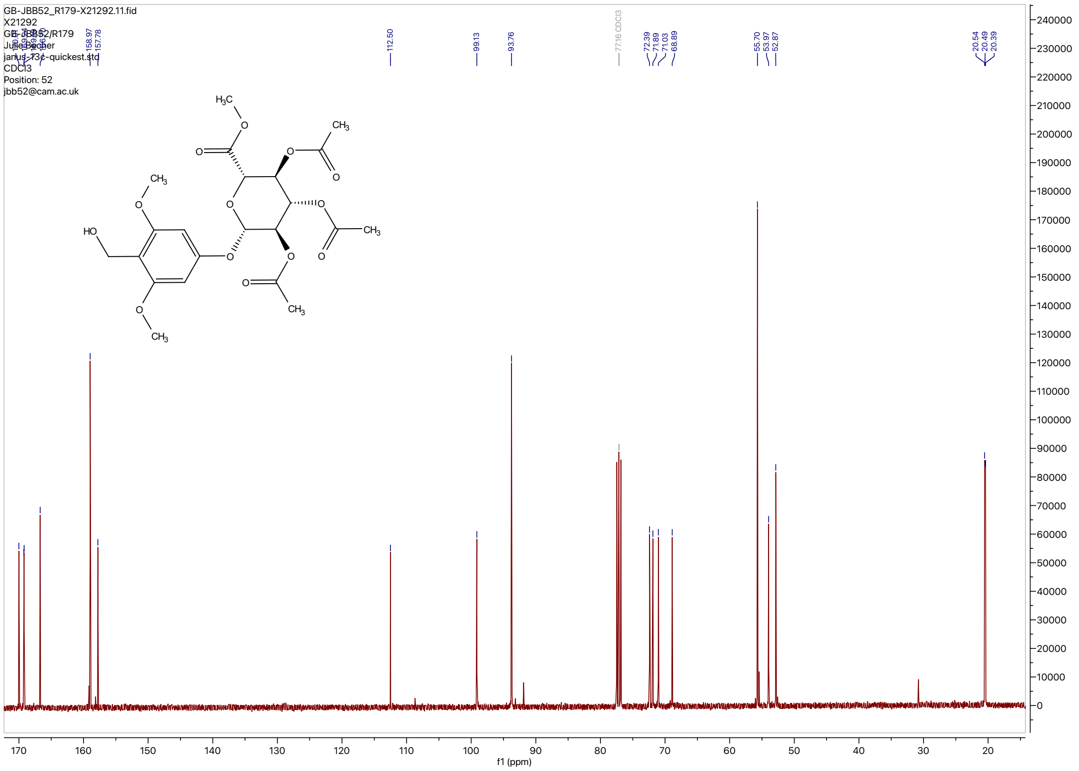

^{13}**C NMR (101 MHz, CDCl$_3$) of 13g.**

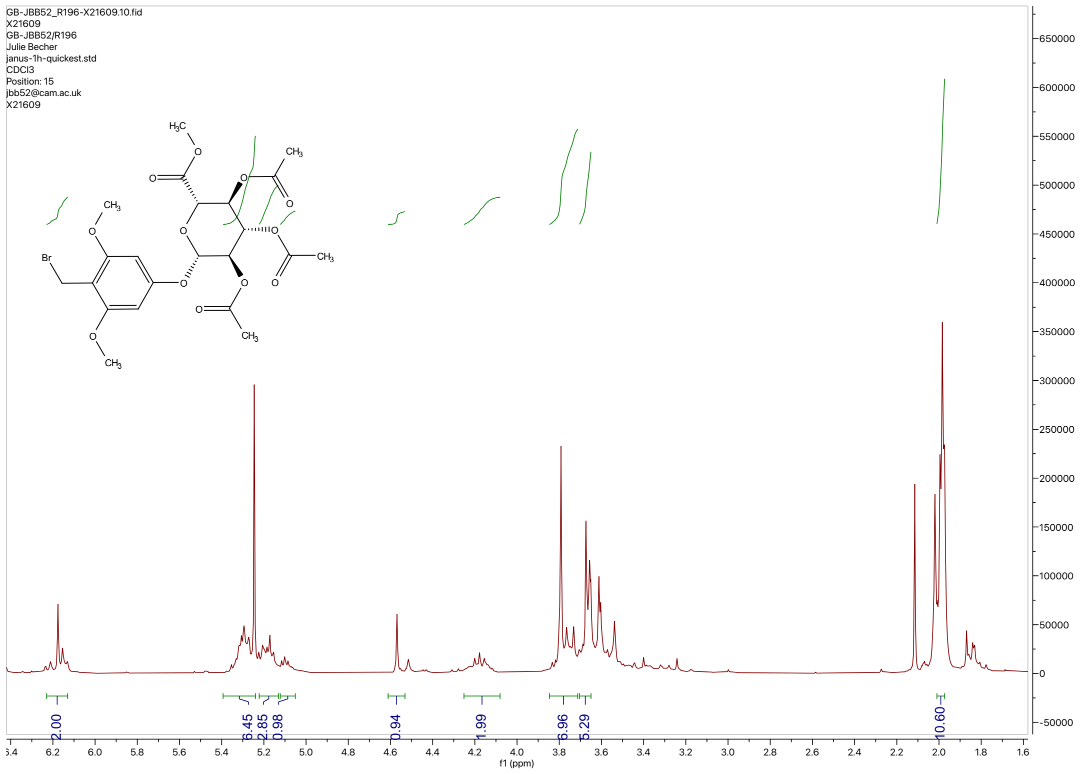

^{1}H NMR (400 MHz, CDCl₃) of 14g.

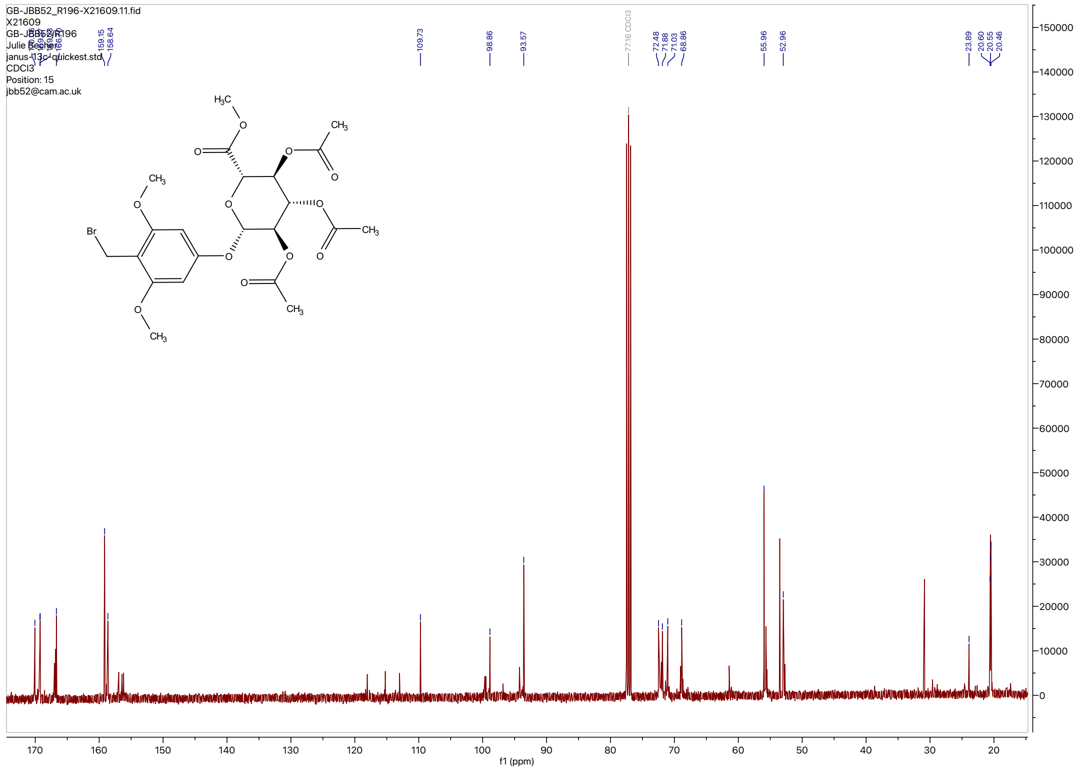

^{13}C NMR (101 MHz, CDCl₃) of 14g.

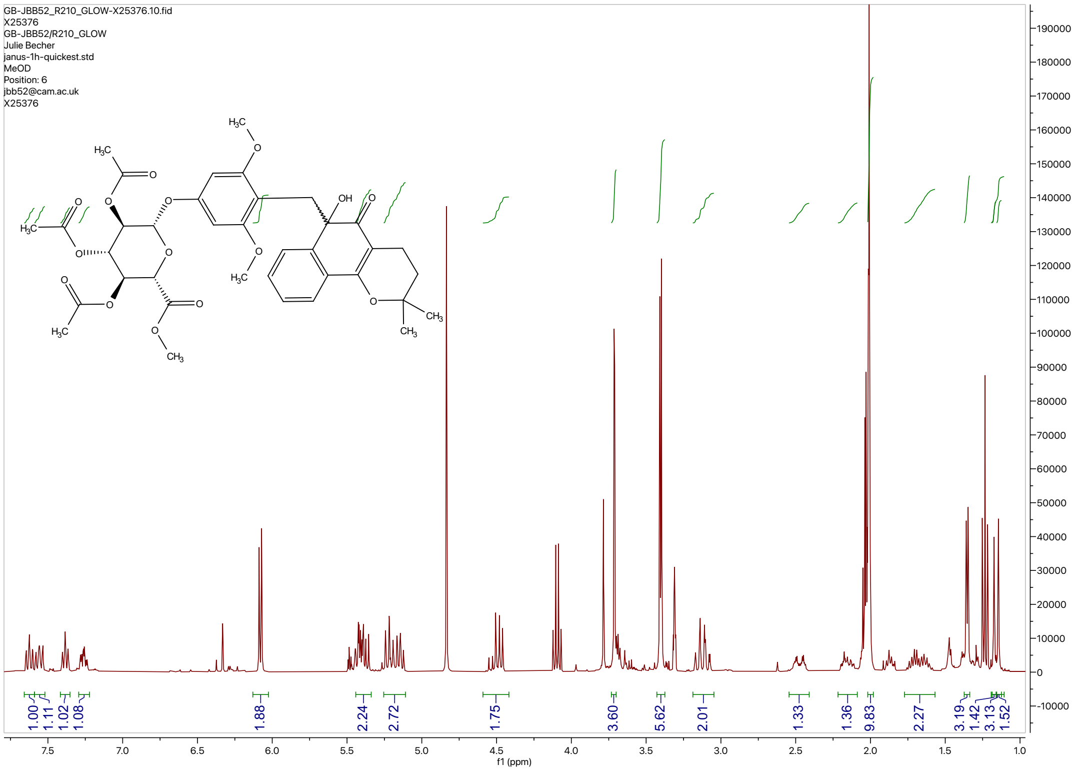

^{1}H NMR (400 MHz, MeOD, mixture of diastereomers 1:1) of 15g.

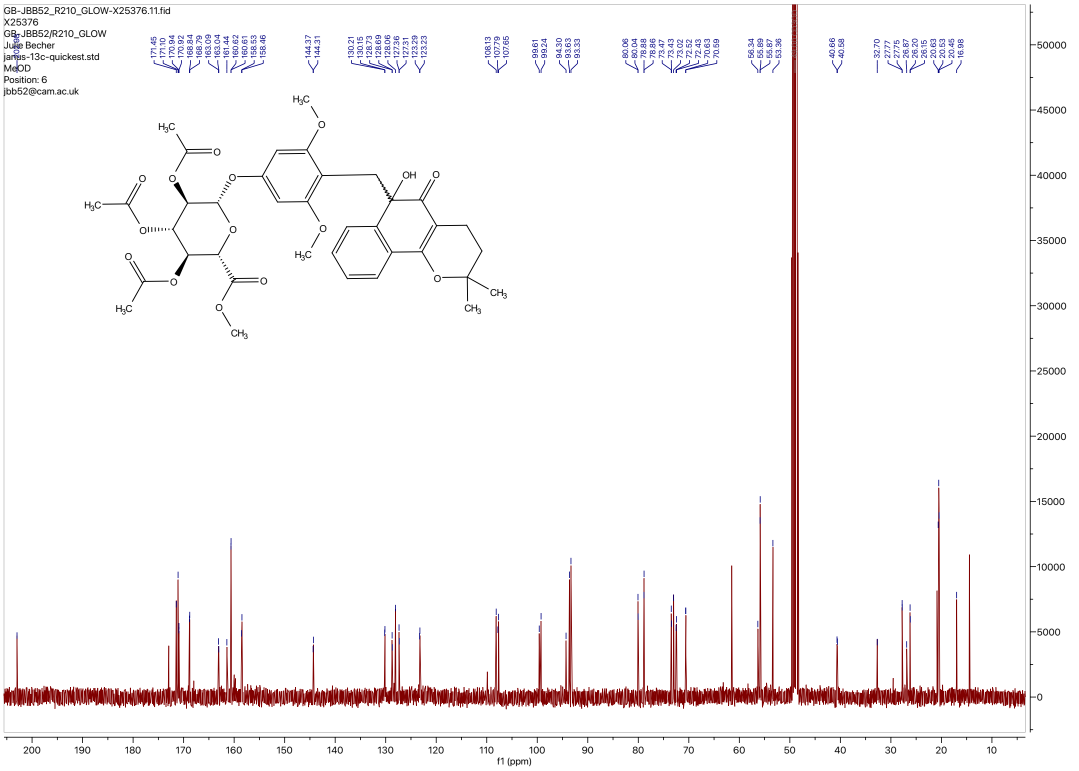

^{13}C NMR (101 MHz, MeOD, mixture of diastereomers 1:1) of 15g.

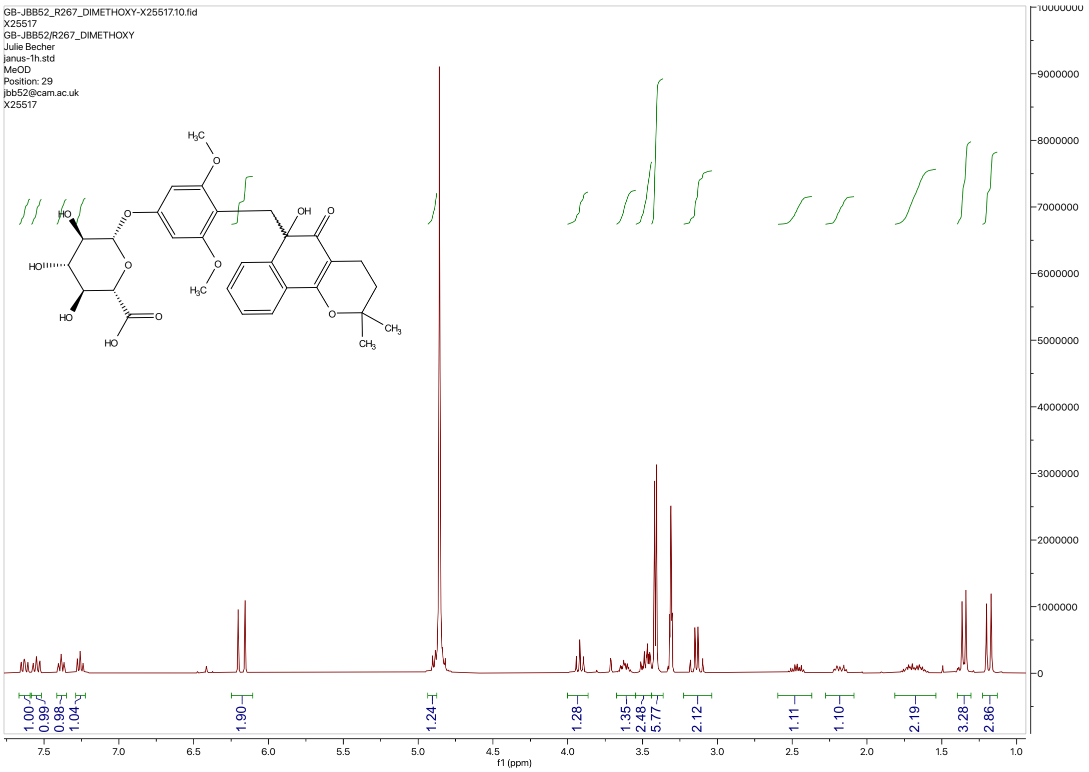

^{1}H NMR (400 MHz, MeOD, mixture of diastereomers 1:1) of 16g.

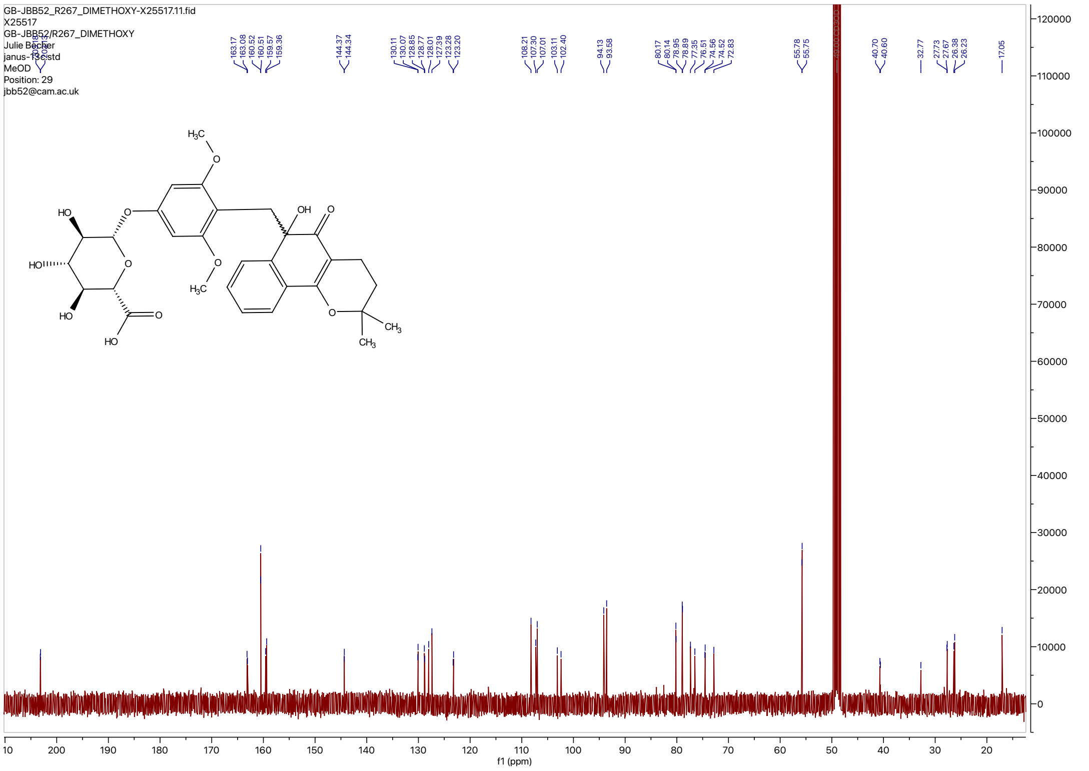

^{13}C NMR (101 MHz, MeOD, mixture of diastereomers) of 16g.

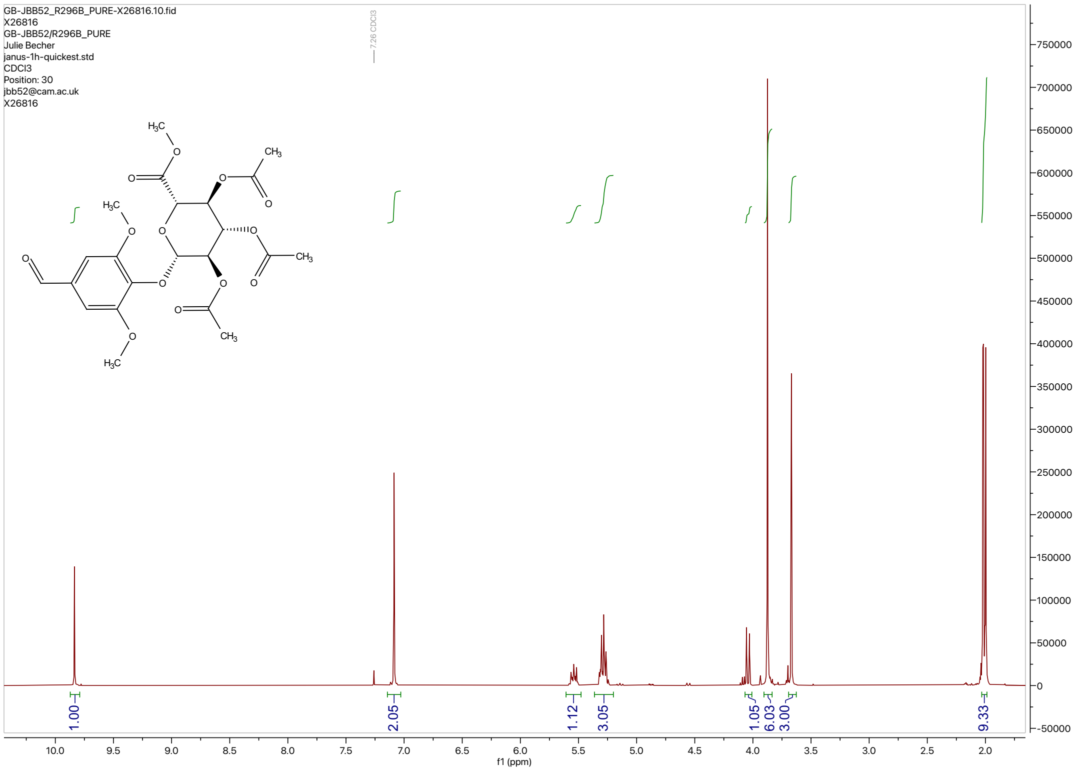

^{1}H NMR (400 MHz, CDCl$_3$) of 12h.

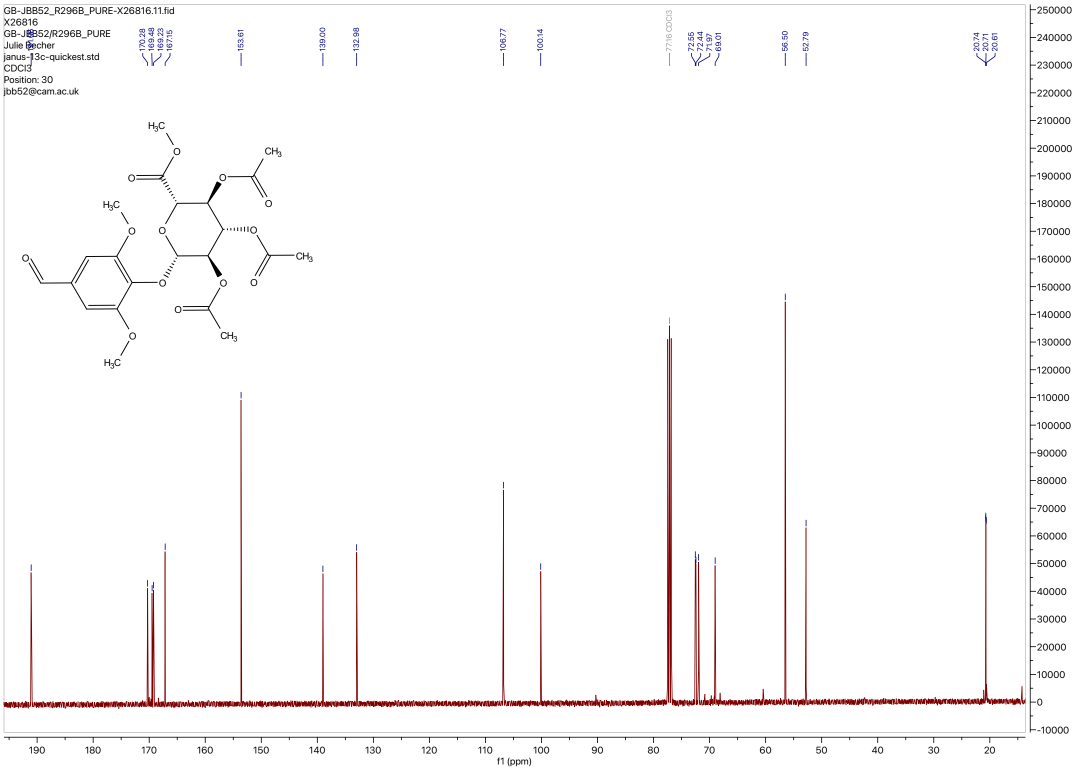

^{13}C NMR (101 MHz, CDCl$_3$) of 12h.

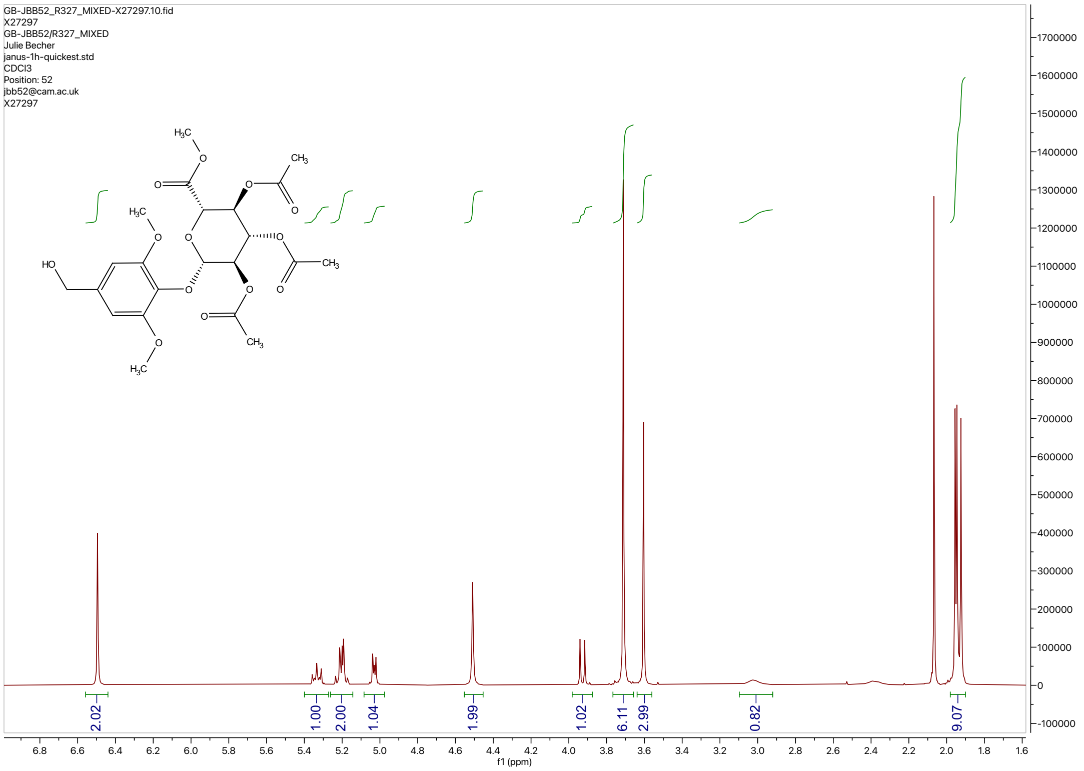

^1H NMR (400 MHz, $CDCl_3$) of 13h.

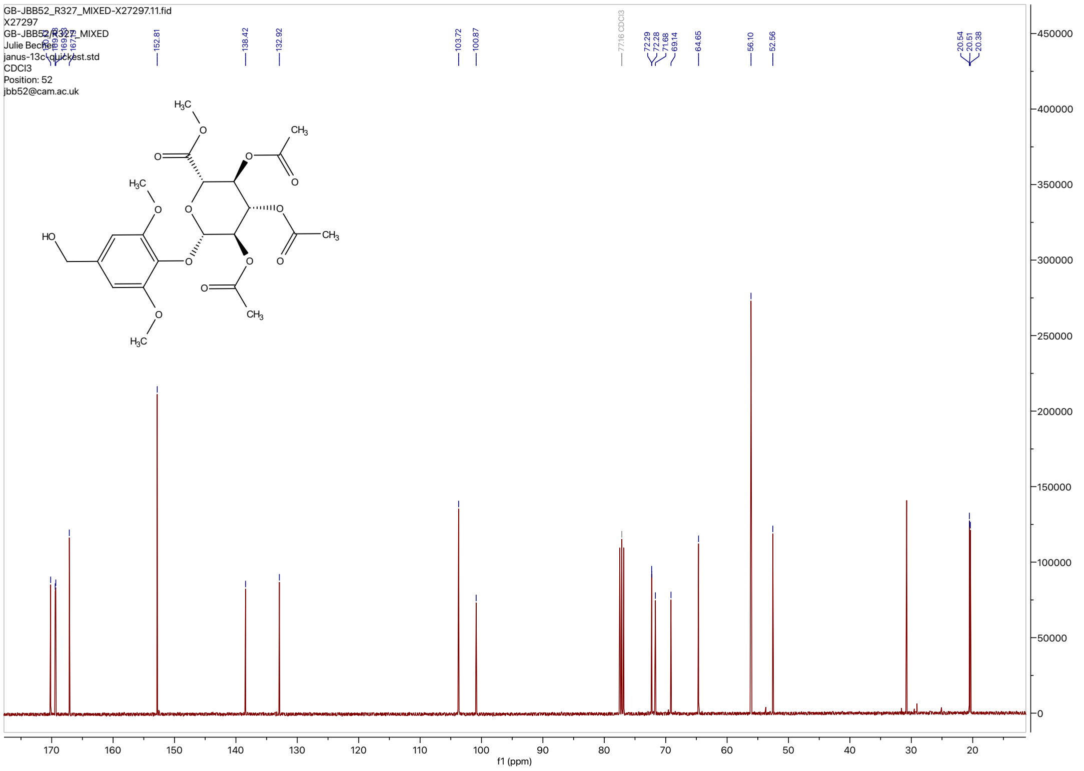

^{13}C NMR (101 MHz, $CDCl_3$) of 13h.

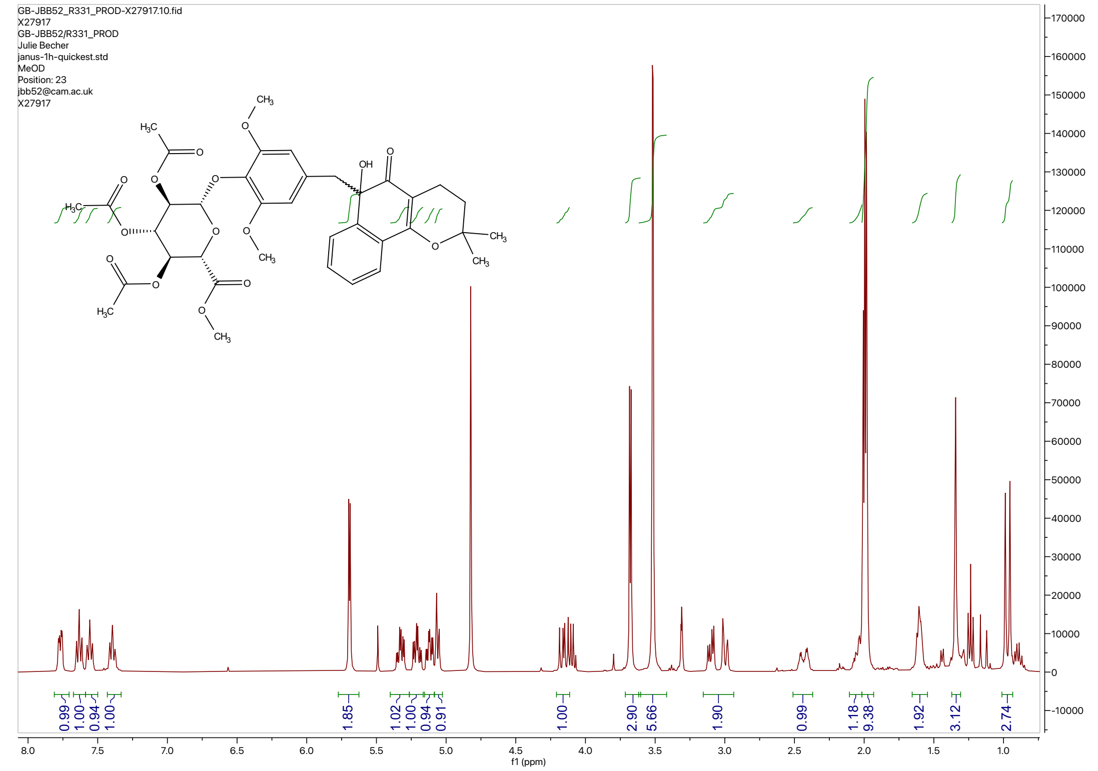

^{1}H NMR (400 MHz, MeOD, mixture of diastereomers 1:1) of 15h.

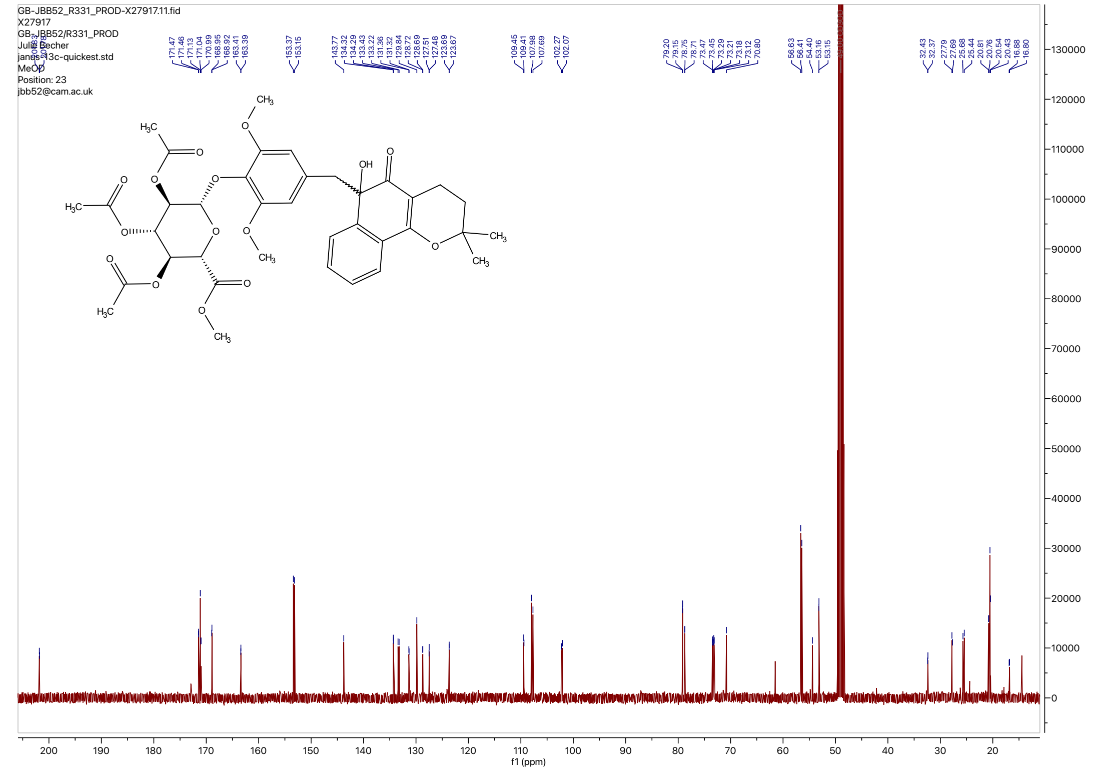

^{13}C NMR (101 MHz, MeOD, mixture of diastereomers 1:1) of 15h.

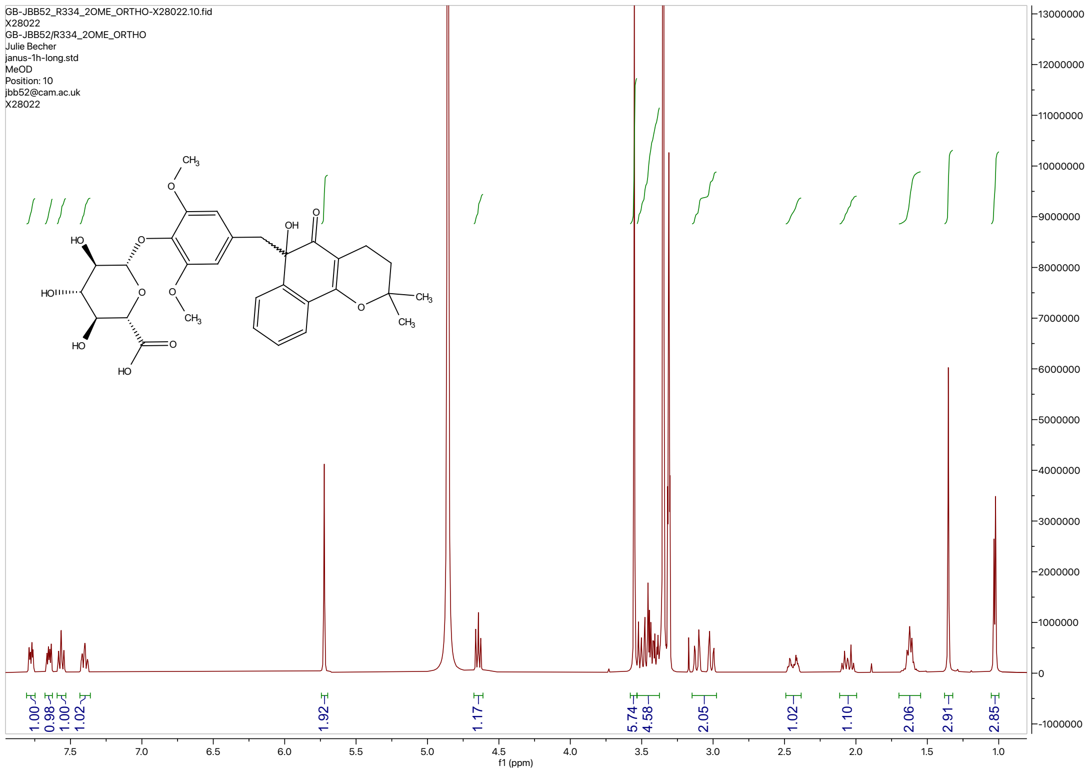

^{1}H NMR (400 MHz, MeOD, mixture of diastereomers 1:1) of 16h.

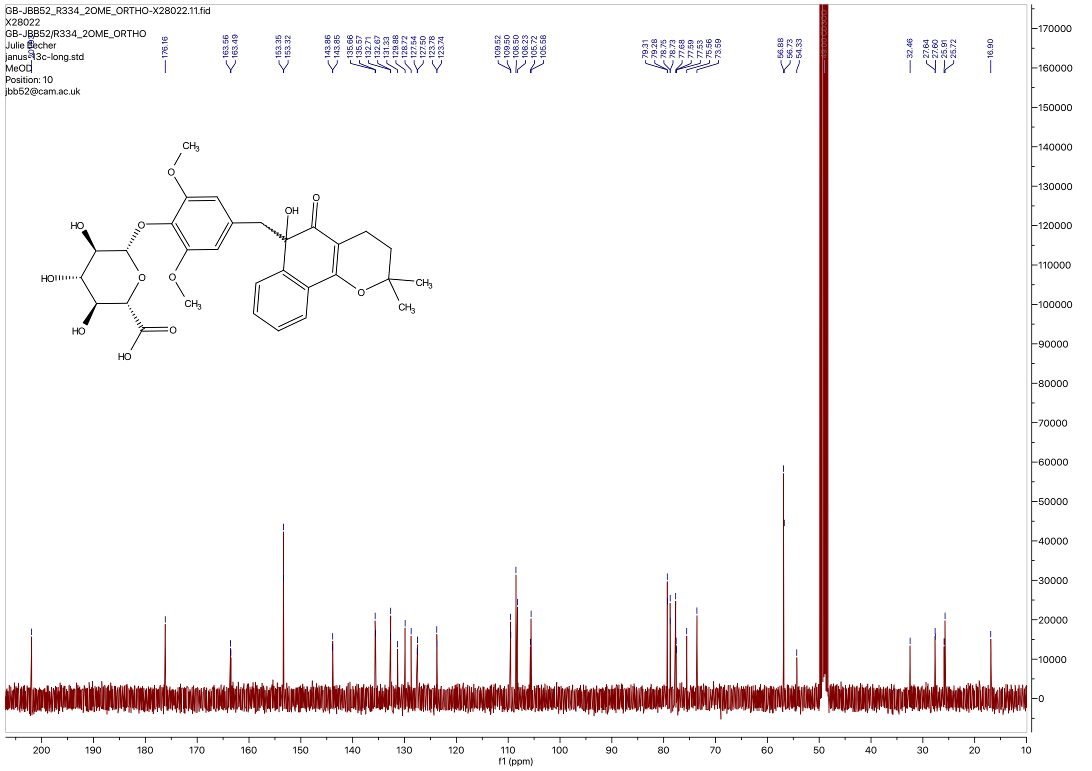

^{13}C NMR (101 MHz, MeOD, mixture of diastereomers 1:1) of 16h.

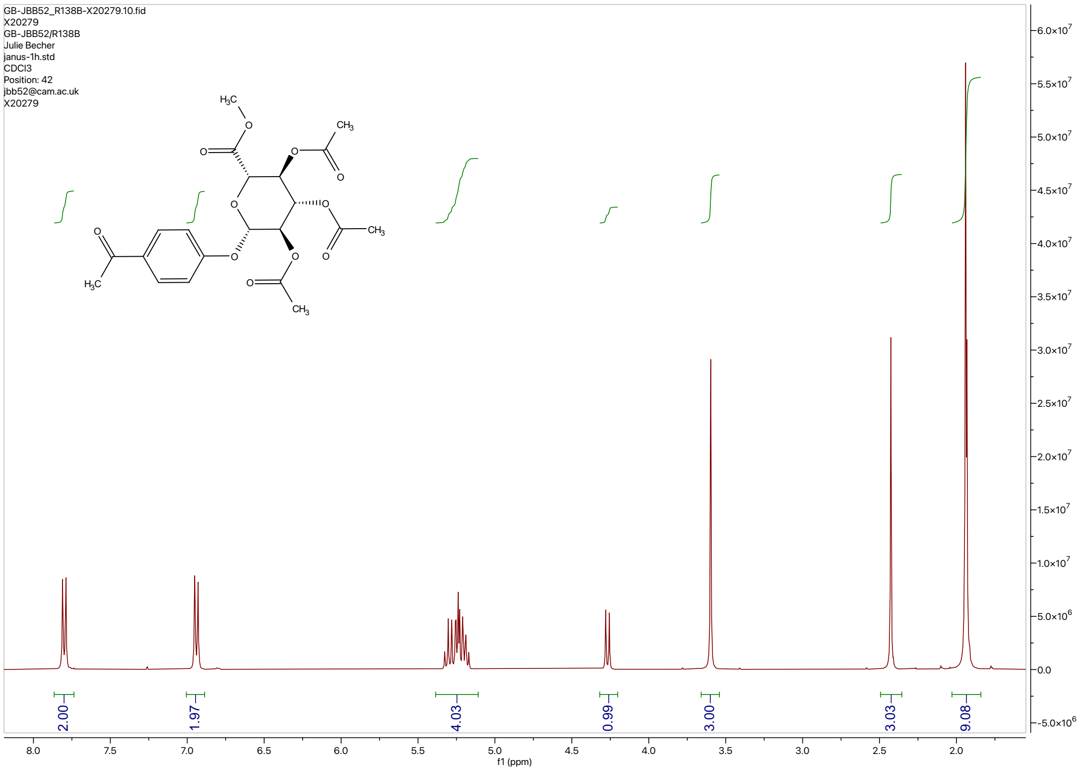

^{1}H NMR (400 MHz, CDCl$_3$) of 12i.

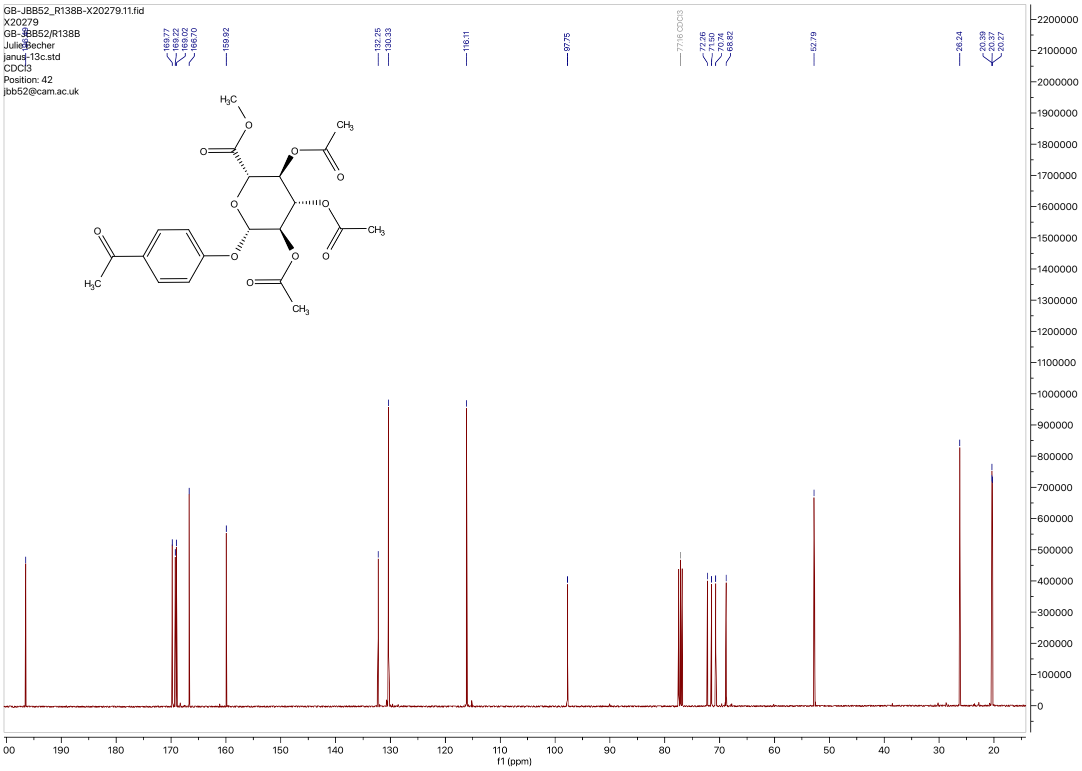

^{13}C NMR (101 MHz, CDCl$_3$) of 12i.

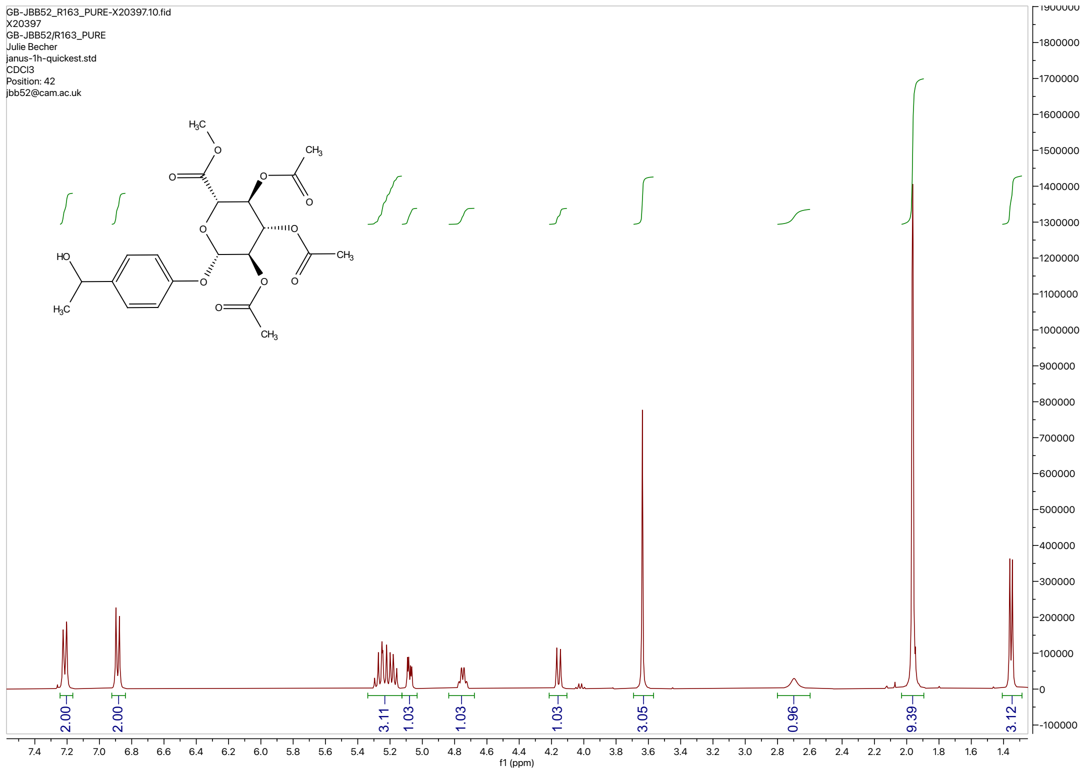

^{1}H NMR (400 MHz, $CDCl_3$, mixture of diastereomers 1:1) of 13i.

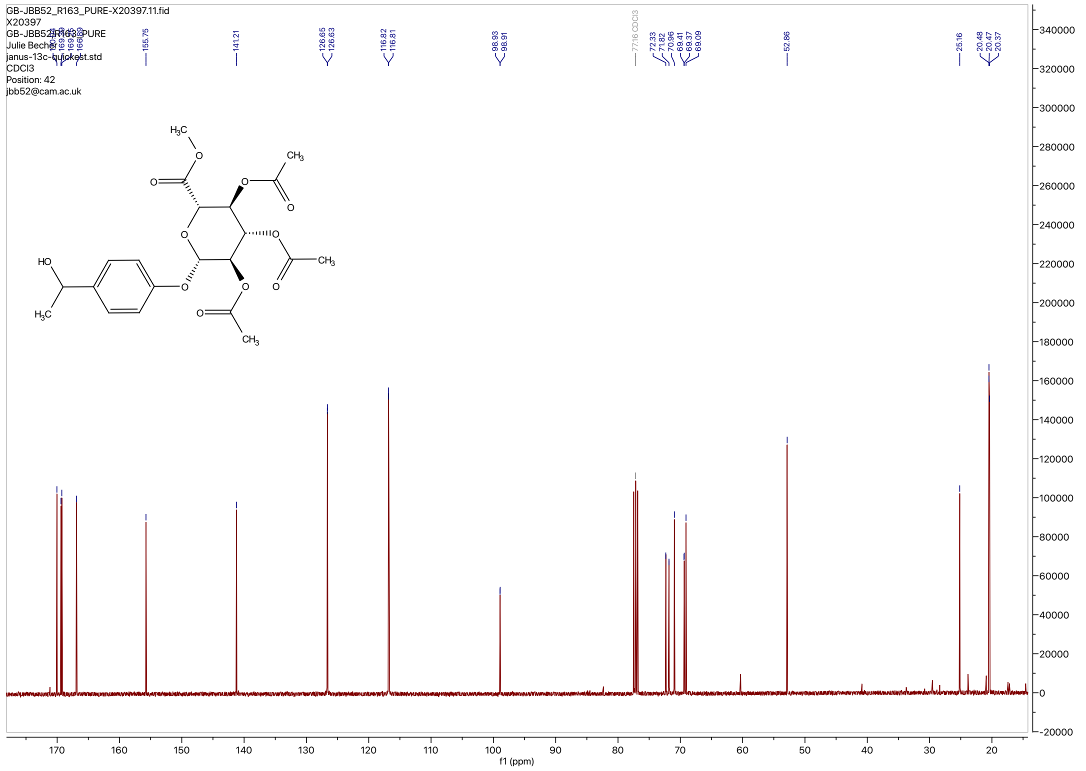

^{13}C NMR (101 MHz, $CDCl_3$, mixture of diastereomers 1:1) of 13i.

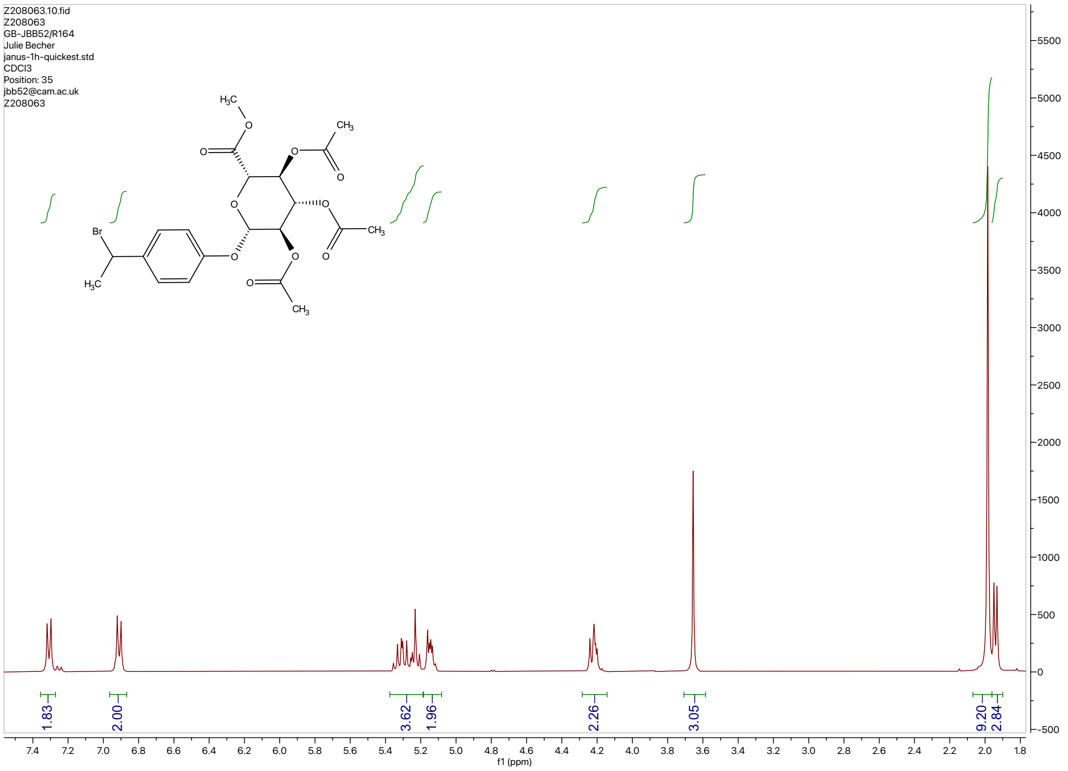

^{1}H NMR (400 MHz, CDCl$_3$, mixture of diastereomers 1:1) of 14i.

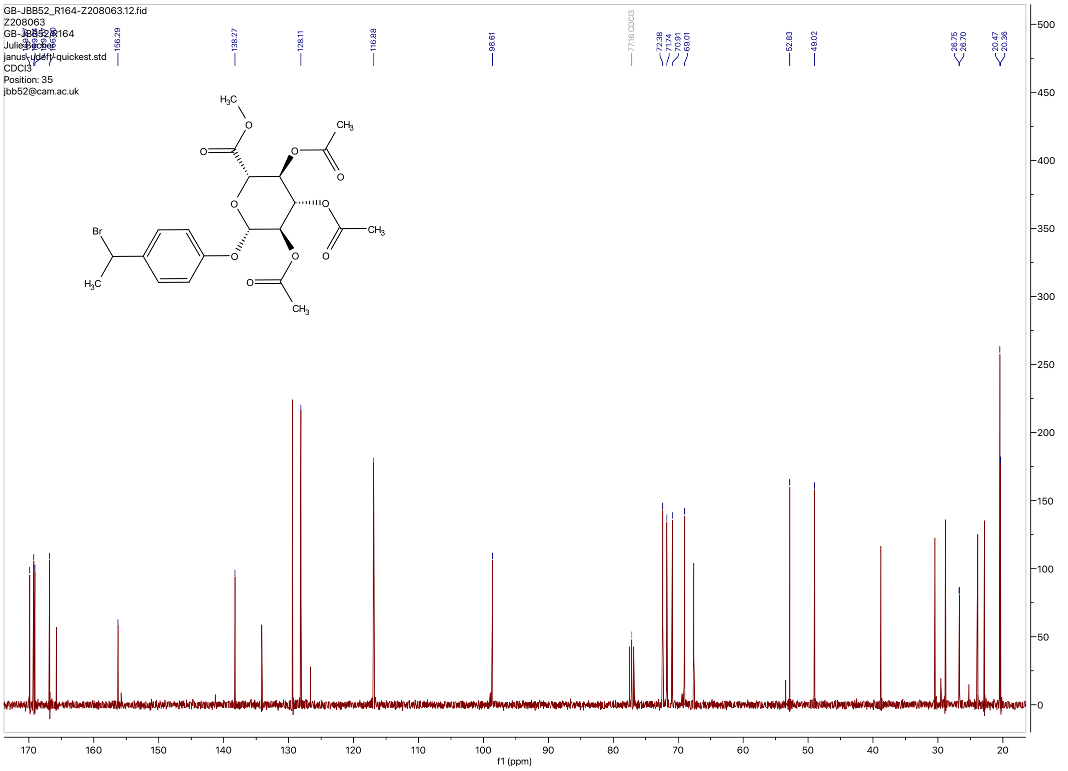

^{13}C NMR (101 MHz, CDCl$_3$, mixture of diastereomers 1:1) of 14i.

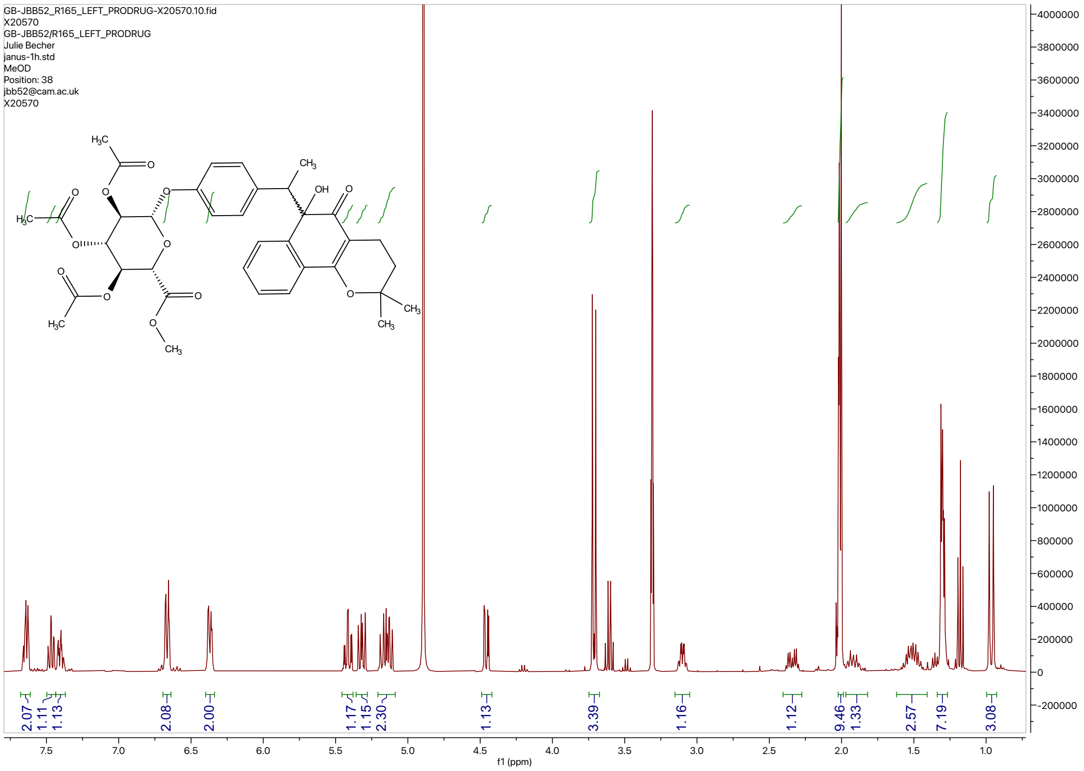

^{1}H NMR (400 MHz, MeOD, mixture of 2 diastereomers 1:1) of 15i.

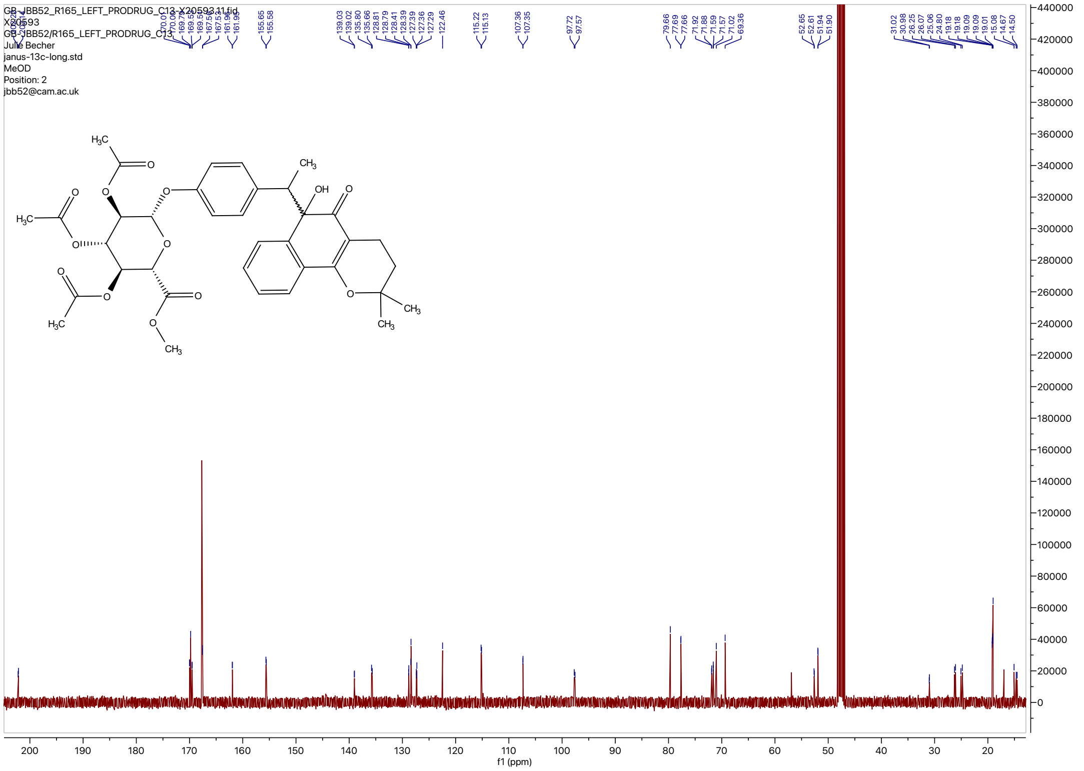

^{13}C NMR (101 MHz, MeOD, mixture of 2 diastereomers 1:1) of 15i.

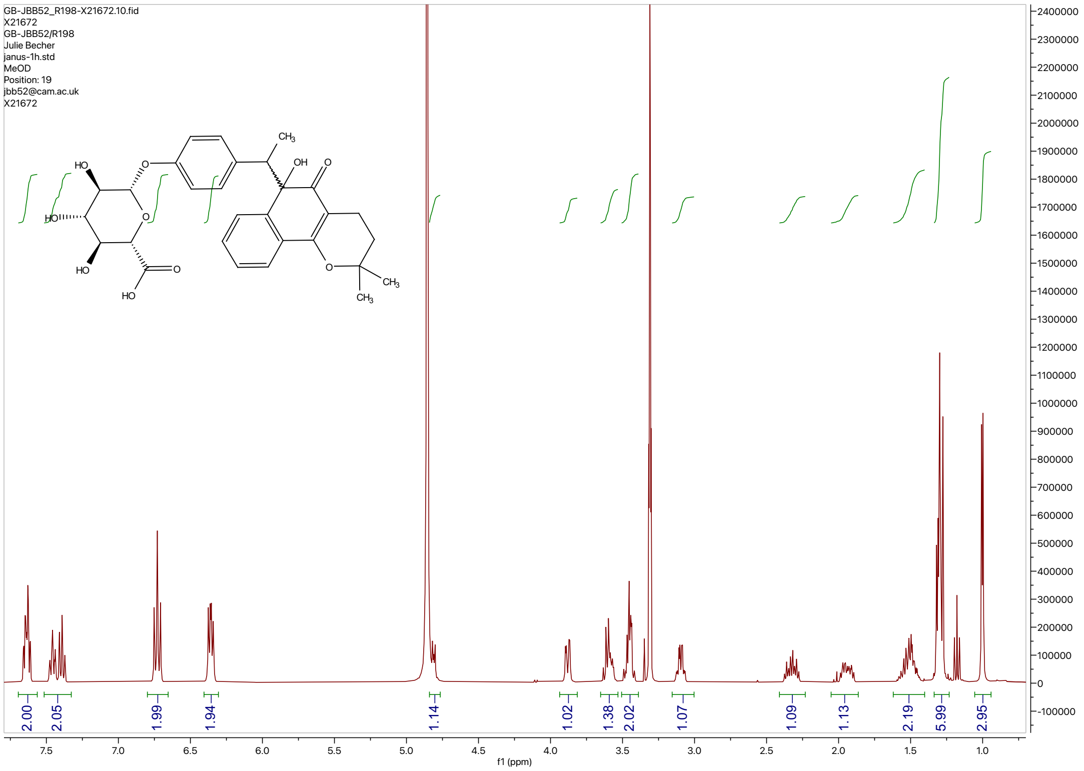

^{1}H NMR (400 MHz, MeOD, mixture of 2 diastereomers 1:1) of 16i.

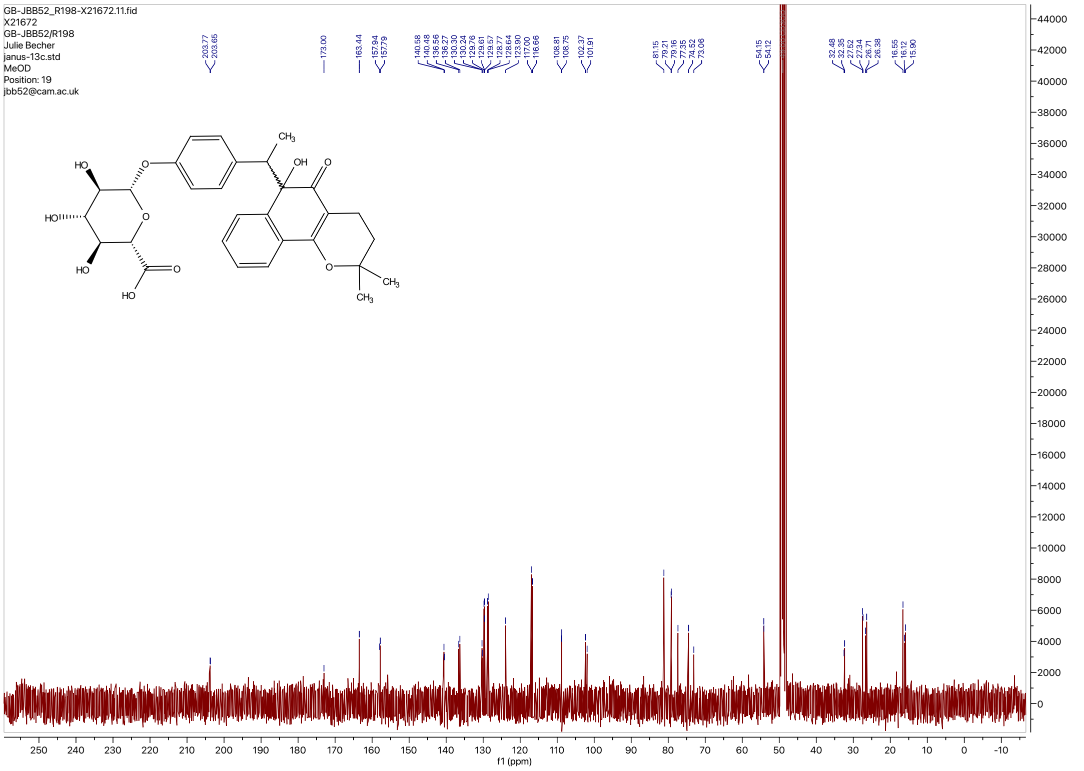

^{13}C NMR (101 MHz, MeOD, mixture of 2 diastereomers 1:1) of 16i.

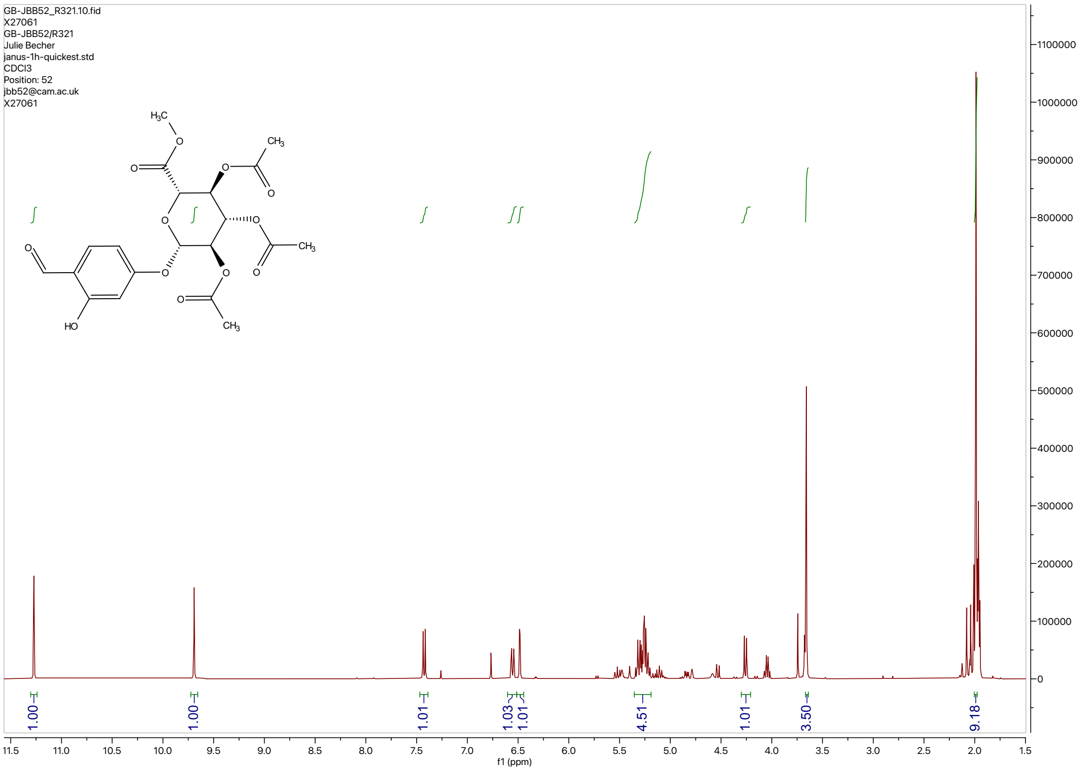

^{1}H NMR (400 MHz, CDCl$_3$) of 12j.

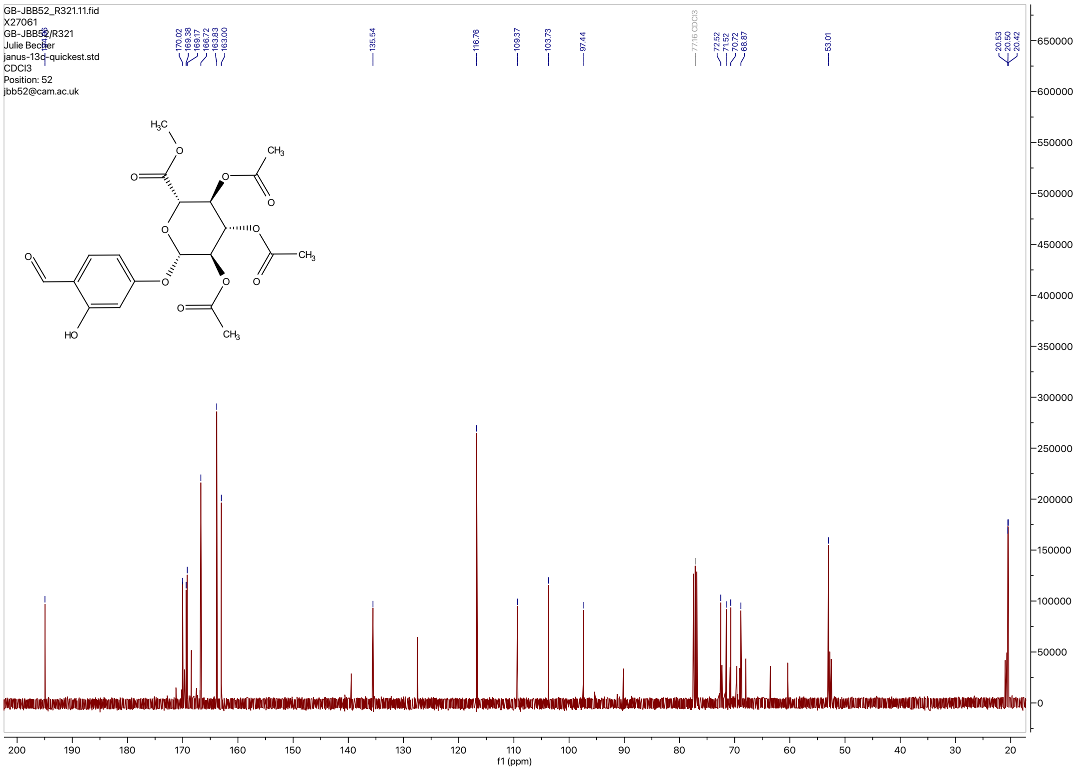

^{13}C NMR (101 MHz, CDCl$_3$) of 12j.

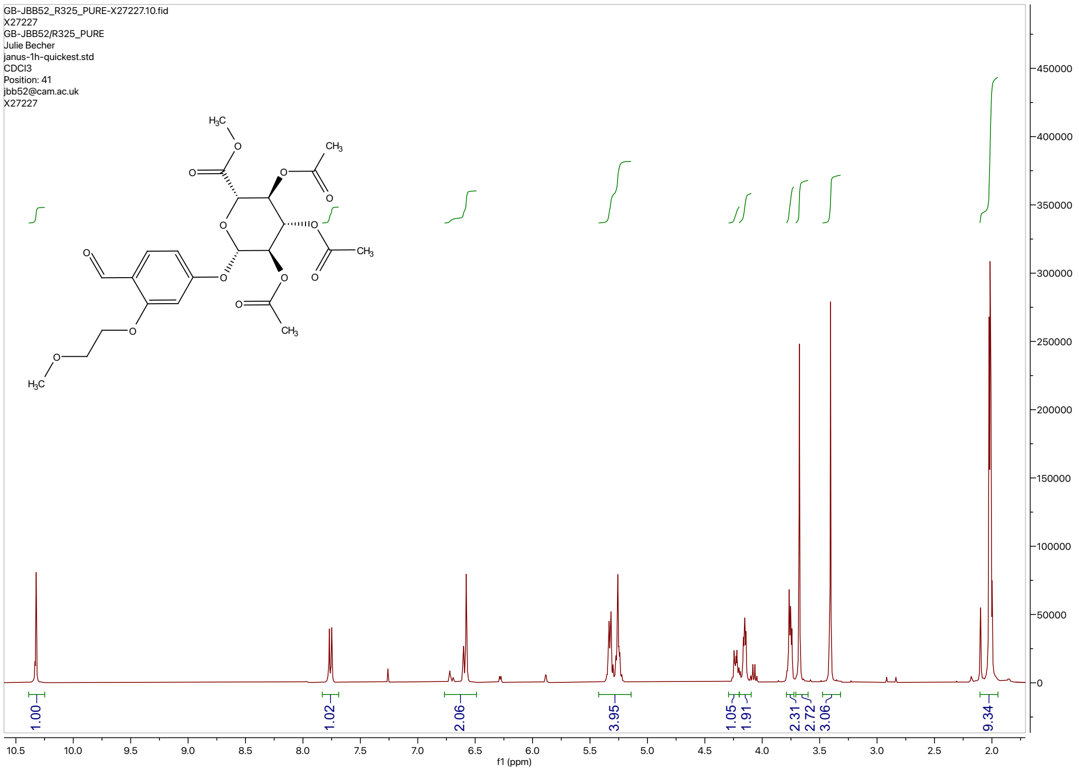

^{1}H NMR (400 MHz, CDCl$_3$) of 17.

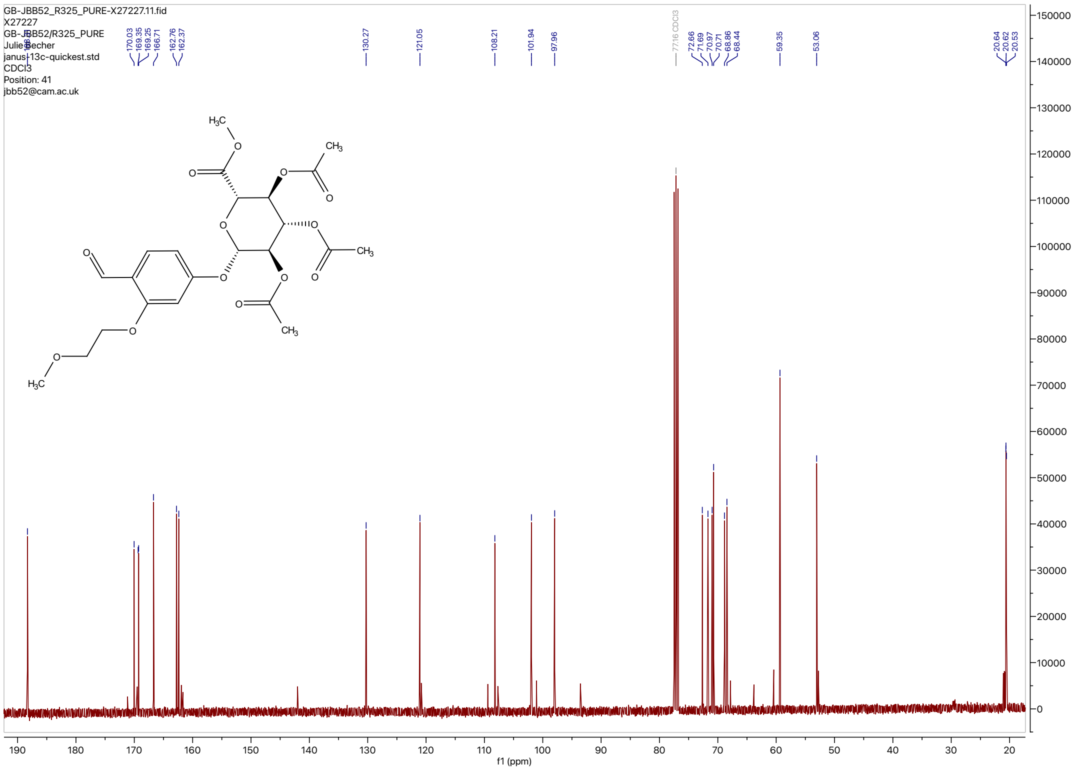

^{13}C NMR (101 MHz, CDCl$_3$) of 17.

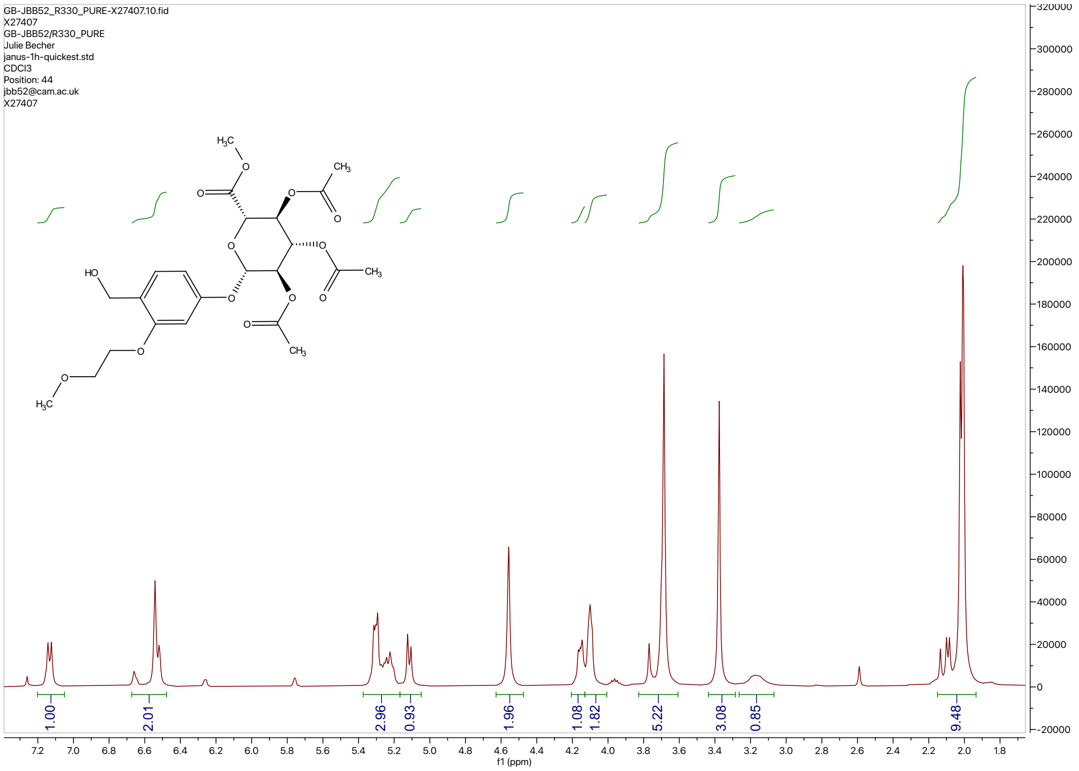

^{1}H NMR (400 MHz, $CDCl_3$) of 13j.

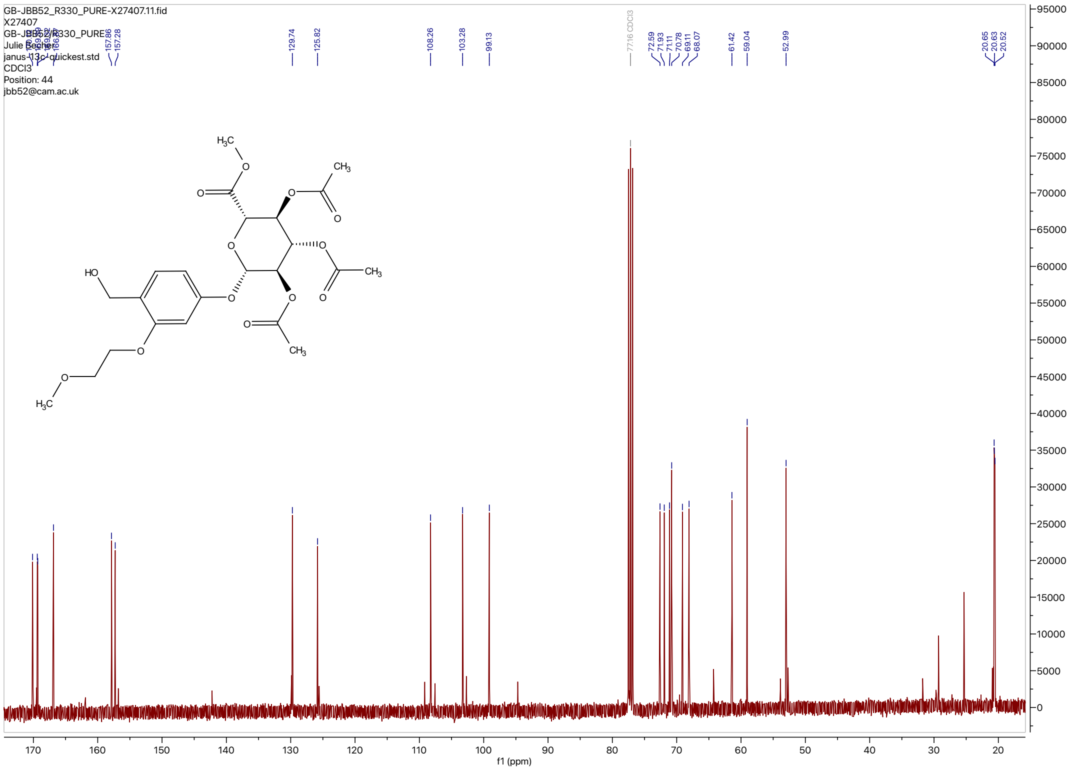

^{13}C NMR (101 MHz, $CDCl_3$) of 13j.

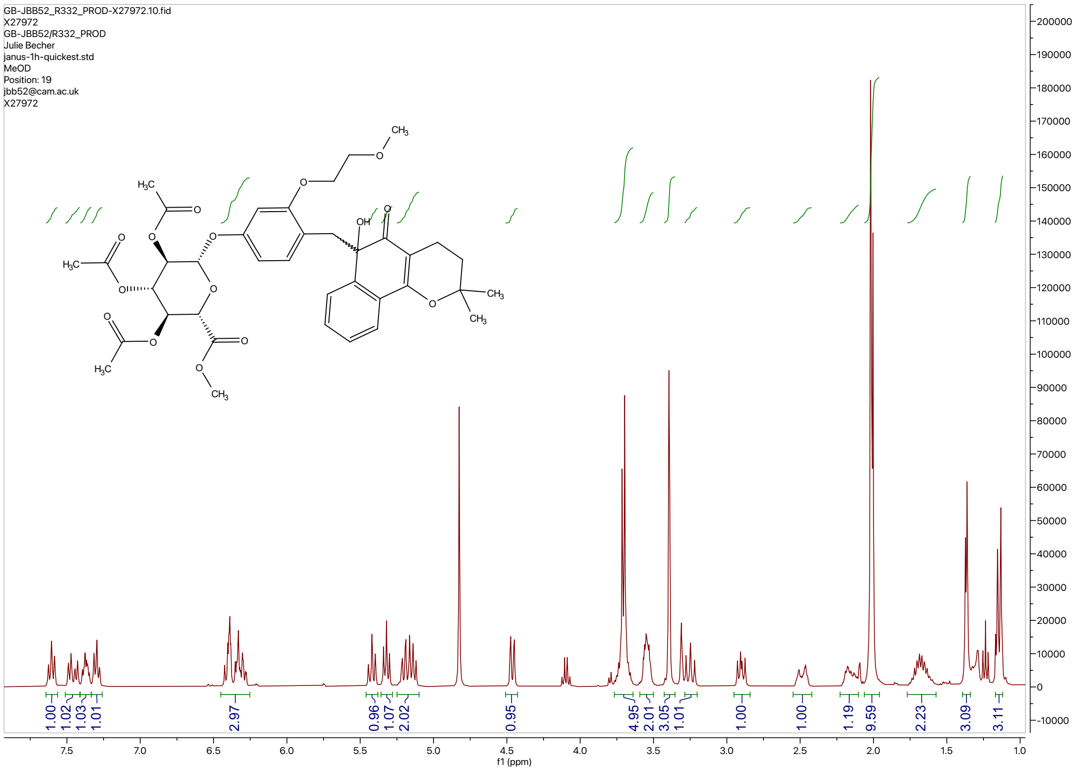

^{1}H NMR (400 MHz, MeOD, mixture of diastereomers 1:1) of 15j.

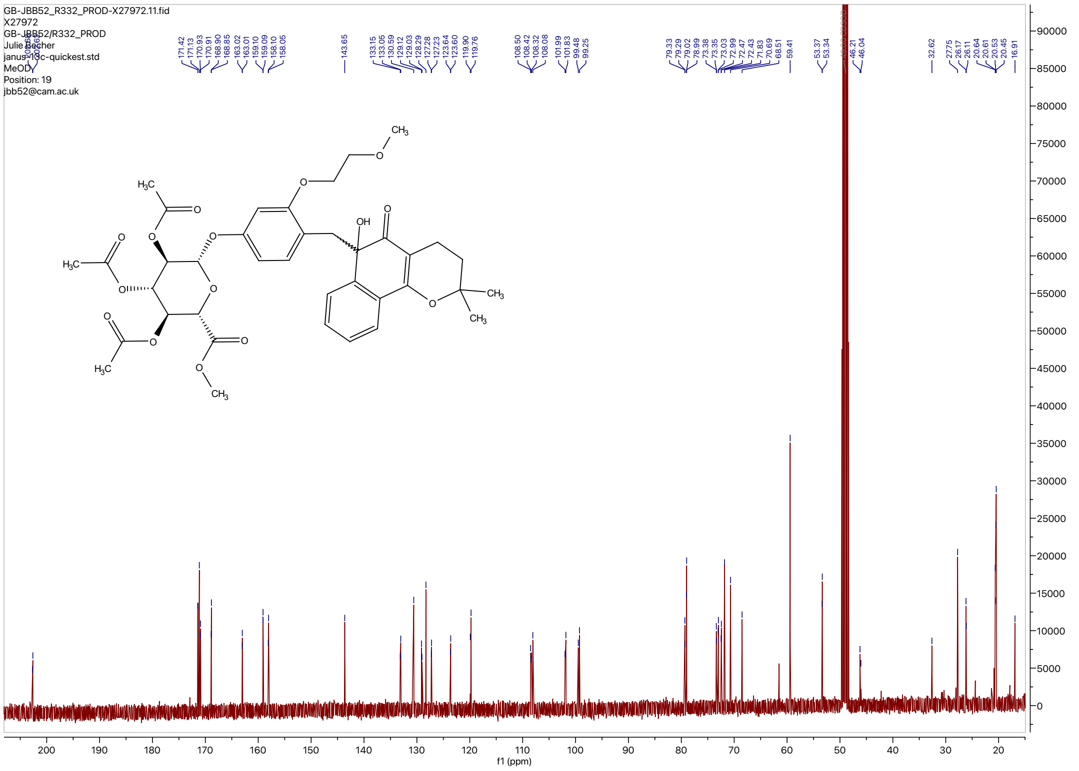

^{13}C NMR (101 MHz, MeOD, mixture of diastereomers 1:1) of 15j.

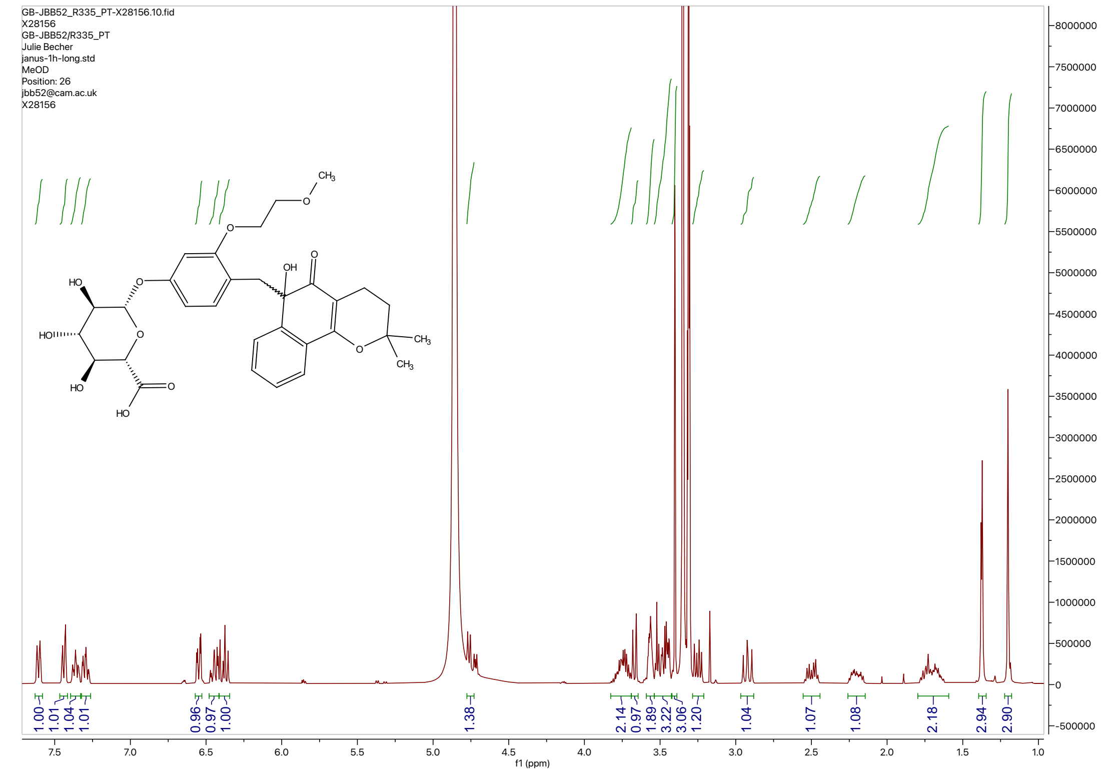

^{1}H NMR (400 MHz, MeOD, mixture of diastereomers 1:1) of 16j.

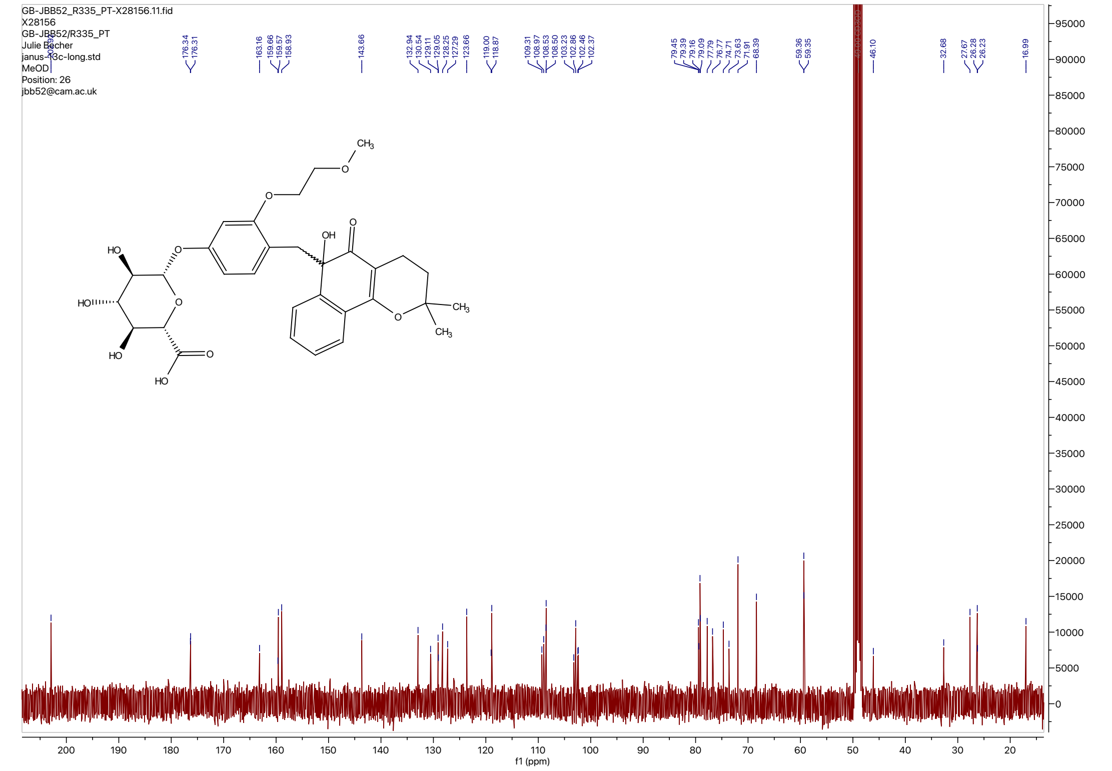

^{13}C NMR (101 MHz, MeOD, mixture of diastereomers 1:1) of 16j.

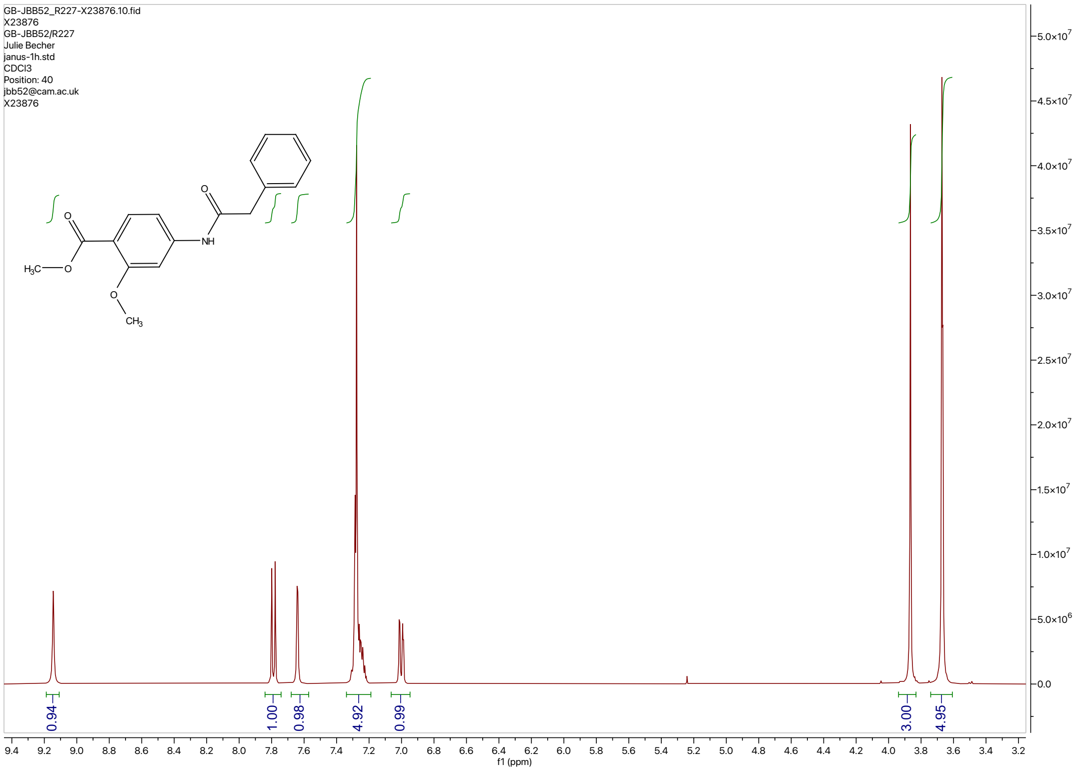

^{1}H NMR (400 MHz, $CDCl_3$) of 19.

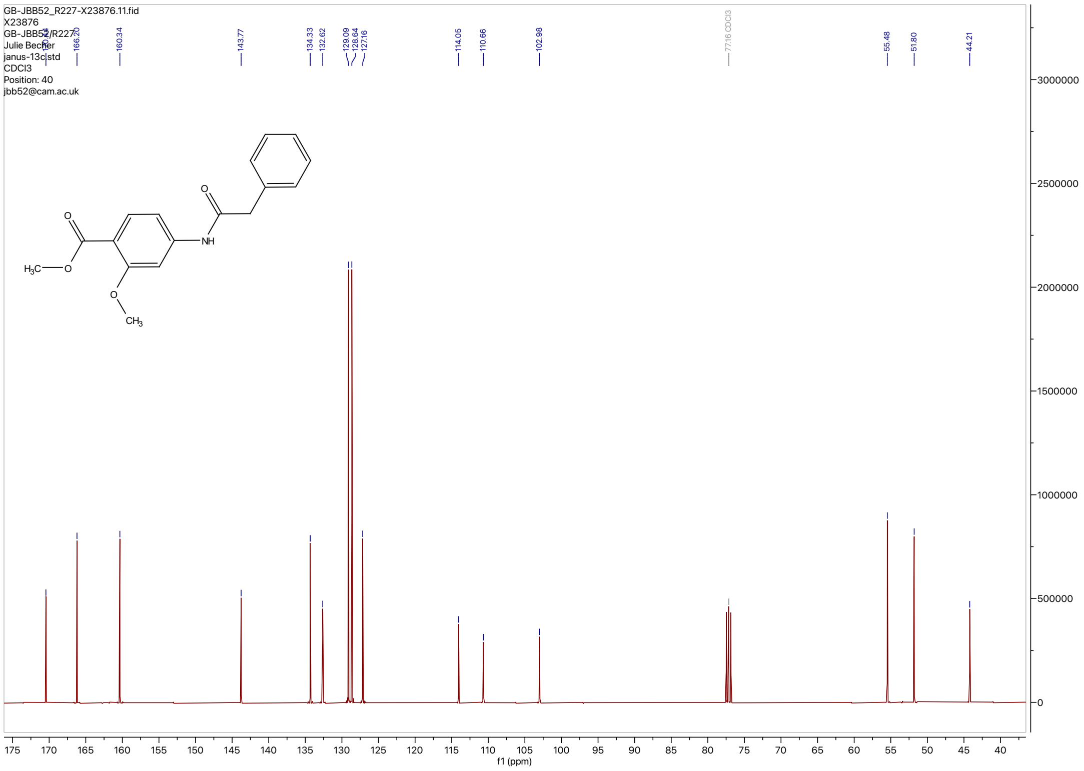

^{13}C NMR (101 MHz, $CDCl_3$) of 19.

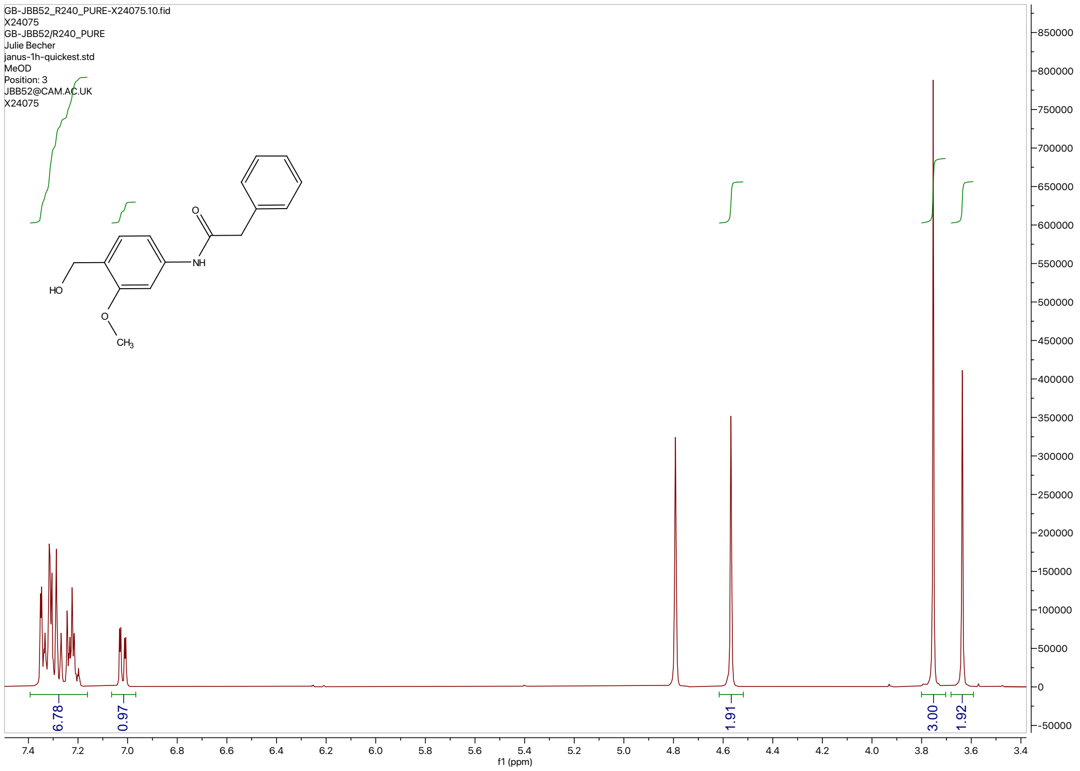

^{1}H NMR (400 MHz, MeOD) of 20.

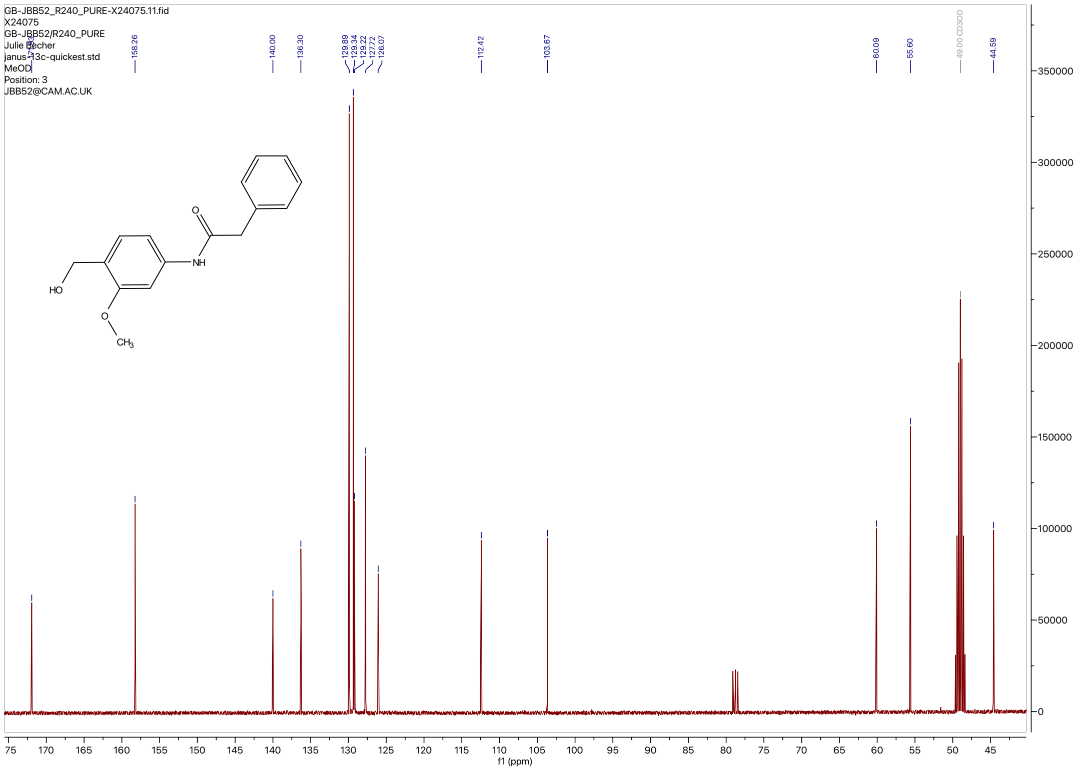

^{13}C NMR (101 MHz, MeOD) of 20.

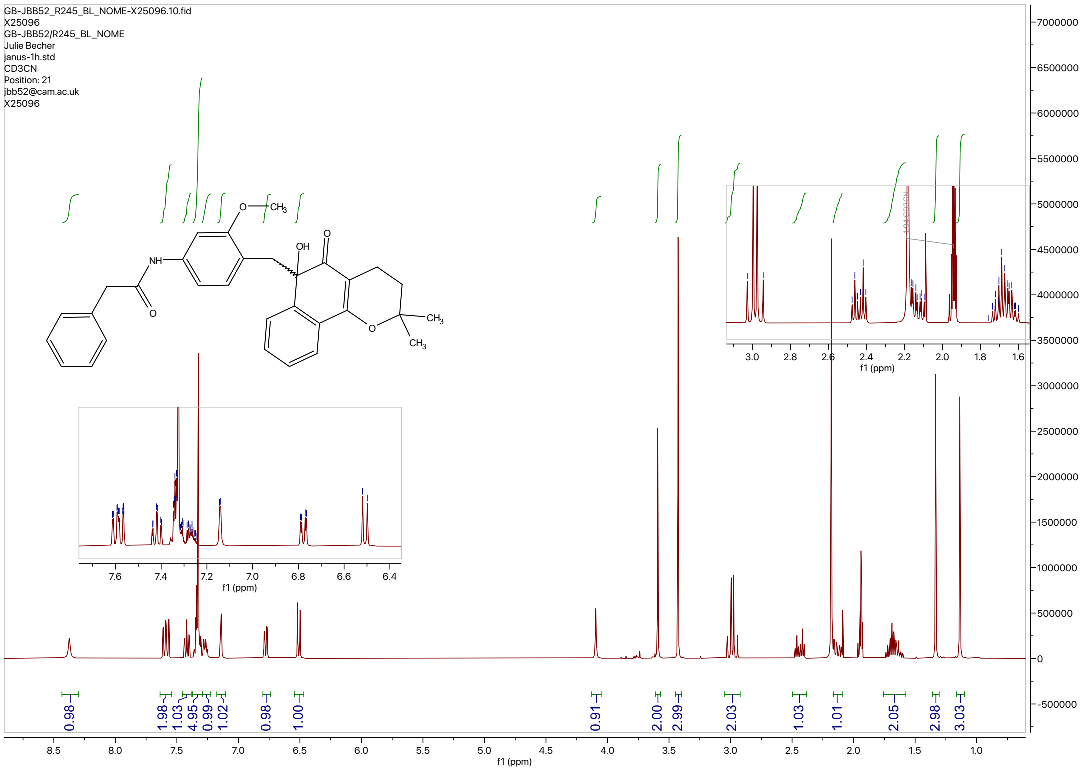

^{1}H NMR (400 MHz, CD_3CN) of 22.

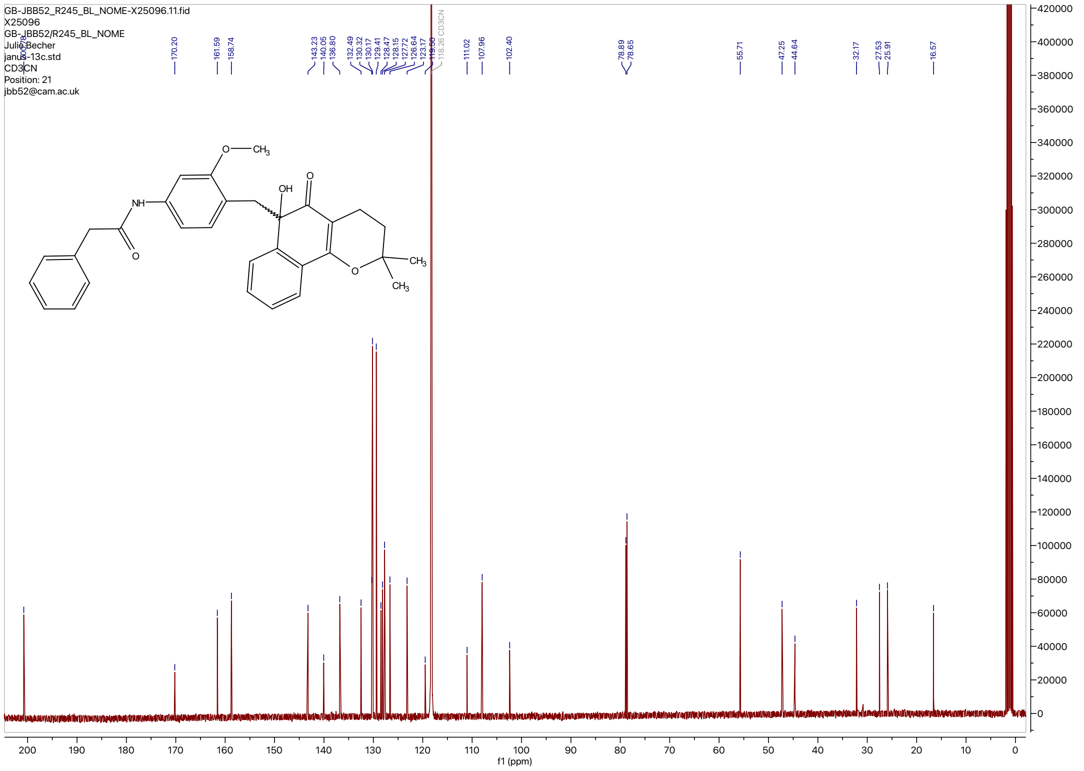

^{13}C NMR (101 MHz, CD_3CN) of 22.

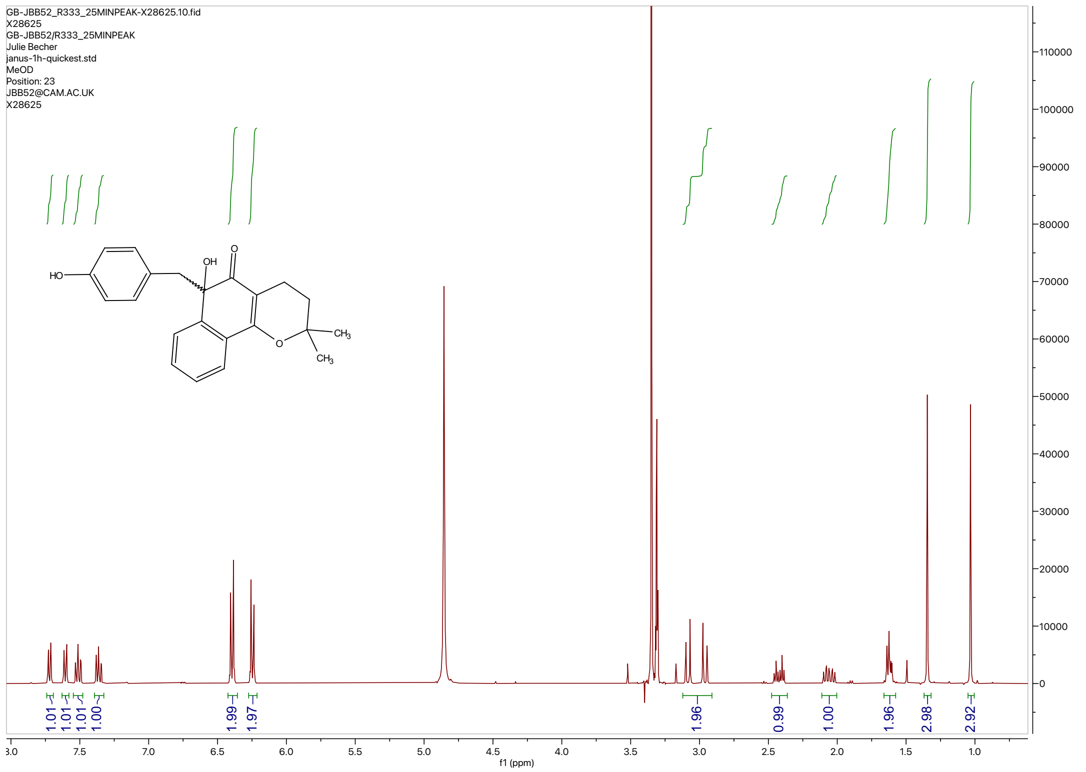

^{1}H NMR (400 MHz, MeOD) of 3a.

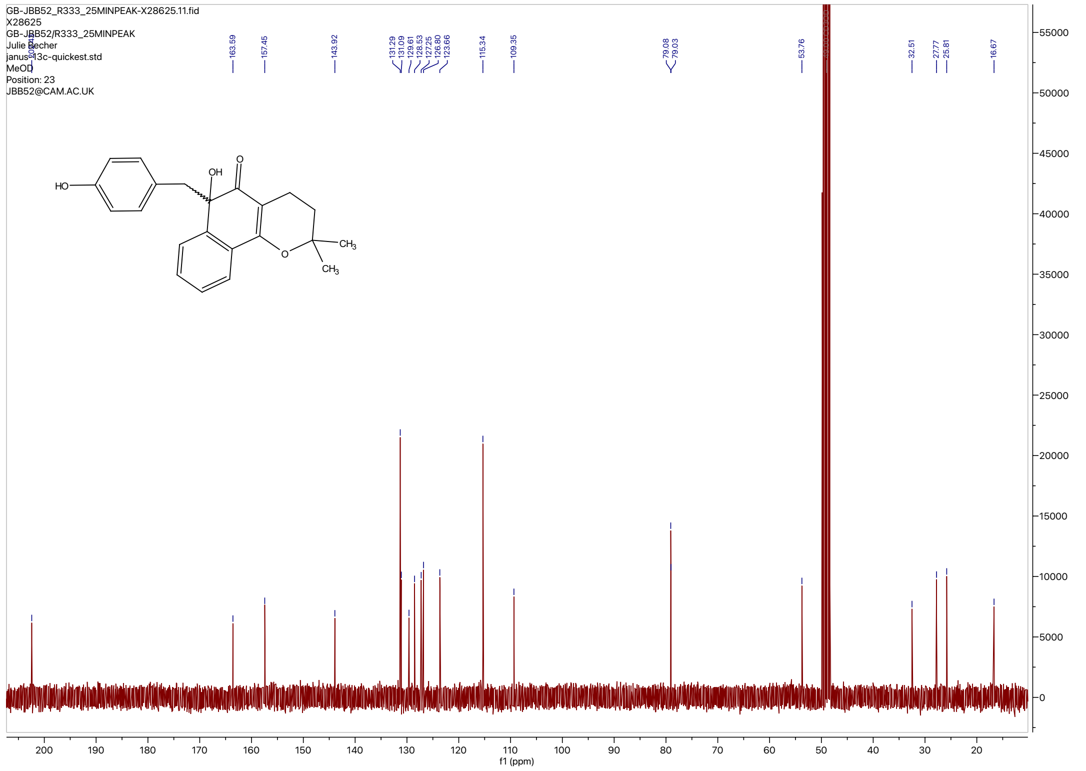

^{13}C NMR (101 MHz, MeOD) of 3a.

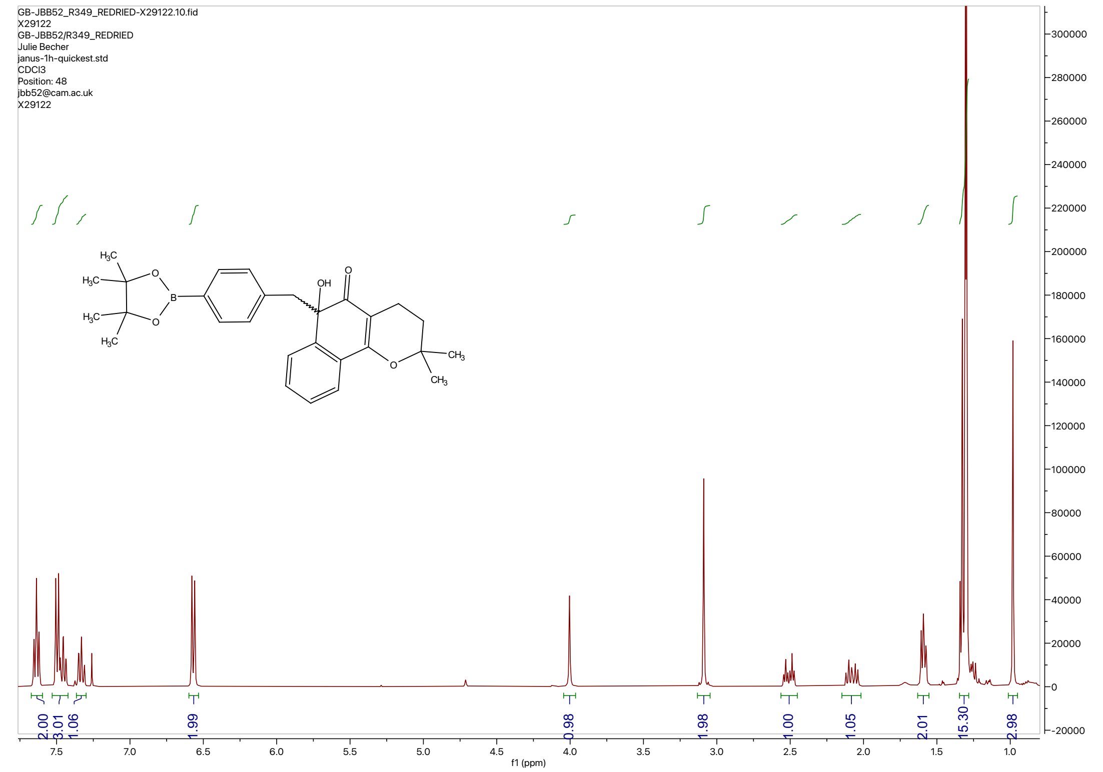

^{1}H NMR (400 MHz, CDCl$_3$) of 24.

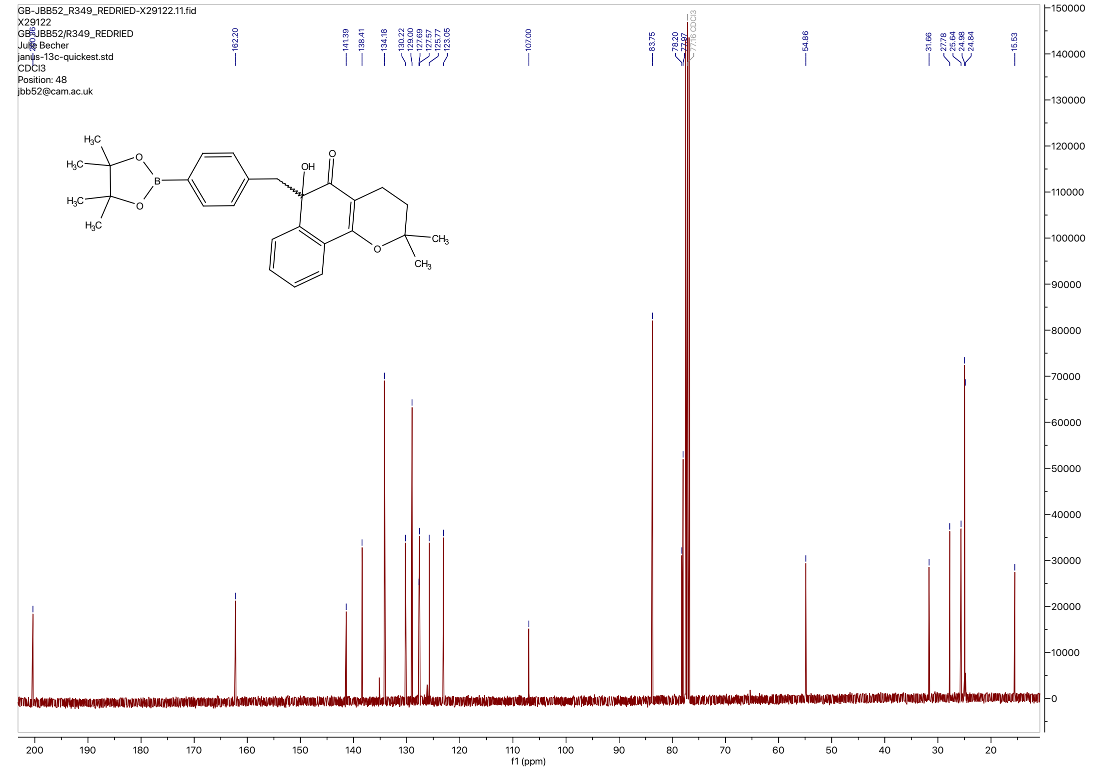

^{13}C NMR (101 MHz, CDCl$_3$) of 24.

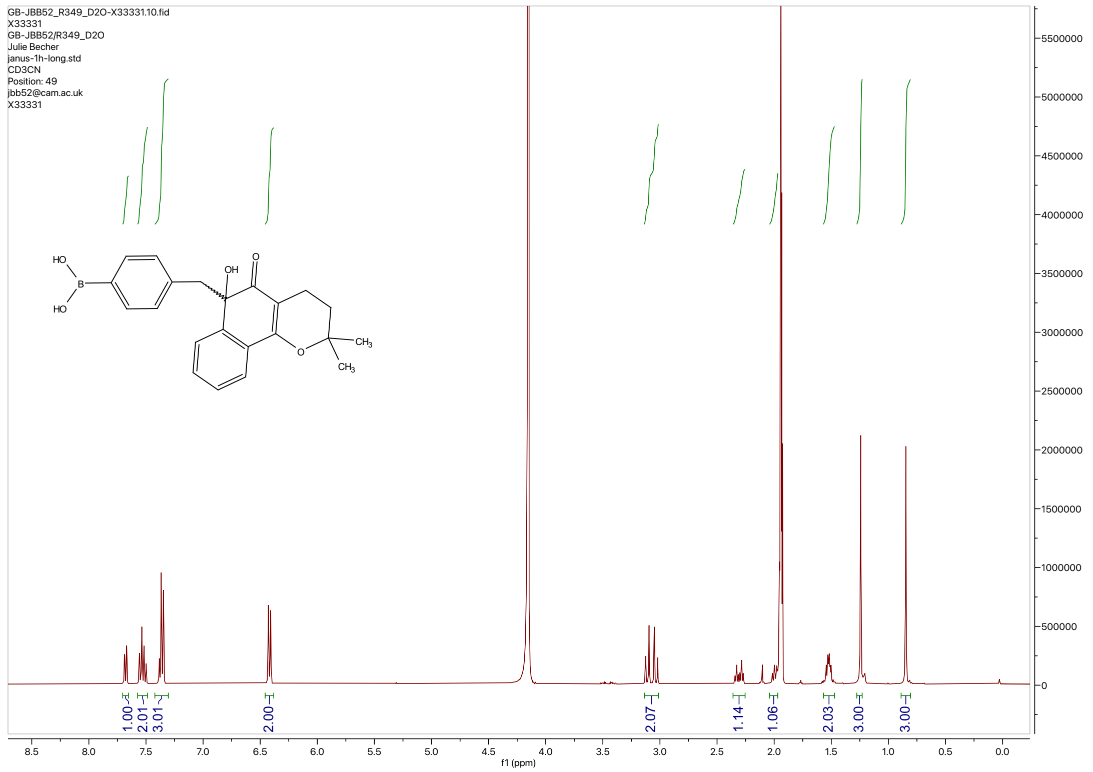

^{1}H NMR (400 MHz, 1:1 D_2O:CD_3CN) of 26.

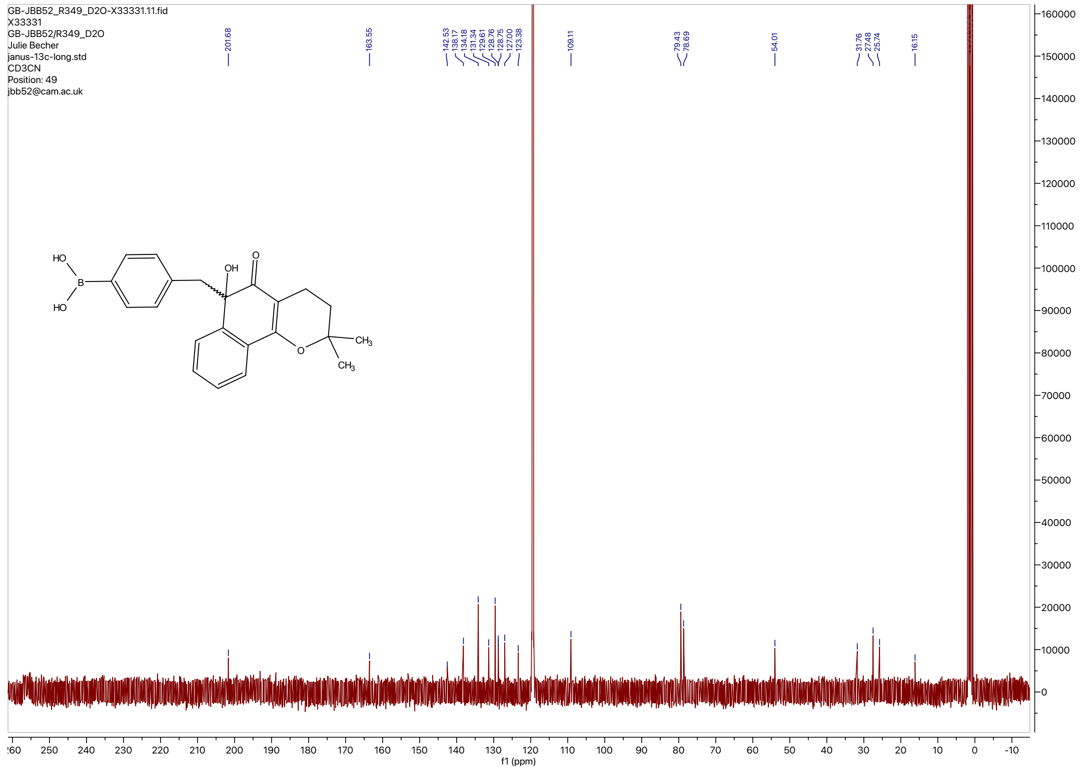

^{13}C NMR (101 MHz, 1:1 D_2O:CD_3CN) of 26.

Section 13. References

(1) Liu, F.; Li, Y.; Li, Y.; Wang, Z.; Li, X.; Liu, Y.; Zhao, Y. Triggering Multiple Modalities of Cell Death *via* Dual-Responsive Nanomedicines to Address the Narrow Therapeutic Window of *β*-Lapachone. *J. Colloid Interface Sci.* **2025**, *678*, 915–924. https://doi.org/10.1016/j.jcis.2024.09.063.

(2) Bey, E. A.; Bentle, M. S.; Reinicke, K. E.; Dong, Y.; Yang, C.-R.; Girard, L.; Minna, J. D.; Bornmann, W. G.; Gao, J.; Boothman, D. A. An NQO1- and PARP-1-Mediated Cell Death Pathway Induced in Non-Small-Cell Lung Cancer Cells by β-Lapachone. *Proc. Natl. Acad. Sci. U. S. A.* **2007**, *104* (28), 11832–11837. https://doi.org/10.1073/pnas.0702176104.

(3) Bentle, M. S.; Bey, E. A.; Dong, Y.; Reinicke, K. E.; Boothman, D. A. New Tricks for Old Drugs: The Anticarcinogenic Potential of DNA Repair Inhibitors. *J. Mol. Histol.* **2006**, *37* (5–7), 203–218. https://doi.org/10.1007/s10735-006-9043-8.

(4) Silvers, M. A.; Deja, S.; Singh, N.; Egnatchik, R. A.; Sudderth, J.; Luo, X.; Beg, M. S.; Burgess, S. C.; DeBerardinis, R. J.; Boothman, D. A.; Merritt, M. E. The NQO1 Bioactivatable Drug, β-Lapachone, Alters the Redox State of NQO1+ Pancreatic Cancer Cells, Causing Perturbation in Central Carbon Metabolism. *J. Biol. Chem.* **2017**, *292* (44), 18203–18216. https://doi.org/10.1074/jbc.M117.813923.

(5) Beg, M. S.; Huang, X.; Silvers, M. A.; Gerber, D. E.; Bolluyt, J.; Sarode, V.; Fattah, F.; Deberardinis, R. J.; Merritt, M. E.; Xie, X.-J.; Leff, R.; Laheru, D.; Boothman, D. A. Using a Novel NQO1 Bioactivatable Drug, Beta-Lapachone (ARQ761), to Enhance Chemotherapeutic Effects by Metabolic Modulation in Pancreatic Cancer. *J. Surg. Oncol.* **2017**, *116* (1), 83–88. https://doi.org/10.1002/jso.24624.

(6) Rodrigues, T.; Werner, M.; Roth, J.; Cruz, E. H. G. da; Marques, M. C.; Akkapeddi, P.; Lobo, S. A.; Koeberle, A.; Corzana, F.; Júnior, E. N. da S.; Werz, O.; Bernardes, G. J. L. Machine Intelligence Decrypts β-Lapachone as an Allosteric 5-Lipoxygenase Inhibitor. *Chem. Sci.* **2018**, *9* (34), 6899–6903. https://doi.org/10.1039/C8SC02634C.

(7) Zhang, J.; Xu, Q.; Ma, D. Inhibition of Thioredoxin Reductase by Natural Anticancer Candidate β-Lapachone Accounts for Triggering Redox Activation-Mediated HL-60 Cell Apoptosis. *Free Radic. Biol. Med.* **2022**, *180*, 244–252. https://doi.org/10.1016/j.freeradbiomed.2022.01.019.

(8) Dunsmore, L.; Navo, C. D.; Becher, J.; de Montes, E. G.; Guerreiro, A.; Hoyt, E.; Brown, L.; Zelenay, V.; Mikutis, S.; Cooper, J.; Barbieri, I.; Lawrinowitz, S.; Siouve, E.; Martin, E.; Ruivo, P. R.; Rodrigues, T.; da Cruz, F. P.; Werz, O.; Vassiliou, G.; Ravn, P.; Jiménez-Osés, G.; Bernardes, G. J. L. Controlled Masking and Targeted Release of Redox-Cycling Ortho-Quinones via a C–C Bond-Cleaving 1,6-Elimination. *Nat. Chem.* **2022**, *14* (7), 754–765. https://doi.org/10.1038/s41557-022-00964-7.

(9) M. J. Frisch, G. W. Trucks, H. B. Schlegel,; G. E. Scuseria, M. A. Robb, J. R. Cheeseman,; G. Scalmani, V. Barone, G. A. Petersson, H. Nakatsuji,; X. Li, M. Caricato, A. V. Marenich, J. Bloino, B. G. Janesko,; R. Gomperts, B. Mennucci, H. P. Hratchian, J. V. Ortiz,; A. F. Izmaylov, J. L. Sonnenberg, D. Williams-Young, F. Ding, F. Lipparini, F. Egidi,; J. Goings, B. Peng, A. Petrone, T. Henderson, D. Ranasinghe,; V. G. Zakrzewski, J. Gao, N. Rega, G. Zheng,; W. Liang, M. Hada, M. Ehara, K. Toyota,; R. Fukuda, J.

Hasegawa, M. Ishida, T. Nakajima, Y. Honda,; O. Kitao, H. Nakai, T. Vreven, K. Throssell,; J. A. Montgomery, Jr., J. E. Peralta,; F. Ogliaro, M. J. Bearpark, J. J. Heyd, E. N. Brothers,; K. N. Kudin, V. N. Staroverov, T. A. Keith,; R. Kobayashi, J. Normand, K. Raghavachari, A. P. Rendell,; J. C. Burant, S. S. Iyengar, J. Tomasi, M. Cossi,; J. M. Millam, M. Klene, C. Adamo, R. Cammi,; J. W. Ochterski, R. L. Martin, K. Morokuma,; O. Farkas, J. B. Foresman, and D. J. Fox. Gaussian 16 Rev. C.01, 2016.

(10) Zhao, Y.; Truhlar, D. G. The M06 Suite of Density Functionals for Main Group Thermochemistry, Thermochemical Kinetics, Noncovalent Interactions, Excited States, and Transition Elements: Two New Functionals and Systematic Testing of Four M06-Class Functionals and 12 Other Functionals. *Theor. Chem. Acc.* **2008**, *120* (1), 215–241. https://doi.org/10.1007/s00214-007-0310-x.

(11) Scalmani, G.; Frisch, M. J. Continuous Surface Charge Polarizable Continuum Models of Solvation. I. General Formalism. *J. Chem. Phys.* **2010**, *132* (11), 114110. https://doi.org/10.1063/1.3359469.

(12) Ribeiro, R. F.; Marenich, A. V.; Cramer, C. J.; Truhlar, D. G. Use of Solution-Phase Vibrational Frequencies in Continuum Models for the Free Energy of Solvation. *J. Phys. Chem. B* **2011**, *115* (49), 14556–14562. https://doi.org/10.1021/jp205508z.

(13) Hratchian, H. P.; Schlegel, H. B. Accurate Reaction Paths Using a Hessian Based Predictor–Corrector Integrator. *J. Chem. Phys.* **2004**, *120* (21), 9918–9924. https://doi.org/10.1063/1.1724823.

(14) Hratchian, H. P.; Schlegel, H. B. Using Hessian Updating To Increase the Efficiency of a Hessian Based Predictor-Corrector Reaction Path Following Method. *J. Chem. Theory Comput.* **2005**, *1* (1), 61–69. https://doi.org/10.1021/ct0499783.

(15) Rose, D. A.; Treacy, J. W.; Yang, Z. J.; Ko, J. H.; Houk, K. N.; Maynard, H. D. Self-Immolative Hydroxybenzylamine Linkers for Traceless Protein Modification. *J. Am. Chem. Soc.* **2022**, *144* (13), 6050–6058. https://doi.org/10.1021/jacs.2c01136.

(16) Antunes, I. F.; Haisma, H. J.; Elsinga, P. H.; Dierckx, R. A.; de Vries, E. F. J. Synthesis and Evaluation of [18F]-FEAnGA as a PET Tracer for Beta-Glucuronidase Activity. *Bioconjug. Chem.* **2010**, *21* (5), 911–920. https://doi.org/10.1021/bc9004602.

(17) Smith, D. G. A.; Burns, L. A.; Simmonett, A. C.; Parrish, R. M.; Schieber, M. C.; Galvelis, R.; Kraus, P.; Kruse, H.; Di Remigio, R.; Alenaizan, A.; James, A. M.; Lehtola, S.; Misiewicz, J. P.; Scheurer, M.; Shaw, R. A.; Schriber, J. B.; Xie, Y.; Glick, Z. L.; Sirianni, D. A.; O'Brien, J. S.; Waldrop, J. M.; Kumar, A.; Hohenstein, E. G.; Pritchard, B. P.; Brooks, B. R.; Schaefer, H. F., III; Sokolov, A. Yu.; Patkowski, K.; DePrince, A. E., III; Bozkaya, U.; King, R. A.; Evangelista, F. A.; Turney, J. M.; Crawford, T. D.; Sherrill, C. D. PSI4 1.4: Open-Source Software for High-Throughput Quantum Chemistry. *J. Chem. Phys.* **2020**, *152* (18), 184108. https://doi.org/10.1063/5.0006002.

(18) *Rowan Scientific*. Rowan Scientific. https://www.rowansci.com (accessed 2025-10-02).

(19) Nair, V.; Jayan, C. N.; Ros, S. Novel Reactions of Indium Reagents with 1,2-Diones: A Facile Synthesis of α-Hydroxy Ketones. *Tetrahedron* **2001**, *57* (46), 9453–9459. https://doi.org/10.1016/S0040-4020(01)00937-1.

(20) Winn, J. S. *Physical Chemistry*; HarperCollins College Publishers, 1995.

(21) Inagaki, R.; Ninomiya, M.; Tanaka, K.; Koketsu, M. Synthesis, Characterization, and Antileukemic Properties of Naphthoquinone Derivatives of Lawsone. *ChemMedChem* **2015**, *10* (8), 1413–1423. https://doi.org/10.1002/cmdc.201500189.

(22) Jiang, Z.; Hogeland, J. Synthesis of Beta-Lapachone and Its Intermediates, August 1, 2002. https://patentscope.wipo.int/search/en/WO2002059103 (accessed 2025-03-18).

(23) Di Chenna, P. H.; Benedetti-Doctorovich, V.; Baggio, R. F.; Garland, M. T.; Burton, G. Preparation and Cytotoxicity toward Cancer Cells of Mono(Arylimino) Derivatives of β-Lapachone. *J. Med. Chem.* **2001**, *44* (15), 2486–2489. https://doi.org/10.1021/jm010050u.

(24) Walther, R.; Olesen, M. T. J.; Zelikin, A. N. Extended Scaffold Glucuronides: En Route to the Universal Synthesis of O-Aryl Glucuronide Prodrugs. *Org. Biomol. Chem.* **2019**, *17* (29), 6970–6974. https://doi.org/10.1039/C9OB01384A.

(25) Lioux, T.; Vernejoul, F.; Boularan, C.; Tiraby, M. Pro-Cyclic Dinucleotides and Pro-Cyclic Dinucleotide Conjugates for Cytokine Induction. WO2019129880 (A1), 2019. https://worldwide.espacenet.com/publicationDetails/biblio?FT=D&date=20190704&DB=&locale=en_EP&CC=WO&NR=2019129880A1&KC=A1&ND=1 (accessed 2020-08-06).

(26) Lyon, R. P.; Bovee, T. D.; Doronina, S. O.; Burke, P. J.; Hunter, J. H.; Neff-LaFord, H. D.; Jonas, M.; Anderson, M. E.; Setter, J. R.; Senter, P. D. Reducing Hydrophobicity of Homogeneous Antibody-Drug Conjugates Improves Pharmacokinetics and Therapeutic Index. *Nat. Biotechnol.* **2015**, *33* (7), 733–735. https://doi.org/10.1038/nbt.3212.

(27) Sanchini, S.; Perruccio, F.; Piizzi, G. Rational Design, Synthesis and Biological Evaluation of Modular Fluorogenic Substrates with High Affinity and Selectivity for PTP1B. *ChemBioChem* **2014**, *15* (7), 961–976. https://doi.org/10.1002/cbic.201400033.

(28) Doura, T.; Takahashi, K.; Ogra, Y.; Suzuki, N. Combretastatin A4-β-Galactosyl Conjugates for Ovarian Cancer Prodrug Monotherapy. *ACS Med. Chem. Lett.* **2017**, *8* (2), 211–214. https://doi.org/10.1021/acsmedchemlett.6b00427.

(29) Wrobel, J.; Green, D.; Jetter, J.; Kao, W.; Rogers, J.; Claudia Pérez, M.; Hardenburg, J.; Deecher, D. C.; López, F. J.; Arey, B. J.; Shen, E. S. Synthesis of (Bis)Sulfonic Acid, (Bis)Benzamides as Follicle-Stimulating Hormone (FSH) Antagonists. *Bioorg. Med. Chem.* **2002**, *10* (3), 639–656. https://doi.org/10.1016/S0968-0896(01)00324-8.

(30) Duan, H.; Zheng, J.; Lai, Q.; Liu, Z.; Tian, G.; Wang, Z.; Li, J.; Shen, J. 2-Phenylquinazolin-4(3*H*)-One, a Class of Potent PDE5 Inhibitors with High Selectivity versus PDE6. *Bioorg. Med. Chem. Lett.* **2009**, *19* (10), 2777–2779. https://doi.org/10.1016/j.bmcl.2009.03.125.

(31) Pezzato, C.; Nguyen, M. T.; Cheng, C.; Kim, D. J.; Otley, M. T.; Stoddart, J. F. An Efficient Artificial Molecular Pump. *Tetrahedron* **2017**, *73* (33), 4849–4857. https://doi.org/10.1016/j.tet.2017.05.087.

(32) Wei, P.; Xue, F.; Shi, Y.; Strand, R.; Chen, H.; Yi, T. A Fluoride Activated Methylene Blue Releasing Platform for Imaging and Antimicrobial Photodynamic Therapy of Human Dental Plaque. *Chem. Commun.* **2018**, *54* (93), 13115–13118. https://doi.org/10.1039/C8CC07410K.

(33) Hayashi, T.; Ohmori, K.; Suzuki, K. Synthetic Study on Carthamin: Problem and Solution for Oxidative Dearomatization Approach to Quinol C-Glycoside. *Synlett* **2016**, *27*, 2345–2351. https://doi.org/10.1055/s-0035-1562511.

(34) Ohsawa, K.; Yoshida, M.; Doi, T. A Direct and Mild Formylation Method for Substituted Benzenes Utilizing Dichloromethyl Methyl Ether–Silver Trifluoromethanesulfonate. *J. Org. Chem.* **2013**, *78* (7), 3438–3444. https://doi.org/10.1021/jo400056k.

(35) Kaburagi, Y.; Kishi, Y. Operationally Simple and Efficient Workup Procedure for TBAF-Mediated Desilylation: Application to Halichondrin Synthesis. *Org. Lett.* **2007**, *9* (4), 723–726. https://doi.org/10.1021/ol063113h.

(36) Landi Jr, J. J.; and Ramig, K. Regioselective Preparation of 4-Formyl-3,5-Dimethoxyphenol, an Intermediate in the Synthesis of the PAL Solid-Phase Peptide Synthesis Handle. *Synth. Commun.* **1991**, *21* (2), 167–171. https://doi.org/10.1080/00397919108020808.

(37) Holz, J.; Pfeffer, C.; Zuo, H.; Beierlein, D.; Richter, G.; Klemm, E.; Peters, R. In Situ Generated Gold Nanoparticles on Active Carbon as Reusable Highly Efficient Catalysts for a C −C Stille Coupling. *Angew. Chem. Int. Ed.* **2019**, *58* (30), 10330–10334. https://doi.org/10.1002/anie.201902352.

(38) Lumba, M. A.; Willis, L. M.; Santra, S.; Rana, R.; Schito, L.; Rey, S.; Wouters, B. G.; Nitz, M. A β-Galactosidase Probe for the Detection of Cellular Senescence by Mass Cytometry. *Org. Biomol. Chem.* **2017**, *15* (30), 6388–6392. https://doi.org/10.1039/C7OB01227F.

(39) Patel, A.; Richardson, A. C. 3-Methoxy-4-(2-Nitrovinyl)Phenyl Glycosides as Potential Chromogenic Substrates for the Assay of Glycosidases. *Carbohydr. Res.* **1986**, *146* (2), 241–249. https://doi.org/10.1016/0008-6215(86)85043-1.

(40) Boghaert, E. R.; Ackler, S. L.; Tao, Z.-F.; Wang, X.; Doherty, G.; MARIN, V. L.; Sullivan, G. M.; Song, X.; Kunzer, A. R.; Welch, D. S.; Bruncko, M.; Judd, A. S.; Souers, A. J. Antibody Drug Conjugates with Cell Permeable Bcl-Xl Inhibitors. WO2016094505A1, June 16, 2016. https://patents.google.com/patent/WO2016094505A1/en (accessed 2025-03-18).

(41) Caygill, G. B.; Larsen, D. S.; Brooker, S. Total Synthesis of (+)-Hatomarubigin B. *J. Org. Chem.* **2001**, *66* (22), 7427–7431. https://doi.org/10.1021/jo015839r.

(42) Beasley, S.; Nguyen, K.; Fazio, M.; Spitale, R. C. Protected Pyrimidine Nucleosides for Cell-Specific Metabolic Labeling of RNA. *Tetrahedron Lett.* **2018**, *59* (44), 3912–3915. https://doi.org/10.1016/j.tetlet.2018.09.040.

(43) Liu, L.; Tang, M.; Pragani, R.; Whitby, F. G.; Zhang, Y.; Balakrishnan, B.; Fang, Y.; Karavadhi, S.; Tao, D.; LeClair, C. A.; Hall, M. D.; Marugan, J. J.; Boxer, M.; Shen, M.; Hill, C. P.; Lai, K.; Patnaik, S. Structure-Based Optimization of Small Molecule Human Galactokinase Inhibitors. *J. Med. Chem.* **2021**, *64* (18), 13551–13571. https://doi.org/10.1021/acs.jmedchem.1c00945.

(44) Gong, Q.; Li, X.; Li, T.; Wu, X.; Hu, J.; Yang, F.; Zhang, X. A Carbon-Carbon Bond Cleavage-Based Prodrug Activation Strategy Applied to β-Lapachone for Cancer-Specific Targeting. *Angew. Chem. Int. Ed.* **2022**, *61* (40), e202210001. https://doi.org/10.1002/anie.202210001.
